# Simple Sequence Repeat (SSR) Genetic Linkage Map of D Genome Diploid Cotton Derived from an Interspecific Cross between *Gossypium davidsonii* and *Gossypium klotzschianum*

**DOI:** 10.3390/ijms19010204

**Published:** 2018-01-11

**Authors:** Joy Nyangasi Kirungu, Yanfeng Deng, Xiaoyan Cai, Richard Odongo Magwanga, Zhongli Zhou, Xingxing Wang, Yuhong Wang, Zhenmei Zhang, Kunbo Wang, Fang Liu

**Affiliations:** 1 State Key Laboratory of Cotton Biology/Institute of Cotton Research, Chinese Academy of Agricultural Sciences, Anyang 455000, China; joynk@cricaas.com.cn (J.N.K.); dengyf@cricaas.com.cn (Y.D.); caixy@cricaas.com.cn (X.C.); richard@cricaas.com.cn (R.O.M.); zhouzl@cricaas.com.cn (Z.Z.); wangxx@cricaas.com.cn (X.W.); wangyh@cricaas.com.cn (Y.W.); zhangzm@cricaas.com.cn (Z.Z.); 2 School of Biological and Physical Sciences (SBPS), Main Campus, Jaramogi Oginga Odinga University of Science and Technology (JOOUST), Main Campus, P.O. Box 210-40601 Bondo, Kenya

**Keywords:** wild cotton spp, genetic map, polymorphic primers, null alleles

## Abstract

The challenge in tetraploid cotton cultivars is the narrow genetic base and therefore, the bottleneck is how to obtain interspecific hybrids and introduce the germplasm directly from wild cotton to elite cultivars. Construction of genetic maps has provided insight into understanding the genome structure, interrelationships between organisms in relation to evolution, and discovery of genes that carry important agronomic traits in plants. In this study, we generated an interspecific hybrid between two wild diploid cottons, *Gossypium davidsonii* and *Gossypium klotzschianum*, and genotyped 188 F_2:3_ populations in order to develop a genetic map. We screened 12,560 SWU Simple Sequence Repeat (SSR) primers and obtained 1000 polymorphic markers which accounted for only 8%. A total of 928 polymorphic primers were successfully scored and only 728 were effectively linked across the 13 chromosomes, but with an asymmetrical distribution. The map length was 1480.23 cM, with an average length of 2.182 cM between adjacent markers. A high percentage of the markers on the map developed, and for the physical map of *G. raimondii,* exhibited highly significant collinearity, with two types of duplication. High level of segregation distortion was observed. A total of 27 key genes were identified with diverse roles in plant hormone signaling, development, and defense reactions. The achievement of developing the F_2:3_ population and its genetic map constructions may be a landmark in establishing a new tool for the genetic improvement of cultivars from wild plants in cotton. Our map had an increased recombination length compared to other maps developed from other D genome cotton species.

## 1. Introduction

Wild cotton species have been well studied in terms of their variation in desired traits, such as chemical defenses [1], oil content [2], and fibre production [3,4]. With cotton being the backbone of the textile industries, its continued production with enhanced fibre quality is necessary for the sustainability of the sector [5,6]. The production of cotton is ever-declining due to abiotic and biotic stress: currently the loss in cotton production is estimated to stand at 30% as a result of stress effects [7]. This is projected to continue further due to available lands being highly polluted with increased salinity, and is estimated to cover more than 6% of the world’s arable land [8]. In light of this, breeding of tolerant cotton genotypes is important for the existence of the textile industries. Elite cultivated cotton has a narrow genetic base with ever-declining hybrid vigor due to inbreeding [9]. For the continuation of cotton production, increased vigor and broadening of the genetic base is essential. The use of wild progenitors will help to break the bottleneck [10]. The direct use of wild cotton species in improving the elite tetraploid cotton, which has a small amounts of variation due to the specie’s monophyletic origin and domestication compounded by intense selection, has proved futile through conventional breeding [4]. Wild cotton species have immense genetic diversity with great potential to improve many important agronomic traits, such as resistance to disease, fibre qualities, and abiotic stress tolerance [11,12,13]. The valuable agronomic traits in wild cotton progenitors can be exploited effectively to improve cultivated cotton cultivars and solve many problems associated with fibre quality, resistance to insects, pathogens, and tolerance to abiotic stress [11,14].

The majority of elite cotton (*Gossypium hirsutum* L.) varieties have been either bred from cultivars with common ancestry or are known to have related ancestors, but so far only limited increases in productivity have been obtained [15]. The use of D-genome cotton as a source of genetic diversity for gaining stress tolerance, fibre quality, and earlier maturity has been suggested [7,11,16]. *G. davidsonii* and *G. klotzschianum* are both wild diploid cotton species which are endemic to the Sonoran Desert of north-western Mexico [17]. The two cotton species are found in the subsection of Integrifolia within the genus *Gossypium* (Family Malvaceae) [17]. They are known to exhibit several characteristic traits such as salt tolerance, and resistance to sucking pests and bacterial blight [18,19].

Genetic linkage maps based on DNA markers are essential tools for plant molecular research in areas such as marker-assisted selection, quantitative trait loci (QTL) mapping, and map-based cloning. A number of DNA markers have been used in the development of genetic linkage maps for cotton [20,21,22,23,24]. The genetic maps have been used to map many beneficial agronomical genes and/or quantitative trait loci (QTLs). Several comparative maps using a common set of amplified fragment length polymorphisms (AFLP), restriction fragment length polymorphisms (RFLP), or SSR markers have been produced for cotton [25,26]. Interspecific linkage maps of diploid cottons have been constructed for the D genome (*G. raimondii* × *G. trilobum*), A genome (*G. arboretum* × *G. herbaceum*), [25,27] and G genome (*G. australe* × *G. nelsonii*) [28]. However, maps derived from wild progenitors are still lacking.

Marker-assisted selection (MAS) is the most recent innovation in molecular breeding for crop improvement [29]. MAS employs the technique of molecular markers in detecting or mining valuable traits in mapping various populations. The markers are developed based on DNA variation among individuals in a breeding population. In contrast to phenotypic selection, MAS is a direct form of genotype selection [30]. The application of markers derived from a specific genome provides the opportunity to detect the inherent traits of a given genotype [31]. SWU markers are recently developed SSR markers from the D genome of *Gossypium raimondii*. To date, no genetic map has been developed from mono-markers of the D genome in cotton. The application of mono-markers provides the best approach for examining genetic variability in a pure line developed from a given genome, as evidenced from SSR markers developed in barley [32]. So far, no comprehensive SSR-based interspecific linkage maps of F_2:3_ populations derived from two wild cotton generations of D genome in *Gossypium* with closer genetic relatedness have been reported. We managed to develop an F_2:3_ generation through the interspecific cross of two wild cotton species, *G. davidsonii* and *G. klotzschianum,* which we employed in developing the genetic map. This study aimed to genotype the F_2:3_ populations and develop fine linkage maps with broader applications in molecular studies, with the net effect of breeding for an enhanced or broader genetic base.

## 2. Results

### 2.1. Parental Polymorphism

A total of 12,560 SWU SSRs markers derived from *G. raimondii* were used to screen the F_2:3_ interspecific populations formed using two wild cotton species of D genome: *G. davidsonii* (accession number PI 530809) and *G. klotzschianum* (accession number PI 499748). Among them, 1000 markers were found to be polymorphic, accounting for only 8% of the total markers used. A total of 728 loci were generated by 1000 polymorphic primer pairs. The number of polymorphic dominant loci identified was 200 (27.17%), while 528 (72.83%) non-dominant loci were identified (Table 1).

### 2.2. Linkage Analysis and Map Construction

**Figure 1:**
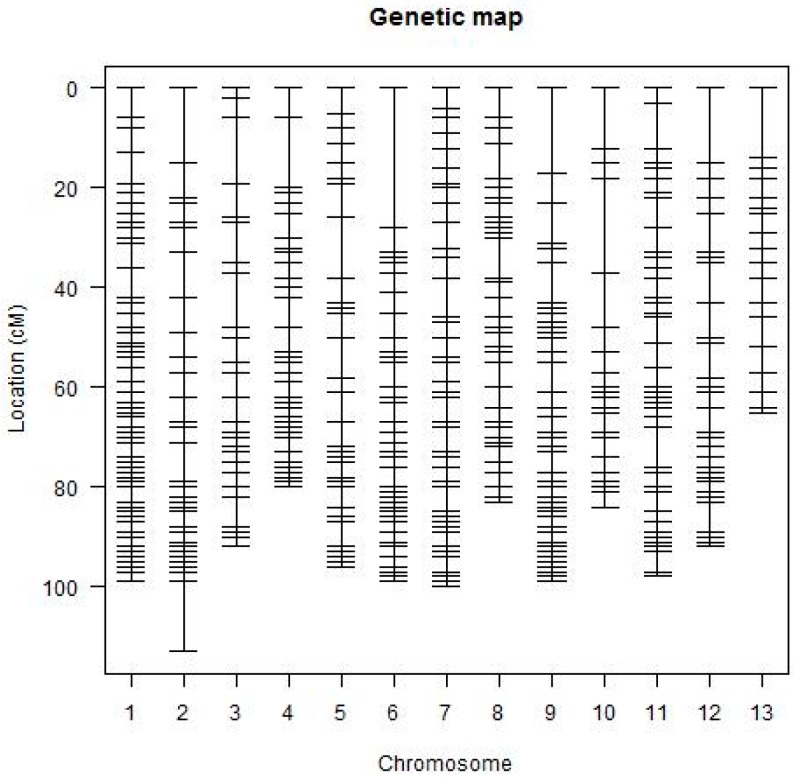
Genetic map constructed using the F_2:3_ Population derived from the parental lines. The visual presentation was analysed by R software.

A total of 728 segregating loci were assembled into 13 linkage groups (Figure 1). Two hundred (200) loci remained unlinked to any of the generated linkage groups (LGs). The total length of the map was 1480.225 cM. The average genetic distance between two loci was 2.011 cM, while the largest gap was 36.597 cM between adjacent loci on LG11 (Chr11), and the smallest gap was 0.002 cM on LG06 (Chr06). The largest linkage group consisted of 89 marker loci covering 116.045 cM in LG1 (Chr01), and the smallest was in linkage group LG10 (Chr10) consisting of 34 marker loci covering only 101.93 cM (Figure 1 and Table 2). All the markers were distributed evenly among the linkage groups without clustering of loci. The map obtained through mono-markers had similar attributes to earlier constructed D genome maps, and was composed of 763 loci and with map size of 1493.3 cM in 13 linkage groups [27]. This clearly shows a high level of success in the development of F_2:3_ populations from two closely related wild cotton genotypes, despite the difficulties faced in the development of the mapping population. The assigned chromosomes are provided in (Supplementary Figure S1).

**Figure 2:**
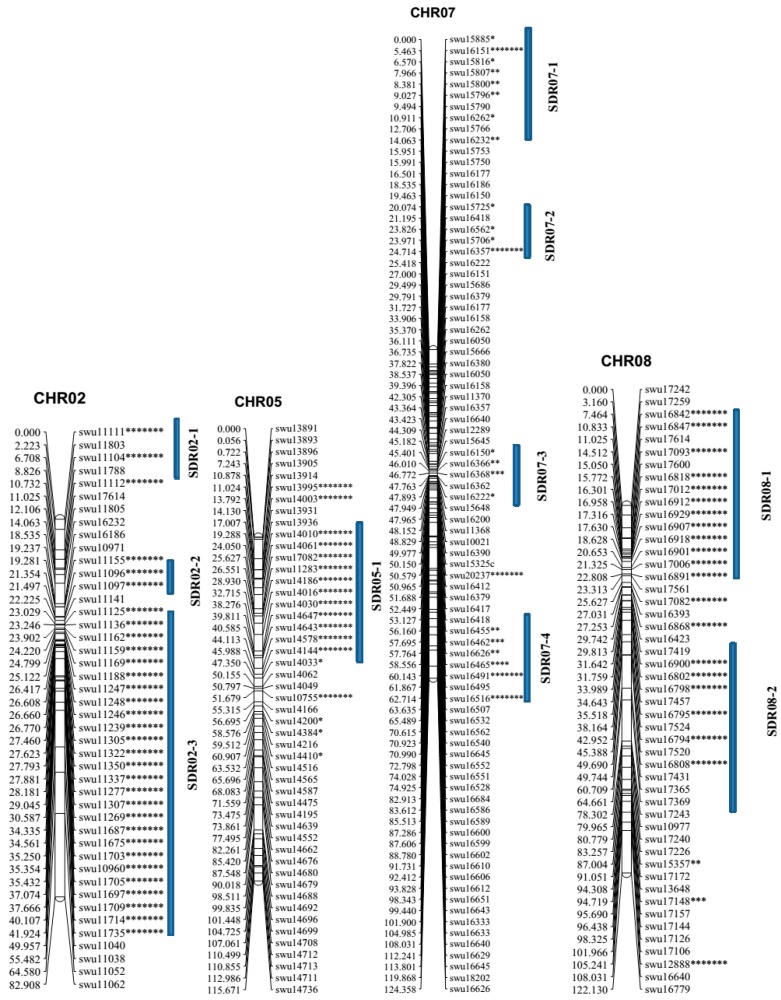
Linkage groups with the highest segregation distortion. Segregation distortion region (SDR); chromosome 2 has three regions, chromosome 5 has one region, chromosome 7 has four regions, and chromosome 8 has two regions. The marker positions are in centiMorgans (cM).

A total of 159 distorted loci were detected, which translates to (21.6%) of all the mapped loci. The loci showed significant deviation from the Mendelian ratios at *p* < 0.05 to *p* < 0.00005. The highest level of distortion was observed in two linkage groups with 35 distorted markers on each, accounting for 22.01% of the entire linkage map (Table 3 and Figure 2). Several large clusters of segregation distortions were detected in LG02 (35), LG07 (35), LG08 (25), LG05 (17), and LG09 (12). LG02, LG05. LG07, and LG08 had dominant loci with preferential transmission of *Gossypium klotzschianum* alleles in the F_2:3_ populations, while LG01, LG03, LG04, LG06, LG10, and LG12 showed preferential transmission of *Gossypium davidsonii* alleles in the F_2:3_ populations (Table 3). In general, the alleles from *Gossypium klotzschianum* were highly preferentially transferred to the F_2:3_ populations compared to *Gossypium davidsonii* alleles, as evidenced by levels of distortion segregation of 74 and 12, respectively (Table 3).

### 2.3. Collinearity Analysis

**Figure 3:**
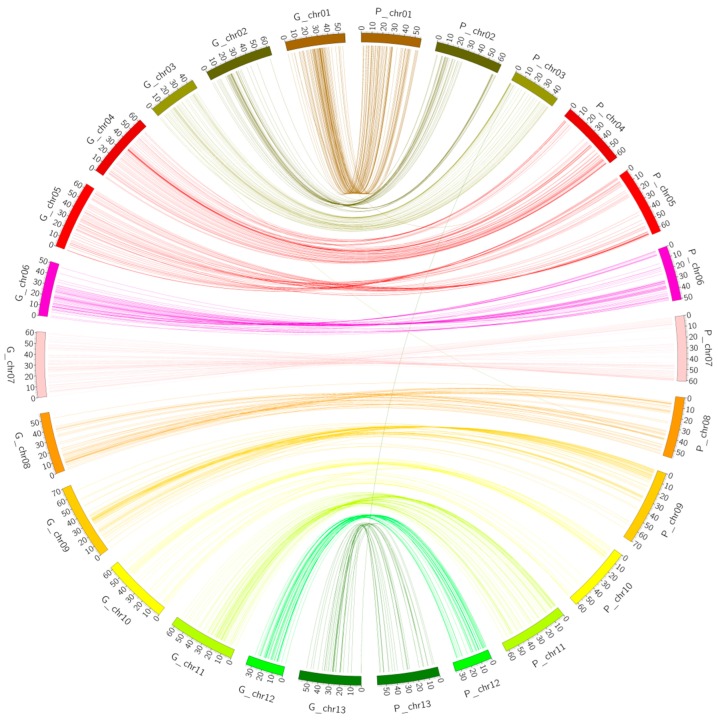
Collinearity between the genetic map and the physical map of diploid cotton.

**Figure 4:**
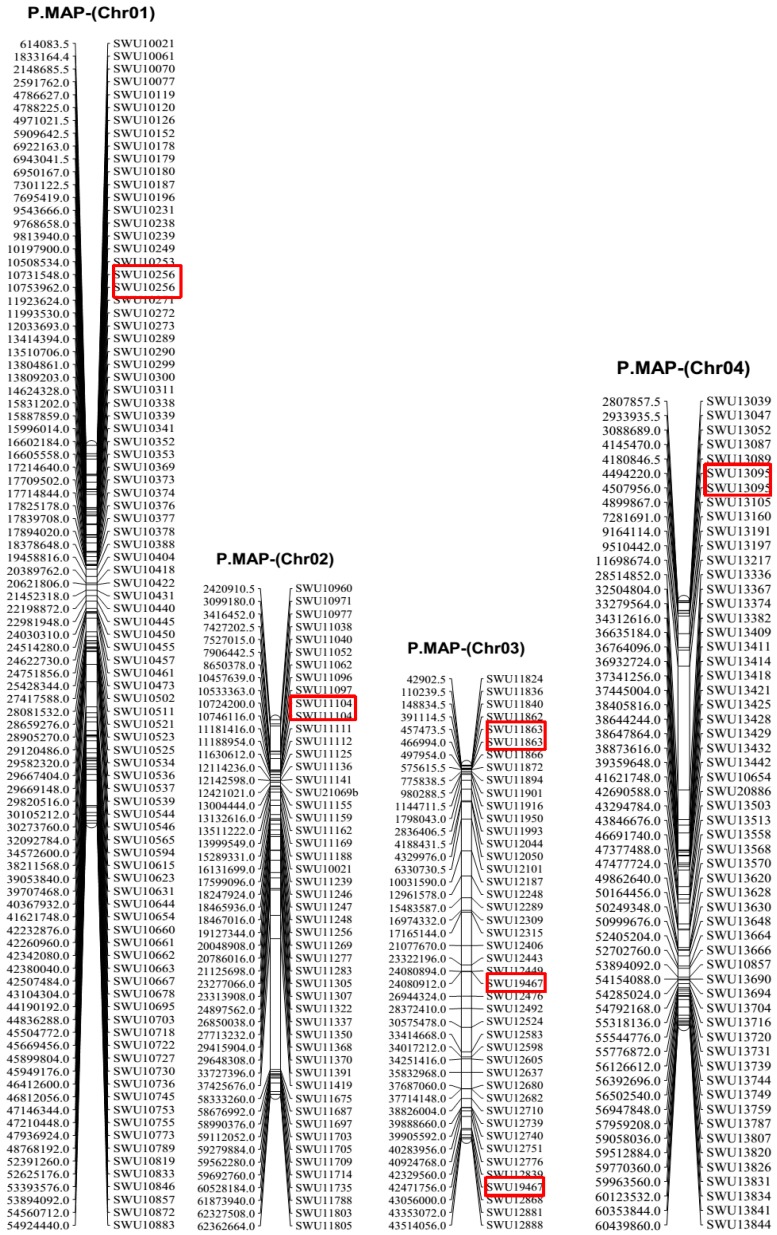
A section of physical map (P.MAP) with intra-chromosomal duplication. The red boxes indicate the duplicated markers. The marker positions are in base pairs (Bp).

Syntenic blocks were obtained by comparing the 728 markers’ mapped positions on both genetic map and the physical map using D cotton genome as the reference. The results showed that most of the markers had good collinearity (Figure 3). However, duplication was observed; two types of duplication were noted: intra- and inter-chromosomal duplication. The majority of the duplication was observed for inter-chromosomal as opposed to intra-chromosomal duplication. A total of 54 markers showed inter-chromosomal duplication while only 11 had intra-chromosomal duplication (Figure 4 and Supplementary Figure S2). Similar observations have been reported in the A genome, in which inter-chromosomal duplicated loci were recorded to be twice the number of intra-chromosomal duplicated loci [33].

### 2.4. Collinearity Analysis of the Genetic Map and the Physical Map of the (D_t_) Sub-Genome

**Figure 5:**
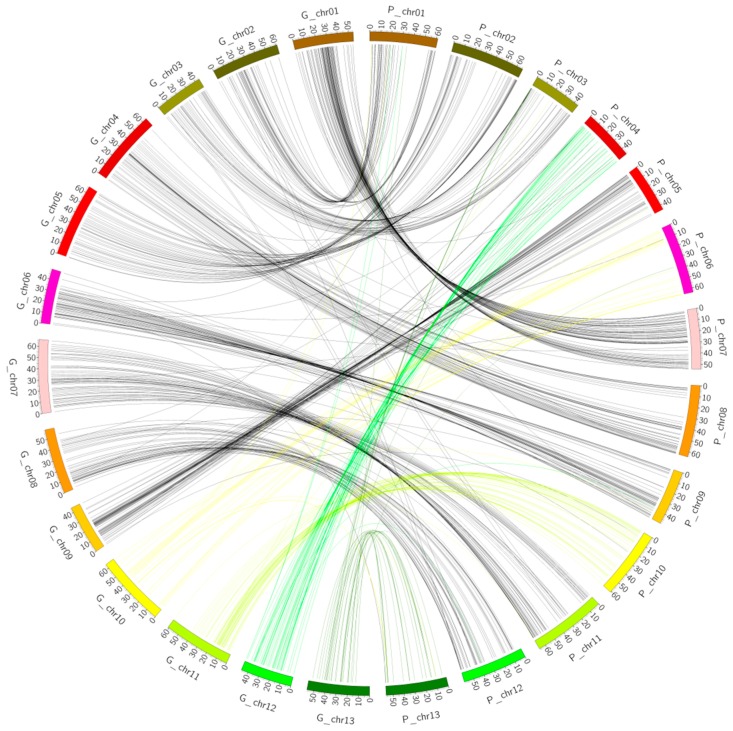
Collinearity between the genetic map and the physical map of the Dt sub-genome of *G. hirsutum*. The different line colors represent the various syntenic block regions between the chromosomes.

A total of 728 markers were blasted against the AD genome of *G. hirsutum* and 633 matches were obtained. Upon removal of the redundant markers, 599 markers were finally used in the collinearity analysis. The markers were used to analyze the collinearity blocks between the genetic map and the physical map of the Dt sub-genome of *G. hirsutum* (Figure 5 and Supplementary Table S1). The syntenic blocks showed good collinearity between the two maps.

### 2.5. Gene Mining, GO Functional Annotation, and Expression

**Figure 6:**
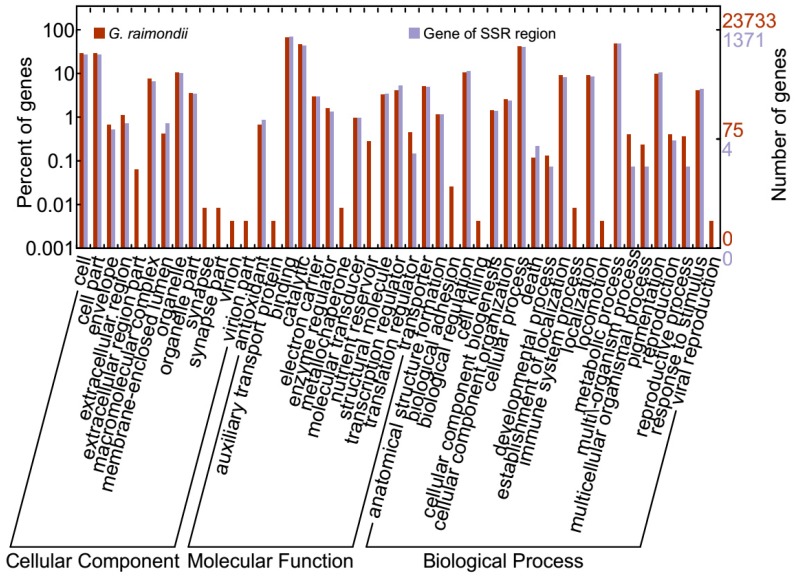
Gene ontology (GO) annotation results of the mined genes from the SSR derived genetic map of the D genome. GO analysis of 2063 protein sequences predicted their involvement in biological processes (BP), molecular functions (MF), and cellular components (CC).

**Figure 7:**
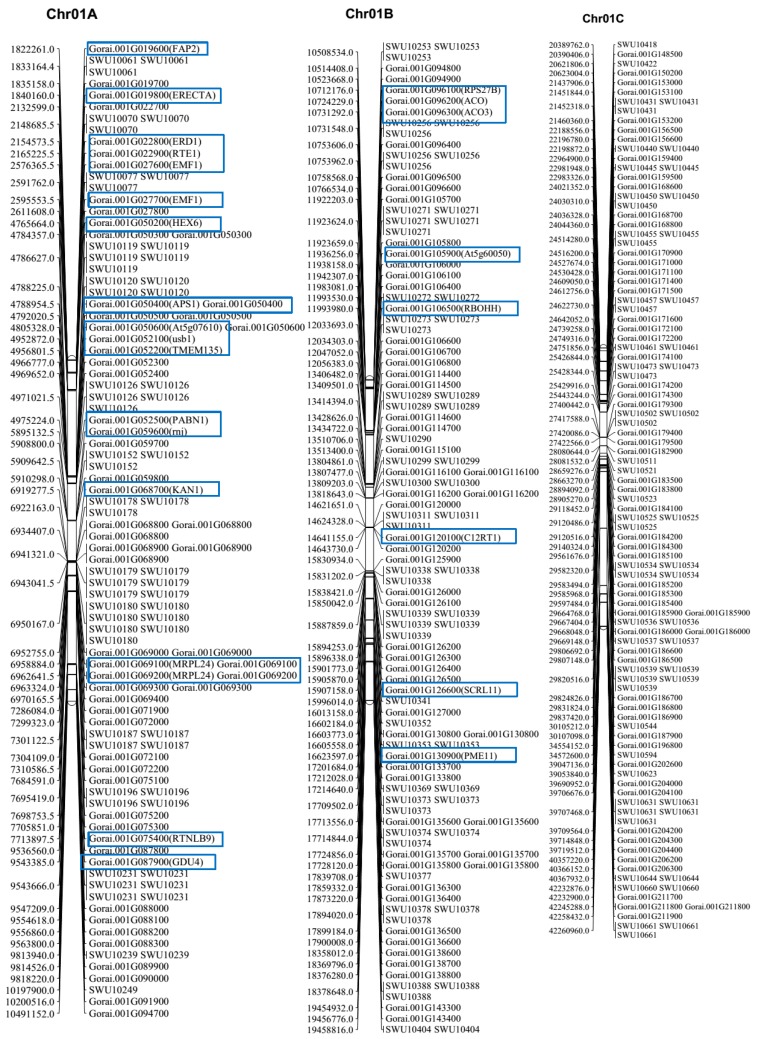
Physical map showing the exact locations of the identified key genes. The gene positions are in base pairs (bp). “SWU” are the SSR markers while the “Gorai” are the gene identities. The blue boxes indicate the key genes.

**Figure 8:**
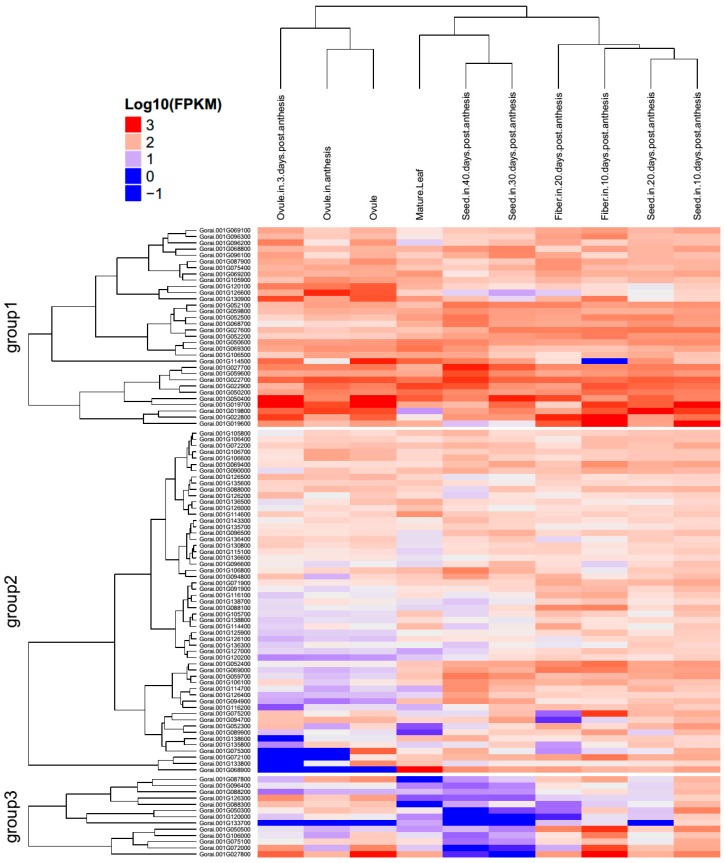
RNA sequence data analysis of 100 highly up regulated genes as per the RNA sequence. The heatmap was generated by log_10_ of the expression values. Colour coding indicates expression as illustrated in the key. *Y*-axis is the relative expression (2^−ΔΔ*C*t^).

A blast search of the regions 20 Kb up- and downstream of each SSR location was done and 2063 genes were obtained (Supplementary Table S2). The genes were distributed in all 13 chromosomes; the highest number of genes was found in chr09 with 269 genes, followed chr07 with 259 genes, while the least number of genes was detected in chr13 with only 58 genes. The rest of the chromosomes contained genes ranging from 100 to 200 in number (Supplementary Table S2). In the analysis of the physiochemical properties of the mined genes, their molecular weights ranged from 4.272 to 318.34 kDa, their charge ranged from −159.5 to 68.5, the Isoelectric Point (PI) values ranged from 3.562 to 12.546, and the Grand Average of Hydropathy (GRAVY) values ranged from −1.982 to 1.148, which implies that the genes were both hydrophobic and hydrophilic in nature. In addition, we analysed the gene structures in order to determine the intron–exon interactions. Out of 2063 genes, only 274 were not disrupted, which implies that these genes were highly conserved. The highest intron disruption was detected in Gorai.009G087200, with 49 introns (Supplementary Table S3); similarly high levels of intron disruption have also been recorded for cyclin dependent kinase (*CDK*) genes [34]. We further analysed gene features in order to determine the various descriptions of the mined genes; several classes of genes of interest observed were stress- and fibre-related genes. The stress-related genes detected were: heat shock protein, abscisic acid receptor, aluminum-activated malate transporter, calcium-dependent protein kinase, E3 ubiquitin-protein ligase, ethylene-responsive transcription factor, expansin, Myb-like protein, and mitogen-activated protein kinase, among others. In addition, we carried out gene ontology (GO) annotation. The GO terms describe the genes in relation to cellular components (CC), molecular functions (MF), and biological processes. All GO terms were detected. In cellular components, functions such as cell, cell part, extracellular regions, and membrane enclosed lumens, among others, were detected for the genes mined. Similarly, 13 functions were observed under molecular function, while the highest numbers of genes were found to be involved in biological processes (Figure 6). Finally, we carried out RNA seq. expression in order to validate the functions annotated from gene ontology. The RNA sequences were downloaded from the cotton genome database (https://cottonfgd.org/search/). The genes showed differential expression: more than 70% of the genes were found to be up regulated at fibre developmental stages at 10 and 20 DPA; in seed development at 10, 20, 30, and 40 DPA; in mature leaves, in ovules in anthesis; and 3 days post-anthesis (Supplementary Table S4). We selected the top 100 highly up regulated genes in the various tissues and constructed a heatmap based on their respective expression to levels (log_10_). The genes were categorized into three distinct groups. Group 1, had 32 genes that were significantly highly expressed, with expression levels of more than one. Among the 32 highly up regulated genes, *FAP2* (Fatty-acid-binding protein 2), with two GO functions: intramolecular lyase activity (GO:0016872-MF) and a cellular modified amino acid biosynthetic process (GO:0042398-CC); *ERECTA* (LRR receptor-like serine/threonine-protein kinase ERECTA) with seven GO functions such protein kinase activity (GO:0004672-MF), protein binding (GO:0005515-MF), ATP binding (GO:0005524-MF), protein phosphorylation (GO:0006468-MF), protein serine/threonine kinase activity (GO:0004674-MF), protein tyrosine kinase activity (GO:0004713-MF), and transferase activity, transferring phosphorus-containing groups (GO:0016772-MF); *EDR1* (Serine/threonine-protein kinase EDR1) with nine GO functions; *HEX6* (Hexose carrier protein HEX6) with one GO function; *RBOHH* (Putative respiratory burst oxidase homolog protein H) with 10 GO functions; *C12RT1* (Flavanone 7-*O*-glucoside-2″-*O*-beta-l-rhamnosyltransferase) with two GO functions; *SCRL11* (Putative defensin-like protein 244) with one GO function; and *PME11* (Putative pectinesterase 11) with three GO functions, could be the key genes responsible for fibre quality and other stress factor tolerance (Figure 7 and Supplementary Table S5). The second group, with 13 genes, was relatively down regulated in various tissues examined, while the third group, which was the majority with 55 genes, exhibited differential expression of both up- and down regulation. The expression profile is as illustrated in (Figure 8). As cotton is an important crop for fibre, seed development is critical for high fibre quality. The RNA seq. data provides clear indication that the genes mined within the SSR marker sequences have potential roles in both biotic stress and enhanced fibre quality.

## 3. Discussion

Simple sequence repeat (SSR) markers have gained significant use in plant genetics and breeding because of their multi-allelic nature, reproducibility, relative abundance, codominant inheritance, and good genome coverage [35]. SSRs are developed from genomic libraries, and can belong to either the transcribed region or the non-transcribed region of the genome [36,37].

In the present study, we explored the expressed sequence tag simple sequence repeat (EST-SSR) primers to access the nature of the F_2:3_ populations, which we developed from an interspecific cross between two wild cotton species of the D genome: *G. Klotzschianum* and *G. davidsonii*. Out of 12,500 SWU primers screened, only 1000 primers were found to be polymorphic, accounting for less than 10% of all the primers used. The result showed that these markers had relatively low levels of polymorphism in the F_2:3_ populations used. The low level of polymorphism in these markers indicates that EST-SSR primers are less polymorphic. The results obtained are in agreement with previous findings in which 54% and 83.8% polymorphism levels were detected in EST-SSRs and gSSRs in rice, respectively [38]. Similar results have also been obtained in wheat, in which 53% of gSSRs were found to be polymorphic compared to 25% of EST-SSRs [39]. Despite low polymorphism rates among the EST-SSRs, significant deviation has been observed in a high percentage of eSSRs, showing a relatively high polymorphism rate; for example, a 93.5% polymorphism rate was observed in an intraspecific cross between two genotypes of diploid *Actinidia chinensis* [40]. High polymorphism rates of 66% in EST-SSRs have also been found between the parents of rye grass and tall fescue populations [41]. In cotton, higher polymorphism rates have been recorded in *G. hirsutum* and *G. barbadense* gSSR markers (49%, 56%) than in EST-SSRs (18.2%, 19.8%, 23.3%, 23.9%, and 26%) [35,42,43,44]. Despite the low polymorphism in EST-SSR markers, EST-SSR markers still remain the markers of choice in plant genome studies due to their close association with genes of known function, high levels of transferability, codominant inheritance, and low cost [45,46].

In allotetraploid cotton map constructions a high number of dominant marker loci ranging from 29% to 67.8% have been reported [25]. A total of 106 dominant restriction fragment length polymorphism (RFLP) loci, accounting for 38.5% of an interspecific A diploid map, have been reported [25]. In the present study of the D genome interspecific map construction, 280 (28%) markers were scored as dominant; among them 200 (27.17%) were mapped. The high number of dominant loci in diploid cotton could be explained by the presence of non-functional copies of genes as a result of genetic mutation. These copies of genes are referred to as null alleles [47]. The null alleles have caused perennial challenges in population genetics as a result of the introduction and application of each new techniques of molecular assay such as microsatellites, protein electrophoresis, serological typing, and RFLPs [48]. The A, B, and O blood grouping in humans is the best example of a locus with a null allele: in people with the blood groups AO and BO, the O allele is a null allele that produces no phenotype because it is masked by the presence of the A or B alleles, which are co-dominant to each other. In plants or animals, a microsatellite null allele is a type of allele which consistently fails to amplify during a polymerase chain reaction (PCR), and therefore is not detected in the process of genotyping individuals or populations [48]. A number of studies have recorded the detection of null alleles: for example, in bread wheat a 45% rate of polymorphism was recorded due to presence of null alleles [49]. Similarly, 10% of eSSR primers detected 7–14 null alleles in bread wheat [50].

There are three major explanations for the occurrence of null alleles. The first is due to mutation—either deletion or substitution in the primer site [51]—which causes poor primer annealing during the PCR amplification process. Another possible cause for the occurrence of null alleles is the differential amplification of size-variant alleles [52]: short length alleles amplify more efficiently than larger ones, such that only the smaller of two alleles might be detected in a heterozygous individual. The third option could be due to PCR failure as a result of inconsistent DNA template quality or low template quantity.

Non-normalized segregation ratios have been observed in cotton [25]. In some cases, the distortion percentage is as high as 80%. This is similar to what was observed in this study, in which the highest segregation distortion was 76.087% in Chr02. Similar results have been observed in previous publications, and this high frequency of segregation distortion in interspecific crosses is believed to occur as a result of species divergence [53]. The map generated in this study is more detailed than other maps which have been generated from the D genome. The map was developed from mono-markers and the map features are coherent to other maps produced from high quality methods such as the use of RLFPs. High levels of segregation distortion (SD) are not only limited to maps developed from interspecific crosses; even in intraspecific crosses, the frequency of distorted ratios is also relatively high, ranging from 44.1% to 52.49% [54].

SD is a problem often encountered in mapping populations. In this work, the map generated exhibited the lowest observed SDs in the following linkage groups; LG01 (3.371%), LG04 (3.571%), LG10 (5.882%), LG11 (7.937%), and LG06 (8.621%); medium SDs were detected in LG13 (10.526%), LG12 (12.245), and LG09 (16.216); while the following LDs showed highest level of SDs; LG05 (34.694%), LG07 (40.698%), and LG02 (76.087%). A number of variables could have led to this kind of distortion, such as genetic drift, or cytological attributes such as pollen tube competition, mutation of the SSR binding site, and redundant heterozygotes, which are possible causes of segregation distortion. Pollen death, hitchhiking, gametophyte selection, preferential fertilization, and zygotic selection all result in segregation distortion [55]. Seven hundred and twenty-eight (728) of the mapped markers in the genetic map were syntenic at the chromosomal level with their corresponding chromosomes in the D genome. Marker duplication is a common feature in most maps of diploid cotton. The two types of duplication are inter- and intra-chromosomal duplication. In this study, 94 markers were found to be duplicated. Out of the duplicated markers, the majority showed inter-chromosomal duplication as opposed to intra-chromosomal duplication. The results obtained correlate positively with a number of reports in which markers have been found to be duplicated in diploid cotton with inter-chromosomal duplication constituting the majority [25]. Similar findings have been reported in other plants; for instance in maize, only one intra-chromosomal duplication has been reported on chromosome 8 [56]. Helentjaris et al. [57] reported there being little evidence supporting the existence of extensive intra-chromosomal duplication in maize. More duplicated loci were shared between the linkage group pairs LG03-LG13 (4), LG06-LG08 (3), LG07-LG08 (5), LG09-LG12 (7), and LG10-LG13 (2). Also, LG07 shared duplicated loci with LG01, LG02, LG06, LG08, LG09, LG11, and LG12. Twelve intra-chromosomal duplications were observed in LG01, LG02, LG03, LG04, LG05, LG06, LG07, LG08, LG09, LG12, and LG13. The two forms of duplication, inter- and intra-chromosomal, have been explained by a number of mechanisms, and therefore the occurrence of conserved syntenic blocks common between two chromosomes of a diploid genome or two chromosomes of a polyploid genome is evidence of a paleopolyploid [58]. Therefore, in this study, a locus shared by two or more linkage groups was anticipated. The diploid genomes of cotton are paleopolyploids as proven by evidence through cytogenetic, biochemical, and genetic mapping [58].

Cotton production has been on the decline due to effects of biotic and abiotic stresses, which have been aggravated due to narrow genetic base of elite tetraploid cotton [59]. To solve this, the important agronomic traits of wild progenitors can be introgressed into the cultivated cotton cultivars [4]. Therefore, based on the genetic map developed from this study, a total of 2063 genes were mined in reference to *G. raimondii*. Of significance were the genes responsible for fibre development, and abiotic and biotic stress tolerance. In relation to stress factors, various drought-related stress genes were found, including eight *NAC* gene members: *NAC007* (NAC domain-containing protein 7), *NAC073* (NAC domain-containing protein 73), *NAC083* (NAC domain-containing protein 83), *NAC086* (NAC domain-containing protein 86), *NAC091* (NAC domain-containing protein 91), *NAM-B2* (NAC transcription factor *NAM-B2*, and *ONAC010* (NAC transcription factor *ONAC010*). The plant-specific *NAC* family has been shown to regulate several biological processes in wheat. *NAC TFs* are known to be involved in processes such as senescence and nutrient remobilization [60], as well as responses to abiotic and biotic stresses, ranging from biotic stresses such as stripe rust [61] to abiotic stresses including drought and salt [62,63]. The mitogen-activated protein kinase (*MAPK*) signaling cascades have been reported to play a significant role in plant environment adaptation, growth, and development [64]. Four members of *MAPK* genes were mined, such as *MPK4* (Mitogen-activated protein kinase 4), *MMK1* (Mitogen-activated protein kinase homolog *MMK1*), *NPK1* (Mitogen-activated protein kinase *NPK1*), and *YDA* (Mitogen-activated protein kinase *YODA*). The MAPKs have been linked to drought, cold, and salt stress signal-associated cascades, such as *AtMEKK1*-*AtMKK1*/*AtMKK2*-*AtMPK4* [65], and *MEKK1*-*MKK4*/*5-MPK3*/6-*WRKY22*/*WRKY29* which is actively involved in plant innate immunity [66], *AtMKK2*-*AtMPK10 MAPK* has been found to be involved in the regulation of venation complexity by altering polar auxin transport efficiency [67]. Eighteen members of the serine/threonine-protein kinases were also found; these proteins have been found to have significant roles in plant cell growth, development, and oncogenesis [68]. Plant survival in ever-increasing environmental degradation has been associated with vital proteins known as ubiquitin carboxyl-terminal hydrolases. These proteins detects destructive molecules within the cell and either break or form compounds which are non-toxic to the plant. We identified three members of these proteins: *coq6* (Ubiquinone biosynthesis monooxygenase *coq6*, mitochondrial), *UBP15* (Ubiquitin carboxyl-terminal hydrolase 15), and *UBP8* (Ubiquitin carboxyl-terminal hydrolase 8). This group of proteins has been found to confer tolerance to drought and salt stress in *Brassica napus* [69].

In the RNA sequence expression of the first 100 highly upregulated genes, 32 genes were found to be significantly upregulated in the various tissues, for instance *FAP2* (Fatty-acid-binding protein 2) which is linked to heat stress tolerance in plants [70]. *ERECTA* (*LRR* receptor-like serine/threonine-protein kinase *ERECTA*) has been found to have roles in signaling and plant defense [71]. *FKBP42* (Peptidyl-prolyl cis-trans isomerase *FKBP42*) has been found to be induced by both abiotic and biotic stresses in maize [72], and by salt stress, heat shock, and cold shock in *Solanum tuberosum* [73]. *RBOHH* (Putative respiratory burst oxidase homolog protein H) has been found to have a crucial role in plant hormone signaling, development, and defense reactions [74]. The identification of these genes from the map constructed provides a platform for further use of the identified genes in improving the performance of elite tetraploid cotton. The D genome of cotton, has been found to be associated with various stable QTLs for physiological, morphological, and biochemical traits related to fibre qualities, and abiotic and biotic stress factors, when compared to the A genome as evident in the cotton QTL database (http://www2.cottonqtldb.org:8081/).

## 4. Materials and Methods

### 4.1. Plant Materials

*G. klotzschianum* (accession number PI 499748) as the female parent was crossed with *G. davidsonii* (accession number PI 530809) as the male parent to establish an F_1_ population. The F_1_ was then selfed to generate F_2:3_ progenies. *Gossypium davidsonii* has economically significant traits such as resistance to aphids, salinity, and bacterial blight [18], while *Gossypium klotzschianum* has traits for resistance to sucking pests [19]. The two parental materials and the F_2:3_ lines were developed at the National Wild Cotton Nursery in Sanya, Hainan Island, China.

### 4.2. DNA Extraction, Quantification, and Qualification

The CTAB method for DNA extraction was used for the extraction of DNA for the entire population and the two parental lines. [26]. Fresh and young leaves were collected from each line, then immediately frozen in liquid nitrogen to prevent DNA degradation before extraction. Each sample was then ground in liquid nitrogen into a fine powder, and CTAB mixture was then added immediately. For each 100 mg of the homogenized tissue, 500 µL of CTAB Extraction Buffer was used. Upon addition of the buffer, the mixture was thoroughly vortexed. The homogenized mixture was then incubated in a 60 °C bath for 30 min. Following the incubation period, the mixture was centrifuged to homogenate for 5 min at 12,000 rpm. After centrifuging, the supernatant was transferred to a new tube. An equal volume of chloroform/isoamyl alcohol (24:1) was added then vortexed for 5 s. The sample was centrifuged for 1 min at 12,000 rpm to separate the phases. The upper phase was then pipetted into a new tube; the method was then repeated until the upper phase was clear. The upper clear aqueous phase was then transferred to a new tube. DNA was precipitated by adding 70% by volume ice-cold isopropanol and then incubated at −20 °C for 15 min. The precipitated DNA samples were then centrifuged at 12,000× *g* for 10 min. The supernatant was then extracted without disturbing the pellet and subsequently washed with 500 µL of ice cold 70% by volume ethanol two times, and then finally with absolute alcohol. Then DNA pellets were later dissolved in 20 µL TE buffer (10 mM Tris, pH 8, 1 mM EDTA) [75]. DNA degradation and contamination were checked by using 1% agarose gels, while the purity of the DNA extracted was evaluated by the use of a Nano Photometer^®^ spectrophotometer (IMPLEN, Westlake Village, CA, USA). Ratios of 260 nm and 280 nm were the guidelines to assess the purity of the DNA. The DNA samples with ratios of ~1.8 were selected as pure [76]. For the use of SSR and for the PCR process, specific concentrations of DNA are necessary; we therefore determined the concentration of the DNA samples by using a Qubit^®^ DNA Assay Kit in a Qubit^®^ 2.0 Fluorimeter (Life Technologies, Carlsbad, CA, USA). The Qubit^®^ dsDNA HS (High Sensitivity) Assay Kits make DNA quantitation easy and accurate. The manufacturer’s protocol was followed. Upon mixing the reagents, 1 to 20 μL was added to each DNA sample. The concentrations were read using the Qubit^®^ Fluorometer. Only the DNA samples with concentration ranges of 10 pg/µL to 100 ng/µL were finally used [77].

### 4.3. Screening of SWU Markers and Genotyping of F_2:3_ Populations

The SWU markers used are expressed sequence tag-simple sequence repeat (EST-SSR) primers which were developed from *G. raimondii* by Southwest University, China, thus the acronym SWU. A total of 12,500 pairs of SSR primer were used to screen polymorphic loci between three parents, *G. davidsonii*, *Gossypium klotzschianum*, and their F_1_ generation [78]. In total, 1000 polymorphic loci were used to conduct genotype analysis of 188 F_2:3_ populations. The polymerase chain reaction (PCR) profile was set as follows: denaturation at 94 °C for 2 min, 35 cycles of 30 s at 94 °C for denaturation, 30 s at 52 °C for annealing, 30 s at 72 °C for extension, and 5 min at 72 °C for final extension after the last cycle. The amplified PCR products were separated on 8% denaturing polyacrylamide gel and visualized by silver nitrate staining [79].

### 4.4. Construction of the Linkage Maps

Linkage map analysis was conducted using Join Map 4.0 [69] with a recombination frequency of 0.40 and a LOD score of 2.5 for the F_2:3_ population. The Kosambi mapping function was used to convert the recombination frequencies to map distances. Linkage groups were assigned to chromosomes based on a marker sequence blast search, SWU markers being new sets of SSR markers and not previously annotated to any chromosome. The obtained linkage groups were drawn using Mapchart 2.2 Software [80] and R Software’s mapping function [81].

### 4.5. Collinearity Analysis

Based on the fine genetic linkage map constructed from the F_2:3_ population, the sequences corresponding to SWU markers were used to carry out collinearity analysis between genetic and physical maps of the D genome of *Gossypium raimondii*. A BLASTN Search with E ≤ 1 × 10^−5^, identity ≥ 80%, and matched length ≥ 200 bp was applied (https://blast.ncbi.nlm.nih.gov/Blast.cgi). The best hit for each marker was chosen and were illustrated intuitively using online drawing tools (http://circos.ca/).

### 4.6. Gene Mining, GO Functional Annotation, and Expression Analysis

Regions 20 kb up- and downstream of each SSR location were screened in order to mine the genes from the physical map by the use of full sequences of the SWU markers used in construction of the genetic map. The 20 kb up- and downstream value was selected as the average distance of markers in the genetic map was 2.182 cM, and a similar method has also been adopted in mining several genes; for example, in the identification of the candidate gene for anthracnose disease resistance in *Lupinus angustifolius*, the largest gap ever used was 3.5 cM [82]. The genes mined were analysed for their gene features, protein characteristics, GO, and RNA expression profile using the cotton functional genome database (https://cottonfgd.org/search/), adopting *G. raimondii* as the reference genome. The RNA expression data obtained were then analysed and a heatmap was constructed using the R statistical software package [81].

## 5. Conclusions

In conclusion, wild cotton species harbor many important agronomic traits which can be used to improve the current cultivated cotton cultivar. Plant germplasm resources originate from a number of historical genetic events as a response to environmental stresses and selection; therefore, wild progenitors are important reservoirs of natural genetic variation that can be exploited to increase the genetic base of the elite cultivars. Our map is the first map developed from wild progenitors of the D genome; it covers a total map size of 1480.23 cM, with 728 mono-markers. We further identified a total of 2063 genes from the SSR regions of the physical map. Of significance were the stress-related genes, which exhibited differential expression in various tissues. The genes such as *NAC*, *Myb*, and ubiquitin-like genes, have been highly associated with abiotic and biotic stress factors in plants, and thus the detection of these genes provides valuable information for future exploration. Twenty-seven (27) key genes were identified with diverse roles such as plant hormone signaling, development, and defense reactions. The use of the two wild species, *G. davidsonii* and *G. klotzschianum*, in the construction of the first fine genetic map of two wild species in the D genome will provide insight for further genetic analysis. This is the first genetic map developed from the population of F_2:3_. The map covers a greater percentage of the whole D genome and therefore it forms a valuable tool for breeders in detecting QTLs related to salt stress and biotic stress tolerance, especially aphids, sucking pests, and the secondary metabolic product gossypol.

## Figures and Tables

**Table 1:** Polymorphic rate of screened SWU primers.

Primer	No. of Markers	No. of Polymorphic Primers	Dominant Markers	Non-Dominant Markers	No. of Linked Markers	Polymorphic Rate
SWU	12,650	1000	200 (27.17%)	528 (72.83%)	728	8.00%

**Table 2:** Characteristics of the genetic map.

Chr.	Markers per Chromosome	SD	Average Distance (cM)	Average % SD	Map Size (cM)	Marker Distance Gaps (cM) per Chromosome						Ratio
						Smallest Gap (cM)	Largest Gap in (cM)	<5 cM	5–10 cM	10–20 cM	>20 cM	
**Chr01**	89	3	1.304	3.371	116.045	0.005	8.756	83	5	0	0	0.9326
**Chr02**	44	35	1.884	76.087	82.908	0.044	18.328	39	3	1	0	0.8864
**Chr03**	45	8	2.59	17.778	116.528	0.14	10.009	39	1	1	0	0.8667
**Chr04**	56	2	1.997	3.571	111.846	0.008	17.538	53	1	1	0	0.9464
**Chr05**	49	17	2.361	34.694	115.671	0.056	8.493	45	5	0	0	0.9184
**Chr06**	58	5	2.001	8.621	116.045	0.002	14.926	52	3	2	0	0.8966
**Chr07**	86	35	1.446	40.698	124.358	0.016	7.988	81	4	0	0	0.9419
**Chr08**	49	25	2.492	51.02	122.13	0.054	14.099	45	0	3	0	0.9184
**Chr09**	69	12	1.697	16.216	117.06	0.046	11.167	63	3	2	0	0.9130
**chr10**	34	2	2.998	5.882	101.93	0.117	12.472	25	7	1	0	0.7353
**Chr11**	63	5	1.806	7.937	113.801	0.042	36.597	60	1	0	1	0.9524
**Chr12**	49	6	2.301	12.245	112.739	0.065	9.588	43	5	0	0	0.8776
**Chr13**	37	4	3.491	10.526	129.164	0.137	25.3	28	7	0	1	0.7568
**Genetic map**	728	159	2.182	22.20	1480.23	0.0563	15.020	656	45	11	2	0.9011

Chr chromosome; SD segregation distortion; cM centiMorgan; % percentage; <less than; The ratio is the number of markers less than 5 divided by the number markers in the same linkage group.

**Table 3:** Segregation distortion in the F_2:3_ interspecific populations derived from two wild cottons of D genome.

Chromosome	Chi Square (χ^2^) Values							Loci Number			
	*p* < 0.05	*p* < 0.01	*p* < 0.005	*p* < 0.001	*p* < 0.0005	*p* < 0.0001	*p* < 0.00005	*G. davidsonii*	*G. klotzschianum*	Heterozygote’s	Totals
Chr01	0	1	0	0	0	0	2	2	0	1	3
Chr02	1	0	0	0	0	0	34	0	34	1	35
Chr03	2	2	1	0	0	0	3	1	0	7	8
Chr04	0	1	0	0	0	0	1	1	0	1	2
Chr05	3	1	0	0	0	0	13	0	11	6	17
Chr06	0	1	0	0	0	0	4	4	0	1	5
Chr07	11	10	5	1	1	0	7	1	5	29	35
Chr08	2	0	1	0	0	0	22	0	22	3	25
Chr09	5	5	1	0	1	0	0	0	0	12	12
Chr10	0	0	0	0	1	0	1	1	0	1	2
Chr11	3	1	0	1	0	0	0	0	0	5	5
Chr12	3	0	0	0	0	0	3	2	1	3	6
Chr13	2	0	0	0	0	0	2	0	1	3	4
Total	32	22	8	2	3	0	92	12	74	73	159

*p* < 0.05; *p* < 0.01; *p* < 0.005; *p* < 0.001; *p* < 0.0005; *p* < 0.0001; *p* < 0.00005 significant levels at 0.05, 0.01, 0.005, 0.001, 0.0005, 0.00001, and 0.00005 respectively.

## Supplementary Materials

Supplementary materials can be found at www.mdpi.com/1422-0067/19/1/204/s1.

## Abbreviations

LG	linkage group
EST-SSR	Express sequence tag-simple sequence repeat
gSSR	Genomic simple sequence repeat
GO	Gene ontology
BP	Biological process
MF	Molecular function
CC	Cellular components
GRAVY	Grand average of hydropathy
SWU	South west university
MPK	Mitogen-activated protein kinase
