# Supplementary material for: Simple Sequence Repeat (SSR) Genetic Linkage Map of D Genome Diploid Cotton Derived from an Interspecific Cross between Gossypium davidsonii and Gossypium klotzschianum
Source: Int J Mol Sci. 2018 Jan 11;19(1):204. doi: 10.3390/ijms19010204 (PMC5796153; doi:10.3390/ijms19010204)
Supplement: Supplementary file 1 [file ijms-19-00204-s001.zip › ijms-254865 final supplementary/Supplementary Figure 2.pdf]

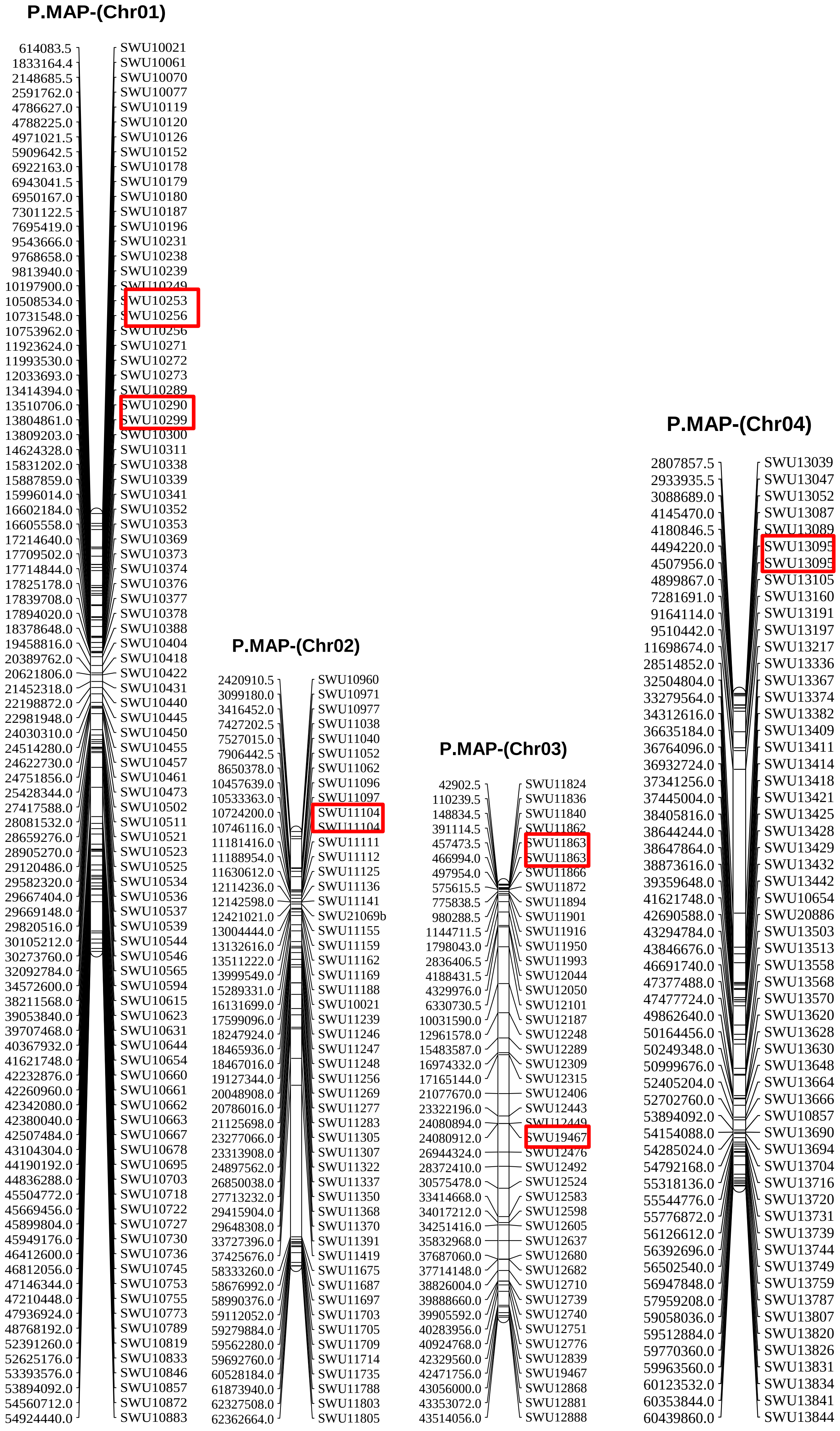
P.MAP-(Chr01)
614083.5 SWU10021
1833164.4 SWU10061
2148685.5 SWU10070
2591762.0 SWU10077
4786627.0 SWU10119
4788225.0 SWU10120
4971021.5 SWU10126
5909642.5 SWU10152
6922163.0 SWU10178
6943041.5 SWU10179
6950167.0 SWU10180
7301122.5 SWU10187
7695419.0 SWU10196
9543666.0 SWU10231
9768658.0 SWU10238
9813940.0 SWU10239
10197900.0 SWU10249
10508534.0 SWU10253
10731548.0 SWU10256
10753962.0 SWU10256
11923624.0 SWU10271
11993530.0 SWU10272
12033693.0 SWU10273
13414394.0 SWU10289
13510706.0 SWU10290
13804861.0 SWU10299
13809203.0 SWU10300
14624328.0 SWU10311
15831202.0 SWU10338
15887859.0 SWU10339
15996014.0 SWU10341
16602184.0 SWU10352
16605558.0 SWU10353
17214640.0 SWU10369
17709502.0 SWU10373
17714844.0 SWU10374
17825178.0 SWU10376
17839708.0 SWU10377
17894020.0 SWU10378
18378648.0 SWU10388
19458816.0 SWU10404
20389762.0 SWU10418
20621806.0 SWU10422
21452318.0 SWU10431
22198872.0 SWU10440
22981948.0 SWU10445
24030310.0 SWU10450
24514280.0 SWU10455
24622730.0 SWU10457
24751856.0 SWU10461
25428344.0 SWU10473
27417588.0 SWU10502
28081532.0 SWU10511
28659276.0 SWU10521
28905270.0 SWU10523
29120486.0 SWU10525
29582320.0 SWU10534
29667404.0 SWU10536
29669148.0 SWU10537
29820516.0 SWU10539
30105212.0 SWU10544
30273760.0 SWU10546
32092784.0 SWU10565
34572600.0 SWU10594
38211568.0 SWU10615
39053840.0 SWU10623
39707468.0 SWU10631
40367932.0 SWU10644
41621748.0 SWU10654
42232876.0 SWU10660
42260960.0 SWU10661
42342080.0 SWU10662
42380040.0 SWU10663
42507484.0 SWU10667
43104304.0 SWU10678
44190192.0 SWU10695
44836288.0 SWU10703
45504772.0 SWU10718
45669456.0 SWU10722
45899804.0 SWU10727
45949176.0 SWU10730
46412600.0 SWU10736
46812056.0 SWU10745
47146344.0 SWU10753
47210448.0 SWU10755
47936924.0 SWU10773
48768192.0 SWU10789
52391260.0 SWU10819
52625176.0 SWU10833
53393576.0 SWU10846
53894092.0 SWU10857
54560712.0 SWU10872
54924440.0 SWU10883
P.MAP-(Chr02)
2420910.5 SWU10960
3099180.0 SWU10971
3416452.0 SWU10977
7427202.5 SWU11038
7527015.0 SWU11040
7906442.5 SWU11052
8650378.0 SWU11062
10457639.0 SWU11096
10533363.0 SWU11097
10724200.0 SWU11104
10746116.0 SWU11104
11181416.0 SWU11111
11188954.0 SWU11112
11630612.0 SWU11125
12114236.0 SWU11136
12142598.0 SWU11141
12421021.0 SWU21069b
13004444.0 SWU11155
13132616.0 SWU11159
13511222.0 SWU11162
13999549.0 SWU11169
15289331.0 SWU11188
16131699.0 SWU10021
17599096.0 SWU11239
18247924.0 SWU11246
18465936.0 SWU11247
18467016.0 SWU11248
19127344.0 SWU11256
20048908.0 SWU11269
20786016.0 SWU11277
21125698.0 SWU11283
23277066.0 SWU11305
23313908.0 SWU11307
24897562.0 SWU11322
26850038.0 SWU11337
27713232.0 SWU11350
29415904.0 SWU11368
29648308.0 SWU11370
33727396.0 SWU11391
37425676.0 SWU11419
58333260.0 SWU11675
58676992.0 SWU11687
58990376.0 SWU11697
59112052.0 SWU11703
59279884.0 SWU11705
59562280.0 SWU11709
59692760.0 SWU11714
60528184.0 SWU11735
61873940.0 SWU11788
62327508.0 SWU11803
62362664.0 SWU11805
P.MAP-(Chr03)
42902.5 SWU11824
110239.5 SWU11836
148834.5 SWU11840
391114.5 SWU11862
457473.5 SWU11863
466994.0 SWU11863
497954.0 SWU11866
575615.5 SWU11872
775838.5 SWU11894
980288.5 SWU11901
1144711.5 SWU11916
1798043.0 SWU11950
2836406.5 SWU11993
4188431.5 SWU12044
4329976.0 SWU12050
6330730.5 SWU12101
10031590.0 SWU12187
12961578.0 SWU12248
15483587.0 SWU12289
16974332.0 SWU12309
17165144.0 SWU12315
21077670.0 SWU12406
23322196.0 SWU12443
24080894.0 SWU12449
24080912.0 SWU19467
26944324.0 SWU12476
28372410.0 SWU12492
30575478.0 SWU12524
33414668.0 SWU12583
34017212.0 SWU12598
34251416.0 SWU12605
35832968.0 SWU12637
37687060.0 SWU12680
37714148.0 SWU12682
38826004.0 SWU12710
39888660.0 SWU12739
39905592.0 SWU12740
40283956.0 SWU12751
40924768.0 SWU12776
42329560.0 SWU12839
42471756.0 SWU19467
43056000.0 SWU12868
43353072.0 SWU12881
43514056.0 SWU12888
P.MAP-(Chr04)
2807857.5 SWU13039
2933935.5 SWU13047
3088689.0 SWU13052
4145470.0 SWU13087
4180846.5 SWU13089
4494220.0 SWU13095
4507956.0 SWU13095
4899867.0 SWU13105
7281691.0 SWU13160
9164114.0 SWU13191
9510442.0 SWU13197
11698674.0 SWU13217
28514852.0 SWU13336
32504804.0 SWU13367
33279564.0 SWU13374
34312616.0 SWU13382
36635184.0 SWU13409
36764096.0 SWU13411
36932724.0 SWU13414
37341256.0 SWU13418
37445004.0 SWU13421
38405816.0 SWU13425
38644244.0 SWU13428
38647864.0 SWU13429
38873616.0 SWU13432
39359648.0 SWU13442
41621748.0 SWU10654
42690588.0 SWU20886
43294784.0 SWU13503
43846676.0 SWU13513
46691740.0 SWU13558
47377488.0 SWU13568
47477724.0 SWU13570
49862640.0 SWU13620
50164456.0 SWU13628
50249348.0 SWU13630
50999676.0 SWU13648
52405204.0 SWU13664
52702760.0 SWU13666
53894092.0 SWU10857
54154088.0 SWU13690
54285024.0 SWU13694
54792168.0 SWU13704
55318136.0 SWU13716
55544776.0 SWU13720
55776872.0 SWU13731
56126612.0 SWU13739
56392696.0 SWU13744
56502540.0 SWU13749
56947848.0 SWU13759
57959208.0 SWU13787
59058036.0 SWU13807
59512884.0 SWU13820
59770360.0 SWU13826
59963560.0 SWU13831
60123532.0 SWU13834
60353844.0 SWU13841
60439860.0 SWU13844

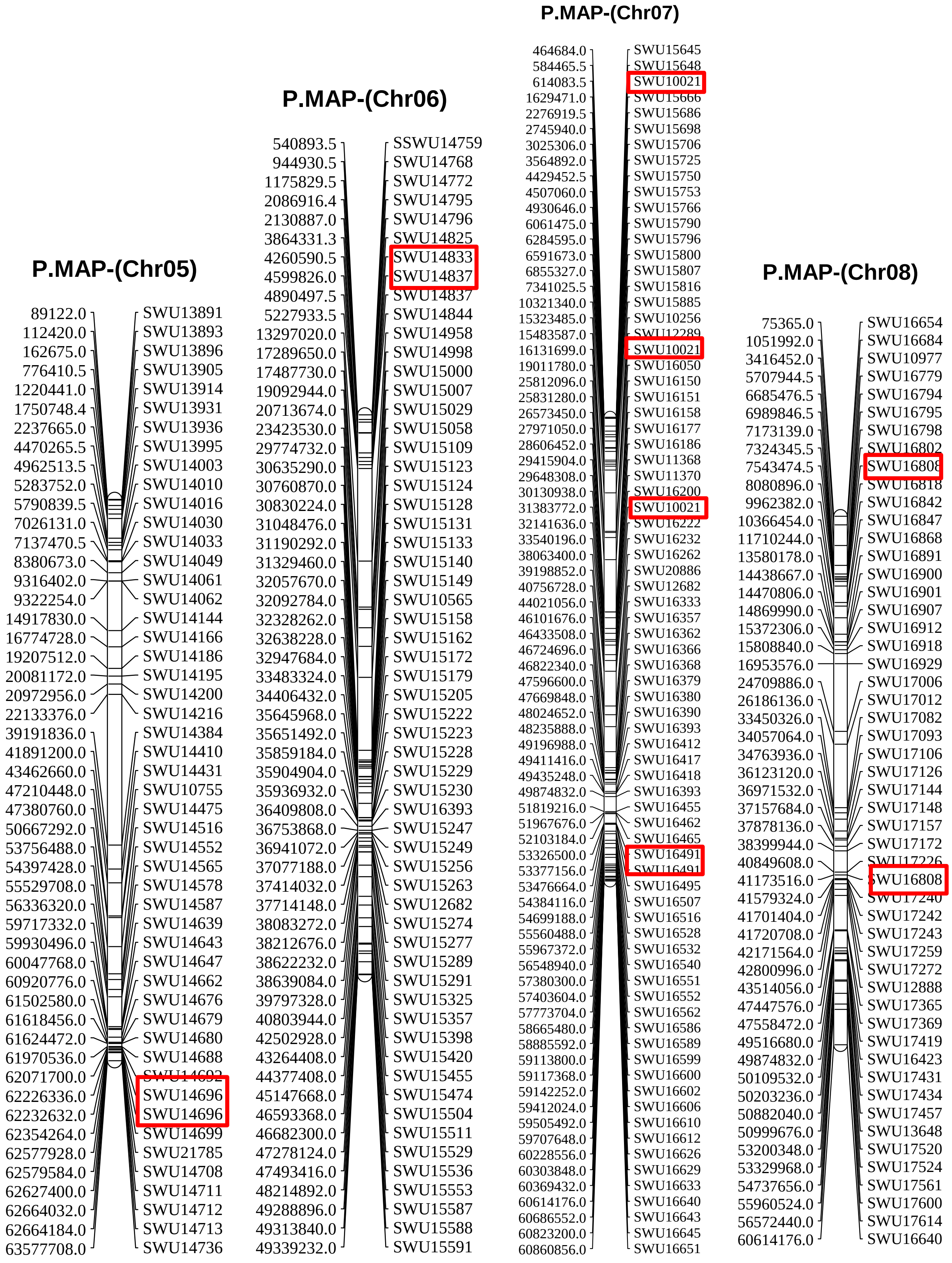
P.MAP-(Chr05)
89122.0 SWU13891
112420.0 SWU13893
162675.0 SWU13896
776410.5 SWU13905
1220441.0 SWU13914
1750748.4 SWU13931
2237665.0 SWU13936
4470265.5 SWU13995
4962513.5 SWU14003
5283752.0 SWU14010
5790839.5 SWU14016
7026131.0 SWU14030
7137470.5 SWU14033
8380673.0 SWU14049
9316402.0 SWU14061
9322254.0 SWU14062
14917830.0 SWU14144
16774728.0 SWU14166
19207512.0 SWU14186
20081172.0 SWU14195
20972956.0 SWU14200
22133376.0 SWU14216
39191836.0 SWU14384
41891200.0 SWU14410
43462660.0 SWU14431
47210448.0 SWU10755
47380760.0 SWU14475
50667292.0 SWU14516
53756488.0 SWU14552
54397428.0 SWU14565
55529708.0 SWU14578
56336320.0 SWU14587
59717332.0 SWU14639
59930496.0 SWU14643
60047768.0 SWU14647
60920776.0 SWU14662
61502580.0 SWU14676
61618456.0 SWU14679
61624472.0 SWU14680
61970536.0 SWU14688
62071700.0 SWU14692
62226336.0 SWU14696
62232632.0 SWU14696
62354264.0 SWU14699
62577928.0 SWU21785
62579584.0 SWU14708
62627400.0 SWU14711
62664032.0 SWU14712
62664184.0 SWU14713
63577708.0 SWU14736
P.MAP-(Chr06)
540893.5 SSWU14759
944930.5 SWU14768
1175829.5 SWU14772
2086916.4 SWU14795
2130887.0 SWU14796
3864331.3 SWU14825
4260590.5 SWU14833
4599826.0 SWU14837
4890497.5 SWU14837
5227933.5 SWU14844
13297020.0 SWU14958
17289650.0 SWU14998
17487730.0 SWU15000
19092944.0 SWU15007
20713674.0 SWU15029
23423530.0 SWU15058
29774732.0 SWU15109
30635290.0 SWU15123
30760870.0 SWU15124
30830224.0 SWU15128
31048476.0 SWU15131
31190292.0 SWU15133
31329460.0 SWU15140
32057670.0 SWU15149
32092784.0 SWU10565
32328262.0 SWU15158
32638228.0 SWU15162
32947684.0 SWU15172
33483324.0 SWU15179
34406432.0 SWU15205
35645968.0 SWU15222
35651492.0 SWU15223
35859184.0 SWU15228
35904904.0 SWU15229
35936932.0 SWU15230
36409808.0 SWU16393
36753868.0 SWU15247
36941072.0 SWU15249
37077188.0 SWU15256
37414032.0 SWU15263
37714148.0 SWU12682
38083272.0 SWU15274
38212676.0 SWU15277
38622232.0 SWU15289
38639084.0 SWU15291
39797328.0 SWU15325
40803944.0 SWU15357
42502928.0 SWU15398
43264408.0 SWU15420
44377408.0 SWU15455
45147668.0 SWU15474
46593368.0 SWU15504
46682300.0 SWU15511
47278124.0 SWU15529
47493416.0 SWU15536
48214892.0 SWU15553
49288896.0 SWU15587
49313840.0 SWU15588
49339232.0 SWU15591
P.MAP-(Chr07)
464684.0 SWU15645
584465.5 SWU15648
614083.5 SWU10021
1629471.0 SWU15666
2276919.5 SWU15686
2745940.0 SWU15698
3025306.0 SWU15706
3564892.0 SWU15725
4429452.5 SWU15750
4507060.0 SWU15753
4930646.0 SWU15766
6061475.0 SWU15790
6284595.0 SWU15796
6591673.0 SWU15800
6855327.0 SWU15807
7341025.5 SWU15816
10321340.0 SWU15885
15323485.0 SWU10256
15483587.0 SWU12289
16131699.0 SWU10021
19011780.0 SWU16050
25812096.0 SWU16150
25831280.0 SWU16151
26573450.0 SWU16158
27971050.0 SWU16177
28606452.0 SWU16186
29415904.0 SWU11368
29648308.0 SWU11370
30130938.0 SWU16200
31383772.0 SWU10021
32141636.0 SWU16222
33540196.0 SWU16232
38063400.0 SWU16262
39198852.0 SWU20886
40756728.0 SWU12682
44021056.0 SWU16333
46101676.0 SWU16357
46433508.0 SWU16362
46724696.0 SWU16366
46822340.0 SWU16368
47596600.0 SWU16379
47669848.0 SWU16380
48024652.0 SWU16390
48235888.0 SWU16393
49196988.0 SWU16412
49411416.0 SWU16417
49435248.0 SWU16418
49874832.0 SWU16393
51819216.0 SWU16455
51967676.0 SWU16462
52103184.0 SWU16465
53326500.0 SWU16491
53377156.0 SWU16491
53476664.0 SWU16495
54384116.0 SWU16507
54699188.0 SWU16516
55560488.0 SWU16528
55967372.0 SWU16532
56548940.0 SWU16540
57380300.0 SWU16551
57403604.0 SWU16552
57773704.0 SWU16562
58665480.0 SWU16586
58885592.0 SWU16589
59130800.0 SWU16599
59117368.0 SWU16600
59142252.0 SWU16602
59412024.0 SWU16606
59505492.0 SWU16610
59707648.0 SWU16612
60228556.0 SWU16626
60303848.0 SWU16629
60369432.0 SWU16633
60614176.0 SWU16640
60686552.0 SWU16643
60823200.0 SWU16645
60860856.0 SWU16651
P.MAP-(Chr08)
75365.0 SWU16654
1051992.0 SWU16684
3416452.0 SWU10977
5707944.5 SWU16779
6685476.5 SWU16794
6989846.5 SWU16795
7173139.0 SWU16798
7324345.5 SWU16802
7543474.5 SWU1680
8080896.0 SWU16818
9962382.0 SWU16842
10366454.0 SWU16847
11710244.0 SWU16868
13580178.0 SWU16891
14438667.0 SWU16900
14470806.0 SWU16901
14869990.0 SWU16907
15372306.0 SWU16912
15808840.0 SWU16918
16953576.0 SWU16929
24709886.0 SWU17006
26186136.0 SWU17012
33450326.0 SWU17082
34057064.0 SWU17093
34763936.0 SWU17106
36123120.0 SWU17126
36971532.0 SWU17144
37157684.0 SWU17148
37878136.0 SWU17157
38399944.0 SWU17172
40849608.0 SWU17226
41173516.0 SWU16808
41579324.0 SWU17240
41701404.0 SWU17242
41720708.0 SWU17243
42171564.0 SWU17259
42800996.0 SWU17272
43514056.0 SWU12888
47447576.0 SWU17365
47558472.0 SWU17369
49516680.0 SWU17419
49874832.0 SWU16423
50109532.0 SWU17431
50203236.0 SWU17434
50882040.0 SWU17457
50999676.0 SWU13648
53200348.0 SWU17520
53329968.0 SWU17524
54737656.0 SWU17561
55960524.0 SWU17600
56572440.0 SWU17614
60614176.0 SWU16640

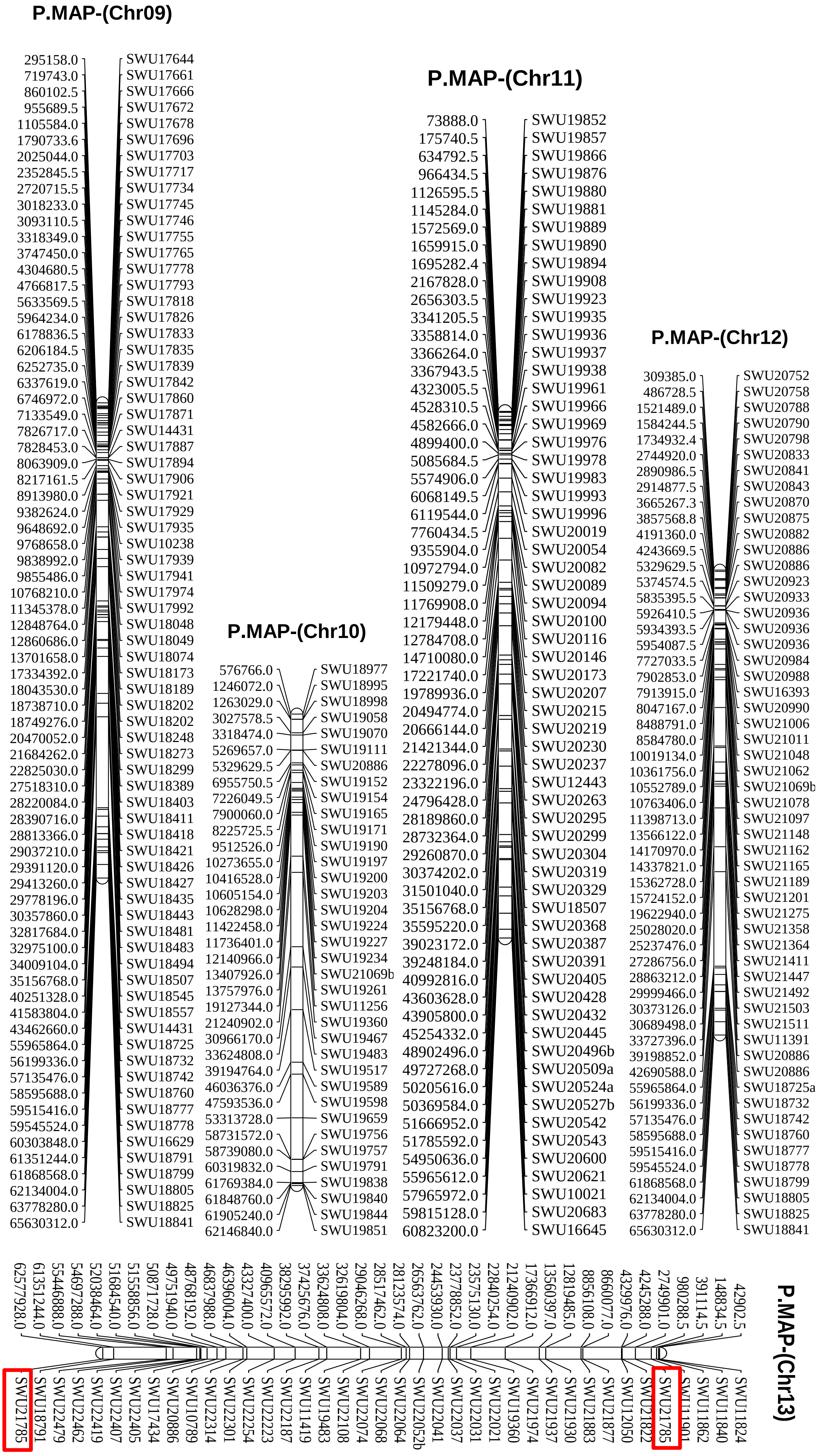

P.MAP-(Chr09)
295158.0 SWU17644
719743.0 SWU17661
860102.5 SWU17666
955689.5 SWU17672
1105584.0 SWU17678
1790733.6 SWU17696
2025044.0 SWU17703
2352845.5 SWU17717
2720715.5 SWU17734
3018233.0 SWU17745
3093110.5 SWU17746
3318349.0 SWU17755
3747450.0 SWU17765
4304680.5 SWU17778
4766817.5 SWU17793
5633569.5 SWU17818
5964234.0 SWU17826
6178836.5 SWU17833
6206184.5 SWU17835
6252735.0 SWU17839
6337619.0 SWU17842
6746972.0 SWU17860
7133549.0 SWU17871
7826717.0 SWU14431
7828453.0 SWU17887
8063909.0 SWU17894
8217161.5 SWU17906
8913980.0 SWU17921
9382624.0 SWU17929
9648692.0 SWU17935
9768658.0 SWU10238
9838992.0 SWU17939
9855486.0 SWU17941
10768210.0 SWU17974
11345378.0 SWU17992
12848764.0 SWU18048
12860686.0 SWU18049
13701658.0 SWU18074
17334392.0 SWU18173
18043530.0 SWU18189
18738710.0 SWU18202
18749276.0 SWU18202
20470052.0 SWU18248
21684262.0 SWU18273
22825030.0 SWU18299
27518310.0 SWU18389
28220084.0 SWU18403
28390716.0 SWU18411
28813366.0 SWU18418
29037210.0 SWU18421
29391120.0 SWU18426
29413260.0 SWU18427
29778196.0 SWU18435
30357860.0 SWU18443
32817684.0 SWU18481
32975100.0 SWU18483
34009104.0 SWU18494
35156768.0 SWU18507
40251328.0 SWU18545
41583804.0 SWU18557
43462660.0 SWU14431
55965864.0 SWU18725
56199336.0 SWU18732
57135476.0 SWU18742
58595688.0 SWU18760
59515416.0 SWU18777
59545524.0 SWU18778
60303848.0 SWU16629
61351244.0 SWU18791
61868568.0 SWU18799
62134004.0 SWU18805
63778280.0 SWU18825
65630312.0 SWU18841
P.MAP-(Chr10)
576766.0 SWU18977
1246072.0 SWU18995
1263029.0 SWU18998
3027578.5 SWU19058
3318474.0 SWU19070
5269657.0 SWU19111
5329629.5 SWU20886
6955750.5 SWU19152
7226049.5 SWU19154
7900060.0 SWU19165
8225725.5 SWU19171
9512526.0 SWU19190
10273655.0 SWU19197
10416528.0 SWU19200
10605154.0 SWU19203
10628298.0 SWU19204
11422458.0 SWU19224
11736401.0 SWU19227
12140966.0 SWU19234
13407926.0 SWU21069b
13757976.0 SWU19261
19127344.0 SWU11256
21240902.0 SWU19360
30966170.0 SWU19467
33624808.0 SWU19483
39194764.0 SWU19517
46036376.0 SWU19589
47593536.0 SWU19598
53313728.0 SWU19659
58731572.0 SWU19756
58739080.0 SWU19757
60319832.0 SWU19791
61769384.0 SWU19838
61848760.0 SWU19840
61905240.0 SWU19844
62146840.0 SWU19851
P.MAP-(Chr11)
73888.0 SWU19852
175740.5 SWU19857
634792.5 SWU19866
966434.5 SWU19876
1126595.5 SWU19880
1145284.0 SWU19881
1572569.0 SWU19889
1659915.0 SWU19890
1695282.4 SWU19894
2167828.0 SWU19908
2656303.5 SWU19923
3341205.5 SWU19935
3358814.0 SWU19936
3366264.0 SWU19937
3367943.5 SWU19938
4323005.5 SWU19961
4528310.5 SWU19966
4582666.0 SWU19969
4899400.0 SWU19976
5085684.5 SWU19978
5574906.0 SWU19983
6068149.5 SWU19993
6119544.0 SWU19996
7760434.5 SWU20019
9355904.0 SWU20054
10972794.0 SWU20082
11509279.0 SWU20089
11769908.0 SWU20094
12179448.0 SWU20100
12784708.0 SWU20116
14710080.0 SWU20146
17221740.0 SWU20173
19789936.0 SWU20207
20494774.0 SWU20215
20666144.0 SWU20219
21421344.0 SWU20230
22278096.0 SWU20237
23322196.0 SWU12443
24796428.0 SWU20263
28189860.0 SWU20295
28732364.0 SWU20299
29260870.0 SWU20304
30374202.0 SWU20319
31501040.0 SWU20329
35156768.0 SWU18507
35595220.0 SWU20368
39023172.0 SWU20387
39248184.0 SWU20391
40992816.0 SWU20405
43603628.0 SWU20428
43905800.0 SWU20432
45254332.0 SWU20445
48902496.0 SWU20496b
49727268.0 SWU20509a
50205616.0 SWU20524a
50369584.0 SWU20527b
51666952.0 SWU20542
51785592.0 SWU20543
54950636.0 SWU20600
55965612.0 SWU20621
57965972.0 SWU10021
59815128.0 SWU20683
60823200.0 SWU16645
P.MAP-(Chr12)
309385.0 SWU20752
486728.5 SWU20758
1521489.0 SWU20788
1584244.5 SWU20790
1734932.4 SWU20798
2744920.0 SWU20833
2890986.5 SWU20841
2914877.5 SWU20843
3665267.3 SWU20870
3857568.8 SWU20875
4191360.0 SWU20882
4243669.5 SWU20886
5329629.5 SWU20886
5374574.5 SWU20923
5835395.5 SWU20933
5926410.5 SWU20936
5934393.5 SWU20936
5954087.5 SWU20936
7727033.5 SWU20984
7902853.0 SWU20988
7913915.0 SWU16393
8047167.0 SWU20990
8488791.0 SWU21006
8584780.0 SWU21011
10019134.0 SWU21048
10361756.0 SWU21062
10552789.0 SWU21069b
10763406.0 SWU21078
11398713.0 SWU21097
13566122.0 SWU21148
14170970.0 SWU21162
14337821.0 SWU21165
15362728.0 SWU21189
15724152.0 SWU21201
19622940.0 SWU21275
25028020.0 SWU21358
25237476.0 SWU21364
27286756.0 SWU21411
28863212.0 SWU21447
29999466.0 SWU21492
30373126.0 SWU21503
30689498.0 SWU21511
33727396.0 SWU11391
39198852.0 SWU20886
42690588.0 SWU20886
55965864.0 SWU18725a
56199336.0 SWU18732
57135476.0 SWU18742
58595688.0 SWU18760
59515416.0 SWU18777
59545524.0 SWU18778
61868568.0 SWU18799
62134004.0 SWU18805
63778280.0 SWU18825
65630312.0 SWU18841
P.MAP-(Chr13)
62577928.0 61351244.0 55446888.0 54697288.0 52038464.0 51684540.0 51558856.0 50871728.0 49751940.0 48768192.0 46837988.0 46396004.0 43327400.0 40965572.0 38295992.0 37425676.0 33624808.0 32619804.0 29046268.0 28517462.0 28123574.0 26563762.0 24453930.0 23778852.0 23575130.0 22840254.0 21240902.0 17366912.0 13560397.0 12819485.0 8856108.0 8660077.0 4329976.0 4245288.0 2749901.0 980288.5 391114.5 148834.5 42902.5
SWU21785 SWU18791 SWU22479 SWU22462 SWU22419 SWU22407 SWU22405 SWU17434 SWU20886 SWU10789 SWU22314 SWU22301 SWU22254 SWU22223 SWU22187 SWU11419 SWU19483 SWU22108 SWU22074 SWU22068 SWU22064 SWU22052b SWU22041 SWU22037 SWU22031 SWU22021 SWU19360 SWU21974 SWU21937 SWU21930 SWU21883 SWU21877 SWU12050 SWU21822 SWU21785 SWU11901 SWU11862 SWU11840 SWU11824
