# Supplementary material for: Simple Sequence Repeat (SSR) Genetic Linkage Map of D Genome Diploid Cotton Derived from an Interspecific Cross between Gossypium davidsonii and Gossypium klotzschianum
Source: Int J Mol Sci. 2018 Jan 11;19(1):204. doi: 10.3390/ijms19010204 (PMC5796153; doi:10.3390/ijms19010204)
Supplement: Supplementary file 1 [file ijms-19-00204-s001.zip › ijms-254865 final supplementary/Supplementary table S2.docx]

Supplementary Table S2: The genes mined from the 20 kb up and down stream of the SSR marker regions, with their chromosome location, gene description , lengths and the strand type.

| Gene ID | Gene Name | Description | Chromosome | Start | End | Strand | Length (bp) |
| --- | --- | --- | --- | --- | --- | --- | --- |
| Gorai.001G212500 | DAO | 2-oxoglutarate-dependent dioxygenase DAO | Chr01 | 42,380,139 | 42,381,864 | + | 1,726 |
| Gorai.001G087800 | pgdC | 6-phosphogluconate dehydrogenase, decarboxylating 1 | Chr01 | 9,536,276 | 9,536,844 | + | 569 |
| Gorai.001G179300 | At4g26100 | Casein kinase I isoform delta-like | Chr01 | 27,397,367 | 27,403,514 | + | 6,148 |
| Gorai.001G254700 | At4g26100 | Casein kinase I isoform delta-like | Chr01 | 52,629,131 | 52,634,091 | + | 4,961 |
| Gorai.001G106000 | NA | Chitinase 2 | Chr01 | 11,937,695 | 11,938,621 | - | 927 |
| Gorai.001G156500 | mvd | Diphosphomevalonate decarboxylase | Chr01 | 22,187,716 | 22,189,397 | - | 1,682 |
| Gorai.001G136600 | rpoB | DNA-directed RNA polymerase subunit beta | Chr01 | 17,899,781 | 17,900,237 | - | 457 |
| Gorai.001G172100 | RPC19 | DNA-directed RNA polymerases I and III subunit RPAC2 | Chr01 | 24,738,767 | 24,739,750 | + | 984 |
| Gorai.001G126200 | DNAJ1 | DnaJ protein homolog | Chr01 | 15,892,758 | 15,895,745 | + | 2,988 |
| Gorai.001G268500 | GAPC2 | Glyceraldehyde-3-phosphate dehydrogenase 2, cytosolic | Chr01 | 54,541,511 | 54,544,613 | + | 3,103 |
| Gorai.001G252300 | H6H | Hyoscyamine 6-dioxygenase | Chr01 | 52,377,802 | 52,379,871 | + | 2,070 |
| Gorai.001G206200 | SLC4A1AP | Kanadaptin | Chr01 | 40,353,885 | 40,360,552 | + | 6,668 |
| Gorai.001G075200 | NACK1 | Kinesin-like protein NACK1 | Chr01 | 7,695,877 | 7,701,631 | + | 5,755 |
| Gorai.001G019800 | ERECTA | LRR receptor-like serine/threonine-protein kinase ERECTA | Chr01 | 1,836,341 | 1,843,979 | - | 7,639 |
| Gorai.001G050500 | LECRK41 | L-type lectin-domain containing receptor kinase IV.1 | Chr01 | 4,791,007 | 4,793,034 | - | 2,028 |
| Gorai.001G052400 | MED31 | Mediator of RNA polymerase II transcription subunit 31 | Chr01 | 4,967,649 | 4,971,655 | - | 4,007 |
| Gorai.001G106100 | ndhB2 | NAD(P)H-quinone oxidoreductase subunit 2 B, chloroplastic | Chr01 | 11,942,176 | 11,942,438 | - | 263 |
| Gorai.001G204300 | NA | NADH dehydrogenase [ubiquinone] iron-sulfur protein 1, mitochondrial | Chr01 | 39,711,998 | 39,717,699 | + | 5,702 |
| Gorai.001G072000 | NRP2 | NAP1-related protein 2 | Chr01 | 7,297,048 | 7,301,599 | + | 4,552 |
| Gorai.001G263400 | At1g48100 | Polygalacturonase At1g48100 | Chr01 | 53,890,866 | 53,893,949 | - | 3,084 |
| Gorai.001G186800 | At5g48740 | Probable LRR receptor-like serine/threonine-protein kinase At5g48740 | Chr01 | 29,829,300 | 29,834,348 | + | 5,049 |
| Gorai.001G186500 | At3g03100 | Probable NADH dehydrogenase [ubiquinone] 1 alpha subcomplex subunit 12 | Chr01 | 29,805,581 | 29,808,715 | + | 3,135 |
| Gorai.001G126300 | At1g49730 | Probable receptor-like protein kinase At1g49730 | Chr01 | 15,894,642 | 15,898,033 | - | 3,392 |
| Gorai.001G114400 | At5g41260 | Probable serine/threonine-protein kinase At5g41260 | Chr01 | 13,404,777 | 13,408,188 | + | 3,412 |
| Gorai.001G212400 | utp7 | Probable U3 small nucleolar RNA-associated protein 7 | Chr01 | 42,374,119 | 42,378,622 | + | 4,504 |
| Gorai.001G106400 | At3g62120 | Proline--tRNA ligase, cytoplasmic | Chr01 | 11,981,213 | 11,984,949 | - | 3,737 |
| Gorai.001G071900 | PKS1 | Protein PHYTOCHROME KINASE SUBSTRATE 1 | Chr01 | 7,285,266 | 7,286,902 | + | 1,637 |
| Gorai.001G069000 | DHBK | Putative 3,4-dihydroxy-2-butanone kinase | Chr01 | 6,948,242 | 6,957,268 | + | 9,027 |
| Gorai.001G211900 | FER | Receptor-like protein kinase FERONIA | Chr01 | 42,257,155 | 42,259,708 | + | 2,554 |
| Gorai.001G182900 | ALE2 | Receptor-like serine/threonine-protein kinase ALE2 | Chr01 | 28,076,415 | 28,084,869 | + | 8,455 |
| Gorai.001G094900 | BCK1 | Serine/threonine-protein kinase BCK1/SLK1/SSP31 | Chr01 | 10,523,209 | 10,524,127 | - | 919 |
| Gorai.001G022800 | EDR1 | Serine/threonine-protein kinase EDR1 | Chr01 | 2,150,897 | 2,158,250 | + | 7,354 |
| Gorai.001G185100 | EDR1 | Serine/threonine-protein kinase EDR1 | Chr01 | 29,557,800 | 29,565,550 | + | 7,751 |
| Gorai.001G234100 | LUH | Transcriptional corepressor LEUNIG_HOMOLOG | Chr01 | 47,146,002 | 47,153,506 | - | 7,505 |
| Gorai.001G052100 | usb1 | U6 snRNA phosphodiesterase | Chr01 | 4,951,115 | 4,954,629 | + | 3,515 |
| Gorai.001G174300 | GAE3 | UDP-glucuronate 4-epimerase 3 | Chr01 | 25,442,236 | 25,444,250 | - | 2,015 |
| Gorai.002G032100 | PDK | [Pyruvate dehydrogenase (acetyl-transferring)] kinase, mitochondrial | Chr02 | 2,397,956 | 2,402,060 | + | 4,105 |
| Gorai.002G263200 | Dnajb13 | DnaJ homolog subfamily B member 13 | Chr02 | 62,379,247 | 62,382,541 | - | 3,295 |
| Gorai.002G082800 | GA2OX8 | Gibberellin 2-beta-dioxygenase 8 | Chr02 | 10,530,616 | 10,532,770 | - | 2,155 |
| Gorai.002G137800 | HEMA1 | Glutamyl-tRNA reductase 1, chloroplastic | Chr02 | 23,280,198 | 23,283,279 | + | 3,082 |
| Gorai.002G104400 | RLK1 | G-type lectin S-receptor-like serine/threonine-protein kinase RLK1 | Chr02 | 13,497,346 | 13,499,907 | + | 2,562 |
| Gorai.002G103000 | HSC80 | Heat shock cognate protein 80 | Chr02 | 13,118,219 | 13,121,608 | - | 3,390 |
| Gorai.002G256700 | LECRKS4 | L-type lectin-domain containing receptor kinase S.4 | Chr02 | 61,871,757 | 61,874,150 | + | 2,394 |
| Gorai.002G256800 | LECRKS4 | L-type lectin-domain containing receptor kinase S.4 | Chr02 | 61,879,161 | 61,882,206 | + | 3,046 |
| Gorai.002G063300 | At3g15020 | Malate dehydrogenase 2, mitochondrial | Chr02 | 7,415,313 | 7,418,604 | - | 3,292 |
| Gorai.002G038500 | G9 | Polygalacturonase | Chr02 | 3,077,722 | 3,079,801 | - | 2,080 |
| Gorai.002G103300 | At4g26540 | Probable LRR receptor-like serine/threonine-protein kinase At4g26540 | Chr02 | 13,133,249 | 13,137,153 | + | 3,905 |
| Gorai.002G096100 | WNK11 | Probable serine/threonine-protein kinase WNK11 | Chr02 | 12,124,912 | 12,126,689 | - | 1,778 |
| Gorai.002G095800 | TRN1 | Protein TORNADO 1 | Chr02 | 12,108,790 | 12,113,667 | - | 4,878 |
| Gorai.002G096000 | At1g64210 | Putative inactive receptor-like protein kinase At1g64210 | Chr02 | 12,121,983 | 12,123,813 | - | 1,831 |
| Gorai.002G088600 | RIDA | Reactive Intermediate Deaminase A, chloroplastic | Chr02 | 11,193,387 | 11,194,572 | - | 1,186 |
| Gorai.002G122500 | RBK2 | Receptor-like cytosolic serine/threonine-protein kinase RBK2 | Chr02 | 17,602,615 | 17,606,296 | - | 3,682 |
| Gorai.002G235300 | TMK3 | Receptor-like kinase TMK3 | Chr02 | 59,576,586 | 59,580,461 | + | 3,876 |
| Gorai.002G067900 | svkA | Serine/threonine-protein kinase svkA | Chr02 | 7,883,154 | 7,892,417 | - | 9,264 |
| Gorai.002G032600 | WAG2 | Serine/threonine-protein kinase WAG2 | Chr02 | 2,437,483 | 2,438,790 | - | 1,308 |
| Gorai.002G256500 | TAC1 | Transcriptional regulator TAC1 | Chr02 | 61,860,042 | 61,860,657 | + | 616 |
| Gorai.003G012100 | ROT3 | 3-epi-6-deoxocathasterone 23-monooxygenase | Chr03 | 773,760 | 781,101 | - | 7,342 |
| Gorai.003G093000 | PYD3 | Beta-ureidopropionase | Chr03 | 28,363,894 | 28,364,543 | + | 650 |
| Gorai.003G009500 | CPK28 | Calcium-dependent protein kinase 28 | Chr03 | 578,105 | 583,466 | - | 5,362 |
| Gorai.003G009300 | ATJ11 | Chaperone protein dnaJ 11, chloroplastic | Chr03 | 567,991 | 568,296 | - | 306 |
| Gorai.003G132600 | SMR3 | Cyclin-dependent protein kinase inhibitor SMR3 | Chr03 | 38,841,002 | 38,843,067 | + | 2,066 |
| Gorai.003G002000 | MLH3 | DNA mismatch repair protein MLH3 | Chr03 | 111,645 | 120,535 | + | 8,891 |
| Gorai.003G126400 | Polr1d | DNA-directed RNA polymerases I and III subunit RPAC2 | Chr03 | 37,666,711 | 37,668,505 | - | 1,795 |
| Gorai.003G008400 | AHK1 | Histidine kinase 1 | Chr03 | 503,618 | 510,333 | - | 6,716 |
| Gorai.003G155700 | MPK4 | Mitogen-activated protein kinase 4 | Chr03 | 42,470,910 | 42,474,929 | + | 4,020 |
| Gorai.003G111100 | At4g02580 | NADH dehydrogenase [ubiquinone] flavoprotein 2, mitochondrial | Chr03 | 34,027,440 | 34,029,449 | + | 2,010 |
| Gorai.003G030900 | NAGS2 | Probable amino-acid acetyltransferase NAGS2, chloroplastic | Chr03 | 2,815,754 | 2,824,922 | - | 9,169 |
| Gorai.003G016700 | At4g36750 | Probable NAD(P)H dehydrogenase (quinone) FQR1-like 2 | Chr03 | 1,164,570 | 1,166,715 | + | 2,146 |
| Gorai.003G155800 | GSVIVT00026920001 | Probable polygalacturonase | Chr03 | 42,474,726 | 42,477,381 | - | 2,656 |
| Gorai.003G126600 | At5g41260 | Probable serine/threonine-protein kinase At5g41260 | Chr03 | 37,670,473 | 37,674,418 | - | 3,946 |
| Gorai.003G002600 | At4g35600 | Probable serine/threonine-protein kinase Cx32, chloroplastic | Chr03 | 153,919 | 158,622 | + | 4,704 |
| Gorai.003G083900 | APK2B | Protein kinase 2B, chloroplastic | Chr03 | 21,084,297 | 21,087,659 | - | 3,363 |
| Gorai.003G154200 | NA | Pyruvate kinase, cytosolic isozyme | Chr03 | 42,312,112 | 42,315,476 | + | 3,365 |
| Gorai.003G012000 | DIM1B | Ribosomal RNA small subunit methyltransferase, mitochondrial | Chr03 | 770,168 | 772,337 | + | 2,170 |
| Gorai.003G119300 | Os02g0512300 | RNA pseudouridine synthase 7 | Chr03 | 35,843,891 | 35,855,882 | + | 11,992 |
| Gorai.003G001800 | NA | Serine/threonine-protein kinase SRPK | Chr03 | 105,012 | 108,245 | + | 3,234 |
| Gorai.004G129000 | At1g64710 | Alcohol dehydrogenase-like 4 | Chr04 | 34,309,214 | 34,311,804 | - | 2,591 |
| Gorai.004G262400 | At5g24760 | Alcohol dehydrogenase-like 6 | Chr04 | 59,777,292 | 59,781,251 | - | 3,960 |
| Gorai.004G209200 | UGE1 | Bifunctional UDP-glucose 4-epimerase and UDP-xylose 4-epimerase 1 | Chr04 | 54,162,235 | 54,165,390 | - | 3,156 |
| Gorai.004G209000 | RH47 | DEAD-box ATP-dependent RNA helicase 47, mitochondrial | Chr04 | 54,157,704 | 54,160,653 | + | 2,950 |
| Gorai.004G134500 | Os07g0301200 | DEAD-box ATP-dependent RNA helicase 5 | Chr04 | 36,921,679 | 36,923,819 | + | 2,141 |
| Gorai.004G262200 | RHL1 | DNA-binding protein RHL1 | Chr04 | 59,763,364 | 59,773,441 | - | 10,078 |
| Gorai.004G210400 | rpoC2 | DNA-directed RNA polymerase subunit beta'' | Chr04 | 54,292,739 | 54,293,814 | - | 1,076 |
| Gorai.004G229300 | DRB2 | Double-stranded RNA-binding protein 2 | Chr04 | 56,383,012 | 56,386,610 | + | 3,599 |
| Gorai.004G053300 | NA | Dynamin-related protein 5A | Chr04 | 4,917,438 | 4,919,596 | - | 2,159 |
| Gorai.004G133700 | At1g64390 | Endoglucanase 6 | Chr04 | 36,626,402 | 36,631,042 | - | 4,641 |
| Gorai.004G034200 | VCS | Enhancer of mRNA-decapping protein 4 | Chr04 | 2,799,018 | 2,805,771 | + | 6,754 |
| Gorai.004G138800 | ALG11 | GDP-Man:Man(3)GlcNAc(2)-PP-Dol alpha-1,2-mannosyltransferase | Chr04 | 38,882,227 | 38,883,496 | + | 1,270 |
| Gorai.004G241500 | CAISE5 | Glucose and ribitol dehydrogenase | Chr04 | 57,971,110 | 57,972,657 | - | 1,548 |
| Gorai.004G034000 | HSC80 | Heat shock cognate protein 80 | Chr04 | 2,786,127 | 2,789,058 | - | 2,932 |
| Gorai.004G220400 | H6H | Hyoscyamine 6-dioxygenase | Chr04 | 55,524,164 | 55,525,862 | + | 1,699 |
| Gorai.004G220500 | H6H | Hyoscyamine 6-dioxygenase | Chr04 | 55,533,589 | 55,535,269 | + | 1,681 |
| Gorai.004G220600 | H6H | Hyoscyamine 6-dioxygenase | Chr04 | 55,536,378 | 55,538,894 | + | 2,517 |
| Gorai.004G220700 | H6H | Hyoscyamine 6-dioxygenase | Chr04 | 55,543,667 | 55,545,244 | + | 1,578 |
| Gorai.004G135500 | PHY1 | Light-sensor Protein kinase | Chr04 | 37,337,349 | 37,340,437 | - | 3,089 |
| Gorai.004G232400 | NPK1 | Mitogen-activated protein kinase kinase kinase NPK1 | Chr04 | 56,931,124 | 56,932,679 | + | 1,556 |
| Gorai.004G201100 | NAC007 | NAC domain-containing protein 7 | Chr04 | 52,722,129 | 52,725,755 | - | 3,627 |
| Gorai.004G268400 | NAC086 | NAC domain-containing protein 86 | Chr04 | 60,338,982 | 60,342,509 | - | 3,528 |
| Gorai.004G227000 | NAC091 | NAC domain-containing protein 91 | Chr04 | 56,133,391 | 56,136,326 | + | 2,936 |
| Gorai.004G253100 | CBR1 | NADH--cytochrome b5 reductase 1 | Chr04 | 59,036,710 | 59,039,857 | + | 3,148 |
| Gorai.004G037600 | NA | Polygalacturonase | Chr04 | 3,105,089 | 3,109,017 | + | 3,929 |
| Gorai.004G223200 | YUC8 | Probable indole-3-pyruvate monooxygenase YUCCA8 | Chr04 | 55,775,230 | 55,776,835 | + | 1,606 |
| Gorai.004G241400 | LOX1.5 | Probable linoleate 9S-lipoxygenase 5 | Chr04 | 57,956,547 | 57,960,683 | - | 4,137 |
| Gorai.004G210200 | At1g63430 | Probable LRR receptor-like serine/threonine-protein kinase At1g63430 | Chr04 | 54,265,497 | 54,270,104 | + | 4,608 |
| Gorai.004G214800 | RKF3 | Probable LRR receptor-like serine/threonine-protein kinase RKF3 | Chr04 | 54,773,628 | 54,775,622 | + | 1,995 |
| Gorai.004G262100 | YSL7 | Probable metal-nicotianamine transporter YSL7 | Chr04 | 59,755,472 | 59,762,587 | + | 7,116 |
| Gorai.004G134000 | GSVIVT00026920001 | Probable polygalacturonase | Chr04 | 36,758,798 | 36,763,777 | + | 4,980 |
| Gorai.004G124700 | ARP1 | Probable RNA-binding protein ARP1 | Chr04 | 32,508,395 | 32,510,758 | + | 2,364 |
| Gorai.004G184400 | TRN2 | Protein TORNADO 2 | Chr04 | 49,842,253 | 49,843,758 | + | 1,506 |
| Gorai.004G266000 | WHY2 | Single-stranded DNA-bindig protein WHY2, mitochondrial | Chr04 | 60,141,784 | 60,146,168 | + | 4,385 |
| Gorai.004G200900 | TL20.3 | Thylakoid lumenal protein TL20.3, chloroplastic | Chr04 | 52,702,348 | 52,707,611 | + | 5,264 |
| Gorai.005G245400 | NA | Bifunctional dihydrofolate reductase-thymidylate synthase | Chr05 | 62,562,792 | 62,567,951 | - | 5,160 |
| Gorai.005G246000 | HISN7 | Bifunctional phosphatase IMPL2, chloroplastic | Chr05 | 62,618,761 | 62,621,374 | + | 2,614 |
| Gorai.005G216500 | CPK4 | Calcium-dependent protein kinase 4 | Chr05 | 59,941,505 | 59,946,021 | - | 4,517 |
| Gorai.005G021200 | RH21 | DEAD-box ATP-dependent RNA helicase 21 | Chr05 | 1,744,957 | 1,747,872 | - | 2,916 |
| Gorai.005G233700 | DDB2 | DNA damage-binding protein 2 | Chr05 | 61,593,261 | 61,603,281 | + | 10,021 |
| Gorai.005G056900 | Dnajb12 | DnaJ homolog subfamily B member 12 | Chr05 | 5,766,904 | 5,771,242 | - | 4,339 |
| Gorai.005G217700 | ARC5 | Dynamin-like protein ARC5 | Chr05 | 60,063,908 | 60,070,310 | - | 6,403 |
| Gorai.005G057300 | BAM9 | Inactive beta-amylase 9 | Chr05 | 5,805,609 | 5,808,605 | - | 2,997 |
| Gorai.005G217600 | LECRK42 | L-type lectin-domain containing receptor kinase IV.2 | Chr05 | 60,061,316 | 60,063,623 | + | 2,308 |
| Gorai.005G050200 | MD1 | Malate dehydrogenase | Chr05 | 4,944,349 | 4,947,387 | - | 3,039 |
| Gorai.005G226800 | ALDH6B2 | Methylmalonate-semialdehyde dehydrogenase [acylating], mitochondrial | Chr05 | 60,923,990 | 60,930,360 | - | 6,371 |
| Gorai.005G226900 | ALDH6B2 | Methylmalonate-semialdehyde dehydrogenase [acylating], mitochondrial | Chr05 | 60,931,081 | 60,940,609 | - | 9,529 |
| Gorai.005G011100 | MMK1 | Mitogen-activated protein kinase homolog MMK1 | Chr05 | 764,244 | 768,637 | + | 4,394 |
| Gorai.005G217400 | NAM-B2 | NAC transcription factor NAM-B2 | Chr05 | 60,047,648 | 60,049,783 | - | 2,136 |
| Gorai.005G011400 | NA | Natterin-4 | Chr05 | 781,201 | 783,627 | - | 2,427 |
| Gorai.005G053600 | PRP19 | Pre-mRNA-processing factor 19 | Chr05 | 5,295,835 | 5,301,542 | + | 5,708 |
| Gorai.005G050300 | At1g65660 | Pre-mRNA-splicing factor SLU7-A | Chr05 | 4,950,723 | 4,954,873 | + | 4,151 |
| Gorai.005G193500 | At1g66830 | Probable inactive leucine-rich repeat receptor-like protein kinase At1g66830 | Chr05 | 56,314,506 | 56,317,700 | + | 3,195 |
| Gorai.005G239500 | RKL1 | Probable inactive receptor kinase At1g48480 | Chr05 | 62,080,730 | 62,082,565 | - | 1,836 |
| Gorai.005G002700 | At1g06840 | Probable LRR receptor-like serine/threonine-protein kinase At1g06840 | Chr05 | 136,764 | 144,255 | - | 7,492 |
| Gorai.005G246200 | At4g39110 | Probable receptor-like protein kinase At4g39110 | Chr05 | 62,624,574 | 62,627,365 | + | 2,792 |
| Gorai.005G217300 | At4g35600 | Probable serine/threonine-protein kinase Cx32, chloroplastic | Chr05 | 60,035,993 | 60,039,749 | + | 3,757 |
| Gorai.005G016800 | osgep | Probable tRNA N6-adenosine threonylcarbamoyltransferase | Chr05 | 1,223,945 | 1,227,816 | + | 3,872 |
| Gorai.005G107000 | BZR1 | Protein BRASSINAZOLE-RESISTANT 1 | Chr05 | 19,206,384 | 19,208,322 | - | 1,939 |
| Gorai.005G238300 | At2g14440 | Putative leucine-rich repeat receptor-like serine/threonine-protein kinase At2g14440 | Chr05 | 61,977,772 | 61,982,819 | + | 5,048 |
| Gorai.005G237900 | NA | Pyruvate kinase, cytosolic isozyme | Chr05 | 61,962,363 | 61,962,602 | - | 240 |
| Gorai.005G112100 | CLV1 | Receptor protein kinase CLAVATA1 | Chr05 | 20,969,345 | 20,972,966 | - | 3,622 |
| Gorai.005G067100 | ANX2 | Receptor-like protein kinase ANXUR2 | Chr05 | 7,148,352 | 7,150,898 | - | 2,547 |
| Gorai.005G016300 | CPL4 | RNA polymerase II C-terminal domain phosphatase-like 4 | Chr05 | 1,195,206 | 1,201,272 | - | 6,067 |
| Gorai.005G246500 | RBM28 | RNA-binding protein 28 | Chr05 | 62,653,251 | 62,657,895 | + | 4,645 |
| Gorai.005G001500 | At5g01020 | Serine/threonine-protein kinase At5g01020 | Chr05 | 89,115 | 93,142 | + | 4,028 |
| Gorai.005G057000 | RUK | Serine/threonine-protein kinase RUNKEL | Chr05 | 5,776,993 | 5,783,612 | + | 6,620 |
| Gorai.005G003400 | STN8 | Serine/threonine-protein kinase STN8, chloroplastic | Chr05 | 181,340 | 184,270 | - | 2,931 |
| Gorai.005G144700 | UGD5 | UDP-glucose 6-dehydrogenase 5 | Chr05 | 39,204,541 | 39,207,343 | - | 2,803 |
| Gorai.006G090300 | FAB1B | 1-phosphatidylinositol-3-phosphate 5-kinase FAB1B | Chr06 | 32,656,297 | 32,663,732 | + | 7,436 |
| Gorai.006G119500 | ADT2 | Arogenate dehydratase/prephenate dehydratase 2, chloroplastic | Chr06 | 36,925,796 | 36,930,800 | - | 5,005 |
| Gorai.006G103200 | At1g65240 | Aspartic proteinase-like protein 2 | Chr06 | 34,398,913 | 34,402,633 | - | 3,721 |
| Gorai.006G248000 | BET12 | Bet1-like SNARE 1-2 | Chr06 | 49,287,257 | 49,290,151 | - | 2,895 |
| Gorai.006G122800 | EPHX2 | Bifunctional epoxide hydrolase 2 | Chr06 | 37,429,689 | 37,431,626 | - | 1,938 |
| Gorai.006G233100 | shkD | Dual specificity protein kinase shkD | Chr06 | 48,193,586 | 48,200,132 | - | 6,547 |
| Gorai.006G174000 | HSP70 | Heat shock cognate 70 kDa protein | Chr06 | 43,274,425 | 43,277,516 | - | 3,092 |
| Gorai.006G120500 | Os05g0125500 | Isovaleryl-CoA dehydrogenase, mitochondrial | Chr06 | 37,082,308 | 37,087,904 | - | 5,597 |
| Gorai.006G131800 | PXC3 | Leucine-rich repeat receptor-like tyrosine-protein kinase PXC3 | Chr06 | 38,618,612 | 38,622,419 | - | 3,808 |
| Gorai.006G127700 | ONAC010 | NAC transcription factor ONAC010 | Chr06 | 38,066,641 | 38,067,848 | + | 1,208 |
| Gorai.006G119400 | At5g47890 | NADH dehydrogenase [ubiquinone] 1 alpha subcomplex subunit 2 | Chr06 | 36,923,721 | 36,926,437 | + | 2,717 |
| Gorai.006G004900 | Nrd1 | Nardilysin | Chr06 | 955,069 | 958,033 | - | 2,965 |
| Gorai.006G186600 | NA | Polygalacturonase | Chr06 | 44,363,049 | 44,364,677 | - | 1,629 |
| Gorai.006G020800 | At4g08850 | Probable LRR receptor-like serine/threonine-protein kinase At4g08850 | Chr06 | 5,217,456 | 5,224,386 | - | 6,931 |
| Gorai.006G165500 | At5g47070 | Probable receptor-like protein kinase At5g47070 | Chr06 | 42,522,740 | 42,526,695 | - | 3,956 |
| Gorai.006G103100 | AGO5 | Protein argonaute 5 | Chr06 | 34,390,988 | 34,396,856 | + | 5,869 |
| Gorai.006G233700 | PK1 | Putative receptor protein kinase ZmPK1 | Chr06 | 48,230,986 | 48,233,502 | + | 2,517 |
| Gorai.006G165200 | SIGF | RNA polymerase sigma factor sigF, chloroplastic | Chr06 | 42,500,097 | 42,503,417 | - | 3,321 |
| Gorai.006G056600 | EDR1 | Serine/threonine-protein kinase EDR1 | Chr06 | 20,681,725 | 20,698,700 | - | 16,976 |
| Gorai.006G111800 | TGA21 | TGACG-sequence-specific DNA-binding protein TGA-2.1 | Chr06 | 35,932,264 | 35,937,880 | - | 5,617 |
| Gorai.006G220300 | coq6 | Ubiquinone biosynthesis monooxygenase COQ6, mitochondrial | Chr06 | 47,280,044 | 47,283,955 | + | 3,912 |
| Gorai.006G117900 | UBP8 | Ubiquitin carboxyl-terminal hydrolase 8 | Chr06 | 36,740,511 | 36,751,665 | - | 11,155 |
| Gorai.006G018200 | WAKL1 | Wall-associated receptor kinase-like 1 | Chr06 | 4,254,838 | 4,259,383 | - | 4,546 |
| Gorai.006G018600 | WAKL2 | Wall-associated receptor kinase-like 2 | Chr06 | 4,597,252 | 4,598,798 | - | 1,547 |
| Gorai.006G018700 | WAKL22 | Wall-associated receptor kinase-like 22 | Chr06 | 4,601,409 | 4,605,697 | - | 4,289 |
| Gorai.006G018800 | WAKL22 | Wall-associated receptor kinase-like 22 | Chr06 | 4,606,974 | 4,613,186 | - | 6,213 |
| Gorai.007G347100 | DAPB2 | 4-hydroxy-tetrahydrodipicolinate reductase 2, chloroplastic | Chr07 | 57,748,162 | 57,753,918 | - | 5,757 |
| Gorai.007G091100 | KU80 | ATP-dependent DNA helicase 2 subunit KU80 | Chr07 | 6,608,439 | 6,613,508 | - | 5,070 |
| Gorai.007G043800 | SWEET12 | Bidirectional sugar transporter SWEET12 | Chr07 | 3,023,940 | 3,025,406 | - | 1,467 |
| Gorai.007G304300 | RH10 | DEAD-box ATP-dependent RNA helicase 10 | Chr07 | 51,812,182 | 51,818,395 | - | 6,214 |
| Gorai.007G362700 | RH37 | DEAD-box ATP-dependent RNA helicase 37 | Chr07 | 59,429,333 | 59,434,244 | - | 4,912 |
| Gorai.007G374300 | RH57 | DEAD-box ATP-dependent RNA helicase 57 | Chr07 | 60,633,068 | 60,637,091 | + | 4,024 |
| Gorai.007G022200 | NRPD1 | DNA-directed RNA polymerase IV subunit 1 | Chr07 | 1,605,147 | 1,612,611 | - | 7,465 |
| Gorai.007G033100 | ITPK1 | Inositol-tetrakisphosphate 1-kinase 1 | Chr07 | 2,253,121 | 2,257,186 | + | 4,066 |
| Gorai.007G340200 | GSO1 | LRR receptor-like serine/threonine-protein kinase GSO1 | Chr07 | 56,560,323 | 56,563,345 | - | 3,023 |
| Gorai.007G099500 | MED12 | Mediator of RNA polymerase II transcription subunit 12 | Chr07 | 7,339,955 | 7,353,492 | - | 13,538 |
| Gorai.007G378000 | Rngtt | mRNA-capping enzyme | Chr07 | 60,848,463 | 60,856,120 | + | 7,658 |
| Gorai.007G043900 | NAC083 | NAC domain-containing protein 83 | Chr07 | 3,033,324 | 3,035,348 | + | 2,025 |
| Gorai.007G369400 | ndhA | NAD(P)H-quinone oxidoreductase subunit 1, chloroplastic | Chr07 | 60,221,997 | 60,222,320 | - | 324 |
| Gorai.007G084700 | PIP5K8 | Phosphatidylinositol 4-phosphate 5-kinase 8 | Chr07 | 6,056,701 | 6,060,050 | - | 3,350 |
| Gorai.007G360000 | NA | Polygalacturonase | Chr07 | 59,114,593 | 59,119,141 | + | 4,549 |
| Gorai.007G093500 | GAUT1 | Polygalacturonate 4-alpha-galacturonosyltransferase | Chr07 | 6,832,789 | 6,837,085 | - | 4,297 |
| Gorai.007G335100 | At1g67000 | Probable receptor-like protein kinase At1g67000 | Chr07 | 55,952,983 | 55,956,179 | + | 3,197 |
| Gorai.007G084600 | At5g47070 | Probable receptor-like protein kinase At5g47070 | Chr07 | 6,040,590 | 6,043,865 | - | 3,276 |
| Gorai.007G286400 | SDE3 | Probable RNA helicase SDE3 | Chr07 | 49,214,379 | 49,218,710 | - | 4,332 |
| Gorai.007G377000 | RBE | Probable transcriptional regulator RABBIT EARS | Chr07 | 60,806,990 | 60,807,880 | - | 891 |
| Gorai.007G043700 | TPD1 | Protein TAPETUM DETERMINANT 1 | Chr07 | 3,019,243 | 3,021,827 | + | 2,585 |
| Gorai.007G084900 | At2g44680 | Putative casein kinase II subunit beta-4 | Chr07 | 6,064,111 | 6,067,952 | - | 3,842 |
| Gorai.007G274500 | FER | Receptor-like protein kinase FERONIA | Chr07 | 46,811,592 | 46,814,191 | + | 2,600 |
| Gorai.007G022300 | v1g245966 | Ribosomal RNA processing protein 36 homolog | Chr07 | 1,619,158 | 1,622,547 | - | 3,390 |
| Gorai.007G347300 | SIGB | RNA polymerase sigma factor sigB | Chr07 | 57,758,649 | 57,762,734 | - | 4,086 |
| Gorai.007G370900 | KIPK | Serine/threonine-protein kinase KIPK | Chr07 | 60,372,052 | 60,376,462 | + | 4,411 |
| Gorai.007G378500 | At5g14050 | U3 small nucleolar RNA-associated protein 18 homolog | Chr07 | 60,879,594 | 60,882,466 | - | 2,873 |
| Gorai.007G335200 | UBP15 | Ubiquitin carboxyl-terminal hydrolase 15 | Chr07 | 55,958,324 | 55,966,086 | - | 7,763 |
| Gorai.007G090500 | GAE3 | UDP-glucuronate 4-epimerase 3 | Chr07 | 6,591,543 | 6,593,384 | + | 1,842 |
| Gorai.008G062200 | DHQS | 3-dehydroquinate synthase, chloroplastic | Chr08 | 9,943,621 | 9,948,228 | - | 4,608 |
| Gorai.008G049500 | EMB1027 | Arginine--tRNA ligase, chloroplastic/mitochondrial | Chr08 | 6,980,808 | 6,983,870 | + | 3,063 |
| Gorai.008G192200 | rpoC1 | DNA-directed RNA polymerase subunit beta' | Chr08 | 47,552,427 | 47,552,627 | - | 201 |
| Gorai.008G042900 | LGALDH | L-galactose dehydrogenase | Chr08 | 5,705,951 | 5,708,454 | - | 2,504 |
| Gorai.008G109400 | NA | Nucleoside diphosphate kinase B | Chr08 | 34,045,785 | 34,047,453 | + | 1,669 |
| Gorai.008G291100 | At1g72550 | Phenylalanine--tRNA ligase beta subunit, cytoplasmic | Chr08 | 56,560,932 | 56,569,949 | - | 9,018 |
| Gorai.008G222400 | NA | Phosphoribulokinase, chloroplastic | Chr08 | 50,859,664 | 50,862,466 | - | 2,803 |
| Gorai.008G135600 | At2g47060 | Probable receptor-like protein kinase At2g47060 | Chr08 | 38,381,303 | 38,384,894 | + | 3,592 |
| Gorai.008G283700 | At3g22660 | Probable rRNA-processing protein EBP2 homolog | Chr08 | 55,957,888 | 55,958,728 | - | 841 |
| Gorai.008G151500 | At3g62120 | Proline--tRNA ligase, cytoplasmic | Chr08 | 40,830,418 | 40,834,229 | + | 3,812 |
| Gorai.008G106400 | Noca_2408 | Putative pre-16S rRNA nuclease | Chr08 | 33,445,401 | 33,447,771 | - | 2,371 |
| Gorai.008G128200 | EMG1 | Ribosomal RNA small subunit methyltransferase NEP1 | Chr08 | 36,990,144 | 36,993,024 | - | 2,881 |
| Gorai.008G249100 | Emg1 | Ribosomal RNA small subunit methyltransferase NEP1 | Chr08 | 53,326,027 | 53,327,722 | + | 1,696 |
| Gorai.008G115000 | SYT4 | Synaptotagmin-4 | Chr08 | 34,752,234 | 34,758,912 | + | 6,679 |
| Gorai.008G115100 | URT1 | UTP:RNA uridylyltransferase 1 | Chr08 | 34,763,648 | 34,767,985 | + | 4,338 |
| Gorai.009G262500 | GL12933 | Bifunctional lysine-specific demethylase and histidyl-hydroxylase NO66 | Chr09 | 21,684,761 | 21,690,816 | - | 6,056 |
| Gorai.009G078000 | CPK17 | Calcium-dependent protein kinase 17 | Chr09 | 5,620,081 | 5,622,436 | + | 2,356 |
| Gorai.009G251300 | CPK3 | Calcium-dependent protein kinase 3 | Chr09 | 20,460,232 | 20,463,369 | - | 3,138 |
| Gorai.009G222500 | dnaJ | Chaperone protein DnaJ | Chr09 | 17,312,004 | 17,315,623 | - | 3,620 |
| Gorai.009G307700 | SMR3 | Cyclin-dependent protein kinase inhibitor SMR3 | Chr09 | 28,221,814 | 28,222,733 | + | 920 |
| Gorai.009G031100 | CDA1 | Cytidine deaminase 1 | Chr09 | 2,346,693 | 2,347,607 | + | 915 |
| Gorai.009G040700 | CKX7 | Cytokinin dehydrogenase 7 | Chr09 | 3,002,855 | 3,006,351 | - | 3,497 |
| Gorai.009G087200 | POL2A | DNA polymerase epsilon catalytic subunit A | Chr09 | 6,339,628 | 6,359,176 | - | 19,549 |
| Gorai.009G085200 | POLE3 | DNA polymerase epsilon subunit 3 | Chr09 | 6,202,258 | 6,203,339 | - | 1,082 |
| Gorai.009G130400 | GINS1 | DNA replication complex GINS protein PSF1 | Chr09 | 9,817,517 | 9,820,992 | - | 3,476 |
| Gorai.009G011600 | DNAJB4 | DnaJ homolog subfamily B member 4 | Chr09 | 954,787 | 958,120 | - | 3,334 |
| Gorai.009G052000 | YUC6 | Indole-3-pyruvate monooxygenase YUCCA6 | Chr09 | 3,738,723 | 3,742,006 | - | 3,284 |
| Gorai.009G010500 | TDR | Leucine-rich repeat receptor-like protein kinase TDR | Chr09 | 848,861 | 851,950 | - | 3,090 |
| Gorai.009G010400 | GSO2 | LRR receptor-like serine/threonine-protein kinase GSO2 | Chr09 | 838,538 | 843,236 | - | 4,699 |
| Gorai.009G041100 | ndhU | NAD(P)H-quinone oxidoreductase subunit U, chloroplastic | Chr09 | 3,027,383 | 3,028,809 | - | 1,427 |
| Gorai.009G229300 | NA | NADPH--cytochrome P450 reductase | Chr09 | 18,024,300 | 18,029,945 | + | 5,646 |
| Gorai.009G036600 | PRK3 | Pollen receptor-like kinase 3 | Chr09 | 2,698,605 | 2,701,437 | - | 2,833 |
| Gorai.009G127700 | Prpf8 | Pre-mRNA-processing-splicing factor 8 | Chr09 | 9,618,231 | 9,629,371 | + | 11,141 |
| Gorai.009G229700 | prpf18 | Pre-mRNA-splicing factor 18 | Chr09 | 18,058,014 | 18,059,941 | + | 1,928 |
| Gorai.009G042200 | NSUN5 | Probable 28S rRNA (cytosine-C(5))-methyltransferase | Chr09 | 3,084,487 | 3,088,759 | - | 4,273 |
| Gorai.009G262600 | ADAT3 | Probable inactive tRNA-specific adenosine deaminase-like protein 3 | Chr09 | 21,691,948 | 21,700,556 | - | 8,609 |
| Gorai.009G313800 | At3g47570 | Probable LRR receptor-like serine/threonine-protein kinase At3g47570 | Chr09 | 29,404,907 | 29,408,216 | - | 3,310 |
| Gorai.009G036700 | GSVIVT00026920001 | Probable polygalacturonase | Chr09 | 2,701,971 | 2,705,238 | + | 3,268 |
| Gorai.009G251100 | PERK13 | Proline-rich receptor-like protein kinase PERK13 | Chr09 | 20,445,856 | 20,449,971 | + | 4,116 |
| Gorai.009G127800 | At3g15890 | PTI1-like tyrosine-protein kinase At3g15890 | Chr09 | 9,629,423 | 9,633,132 | - | 3,710 |
| Gorai.009G149200 | RCA | Ribulose bisphosphate carboxylase/oxygenase activase, chloroplastic | Chr09 | 11,329,963 | 11,335,158 | + | 5,196 |
| Gorai.009G176900 | At4g21770 | RNA pseudouridine synthase 6, chloroplastic | Chr09 | 13,682,024 | 13,686,039 | - | 4,016 |
| Gorai.009G042700 | CBK1 | Serine/threonine-protein kinase CBK1 | Chr09 | 3,109,325 | 3,116,522 | - | 7,198 |
| Gorai.009G149600 | fray2 | Serine/threonine-protein kinase fray2 | Chr09 | 11,356,315 | 11,364,533 | - | 8,219 |
| Gorai.009G078200 | GRIK2 | Serine/threonine-protein kinase GRIK2 | Chr09 | 5,625,499 | 5,630,091 | - | 4,593 |
| Gorai.009G130600 | SEC11C | Signal peptidase complex catalytic subunit SEC11C | Chr09 | 9,825,322 | 9,829,108 | - | 3,787 |
| Gorai.009G128200 | PV42A | SNF1-related protein kinase regulatory subunit gamma-like PV42a | Chr09 | 9,643,598 | 9,645,237 | + | 1,640 |
| Gorai.009G086200 | TGA21 | TGACG-sequence-specific DNA-binding protein TGA-2.1 | Chr09 | 6,268,476 | 6,274,678 | + | 6,203 |
| Gorai.009G328200 | ADA2 | Transcriptional adapter ADA2 | Chr09 | 32,977,411 | 32,983,000 | - | 5,590 |
| Gorai.010G080400 | SYNC1 | Asparagine--tRNA ligase, cytoplasmic 1 | Chr10 | 11,729,817 | 11,732,642 | + | 2,826 |
| Gorai.010G008300 | BBD1 | Bifunctional nuclease 1 | Chr10 | 587,956 | 591,062 | + | 3,107 |
| Gorai.010G252400 | CPK21 | Calcium-dependent protein kinase 21 | Chr10 | 61,853,992 | 61,856,356 | - | 2,365 |
| Gorai.010G060400 | CRCK3 | Calmodulin-binding receptor-like cytoplasmic kinase 3 | Chr10 | 7,221,506 | 7,226,509 | - | 5,004 |
| Gorai.010G253400 | CRK25 | Cysteine-rich receptor-like protein kinase 25 | Chr10 | 61,913,038 | 61,916,698 | + | 3,661 |
| Gorai.010G008400 | IPK1 | Inositol-pentakisphosphate 2-kinase | Chr10 | 591,395 | 598,004 | - | 6,610 |
| Gorai.010G078900 | NAC073 | NAC domain-containing protein 73 | Chr10 | 11,417,835 | 11,423,463 | - | 5,629 |
| Gorai.010G064600 | EMB1187 | Probable ethanolamine kinase | Chr10 | 8,226,446 | 8,231,523 | + | 5,078 |
| Gorai.010G218400 | IRK | Probable LRR receptor-like serine/threonine-protein kinase IRK | Chr10 | 58,720,017 | 58,724,035 | + | 4,019 |
| Gorai.010G074600 | At2g20050/At2g20040 | Protein phosphatase 2C and cyclic nucleotide-binding/kinase domain-containing protein | Chr10 | 10,616,511 | 10,623,751 | + | 7,241 |
| Gorai.010G253500 | CRK35 | Putative cysteine-rich receptor-like protein kinase 35 | Chr10 | 61,918,588 | 61,922,570 | + | 3,983 |
| Gorai.010G064400 | SEC11C | Signal peptidase complex catalytic subunit SEC11C | Chr10 | 8,203,967 | 8,207,647 | + | 3,681 |
| Gorai.010G252100 | tdp2 | Tyrosyl-DNA phosphodiesterase 2 | Chr10 | 61,841,587 | 61,843,916 | - | 2,330 |
| Gorai.011G162200 | BAK1 | BRASSINOSTEROID INSENSITIVE 1-associated receptor kinase 1 | Chr11 | 30,386,854 | 30,389,231 | + | 2,378 |
| Gorai.011G232400 | Os01g0172200 | DEAD-box ATP-dependent RNA helicase 14 | Chr11 | 54,943,508 | 54,944,346 | + | 839 |
| Gorai.011G066000 | RH20 | DEAD-box ATP-dependent RNA helicase 20 | Chr11 | 5,584,384 | 5,590,544 | + | 6,161 |
| Gorai.011G008500 | ALKBH2 | DNA oxidative demethylase ALKBH2 | Chr11 | 636,630 | 639,262 | - | 2,633 |
| Gorai.011G135800 | rpoC2 | DNA-directed RNA polymerase subunit beta'' | Chr11 | 20,659,044 | 20,659,750 | - | 707 |
| Gorai.011G057100 | DNAJB6 | DnaJ homolog subfamily B member 6 | Chr11 | 4,522,010 | 4,524,714 | + | 2,705 |
| Gorai.011G131000 | shkC | Dual specificity protein kinase shkC | Chr11 | 19,792,154 | 19,802,769 | + | 10,616 |
| Gorai.011G023000 | NDB2 | External alternative NAD(P)H-ubiquinone oxidoreductase B2, mitochondrial | Chr11 | 1,640,471 | 1,644,994 | + | 4,524 |
| Gorai.011G089400 | FPG1 | Formamidopyrimidine-DNA glycosylase | Chr11 | 9,348,978 | 9,356,445 | - | 7,468 |
| Gorai.011G158900 | SPAC25A8.03c | NADH dehydrogenase [ubiquinone] complex I, assembly factor 7 homolog | Chr11 | 28,720,032 | 28,729,435 | + | 9,404 |
| Gorai.011G108600 | ayr1 | NADPH-dependent 1-acyldihydroxyacetone phosphate reductase | Chr11 | 12,796,727 | 12,797,566 | + | 840 |
| Gorai.011G078200 | NAA16 | N-alpha-acetyltransferase 16, NatA auxiliary subunit | Chr11 | 7,757,571 | 7,771,070 | + | 13,500 |
| Gorai.011G209500 | CYP79A2 | Phenylalanine N-monooxygenase | Chr11 | 50,382,075 | 50,384,372 | - | 2,298 |
| Gorai.011G215600 | PIP5K6 | Phosphatidylinositol 4-phosphate 5-kinase 6 | Chr11 | 51,799,212 | 51,802,855 | - | 3,644 |
| Gorai.011G001900 | PRK4 | Pollen receptor-like kinase 4 | Chr11 | 175,662 | 178,144 | - | 2,483 |
| Gorai.011G205600 | CRSH | Probable GTP diphosphokinase CRSH, chloroplastic | Chr11 | 49,717,913 | 49,721,461 | + | 3,549 |
| Gorai.011G164200 | YUC3 | Probable indole-3-pyruvate monooxygenase YUCCA3 | Chr11 | 31,513,775 | 31,516,061 | - | 2,287 |
| Gorai.011G029600 | At2g14440 | Putative leucine-rich repeat receptor-like serine/threonine-protein kinase At2g14440 | Chr11 | 2,174,274 | 2,179,469 | + | 5,196 |
| Gorai.011G000900 | At2g24130 | Putative leucine-rich repeat receptor-like serine/threonine-protein kinase At2g24130 | Chr11 | 73,908 | 77,201 | - | 3,294 |
| Gorai.011G158300 | LECRKS2 | Receptor like protein kinase S.2 | Chr11 | 28,179,900 | 28,182,398 | + | 2,499 |
| Gorai.011G008200 | INRPK1 | Receptor-like protein kinase | Chr11 | 618,667 | 622,875 | - | 4,209 |
| Gorai.011G162100 | RCH2 | Receptor-like protein kinase 2 | Chr11 | 30,378,807 | 30,382,872 | + | 4,066 |
| Gorai.011G008300 | Rbpms2 | RNA-binding protein with multiple splicing 2 | Chr11 | 625,096 | 630,459 | - | 5,364 |
| Gorai.011G015800 | PBS1 | Serine/threonine-protein kinase PBS1 | Chr11 | 1,104,404 | 1,107,356 | + | 2,953 |
| Gorai.011G022500 | SPHK1 | Sphingosine kinase 1 | Chr11 | 1,579,217 | 1,583,090 | - | 3,874 |
| Gorai.011G069100 | CT0009 | Uncharacterized RNA methyltransferase CT0009 | Chr11 | 6,129,368 | 6,134,688 | + | 5,321 |
| Gorai.012G013700 | POLR1C | DNA-directed RNA polymerases I and III subunit RPAC1 | Chr12 | 1,537,343 | 1,539,788 | - | 2,446 |
| Gorai.012G089700 | G6PDH | Glucose-6-phosphate 1-dehydrogenase, cytoplasmic isoform | Chr12 | 15,358,462 | 15,363,327 | - | 4,866 |
| Gorai.012G023300 | At5g26707 | Glutamate--tRNA ligase, cytoplasmic | Chr12 | 2,868,184 | 2,872,589 | - | 4,406 |
| Gorai.012G124900 | CRN | Inactive leucine-rich repeat receptor-like protein kinase CORYNE | Chr12 | 28,859,421 | 28,862,498 | + | 3,078 |
| Gorai.012G034500 | YDA | Mitogen-activated protein kinase kinase kinase YODA | Chr12 | 4,208,547 | 4,214,124 | + | 5,578 |
| Gorai.012G056900 | pvaA | Polyvinylalcohol dehydrogenase | Chr12 | 7,738,398 | 7,740,248 | - | 1,851 |
| Gorai.012G058000 | At1g27190 | Probable inactive receptor kinase At1g27190 | Chr12 | 7,907,789 | 7,909,947 | - | 2,159 |
| Gorai.012G134500 | IRK | Probable LRR receptor-like serine/threonine-protein kinase IRK | Chr12 | 30,676,352 | 30,678,645 | - | 2,294 |
| Gorai.012G125200 | CPL3 | RNA polymerase II C-terminal domain phosphatase-like 3 | Chr12 | 28,880,827 | 28,887,434 | - | 6,608 |
| Gorai.012G029300 | At3g07070 | Serine/threonine-protein kinase At3g07070 | Chr12 | 3,651,086 | 3,652,909 | - | 1,824 |
| Gorai.012G023400 | D6PKL2 | Serine/threonine-protein kinase D6PKL2 | Chr12 | 2,875,885 | 2,879,885 | - | 4,001 |
| Gorai.012G057700 | D6PKL2 | Serine/threonine-protein kinase D6PKL2 | Chr12 | 7,894,891 | 7,898,428 | + | 3,538 |
| Gorai.012G110300 | nusG | Transcription termination/antitermination protein NusG | Chr12 | 25,044,173 | 25,049,124 | + | 4,952 |
| Gorai.012G096800 | GAE1 | UDP-glucuronate 4-epimerase 1 | Chr12 | 19,632,410 | 19,634,924 | + | 2,515 |
| Gorai.012G013600 | At5g05200 | Uncharacterized aarF domain-containing protein kinase At5g05200, chloroplastic | Chr12 | 1,527,378 | 1,531,957 | - | 4,580 |
| Gorai.013G206000 | SWEET17 | Bidirectional sugar transporter SWEET17 | Chr13 | 51,663,470 | 51,665,134 | - | 1,665 |
| Gorai.013G204800 | At5g38830 | Cysteine--tRNA ligase 2, cytoplasmic | Chr13 | 51,572,626 | 51,575,367 | - | 2,742 |
| Gorai.013G227400 | CKX3 | Cytokinin dehydrogenase 3 | Chr13 | 54,694,905 | 54,698,634 | - | 3,730 |
| Gorai.013G149500 | DDI1 | DNA damage-inducible protein 1 | Chr13 | 40,958,734 | 40,962,818 | + | 4,085 |
| Gorai.013G204500 | ERCC1 | DNA excision repair protein ERCC-1 | Chr13 | 51,558,588 | 51,562,896 | + | 4,309 |
| Gorai.013G227300 | At2g19490 | DNA repair protein recA homolog 3, mitochondrial | Chr13 | 54,687,969 | 54,691,881 | + | 3,913 |
| Gorai.013G047900 | REV1 | DNA repair protein REV1 | Chr13 | 4,238,255 | 4,248,871 | - | 10,617 |
| Gorai.013G072500 | At4g03230 | G-type lectin S-receptor-like serine/threonine-protein kinase At4g03230 | Chr13 | 8,636,452 | 8,640,397 | + | 3,946 |
| Gorai.013G236800 | GSO2 | LRR receptor-like serine/threonine-protein kinase GSO2 | Chr13 | 55,462,862 | 55,469,090 | - | 6,229 |
| Gorai.013G117600 | NA | Pyruvate kinase, cytosolic isozyme | Chr13 | 29,046,313 | 29,048,304 | + | 1,992 |
| Gorai.013G149600 | l(1)G0004 | RNA-binding protein pno1 | Chr13 | 40,962,874 | 40,964,553 | - | 1,680 |
| Gorai.013G204600 | CDL1 | Serine/threonine-protein kinase CDL1 | Chr13 | 51,563,071 | 51,565,381 | - | 2,311 |
