# Supplementary material for: Simple Sequence Repeat (SSR) Genetic Linkage Map of D Genome Diploid Cotton Derived from an Interspecific Cross between Gossypium davidsonii and Gossypium klotzschianum
Source: Int J Mol Sci. 2018 Jan 11;19(1):204. doi: 10.3390/ijms19010204 (PMC5796153; doi:10.3390/ijms19010204)
Supplement: Supplementary file 1 [file ijms-19-00204-s001.zip › ijms-254865 final supplementary/Supplementary Table S3.docx]

Supplementary Table S3: The characteristics of the mined genes from the SSR regions

| Gene ID | Protein Length (aa) | Molecular Weight (kDa) | Charge | Isoelectric Point | Grand Average of Hydropathy | Transcript Length (bp) | CDS Length (bp) | CDS GC Content (%) | Exon Number | Mean Exon Length (bp) | Mean Intron Length (bp) |
| --- | --- | --- | --- | --- | --- | --- | --- | --- | --- | --- | --- |
| Gorai.001G019600 | 437 | 48.401 | 4.5 | 7.025 | -0.022 | 1,903 | 1,314 | 40.6 | 10 | 190.3 | 137 |
| Gorai.001G019700 | 255 | 27.74 | -2 | 5.441 | -0.569 | 1,476 | 768 | 43.4 | 2 | 738 | 91 |
| Gorai.001G019800 | 983 | 108.37 | -5 | 6.189 | -0.021 | 3,307 | 2,952 | 41.8 | 28 | 118.1 | 136.8 |
| Gorai.001G022700 | 296 | 33.184 | -10 | 4.78 | -0.778 | 891 | 891 | 46.8 | 1 | 891 | No intron |
| Gorai.001G022800 | 937 | 104.274 | -8.5 | 5.84 | -0.284 | 3,416 | 2,814 | 43.3 | 13 | 262.8 | 328.2 |
| Gorai.001G022900 | 241 | 27.371 | 1.5 | 6.833 | 0.293 | 1,720 | 726 | 46.6 | 3 | 573.3 | 704 |
| Gorai.001G027600 | 1,319 | 146.374 | 39 | 8.354 | -0.68 | 4,613 | 3,960 | 41.2 | 7 | 659 | 468.2 |
| Gorai.001G027700 | 1,255 | 139.014 | 44 | 8.806 | -0.769 | 4,904 | 3,768 | 41.7 | 7 | 700.6 | 421.7 |
| Gorai.001G027800 | 157 | 17.772 | 4.5 | 8.633 | -0.322 | 990 | 474 | 44.3 | 6 | 165 | 546.6 |
| Gorai.001G050200 | 511 | 55.186 | 12.5 | 9.573 | 0.569 | 1,798 | 1,536 | 43.4 | 3 | 599.3 | 900.5 |
| Gorai.001G050300 | 821 | 92.147 | 8 | 7.275 | -0.501 | 2,724 | 2,466 | 41.9 | 9 | 302.7 | 167.8 |
| Gorai.001G050400 | 319 | 37.191 | -8.5 | 4.828 | -0.116 | 1,294 | 960 | 40.5 | 3 | 431.3 | 269 |
| Gorai.001G050500 | 675 | 74.915 | 17 | 9.038 | -0.105 | 2,028 | 2,028 | 42.3 | 1 | 2,028.00 | No intron |
| Gorai.001G050600 | 416 | 48.264 | 3 | 7.161 | -0.313 | 1,251 | 1,251 | 39.6 | 2 | 625.5 | 30 |
| Gorai.001G052100 | 290 | 32.634 | 6.5 | 8.031 | -0.349 | 2,555 | 873 | 42.8 | 7 | 365 | 160 |
| Gorai.001G052200 | 977 | 108.411 | 34.5 | 9.022 | -0.019 | 2,934 | 2,934 | 43.9 | 15 | 195.6 | 321.9 |
| Gorai.001G052300 | 248 | 28.185 | 4 | 8.447 | -0.544 | 1,080 | 747 | 42 | 3 | 360 | 242.5 |
| Gorai.001G052400 | 208 | 23.987 | 9 | 9.547 | -0.743 | 1,315 | 627 | 43.7 | 7 | 187.9 | 448.7 |
| Gorai.001G052500 | 228 | 26.102 | -10 | 4.75 | -1.043 | 1,108 | 687 | 46.7 | 7 | 158.3 | 397.5 |
| Gorai.001G059600 | 884 | 98.33 | 21.5 | 8.675 | -0.367 | 3,409 | 2,655 | 42.6 | 18 | 189.4 | 282.1 |
| Gorai.001G059700 | 293 | 33.518 | 2.5 | 7.174 | 0.517 | 1,536 | 882 | 43 | 8 | 192 | 630.7 |
| Gorai.001G059800 | 81 | 9.259 | 7 | 9.225 | -0.31 | 409 | 246 | 37.4 | 2 | 204.5 | 424 |
| Gorai.001G068700 | 318 | 35.017 | 9 | 8.745 | -0.58 | 1,600 | 957 | 42.2 | 4 | 400 | 220 |
| Gorai.001G068800 | 92 | 10.911 | 6 | 10.584 | -0.877 | 612 | 279 | 47 | 3 | 204 | 2,292.50 |
| Gorai.001G068900 | 363 | 40.901 | 18.5 | 9.785 | -0.276 | 1,465 | 1,092 | 42 | 6 | 244.2 | 608.6 |
| Gorai.001G069000 | 535 | 55.949 | -7 | 5.074 | 0.203 | 2,340 | 1,608 | 43.7 | 18 | 130 | 393.4 |
| Gorai.001G069100 | 212 | 24.438 | 13 | 10.183 | -0.855 | 1,059 | 639 | 46.9 | 2 | 529.5 | 814 |
| Gorai.001G069200 | 354 | 40.885 | 0 | 6.525 | -0.638 | 1,641 | 1,065 | 41.5 | 6 | 273.5 | 413.4 |
| Gorai.001G069300 | 119 | 13.95 | 6 | 9.617 | -0.168 | 447 | 360 | 45.8 | 1 | 447 | No intron |
| Gorai.001G069400 | 347 | 38.612 | 7.5 | 9.508 | 0.024 | 1,522 | 1,044 | 44.6 | 13 | 117.1 | 598.3 |
| Gorai.001G071900 | 417 | 46.668 | 15.5 | 9.786 | -0.801 | 1,637 | 1,254 | 44.3 | 1 | 1,637.00 | No intron |
| Gorai.001G072000 | 274 | 32.037 | -23 | 4.32 | -0.573 | 1,274 | 825 | 39.8 | 10 | 127.4 | 350 |
| Gorai.001G072100 | 314 | 35.839 | -1.5 | 6.18 | -0.524 | 1,334 | 945 | 44.2 | 3 | 444.7 | 1,121.00 |
| Gorai.001G072200 | 409 | 43.09 | 10 | 8.531 | -0.637 | 2,514 | 1,230 | 47.7 | 1 | 2,514.00 | No intron |
| Gorai.001G075100 | 244 | 27.915 | 15 | 10.353 | -0.271 | 1,137 | 735 | 45.9 | 7 | 162.4 | 435.3 |
| Gorai.001G075200 | 991 | 112.187 | 7 | 7.039 | -0.555 | 3,392 | 2,976 | 41.5 | 14 | 242.3 | 174.1 |
| Gorai.001G075300 | 474 | 51.829 | 5 | 7.658 | 0.214 | 1,943 | 1,425 | 42.5 | 7 | 277.6 | 229.7 |
| Gorai.001G075400 | 222 | 25.815 | 8.5 | 9.418 | 0.058 | 975 | 669 | 42.3 | 5 | 195 | 93.5 |
| Gorai.001G087800 | 67 | 7.511 | -4.5 | 4.33 | -0.412 | 569 | 204 | 38.7 | 1 | 569 | No intron |
| Gorai.001G087900 | 100 | 10.726 | 0 | 6.411 | 0.207 | 303 | 303 | 44.2 | 1 | 303 | No intron |
| Gorai.001G088000 | 101 | 10.703 | 5 | 9.753 | -0.067 | 543 | 306 | 45.8 | 1 | 543 | No intron |
| Gorai.001G088100 | 433 | 50.567 | 19.5 | 9.189 | 0.541 | 1,893 | 1,302 | 39.2 | 4 | 473.3 | 564 |
| Gorai.001G088200 | 159 | 18.074 | 8 | 9.294 | -0.118 | 725 | 480 | 47.5 | 1 | 725 | No intron |
| Gorai.001G088300 | 428 | 46.636 | -1.5 | 5.57 | -0.493 | 1,805 | 1,287 | 42.7 | 14 | 128.9 | 264.5 |
| Gorai.001G089900 | 223 | 25.295 | -4.5 | 5.355 | -0.719 | 1,017 | 672 | 42.3 | 5 | 203.4 | 312.3 |
| Gorai.001G090000 | 239 | 27.299 | 12.5 | 10.444 | -0.669 | 1,378 | 720 | 40.7 | 8 | 172.3 | 583.1 |
| Gorai.001G091900 | 334 | 38.433 | 15 | 8.532 | -0.873 | 1,156 | 1,005 | 45.4 | 3 | 385.3 | 2,128.00 |
| Gorai.001G094700 | 536 | 62.96 | 15 | 7.236 | -0.408 | 2,043 | 1,611 | 38.8 | 2 | 1,021.50 | 77 |
| Gorai.001G094800 | 178 | 20.022 | -0.5 | 6.356 | -0.458 | 537 | 537 | 41.5 | 2 | 268.5 | 1,167.00 |
| Gorai.001G094900 | 271 | 30.821 | 7.5 | 8.546 | -0.283 | 816 | 816 | 37.5 | 3 | 272 | 51.5 |
| Gorai.001G096100 | 118 | 13.473 | 9 | 9.859 | 0.036 | 642 | 357 | 44 | 4 | 160.5 | 259.3 |
| Gorai.001G096200 | 66 | 7.585 | 0 | 6.802 | -0.285 | 487 | 201 | 46.3 | 1 | 487 | No intron |
| Gorai.001G096300 | 313 | 35.62 | -9 | 4.928 | -0.414 | 1,403 | 942 | 49.5 | 2 | 701.5 | 85 |
| Gorai.001G096400 | 313 | 35.62 | -9 | 4.928 | -0.414 | 1,375 | 942 | 49.4 | 2 | 687.5 | 85 |
| Gorai.001G096500 | 213 | 24.429 | 5.5 | 8.286 | -0.291 | 1,358 | 642 | 42.2 | 5 | 271.6 | 407 |
| Gorai.001G096600 | 196 | 22.421 | 2.5 | 8.888 | -0.416 | 591 | 591 | 41.5 | 4 | 147.8 | 298.3 |
| Gorai.001G105700 | 117 | 12.548 | 0 | 6.506 | 0.517 | 460 | 354 | 47.5 | 2 | 230 | 195 |
| Gorai.001G105800 | 510 | 59.274 | 33 | 9.978 | -0.434 | 1,533 | 1,533 | 45 | 3 | 511 | 91 |
| Gorai.001G105900 | 511 | 58.765 | 3 | 7.026 | -0.369 | 2,201 | 1,536 | 43.2 | 2 | 1,100.50 | 338 |
| Gorai.001G106000 | 308 | 34.883 | 0.5 | 6.572 | -0.195 | 927 | 927 | 42.7 | 1 | 927 | No intron |
| Gorai.001G106100 | 82 | 9.174 | 1.5 | 9.034 | 0.635 | 263 | 249 | 39 | 1 | 263 | No intron |
| Gorai.001G106400 | 173 | 19.777 | 8.5 | 9.562 | -0.442 | 1,195 | 522 | 41.8 | 5 | 239 | 618.5 |
| Gorai.001G106500 | 841 | 95.959 | 30 | 9.327 | -0.322 | 2,526 | 2,526 | 42 | 13 | 194.3 | 104.8 |
| Gorai.001G106600 | 246 | 27.868 | 4 | 7.907 | -0.716 | 1,056 | 741 | 46.6 | 3 | 352 | 658.5 |
| Gorai.001G106700 | 154 | 17.6 | 1.5 | 7.787 | -0.599 | 839 | 465 | 52 | 2 | 419.5 | 327 |
| Gorai.001G106800 | 1,061 | 116.413 | -15.5 | 5.266 | 0.008 | 3,879 | 3,186 | 43.6 | 9 | 431 | 271.1 |
| Gorai.001G114400 | 354 | 40.029 | -0.5 | 6.446 | -0.346 | 1,922 | 1,065 | 44.4 | 10 | 192.2 | 165.6 |
| Gorai.001G114500 | 154 | 17.062 | 19 | 10.928 | -0.677 | 990 | 465 | 47.5 | 3 | 330 | 714.5 |
| Gorai.001G114600 | 300 | 32.91 | 8 | 9.07 | 0.27 | 1,467 | 903 | 41.3 | 10 | 146.7 | 351.2 |
| Gorai.001G114700 | 154 | 17.431 | 5 | 8.593 | 0.087 | 1,905 | 465 | 38.1 | 3 | 635 | 449.5 |
| Gorai.001G115100 | 514 | 58.559 | 19 | 9.79 | -0.178 | 1,764 | 1,545 | 46.9 | 1 | 1,764.00 | No intron |
| Gorai.001G116100 | 525 | 57.947 | -2 | 6.217 | -0.668 | 2,530 | 1,578 | 44.9 | 11 | 230 | 334.9 |
| Gorai.001G116200 | 513 | 59.291 | 7.5 | 7.815 | -0.374 | 2,162 | 1,542 | 45.7 | 6 | 360.3 | 340 |
| Gorai.001G120000 | 277 | 31.61 | 9 | 9.326 | -0.239 | 1,527 | 834 | 41.7 | 2 | 763.5 | 1,545.00 |
| Gorai.001G120100 | 316 | 35.545 | 2.5 | 7.198 | -0.115 | 948 | 948 | 41 | 3 | 316 | 114.5 |
| Gorai.001G120200 | 212 | 23.679 | 5.5 | 9.148 | -0.211 | 1,360 | 639 | 47.9 | 2 | 680 | 86 |
| Gorai.001G125900 | 66 | 7.991 | 3 | 8.72 | -0.605 | 201 | 201 | 32.3 | 1 | 201 | No intron |
| Gorai.001G126000 | 485 | 53.938 | -11.5 | 4.996 | -0.703 | 1,919 | 1,458 | 45.7 | 2 | 959.5 | 118 |
| Gorai.001G126100 | 79 | 8.724 | 2.5 | 8.562 | 0.49 | 827 | 240 | 44.2 | 2 | 413.5 | 240 |
| Gorai.001G126200 | 416 | 46.199 | -2.5 | 6.203 | -0.684 | 2,280 | 1,251 | 46.8 | 6 | 380 | 141.6 |
| Gorai.001G126300 | 367 | 41.75 | 16.5 | 8.86 | -0.049 | 1,865 | 1,104 | 39.7 | 6 | 310.8 | 124.2 |
| Gorai.001G126400 | 157 | 16.537 | -2 | 5.47 | 0.331 | 952 | 474 | 41.8 | 3 | 317.3 | 1,072.50 |
| Gorai.001G126500 | 155 | 16.655 | 11.5 | 9.488 | 0.023 | 801 | 468 | 43.4 | 4 | 200.3 | 393 |
| Gorai.001G126600 | 87 | 9.359 | -2 | 4.429 | 0.351 | 264 | 264 | 43.2 | 2 | 132 | 95 |
| Gorai.001G127000 | 183 | 20.437 | 6.5 | 8.22 | -0.622 | 1,294 | 552 | 43.1 | 6 | 215.7 | 926.8 |
| Gorai.001G130800 | 584 | 67.363 | 19.5 | 8.486 | -0.091 | 2,241 | 1,755 | 42.1 | 15 | 149.4 | 135.1 |
| Gorai.001G130900 | 298 | 32.736 | 5.5 | 8.334 | -0.227 | 894 | 894 | 41.5 | 4 | 223.5 | 108.3 |
| Gorai.001G133700 | 971 | 108.892 | 10 | 7.277 | -0.365 | 3,841 | 2,916 | 42.7 | 8 | 480.1 | 386.1 |
| Gorai.001G133800 | 520 | 58.713 | 4 | 6.972 | -0.389 | 2,022 | 1,563 | 49.9 | 4 | 505.5 | 832.3 |
| Gorai.001G135600 | 433 | 46.45 | 4.5 | 7.585 | -0.083 | 1,861 | 1,302 | 45.6 | 9 | 206.8 | 820.6 |
| Gorai.001G135700 | 349 | 37.893 | 2 | 7.059 | 0.661 | 1,853 | 1,050 | 42.1 | 9 | 205.9 | 296.4 |
| Gorai.001G135800 | 97 | 10.928 | -8.5 | 4.196 | -0.477 | 635 | 294 | 47.3 | 2 | 317.5 | 308 |
| Gorai.001G136300 | 1,653 | 183.696 | 40 | 8.235 | -0.446 | 6,102 | 4,962 | 41.2 | 9 | 678 | 712.6 |
| Gorai.001G136400 | 1,618 | 180.575 | 41 | 8.299 | -0.457 | 6,145 | 4,857 | 41 | 10 | 614.5 | 501.1 |
| Gorai.001G136500 | 128 | 14.387 | -2.5 | 5.199 | -0.212 | 387 | 387 | 45 | 1 | 387 | No intron |
| Gorai.001G136600 | 140 | 15.769 | 6 | 10.164 | 0.219 | 457 | 423 | 33.6 | 1 | 457 | No intron |
| Gorai.001G138600 | 119 | 13.752 | -0.5 | 6.258 | -0.318 | 944 | 360 | 35.8 | 2 | 472 | 1,999.00 |
| Gorai.001G138700 | 421 | 49.068 | 16.5 | 8.795 | -0.294 | 1,266 | 1,266 | 33.5 | 8 | 158.3 | 581 |
| Gorai.001G138800 | 333 | 37.006 | -4.5 | 5.221 | -0.626 | 1,591 | 1,002 | 42.5 | 2 | 795.5 | 1,642.00 |
| Gorai.001G143300 | 585 | 65.012 | 24.5 | 8.957 | -0.363 | 2,585 | 1,758 | 48.4 | 3 | 861.7 | 106 |
| Gorai.001G143400 | 88 | 9.978 | 8 | 8.348 | -0.158 | 588 | 267 | 43.8 | 2 | 294 | 270 |
| Gorai.001G148500 | 431 | 48.12 | 1.5 | 6.726 | -0.741 | 1,994 | 1,296 | 45 | 3 | 664.7 | 83 |
| Gorai.001G150200 | 337 | 38.734 | 17 | 9.518 | -0.895 | 2,072 | 1,014 | 45.7 | 4 | 518 | 1,273.30 |
| Gorai.001G153000 | 261 | 28.455 | -1 | 6.294 | -0.023 | 1,644 | 786 | 46.9 | 2 | 822 | 764 |
| Gorai.001G153100 | 482 | 53.222 | 17.5 | 8.889 | 0.187 | 1,904 | 1,449 | 43.3 | 6 | 317.3 | 94 |
| Gorai.001G153200 | 157 | 18.273 | 7 | 9.602 | 0.318 | 1,203 | 474 | 47.3 | 3 | 401 | 1,106.00 |
| Gorai.001G156500 | 105 | 11.717 | -6.5 | 4.363 | -0.052 | 1,040 | 318 | 43.4 | 5 | 208 | 160.5 |
| Gorai.001G156600 | 585 | 66.223 | -1 | 6.387 | -0.209 | 2,262 | 1,758 | 44 | 5 | 452.4 | 594.3 |
| Gorai.001G159400 | 284 | 32.214 | 9.5 | 9.653 | -0.56 | 1,337 | 855 | 36 | 3 | 445.7 | 100.5 |
| Gorai.001G159500 | 268 | 29.24 | -4.5 | 5.123 | -0.516 | 831 | 807 | 50.4 | 5 | 166.2 | 105.5 |
| Gorai.001G168600 | 76 | 8.289 | 2 | 8.51 | -0.163 | 460 | 231 | 50.6 | 2 | 230 | 1,082.00 |
| Gorai.001G168700 | 2,238 | 251.24 | 51.5 | 9.347 | -0.727 | 7,079 | 6,717 | 44.1 | 14 | 505.6 | 312.1 |
| Gorai.001G168800 | 105 | 11.839 | 5.5 | 9.806 | 0.059 | 1,011 | 318 | 45.3 | 5 | 202.2 | 283.5 |
| Gorai.001G170900 | 157 | 17.482 | 4.5 | 9.058 | -1.164 | 1,126 | 474 | 46.2 | 5 | 225.2 | 591.5 |
| Gorai.001G171000 | 790 | 85.7 | -2.5 | 6.276 | -0.226 | 3,085 | 2,373 | 50.2 | 9 | 342.8 | 235.4 |
| Gorai.001G171100 | 67 | 8.191 | 10 | 10.856 | -1.167 | 399 | 204 | 31.4 | 1 | 399 | No intron |
| Gorai.001G171400 | 504 | 55.998 | -10.5 | 5.273 | -0.691 | 1,977 | 1,515 | 42.6 | 11 | 179.7 | 232.8 |
| Gorai.001G171500 | 628 | 71.806 | 16 | 8.36 | -0.32 | 2,424 | 1,887 | 46.1 | 6 | 404 | 333.4 |
| Gorai.001G171600 | 150 | 16.51 | 5 | 8.575 | -0.352 | 1,619 | 453 | 43.5 | 7 | 231.3 | 382.8 |
| Gorai.001G172100 | 106 | 12.213 | 3.5 | 7.814 | -0.422 | 349 | 321 | 39.6 | 3 | 116.3 | 317.5 |
| Gorai.001G172200 | 243 | 28.003 | 4 | 7.533 | -0.608 | 1,132 | 732 | 43.7 | 8 | 141.5 | 622.1 |
| Gorai.001G174100 | 297 | 33.619 | 3.5 | 7.39 | 0.287 | 1,340 | 894 | 44 | 8 | 167.5 | 251.3 |
| Gorai.001G174200 | 171 | 19.061 | 17.5 | 10.553 | -0.394 | 800 | 516 | 49.2 | 5 | 160 | 188.3 |
| Gorai.001G174300 | 437 | 48.549 | 30.5 | 10.51 | -0.249 | 1,921 | 1,314 | 44.7 | 2 | 960.5 | 94 |
| Gorai.001G179300 | 473 | 54.456 | 36 | 10.137 | -0.528 | 2,681 | 1,422 | 41.7 | 15 | 178.7 | 224.8 |
| Gorai.001G179400 | 212 | 23.149 | 2.5 | 8.97 | -0.697 | 2,144 | 639 | 44.3 | 11 | 194.9 | 182.2 |
| Gorai.001G179500 | 66 | 7.266 | 3.5 | 10.495 | 0.462 | 846 | 201 | 43.3 | 1 | 846 | No intron |
| Gorai.001G182900 | 779 | 84.865 | 9.5 | 7.349 | -0.052 | 3,974 | 2,340 | 44.4 | 9 | 441.6 | 560.1 |
| Gorai.001G183500 | 457 | 51.021 | 4.5 | 7.255 | -0.135 | 1,681 | 1,374 | 42 | 5 | 336.2 | 310.5 |
| Gorai.001G183800 | 120 | 10.609 | 0 | 6.482 | 0.502 | 597 | 363 | 54.5 | 1 | 597 | No intron |
| Gorai.001G184100 | 80 | 8.381 | 0 | 6.057 | 0.711 | 440 | 243 | 44 | 2 | 220 | 80 |
| Gorai.001G184200 | 76 | 8.291 | 3 | 8.198 | 0.459 | 280 | 231 | 43.3 | 2 | 140 | 123 |
| Gorai.001G184300 | 764 | 85.368 | 3 | 6.658 | -0.836 | 2,673 | 2,295 | 46.4 | 6 | 445.5 | 100 |
| Gorai.001G185100 | 1,320 | 145.54 | -15 | 5.771 | -0.493 | 4,257 | 3,963 | 41.6 | 11 | 387 | 325.3 |
| Gorai.001G185200 | 465 | 48.76 | 6.5 | 8.008 | -0.002 | 1,849 | 1,398 | 47.4 | 14 | 132.1 | 99.8 |
| Gorai.001G185300 | 87 | 9.628 | -4 | 4.331 | -0.069 | 669 | 264 | 45.1 | 4 | 167.3 | 375 |
| Gorai.001G185400 | 177 | 19.756 | 3 | 8.076 | -0.229 | 577 | 534 | 44 | 1 | 577 | No intron |
| Gorai.001G185900 | 321 | 35.576 | 5.5 | 7.932 | -0.351 | 1,468 | 966 | 43.7 | 4 | 367 | 511.3 |
| Gorai.001G186000 | 219 | 24.544 | 8 | 8.973 | -0.084 | 1,238 | 660 | 45.8 | 5 | 247.6 | 522.3 |
| Gorai.001G186500 | 72 | 8.114 | 2 | 8.249 | 0.489 | 326 | 219 | 43.4 | 3 | 108.7 | 1,404.50 |
| Gorai.001G186600 | 111 | 13.005 | 11.5 | 10.681 | -0.969 | 342 | 336 | 33 | 2 | 171 | 98 |
| Gorai.001G186700 | 206 | 23.625 | 32.5 | 11.088 | -0.451 | 1,042 | 621 | 52.2 | 4 | 260.5 | 306 |
| Gorai.001G186800 | 909 | 100.526 | -4 | 6.213 | -0.049 | 3,286 | 2,730 | 43.3 | 16 | 205.4 | 117.5 |
| Gorai.001G186900 | 677 | 76.213 | -8.5 | 5.665 | -0.385 | 2,325 | 2,034 | 39.7 | 2 | 1,162.50 | 123 |
| Gorai.001G187900 | 215 | 25.562 | 10.5 | 9.549 | 0.388 | 1,238 | 648 | 40.7 | 6 | 206.3 | 836.2 |
| Gorai.001G196800 | 250 | 27.063 | -6.5 | 4.94 | -0.242 | 1,422 | 753 | 46.5 | 2 | 711 | 2,624.00 |
| Gorai.001G202600 | 96 | 10.531 | 3.5 | 8.139 | 0.121 | 291 | 291 | 48.1 | 1 | 291 | No intron |
| Gorai.001G204000 | 220 | 25.121 | -1.5 | 5.465 | 0.018 | 663 | 663 | 46.2 | 2 | 331.5 | 92 |
| Gorai.001G204100 | 85 | 9.553 | 1 | 7.416 | 0.579 | 511 | 258 | 38 | 2 | 255.5 | 662 |
| Gorai.001G204200 | 222 | 25.695 | -4 | 5.171 | 0.607 | 669 | 669 | 41.4 | 6 | 111.5 | 331.2 |
| Gorai.001G204300 | 743 | 80.659 | 1 | 6.692 | -0.153 | 2,730 | 2,232 | 47 | 5 | 546 | 743 |
| Gorai.001G204400 | 143 | 16.251 | 9 | 9.521 | 0.415 | 1,033 | 432 | 46.5 | 3 | 344.3 | 766.5 |
| Gorai.001G206200 | 775 | 86.118 | -29 | 4.713 | -0.822 | 2,357 | 2,328 | 45.1 | 11 | 214.3 | 349.3 |
| Gorai.001G206300 | 512 | 59.288 | 13 | 8.569 | -0.303 | 2,537 | 1,539 | 41.5 | 8 | 317.1 | 231.7 |
| Gorai.001G211700 | 255 | 28.836 | 17.5 | 10.355 | 0.284 | 1,423 | 768 | 37.5 | 5 | 284.6 | 158.8 |
| Gorai.001G211800 | 109 | 11.813 | 2.5 | 9.393 | -0.474 | 943 | 330 | 47 | 4 | 235.8 | 453.7 |
| Gorai.001G211900 | 809 | 90.829 | -0.5 | 6.467 | -0.155 | 2,490 | 2,430 | 41.7 | 2 | 1,245.00 | 64 |
| Gorai.001G212000 | 129 | 13.881 | -4.5 | 4.455 | 0.281 | 493 | 390 | 40.8 | 6 | 82.2 | 181.6 |
| Gorai.001G212200 | 229 | 26.477 | 3 | 7.295 | -0.955 | 690 | 690 | 42 | 2 | 345 | 980 |
| Gorai.001G212300 | 94 | 10.92 | -3 | 4.856 | -0.815 | 285 | 285 | 32.6 | 1 | 285 | No intron |
| Gorai.001G212400 | 522 | 58.915 | 30.5 | 10.179 | -0.615 | 2,166 | 1,569 | 41.7 | 7 | 309.4 | 389.7 |
| Gorai.001G212500 | 297 | 33.178 | -5 | 5.391 | -0.327 | 1,507 | 894 | 47.1 | 3 | 502.3 | 109.5 |
| Gorai.001G212900 | 465 | 52.641 | 4.5 | 7.145 | -0.143 | 1,547 | 1,398 | 45.4 | 2 | 773.5 | 809 |
| Gorai.001G215300 | 307 | 34.318 | 3 | 7.316 | 0.035 | 1,460 | 924 | 44.7 | 9 | 162.2 | 315.6 |
| Gorai.001G215400 | 307 | 34.252 | 3 | 7.55 | 0.073 | 1,471 | 924 | 44.3 | 9 | 163.4 | 325.8 |
| Gorai.001G215500 | 768 | 87.993 | 15 | 8.988 | 0.18 | 3,080 | 2,307 | 41 | 12 | 256.7 | 370.7 |
| Gorai.001G218800 | 398 | 45.281 | 8 | 8.455 | -0.148 | 2,230 | 1,197 | 41.9 | 7 | 318.6 | 277 |
| Gorai.001G218900 | 663 | 74.669 | -12.5 | 5.146 | -0.275 | 2,502 | 1,992 | 41.1 | 15 | 166.8 | 212.5 |
| Gorai.001G219000 | 263 | 27.968 | -2 | 5.91 | 0.034 | 1,223 | 792 | 47.6 | 2 | 611.5 | 174 |
| Gorai.001G221100 | 1,210 | 135.555 | -19.5 | 5.452 | -0.284 | 4,348 | 3,633 | 42 | 8 | 543.5 | 462 |
| Gorai.001G221200 | 159 | 17.94 | 8.5 | 8.766 | -0.186 | 1,064 | 480 | 55 | 1 | 1,064.00 | No intron |
| Gorai.001G222900 | 130 | 14.804 | -2 | 4.974 | -0.232 | 393 | 393 | 36.1 | 1 | 393 | No intron |
| Gorai.001G223700 | 149 | 16.618 | -4 | 4.948 | 0.132 | 1,225 | 450 | 45.3 | 2 | 612.5 | 452 |
| Gorai.001G223800 | 408 | 44.601 | -10 | 4.812 | -0.569 | 2,417 | 1,227 | 44.3 | 5 | 483.4 | 631 |
| Gorai.001G225400 | 284 | 32.18 | -28 | 4.19 | -0.744 | 1,239 | 855 | 40.4 | 4 | 309.8 | 211.3 |
| Gorai.001G225500 | 655 | 69.78 | -37.5 | 4.21 | -0.534 | 2,331 | 1,968 | 45.5 | 6 | 388.5 | 630.2 |
| Gorai.001G225600 | 659 | 74.398 | 3 | 7.008 | 0.432 | 2,655 | 1,980 | 42.5 | 2 | 1,327.50 | 351 |
| Gorai.001G225700 | 55 | 6.621 | 3.5 | 9.791 | -0.153 | 168 | 168 | 44 | 3 | 56 | 1,378.50 |
| Gorai.001G225800 | 259 | 30.851 | -7.5 | 5.054 | -0.731 | 1,291 | 780 | 40.6 | 6 | 215.2 | 288 |
| Gorai.001G225900 | 955 | 106.075 | -3.5 | 6.171 | -1.029 | 3,893 | 2,868 | 46.6 | 8 | 486.6 | 419 |
| Gorai.001G227700 | 196 | 21.58 | 5.5 | 8.775 | 0.324 | 898 | 591 | 50.4 | 2 | 449 | 110 |
| Gorai.001G227800 | 225 | 25.744 | 4 | 8.085 | -0.839 | 1,039 | 678 | 41.4 | 3 | 346.3 | 1,190.00 |
| Gorai.001G227900 | 152 | 17.183 | -17 | 3.992 | -0.468 | 459 | 459 | 39 | 1 | 459 | No intron |
| Gorai.001G228000 | 155 | 17.235 | 5.5 | 8.662 | -0.163 | 694 | 468 | 56.2 | 1 | 694 | No intron |
| Gorai.001G231100 | 368 | 41.887 | 16 | 9.443 | 0.499 | 1,338 | 1,107 | 41.1 | 7 | 191.1 | 550 |
| Gorai.001G231200 | 434 | 47.007 | 12 | 8.603 | -0.823 | 1,882 | 1,305 | 48.7 | 2 | 941 | 577 |
| Gorai.001G233900 | 138 | 15.339 | -3.5 | 5.198 | -0.387 | 1,081 | 417 | 46 | 3 | 360.3 | 692.5 |
| Gorai.001G234000 | 599 | 68.022 | 12 | 7.658 | -0.222 | 2,010 | 1,800 | 41.7 | 4 | 502.5 | 337.3 |
| Gorai.001G234100 | 781 | 85.586 | 8 | 7.079 | -0.434 | 2,389 | 2,346 | 42.9 | 17 | 140.5 | 261.3 |
| Gorai.001G239700 | 253 | 27.615 | 15 | 9.382 | -0.059 | 1,056 | 762 | 45.5 | 3 | 352 | 305.5 |
| Gorai.001G252300 | 363 | 41.434 | -1 | 6.392 | -0.292 | 1,795 | 1,092 | 42.7 | 4 | 448.8 | 91.7 |
| Gorai.001G252400 | 140 | 15.543 | -5 | 4.745 | -0.884 | 423 | 423 | 48 | 1 | 423 | No intron |
| Gorai.001G252500 | 111 | 12.603 | 2.5 | 8.699 | -0.597 | 336 | 336 | 46.1 | 2 | 168 | 52 |
| Gorai.001G252600 | 223 | 25.269 | -14 | 4.365 | -0.735 | 672 | 672 | 45.8 | 1 | 672 | No intron |
| Gorai.001G252700 | 135 | 15.318 | 3.5 | 9.235 | -0.787 | 408 | 408 | 48 | 1 | 408 | No intron |
| Gorai.001G252800 | 189 | 22.088 | 31.5 | 11.571 | -0.784 | 570 | 570 | 46 | 2 | 285 | 45 |
| Gorai.001G254400 | 275 | 29.927 | 4 | 7.327 | -0.646 | 1,309 | 828 | 50.4 | 3 | 436.3 | 553 |
| Gorai.001G254500 | 329 | 37.076 | 6 | 7.543 | -0.21 | 1,413 | 990 | 43.2 | 5 | 282.6 | 191.8 |
| Gorai.001G254600 | 512 | 57.498 | 0.5 | 6.547 | -0.173 | 2,004 | 1,539 | 45.9 | 1 | 2,004.00 | No intron |
| Gorai.001G254700 | 457 | 52.091 | 36.5 | 10.329 | -0.621 | 2,143 | 1,374 | 43.2 | 14 | 153.1 | 216.8 |
| Gorai.001G254800 | 247 | 27.891 | 2.5 | 7.724 | -0.623 | 1,260 | 744 | 43.1 | 10 | 126 | 168.9 |
| Gorai.001G259000 | 234 | 25.934 | 5 | 8.595 | -0.011 | 1,074 | 705 | 43 | 7 | 153.4 | 272.8 |
| Gorai.001G259100 | 148 | 16.789 | 6 | 8.401 | -0.297 | 781 | 447 | 44.5 | 5 | 156.2 | 569.8 |
| Gorai.001G259200 | 187 | 20.811 | 15 | 8.683 | -0.593 | 849 | 564 | 42.7 | 2 | 424.5 | 537 |
| Gorai.001G259300 | 546 | 59.953 | 12.5 | 8.229 | -0.21 | 2,157 | 1,641 | 44.4 | 1 | 2,157.00 | No intron |
| Gorai.001G263200 | 208 | 24.27 | 2 | 7.447 | -0.904 | 774 | 627 | 40.7 | 3 | 258 | 811 |
| Gorai.001G263300 | 205 | 23.58 | 2 | 7.503 | -0.236 | 1,437 | 618 | 44.7 | 6 | 239.5 | 333.8 |
| Gorai.001G263400 | 464 | 49.44 | -5 | 5.414 | -0.194 | 1,632 | 1,395 | 46.2 | 6 | 272 | 290.4 |
| Gorai.001G268500 | 336 | 36.457 | 3.5 | 7.624 | -0.079 | 1,494 | 1,011 | 45.9 | 11 | 135.8 | 160.9 |
| Gorai.001G268600 | 454 | 50.981 | 8.5 | 8.656 | 0.425 | 1,571 | 1,365 | 41.6 | 8 | 196.4 | 236 |
| Gorai.001G268700 | 454 | 50.813 | 10 | 8.21 | 0.447 | 1,368 | 1,365 | 42.7 | 6 | 228 | 84.8 |
| Gorai.001G268800 | 687 | 76.903 | -9.5 | 5.598 | -0.745 | 2,840 | 2,064 | 42.9 | 9 | 315.6 | 167.9 |
| Gorai.001G268900 | 477 | 52.993 | 6 | 7.611 | -0.116 | 1,434 | 1,434 | 40 | 7 | 204.9 | 193.5 |
| Gorai.001G272200 | 203 | 22.383 | 3 | 8.231 | -0.706 | 881 | 612 | 48 | 4 | 220.3 | 414 |
| Gorai.001G272300 | 111 | 12.922 | 0 | 6.537 | -0.233 | 1,111 | 336 | 42 | 2 | 555.5 | 81 |
| Gorai.001G272400 | 444 | 49.375 | -3 | 6.097 | -0.198 | 1,854 | 1,335 | 48.8 | 3 | 618 | 106 |
| Gorai.001G272500 | 70 | 7.884 | -0.5 | 5.592 | 1.139 | 466 | 213 | 41.3 | 1 | 466 | No intron |
| Gorai.002G032100 | 369 | 41.923 | 4 | 7.15 | -0.261 | 1,691 | 1,110 | 43.9 | 6 | 281.8 | 482.8 |
| Gorai.002G032200 | 70 | 8.102 | 4.5 | 8.912 | -0.661 | 848 | 213 | 45.5 | 2 | 424 | 815 |
| Gorai.002G032300 | 515 | 56.878 | -5.5 | 5.835 | 0.497 | 1,917 | 1,548 | 40.6 | 23 | 83.3 | 200.9 |
| Gorai.002G032400 | 658 | 73.838 | 19 | 8.018 | -0.021 | 2,440 | 1,977 | 45 | 1 | 2,440.00 | No intron |
| Gorai.002G032500 | 1,173 | 131.02 | 13 | 7.547 | -0.523 | 3,897 | 3,522 | 42.6 | 18 | 216.5 | 141.6 |
| Gorai.002G032600 | 435 | 48.404 | 11.5 | 8.238 | -0.199 | 1,308 | 1,308 | 50.2 | 1 | 1,308.00 | No intron |
| Gorai.002G038500 | 395 | 43.833 | 22.5 | 9.357 | -0.187 | 1,188 | 1,188 | 38.4 | 4 | 297 | 297.3 |
| Gorai.002G038600 | 109 | 12.305 | 3 | 9.938 | -0.726 | 569 | 330 | 48.5 | 1 | 569 | No intron |
| Gorai.002G038700 | 429 | 47.533 | -16.5 | 4.54 | -0.621 | 2,218 | 1,290 | 44.1 | 9 | 246.4 | 370.1 |
| Gorai.002G038800 | 386 | 42.918 | -5 | 5.357 | -1.056 | 1,570 | 1,161 | 44.6 | 3 | 523.3 | 967.5 |
| Gorai.002G063100 | 659 | 73.482 | -0.5 | 6.474 | -0.831 | 2,665 | 1,980 | 43.9 | 5 | 533 | 278 |
| Gorai.002G063200 | 397 | 45.442 | -3.5 | 5.743 | -0.754 | 1,508 | 1,194 | 42.5 | 6 | 251.3 | 203.6 |
| Gorai.002G063300 | 339 | 35.468 | 6.5 | 8.893 | 0.076 | 1,691 | 1,020 | 47.6 | 7 | 241.6 | 266.8 |
| Gorai.002G063400 | 438 | 44.702 | 5.5 | 8.168 | -0.379 | 1,899 | 1,317 | 51.6 | 2 | 949.5 | 1,680.00 |
| Gorai.002G063500 | 613 | 68.031 | 14 | 9.309 | -0.908 | 2,672 | 1,842 | 42.2 | 5 | 534.4 | 261.3 |
| Gorai.002G063600 | 87 | 9.677 | 2 | 7.836 | -0.138 | 591 | 264 | 46.6 | 1 | 591 | No intron |
| Gorai.002G063700 | 128 | 14.194 | 5.5 | 9.514 | -0.447 | 398 | 387 | 42.1 | 1 | 398 | No intron |
| Gorai.002G064400 | 651 | 71.975 | -9.5 | 5.458 | -0.253 | 2,515 | 1,956 | 43 | 9 | 279.4 | 430.1 |
| Gorai.002G067900 | 724 | 80.788 | 5 | 7.842 | -0.669 | 2,581 | 2,175 | 42 | 20 | 129.1 | 341.3 |
| Gorai.002G068000 | 407 | 45.564 | -6.5 | 5.285 | -0.58 | 2,041 | 1,224 | 46 | 7 | 291.6 | 208.2 |
| Gorai.002G068100 | 231 | 25.772 | 21 | 10.61 | -0.845 | 696 | 696 | 45.5 | 1 | 696 | No intron |
| Gorai.002G068200 | 286 | 31.297 | -9.5 | 4.914 | -0.299 | 1,637 | 861 | 43.2 | 5 | 327.4 | 93.5 |
| Gorai.002G068300 | 182 | 20.261 | 8.5 | 8.693 | -1.014 | 1,344 | 549 | 47.5 | 2 | 672 | 114 |
| Gorai.002G068400 | 178 | 19.34 | -11.5 | 4.369 | -0.799 | 887 | 537 | 49.7 | 2 | 443.5 | 80 |
| Gorai.002G068500 | 1,052 | 115.29 | 5.5 | 6.937 | 0.039 | 4,045 | 3,159 | 43 | 3 | 1,348.30 | 148.5 |
| Gorai.002G082500 | 390 | 43.459 | 16 | 10.046 | 0.093 | 1,173 | 1,173 | 41.7 | 7 | 167.6 | 185.7 |
| Gorai.002G082600 | 864 | 97.084 | 3.5 | 6.804 | -0.218 | 3,091 | 2,595 | 39.6 | 16 | 193.2 | 297.3 |
| Gorai.002G082800 | 330 | 37.929 | 2.5 | 7.449 | -0.465 | 993 | 993 | 40.6 | 3 | 331 | 581 |
| Gorai.002G082900 | 157 | 17.614 | 7 | 10.223 | -0.499 | 474 | 474 | 42.6 | 2 | 237 | 251 |
| Gorai.002G083000 | 461 | 51.739 | 4 | 8.656 | -0.424 | 1,858 | 1,386 | 48 | 1 | 1,858.00 | No intron |
| Gorai.002G084500 | 634 | 70.999 | -14 | 4.845 | -0.26 | 2,221 | 1,905 | 41.3 | 8 | 277.6 | 321 |
| Gorai.002G084600 | 155 | 17.063 | -5.5 | 4.511 | 0.048 | 543 | 468 | 40.8 | 3 | 181 | 280 |
| Gorai.002G084700 | 363 | 40.678 | 14.5 | 9.84 | -0.216 | 1,092 | 1,092 | 42.1 | 4 | 273 | 113.3 |
| Gorai.002G084800 | 570 | 63.465 | -1.5 | 6.306 | -0.109 | 2,061 | 1,713 | 44 | 15 | 137.4 | 262.3 |
| Gorai.002G084900 | 421 | 47.662 | 13.5 | 8.656 | 0.27 | 1,266 | 1,266 | 37.8 | 3 | 422 | 86.5 |
| Gorai.002G088400 | 702 | 79.4 | 13 | 8.75 | -0.909 | 2,843 | 2,109 | 46 | 3 | 947.7 | 700 |
| Gorai.002G088500 | 208 | 23.453 | -7 | 4.714 | -0.301 | 1,165 | 627 | 44.7 | 10 | 116.5 | 258.8 |
| Gorai.002G088600 | 70 | 7.582 | 2 | 8.478 | 0.256 | 652 | 213 | 44.6 | 3 | 217.3 | 267 |
| Gorai.002G091700 | 145 | 16.079 | 14.5 | 8.96 | -0.37 | 438 | 438 | 40.9 | 1 | 438 | No intron |
| Gorai.002G091800 | 141 | 15.524 | 15.5 | 9.196 | -0.427 | 426 | 426 | 41.1 | 1 | 426 | No intron |
| Gorai.002G091900 | 219 | 24.917 | -6.5 | 5.084 | -0.19 | 1,060 | 660 | 40.5 | 3 | 353.3 | 1,081.00 |
| Gorai.002G095700 | 423 | 45.864 | -19 | 4.449 | -0.346 | 1,751 | 1,272 | 45.7 | 3 | 583.7 | 349.5 |
| Gorai.002G095800 | 1,380 | 157.083 | 23.5 | 7.801 | -0.219 | 4,570 | 4,143 | 42.8 | 3 | 1,523.30 | 154 |
| Gorai.002G095900 | 480 | 50.964 | 3.5 | 8.482 | -0.451 | 2,350 | 1,443 | 45.5 | 4 | 587.5 | 211.3 |
| Gorai.002G096000 | 583 | 65.766 | 15 | 8.715 | -0.187 | 1,752 | 1,752 | 41.3 | 2 | 876 | 79 |
| Gorai.002G096100 | 296 | 33.941 | -6 | 5.269 | -0.503 | 1,226 | 891 | 46 | 3 | 408.7 | 213.5 |
| Gorai.002G096200 | 568 | 61.446 | 4.5 | 6.88 | -0.668 | 2,103 | 1,707 | 44.1 | 6 | 350.5 | 445.6 |
| Gorai.002G101700 | 651 | 72.136 | -9.5 | 5.314 | -0.713 | 3,073 | 1,956 | 44 | 4 | 768.3 | 352.3 |
| Gorai.002G101800 | 432 | 47.906 | -0.5 | 6.435 | -0.448 | 1,726 | 1,299 | 44.1 | 5 | 345.2 | 469.3 |
| Gorai.002G101900 | 337 | 38.23 | 10 | 8.346 | -0.427 | 1,597 | 1,014 | 40.3 | 11 | 145.2 | 280 |
| Gorai.002G102800 | 93 | 10.372 | 7.5 | 9.444 | -0.808 | 570 | 282 | 52.5 | 2 | 285 | 382 |
| Gorai.002G102900 | 226 | 25.983 | -12 | 4.536 | -0.189 | 1,456 | 681 | 40.5 | 10 | 145.6 | 184.1 |
| Gorai.002G103000 | 699 | 80.007 | -28.5 | 4.707 | -0.566 | 2,522 | 2,100 | 44 | 3 | 840.7 | 434 |
| Gorai.002G103100 | 210 | 23.646 | 8 | 9.46 | -0.209 | 956 | 633 | 43.9 | 8 | 119.5 | 191.9 |
| Gorai.002G103200 | 233 | 24.984 | 3 | 8.442 | 0.452 | 1,254 | 702 | 44.3 | 5 | 250.8 | 539.3 |
| Gorai.002G103300 | 1,100 | 119.483 | -8.5 | 5.875 | -0.021 | 3,627 | 3,303 | 42.9 | 2 | 1,813.50 | 100 |
| Gorai.002G103400 | 1,180 | 131.802 | 18 | 8.343 | -0.351 | 4,768 | 3,543 | 41.6 | 16 | 298 | 113.8 |
| Gorai.002G104400 | 833 | 92.949 | -2 | 6.288 | -0.176 | 2,562 | 2,502 | 42.8 | 1 | 2,562.00 | No intron |
| Gorai.002G106000 | 141 | 16.095 | 6.5 | 9.588 | -0.193 | 1,404 | 426 | 55.6 | 1 | 1,404.00 | No intron |
| Gorai.002G110600 | 780 | 88.995 | -6 | 6.174 | -0.245 | 2,808 | 2,343 | 40.9 | 15 | 187.2 | 136.9 |
| Gorai.002G122400 | 308 | 34.862 | 0 | 6.508 | -0.635 | 1,612 | 927 | 42.4 | 2 | 806 | 101 |
| Gorai.002G122500 | 735 | 83.071 | 17 | 8.404 | -0.379 | 2,508 | 2,208 | 41.7 | 10 | 250.8 | 129 |
| Gorai.002G122600 | 766 | 86.374 | -14 | 5.248 | -0.139 | 2,611 | 2,301 | 41.5 | 21 | 124.3 | 232 |
| Gorai.002G129200 | 454 | 52.463 | 7.5 | 8.124 | -0.288 | 1,795 | 1,365 | 44.4 | 4 | 448.8 | 825 |
| Gorai.002G132400 | 123 | 13.566 | 5.5 | 10.015 | -0.284 | 733 | 372 | 40.1 | 4 | 183.3 | 225.3 |
| Gorai.002G132500 | 197 | 22.636 | 3.5 | 7.503 | -0.638 | 878 | 594 | 41.8 | 5 | 175.6 | 348.3 |
| Gorai.002G137800 | 551 | 60.466 | 13 | 8.664 | -0.143 | 2,373 | 1,656 | 44.8 | 3 | 791 | 354.5 |
| Gorai.002G137900 | 323 | 37.189 | -5.5 | 5.447 | -0.606 | 1,153 | 972 | 39 | 3 | 384.3 | 109 |
| Gorai.002G141400 | 293 | 32.84 | 3 | 7.665 | -0.504 | 1,424 | 882 | 41.5 | 4 | 356 | 119.7 |
| Gorai.002G146200 | 313 | 34.236 | 17 | 10.248 | 0.809 | 1,514 | 942 | 42.7 | 4 | 378.5 | 738.7 |
| Gorai.002G147700 | 81 | 9.017 | 1 | 7.294 | -0.027 | 246 | 246 | 47.6 | 1 | 246 | No intron |
| Gorai.002G147800 | 772 | 84.671 | -3.5 | 6.29 | -0.139 | 2,967 | 2,319 | 47.5 | 14 | 211.9 | 331.8 |
| Gorai.002G226600 | 1,689 | 184.553 | 2 | 6.607 | -0.124 | 5,681 | 5,070 | 43.2 | 34 | 167.1 | 827.4 |
| Gorai.002G228000 | 368 | 40.562 | 4.5 | 7.178 | -0.05 | 1,459 | 1,107 | 44.7 | 3 | 486.3 | 1,272.50 |
| Gorai.002G228100 | 572 | 62.676 | -14 | 5.143 | -0.207 | 2,400 | 1,719 | 46.1 | 9 | 266.7 | 728 |
| Gorai.002G230500 | 1,003 | 111.749 | 2.5 | 6.669 | -0.294 | 3,906 | 3,012 | 41 | 22 | 177.5 | 383 |
| Gorai.002G230600 | 80 | 8.64 | 2 | 9.729 | -0.074 | 722 | 243 | 45.3 | 3 | 240.7 | 797.5 |
| Gorai.002G230700 | 979 | 106.61 | -6.5 | 5.78 | -0.442 | 3,714 | 2,940 | 43.4 | 25 | 148.6 | 412.9 |
| Gorai.002G231500 | 475 | 53.739 | 12.5 | 9.191 | -1.013 | 2,050 | 1,428 | 42.3 | 2 | 1,025.00 | 99 |
| Gorai.002G231600 | 323 | 35.764 | -3.5 | 5.654 | -0.086 | 1,342 | 972 | 44 | 6 | 223.7 | 999.4 |
| Gorai.002G231700 | 268 | 29.439 | 18 | 10.528 | -0.418 | 1,053 | 807 | 41.8 | 9 | 117 | 148.6 |
| Gorai.002G231800 | 164 | 18.649 | 22 | 10.987 | -0.677 | 921 | 495 | 49.1 | 2 | 460.5 | 629 |
| Gorai.002G231900 | 602 | 66.842 | 5 | 6.895 | -0.23 | 3,001 | 1,809 | 44 | 13 | 230.8 | 342.9 |
| Gorai.002G232900 | 235 | 25.906 | 31.5 | 11.372 | -0.465 | 1,302 | 708 | 41.2 | 2 | 651 | 179 |
| Gorai.002G233000 | 115 | 13.187 | 14 | 10.926 | -0.608 | 779 | 348 | 44.3 | 3 | 259.7 | 679.5 |
| Gorai.002G233100 | 410 | 43.9 | 2 | 7.527 | 0.7 | 1,233 | 1,233 | 42.2 | 4 | 308.3 | 79.3 |
| Gorai.002G233200 | 521 | 59.402 | -12.5 | 4.996 | -0.661 | 2,812 | 1,566 | 43.6 | 4 | 703 | 222.3 |
| Gorai.002G233300 | 621 | 67.938 | 8.5 | 7.496 | -0.251 | 2,360 | 1,866 | 41.3 | 15 | 157.3 | 404.4 |
| Gorai.002G235000 | 76 | 8.977 | 6 | 10.115 | -1.062 | 649 | 231 | 43.7 | 2 | 324.5 | 3,988.00 |
| Gorai.002G235100 | 792 | 89.656 | 11 | 7.727 | -0.443 | 3,003 | 2,379 | 44.2 | 7 | 429 | 691.3 |
| Gorai.002G235200 | 260 | 29.73 | 32.5 | 10.813 | -0.74 | 1,322 | 783 | 42.9 | 10 | 132.2 | 162.2 |
| Gorai.002G235300 | 953 | 103.751 | -8.5 | 5.653 | -0.165 | 3,493 | 2,862 | 42 | 3 | 1,164.30 | 190 |
| Gorai.002G236100 | 215 | 23.718 | -0.5 | 6.362 | -0.016 | 969 | 648 | 45.5 | 7 | 138.4 | 347.3 |
| Gorai.002G241600 | 506 | 58.066 | -32 | 4.481 | -0.846 | 2,076 | 1,521 | 39.8 | 3 | 692 | 617 |
| Gorai.002G256400 | 536 | 61.201 | -8.5 | 5.591 | -0.405 | 2,079 | 1,611 | 42 | 10 | 207.9 | 321.7 |
| Gorai.002G256500 | 146 | 16.101 | -2.5 | 5.635 | -0.803 | 616 | 441 | 41.5 | 1 | 616 | No intron |
| Gorai.002G256600 | 465 | 52.767 | 15.5 | 9.113 | -0.317 | 1,398 | 1,398 | 42.3 | 1 | 1,398.00 | No intron |
| Gorai.002G256700 | 672 | 74.028 | -1 | 6.406 | -0.036 | 2,394 | 2,019 | 41.8 | 1 | 2,394.00 | No intron |
| Gorai.002G256800 | 143 | 15.361 | -2 | 5.089 | 0.118 | 937 | 432 | 43.5 | 3 | 312.3 | 1,054.50 |
| Gorai.002G256900 | 116 | 12.331 | 6.5 | 9.703 | -0.284 | 572 | 351 | 45 | 1 | 572 | No intron |
| Gorai.002G257000 | 294 | 33.308 | 19.5 | 8.904 | 0.362 | 1,015 | 885 | 44 | 8 | 126.9 | 276.9 |
| Gorai.002G257100 | 573 | 63.842 | 13.5 | 8.445 | -0.185 | 2,184 | 1,722 | 43.5 | 6 | 364 | 453.6 |
| Gorai.002G262500 | 1,010 | 114.307 | -11 | 5.706 | 0.055 | 3,905 | 3,033 | 39.7 | 24 | 162.7 | 272.2 |
| Gorai.002G262600 | 226 | 24.885 | -14.5 | 4.5 | -0.742 | 1,159 | 681 | 47.1 | 4 | 289.8 | 512.7 |
| Gorai.002G262700 | 393 | 43.012 | 4.5 | 7.525 | -0.411 | 1,667 | 1,182 | 43.6 | 10 | 166.7 | 342.7 |
| Gorai.002G262800 | 477 | 55.299 | 14 | 8.04 | -0.118 | 1,744 | 1,434 | 41.2 | 8 | 218 | 242.4 |
| Gorai.002G262900 | 259 | 31.418 | 7 | 8.469 | -1.22 | 950 | 780 | 40.5 | 2 | 475 | 88 |
| Gorai.002G263000 | 601 | 68.537 | 7 | 7.346 | -0.235 | 2,803 | 1,806 | 41.3 | 8 | 350.4 | 1,347.30 |
| Gorai.002G263100 | 357 | 40.478 | 3 | 7.04 | -0.341 | 1,569 | 1,074 | 46.2 | 3 | 523 | 272 |
| Gorai.002G263200 | 340 | 37.299 | 9 | 9.79 | -0.628 | 1,662 | 1,023 | 45.4 | 3 | 554 | 816.5 |
| Gorai.003G000500 | 70 | 8.256 | -3 | 4.792 | 0.506 | 213 | 213 | 37.1 | 2 | 106.5 | 904 |
| Gorai.003G000600 | 134 | 15.485 | 17 | 11.251 | -0.562 | 818 | 405 | 44.4 | 4 | 204.5 | 174.3 |
| Gorai.003G000700 | 75 | 8.755 | 3.5 | 8.489 | 0.757 | 942 | 228 | 35.1 | 4 | 235.5 | 947.3 |
| Gorai.003G000800 | 537 | 59.521 | 2.5 | 6.73 | -0.634 | 2,190 | 1,614 | 43.6 | 13 | 168.5 | 307.6 |
| Gorai.003G000900 | 439 | 47.615 | 17.5 | 9.706 | 0.648 | 1,943 | 1,320 | 43.6 | 12 | 161.9 | 342 |
| Gorai.003G001000 | 1,268 | 144.216 | 4.5 | 6.888 | -0.471 | 4,473 | 3,807 | 39.7 | 24 | 186.4 | 204 |
| Gorai.003G001400 | 944 | 105.745 | 21.5 | 9.35 | -0.01 | 3,404 | 2,835 | 44.2 | 8 | 425.5 | 130.6 |
| Gorai.003G001500 | 383 | 41.688 | -3.5 | 5.87 | -0.305 | 2,015 | 1,152 | 44.7 | 4 | 503.8 | 249 |
| Gorai.003G001600 | 424 | 46.175 | 1.5 | 6.962 | 0.846 | 1,275 | 1,275 | 45.7 | 1 | 1,275.00 | No intron |
| Gorai.003G001700 | 307 | 33.496 | 9 | 10.352 | -0.259 | 924 | 924 | 45.5 | 3 | 308 | 480.5 |
| Gorai.003G001800 | 433 | 48.742 | 11.5 | 8.537 | -0.315 | 2,017 | 1,302 | 44.9 | 4 | 504.3 | 405.7 |
| Gorai.003G001900 | 386 | 41.507 | 18.5 | 9.882 | -0.732 | 1,886 | 1,161 | 43.1 | 2 | 943 | 616 |
| Gorai.003G002000 | 1,184 | 133.237 | 3 | 6.669 | -0.472 | 3,939 | 3,555 | 40.7 | 24 | 164.1 | 215.3 |
| Gorai.003G002100 | 179 | 20.038 | 4.5 | 8.223 | 0.577 | 1,487 | 540 | 43.9 | 6 | 247.8 | 356.6 |
| Gorai.003G002200 | 246 | 27.989 | -4 | 5.597 | -0.804 | 1,179 | 741 | 43.3 | 2 | 589.5 | 684 |
| Gorai.003G002300 | 874 | 99.827 | -1 | 6.446 | -0.811 | 3,467 | 2,625 | 41 | 6 | 577.8 | 411.6 |
| Gorai.003G002400 | 1,040 | 115.583 | 6 | 6.94 | -0.226 | 3,702 | 3,123 | 39.1 | 24 | 154.3 | 201.9 |
| Gorai.003G002500 | 321 | 36.948 | 12 | 9.401 | -0.571 | 1,474 | 966 | 44.3 | 5 | 294.8 | 421.8 |
| Gorai.003G002600 | 768 | 86.371 | 14.5 | 8.161 | -0.396 | 2,304 | 2,304 | 42.1 | 7 | 329.1 | 400 |
| Gorai.003G002700 | 596 | 67.628 | 13 | 8.444 | -0.149 | 2,443 | 1,791 | 41.2 | 21 | 116.3 | 234.4 |
| Gorai.003G002800 | 492 | 56.927 | 10.5 | 7.453 | -0.56 | 1,831 | 1,479 | 45.3 | 8 | 228.9 | 217.4 |
| Gorai.003G006600 | 317 | 34.25 | 13 | 9.272 | -0.096 | 1,232 | 954 | 44.1 | 4 | 308 | 91 |
| Gorai.003G006700 | 214 | 23.543 | -1.5 | 6.233 | -0.091 | 1,047 | 645 | 44 | 5 | 209.4 | 628.8 |
| Gorai.003G006800 | 539 | 57.585 | 14 | 10.067 | -0.761 | 2,127 | 1,620 | 48.4 | 6 | 354.5 | 504.2 |
| Gorai.003G006900 | 533 | 56.974 | 13.5 | 9.769 | -0.782 | 2,576 | 1,602 | 47.6 | 7 | 368 | 674.5 |
| Gorai.003G007500 | 266 | 30.311 | 26.5 | 10.785 | -0.699 | 1,637 | 801 | 44.1 | 7 | 233.9 | 459.2 |
| Gorai.003G007600 | 374 | 41.059 | 4 | 7.867 | 0.019 | 1,314 | 1,125 | 39.8 | 5 | 262.8 | 571 |
| Gorai.003G007700 | 88 | 9.879 | 3.5 | 9.066 | -0.212 | 517 | 267 | 38.6 | 3 | 172.3 | 110 |
| Gorai.003G007800 | 155 | 17.571 | 6.5 | 9.11 | -0.491 | 889 | 468 | 51.3 | 2 | 444.5 | 81 |
| Gorai.003G007900 | 325 | 36.883 | 14.5 | 9.359 | -1.285 | 1,697 | 978 | 46 | 3 | 565.7 | 762.5 |
| Gorai.003G008000 | 196 | 21.461 | 6 | 9.101 | -0.215 | 1,368 | 591 | 45.2 | 7 | 195.4 | 425.3 |
| Gorai.003G008100 | 596 | 67.174 | 11.5 | 8.157 | -0.494 | 2,672 | 1,791 | 44.1 | 4 | 668 | 552 |
| Gorai.003G008200 | 302 | 33.87 | 6 | 8.215 | -0.489 | 974 | 909 | 44.8 | 10 | 97.4 | 215.6 |
| Gorai.003G008300 | 249 | 27.146 | 2 | 7.565 | -0.296 | 1,102 | 750 | 48.8 | 3 | 367.3 | 729.5 |
| Gorai.003G008400 | 1,231 | 136.59 | 14 | 7.334 | -0.19 | 3,985 | 3,696 | 42.6 | 13 | 306.5 | 227.6 |
| Gorai.003G008500 | 511 | 55.307 | -1 | 6.364 | -0.357 | 2,167 | 1,536 | 46.8 | 4 | 541.8 | 733.3 |
| Gorai.003G009200 | 291 | 30.4 | -3 | 4.899 | 0.195 | 1,343 | 876 | 45.5 | 3 | 447.7 | 396 |
| Gorai.003G009300 | 101 | 11.386 | 3 | 8.473 | -0.5 | 306 | 306 | 52.3 | 1 | 306 | No intron |
| Gorai.003G009400 | 315 | 35.296 | 6.5 | 9.013 | -1.012 | 1,216 | 948 | 42.4 | 6 | 202.7 | 668.8 |
| Gorai.003G009500 | 541 | 61.274 | 13.5 | 9.137 | -0.553 | 2,166 | 1,626 | 43.7 | 12 | 180.5 | 290.5 |
| Gorai.003G009600 | 425 | 46.881 | -32.5 | 4.272 | -0.69 | 1,873 | 1,278 | 42.4 | 3 | 624.3 | 92.5 |
| Gorai.003G011900 | 854 | 94.746 | 6 | 6.932 | -0.28 | 3,427 | 2,565 | 44.2 | 18 | 190.4 | 134.8 |
| Gorai.003G012000 | 393 | 44.923 | 9 | 8.825 | -0.503 | 1,562 | 1,182 | 40.9 | 2 | 781 | 608 |
| Gorai.003G012100 | 516 | 58.337 | 9.5 | 8.622 | -0.143 | 2,144 | 1,551 | 41.8 | 9 | 238.2 | 649.8 |
| Gorai.003G012200 | 161 | 18.332 | 4.5 | 7.918 | -0.501 | 1,099 | 486 | 43 | 6 | 183.2 | 326.6 |
| Gorai.003G012300 | 909 | 105.375 | 16.5 | 8.171 | -0.303 | 2,730 | 2,730 | 41.5 | 1 | 2,730.00 | No intron |
| Gorai.003G014500 | 225 | 24.29 | -10 | 4.498 | 0.095 | 963 | 678 | 45 | 9 | 107 | 405 |
| Gorai.003G014600 | 88 | 10.058 | 3.5 | 9.398 | -0.818 | 755 | 267 | 39.7 | 4 | 188.8 | 906.7 |
| Gorai.003G014700 | 564 | 61.975 | 11.5 | 7.933 | -0.194 | 1,781 | 1,695 | 45.7 | 16 | 111.3 | 222.9 |
| Gorai.003G014800 | 562 | 61.052 | 3.5 | 6.911 | -0.14 | 2,080 | 1,689 | 46.8 | 16 | 130 | 263.5 |
| Gorai.003G014900 | 167 | 19.108 | -3 | 5.021 | 0.085 | 1,014 | 504 | 40.5 | 7 | 144.9 | 429.7 |
| Gorai.003G015000 | 268 | 29.504 | 1 | 6.734 | -0.066 | 807 | 807 | 47.2 | 1 | 807 | No intron |
| Gorai.003G015100 | 983 | 109.313 | 14.5 | 7.999 | -0.172 | 3,275 | 2,952 | 43 | 12 | 272.9 | 314.9 |
| Gorai.003G016400 | 375 | 39.998 | 0.5 | 6.581 | -0.202 | 1,697 | 1,128 | 46.7 | 6 | 282.8 | 415 |
| Gorai.003G016500 | 533 | 60.615 | 1.5 | 6.657 | -0.238 | 2,238 | 1,602 | 45.5 | 1 | 2,238.00 | No intron |
| Gorai.003G016600 | 218 | 24.947 | -1 | 5.949 | -0.793 | 1,072 | 657 | 46.6 | 3 | 357.3 | 479 |
| Gorai.003G016700 | 264 | 28.107 | 1.5 | 7.059 | -0.213 | 1,272 | 795 | 54.1 | 2 | 636 | 874 |
| Gorai.003G023300 | 170 | 19.391 | -12.5 | 4.401 | -0.859 | 708 | 513 | 38.6 | 7 | 101.1 | 291.3 |
| Gorai.003G023400 | 118 | 13.338 | 3 | 9.488 | 0.181 | 1,356 | 357 | 40.9 | 10 | 135.6 | 285.3 |
| Gorai.003G030900 | 610 | 66.826 | 9.5 | 7.912 | -0.084 | 2,312 | 1,833 | 43.6 | 10 | 231.2 | 760.1 |
| Gorai.003G031000 | 501 | 56.076 | 9 | 7.861 | -0.862 | 2,049 | 1,506 | 48 | 5 | 409.8 | 470.3 |
| Gorai.003G031100 | 180 | 19.138 | 10 | 10.021 | -0.334 | 1,101 | 543 | 46.4 | 3 | 367 | 821 |
| Gorai.003G031200 | 301 | 34.139 | -2 | 6.168 | 0.043 | 1,104 | 906 | 40.2 | 5 | 220.8 | 364 |
| Gorai.003G031300 | 386 | 44.461 | 21.5 | 9.314 | 0.181 | 1,161 | 1,161 | 43.8 | 3 | 387 | 947.5 |
| Gorai.003G038800 | 630 | 70.572 | -9 | 5.617 | -0.287 | 2,759 | 1,893 | 43.7 | 4 | 689.8 | 394.3 |
| Gorai.003G038900 | 1,340 | 150.367 | 38 | 9.407 | -0.07 | 4,231 | 4,023 | 41.7 | 24 | 176.3 | 280.9 |
| Gorai.003G039000 | 370 | 40.9 | -7 | 5.097 | -0.625 | 2,507 | 1,113 | 43.3 | 9 | 278.6 | 772.9 |
| Gorai.003G039100 | 481 | 54.328 | -9 | 5.165 | -0.257 | 1,446 | 1,446 | 41.1 | 5 | 289.2 | 234 |
| Gorai.003G039700 | 79 | 8.97 | -20 | 3.562 | -1.01 | 240 | 240 | 41.2 | 2 | 120 | 321 |
| Gorai.003G047100 | 569 | 64.531 | -3.5 | 6.131 | -0.26 | 2,440 | 1,710 | 42.9 | 16 | 152.5 | 600.8 |
| Gorai.003G047200 | 338 | 36.953 | 7 | 9.174 | 0.047 | 1,401 | 1,017 | 42.1 | 10 | 140.1 | 278.4 |
| Gorai.003G058400 | 352 | 39.298 | 3 | 7.179 | -0.695 | 1,753 | 1,059 | 45.3 | 4 | 438.3 | 668.7 |
| Gorai.003G058500 | 391 | 44.951 | -11 | 4.913 | -1.199 | 1,805 | 1,176 | 46.1 | 2 | 902.5 | 2,031.00 |
| Gorai.003G058600 | 512 | 59.254 | 16.5 | 9.62 | -0.169 | 1,983 | 1,539 | 43.4 | 1 | 1,983.00 | No intron |
| Gorai.003G072300 | 666 | 73.079 | 9 | 8.417 | -0.825 | 3,405 | 2,001 | 48 | 4 | 851.3 | 227.3 |
| Gorai.003G083700 | 676 | 70.009 | 41 | 8.82 | -0.483 | 3,443 | 2,031 | 48.9 | 3 | 1,147.70 | 684 |
| Gorai.003G083800 | 392 | 43.824 | 0 | 6.509 | 0.377 | 1,179 | 1,179 | 42.8 | 6 | 196.5 | 189.4 |
| Gorai.003G083900 | 497 | 53.996 | 9 | 8.585 | -0.171 | 1,911 | 1,494 | 45.9 | 6 | 318.5 | 290.4 |
| Gorai.003G088400 | 240 | 25.702 | 5.5 | 8.819 | 0.366 | 1,562 | 723 | 42.3 | 2 | 781 | 161 |
| Gorai.003G088500 | 96 | 10.843 | 2 | 8.039 | -0.247 | 1,012 | 291 | 36.4 | 3 | 337.3 | 477.5 |
| Gorai.003G090500 | 511 | 58.573 | 12.5 | 8.208 | -0.344 | 2,164 | 1,536 | 41.9 | 3 | 721.3 | 135 |
| Gorai.003G093000 | 51 | 5.754 | 0 | 6.508 | -1.016 | 394 | 156 | 38.5 | 3 | 131.3 | 128 |
| Gorai.003G093100 | 484 | 53.99 | 6 | 7.828 | -0.209 | 1,523 | 1,455 | 40.5 | 10 | 152.3 | 471.3 |
| Gorai.003G098800 | 657 | 70.828 | -18.5 | 4.473 | -0.188 | 1,974 | 1,974 | 44.5 | 7 | 282 | 165.5 |
| Gorai.003G098900 | 167 | 18.924 | 13.5 | 10.154 | -0.441 | 1,207 | 504 | 46.8 | 3 | 402.3 | 133.5 |
| Gorai.003G099000 | 101 | 11.229 | 1.5 | 7.604 | -0.137 | 306 | 306 | 39.5 | 2 | 153 | 118 |
| Gorai.003G109100 | 253 | 29.23 | -1.5 | 6.155 | -0.104 | 1,622 | 762 | 39.6 | 5 | 324.4 | 285 |
| Gorai.003G109200 | 535 | 56.912 | 6.5 | 8.226 | 0.546 | 1,608 | 1,608 | 46.5 | 1 | 1,608.00 | No intron |
| Gorai.003G111000 | 521 | 58.005 | 28 | 9.762 | -0.049 | 2,122 | 1,566 | 45.2 | 8 | 265.3 | 262.7 |
| Gorai.003G111100 | 94 | 10.577 | -5 | 4.336 | -0.08 | 782 | 285 | 37.2 | 8 | 97.8 | 175.4 |
| Gorai.003G111200 | 336 | 37.654 | 10.5 | 9.474 | 0.546 | 1,011 | 1,011 | 38.4 | 1 | 1,011.00 | No intron |
| Gorai.003G111800 | 184 | 19.68 | -0.5 | 6.233 | 0.109 | 704 | 555 | 38.7 | 2 | 352 | 103 |
| Gorai.003G111900 | 203 | 22.563 | 1.5 | 7.899 | -0.258 | 1,069 | 612 | 43.1 | 8 | 133.6 | 267.4 |
| Gorai.003G118900 | 84 | 8.884 | -2 | 4.414 | 0.202 | 864 | 255 | 47.5 | 2 | 432 | 1,680.00 |
| Gorai.003G119000 | 433 | 47.77 | -7.5 | 5.116 | -0.037 | 1,903 | 1,302 | 42.3 | 3 | 634.3 | 492.5 |
| Gorai.003G119100 | 473 | 50.243 | 0 | 6.519 | -0.03 | 2,110 | 1,422 | 45.6 | 3 | 703.3 | 466 |
| Gorai.003G119200 | 118 | 12.949 | 4.5 | 9.252 | -0.084 | 1,315 | 357 | 41.5 | 4 | 328.8 | 713.3 |
| Gorai.003G119300 | 393 | 44.482 | 4 | 6.883 | -0.512 | 1,637 | 1,182 | 42.9 | 12 | 136.4 | 941.4 |
| Gorai.003G126400 | 117 | 12.957 | -6 | 4.595 | -0.613 | 1,103 | 354 | 40.7 | 5 | 220.6 | 173 |
| Gorai.003G126500 | 85 | 9.666 | -1 | 5.068 | -0.526 | 1,092 | 258 | 45.3 | 6 | 182 | 223.2 |
| Gorai.003G126600 | 485 | 54.302 | -4.5 | 5.927 | -0.34 | 2,187 | 1,458 | 45 | 10 | 218.7 | 195.4 |
| Gorai.003G126700 | 914 | 101.024 | 44 | 9.921 | -0.706 | 4,361 | 2,745 | 41.6 | 4 | 1,090.30 | 494 |
| Gorai.003G132600 | 154 | 17.111 | 0.5 | 7.264 | -0.538 | 1,457 | 465 | 42.8 | 3 | 485.7 | 124 |
| Gorai.003G143700 | 354 | 40.184 | -23.5 | 4.398 | -0.589 | 1,897 | 1,065 | 41.5 | 11 | 172.5 | 297.7 |
| Gorai.003G143800 | 537 | 60.754 | 18 | 8.633 | 0.212 | 2,706 | 1,614 | 43 | 12 | 225.5 | 551.1 |
| Gorai.003G154200 | 506 | 54.978 | 1 | 6.643 | -0.045 | 2,430 | 1,521 | 45.8 | 3 | 810 | 467.5 |
| Gorai.003G154300 | 364 | 40.531 | -8.5 | 5.008 | -0.812 | 1,645 | 1,095 | 45.2 | 2 | 822.5 | 526 |
| Gorai.003G154400 | 229 | 25.299 | -2 | 5.578 | 0.275 | 2,073 | 690 | 46.4 | 4 | 518.3 | 159.7 |
| Gorai.003G154500 | 226 | 25.563 | 4 | 8.254 | 0.02 | 681 | 681 | 42.1 | 3 | 227 | 93.5 |
| Gorai.003G154600 | 297 | 32.663 | -2 | 5.99 | -0.469 | 1,301 | 894 | 48.5 | 4 | 325.3 | 2,850.30 |
| Gorai.003G155400 | 324 | 36.58 | -4 | 5.617 | -0.526 | 1,460 | 975 | 39.8 | 3 | 486.7 | 256 |
| Gorai.003G155500 | 165 | 18.711 | 5.5 | 8.477 | 0.416 | 838 | 498 | 50.8 | 6 | 139.7 | 238.2 |
| Gorai.003G155600 | 707 | 81.117 | -24.5 | 4.792 | -0.617 | 2,706 | 2,124 | 42.5 | 4 | 676.5 | 302.7 |
| Gorai.003G155700 | 374 | 42.742 | -1.5 | 6.32 | -0.305 | 1,665 | 1,125 | 41.4 | 6 | 277.5 | 471 |
| Gorai.003G155800 | 490 | 53.781 | -9 | 4.931 | -0.154 | 1,584 | 1,473 | 43.6 | 7 | 226.3 | 118.3 |
| Gorai.003G155900 | 543 | 62.984 | 32 | 9.954 | -0.453 | 2,608 | 1,632 | 46.9 | 3 | 869.3 | 338 |
| Gorai.003G161300 | 320 | 37.629 | -5 | 5.871 | -0.747 | 997 | 963 | 46.2 | 2 | 498.5 | 146 |
| Gorai.003G161400 | 323 | 37.89 | 2 | 6.764 | -0.774 | 972 | 972 | 45.1 | 2 | 486 | 144 |
| Gorai.003G161500 | 328 | 38.043 | 1.5 | 6.794 | -0.44 | 1,037 | 987 | 44.9 | 3 | 345.7 | 85 |
| Gorai.003G161600 | 319 | 37.022 | -1 | 6.358 | -0.514 | 960 | 960 | 44.7 | 2 | 480 | 121 |
| Gorai.003G161700 | 226 | 25.445 | -24.5 | 4.079 | -0.754 | 681 | 681 | 50.2 | 2 | 340.5 | 115 |
| Gorai.003G161800 | 254 | 29.52 | -9 | 4.801 | -0.713 | 765 | 765 | 43.8 | 2 | 382.5 | 232 |
| Gorai.003G161900 | 356 | 40.31 | -17.5 | 4.532 | -0.503 | 1,071 | 1,071 | 45.8 | 2 | 535.5 | 90 |
| Gorai.003G162000 | 489 | 54.187 | 45.5 | 10.68 | -0.848 | 1,919 | 1,470 | 44.7 | 4 | 479.8 | 889.3 |
| Gorai.003G163700 | 783 | 89.748 | -4.5 | 6.137 | -0.221 | 2,759 | 2,352 | 40.3 | 2 | 1,379.50 | 110 |
| Gorai.003G163800 | 299 | 33.597 | -1.5 | 6.292 | -0.639 | 1,891 | 900 | 44.3 | 5 | 378.2 | 540.5 |
| Gorai.003G163900 | 209 | 24.21 | 5 | 9.503 | -0.942 | 1,543 | 630 | 41.3 | 8 | 192.9 | 832.4 |
| Gorai.004G034000 | 666 | 76.096 | -24.5 | 4.765 | -0.561 | 2,001 | 2,001 | 44.7 | 4 | 500.3 | 310.3 |
| Gorai.004G034100 | 772 | 83.026 | 17.5 | 8.72 | -0.126 | 2,319 | 2,319 | 44.2 | 8 | 289.9 | 89 |
| Gorai.004G034200 | 1,354 | 147.27 | -3 | 6.364 | -0.368 | 4,722 | 4,065 | 43.5 | 12 | 393.5 | 184.7 |
| Gorai.004G034300 | 148 | 16.492 | 3 | 8.073 | -0.334 | 939 | 447 | 46.5 | 5 | 187.8 | 429 |
| Gorai.004G034400 | 192 | 20.832 | 4 | 7.671 | 0.529 | 1,948 | 579 | 53.5 | 2 | 974 | 147 |
| Gorai.004G034500 | 1,251 | 138.678 | 8 | 6.842 | -0.697 | 4,565 | 3,756 | 41.4 | 4 | 1,141.30 | 397.3 |
| Gorai.004G035600 | 662 | 73.192 | -34 | 4.473 | -0.461 | 2,464 | 1,989 | 43.3 | 12 | 205.3 | 174.9 |
| Gorai.004G035700 | 363 | 40.727 | 1.5 | 6.794 | -0.375 | 1,684 | 1,092 | 46.5 | 7 | 240.6 | 319 |
| Gorai.004G035800 | 339 | 37.946 | 5 | 8.131 | -0.756 | 1,793 | 1,020 | 44 | 7 | 256.1 | 255.5 |
| Gorai.004G035900 | 431 | 47.276 | 9.5 | 9.308 | -0.932 | 1,715 | 1,296 | 44.7 | 9 | 190.6 | 295.4 |
| Gorai.004G037200 | 386 | 42.376 | 23.5 | 10.327 | NA | 2,599 | 1,161 | 45.4 | 5 | 519.8 | 369.3 |
| Gorai.004G037300 | 318 | 35.751 | -1.5 | 6.237 | -0.687 | 1,699 | 957 | 43.7 | 2 | 849.5 | 170 |
| Gorai.004G037400 | 487 | 52.033 | 15 | 9.884 | 0.714 | 1,828 | 1,464 | 44.1 | 11 | 166.2 | 306.8 |
| Gorai.004G037500 | 710 | 77.266 | 5.5 | 6.878 | -0.455 | 3,002 | 2,133 | 46.2 | 2 | 1,501.00 | 947 |
| Gorai.004G037600 | 390 | 43.001 | 21.5 | 9.011 | -0.218 | 1,471 | 1,173 | 41.9 | 9 | 163.4 | 307.3 |
| Gorai.004G037700 | 857 | 95.863 | 33 | 8.598 | -0.687 | 4,133 | 2,574 | 43 | 15 | 275.5 | 313.7 |
| Gorai.004G046500 | 636 | 69.765 | 30 | 9.933 | -0.35 | 2,588 | 1,911 | 45 | 7 | 369.7 | 418.3 |
| Gorai.004G046600 | 624 | 66.056 | 5.5 | 7.86 | -0.594 | 2,586 | 1,875 | 46.1 | 8 | 323.3 | 711.9 |
| Gorai.004G046700 | 203 | 23.038 | -9 | 4.695 | -1.103 | 1,037 | 612 | 43 | 7 | 148.1 | 547.7 |
| Gorai.004G046800 | 215 | 24.645 | -7 | 5.287 | 0.032 | 648 | 648 | 43.5 | 1 | 648 | No intron |
| Gorai.004G046900 | 176 | 20.223 | -13.5 | 4.314 | -0.018 | 531 | 531 | 44.4 | 3 | 177 | 284.5 |
| Gorai.004G047000 | 232 | 26.614 | -9.5 | 4.988 | 0.016 | 699 | 699 | 43.3 | 1 | 699 | No intron |
| Gorai.004G047100 | 451 | 50.508 | 1.5 | 6.65 | -0.283 | 1,996 | 1,356 | 45.4 | 2 | 998 | 639 |
| Gorai.004G047200 | 982 | 108.789 | -4.5 | 6.239 | -0.392 | 3,640 | 2,949 | 45.2 | 11 | 330.9 | 236.5 |
| Gorai.004G049000 | 334 | 36.177 | 4.5 | 7.674 | -0.083 | 1,198 | 1,005 | 50.4 | 2 | 599 | 86 |
| Gorai.004G049100 | 334 | 35.695 | 10 | 8.548 | -0.013 | 1,225 | 1,005 | 51 | 2 | 612.5 | 86 |
| Gorai.004G049200 | 334 | 35.745 | 9 | 8.546 | -0.018 | 1,005 | 1,005 | 51.7 | 2 | 502.5 | 81 |
| Gorai.004G049300 | 131 | 14.983 | 16 | 10.536 | -0.908 | 393 | 393 | 50.9 | 2 | 196.5 | 30 |
| Gorai.004G049400 | 394 | 43.399 | -15.5 | 4.645 | -0.113 | 1,734 | 1,185 | 45.8 | 3 | 578 | 127.5 |
| Gorai.004G053100 | 1,554 | 170.282 | 37 | 8.579 | -0.828 | 5,614 | 4,665 | 40.8 | 15 | 374.3 | 430.8 |
| Gorai.004G053200 | 377 | 40.934 | 17 | 8.592 | -0.537 | 2,216 | 1,134 | 49.3 | 7 | 316.6 | 569 |
| Gorai.004G053300 | 375 | 42.757 | 15 | 9.411 | -0.349 | 1,128 | 1,128 | 41.2 | 9 | 125.3 | 128.9 |
| Gorai.004G069100 | 75 | 8.225 | 4.5 | 8.505 | 0.461 | 307 | 228 | 46.1 | 2 | 153.5 | 227 |
| Gorai.004G069200 | 449 | 49.906 | 3.5 | 7.087 | 0.119 | 1,663 | 1,350 | 44.1 | 2 | 831.5 | 112 |
| Gorai.004G069300 | 205 | 23.382 | 1.5 | 6.87 | -0.046 | 1,470 | 618 | 40.8 | 2 | 735 | 523 |
| Gorai.004G069400 | 533 | 61.123 | 0 | 6.538 | -0.44 | 2,266 | 1,602 | 41.7 | 3 | 755.3 | 676.5 |
| Gorai.004G069500 | 217 | 25.104 | 12 | 9.372 | -1.132 | 1,207 | 654 | 44.5 | 4 | 301.8 | 820.3 |
| Gorai.004G078000 | 346 | 37.751 | -3 | 5.714 | -0.127 | 1,576 | 1,041 | 45.1 | 7 | 225.1 | 361 |
| Gorai.004G078100 | 574 | 65.873 | 15 | 8.601 | -0.68 | 2,807 | 1,725 | 44.2 | 13 | 215.9 | 137.4 |
| Gorai.004G079600 | 205 | 21.946 | 3.5 | 7.635 | 0.377 | 892 | 618 | 51.1 | 2 | 446 | 547 |
| Gorai.004G079700 | 170 | 19.096 | 7.5 | 8.462 | 0.014 | 672 | 513 | 42.9 | 2 | 336 | 87 |
| Gorai.004G088100 | 1,009 | 110.968 | 20.5 | 9.31 | -0.63 | 3,446 | 3,030 | 45.2 | 13 | 265.1 | 433.3 |
| Gorai.004G088200 | 520 | 57.224 | 17 | 8 | -0.07 | 2,096 | 1,563 | 43.4 | 11 | 190.5 | 206 |
| Gorai.004G088300 | 281 | 32.471 | -3 | 5.711 | -0.427 | 1,161 | 846 | 40.4 | 2 | 580.5 | 39 |
| Gorai.004G117300 | 328 | 35.714 | 0 | 6.663 | 0.046 | 1,184 | 987 | 51.5 | 2 | 592 | 332 |
| Gorai.004G117400 | 252 | 28.225 | 13 | 9.656 | -0.502 | 759 | 759 | 45.7 | 3 | 253 | 73.5 |
| Gorai.004G117500 | 282 | 31.46 | 6.5 | 7.766 | -0.457 | 1,559 | 849 | 43.2 | 5 | 311.8 | 538.3 |
| Gorai.004G124700 | 273 | 29.03 | 12.5 | 9.377 | -0.493 | 1,783 | 822 | 48.8 | 5 | 356.6 | 145.3 |
| Gorai.004G124800 | 382 | 41.813 | 0 | 6.507 | -0.445 | 1,534 | 1,149 | 43 | 12 | 127.8 | 226.5 |
| Gorai.004G126800 | 402 | 44.866 | 9 | 7.663 | -0.322 | 1,552 | 1,209 | 42.3 | 7 | 221.7 | 247.3 |
| Gorai.004G126900 | 298 | 31.13 | 6 | 8.375 | 0.418 | 1,384 | 897 | 54.7 | 6 | 230.7 | 572.2 |
| Gorai.004G128800 | 406 | 44.662 | -3 | 5.858 | -0.132 | 1,659 | 1,221 | 44.4 | 3 | 553 | 85.5 |
| Gorai.004G128900 | 349 | 38.513 | 4 | 7.126 | -0.449 | 1,562 | 1,050 | 46.7 | 12 | 130.2 | 272.4 |
| Gorai.004G129000 | 392 | 42.301 | 1.5 | 6.686 | 0.112 | 1,508 | 1,179 | 48 | 10 | 150.8 | 111.2 |
| Gorai.004G133600 | 377 | 42.027 | -9.5 | 4.623 | 0.16 | 1,134 | 1,134 | 44.9 | 2 | 567 | 1,682.00 |
| Gorai.004G133700 | 628 | 69.544 | 18 | 9.281 | -0.329 | 2,871 | 1,887 | 47.6 | 9 | 319 | 221.3 |
| Gorai.004G133900 | 85 | 9.915 | 11 | 10.726 | -0.515 | 880 | 258 | 43.8 | 3 | 293.3 | 564.5 |
| Gorai.004G134000 | 448 | 48.808 | 0.5 | 6.593 | -0.08 | 1,778 | 1,347 | 47.1 | 5 | 355.6 | 800.5 |
| Gorai.004G134100 | 255 | 29.895 | 3 | 6.708 | -0.893 | 1,091 | 768 | 51 | 2 | 545.5 | 52 |
| Gorai.004G134200 | 100 | 11.782 | 10 | 10.9 | 0.936 | 727 | 303 | 36.3 | 4 | 181.8 | 651.7 |
| Gorai.004G134500 | 46 | 5.173 | -4 | 4.165 | 0.154 | 559 | 141 | 44 | 3 | 186.3 | 791 |
| Gorai.004G134600 | 355 | 40.059 | -4 | 5.444 | -0.727 | 1,650 | 1,068 | 44.5 | 3 | 550 | 91 |
| Gorai.004G134700 | 189 | 20.731 | 10 | 10.036 | 0.087 | 1,121 | 570 | 45.1 | 3 | 373.7 | 478 |
| Gorai.004G135500 | 705 | 80.53 | 21.5 | 9.095 | -0.551 | 2,752 | 2,118 | 44.4 | 4 | 688 | 112.3 |
| Gorai.004G135900 | 148 | 16.575 | 3 | 8.069 | -0.33 | 980 | 447 | 47.7 | 6 | 163.3 | 477.6 |
| Gorai.004G136000 | 298 | 33.92 | -4 | 5.276 | -0.457 | 1,838 | 897 | 45.5 | 7 | 262.6 | 88.5 |
| Gorai.004G136100 | 129 | 14.516 | 3 | 8.702 | -0.133 | 512 | 390 | 42.3 | 3 | 170.7 | 103 |
| Gorai.004G137300 | 921 | 105.064 | 36.5 | 9.705 | -0.218 | 3,074 | 2,766 | 45.3 | 14 | 219.6 | 278.8 |
| Gorai.004G138100 | 1,430 | 162.153 | 19 | 8.316 | 0.073 | 4,547 | 4,293 | 42.4 | 24 | 189.5 | 277.5 |
| Gorai.004G138400 | 180 | 19.83 | 10 | 10.051 | -0.812 | 924 | 543 | 50.1 | 5 | 184.8 | 184 |
| Gorai.004G138500 | 703 | 78.806 | 2 | 6.638 | -0.022 | 2,263 | 2,112 | 40.9 | 1 | 2,263.00 | No intron |
| Gorai.004G138600 | 704 | 81.025 | -24 | 4.794 | -0.591 | 2,576 | 2,115 | 44.2 | 4 | 644 | 194.3 |
| Gorai.004G138700 | 76 | 8.628 | 15 | 10.794 | -0.355 | 228 | 228 | 40.8 | 3 | 76 | 202.5 |
| Gorai.004G138800 | 242 | 27.295 | 14.5 | 10.186 | -0.261 | 729 | 729 | 41.2 | 5 | 145.8 | 135.3 |
| Gorai.004G139700 | 238 | 26.638 | 2.5 | 7.155 | -0.103 | 1,010 | 717 | 46.4 | 9 | 112.2 | 255.5 |
| Gorai.004G139800 | 412 | 46.133 | -9 | 5.12 | -0.25 | 1,771 | 1,239 | 51 | 2 | 885.5 | 1,318.00 |
| Gorai.004G152300 | 186 | 21.151 | -1 | 6.303 | -0.391 | 1,569 | 561 | 49.4 | 4 | 392.3 | 193.7 |
| Gorai.004G153900 | 1,175 | 132.659 | -63.5 | 4.62 | -0.98 | 3,967 | 3,528 | 42.2 | 3 | 1,322.30 | 361.5 |
| Gorai.004G154000 | 143 | 15.967 | 9.5 | 8.807 | -0.57 | 724 | 432 | 47.7 | 1 | 724 | No intron |
| Gorai.004G168900 | 252 | 28.911 | -6 | 4.739 | -0.335 | 1,366 | 759 | 43.5 | 8 | 170.8 | 259.3 |
| Gorai.004G169000 | 251 | 28.776 | 7 | 8.704 | -0.72 | 1,262 | 756 | 44.2 | 8 | 157.8 | 305.4 |
| Gorai.004G169100 | 313 | 34.717 | 2 | 6.751 | 0.005 | 1,172 | 942 | 43.8 | 1 | 1,172.00 | No intron |
| Gorai.004G172700 | 327 | 36.778 | 0 | 6.509 | -0.72 | 1,685 | 984 | 44.2 | 2 | 842.5 | 436 |
| Gorai.004G172800 | 68 | 8.012 | 1.5 | 8.218 | 0.359 | 1,276 | 207 | 50.7 | 2 | 638 | 71 |
| Gorai.004G172900 | 367 | 41.21 | 7.5 | 8.297 | -0.942 | 1,285 | 1,104 | 44.3 | 7 | 183.6 | 89.5 |
| Gorai.004G173100 | 528 | 59.131 | -2 | 6.203 | -0.03 | 2,431 | 1,587 | 42.4 | 1 | 2,431.00 | No intron |
| Gorai.004G173200 | 105 | 12.07 | 10 | 10.391 | 0.263 | 1,013 | 318 | 43.1 | 4 | 253.3 | 841.3 |
| Gorai.004G173300 | 360 | 40.493 | 22 | 8.518 | -0.556 | 1,941 | 1,083 | 41.9 | 8 | 242.6 | 492.4 |
| Gorai.004G184400 | 361 | 40.906 | 12.5 | 8.615 | 0.54 | 1,422 | 1,086 | 42.5 | 2 | 711 | 84 |
| Gorai.004G184500 | 217 | 24.637 | 16.5 | 9.447 | -0.624 | 1,724 | 654 | 44.8 | 4 | 431 | 371.3 |
| Gorai.004G187000 | 73 | 8.303 | -11 | 3.838 | -0.523 | 256 | 219 | 36.5 | 3 | 85.3 | 481.5 |
| Gorai.004G187100 | 402 | 46.205 | 12 | 9.353 | -0.19 | 1,209 | 1,209 | 41.9 | 7 | 172.7 | 174 |
| Gorai.004G187300 | 487 | 55.69 | 12.5 | 9.152 | -0.155 | 1,858 | 1,464 | 42.7 | 8 | 232.3 | 136.4 |
| Gorai.004G187400 | 445 | 49.892 | 1.5 | 6.736 | -0.066 | 1,493 | 1,338 | 43.9 | 1 | 1,493.00 | No intron |
| Gorai.004G192300 | 252 | 27.598 | 0 | 6.487 | -0.135 | 1,070 | 759 | 46.4 | 5 | 214 | 107.5 |
| Gorai.004G192400 | 248 | 27.571 | 10 | 8.847 | -0.128 | 993 | 747 | 46.2 | 4 | 248.3 | 105 |
| Gorai.004G192500 | 289 | 31.871 | 3.5 | 7.538 | -0.019 | 1,106 | 870 | 46 | 4 | 276.5 | 110 |
| Gorai.004G192600 | 428 | 47.477 | 8 | 8.288 | 0.115 | 1,739 | 1,287 | 43.2 | 2 | 869.5 | 84 |
| Gorai.004G200500 | 172 | 19.166 | 8.5 | 9.837 | -0.559 | 694 | 519 | 40.3 | 4 | 173.5 | 293.3 |
| Gorai.004G200800 | 702 | 78.184 | -12 | 5.14 | -0.231 | 2,400 | 2,109 | 45 | 7 | 342.9 | 150.8 |
| Gorai.004G200900 | 283 | 30.252 | 5 | 8.576 | -0.194 | 1,328 | 852 | 43.3 | 7 | 189.7 | 656 |
| Gorai.004G201000 | 371 | 41.078 | -16.5 | 4.568 | -0.84 | 1,116 | 1,116 | 42.8 | 2 | 558 | 90 |
| Gorai.004G201100 | 357 | 41.577 | 0.5 | 6.584 | -0.899 | 1,782 | 1,074 | 43.3 | 4 | 445.5 | 615 |
| Gorai.004G208600 | 381 | 42.823 | -5 | 5.296 | 0.009 | 1,776 | 1,146 | 47.8 | 6 | 296 | 175.6 |
| Gorai.004G208700 | 334 | 38.385 | -20 | 4.391 | -0.457 | 1,666 | 1,005 | 43.3 | 3 | 555.3 | 229 |
| Gorai.004G208800 | 311 | 34.363 | -8.5 | 4.759 | -0.686 | 1,610 | 936 | 51.3 | 3 | 536.7 | 260 |
| Gorai.004G208900 | 194 | 21.999 | -17.5 | 4.292 | -0.844 | 624 | 585 | 40.3 | 3 | 208 | 89.5 |
| Gorai.004G209000 | 548 | 59.417 | 28.5 | 10.308 | -0.143 | 1,892 | 1,647 | 45.2 | 3 | 630.7 | 430.5 |
| Gorai.004G209100 | 213 | 23.399 | 6.5 | 9.059 | 0.345 | 642 | 642 | 44.9 | 3 | 214 | 112.5 |
| Gorai.004G209200 | 354 | 39.608 | 2 | 6.936 | -0.188 | 1,552 | 1,065 | 41.8 | 8 | 194 | 156.7 |
| Gorai.004G209300 | 192 | 22.206 | -6.5 | 4.876 | -1.188 | 743 | 579 | 43.4 | 7 | 106.1 | 261.8 |
| Gorai.004G210200 | 659 | 73.374 | 2.5 | 6.724 | -0.118 | 2,710 | 1,980 | 42.5 | 13 | 208.5 | 158.2 |
| Gorai.004G210300 | 149 | 15.989 | 1.5 | 7.821 | 0.27 | 581 | 450 | 42 | 3 | 193.7 | 4,051.50 |
| Gorai.004G210400 | 274 | 31.324 | 7.5 | 8.024 | -0.143 | 935 | 825 | 35.4 | 4 | 233.8 | 47 |
| Gorai.004G214800 | 613 | 66.91 | 3.5 | 7.023 | 0.044 | 1,995 | 1,842 | 44.8 | 1 | 1,995.00 | No intron |
| Gorai.004G214900 | 582 | 65.455 | 0 | 6.51 | -0.151 | 2,201 | 1,749 | 42.1 | 3 | 733.7 | 97.5 |
| Gorai.004G215000 | 490 | 55.543 | 16.5 | 9.547 | -0.652 | 2,340 | 1,473 | 44.5 | 4 | 585 | 1,443.30 |
| Gorai.004G215100 | 2,487 | 285.362 | 34 | 7.902 | 0.267 | 8,342 | 7,464 | 40.4 | 21 | 397.2 | 337.2 |
| Gorai.004G215200 | 176 | 19.208 | 0 | 6.549 | -0.625 | 626 | 531 | 45.6 | 1 | 626 | No intron |
| Gorai.004G219300 | 320 | 35.92 | 33.5 | 10.58 | -0.881 | 1,815 | 963 | 43.2 | 3 | 605 | 379 |
| Gorai.004G219400 | 323 | 36.241 | 31 | 10.307 | -0.864 | 1,936 | 972 | 42.8 | 3 | 645.3 | 399 |
| Gorai.004G220400 | 340 | 38.522 | -2 | 6.27 | -0.24 | 1,374 | 1,023 | 44.4 | 4 | 343.5 | 108.3 |
| Gorai.004G220500 | 360 | 41.329 | 4.5 | 7.295 | -0.249 | 1,374 | 1,083 | 41.6 | 4 | 343.5 | 102.3 |
| Gorai.004G220600 | 340 | 38.229 | -2 | 6.235 | -0.249 | 1,802 | 1,023 | 42.6 | 4 | 450.5 | 238.3 |
| Gorai.004G220700 | 333 | 37.831 | -1 | 6.388 | -0.196 | 1,281 | 1,002 | 43.5 | 4 | 320.3 | 99 |
| Gorai.004G220800 | 216 | 24.655 | -2.5 | 5.241 | -0.713 | 951 | 651 | 48.1 | 1 | 951 | No intron |
| Gorai.004G220900 | 156 | 17.324 | 3 | 8.926 | -0.887 | 1,111 | 471 | 45.9 | 8 | 138.9 | 180.4 |
| Gorai.004G221000 | 476 | 53.846 | 12 | 9.467 | -0.709 | 2,060 | 1,431 | 41.6 | 14 | 147.1 | 208.6 |
| Gorai.004G223200 | 424 | 47.284 | 14.5 | 9.521 | -0.203 | 1,275 | 1,275 | 43.7 | 3 | 425 | 165.5 |
| Gorai.004G223300 | 1,002 | 111.845 | 2.5 | 6.656 | -0.874 | 3,486 | 3,009 | 43.1 | 6 | 581 | 398.4 |
| Gorai.004G223400 | 295 | 32.695 | -2 | 5.946 | 0.192 | 1,584 | 888 | 43.8 | 6 | 264 | 618.2 |
| Gorai.004G226600 | 261 | 29.006 | 14.5 | 10.069 | -1.387 | 2,043 | 786 | 50.6 | 4 | 510.8 | 256.7 |
| Gorai.004G226700 | 376 | 42.874 | 12 | 9.013 | -0.47 | 1,131 | 1,131 | 45.4 | 7 | 161.6 | 168.5 |
| Gorai.004G226800 | 370 | 42.024 | 0 | 6.514 | -0.502 | 1,532 | 1,113 | 46.1 | 7 | 218.9 | 213.3 |
| Gorai.004G226900 | 289 | 32.035 | 2 | 6.8 | -0.467 | 1,134 | 870 | 51.6 | 4 | 283.5 | 460 |
| Gorai.004G227000 | 481 | 55.019 | -29 | 4.425 | -0.701 | 2,026 | 1,446 | 38.9 | 6 | 337.7 | 182 |
| Gorai.004G227100 | 250 | 27.529 | -3.5 | 5.813 | -0.366 | 1,433 | 753 | 50.9 | 9 | 159.2 | 155.1 |
| Gorai.004G229200 | 599 | 66.678 | -13.5 | 5.17 | -0.277 | 1,997 | 1,800 | 43.2 | 15 | 133.1 | 308.1 |
| Gorai.004G229300 | 421 | 45.653 | 16.5 | 10.004 | -0.427 | 2,052 | 1,266 | 48.5 | 3 | 684 | 773.5 |
| Gorai.004G229400 | 843 | 92.344 | -4.5 | 6.122 | -0.106 | 3,283 | 2,532 | 46 | 19 | 172.8 | 154.4 |
| Gorai.004G229500 | 188 | 19.938 | -3.5 | 4.681 | 0.528 | 978 | 567 | 47.6 | 4 | 244.5 | 786.3 |
| Gorai.004G229800 | 852 | 94.512 | -59.5 | 4.268 | -0.357 | 2,878 | 2,559 | 41.5 | 19 | 151.5 | 192.8 |
| Gorai.004G232400 | 495 | 55.419 | -5 | 5.694 | -0.309 | 1,556 | 1,488 | 46.9 | 1 | 1,556.00 | No intron |
| Gorai.004G232500 | 271 | 30.259 | -13.5 | 4.707 | -0.871 | 1,289 | 816 | 46.4 | 4 | 322.3 | 497.7 |
| Gorai.004G232600 | 755 | 84.126 | 14.5 | 7.887 | 0.424 | 2,652 | 2,268 | 41.4 | 9 | 294.7 | 192.4 |
| Gorai.004G232700 | 325 | 34.617 | 5.5 | 7.607 | 0.682 | 1,650 | 978 | 51.2 | 2 | 825 | 471 |
| Gorai.004G232800 | 378 | 42.992 | 20 | 9.786 | -0.136 | 1,926 | 1,137 | 45.1 | 2 | 963 | 72 |
| Gorai.004G241400 | 859 | 97.075 | -16.5 | 5.083 | -0.279 | 2,580 | 2,580 | 43.3 | 10 | 258 | 155.2 |
| Gorai.004G241500 | 340 | 37.536 | 8.5 | 9.051 | -0.389 | 1,380 | 1,023 | 47.7 | 3 | 460 | 84 |
| Gorai.004G241600 | 644 | 71.792 | -10 | 5.125 | -0.239 | 2,617 | 1,935 | 44.4 | 15 | 174.5 | 263.8 |
| Gorai.004G253100 | 280 | 31.09 | 3.5 | 7.573 | -0.114 | 1,473 | 843 | 45.7 | 9 | 163.7 | 209.4 |
| Gorai.004G253200 | 495 | 54.619 | 12.5 | 8.652 | 0.912 | 2,180 | 1,488 | 40.9 | 7 | 311.4 | 403.5 |
| Gorai.004G253300 | 308 | 33.942 | 7.5 | 8.476 | 0.874 | 927 | 927 | 38.8 | 5 | 185.4 | 369.8 |
| Gorai.004G253400 | 76 | 8.856 | 4 | 9.61 | -1.014 | 911 | 231 | 42.9 | 1 | 911 | No intron |
| Gorai.004G258900 | 1,076 | 124.039 | -16.5 | 5.319 | -0.061 | 4,136 | 3,231 | 40.4 | 32 | 129.3 | 282.4 |
| Gorai.004G259000 | 193 | 21.837 | 1.5 | 7.597 | -0.12 | 1,212 | 582 | 45.2 | 7 | 173.1 | 458 |
| Gorai.004G259100 | 284 | 31.712 | 1.5 | 6.88 | -0.315 | 932 | 855 | 49.7 | 1 | 932 | No intron |
| Gorai.004G259200 | 512 | 58.51 | -20.5 | 4.802 | -0.729 | 2,079 | 1,539 | 41.5 | 10 | 207.9 | 388.4 |
| Gorai.004G259300 | 1,129 | 124.172 | 12.5 | 7.398 | -0.26 | 4,568 | 3,390 | 43.9 | 25 | 182.7 | 159.8 |
| Gorai.004G262100 | 697 | 76.807 | 12.5 | 8.638 | 0.387 | 2,603 | 2,094 | 44.8 | 6 | 433.8 | 902.6 |
| Gorai.004G262200 | 447 | 49.509 | 8.5 | 9.428 | -0.85 | 2,081 | 1,344 | 41 | 12 | 173.4 | 540 |
| Gorai.004G262300 | 473 | 52.895 | 9.5 | 7.791 | -0.177 | 2,015 | 1,422 | 42.5 | 4 | 503.8 | 579.3 |
| Gorai.004G262400 | 380 | 41.134 | 3.5 | 7.13 | -0.004 | 1,516 | 1,143 | 45.1 | 10 | 151.6 | 271.6 |
| Gorai.004G262500 | 447 | 51.545 | 2.5 | 7.129 | -0.22 | 1,886 | 1,344 | 41.6 | 12 | 157.2 | 172.5 |
| Gorai.004G264200 | 2,042 | 231.373 | -37 | 5.416 | -0.612 | 6,899 | 6,129 | 42.7 | 21 | 328.5 | 593 |
| Gorai.004G264300 | 1,060 | 119.782 | 11 | 7.238 | -0.08 | 3,425 | 3,183 | 40.7 | 6 | 570.8 | 650.8 |
| Gorai.004G264400 | 399 | 44.122 | 19.5 | 9.988 | 0.448 | 1,836 | 1,200 | 39.6 | 16 | 114.8 | 527.1 |
| Gorai.004G265400 | 666 | 71.842 | 16 | 9.073 | -0.368 | 2,583 | 2,001 | 44.4 | 15 | 172.2 | 499.1 |
| Gorai.004G265500 | 146 | 15.328 | 7 | 10.109 | -0.191 | 1,028 | 441 | 50.3 | 4 | 257 | 1,177.70 |
| Gorai.004G265600 | 334 | 37.729 | 4.5 | 7.783 | -0.275 | 1,334 | 1,005 | 45.5 | 1 | 1,334.00 | No intron |
| Gorai.004G265700 | 283 | 31.678 | 3 | 7.43 | -0.175 | 1,048 | 852 | 49.2 | 2 | 524 | 831 |
| Gorai.004G265800 | 282 | 32.503 | -11.5 | 4.781 | -0.666 | 1,200 | 849 | 40.5 | 12 | 100 | 360.9 |
| Gorai.004G265900 | 322 | 34.883 | 4.5 | 7.753 | -0.08 | 1,122 | 969 | 45.4 | 5 | 224.4 | 117.5 |
| Gorai.004G266000 | 252 | 27.755 | 13.5 | 10.092 | -0.344 | 1,058 | 759 | 44.8 | 9 | 117.6 | 415.9 |
| Gorai.004G268300 | 301 | 33.642 | 4 | 7.236 | -0.657 | 977 | 906 | 42.7 | 3 | 325.7 | 380 |
| Gorai.004G268400 | 322 | 37.027 | -12 | 4.681 | -0.77 | 1,218 | 969 | 40.9 | 4 | 304.5 | 770 |
| Gorai.004G268500 | 362 | 41.198 | 13.5 | 8.571 | -0.798 | 1,350 | 1,089 | 42.5 | 4 | 337.5 | 170.7 |
| Gorai.004G268600 | 408 | 44.164 | 2.5 | 6.882 | -0.297 | 1,651 | 1,227 | 43.8 | 11 | 150.1 | 674.9 |
| Gorai.004G269300 | 1,301 | 143.698 | 3.5 | 6.662 | -0.031 | 4,169 | 3,906 | 42.2 | 15 | 277.9 | 441.1 |
| Gorai.004G269400 | 318 | 35.637 | -6 | 5.27 | -0.803 | 1,431 | 957 | 43.6 | 3 | 477 | 811 |
| Gorai.004G269500 | 142 | 15.906 | 3 | 8.228 | -0.2 | 1,085 | 429 | 51.3 | 2 | 542.5 | 26 |
| Gorai.005G001200 | 96 | 11.063 | 1 | 7.684 | -0.156 | 435 | 291 | 41.6 | 3 | 145 | 541 |
| Gorai.005G001300 | 756 | 82.816 | -2.5 | 6.343 | -0.287 | 2,426 | 2,271 | 51.3 | 2 | 1,213.00 | 519 |
| Gorai.005G001400 | 418 | 46.5 | 18.5 | 9.527 | -0.273 | 1,378 | 1,257 | 43.4 | 9 | 153.1 | 448.8 |
| Gorai.005G001500 | 425 | 47.162 | 13.5 | 8.858 | -0.346 | 1,883 | 1,278 | 46.1 | 6 | 313.8 | 429 |
| Gorai.005G001600 | 127 | 13.747 | 4.5 | 8.066 | 0.093 | 841 | 384 | 50 | 2 | 420.5 | 66 |
| Gorai.005G001700 | 466 | 51.787 | 5.5 | 7.516 | -0.045 | 2,444 | 1,401 | 43.8 | 9 | 271.6 | 457.6 |
| Gorai.005G001800 | 453 | 50.26 | 9 | 8.966 | -0.846 | 1,637 | 1,362 | 49 | 6 | 272.8 | 189.6 |
| Gorai.005G001900 | 452 | 51.055 | 15 | 9.188 | -0.703 | 1,686 | 1,359 | 39.4 | 8 | 210.8 | 76.1 |
| Gorai.005G002000 | 527 | 58.634 | -29.5 | 4.496 | -0.703 | 2,314 | 1,584 | 44.8 | 2 | 1,157.00 | 598 |
| Gorai.005G002100 | 423 | 45.484 | 11 | 9.419 | 0.706 | 1,917 | 1,272 | 41.6 | 10 | 191.7 | 103.4 |
| Gorai.005G002200 | 739 | 83.307 | 22 | 9.268 | -0.28 | 3,030 | 2,220 | 42.2 | 1 | 3,030.00 | No intron |
| Gorai.005G002300 | 282 | 31.672 | 8 | 9.469 | -0.613 | 1,710 | 849 | 44.3 | 7 | 244.3 | 402 |
| Gorai.005G002400 | 344 | 37.019 | 9 | 9.309 | 0.037 | 1,630 | 1,035 | 46.8 | 2 | 815 | 150 |
| Gorai.005G002500 | 341 | 36.568 | 9 | 9.631 | -0.007 | 1,026 | 1,026 | 45.8 | 1 | 1,026.00 | No intron |
| Gorai.005G002600 | 595 | 62.108 | 6.5 | 7.451 | 0.771 | 2,561 | 1,788 | 44.9 | 6 | 426.8 | 311 |
| Gorai.005G002700 | 959 | 106.328 | 4.5 | 6.906 | -0.135 | 3,343 | 2,880 | 41.7 | 21 | 159.2 | 207.5 |
| Gorai.005G002800 | 607 | 67.587 | -2.5 | 6.106 | -0.165 | 2,348 | 1,824 | 42.5 | 4 | 587 | 127 |
| Gorai.005G002900 | 232 | 26.621 | 4.5 | 9.283 | -0.61 | 1,983 | 699 | 47.5 | 4 | 495.8 | 91.3 |
| Gorai.005G003000 | 261 | 29.74 | -0.5 | 6.281 | -0.148 | 1,224 | 786 | 43.8 | 4 | 306 | 407.7 |
| Gorai.005G003100 | 590 | 63.454 | 40.5 | 11.472 | -0.821 | 2,093 | 1,773 | 46.2 | 5 | 418.6 | 372.5 |
| Gorai.005G003200 | 148 | 16.492 | 3 | 8.069 | -0.272 | 769 | 447 | 47 | 6 | 128.2 | 651.6 |
| Gorai.005G003300 | 148 | 16.492 | 3 | 8.069 | -0.272 | 1,128 | 447 | 47 | 5 | 225.6 | 350 |
| Gorai.005G003400 | 491 | 54.803 | 19.5 | 9.923 | -0.121 | 1,590 | 1,476 | 41.8 | 4 | 397.5 | 370.7 |
| Gorai.005G010900 | 430 | 49.16 | -16.5 | 4.693 | 0.24 | 2,250 | 1,293 | 35.5 | 3 | 750 | 127.5 |
| Gorai.005G011000 | 429 | 48.624 | 0.5 | 6.576 | 0.195 | 1,876 | 1,290 | 37.4 | 3 | 625.3 | 126.5 |
| Gorai.005G011100 | 397 | 45.506 | -6 | 5.824 | -0.28 | 1,680 | 1,194 | 42.9 | 6 | 280 | 542.8 |
| Gorai.005G011200 | 593 | 68.565 | 8.5 | 8.082 | -0.344 | 1,782 | 1,782 | 34.7 | 5 | 356.4 | 203 |
| Gorai.005G011300 | 94 | 10.365 | 6.5 | 10.34 | -1.664 | 651 | 285 | 41.1 | 2 | 325.5 | 547 |
| Gorai.005G011400 | 637 | 72.497 | 6 | 7.275 | -0.432 | 1,920 | 1,914 | 43.2 | 2 | 960 | 507 |
| Gorai.005G016300 | 470 | 53.641 | -24 | 4.644 | -0.628 | 1,989 | 1,413 | 42.6 | 8 | 248.6 | 486.3 |
| Gorai.005G016400 | 269 | 30.365 | 11 | 9.639 | 0.333 | 1,199 | 810 | 43.1 | 5 | 239.8 | 760.8 |
| Gorai.005G016500 | 584 | 63.814 | -5.5 | 5.796 | -0.067 | 1,755 | 1,755 | 46.6 | 3 | 585 | 39 |
| Gorai.005G016600 | 659 | 71.509 | 8.5 | 8.281 | -0.129 | 2,302 | 1,980 | 45.6 | 1 | 2,302.00 | No intron |
| Gorai.005G016700 | 265 | 30.621 | 3.5 | 7.283 | -0.557 | 1,344 | 798 | 40.9 | 3 | 448 | 640.5 |
| Gorai.005G016800 | 354 | 38.798 | -4.5 | 5.621 | -0.013 | 1,629 | 1,065 | 45.5 | 6 | 271.5 | 448.6 |
| Gorai.005G016900 | 154 | 17.278 | -18 | 3.981 | -0.397 | 465 | 465 | 41.9 | 1 | 465 | No intron |
| Gorai.005G021100 | 604 | 67.505 | 26 | 9.787 | -0.244 | 2,259 | 1,815 | 43.1 | 7 | 322.7 | 359.5 |
| Gorai.005G021200 | 683 | 78.201 | 13 | 9.372 | -0.842 | 2,693 | 2,052 | 44.2 | 2 | 1,346.50 | 223 |
| Gorai.005G021300 | 193 | 22.248 | 0 | 6.522 | -0.545 | 890 | 582 | 39.2 | 3 | 296.7 | 722.5 |
| Gorai.005G021400 | 386 | 42.604 | 7 | 8.58 | 0.401 | 1,514 | 1,161 | 41.1 | 1 | 1,514.00 | No intron |
| Gorai.005G025000 | 199 | 23.129 | 8 | 9.308 | -0.552 | 1,204 | 600 | 42.2 | 4 | 301 | 176.3 |
| Gorai.005G025100 | 720 | 79.075 | -18.5 | 4.948 | -0.615 | 2,597 | 2,163 | 41 | 7 | 371 | 102 |
| Gorai.005G025200 | 120 | 14.095 | 9.5 | 10.025 | -0.952 | 363 | 363 | 42.4 | 1 | 363 | No intron |
| Gorai.005G025300 | 168 | 18.628 | 6 | 9.614 | -0.411 | 878 | 507 | 41 | 3 | 292.7 | 478.5 |
| Gorai.005G025400 | 469 | 52.66 | -9 | 5.153 | -0.233 | 2,432 | 1,410 | 46.2 | 1 | 2,432.00 | No intron |
| Gorai.005G047100 | 750 | 85.698 | 9 | 7.505 | 0.045 | 2,460 | 2,253 | 41.3 | 8 | 307.5 | 252.7 |
| Gorai.005G047200 | 330 | 37.248 | -5.5 | 5.327 | -0.956 | 1,702 | 993 | 46.3 | 2 | 851 | 327 |
| Gorai.005G050100 | 159 | 17.464 | 3.5 | 9.627 | -1.002 | 1,020 | 480 | 45.2 | 6 | 170 | 282.4 |
| Gorai.005G050200 | 332 | 35.511 | 0 | 6.511 | 0.07 | 1,403 | 999 | 46.5 | 8 | 175.4 | 220 |
| Gorai.005G050300 | 543 | 63.086 | -8 | 5.553 | -1.179 | 2,089 | 1,632 | 44.5 | 9 | 232.1 | 257.8 |
| Gorai.005G050400 | 217 | 23.778 | 17.5 | 10.803 | -0.727 | 1,045 | 654 | 56.3 | 2 | 522.5 | 40 |
| Gorai.005G050500 | 616 | 69.683 | 1 | 6.608 | -0.384 | 2,977 | 1,851 | 43.3 | 8 | 372.1 | 351.7 |
| Gorai.005G053300 | 140 | 15.013 | 17 | 11.086 | -0.195 | 816 | 423 | 48.2 | 4 | 204 | 518.7 |
| Gorai.005G053400 | 626 | 68.5 | 19 | 8.563 | -0.424 | 2,617 | 1,881 | 44.2 | 4 | 654.3 | 419 |
| Gorai.005G053500 | 421 | 46.338 | -16 | 4.525 | -0.469 | 1,382 | 1,266 | 45.1 | 4 | 345.5 | 321.7 |
| Gorai.005G053600 | 519 | 56.512 | 2 | 6.716 | -0.319 | 2,083 | 1,560 | 44.5 | 18 | 115.7 | 213.2 |
| Gorai.005G053700 | 382 | 43.061 | 6 | 7.744 | -0.674 | 2,196 | 1,149 | 40.9 | 8 | 274.5 | 269.7 |
| Gorai.005G056900 | 932 | 105.623 | 3.5 | 6.817 | -0.802 | 3,471 | 2,799 | 44.1 | 2 | 1,735.50 | 868 |
| Gorai.005G057000 | 1,368 | 152.441 | 11 | 7.188 | -0.25 | 4,510 | 4,107 | 41.4 | 11 | 410 | 211 |
| Gorai.005G057100 | 535 | 59.093 | 11 | 8.663 | 0.558 | 2,660 | 1,608 | 42.2 | 16 | 166.3 | 232 |
| Gorai.005G057200 | 384 | 42.875 | 20.5 | 9.392 | -0.305 | 1,452 | 1,155 | 44 | 5 | 290.4 | 160.3 |
| Gorai.005G057300 | 536 | 58.795 | 19.5 | 9.443 | -0.29 | 1,965 | 1,611 | 46.4 | 3 | 655 | 516 |
| Gorai.005G065700 | 910 | 102.757 | 15.5 | 7.767 | -0.424 | 3,081 | 2,733 | 41.5 | 13 | 237 | 169.3 |
| Gorai.005G065800 | 193 | 22.075 | 0 | 6.525 | -0.132 | 1,005 | 582 | 44.5 | 3 | 335 | 529.5 |
| Gorai.005G065900 | 116 | 13.664 | 17 | 12.015 | -0.319 | 516 | 351 | 33 | 1 | 516 | No intron |
| Gorai.005G066000 | 61 | 7.129 | 14.5 | 11.252 | -1.982 | 567 | 186 | 48.9 | 3 | 189 | 106 |
| Gorai.005G066100 | 878 | 99.829 | -24 | 4.972 | -0.376 | 3,864 | 2,637 | 40.2 | 24 | 161 | 277.9 |
| Gorai.005G066900 | 399 | 45.548 | 3 | 6.824 | -0.28 | 1,261 | 1,200 | 40.3 | 3 | 420.3 | 285.5 |
| Gorai.005G067000 | 498 | 52.685 | 5.5 | 8.137 | 0.629 | 1,497 | 1,497 | 47.8 | 4 | 374.3 | 224.7 |
| Gorai.005G067100 | 848 | 93.677 | 11 | 7.468 | -0.217 | 2,547 | 2,547 | 44.4 | 1 | 2,547.00 | No intron |
| Gorai.005G097800 | 451 | 49.646 | -19 | 4.604 | -0.188 | 1,775 | 1,356 | 48 | 3 | 591.7 | 294.5 |
| Gorai.005G101700 | 692 | 75.305 | 6 | 6.881 | -0.573 | 2,902 | 2,079 | 44.3 | 6 | 483.7 | 336.8 |
| Gorai.005G106800 | 112 | 13.237 | 12.5 | 10.754 | -0.78 | 773 | 339 | 44.8 | 4 | 193.3 | 961.7 |
| Gorai.005G106900 | 119 | 13.62 | 10.5 | 9.778 | 0.356 | 1,010 | 360 | 35.8 | 2 | 505 | 68 |
| Gorai.005G107000 | 313 | 34.309 | 9.5 | 9.011 | -0.72 | 1,650 | 942 | 46.8 | 2 | 825 | 289 |
| Gorai.005G109800 | 435 | 48.178 | 22 | 9.157 | -0.652 | 2,185 | 1,308 | 43.7 | 5 | 437 | 423 |
| Gorai.005G109900 | 398 | 44.593 | 5.5 | 8.416 | -0.387 | 1,839 | 1,197 | 44.4 | 10 | 183.9 | 183.4 |
| Gorai.005G110000 | 115 | 12.783 | 2 | 8.066 | 0.2 | 814 | 348 | 46 | 3 | 271.3 | 239.5 |
| Gorai.005G110100 | 239 | 26.584 | -2 | 5.311 | -0.503 | 1,514 | 720 | 47.2 | 2 | 757 | 90 |
| Gorai.005G112100 | 980 | 107.99 | -0.5 | 6.472 | 0.048 | 3,533 | 2,943 | 44.6 | 2 | 1,766.50 | 89 |
| Gorai.005G114400 | 71 | 8.301 | 2 | 7.512 | 0.166 | 961 | 216 | 44.9 | 3 | 320.3 | 556.5 |
| Gorai.005G114500 | 207 | 23.063 | 9 | 9.881 | -1.02 | 909 | 624 | 46 | 8 | 113.6 | 114.4 |
| Gorai.005G144600 | 542 | 60.718 | -50 | 4.166 | -0.741 | 2,317 | 1,629 | 42 | 3 | 772.3 | 555 |
| Gorai.005G144700 | 480 | 52.832 | -0.5 | 6.407 | -0.048 | 2,276 | 1,443 | 47.1 | 2 | 1,138.00 | 527 |
| Gorai.005G150700 | 823 | 90.576 | -24 | 4.735 | -0.606 | 2,846 | 2,472 | 47 | 5 | 569.2 | 269.8 |
| Gorai.005G150800 | 196 | 22.068 | 9.5 | 8.835 | -0.549 | 935 | 591 | 43.8 | 6 | 155.8 | 96 |
| Gorai.005G150900 | 95 | 11.115 | 4 | 8.48 | 0.012 | 473 | 288 | 41.7 | 2 | 236.5 | 367 |
| Gorai.005G163000 | 140 | 16.352 | 6.5 | 9.034 | -0.176 | 922 | 423 | 43.7 | 1 | 922 | No intron |
| Gorai.005G174300 | 258 | 29.644 | 18 | 10.417 | -0.274 | 1,368 | 777 | 52.4 | 1 | 1,368.00 | No intron |
| Gorai.005G185500 | 164 | 18.504 | 9 | 9.929 | -0.68 | 1,016 | 495 | 47.7 | 3 | 338.7 | 223.5 |
| Gorai.005G185600 | 328 | 36.805 | -7.5 | 4.721 | -0.131 | 2,335 | 987 | 45.7 | 6 | 389.2 | 176.8 |
| Gorai.005G190500 | 191 | 20.711 | 8.5 | 9.299 | 0.473 | 1,049 | 576 | 44.3 | 4 | 262.3 | 645.7 |
| Gorai.005G190600 | 179 | 19.391 | 3 | 7.921 | -0.404 | 1,922 | 540 | 50.7 | 4 | 480.5 | 333.7 |
| Gorai.005G193500 | 717 | 78.583 | -1 | 6.384 | -0.142 | 3,097 | 2,154 | 42.7 | 2 | 1,548.50 | 98 |
| Gorai.005G193600 | 82 | 8.823 | 5.5 | 9.83 | 0.072 | 852 | 249 | 38.6 | 1 | 852 | No intron |
| Gorai.005G193700 | 309 | 34.156 | 5 | 7.074 | -0.624 | 1,408 | 930 | 50.2 | 3 | 469.3 | 489 |
| Gorai.005G193800 | 72 | 8.278 | 3.5 | 8.534 | 0.085 | 1,086 | 219 | 40.2 | 1 | 1,086.00 | No intron |
| Gorai.005G214400 | 895 | 97.821 | 17 | 8.108 | -0.278 | 2,688 | 2,688 | 43 | 18 | 149.3 | 318.5 |
| Gorai.005G214500 | 323 | 36.535 | -20.5 | 4.131 | -0.495 | 1,627 | 972 | 42.8 | 6 | 271.2 | 327.2 |
| Gorai.005G214600 | 316 | 35.966 | 1.5 | 7.047 | -0.534 | 1,364 | 951 | 44.1 | 9 | 151.6 | 173.5 |
| Gorai.005G214700 | 258 | 27.249 | 2 | 7.096 | 0.058 | 1,479 | 777 | 46.8 | 3 | 493 | 552.5 |
| Gorai.005G214800 | 360 | 38.492 | -24 | 4.23 | -0.507 | 1,764 | 1,083 | 45.9 | 9 | 196 | 311.5 |
| Gorai.005G214900 | 347 | 37.601 | 6 | 8.376 | 0.554 | 1,473 | 1,044 | 43.2 | 5 | 294.6 | 409.8 |
| Gorai.005G215000 | 485 | 54.918 | -18 | 4.899 | -0.456 | 2,095 | 1,458 | 44.2 | 8 | 261.9 | 342.4 |
| Gorai.005G216200 | 451 | 50.425 | -10.5 | 4.915 | -0.268 | 1,760 | 1,356 | 46.1 | 5 | 352 | 303.5 |
| Gorai.005G216300 | 117 | 13.75 | -2.5 | 5.179 | -1.019 | 2,194 | 354 | 49.4 | 3 | 731.3 | 918 |
| Gorai.005G216400 | 160 | 18.333 | 11.5 | 10.332 | -0.783 | 483 | 483 | 46.8 | 1 | 483 | No intron |
| Gorai.005G216500 | 568 | 63.6 | -6.5 | 5.919 | -0.407 | 2,558 | 1,707 | 42.2 | 9 | 284.2 | 244.9 |
| Gorai.005G217300 | 474 | 53.186 | 26 | 9.966 | -0.404 | 1,851 | 1,425 | 42.7 | 7 | 264.4 | 245.8 |
| Gorai.005G217400 | 195 | 22.445 | -6.5 | 4.73 | -0.783 | 1,350 | 588 | 44.9 | 3 | 450 | 393 |
| Gorai.005G217500 | 148 | 16.497 | -0.5 | 6.256 | 0.011 | 447 | 447 | 42.1 | 2 | 223.5 | 347 |
| Gorai.005G217600 | 672 | 75.132 | 5 | 6.849 | -0.18 | 2,308 | 2,019 | 44.4 | 1 | 2,308.00 | No intron |
| Gorai.005G217700 | 764 | 85.613 | 5 | 6.898 | -0.296 | 2,762 | 2,295 | 43.4 | 17 | 162.5 | 227.6 |
| Gorai.005G226700 | 395 | 44.436 | 20 | 9.571 | -0.377 | 1,571 | 1,188 | 40.3 | 5 | 314.2 | 337.5 |
| Gorai.005G226800 | 538 | 57.672 | 9 | 8.618 | -0.028 | 2,319 | 1,617 | 43.3 | 19 | 122.1 | 225.1 |
| Gorai.005G226900 | 708 | 77.69 | 10.5 | 8.129 | -0.205 | 2,728 | 2,127 | 42.7 | 20 | 136.4 | 351 |
| Gorai.005G232300 | 275 | 29.36 | 6 | 8.007 | -0.796 | 1,287 | 828 | 50.7 | 1 | 1,287.00 | No intron |
| Gorai.005G232400 | 680 | 76.394 | 8.5 | 7.907 | -0.291 | 2,161 | 2,043 | 42.7 | 5 | 432.2 | 236.3 |
| Gorai.005G232500 | 216 | 22.595 | -4.5 | 4.678 | -0.062 | 1,058 | 651 | 50.5 | 2 | 529 | 88 |
| Gorai.005G232600 | 578 | 62.781 | 40 | 11.043 | -0.389 | 2,646 | 1,737 | 47.3 | 6 | 441 | 241.2 |
| Gorai.005G233700 | 810 | 90.292 | -4.5 | 6.21 | -0.269 | 3,081 | 2,433 | 42.3 | 10 | 308.1 | 771.1 |
| Gorai.005G233800 | 90 | 9.972 | 11.5 | 8.901 | -0.04 | 593 | 273 | 49.1 | 2 | 296.5 | 100 |
| Gorai.005G233900 | 210 | 24.119 | -4 | 5.439 | -0.589 | 1,032 | 633 | 47.1 | 5 | 206.4 | 98.3 |
| Gorai.005G234000 | 525 | 60.541 | 21.5 | 9.149 | -0.378 | 1,578 | 1,578 | 41.2 | 5 | 315.6 | 387 |
| Gorai.005G234100 | 393 | 44.201 | -7.5 | 5.2 | -0.37 | 1,683 | 1,182 | 41.2 | 6 | 280.5 | 475 |
| Gorai.005G234200 | 598 | 67.855 | 2.5 | 6.963 | -0.325 | 2,221 | 1,797 | 48.9 | 3 | 740.3 | 95 |
| Gorai.005G237800 | 474 | 54.264 | 20 | 9.834 | -0.212 | 1,770 | 1,425 | 42.3 | 9 | 196.7 | 290.4 |
| Gorai.005G237900 | 79 | 9.097 | 2.5 | 8.066 | -0.043 | 240 | 240 | 42.9 | 1 | 240 | No intron |
| Gorai.005G238000 | 292 | 31.439 | -4.5 | 4.839 | -0.557 | 1,569 | 879 | 48 | 2 | 784.5 | 101 |
| Gorai.005G238100 | 633 | 70.238 | -12 | 5.211 | -0.581 | 2,196 | 1,902 | 44.4 | 2 | 1,098.00 | 88 |
| Gorai.005G238200 | 131 | 14.884 | 0.5 | 6.67 | 0.419 | 1,165 | 396 | 38.9 | 5 | 233 | 128.5 |
| Gorai.005G238300 | 504 | 56.101 | -2 | 6.113 | -0.17 | 1,515 | 1,515 | 43.9 | 8 | 189.4 | 504.7 |
| Gorai.005G238400 | 123 | 13.994 | 3.5 | 9.403 | -0.747 | 739 | 372 | 45.7 | 2 | 369.5 | 63 |
| Gorai.005G238500 | 2,019 | 222.189 | -33.5 | 5.415 | -0.195 | 6,458 | 6,060 | 41.1 | 14 | 461.3 | 322.4 |
| Gorai.005G239100 | 320 | 35.86 | -7 | 5.107 | -0.329 | 963 | 963 | 51.3 | 1 | 963 | No intron |
| Gorai.005G239200 | 314 | 35.203 | -4.5 | 5.613 | -0.326 | 945 | 945 | 50.5 | 1 | 945 | No intron |
| Gorai.005G239300 | 707 | 79.014 | 8.5 | 7.922 | -0.101 | 2,680 | 2,124 | 43.5 | 13 | 206.2 | 181.5 |
| Gorai.005G239400 | 306 | 34.091 | -3 | 5.682 | -0.135 | 1,467 | 921 | 50.5 | 1 | 1,467.00 | No intron |
| Gorai.005G239500 | 348 | 38.855 | 10 | 9.119 | 0.233 | 1,167 | 1,047 | 44.4 | 2 | 583.5 | 669 |
| Gorai.005G239600 | 57 | 6.174 | 0 | 6.499 | 0.946 | 845 | 174 | 50 | 1 | 845 | No intron |
| Gorai.005G240900 | 349 | 40.441 | 12.5 | 9.233 | -0.497 | 1,721 | 1,050 | 42.4 | 5 | 344.2 | 397.3 |
| Gorai.005G241000 | 121 | 13.354 | -0.5 | 5.884 | -0.087 | 366 | 366 | 47.3 | 1 | 366 | No intron |
| Gorai.005G241100 | 120 | 13.378 | -2.5 | 4.837 | 0.048 | 363 | 363 | 44.6 | 1 | 363 | No intron |
| Gorai.005G241200 | 128 | 14.784 | 8.5 | 10.106 | -0.347 | 1,512 | 387 | 40.6 | 2 | 756 | 312 |
| Gorai.005G241300 | 119 | 13.839 | 10 | 10.393 | -0.231 | 360 | 360 | 40 | 2 | 180 | 42 |
| Gorai.005G241400 | 334 | 38.195 | 0 | 6.526 | -0.328 | 1,713 | 1,005 | 43.8 | 7 | 244.7 | 246.5 |
| Gorai.005G241500 | 108 | 12.505 | 16.5 | 12.154 | -0.407 | 988 | 327 | 48 | 3 | 329.3 | 557 |
| Gorai.005G241600 | 370 | 41.429 | 8 | 9.237 | -0.939 | 1,826 | 1,113 | 42.3 | 5 | 365.2 | 800.8 |
| Gorai.005G242500 | 656 | 74.451 | 30.5 | 9.337 | -0.277 | 3,158 | 1,971 | 39.2 | 10 | 315.8 | 333.9 |
| Gorai.005G242600 | 1,027 | 111.099 | -38.5 | 4.68 | -0.716 | 3,555 | 3,084 | 45 | 8 | 444.4 | 335.9 |
| Gorai.005G242700 | 283 | 32.588 | 2.5 | 7.212 | -0.018 | 852 | 852 | 37.3 | 1 | 852 | No intron |
| Gorai.005G242800 | 207 | 23.055 | -4 | 4.794 | -0.356 | 1,273 | 624 | 45.5 | 7 | 181.9 | 237.3 |
| Gorai.005G242900 | 887 | 98.46 | -21 | 4.902 | -0.096 | 3,230 | 2,664 | 44.1 | 18 | 179.4 | 162 |
| Gorai.005G243000 | 377 | 41.054 | 5.5 | 7.282 | -0.263 | 1,796 | 1,134 | 45.8 | 6 | 299.3 | 467 |
| Gorai.005G245300 | 478 | 54.566 | 16.5 | 9.374 | -0.364 | 1,437 | 1,437 | 41.5 | 3 | 479 | 900 |
| Gorai.005G245400 | 531 | 59.788 | -1 | 6.376 | -0.192 | 2,633 | 1,596 | 43.1 | 13 | 202.5 | 210.6 |
| Gorai.005G245500 | 578 | 65.647 | 10 | 8.35 | -0.523 | 2,012 | 1,737 | 43.4 | 12 | 167.7 | 90.8 |
| Gorai.005G245600 | 663 | 75.45 | 19.5 | 8.966 | -0.39 | 1,992 | 1,992 | 41.9 | 14 | 142.3 | 217.2 |
| Gorai.005G245700 | 202 | 22.592 | 0 | 6.509 | -0.35 | 609 | 609 | 40.2 | 2 | 304.5 | 161 |
| Gorai.005G245800 | 83 | 9.361 | 0 | 6.525 | -0.065 | 835 | 252 | 40.1 | 3 | 278.3 | 563 |
| Gorai.005G245900 | 161 | 17.95 | -8.5 | 4.508 | -1.052 | 947 | 486 | 48.6 | 2 | 473.5 | 906 |
| Gorai.005G246000 | 284 | 31.321 | -5 | 5.194 | -0.106 | 1,113 | 855 | 43.2 | 9 | 123.7 | 187.6 |
| Gorai.005G246100 | 206 | 23.482 | -1.5 | 5.67 | 0.133 | 1,098 | 621 | 42 | 1 | 1,098.00 | No intron |
| Gorai.005G246200 | 863 | 95.876 | -2.5 | 6.283 | -0.188 | 2,792 | 2,592 | 43.7 | 1 | 2,792.00 | No intron |
| Gorai.005G246300 | 648 | 72.429 | -17.5 | 4.932 | -0.397 | 2,898 | 1,947 | 40.2 | 11 | 263.5 | 335.2 |
| Gorai.005G246400 | 661 | 73.365 | 1 | 6.587 | -0.674 | 2,968 | 1,986 | 44.5 | 6 | 494.7 | 163.8 |
| Gorai.005G246500 | 598 | 66.374 | 43 | 10.194 | -0.624 | 1,797 | 1,797 | 41.6 | 20 | 89.9 | 149.9 |
| Gorai.005G246600 | 732 | 81.963 | 18 | 8.604 | -0.306 | 3,220 | 2,199 | 41 | 17 | 189.4 | 115.7 |
| Gorai.005G246700 | 216 | 24.835 | 8.5 | 8.262 | -0.789 | 1,220 | 651 | 48.8 | 5 | 244 | 437 |
| Gorai.005G246800 | 478 | 54.651 | 7 | 7.324 | -0.465 | 1,502 | 1,437 | 44.7 | 7 | 214.6 | 308 |
| Gorai.005G246900 | 465 | 53.506 | 4.5 | 6.89 | -0.454 | 1,607 | 1,398 | 42 | 7 | 229.6 | 186 |
| Gorai.005G260300 | 225 | 24.975 | 6 | 9.549 | -1.079 | 1,458 | 678 | 45.6 | 4 | 364.5 | 686.7 |
| Gorai.005G260400 | 572 | 65.995 | 8 | 7.233 | -0.841 | 2,088 | 1,719 | 42.4 | 6 | 348 | 338.6 |
| Gorai.005G260500 | 833 | 94.12 | 24.5 | 9.697 | -0.187 | 2,962 | 2,502 | 41.8 | 11 | 269.3 | 187.6 |
| Gorai.005G260600 | 288 | 31.084 | 0 | 6.457 | 0.01 | 1,016 | 867 | 41.4 | 3 | 338.7 | 335 |
| Gorai.005G260700 | 523 | 57.277 | 8.5 | 8.887 | -0.175 | 2,133 | 1,572 | 44.1 | 8 | 266.6 | 245.6 |
| Gorai.005G260800 | 256 | 29.286 | -2.5 | 6.028 | -0.465 | 952 | 771 | 50.3 | 3 | 317.3 | 120 |
| Gorai.005G260900 | 497 | 54.248 | 27 | 10.173 | -0.098 | 2,534 | 1,494 | 44.6 | 5 | 506.8 | 267.8 |
| Gorai.006G001800 | 290 | 32.364 | -9.5 | 4.989 | -0.6 | 883 | 873 | 41.6 | 6 | 147.2 | 106 |
| Gorai.006G001900 | 603 | 68.168 | 13.5 | 8.455 | 0.205 | 2,036 | 1,812 | 40.3 | 1 | 2,036.00 | No intron |
| Gorai.006G002000 | 383 | 43.016 | 1.5 | 6.7 | -0.375 | 2,927 | 1,152 | 45.9 | 7 | 418.1 | 335.3 |
| Gorai.006G002100 | 82 | 9.408 | 6 | 9.319 | -0.27 | 249 | 249 | 43 | 1 | 249 | No intron |
| Gorai.006G004700 | 255 | 28.407 | 7.5 | 8.578 | -0.473 | 1,234 | 768 | 41.1 | 6 | 205.7 | 412 |
| Gorai.006G004800 | 237 | 27.23 | 25 | 10.614 | -0.746 | 1,210 | 714 | 40.1 | 8 | 151.3 | 397.3 |
| Gorai.006G004900 | 108 | 12.448 | -5 | 4.729 | -0.071 | 662 | 327 | 40.4 | 2 | 331 | 2,303.00 |
| Gorai.006G005900 | 154 | 16.53 | -3.5 | 4.619 | -0.034 | 465 | 465 | 39.1 | 1 | 465 | No intron |
| Gorai.006G009400 | 389 | 44.157 | 6.5 | 8.682 | -0.294 | 1,966 | 1,170 | 42.7 | 5 | 393.2 | 275.3 |
| Gorai.006G009500 | 409 | 45.866 | -4.5 | 5.204 | 0.088 | 2,187 | 1,230 | 41.9 | 10 | 218.7 | 618.4 |
| Gorai.006G009600 | 366 | 41.563 | -12.5 | 4.72 | -0.575 | 1,746 | 1,101 | 38.9 | 7 | 249.4 | 463.3 |
| Gorai.006G009700 | 377 | 41.299 | -3 | 6.028 | -0.437 | 1,799 | 1,134 | 41.7 | 2 | 899.5 | 91 |
| Gorai.006G018200 | 930 | 103.651 | 0.5 | 6.539 | -0.18 | 2,793 | 2,793 | 39 | 4 | 698.3 | 584.3 |
| Gorai.006G018600 | 417 | 47.069 | 4.5 | 7.666 | -0.25 | 1,276 | 1,254 | 36.8 | 3 | 425.3 | 135.5 |
| Gorai.006G018700 | 506 | 57.109 | 7.5 | 7.828 | -0.233 | 1,521 | 1,521 | 38.7 | 4 | 380.3 | 922.7 |
| Gorai.006G018800 | 891 | 100.005 | 10.5 | 7.366 | -0.204 | 2,676 | 2,676 | 38.9 | 5 | 535.2 | 884.3 |
| Gorai.006G019400 | 225 | 24.797 | -1.5 | 5.697 | 0.649 | 678 | 678 | 39.5 | 1 | 678 | No intron |
| Gorai.006G020700 | 197 | 23.068 | 19.5 | 10.942 | -0.743 | 993 | 594 | 47.3 | 3 | 331 | 465 |
| Gorai.006G020800 | 1,059 | 115.577 | 25 | 9.271 | -0.008 | 3,261 | 3,180 | 41.2 | 5 | 652.2 | 917.5 |
| Gorai.006G044200 | 1,701 | 189.091 | -35 | 5.346 | -0.797 | 5,869 | 5,106 | 44.4 | 11 | 533.5 | 182.5 |
| Gorai.006G050500 | 94 | 10.9 | 1 | 8.22 | -0.286 | 285 | 285 | 43.5 | 2 | 142.5 | 94 |
| Gorai.006G050600 | 229 | 25.54 | -12.5 | 4.423 | -0.163 | 690 | 690 | 42.9 | 2 | 345 | 74 |
| Gorai.006G051100 | 464 | 48.76 | 15.5 | 10.143 | 0.657 | 1,723 | 1,395 | 44.5 | 6 | 287.2 | 296.4 |
| Gorai.006G053000 | 582 | 64.506 | -9 | 5.299 | -0.238 | 2,090 | 1,749 | 43.6 | 13 | 160.8 | 423.1 |
| Gorai.006G056600 | 1,038 | 113.71 | -13.5 | 5.519 | -0.405 | 4,441 | 3,117 | 43.1 | 13 | 341.6 | 1,044.60 |
| Gorai.006G063500 | 671 | 73.923 | 23.5 | 9.675 | -0.385 | 2,510 | 2,016 | 45.5 | 7 | 358.6 | 255.2 |
| Gorai.006G075300 | 413 | 45.026 | 4 | 7.225 | -0.463 | 1,569 | 1,242 | 43.2 | 12 | 130.8 | 355.3 |
| Gorai.006G075400 | 393 | 43.591 | 26.5 | 9.942 | -0.242 | 1,653 | 1,182 | 45.9 | 6 | 275.5 | 258.8 |
| Gorai.006G075500 | 225 | 24.101 | 2 | 7.455 | 0.126 | 1,163 | 678 | 45.3 | 2 | 581.5 | 1,885.00 |
| Gorai.006G075600 | 355 | 39.898 | 3.5 | 7.068 | -0.089 | 1,536 | 1,068 | 42.6 | 12 | 128 | 267 |
| Gorai.006G079800 | 156 | 17.893 | 3 | 8.705 | -0.561 | 938 | 471 | 40.3 | 1 | 938 | No intron |
| Gorai.006G080200 | 239 | 25.987 | 7.5 | 9.837 | -0.345 | 720 | 720 | 40 | 2 | 360 | 250 |
| Gorai.006G080300 | 84 | 9.77 | -1 | 4.544 | 0.651 | 255 | 255 | 47.1 | 1 | 255 | No intron |
| Gorai.006G080400 | 526 | 58.942 | -3 | 6.205 | -0.21 | 1,616 | 1,581 | 40.7 | 10 | 161.6 | 374.8 |
| Gorai.006G080700 | 644 | 73.044 | -1 | 6.438 | -0.3 | 2,749 | 1,935 | 44.1 | 10 | 274.9 | 300 |
| Gorai.006G080800 | 289 | 32.984 | 3 | 7.07 | -0.698 | 1,189 | 870 | 42.5 | 3 | 396.3 | 586.5 |
| Gorai.006G082000 | 1,625 | 183.205 | 7.5 | 6.891 | -0.456 | 5,584 | 4,878 | 40.1 | 32 | 174.5 | 435.7 |
| Gorai.006G082400 | 338 | 37.133 | -8 | 4.777 | -0.278 | 1,395 | 1,017 | 46.7 | 3 | 465 | 680 |
| Gorai.006G082500 | 226 | 25.984 | 6 | 8.086 | -0.767 | 1,273 | 681 | 45.1 | 5 | 254.6 | 439.3 |
| Gorai.006G085700 | 325 | 37.437 | 15 | 8.867 | -0.212 | 1,096 | 978 | 40.2 | 10 | 109.6 | 322.4 |
| Gorai.006G085800 | 173 | 20.436 | 7.5 | 9.344 | 0.326 | 522 | 522 | 39.5 | 7 | 74.6 | 120.8 |
| Gorai.006G085900 | 290 | 33.013 | 11 | 9.055 | 0.086 | 873 | 873 | 43 | 12 | 72.8 | 162 |
| Gorai.006G086000 | 781 | 85.555 | 13 | 7.725 | -0.268 | 3,016 | 2,346 | 43 | 17 | 177.4 | 125.8 |
| Gorai.006G088000 | 257 | 28.049 | 7 | 9.088 | -0.543 | 774 | 774 | 49.5 | 1 | 774 | No intron |
| Gorai.006G088100 | 233 | 26.367 | -2.5 | 6.028 | -0.405 | 1,502 | 702 | 47 | 5 | 300.4 | 390.3 |
| Gorai.006G088200 | 151 | 16.838 | 9 | 10.467 | -0.492 | 1,075 | 456 | 43.9 | 3 | 358.3 | 1,246.00 |
| Gorai.006G089900 | 711 | 78.307 | 9.5 | 7.879 | -0.001 | 2,804 | 2,136 | 44.2 | 1 | 2,804.00 | No intron |
| Gorai.006G090000 | 534 | 59.228 | -9.5 | 5.441 | 0.032 | 2,136 | 1,605 | 41.6 | 1 | 2,136.00 | No intron |
| Gorai.006G090100 | 517 | 55.28 | 11.5 | 8.301 | 0.029 | 1,666 | 1,554 | 43 | 14 | 119 | 809.6 |
| Gorai.006G090200 | 116 | 12.674 | 4 | 9.82 | -0.895 | 999 | 351 | 45.3 | 3 | 333 | 518.5 |
| Gorai.006G090300 | 1,800 | 201.595 | -24 | 5.607 | -0.505 | 5,939 | 5,403 | 43.3 | 11 | 539.9 | 149.7 |
| Gorai.006G092300 | 368 | 40.396 | 6 | 8.938 | 0.008 | 1,547 | 1,107 | 45.6 | 13 | 119 | 300.3 |
| Gorai.006G092400 | 125 | 14.349 | 11 | 10.722 | -0.864 | 704 | 378 | 42.1 | 3 | 234.7 | 446 |
| Gorai.006G095500 | 1,209 | 137.408 | 5 | 6.968 | -0.087 | 3,630 | 3,630 | 41.4 | 10 | 363 | 165.8 |
| Gorai.006G095600 | 96 | 10.975 | 4 | 8.228 | -0.179 | 554 | 291 | 46.4 | 3 | 184.7 | 317 |
| Gorai.006G095700 | 419 | 46.903 | -6 | 5.646 | -0.274 | 1,257 | 1,257 | 42.2 | 15 | 83.8 | 244.9 |
| Gorai.006G103100 | 939 | 104.295 | 44 | 9.539 | -0.307 | 2,820 | 2,820 | 45.2 | 23 | 122.6 | 122.8 |
| Gorai.006G103200 | 494 | 53.044 | -6.5 | 4.864 | 0.108 | 1,956 | 1,485 | 42.4 | 10 | 195.6 | 196.1 |
| Gorai.006G103300 | 67 | 8.022 | 3 | 10.208 | -0.71 | 524 | 204 | 35.8 | 2 | 262 | 2,002.00 |
| Gorai.006G109900 | 269 | 30.122 | 5.5 | 8.365 | -0.156 | 1,320 | 810 | 44.2 | 2 | 660 | 90 |
| Gorai.006G111500 | 337 | 38.951 | 2.5 | 7.003 | -0.354 | 1,530 | 1,014 | 44 | 6 | 255 | 116.6 |
| Gorai.006G111600 | 392 | 43.419 | 10 | 9.9 | -0.75 | 1,716 | 1,179 | 49 | 5 | 343.2 | 417 |
| Gorai.006G111700 | 1,194 | 130.48 | 15.5 | 8.24 | -0.721 | 3,875 | 3,585 | 41.9 | 16 | 242.2 | 269 |
| Gorai.006G111800 | 485 | 54.097 | 5 | 7.11 | -0.562 | 2,274 | 1,458 | 45.7 | 13 | 174.9 | 278.6 |
| Gorai.006G117900 | 897 | 100.563 | -18 | 5.158 | -0.397 | 3,146 | 2,694 | 42.1 | 14 | 224.7 | 616.1 |
| Gorai.006G119400 | 100 | 11.569 | 4 | 9.657 | -0.309 | 784 | 303 | 44.2 | 4 | 196 | 531 |
| Gorai.006G119500 | 395 | 43.244 | 14 | 8.7 | -0.165 | 1,261 | 1,188 | 44.3 | 12 | 105.1 | 340.4 |
| Gorai.006G120400 | 793 | 88.605 | 3 | 6.75 | -0.462 | 3,096 | 2,382 | 46.1 | 12 | 258 | 172 |
| Gorai.006G120500 | 410 | 44.905 | 3.5 | 7.569 | -0.161 | 1,584 | 1,233 | 43.5 | 15 | 105.6 | 276.6 |
| Gorai.006G122400 | 1,819 | 200.272 | 1 | 6.556 | 0.047 | 5,838 | 5,460 | 41.5 | 35 | 166.8 | 736.9 |
| Gorai.006G122500 | 286 | 33.606 | 28.5 | 10.579 | -0.367 | 861 | 861 | 42.3 | 6 | 143.5 | 262.4 |
| Gorai.006G122600 | 285 | 33.093 | 28.5 | 10.58 | -0.425 | 1,058 | 858 | 44.3 | 8 | 132.3 | 164.1 |
| Gorai.006G122700 | 503 | 56.348 | 11.5 | 8.884 | -0.311 | 2,001 | 1,512 | 42.2 | 13 | 153.9 | 1,027.60 |
| Gorai.006G122800 | 321 | 36.571 | -3.5 | 6.104 | -0.133 | 1,322 | 966 | 44.5 | 3 | 440.7 | 308 |
| Gorai.006G127700 | 343 | 39.753 | -7.5 | 4.917 | -0.871 | 1,032 | 1,032 | 40.6 | 3 | 344 | 88 |
| Gorai.006G127800 | 217 | 24.257 | 9 | 9.586 | -0.41 | 654 | 654 | 45.4 | 1 | 654 | No intron |
| Gorai.006G127900 | 499 | 57.936 | 18 | 9.56 | -0.914 | 1,791 | 1,500 | 44.5 | 5 | 358.2 | 210 |
| Gorai.006G128700 | 662 | 72.2 | 10.5 | 8.336 | 0.1 | 2,750 | 1,989 | 43.2 | 9 | 305.6 | 674.1 |
| Gorai.006G128800 | 545 | 58.87 | -6.5 | 5.127 | 0.575 | 1,905 | 1,638 | 42.7 | 14 | 136.1 | 122 |
| Gorai.006G128900 | 94 | 11.27 | 10 | 10.416 | -0.641 | 367 | 285 | 41.8 | 4 | 91.8 | 190.7 |
| Gorai.006G131600 | 816 | 91.246 | -20 | 5.024 | -0.602 | 2,734 | 2,451 | 43.7 | 7 | 390.6 | 118.2 |
| Gorai.006G131700 | 505 | 55.592 | -2 | 6.302 | -0.564 | 2,122 | 1,518 | 43.5 | 3 | 707.3 | 340.5 |
| Gorai.006G131800 | 944 | 102.772 | 9 | 8.087 | 0.128 | 3,703 | 2,835 | 41.2 | 2 | 1,851.50 | 105 |
| Gorai.006G131900 | 174 | 19.878 | -12.5 | 4.566 | -0.988 | 874 | 525 | 45.1 | 2 | 437 | 955 |
| Gorai.006G132000 | 341 | 39.429 | 19 | 9.871 | -0.582 | 1,026 | 1,026 | 41.5 | 3 | 342 | 628.5 |
| Gorai.006G132100 | 200 | 23.137 | 10.5 | 9.797 | -0.523 | 603 | 603 | 40 | 1 | 603 | No intron |
| Gorai.006G132200 | 420 | 46.839 | -2.5 | 5.947 | 0.056 | 1,498 | 1,263 | 41.3 | 8 | 187.3 | 519 |
| Gorai.006G149200 | 496 | 53.866 | 14.5 | 8.964 | 0.045 | 1,758 | 1,491 | 44.3 | 6 | 293 | 99.2 |
| Gorai.006G149300 | 255 | 27.422 | 5.5 | 7.735 | 0.195 | 1,293 | 768 | 45.6 | 3 | 431 | 628.5 |
| Gorai.006G164900 | 81 | 9.274 | 13 | 10.887 | -0.799 | 246 | 246 | 47.2 | 1 | 246 | No intron |
| Gorai.006G165000 | 542 | 58.769 | 12 | 8.47 | 0.346 | 1,629 | 1,629 | 46.8 | 3 | 543 | 70 |
| Gorai.006G165100 | 613 | 70.225 | 14 | 8.709 | -0.243 | 1,842 | 1,842 | 37.8 | 1 | 1,842.00 | No intron |
| Gorai.006G165200 | 564 | 63.541 | 14.5 | 9.537 | -0.488 | 2,068 | 1,695 | 43.4 | 8 | 258.5 | 179 |
| Gorai.006G165300 | 270 | 29.632 | 11 | 9.285 | -0.321 | 813 | 813 | 51.3 | 2 | 406.5 | 366 |
| Gorai.006G165400 | 348 | 40.006 | -18.5 | 4.631 | -0.638 | 1,532 | 1,047 | 41.6 | 11 | 139.3 | 280.9 |
| Gorai.006G165500 | 419 | 47.711 | 20.5 | 10.083 | -0.502 | 2,011 | 1,260 | 44.6 | 6 | 335.2 | 364.6 |
| Gorai.006G173300 | 943 | 102.912 | 11 | 8.264 | -0.635 | 2,896 | 2,832 | 46.7 | 7 | 413.7 | 107.5 |
| Gorai.006G173400 | 303 | 34.044 | 13 | 9.461 | -0.746 | 1,531 | 912 | 50.4 | 5 | 306.2 | 386.3 |
| Gorai.006G173500 | 460 | 50.127 | 0.5 | 6.54 | -0.142 | 2,211 | 1,383 | 46.7 | 8 | 276.4 | 152.4 |
| Gorai.006G173600 | 115 | 12.845 | 13.5 | 11.192 | -1.064 | 444 | 348 | 48.6 | 2 | 222 | 54 |
| Gorai.006G173700 | 336 | 35.603 | 13 | 9.348 | -0.623 | 1,548 | 1,011 | 49.6 | 2 | 774 | 183 |
| Gorai.006G173800 | 266 | 29.042 | 8 | 8.412 | -0.494 | 801 | 801 | 47.4 | 2 | 400.5 | 420 |
| Gorai.006G173900 | 171 | 18.875 | 0.5 | 6.642 | -0.056 | 516 | 516 | 49.8 | 1 | 516 | No intron |
| Gorai.006G174000 | 646 | 70.871 | -15.5 | 4.838 | -0.393 | 2,377 | 1,941 | 47.3 | 2 | 1,188.50 | 715 |
| Gorai.006G174100 | 458 | 50.913 | 27 | 10.092 | -0.66 | 1,536 | 1,377 | 40.4 | 4 | 384 | 452.3 |
| Gorai.006G174200 | 37 | 4.272 | -3.5 | 3.883 | 0.743 | 1,325 | 114 | 41.2 | 2 | 662.5 | 112 |
| Gorai.006G186600 | 393 | 41.579 | 2.5 | 7.071 | -0.025 | 1,441 | 1,182 | 45.9 | 3 | 480.3 | 94 |
| Gorai.006G186700 | 1,125 | 124.763 | 9.5 | 7.014 | -0.409 | 3,687 | 3,378 | 48.7 | 11 | 335.2 | 139 |
| Gorai.006G186800 | 257 | 28.638 | 2 | 7.114 | -0.2 | 1,202 | 774 | 46.4 | 8 | 150.3 | 248 |
| Gorai.006G186900 | 439 | 49.512 | 18 | 8.842 | -0.178 | 1,822 | 1,320 | 42 | 14 | 130.1 | 464.1 |
| Gorai.006G187000 | 261 | 29.07 | 5.5 | 7.604 | 0.43 | 1,392 | 786 | 45.2 | 5 | 278.4 | 473.5 |
| Gorai.006G194600 | 581 | 68.075 | -22 | 4.765 | -0.763 | 2,518 | 1,746 | 39.6 | 6 | 419.7 | 98.2 |
| Gorai.006G194700 | 143 | 15.599 | 3.5 | 8.493 | -0.522 | 1,294 | 432 | 53 | 5 | 258.8 | 96.3 |
| Gorai.006G211000 | 503 | 56.548 | 12 | 9.123 | -0.097 | 1,829 | 1,512 | 42.8 | 2 | 914.5 | 340 |
| Gorai.006G211100 | 500 | 56.168 | 10 | 8.66 | -0.067 | 1,503 | 1,503 | 42.4 | 2 | 751.5 | 534 |
| Gorai.006G211200 | 370 | 42.347 | 14.5 | 9.907 | -0.085 | 1,113 | 1,113 | 40.4 | 6 | 185.5 | 436 |
| Gorai.006G211300 | 169 | 18.229 | 5.5 | 8.261 | -0.508 | 1,314 | 510 | 55.1 | 1 | 1,314.00 | No intron |
| Gorai.006G211400 | 907 | 99.74 | 0 | 6.535 | -0.458 | 3,608 | 2,724 | 43.8 | 19 | 189.9 | 236.6 |
| Gorai.006G211500 | 299 | 31.775 | 1.5 | 6.704 | -0.494 | 900 | 900 | 53.4 | 1 | 900 | No intron |
| Gorai.006G212700 | 395 | 43.072 | -0.5 | 6.409 | -0.037 | 1,592 | 1,188 | 43.7 | 12 | 132.7 | 194.9 |
| Gorai.006G212800 | 401 | 42.391 | 0 | 6.505 | -0.016 | 2,138 | 1,206 | 45.9 | 8 | 267.3 | 356.4 |
| Gorai.006G212900 | 765 | 87.506 | 1.5 | 6.627 | -0.707 | 2,731 | 2,298 | 43.6 | 11 | 248.3 | 532.4 |
| Gorai.006G220000 | 795 | 88.201 | 23 | 8.535 | -0.908 | 2,512 | 2,388 | 41.2 | 9 | 279.1 | 508.5 |
| Gorai.006G220100 | 650 | 73.173 | 11 | 8.545 | -0.598 | 3,179 | 1,953 | 45.8 | 3 | 1,059.70 | 867.5 |
| Gorai.006G220200 | 105 | 11.392 | -6.5 | 4.502 | -0.943 | 1,279 | 318 | 43.1 | 2 | 639.5 | 138 |
| Gorai.006G220300 | 510 | 55.714 | 13 | 8.764 | -0.133 | 1,882 | 1,533 | 42.9 | 11 | 171.1 | 182.4 |
| Gorai.006G220400 | 791 | 87.507 | -9 | 5.121 | -0.038 | 2,763 | 2,376 | 43.8 | 13 | 212.5 | 326.1 |
| Gorai.006G220500 | 219 | 24.642 | 7 | 8.778 | -0.11 | 1,227 | 660 | 44.8 | 5 | 245.4 | 455.3 |
| Gorai.006G222400 | 253 | 28.124 | -4.5 | 5.203 | -0.496 | 1,275 | 762 | 44.4 | 7 | 182.1 | 412.5 |
| Gorai.006G222500 | 668 | 74.195 | 2 | 6.848 | -0.604 | 2,743 | 2,007 | 41.6 | 4 | 685.8 | 298.3 |
| Gorai.006G222600 | 315 | 35.162 | 0 | 6.534 | -0.57 | 948 | 948 | 43.7 | 1 | 948 | No intron |
| Gorai.006G222700 | 531 | 59.855 | -23.5 | 4.426 | -0.317 | 2,231 | 1,596 | 42.6 | 8 | 278.9 | 437.3 |
| Gorai.006G233100 | 509 | 57.517 | 16 | 9.043 | -0.363 | 1,978 | 1,530 | 42.7 | 12 | 164.8 | 415.4 |
| Gorai.006G233200 | 315 | 34.439 | 10 | 9.064 | -0.047 | 1,132 | 948 | 44.3 | 6 | 188.7 | 391.2 |
| Gorai.006G233300 | 76 | 8.886 | 9 | 10.62 | -0.139 | 786 | 231 | 42.4 | 2 | 393 | 506 |
| Gorai.006G233400 | 150 | 17.232 | 5.5 | 9.642 | -0.241 | 453 | 453 | 47.2 | 2 | 226.5 | 55 |
| Gorai.006G233500 | 224 | 24.848 | 1.5 | 7.443 | -1.081 | 675 | 675 | 42.4 | 1 | 675 | No intron |
| Gorai.006G233600 | 486 | 53.229 | 10.5 | 8.207 | -0.097 | 1,860 | 1,461 | 45.6 | 9 | 206.7 | 241.5 |
| Gorai.006G233700 | 474 | 52.647 | 8.5 | 7.979 | -0.044 | 2,046 | 1,425 | 41.3 | 3 | 682 | 235.5 |
| Gorai.006G247900 | 283 | 28.921 | -3 | 5.62 | -0.395 | 852 | 852 | 52.2 | 2 | 426 | 156 |
| Gorai.006G248000 | 132 | 15.213 | 9 | 10.19 | -0.469 | 894 | 399 | 42.9 | 6 | 149 | 400.2 |
| Gorai.006G248100 | 206 | 22.973 | -8 | 4.542 | 0.171 | 757 | 621 | 37.5 | 1 | 757 | No intron |
| Gorai.006G248200 | 190 | 21.227 | -11.5 | 4.283 | -0.043 | 573 | 573 | 38.2 | 1 | 573 | No intron |
| Gorai.006G248300 | 210 | 22.673 | -7 | 4.679 | -0.164 | 754 | 633 | 42.7 | 1 | 754 | No intron |
| Gorai.006G248400 | 599 | 67.237 | 12.5 | 8.133 | -0.344 | 2,397 | 1,800 | 41.3 | 1 | 2,397.00 | No intron |
| Gorai.006G248500 | 85 | 10.022 | 5 | 10.462 | 0.121 | 1,234 | 258 | 36.8 | 3 | 411.3 | 835.5 |
| Gorai.006G248600 | 310 | 35.225 | 20 | 9.894 | -0.789 | 1,132 | 933 | 39.5 | 11 | 102.9 | 97.6 |
| Gorai.006G248700 | 111 | 12.474 | 5.5 | 10.458 | -0.769 | 569 | 336 | 53.6 | 1 | 569 | No intron |
| Gorai.006G248800 | 1,112 | 126.265 | 13.5 | 7.885 | -0.446 | 3,560 | 3,339 | 42 | 17 | 209.4 | 114.3 |
| Gorai.006G248900 | 635 | 73.352 | 18.5 | 7.228 | -0.249 | 1,995 | 1,908 | 39 | 3 | 665 | 161.5 |
| Gorai.006G249000 | 581 | 65.479 | -5.5 | 6.015 | -0.169 | 2,004 | 1,746 | 41.8 | 19 | 105.5 | 160.1 |
| Gorai.006G249100 | 381 | 43.025 | 6 | 7.931 | -0.381 | 1,213 | 1,143 | 41.1 | 3 | 404.3 | 84.5 |
| Gorai.006G249200 | 364 | 40.888 | 0 | 6.509 | -0.271 | 1,479 | 1,095 | 43.7 | 3 | 493 | 203.5 |
| Gorai.006G249300 | 366 | 41.044 | 0 | 6.511 | -0.19 | 1,294 | 1,101 | 44.8 | 3 | 431.3 | 770.5 |
| Gorai.006G249400 | 363 | 40.794 | 2 | 6.794 | -0.28 | 1,415 | 1,092 | 42.9 | 3 | 471.7 | 358 |
| Gorai.007G005400 | 1,234 | 139.604 | -28.5 | 5.195 | -0.301 | 4,413 | 3,705 | 41.9 | 14 | 315.2 | 358.5 |
| Gorai.007G005500 | 504 | 57.176 | 6 | 7.378 | -0.519 | 1,981 | 1,515 | 40.7 | 15 | 132.1 | 294.6 |
| Gorai.007G005600 | 191 | 22.024 | 13 | 10.373 | -0.468 | 1,029 | 576 | 44.8 | 5 | 205.8 | 134.3 |
| Gorai.007G005700 | 234 | 27.017 | -1 | 6.254 | -0.829 | 1,004 | 705 | 40.3 | 6 | 167.3 | 232 |
| Gorai.007G005800 | 83 | 9.434 | 11.5 | 11.588 | -1.004 | 552 | 252 | 45.2 | 3 | 184 | 95 |
| Gorai.007G005900 | 935 | 104.436 | 29 | 9.386 | -0.63 | 3,362 | 2,808 | 41.2 | 21 | 160.1 | 151.4 |
| Gorai.007G007000 | 452 | 50.695 | 10.5 | 8.587 | -0.343 | 1,809 | 1,359 | 42.7 | 13 | 139.2 | 329.8 |
| Gorai.007G007100 | 694 | 78.301 | 15 | 8.164 | -0.128 | 2,984 | 2,085 | 42.8 | 9 | 331.6 | 417.5 |
| Gorai.007G007200 | 702 | 79.384 | 12 | 7.396 | -0.151 | 2,718 | 2,109 | 43.2 | 9 | 302 | 431.1 |
| Gorai.007G007300 | 44 | 5.295 | 10 | 11.556 | -0.873 | 612 | 135 | 41.5 | 1 | 612 | No intron |
| Gorai.007G007400 | 677 | 76.007 | 12 | 8.26 | -0.207 | 3,033 | 2,034 | 43.2 | 4 | 758.3 | 782.3 |
| Gorai.007G007500 | 324 | 37.215 | 8 | 8.802 | -0.987 | 1,793 | 975 | 41.8 | 2 | 896.5 | 87 |
| Gorai.007G022200 | 1,397 | 157.099 | 12 | 7.134 | -0.223 | 4,194 | 4,194 | 40.8 | 15 | 279.6 | 233.6 |
| Gorai.007G022300 | 267 | 31.401 | 16.5 | 10.283 | -1.295 | 1,134 | 804 | 41.8 | 10 | 113.4 | 238.6 |
| Gorai.007G022400 | 713 | 81.768 | 26.5 | 8.373 | -0.389 | 2,343 | 2,142 | 42.8 | 18 | 130.2 | 249.8 |
| Gorai.007G022500 | 381 | 42.436 | -3.5 | 5.845 | 0.035 | 1,837 | 1,146 | 48.7 | 5 | 367.4 | 244 |
| Gorai.007G022600 | 148 | 16.514 | -15 | 3.984 | -0.424 | 900 | 447 | 44.3 | 5 | 180 | 230.5 |
| Gorai.007G022700 | 914 | 101.324 | 19.5 | 9.059 | -0.806 | 3,279 | 2,745 | 43.8 | 8 | 409.9 | 309.6 |
| Gorai.007G022800 | 283 | 31.974 | 16.5 | 10.355 | -0.67 | 1,355 | 852 | 43.2 | 5 | 271 | 431.8 |
| Gorai.007G033100 | 369 | 42.128 | -1 | 6.326 | -0.331 | 1,967 | 1,110 | 43.2 | 2 | 983.5 | 1,991.00 |
| Gorai.007G033200 | 864 | 97.597 | -6 | 6.024 | -0.179 | 3,230 | 2,595 | 46 | 5 | 646 | 207.8 |
| Gorai.007G033300 | 479 | 52.391 | -18.5 | 4.544 | -0.581 | 2,137 | 1,440 | 46.3 | 3 | 712.3 | 2,022.50 |
| Gorai.007G033400 | 384 | 40.856 | 5 | 8.051 | -0.021 | 1,438 | 1,155 | 52.3 | 3 | 479.3 | 510.5 |
| Gorai.007G033500 | 440 | 48.976 | -0.5 | 6.434 | -0.201 | 1,708 | 1,323 | 43.1 | 12 | 142.3 | 245.8 |
| Gorai.007G033600 | 386 | 44.263 | -5.5 | 5.627 | -0.029 | 1,744 | 1,161 | 42.5 | 6 | 290.7 | 349.6 |
| Gorai.007G033700 | 146 | 15.959 | -1.5 | 5.702 | -0.318 | 1,446 | 441 | 46.7 | 3 | 482 | 525 |
| Gorai.007G043500 | 423 | 47.203 | 18 | 8.972 | -0.548 | 1,859 | 1,272 | 44.7 | 4 | 464.8 | 186.3 |
| Gorai.007G043600 | 258 | 29.295 | 39 | 10.945 | -0.524 | 1,238 | 777 | 46.1 | 8 | 154.8 | 617.3 |
| Gorai.007G043700 | 172 | 19.04 | 12 | 10.064 | 0.187 | 891 | 519 | 50.1 | 4 | 222.8 | 564.7 |
| Gorai.007G043800 | 298 | 33.552 | 6.5 | 8.349 | 0.487 | 994 | 897 | 41.2 | 6 | 165.7 | 94.6 |
| Gorai.007G043900 | 236 | 27.314 | 14 | 9.928 | -0.719 | 1,752 | 711 | 41.4 | 3 | 584 | 136.5 |
| Gorai.007G044000 | 1,706 | 193.607 | 3 | 6.645 | -0.119 | 5,499 | 5,121 | 41.6 | 3 | 1,833.00 | 1,326.00 |
| Gorai.007G050300 | 414 | 46.419 | 16.5 | 9.263 | -0.352 | 2,172 | 1,245 | 46.3 | 7 | 310.3 | 213.8 |
| Gorai.007G050400 | 83 | 9.371 | 3 | 9.019 | -1.049 | 1,396 | 252 | 47.2 | 2 | 698 | 889 |
| Gorai.007G050500 | 297 | 33.489 | 14.5 | 9.166 | -0.96 | 1,829 | 894 | 43.4 | 2 | 914.5 | 405 |
| Gorai.007G050600 | 485 | 52.526 | 4.5 | 8.439 | 0.625 | 1,796 | 1,458 | 44.7 | 18 | 99.8 | 178.3 |
| Gorai.007G050700 | 289 | 33.548 | 6 | 8.425 | -0.558 | 1,674 | 870 | 43.2 | 6 | 279 | 257.6 |
| Gorai.007G050800 | 75 | 8.715 | 8.5 | 9.089 | 0.396 | 893 | 228 | 44.3 | 3 | 297.7 | 555.5 |
| Gorai.007G050900 | 475 | 52.228 | 5.5 | 8.317 | 0.613 | 1,883 | 1,428 | 42.7 | 12 | 156.9 | 213.9 |
| Gorai.007G062900 | 335 | 36.918 | 10.5 | 9.385 | 0.588 | 1,815 | 1,008 | 42.1 | 6 | 302.5 | 773.2 |
| Gorai.007G063000 | 170 | 18.81 | -0.5 | 6.427 | -0.772 | 513 | 513 | 46 | 3 | 171 | 330 |
| Gorai.007G063100 | 249 | 27.183 | 17 | 10.665 | -0.837 | 1,213 | 750 | 47.5 | 8 | 151.6 | 126.1 |
| Gorai.007G063200 | 110 | 12.476 | 3.5 | 8.617 | -0.868 | 582 | 333 | 41.1 | 1 | 582 | No intron |
| Gorai.007G063300 | 375 | 41.031 | 12.5 | 9.306 | 0.635 | 1,208 | 1,128 | 42.7 | 7 | 172.6 | 205.3 |
| Gorai.007G063400 | 459 | 50.594 | -22.5 | 4.55 | -0.834 | 1,729 | 1,380 | 44.1 | 2 | 864.5 | 75 |
| Gorai.007G063500 | 431 | 47.293 | -3.5 | 5.969 | -0.233 | 1,987 | 1,296 | 44.2 | 10 | 198.7 | 158.7 |
| Gorai.007G063600 | 377 | 41.716 | -7.5 | 5.163 | -0.172 | 1,333 | 1,134 | 48.3 | 5 | 266.6 | 3,708.50 |
| Gorai.007G064100 | 334 | 37.818 | 20 | 9.648 | 0.357 | 1,157 | 1,005 | 44.4 | 8 | 144.6 | 295.3 |
| Gorai.007G064200 | 330 | 37.825 | 15 | 9.493 | 0.221 | 1,524 | 993 | 44.4 | 9 | 169.3 | 281 |
| Gorai.007G064300 | 327 | 37.256 | 21 | 9.943 | 0.281 | 1,441 | 984 | 43.5 | 9 | 160.1 | 140.4 |
| Gorai.007G064400 | 118 | 13.332 | 0 | 6.513 | 0.249 | 357 | 357 | 38.4 | 3 | 119 | 713 |
| Gorai.007G064500 | 327 | 37.217 | 12.5 | 9.065 | 0.256 | 1,517 | 984 | 44 | 9 | 168.6 | 273 |
| Gorai.007G069300 | 396 | 43.808 | 4 | 7.292 | -0.157 | 1,863 | 1,191 | 43 | 9 | 207 | 355.6 |
| Gorai.007G069400 | 226 | 25.231 | 7 | 8.274 | -0.507 | 696 | 681 | 47.1 | 2 | 348 | 238 |
| Gorai.007G069500 | 607 | 65.613 | -6 | 5.712 | -1 | 2,240 | 1,824 | 49.5 | 3 | 746.7 | 100.5 |
| Gorai.007G069600 | 312 | 34.674 | -12 | 4.748 | -0.787 | 1,375 | 939 | 45.9 | 8 | 171.9 | 517.6 |
| Gorai.007G069700 | 232 | 24.985 | -43 | 3.694 | -0.981 | 1,383 | 699 | 43.6 | 3 | 461 | 296.5 |
| Gorai.007G069800 | 140 | 16.045 | -7.5 | 4.539 | -0.919 | 423 | 423 | 41.6 | 1 | 423 | No intron |
| Gorai.007G069900 | 351 | 40.057 | 3.5 | 6.8 | -0.216 | 1,775 | 1,056 | 42 | 12 | 147.9 | 421.5 |
| Gorai.007G070000 | 252 | 26.816 | 5 | 8.113 | -0.213 | 1,120 | 759 | 49.9 | 2 | 560 | 95 |
| Gorai.007G070100 | 535 | 60 | -1.5 | 6.253 | -0.006 | 1,608 | 1,608 | 41 | 1 | 1,608.00 | No intron |
| Gorai.007G084600 | 438 | 49.269 | 18 | 9.615 | -0.529 | 2,223 | 1,317 | 46.3 | 6 | 370.5 | 210.6 |
| Gorai.007G084700 | 533 | 59.857 | 44 | 9.975 | -0.434 | 1,920 | 1,602 | 45.8 | 4 | 480 | 375.3 |
| Gorai.007G084800 | 68 | 7.684 | 1 | 7.693 | 0.2 | 952 | 207 | 37.7 | 5 | 190.4 | 119.8 |
| Gorai.007G084900 | 289 | 32.697 | -4.5 | 5.604 | -0.699 | 1,754 | 870 | 42.1 | 5 | 350.8 | 522 |
| Gorai.007G085000 | 101 | 11.712 | -0.5 | 5.869 | -0.429 | 306 | 306 | 38.9 | 3 | 102 | 128 |
| Gorai.007G085100 | 814 | 91.407 | -17.5 | 5.085 | -0.784 | 3,621 | 2,445 | 46.8 | 11 | 329.2 | 135.1 |
| Gorai.007G087300 | 550 | 60.52 | 9 | 7.773 | -0.217 | 2,207 | 1,653 | 42.5 | 9 | 245.2 | 316.9 |
| Gorai.007G087400 | 271 | 29.52 | 1.5 | 7.033 | -0.106 | 1,396 | 816 | 41.1 | 4 | 349 | 107.7 |
| Gorai.007G087500 | 245 | 27.479 | -11 | 4.674 | -0.249 | 1,054 | 738 | 45.7 | 5 | 210.8 | 146.8 |
| Gorai.007G087600 | 753 | 83.7 | -1 | 6.427 | -0.239 | 2,831 | 2,262 | 43.7 | 20 | 141.6 | 176.4 |
| Gorai.007G087700 | 402 | 44.245 | 3 | 6.941 | -0.247 | 1,931 | 1,209 | 44.1 | 14 | 137.9 | 140.5 |
| Gorai.007G087800 | 166 | 18.783 | 4 | 8.262 | -0.539 | 959 | 501 | 42.3 | 5 | 191.8 | 400.8 |
| Gorai.007G090200 | 188 | 21.315 | 14 | 9.933 | -0.36 | 567 | 567 | 47.8 | 1 | 567 | No intron |
| Gorai.007G090300 | 422 | 48.19 | 5 | 7.757 | -0.518 | 2,252 | 1,269 | 41.7 | 3 | 750.7 | 446.5 |
| Gorai.007G090400 | 745 | 80.465 | 5 | 6.913 | -0.228 | 2,876 | 2,238 | 48.3 | 7 | 410.9 | 146.3 |
| Gorai.007G090500 | 437 | 48.401 | 28 | 10.367 | -0.232 | 1,774 | 1,314 | 44.9 | 2 | 887 | 68 |
| Gorai.007G090600 | 151 | 17.108 | 23 | 11.074 | -0.466 | 952 | 456 | 47.6 | 5 | 190.4 | 244.5 |
| Gorai.007G090700 | 637 | 71.663 | 8 | 7.336 | 0.077 | 1,914 | 1,914 | 40 | 1 | 1,914.00 | No intron |
| Gorai.007G090800 | 310 | 34.163 | -4 | 5.687 | -1.039 | 1,468 | 933 | 43.4 | 7 | 209.7 | 284.5 |
| Gorai.007G090900 | 307 | 34.82 | 4 | 7.421 | 0.165 | 1,702 | 924 | 44 | 8 | 212.8 | 234.6 |
| Gorai.007G091000 | 188 | 21.036 | -2.5 | 5.668 | -0.457 | 1,111 | 567 | 46.6 | 5 | 222.2 | 365.5 |
| Gorai.007G091100 | 686 | 77.489 | 10 | 8.069 | -0.422 | 2,465 | 2,061 | 40.7 | 12 | 205.4 | 236.8 |
| Gorai.007G093500 | 676 | 77.785 | 34.5 | 9.934 | -0.471 | 2,709 | 2,031 | 40.3 | 11 | 246.3 | 158.8 |
| Gorai.007G093600 | 161 | 18.173 | 7 | 10.125 | -0.653 | 1,006 | 486 | 47.7 | 6 | 167.7 | 288.8 |
| Gorai.007G093700 | 243 | 27.895 | 8.5 | 9.13 | -0.674 | 1,152 | 732 | 44.5 | 8 | 144 | 346.9 |
| Gorai.007G093800 | 226 | 25.945 | 7 | 9.226 | -0.749 | 1,199 | 681 | 44.8 | 8 | 149.9 | 692.3 |
| Gorai.007G099100 | 699 | 78.6 | -10 | 5.684 | -0.373 | 2,978 | 2,100 | 44.5 | 10 | 297.8 | 413 |
| Gorai.007G099200 | 536 | 60.735 | 1 | 6.625 | -0.296 | 2,323 | 1,611 | 44.6 | 9 | 258.1 | 212.8 |
| Gorai.007G099300 | 631 | 71.255 | -13.5 | 5.224 | -0.401 | 2,602 | 1,896 | 40.9 | 8 | 325.3 | 122 |
| Gorai.007G099400 | 491 | 55.199 | -9.5 | 5.108 | -0.41 | 3,547 | 1,476 | 42.2 | 7 | 506.7 | 141 |
| Gorai.007G099500 | 2,246 | 248.662 | 42.5 | 8.351 | -0.274 | 7,266 | 6,741 | 42.6 | 15 | 484.4 | 368.2 |
| Gorai.007G099600 | 734 | 82.643 | -3.5 | 6.266 | -0.4 | 2,632 | 2,205 | 43 | 9 | 292.4 | 165.1 |
| Gorai.007G128700 | 1,247 | 135.025 | 6.5 | 7.312 | -0.336 | 4,514 | 3,744 | 43 | 27 | 167.2 | 212.4 |
| Gorai.007G193400 | 309 | 32.503 | -13.5 | 4.191 | -0.067 | 1,372 | 930 | 49.9 | 3 | 457.3 | 106 |
| Gorai.007G193500 | 91 | 10.7 | 11.5 | 10.484 | -0.768 | 410 | 276 | 40.2 | 3 | 136.7 | 591.5 |
| Gorai.007G193600 | 290 | 29.871 | -8 | 4.208 | 0.153 | 1,478 | 873 | 49.7 | 3 | 492.7 | 212.5 |
| Gorai.007G221900 | 405 | 45.109 | -10 | 5.026 | 0.047 | 1,353 | 1,218 | 47.1 | 2 | 676.5 | 243 |
| Gorai.007G222800 | 346 | 39.949 | -7.5 | 4.965 | -0.257 | 2,127 | 1,041 | 36.8 | 5 | 425.4 | 84.5 |
| Gorai.007G222900 | 1,239 | 139.37 | 4 | 6.764 | -0.141 | 3,902 | 3,720 | 42.5 | 5 | 780.4 | 341 |
| Gorai.007G229800 | 128 | 14.13 | 7 | 8.991 | -0.159 | 1,127 | 387 | 50.4 | 2 | 563.5 | 103 |
| Gorai.007G235800 | 490 | 54.006 | 3 | 7.377 | 0.791 | 1,862 | 1,473 | 46.5 | 8 | 232.8 | 183.7 |
| Gorai.007G236900 | 89 | 10.701 | 8 | 9.92 | 0.266 | 440 | 270 | 33 | 2 | 220 | 379 |
| Gorai.007G237000 | 348 | 39.122 | 5 | 7.995 | -0.464 | 2,923 | 1,047 | 46.6 | 2 | 1,461.50 | 1,916.00 |
| Gorai.007G246600 | 259 | 29.673 | 10.5 | 9.536 | -0.247 | 1,416 | 780 | 40.9 | 9 | 157.3 | 337.4 |
| Gorai.007G265200 | 180 | 20.993 | 25 | 10.893 | -0.658 | 833 | 543 | 36.6 | 5 | 166.6 | 392 |
| Gorai.007G272800 | 79 | 9.266 | 5.5 | 9.496 | 0.234 | 1,346 | 240 | 39.2 | 4 | 336.5 | 172.3 |
| Gorai.007G273100 | 509 | 54.517 | 6 | 7.775 | 0.401 | 2,172 | 1,530 | 48.8 | 1 | 2,172.00 | No intron |
| Gorai.007G273900 | 1,484 | 168.808 | -24.5 | 5.384 | -0.645 | 5,376 | 4,455 | 41.4 | 26 | 206.8 | 432.4 |
| Gorai.007G274000 | 572 | 64.593 | -18.5 | 4.706 | -0.252 | 2,682 | 1,719 | 43.1 | 2 | 1,341.00 | 1,298.00 |
| Gorai.007G274400 | 403 | 46.716 | 8.5 | 7.001 | -0.334 | 1,487 | 1,212 | 38.5 | 5 | 297.4 | 278.5 |
| Gorai.007G274500 | 830 | 92.819 | 20.5 | 8.434 | -0.127 | 2,567 | 2,493 | 41.8 | 2 | 1,283.50 | 33 |
| Gorai.007G274600 | 102 | 11.7 | 3.5 | 9.2 | -0.261 | 549 | 309 | 42.1 | 2 | 274.5 | 1,121.00 |
| Gorai.007G274700 | 448 | 49.357 | -3.5 | 6.124 | -0.7 | 2,019 | 1,347 | 44.2 | 9 | 224.3 | 273.6 |
| Gorai.007G274800 | 392 | 44.319 | 16.5 | 9.464 | -0.167 | 1,749 | 1,179 | 43.1 | 10 | 174.9 | 400 |
| Gorai.007G274900 | 421 | 46.688 | -19 | 4.615 | -0.739 | 2,554 | 1,266 | 40 | 4 | 638.5 | 81.7 |
| Gorai.007G278200 | 105 | 11.28 | 1 | 7.514 | -0.494 | 527 | 318 | 52.2 | 1 | 527 | No intron |
| Gorai.007G278300 | 191 | 21.536 | 0.5 | 6.775 | -0.705 | 629 | 576 | 45.5 | 2 | 314.5 | 121 |
| Gorai.007G278600 | 284 | 31.316 | 25.5 | 10.778 | -1.06 | 1,348 | 855 | 48.2 | 7 | 192.6 | 389.8 |
| Gorai.007G278700 | 138 | 14.459 | 4.5 | 9.992 | -0.196 | 934 | 417 | 45.3 | 5 | 186.8 | 189.5 |
| Gorai.007G278800 | 550 | 59.653 | -21 | 4.852 | -0.533 | 1,971 | 1,653 | 46.2 | 8 | 246.4 | 690.9 |
| Gorai.007G278900 | 737 | 83.904 | 4 | 6.692 | -0.459 | 3,117 | 2,214 | 45.1 | 2 | 1,558.50 | 820 |
| Gorai.007G280200 | 320 | 36.597 | -0.5 | 6.35 | -0.268 | 1,776 | 963 | 42.3 | 8 | 222 | 787.3 |
| Gorai.007G280400 | 302 | 30.368 | -2 | 5.954 | -0.419 | 909 | 909 | 50.5 | 1 | 909 | No intron |
| Gorai.007G280500 | 407 | 45.458 | 1.5 | 6.87 | -0.756 | 1,879 | 1,224 | 41.3 | 7 | 268.4 | 274.3 |
| Gorai.007G280600 | 773 | 86.946 | 9 | 7.274 | 0.01 | 2,409 | 2,322 | 39.7 | 1 | 2,409.00 | No intron |
| Gorai.007G286400 | 860 | 97.573 | -12.5 | 5.561 | -0.331 | 3,191 | 2,583 | 41.9 | 5 | 638.2 | 285.3 |
| Gorai.007G287300 | 118 | 10.589 | -1 | 4.109 | -0.329 | 955 | 357 | 68.1 | 2 | 477.5 | 295 |
| Gorai.007G287400 | 126 | 13.604 | 1 | 7.727 | 0.295 | 603 | 381 | 46.7 | 1 | 603 | No intron |
| Gorai.007G287500 | 387 | 43.044 | 4 | 7.03 | -0.676 | 1,644 | 1,164 | 43.6 | 7 | 234.9 | 101 |
| Gorai.007G287600 | 887 | 97.201 | -14 | 5.2 | -0.668 | 3,469 | 2,664 | 44.4 | 16 | 216.8 | 291.5 |
| Gorai.007G287700 | 479 | 52.242 | -8.5 | 4.714 | -0.024 | 2,509 | 1,440 | 43.2 | 2 | 1,254.50 | 1,078.00 |
| Gorai.007G287800 | 287 | 31.463 | 7.5 | 8.478 | -0.567 | 1,743 | 864 | 46.3 | 2 | 871.5 | 107 |
| Gorai.007G287900 | 282 | 31.975 | 12 | 9.334 | -0.385 | 1,516 | 849 | 42.6 | 7 | 216.6 | 465.5 |
| Gorai.007G288000 | 149 | 16.408 | 9 | 10.138 | -0.118 | 2,123 | 450 | 43.3 | 9 | 235.9 | 290.6 |
| Gorai.007G288100 | 101 | 11.469 | 8.5 | 9.701 | -0.504 | 523 | 306 | 45.8 | 2 | 261.5 | 1,892.00 |
| Gorai.007G304300 | 444 | 49.545 | 13.5 | 9.089 | -0.234 | 2,126 | 1,335 | 42.6 | 14 | 151.9 | 314.5 |
| Gorai.007G304400 | 173 | 18.693 | -1.5 | 5.721 | -0.077 | 976 | 522 | 48.7 | 4 | 244 | 178 |
| Gorai.007G304500 | 167 | 18.226 | 3 | 7.875 | -0.145 | 1,045 | 504 | 44.6 | 4 | 261.3 | 160.7 |
| Gorai.007G304600 | 193 | 22.055 | 2.5 | 7.703 | -0.166 | 1,181 | 582 | 45 | 3 | 393.7 | 571.5 |
| Gorai.007G304700 | 255 | 30.184 | 9.5 | 7.52 | -0.347 | 768 | 768 | 39.6 | 1 | 768 | No intron |
| Gorai.007G306000 | 188 | 20.646 | -4 | 5.031 | -0.737 | 1,502 | 567 | 43.4 | 4 | 375.5 | 395.7 |
| Gorai.007G306100 | 414 | 47.213 | -8 | 4.892 | -0.813 | 1,245 | 1,245 | 42.2 | 5 | 249 | 137.5 |
| Gorai.007G306200 | 968 | 106.787 | -12.5 | 5.6 | -0.297 | 3,242 | 2,907 | 44.7 | 20 | 162.1 | 266.6 |
| Gorai.007G306300 | 589 | 65.843 | 8 | 8.055 | -0.115 | 1,829 | 1,770 | 37.6 | 3 | 609.7 | 189.5 |
| Gorai.007G307100 | 1,487 | 167.296 | -6.5 | 6.24 | -0.042 | 4,951 | 4,464 | 40.9 | 27 | 183.4 | 694.3 |
| Gorai.007G307200 | 650 | 70.838 | 13.5 | 7.67 | -0.434 | 2,498 | 1,953 | 46 | 9 | 277.6 | 157.5 |
| Gorai.007G307300 | 42 | 4.877 | -1 | 4.499 | 0.879 | 244 | 129 | 35.7 | 1 | 244 | No intron |
| Gorai.007G307400 | 107 | 11.823 | 2 | 7.67 | -0.657 | 1,009 | 324 | 40.4 | 2 | 504.5 | 118 |
| Gorai.007G318500 | 1,256 | 143.009 | 6 | 6.869 | -0.2 | 3,912 | 3,771 | 39.6 | 5 | 782.4 | 457 |
| Gorai.007G318600 | 99 | 11.029 | 5 | 9.32 | 0.631 | 618 | 300 | 37 | 1 | 618 | No intron |
| Gorai.007G318700 | 802 | 91.303 | 3.5 | 6.945 | -0.087 | 2,556 | 2,409 | 38.9 | 2 | 1,278.00 | 73 |
| Gorai.007G318800 | 797 | 90.585 | 8.5 | 7.658 | -0.212 | 2,528 | 2,394 | 40.1 | 4 | 632 | 656.3 |
| Gorai.007G318900 | 1,257 | 142.857 | 14 | 7.413 | -0.188 | 3,997 | 3,774 | 39.4 | 5 | 799.4 | 459.3 |
| Gorai.007G319300 | 1,097 | 123.578 | -7.5 | 5.987 | -0.206 | 4,489 | 3,294 | 41.3 | 18 | 249.4 | 283.1 |
| Gorai.007G319400 | 458 | 49.929 | -0.5 | 6.334 | -0.127 | 1,756 | 1,377 | 41.2 | 12 | 146.3 | 272.1 |
| Gorai.007G319500 | 393 | 44.285 | -19.5 | 4.395 | -0.002 | 1,897 | 1,182 | 39.7 | 2 | 948.5 | 800 |
| Gorai.007G319600 | 330 | 38.599 | 25.5 | 10.219 | -0.731 | 1,345 | 993 | 41.5 | 8 | 168.1 | 276 |
| Gorai.007G319700 | 140 | 15.784 | -13.5 | 4.093 | -0.489 | 880 | 423 | 38.1 | 5 | 176 | 573.3 |
| Gorai.007G324500 | 495 | 56.089 | 9 | 8.253 | -0.223 | 1,488 | 1,488 | 36.6 | 3 | 496 | 3,763.50 |
| Gorai.007G324600 | 921 | 106.015 | 8.5 | 7.236 | -0.257 | 3,130 | 2,766 | 39.8 | 3 | 1,043.30 | 500.5 |
| Gorai.007G324700 | 956 | 109.917 | 3 | 6.748 | -0.225 | 3,071 | 2,871 | 40.4 | 3 | 1,023.70 | 892 |
| Gorai.007G327600 | 414 | 46.098 | -2 | 5.994 | -0.124 | 1,245 | 1,245 | 36.5 | 1 | 1,245.00 | No intron |
| Gorai.007G327700 | 837 | 92.567 | -2 | 6.284 | 0.067 | 2,800 | 2,514 | 36.3 | 10 | 280 | 1,216.10 |
| Gorai.007G332600 | 785 | 89.836 | 6 | 7.27 | -0.274 | 2,937 | 2,358 | 39.5 | 6 | 489.5 | 976.4 |
| Gorai.007G332700 | 416 | 47.306 | 18 | 9.356 | -0.177 | 2,867 | 1,251 | 40.5 | 6 | 477.8 | 248.6 |
| Gorai.007G332800 | 306 | 35.623 | 12 | 9.763 | 0.367 | 1,391 | 921 | 40.5 | 3 | 463.7 | 136.5 |
| Gorai.007G332900 | 821 | 94.953 | 14.5 | 8.11 | -0.258 | 3,568 | 2,466 | 38.8 | 6 | 594.7 | 991.4 |
| Gorai.007G333000 | 164 | 18.643 | 3.5 | 8.201 | -0.191 | 890 | 495 | 40 | 1 | 890 | No intron |
| Gorai.007G333100 | 99 | 11.236 | 2.5 | 8.751 | 0.571 | 416 | 300 | 43.7 | 2 | 208 | 167 |
| Gorai.007G335100 | 685 | 78.154 | 6.5 | 7.444 | -0.17 | 2,204 | 2,058 | 40.8 | 4 | 551 | 331 |
| Gorai.007G335200 | 985 | 109.526 | 9 | 7.193 | -0.434 | 3,641 | 2,958 | 43 | 16 | 227.6 | 273.6 |
| Gorai.007G335300 | 295 | 32.688 | 8 | 8.87 | -0.445 | 1,715 | 888 | 42.2 | 10 | 171.5 | 294.1 |
| Gorai.007G340100 | 713 | 79.277 | 15 | 8.214 | 0.012 | 2,142 | 2,142 | 40.6 | 1 | 2,142.00 | No intron |
| Gorai.007G340200 | 943 | 105.15 | -4.5 | 6.174 | 0.097 | 2,832 | 2,832 | 37.6 | 2 | 1,416.00 | 93 |
| Gorai.007G345500 | 388 | 42.086 | 5.5 | 7.577 | 0.212 | 1,511 | 1,167 | 44.6 | 10 | 151.1 | 207.7 |
| Gorai.007G345600 | 291 | 31.494 | 5 | 8.459 | -0.521 | 1,188 | 876 | 47.4 | 1 | 1,188.00 | No intron |
| Gorai.007G345700 | 447 | 51.833 | -3 | 6.016 | 0.224 | 1,344 | 1,344 | 41.9 | 1 | 1,344.00 | No intron |
| Gorai.007G345800 | 399 | 45.224 | -24 | 4.415 | -0.789 | 1,432 | 1,200 | 38.1 | 2 | 716 | 1,515.00 |
| Gorai.007G345900 | 299 | 34.054 | 9 | 8.397 | 0.015 | 1,564 | 900 | 42 | 4 | 391 | 565.7 |
| Gorai.007G346000 | 450 | 49.843 | -13 | 4.885 | -0.206 | 2,518 | 1,353 | 47.5 | 5 | 503.6 | 496.5 |
| Gorai.007G346100 | 765 | 84.837 | -1.5 | 6.349 | -0.15 | 2,711 | 2,298 | 47.7 | 12 | 225.9 | 143.9 |
| Gorai.007G346200 | 252 | 27.601 | 7.5 | 7.625 | -0.348 | 1,019 | 759 | 51 | 2 | 509.5 | 1,095.00 |
| Gorai.007G346300 | 285 | 31.695 | 0.5 | 6.681 | -0.296 | 1,180 | 858 | 41.5 | 3 | 393.3 | 536 |
| Gorai.007G347100 | 339 | 37.135 | 5 | 7.99 | -0.046 | 1,196 | 1,020 | 43.9 | 9 | 132.9 | 570.1 |
| Gorai.007G347200 | 436 | 48.046 | -13.5 | 4.855 | -1.049 | 2,147 | 1,311 | 42.5 | 6 | 357.8 | 262 |
| Gorai.007G347300 | 578 | 64.627 | 15.5 | 9.115 | -0.54 | 2,190 | 1,737 | 44.4 | 8 | 273.8 | 270.9 |
| Gorai.007G347400 | 155 | 16.507 | 17.5 | 9.897 | -0.956 | 609 | 468 | 47.2 | 2 | 304.5 | 234 |
| Gorai.007G347500 | 432 | 47.568 | 14.5 | 9 | -0.157 | 2,024 | 1,299 | 47.4 | 4 | 506 | 643.7 |
| Gorai.007G347600 | 113 | 12.859 | -5 | 4.781 | -0.821 | 789 | 342 | 43.6 | 4 | 197.3 | 420.3 |
| Gorai.007G347700 | 513 | 58.73 | 10 | 8.563 | -0.074 | 1,542 | 1,542 | 43.1 | 1 | 1,542.00 | No intron |
| Gorai.007G347800 | 509 | 58.775 | 15.5 | 9.358 | -0.151 | 1,815 | 1,530 | 42.6 | 1 | 1,815.00 | No intron |
| Gorai.007G355900 | 234 | 26.649 | -12 | 4.563 | -0.408 | 917 | 705 | 40 | 2 | 458.5 | 146 |
| Gorai.007G356000 | 2,205 | 252.737 | -10 | 6.175 | -0.127 | 7,453 | 6,618 | 38.7 | 8 | 931.6 | 557 |
| Gorai.007G357000 | 169 | 20.066 | 14.5 | 10.137 | -0.758 | 510 | 510 | 39.6 | 6 | 85 | 454 |
| Gorai.007G357100 | 165 | 19.008 | 8 | 9.637 | -0.854 | 1,560 | 498 | 42.2 | 5 | 312 | 705.5 |
| Gorai.007G357200 | 105 | 12.199 | 7.5 | 9.921 | -0.337 | 642 | 318 | 42.8 | 2 | 321 | 149 |
| Gorai.007G357300 | 863 | 96.351 | 6.5 | 7.266 | -0.26 | 3,017 | 2,592 | 43.4 | 15 | 201.1 | 433.4 |
| Gorai.007G359900 | 281 | 31.121 | 4 | 7.398 | -0.935 | 1,512 | 846 | 48.5 | 2 | 756 | 1,710.00 |
| Gorai.007G360000 | 470 | 51.514 | -7 | 5.129 | -0.268 | 1,768 | 1,413 | 39.4 | 9 | 196.4 | 347.6 |
| Gorai.007G360100 | 639 | 70.407 | 35 | 10.562 | -0.286 | 2,382 | 1,920 | 44.2 | 10 | 238.2 | 596.6 |
| Gorai.007G360200 | 987 | 110.733 | 5.5 | 6.884 | -0.262 | 3,687 | 2,964 | 41.1 | 20 | 184.4 | 382.3 |
| Gorai.007G360300 | 70 | 7.716 | 4 | 8.265 | -0.864 | 500 | 213 | 51.6 | 1 | 500 | No intron |
| Gorai.007G360400 | 187 | 20.832 | 23.5 | 11.17 | -0.716 | 564 | 564 | 48.8 | 3 | 188 | 429.5 |
| Gorai.007G360500 | 960 | 104.452 | 0 | 6.542 | -0.477 | 3,200 | 2,883 | 43.7 | 23 | 139.1 | 311.1 |
| Gorai.007G362300 | 384 | 44.299 | 17 | 9.16 | -0.07 | 1,926 | 1,155 | 45.9 | 2 | 963 | 2,619.00 |
| Gorai.007G362400 | 421 | 46.885 | -10.5 | 4.913 | -0.567 | 1,859 | 1,266 | 46.8 | 2 | 929.5 | 310 |
| Gorai.007G362500 | 185 | 20.227 | 6.5 | 9.583 | 0.062 | 558 | 558 | 50.9 | 2 | 279 | 31 |
| Gorai.007G362600 | 195 | 21.087 | 6.5 | 9.265 | 0.085 | 742 | 588 | 48.1 | 1 | 742 | No intron |
| Gorai.007G362700 | 617 | 67.37 | 9 | 8.213 | -0.436 | 2,396 | 1,854 | 45.6 | 7 | 342.3 | 384 |
| Gorai.007G363400 | 204 | 23.285 | 9 | 9.08 | -0.486 | 1,078 | 615 | 42.4 | 2 | 539 | 103 |
| Gorai.007G363500 | 170 | 18.266 | -7 | 4.476 | -0.011 | 669 | 513 | 47.8 | 1 | 669 | No intron |
| Gorai.007G363600 | 85 | 10.178 | 11.5 | 10.554 | -0.789 | 258 | 258 | 37.2 | 1 | 258 | No intron |
| Gorai.007G364800 | 80 | 9.119 | -2 | 5.091 | 0.01 | 243 | 243 | 44.9 | 1 | 243 | No intron |
| Gorai.007G364900 | 1,093 | 124.327 | 10.5 | 7.127 | -0.178 | 3,282 | 3,282 | 38.9 | 3 | 1,094.00 | 411 |
| Gorai.007G365000 | 1,204 | 137.744 | -13.5 | 5.691 | -0.307 | 3,615 | 3,615 | 39.2 | 2 | 1,807.50 | 543 |
| Gorai.007G365100 | 76 | 9.348 | 13 | 10.299 | -0.12 | 545 | 231 | 38.1 | 4 | 136.3 | 393.3 |
| Gorai.007G369400 | 107 | 12.489 | 2 | 9.002 | 0.52 | 324 | 324 | 31.5 | 1 | 324 | No intron |
| Gorai.007G369500 | 97 | 11.589 | 0 | 6.506 | -0.924 | 360 | 294 | 41.5 | 1 | 360 | No intron |
| Gorai.007G369600 | 173 | 20.124 | 7.5 | 9.524 | -0.305 | 2,139 | 522 | 42.3 | 4 | 534.8 | 270.3 |
| Gorai.007G369700 | 75 | 8.644 | 8 | 10.898 | 0.536 | 1,008 | 228 | 40.8 | 5 | 201.6 | 446.5 |
| Gorai.007G369900 | 67 | 7.719 | -1.5 | 6.12 | -0.827 | 783 | 204 | 41.2 | 4 | 195.8 | 245.7 |
| Gorai.007G370000 | 253 | 29.07 | 6.5 | 9.355 | -0.566 | 2,570 | 762 | 43 | 8 | 321.3 | 380 |
| Gorai.007G370100 | 131 | 14.366 | -1.5 | 5.961 | -0.2 | 397 | 396 | 42.2 | 4 | 99.3 | 233 |
| Gorai.007G370200 | 104 | 12.367 | 5 | 9.655 | -1.417 | 317 | 315 | 40.6 | 2 | 158.5 | 413 |
| Gorai.007G370600 | 69 | 7.905 | 5.5 | 9.21 | 0.509 | 413 | 210 | 43.3 | 3 | 137.7 | 1,013.00 |
| Gorai.007G370700 | 397 | 46.939 | 15 | 9.88 | -0.592 | 1,819 | 1,194 | 42.5 | 2 | 909.5 | 334 |
| Gorai.007G370800 | 169 | 18.494 | 8 | 9.323 | 0.511 | 510 | 510 | 40.2 | 1 | 510 | No intron |
| Gorai.007G370900 | 842 | 92.143 | 39.5 | 9.824 | -0.462 | 3,119 | 2,529 | 43.9 | 3 | 1,039.70 | 646 |
| Gorai.007G371000 | 974 | 108.494 | -4 | 6.149 | 0.127 | 3,328 | 2,925 | 39.8 | 19 | 175.2 | 384.8 |
| Gorai.007G373800 | 1,284 | 139.333 | -8.5 | 5.889 | -0.008 | 4,788 | 3,855 | 44.7 | 8 | 598.5 | 271.4 |
| Gorai.007G373900 | 95 | 10.687 | 2 | 8.502 | -0.973 | 298 | 288 | 42.4 | 3 | 99.3 | 425 |
| Gorai.007G374000 | 351 | 39.374 | -0.5 | 6.392 | -0.628 | 1,711 | 1,056 | 49.1 | 3 | 570.3 | 610 |
| Gorai.007G374100 | 241 | 25.674 | 1 | 7.691 | 0.17 | 1,092 | 726 | 50 | 1 | 1,092.00 | No intron |
| Gorai.007G374200 | 105 | 11.371 | -1 | 6.054 | 0.089 | 713 | 318 | 49.4 | 3 | 237.7 | 704 |
| Gorai.007G374300 | 532 | 59.717 | 24 | 9.907 | -0.305 | 2,054 | 1,599 | 41.6 | 12 | 171.2 | 179.1 |
| Gorai.007G374800 | 747 | 80.801 | 10.5 | 7.868 | -0.689 | 3,128 | 2,244 | 43.8 | 10 | 312.8 | 272 |
| Gorai.007G374900 | 737 | 81.536 | 19 | 9.554 | 0.186 | 2,214 | 2,214 | 44.2 | 1 | 2,214.00 | No intron |
| Gorai.007G375000 | 622 | 69.754 | 3 | 7.178 | -0.136 | 1,869 | 1,869 | 46.4 | 1 | 1,869.00 | No intron |
| Gorai.007G375100 | 91 | 10.623 | -2 | 5.582 | -0.786 | 1,061 | 276 | 38 | 3 | 353.7 | 104 |
| Gorai.007G375200 | 268 | 28.904 | 32.5 | 11.727 | -0.486 | 1,417 | 807 | 49.6 | 4 | 354.3 | 395 |
| Gorai.007G375300 | 200 | 22.633 | -14 | 4.407 | -0.765 | 1,460 | 603 | 45.4 | 2 | 730 | 1,248.00 |
| Gorai.007G375400 | 238 | 26.57 | 12.5 | 9.793 | -0.607 | 854 | 717 | 46 | 3 | 284.7 | 566.5 |
| Gorai.007G377000 | 242 | 27.78 | 10 | 7.55 | -1.096 | 891 | 729 | 44.2 | 1 | 891 | No intron |
| Gorai.007G377100 | 316 | 35.334 | 7 | 8.436 | -0.259 | 1,478 | 951 | 43 | 7 | 211.1 | 87.5 |
| Gorai.007G377200 | 740 | 79.984 | 0 | 6.511 | -0.076 | 2,576 | 2,223 | 44.3 | 20 | 128.8 | 117.2 |
| Gorai.007G377300 | 153 | 16.917 | -3.5 | 4.989 | -0.93 | 1,085 | 462 | 41.6 | 7 | 155 | 151.2 |
| Gorai.007G377400 | 118 | 12.317 | 2.5 | 7.912 | 0.241 | 714 | 357 | 47.9 | 2 | 357 | 91 |
| Gorai.007G377500 | 346 | 38.165 | 12.5 | 9.19 | -0.649 | 1,361 | 1,041 | 46.4 | 5 | 272.2 | 89.5 |
| Gorai.007G377600 | 290 | 33.208 | 16 | 9.604 | 0.183 | 2,144 | 873 | 42.2 | 11 | 194.9 | 145.9 |
| Gorai.007G377700 | 176 | 18.749 | -8.5 | 4.27 | 0.252 | 1,223 | 531 | 51 | 3 | 407.7 | 105 |
| Gorai.007G377800 | 412 | 44.648 | -7 | 4.933 | -0.139 | 1,703 | 1,239 | 43.9 | 4 | 425.8 | 87 |
| Gorai.007G377900 | 617 | 70.34 | 26.5 | 9.199 | -0.436 | 1,854 | 1,854 | 43.3 | 7 | 264.9 | 94.3 |
| Gorai.007G378000 | 679 | 80.034 | 9.5 | 7.491 | -0.759 | 3,309 | 2,040 | 41.3 | 18 | 183.8 | 255.8 |
| Gorai.007G378100 | 644 | 72.148 | 7 | 7.763 | -0.908 | 2,789 | 1,935 | 45.5 | 1 | 2,789.00 | No intron |
| Gorai.007G378200 | 556 | 60.64 | 24 | 9.552 | 0.011 | 2,019 | 1,671 | 45.9 | 5 | 403.8 | 92.5 |
| Gorai.007G378300 | 314 | 35.689 | 6.5 | 8.918 | -0.8 | 2,034 | 945 | 42.6 | 2 | 1,017.00 | 178 |
| Gorai.007G378400 | 287 | 30.864 | 9 | 9.241 | 0.33 | 1,558 | 864 | 48.3 | 4 | 389.5 | 98.3 |
| Gorai.007G378500 | 558 | 62.447 | -4 | 5.919 | -0.518 | 2,620 | 1,677 | 41.6 | 2 | 1,310.00 | 253 |
| Gorai.008G042500 | 157 | 18.409 | 8 | 9.742 | -0.73 | 474 | 474 | 40.3 | 1 | 474 | No intron |
| Gorai.008G042600 | 385 | 43.726 | 6 | 7.52 | -0.165 | 5,032 | 1,158 | 43.9 | 7 | 718.9 | 146.5 |
| Gorai.008G042700 | 210 | 22.623 | 9 | 8.856 | -0.235 | 1,387 | 633 | 48.2 | 3 | 462.3 | 115.5 |
| Gorai.008G042800 | 297 | 32.933 | 0.5 | 6.689 | -0.364 | 1,079 | 894 | 44.4 | 6 | 179.8 | 107.4 |
| Gorai.008G042900 | 321 | 34.839 | -4 | 5.248 | -0.015 | 1,595 | 966 | 45.4 | 5 | 319 | 227.3 |
| Gorai.008G043000 | 426 | 46.337 | 0.5 | 6.618 | 0.033 | 1,675 | 1,281 | 45.9 | 3 | 558.3 | 223 |
| Gorai.008G048200 | 586 | 62.527 | 0.5 | 6.807 | 0.119 | 2,147 | 1,761 | 42 | 9 | 238.6 | 240 |
| Gorai.008G048300 | 148 | 16.497 | 6.5 | 10.121 | 0.153 | 674 | 447 | 46.5 | 5 | 134.8 | 198.3 |
| Gorai.008G048400 | 192 | 20.961 | 9.5 | 9.908 | -0.084 | 1,163 | 579 | 43.5 | 7 | 166.1 | 337.5 |
| Gorai.008G048500 | 121 | 13.498 | 2.5 | 8.096 | -0.42 | 477 | 366 | 41 | 3 | 159 | 87.5 |
| Gorai.008G048600 | 528 | 57.85 | -4 | 5.606 | -0.225 | 2,145 | 1,587 | 43.7 | 12 | 178.8 | 206.5 |
| Gorai.008G049500 | 118 | 13.354 | 1 | 7.175 | 0.285 | 878 | 357 | 42 | 6 | 146.3 | 437 |
| Gorai.008G049600 | 206 | 22.503 | -1.5 | 6.139 | -0.68 | 1,261 | 621 | 46.4 | 1 | 1,261.00 | No intron |
| Gorai.008G049700 | 1,011 | 108.229 | -19 | 4.822 | 0.285 | 3,628 | 3,036 | 44 | 9 | 403.1 | 336.4 |
| Gorai.008G050900 | 386 | 41.702 | 8 | 8.223 | -0.337 | 2,526 | 1,161 | 47.3 | 7 | 360.9 | 422 |
| Gorai.008G051000 | 504 | 55.834 | 1.5 | 6.641 | 0.023 | 2,063 | 1,515 | 49.1 | 1 | 2,063.00 | No intron |
| Gorai.008G051100 | 495 | 54.723 | -10 | 5.389 | 0.015 | 1,781 | 1,488 | 49.1 | 1 | 1,781.00 | No intron |
| Gorai.008G051200 | 585 | 64.368 | 9 | 8.701 | 0.445 | 2,109 | 1,758 | 44.5 | 1 | 2,109.00 | No intron |
| Gorai.008G051300 | 865 | 97.081 | 7.5 | 6.85 | -0.09 | 2,857 | 2,598 | 43.6 | 1 | 2,857.00 | No intron |
| Gorai.008G051400 | 268 | 29.953 | 25 | 10.734 | -0.11 | 926 | 807 | 48.1 | 1 | 926 | No intron |
| Gorai.008G051900 | 81 | 9.14 | -1 | 5.614 | -0.263 | 766 | 246 | 55.3 | 3 | 255.3 | 753.5 |
| Gorai.008G052000 | 1,008 | 114.03 | -50 | 4.58 | -1.005 | 3,346 | 3,027 | 44.3 | 7 | 478 | 101.7 |
| Gorai.008G052100 | 492 | 55.684 | 23 | 9.475 | -0.256 | 2,017 | 1,479 | 50.7 | 2 | 1,008.50 | 1,043.00 |
| Gorai.008G055000 | 397 | 42.605 | -2.5 | 5.34 | 0.632 | 1,666 | 1,194 | 47.4 | 7 | 238 | 931 |
| Gorai.008G055100 | 732 | 82.616 | 15 | 7.855 | -0.049 | 2,775 | 2,199 | 41.1 | 1 | 2,775.00 | No intron |
| Gorai.008G055200 | 377 | 41.708 | -2 | 6.243 | -0.306 | 2,732 | 1,134 | 42.4 | 11 | 248.4 | 374.9 |
| Gorai.008G062200 | 446 | 48.485 | 0.5 | 6.622 | -0.021 | 1,797 | 1,341 | 45.8 | 8 | 224.6 | 157.7 |
| Gorai.008G062300 | 313 | 32.738 | -8.5 | 4.486 | 0.059 | 1,494 | 942 | 49.4 | 3 | 498 | 117 |
| Gorai.008G064900 | 253 | 29.387 | 14.5 | 9.964 | -0.639 | 1,338 | 762 | 42.4 | 6 | 223 | 174.4 |
| Gorai.008G069700 | 183 | 21.189 | 0.5 | 6.621 | -0.54 | 1,071 | 552 | 41.8 | 6 | 178.5 | 127.2 |
| Gorai.008G069800 | 390 | 44.276 | -1.5 | 5.948 | -0.216 | 1,874 | 1,173 | 43.1 | 2 | 937 | 350 |
| Gorai.008G069900 | 226 | 24.585 | 3.5 | 7.22 | -0.136 | 1,329 | 681 | 47.1 | 2 | 664.5 | 455 |
| Gorai.008G074700 | 98 | 10.786 | 8 | 11.325 | -0.34 | 297 | 297 | 44.1 | 1 | 297 | No intron |
| Gorai.008G077500 | 228 | 25.039 | 8.5 | 8.594 | 0.19 | 1,430 | 687 | 44 | 5 | 286 | 86 |
| Gorai.008G077600 | 426 | 47.183 | 1.5 | 6.747 | -0.069 | 2,059 | 1,281 | 42.5 | 8 | 257.4 | 137.3 |
| Gorai.008G077700 | 384 | 42.168 | 5.5 | 7.593 | -0.735 | 1,786 | 1,155 | 42.5 | 12 | 148.8 | 253.5 |
| Gorai.008G077800 | 509 | 57.038 | -3 | 6.265 | -0.949 | 2,329 | 1,530 | 44.5 | 10 | 232.9 | 104.7 |
| Gorai.008G078200 | 72 | 8.349 | -0.5 | 5.726 | 0.164 | 741 | 219 | 39.7 | 4 | 185.3 | 542 |
| Gorai.008G079500 | 632 | 70.912 | 14.5 | 7.76 | -0.081 | 1,899 | 1,899 | 44.5 | 1 | 1,899.00 | No intron |
| Gorai.008G080800 | 330 | 35.839 | 9 | 8.647 | -0.099 | 1,631 | 993 | 45.4 | 5 | 326.2 | 96.5 |
| Gorai.008G080900 | 359 | 40.071 | 14.5 | 9.73 | -0.574 | 1,171 | 1,080 | 45 | 2 | 585.5 | 346 |
| Gorai.008G081000 | 82 | 9.647 | 5 | 10.109 | 0.067 | 249 | 249 | 34.9 | 1 | 249 | No intron |
| Gorai.008G081100 | 232 | 25.347 | 5 | 8.209 | -0.19 | 1,329 | 699 | 46.6 | 2 | 664.5 | 85 |
| Gorai.008G081200 | 80 | 8.354 | -6 | 4.209 | -1.026 | 243 | 243 | 51 | 1 | 243 | No intron |
| Gorai.008G094200 | 101 | 11.001 | 3.5 | 9.122 | -0.252 | 306 | 306 | 50 | 2 | 153 | 63 |
| Gorai.008G094300 | 813 | 91.137 | -31 | 4.682 | -0.723 | 3,115 | 2,442 | 43.6 | 9 | 346.1 | 224.3 |
| Gorai.008G096000 | 260 | 28.751 | 7.5 | 8.905 | -0.335 | 1,296 | 783 | 42.3 | 9 | 144 | 758.1 |
| Gorai.008G106200 | 183 | 20.446 | 6 | 9.323 | -0.425 | 552 | 552 | 42.6 | 6 | 92 | 584.2 |
| Gorai.008G106300 | 125 | 13.756 | 5.5 | 10.291 | 0.178 | 378 | 378 | 38.9 | 2 | 189 | 30 |
| Gorai.008G106400 | 266 | 30.092 | 2 | 8.385 | -0.428 | 883 | 801 | 43.2 | 7 | 126.1 | 196 |
| Gorai.008G109400 | 151 | 16.931 | 1.5 | 7.601 | -0.048 | 936 | 456 | 47.8 | 4 | 234 | 225.7 |
| Gorai.008G109500 | 532 | 59.734 | 9.5 | 8.197 | -0.419 | 1,765 | 1,599 | 43.9 | 5 | 353 | 213.3 |
| Gorai.008G109600 | 216 | 24.953 | 9 | 8.173 | -1.089 | 1,255 | 651 | 41.6 | 4 | 313.8 | 510 |
| Gorai.008G115000 | 469 | 51.825 | 1.5 | 7.057 | -0.115 | 2,365 | 1,410 | 42.7 | 12 | 197.1 | 392.1 |
| Gorai.008G115100 | 694 | 78.575 | 9 | 7.612 | -0.623 | 2,658 | 2,085 | 43.9 | 6 | 443 | 336 |
| Gorai.008G115200 | 221 | 25.511 | 2 | 7.936 | -0.768 | 1,254 | 666 | 41.6 | 8 | 156.8 | 510.4 |
| Gorai.008G122900 | 552 | 62.565 | -13 | 4.966 | -0.343 | 2,353 | 1,659 | 45.5 | 6 | 392.2 | 269.8 |
| Gorai.008G123000 | 145 | 16.395 | -6 | 4.744 | -0.874 | 613 | 438 | 47.7 | 1 | 613 | No intron |
| Gorai.008G123100 | 299 | 33.422 | 3 | 7.233 | 0.071 | 1,357 | 900 | 41.7 | 1 | 1,357.00 | No intron |
| Gorai.008G123200 | 371 | 39.973 | 6.5 | 9.247 | -0.881 | 1,263 | 1,116 | 47 | 12 | 105.3 | 175.9 |
| Gorai.008G128100 | 122 | 14.336 | 13 | 8.559 | -0.837 | 998 | 369 | 40.1 | 1 | 998 | No intron |
| Gorai.008G128200 | 288 | 32.442 | 4.5 | 8.786 | -0.539 | 1,465 | 867 | 39.2 | 8 | 183.1 | 202.3 |
| Gorai.008G129100 | 213 | 22.539 | 0 | 6.506 | -0.801 | 1,337 | 642 | 49.7 | 5 | 267.4 | 607.8 |
| Gorai.008G132300 | 90 | 9.887 | 0 | 6.516 | 0.479 | 587 | 273 | 38.5 | 6 | 97.8 | 484 |
| Gorai.008G132400 | 370 | 42.614 | 0.5 | 6.567 | -0.779 | 1,428 | 1,113 | 43.1 | 1 | 1,428.00 | No intron |
| Gorai.008G135500 | 657 | 71.308 | 18.5 | 9.338 | -0.469 | 3,239 | 1,974 | 44.3 | 6 | 539.8 | 389 |
| Gorai.008G135600 | 363 | 39.404 | 3 | 6.948 | -0.245 | 1,480 | 1,092 | 42.8 | 8 | 185 | 301.7 |
| Gorai.008G135700 | 987 | 109.565 | -0.5 | 6.479 | -0.405 | 4,124 | 2,964 | 43.9 | 10 | 412.4 | 383.4 |
| Gorai.008G135800 | 136 | 14.735 | 10 | 8.996 | -0.308 | 892 | 411 | 45.7 | 4 | 223 | 517.3 |
| Gorai.008G151500 | 173 | 19.681 | 7 | 9.22 | -0.429 | 1,340 | 522 | 42 | 5 | 268 | 618 |
| Gorai.008G151600 | 392 | 44.778 | 7 | 7.967 | -0.493 | 1,674 | 1,179 | 43.1 | 4 | 418.5 | 368.7 |
| Gorai.008G151700 | 498 | 55.125 | -3.5 | 6.054 | -0.058 | 2,105 | 1,497 | 46.2 | 2 | 1,052.50 | 117 |
| Gorai.008G153300 | 288 | 33.824 | 2 | 6.852 | -0.402 | 1,958 | 867 | 40.5 | 10 | 195.8 | 354.2 |
| Gorai.008G153400 | 131 | 14.842 | 20.5 | 11.157 | -0.56 | 1,321 | 396 | 46.2 | 1 | 1,321.00 | No intron |
| Gorai.008G153500 | 615 | 70.762 | 19 | 8.35 | -0.8 | 1,845 | 1,845 | 42.8 | 6 | 307.5 | 182.8 |
| Gorai.008G155600 | 271 | 30.009 | -10 | 4.545 | -0.781 | 816 | 816 | 48.7 | 1 | 816 | No intron |
| Gorai.008G155700 | 222 | 25.504 | -4.5 | 5.222 | -0.635 | 1,484 | 669 | 47.5 | 6 | 247.3 | 452 |
| Gorai.008G156200 | 343 | 38.1 | 2.5 | 6.846 | -0.713 | 1,723 | 1,032 | 45.3 | 4 | 430.8 | 1,698.00 |
| Gorai.008G156300 | 833 | 93.674 | 8 | 7.028 | -0.003 | 3,716 | 2,502 | 41.2 | 2 | 1,858.00 | 297 |
| Gorai.008G156400 | 202 | 22.748 | -17 | 4.266 | -0.993 | 1,758 | 609 | 43.3 | 2 | 879 | 720 |
| Gorai.008G156500 | 134 | 14.717 | 0.5 | 6.712 | 0.004 | 1,234 | 405 | 50.9 | 3 | 411.3 | 162 |
| Gorai.008G157600 | 426 | 49.862 | 17.5 | 9.43 | 0.212 | 2,273 | 1,281 | 42.4 | 13 | 174.8 | 417.9 |
| Gorai.008G157700 | 392 | 44.687 | 10 | 8.566 | -0.512 | 1,179 | 1,179 | 44 | 6 | 196.5 | 113.4 |
| Gorai.008G191300 | 152 | 17.363 | -1.5 | 5.196 | -0.574 | 1,283 | 459 | 43.8 | 6 | 213.8 | 559 |
| Gorai.008G191400 | 86 | 9.656 | 3.5 | 7.881 | -0.453 | 485 | 261 | 50.2 | 3 | 161.7 | 131.5 |
| Gorai.008G191500 | 81 | 9.017 | -7.5 | 4.097 | -0.043 | 701 | 246 | 50.4 | 1 | 701 | No intron |
| Gorai.008G191600 | 372 | 39.987 | 16.5 | 10.29 | -0.882 | 1,580 | 1,119 | 46.4 | 6 | 263.3 | 250 |
| Gorai.008G191700 | 950 | 106.064 | 5.5 | 7.669 | -0.002 | 3,473 | 2,853 | 42.4 | 18 | 192.9 | 312.4 |
| Gorai.008G192000 | 87 | 9.679 | -1 | 5.665 | -0.444 | 483 | 264 | 39.4 | 2 | 241.5 | 264 |
| Gorai.008G192100 | 171 | 20.093 | -0.5 | 6.262 | -0.068 | 1,246 | 516 | 43 | 5 | 249.2 | 289.3 |
| Gorai.008G192200 | 66 | 7.923 | 6.5 | 9.884 | -0.088 | 201 | 201 | 33.8 | 1 | 201 | No intron |
| Gorai.008G192300 | 378 | 42.94 | 16 | 9.027 | -0.024 | 1,732 | 1,137 | 40.7 | 10 | 173.2 | 433.4 |
| Gorai.008G192400 | 401 | 43.364 | 7 | 7.232 | -0.687 | 1,698 | 1,206 | 49.8 | 2 | 849 | 104 |
| Gorai.008G209500 | 201 | 22.087 | -7.5 | 4.482 | -0.16 | 906 | 606 | 45.7 | 5 | 181.2 | 428.8 |
| Gorai.008G209600 | 496 | 54.933 | 8.5 | 8.067 | -0.059 | 2,288 | 1,491 | 43.1 | 12 | 190.7 | 155 |
| Gorai.008G209700 | 312 | 34.314 | 0 | 6.522 | -0.808 | 1,964 | 939 | 49.6 | 2 | 982 | 932 |
| Gorai.008G209800 | 440 | 49.344 | 12.5 | 8.757 | -0.266 | 2,081 | 1,323 | 42.6 | 12 | 173.4 | 194.7 |
| Gorai.008G214000 | 236 | 26.288 | 2 | 7.572 | -0.668 | 1,459 | 711 | 46.1 | 1 | 1,459.00 | No intron |
| Gorai.008G214100 | 361 | 40.7 | -3.5 | 6.119 | -0.386 | 1,555 | 1,086 | 43 | 11 | 141.4 | 187.8 |
| Gorai.008G214200 | 169 | 20.066 | -1 | 5.386 | -0.681 | 510 | 510 | 36.9 | 4 | 127.5 | 57.7 |
| Gorai.008G214300 | 457 | 51.567 | -9.5 | 4.954 | -0.085 | 1,949 | 1,374 | 44 | 6 | 324.8 | 489.4 |
| Gorai.008G222400 | 409 | 45.144 | 0 | 6.511 | -0.276 | 1,974 | 1,230 | 46.3 | 5 | 394.8 | 207.3 |
| Gorai.008G222500 | 499 | 56.629 | 12.5 | 9.038 | -0.137 | 1,500 | 1,500 | 44.2 | 2 | 750 | 71 |
| Gorai.008G222600 | 501 | 57.474 | 13.5 | 8.553 | -0.185 | 1,506 | 1,506 | 45.5 | 2 | 753 | 1,244.00 |
| Gorai.008G222700 | 934 | 106.521 | 2.5 | 6.69 | -0.2 | 2,805 | 2,805 | 44 | 1 | 2,805.00 | No intron |
| Gorai.008G222800 | 705 | 77.96 | -2 | 6.294 | -0.172 | 2,609 | 2,118 | 42.8 | 11 | 237.2 | 328.7 |
| Gorai.008G247600 | 562 | 64.517 | 18.5 | 9.276 | -0.185 | 2,156 | 1,689 | 47.6 | 2 | 1,078.00 | 675 |
| Gorai.008G247700 | 286 | 32.408 | 4.5 | 8.474 | -0.163 | 861 | 861 | 41.3 | 4 | 215.3 | 514 |
| Gorai.008G247800 | 464 | 50.509 | 5.5 | 8.206 | -0.187 | 1,395 | 1,395 | 49.1 | 1 | 1,395.00 | No intron |
| Gorai.008G247900 | 457 | 49.491 | -6 | 5.594 | -0.529 | 2,093 | 1,374 | 46.7 | 2 | 1,046.50 | 1,309.00 |
| Gorai.008G248000 | 184 | 21.09 | -17 | 4.144 | -0.628 | 966 | 555 | 41.3 | 6 | 161 | 325.2 |
| Gorai.008G248100 | 211 | 22.978 | -18 | 4.19 | -0.485 | 1,783 | 636 | 40.7 | 5 | 356.6 | 503.8 |
| Gorai.008G249000 | 74 | 8.534 | 1 | 6.926 | 0.754 | 401 | 225 | 37.3 | 1 | 401 | No intron |
| Gorai.008G249100 | 197 | 22.37 | 11 | 9.975 | -0.107 | 594 | 594 | 35.9 | 8 | 74.3 | 157.4 |
| Gorai.008G249200 | 647 | 71.459 | -9.5 | 5.364 | -0.243 | 2,393 | 1,944 | 44.3 | 7 | 341.9 | 644.2 |
| Gorai.008G268600 | 427 | 47.702 | -5 | 5.797 | -0.405 | 1,970 | 1,284 | 43 | 8 | 246.3 | 365.9 |
| Gorai.008G268700 | 1,428 | 159.028 | 68.5 | 9.557 | -0.751 | 5,191 | 4,287 | 42.2 | 13 | 399.3 | 303.3 |
| Gorai.008G268800 | 619 | 67.16 | 8 | 7.921 | -0.168 | 2,432 | 1,860 | 44 | 15 | 162.1 | 275.9 |
| Gorai.008G268900 | 600 | 65.607 | 13.5 | 8.622 | -0.247 | 2,247 | 1,803 | 50.6 | 3 | 749 | 343.5 |
| Gorai.008G283400 | 392 | 44.891 | 16.5 | 9.549 | 0.007 | 1,179 | 1,179 | 38.8 | 1 | 1,179.00 | No intron |
| Gorai.008G283500 | 532 | 59.088 | 4 | 7.261 | -0.42 | 2,131 | 1,599 | 43.8 | 5 | 426.2 | 247.3 |
| Gorai.008G283600 | 335 | 35.308 | 6.5 | 8.45 | -0.309 | 1,539 | 1,008 | 48.1 | 5 | 307.8 | 492 |
| Gorai.008G283700 | 141 | 16.25 | 3.5 | 9.453 | -1.279 | 426 | 426 | 40.8 | 4 | 106.5 | 138.3 |
| Gorai.008G283800 | 787 | 87.716 | 23 | 8.541 | -0.285 | 2,592 | 2,364 | 44.1 | 1 | 2,592.00 | No intron |
| Gorai.008G283900 | 291 | 32.386 | -5 | 5.245 | -0.146 | 1,369 | 876 | 47.4 | 6 | 228.2 | 431 |
| Gorai.008G291100 | 591 | 66.387 | -6 | 5.703 | -0.105 | 2,354 | 1,776 | 40.4 | 20 | 117.7 | 350.7 |
| Gorai.008G291200 | 203 | 23.652 | 1.5 | 7.048 | -0.473 | 927 | 612 | 40.5 | 4 | 231.8 | 648.7 |
| Gorai.008G291300 | 78 | 8.915 | 4.5 | 10.357 | -0.992 | 575 | 237 | 42.2 | 1 | 575 | No intron |
| Gorai.008G291400 | 791 | 88.57 | 25.5 | 8.711 | 0.241 | 2,745 | 2,376 | 42.4 | 8 | 343.1 | 152.4 |
| Gorai.008G291500 | 90 | 10.171 | 6 | 10.469 | -0.253 | 704 | 273 | 43.2 | 2 | 352 | 138 |
| Gorai.008G291600 | 153 | 17.463 | 1 | 6.672 | -0.942 | 812 | 462 | 42 | 4 | 203 | 422.7 |
| Gorai.009G002800 | 172 | 18.764 | 7 | 8.858 | -0.292 | 519 | 519 | 49.3 | 1 | 519 | No intron |
| Gorai.009G002900 | 379 | 44.259 | 19.5 | 8.886 | -0.319 | 2,172 | 1,140 | 42.8 | 11 | 197.5 | 287.1 |
| Gorai.009G003000 | 933 | 100.778 | 24 | 7.823 | -0.023 | 3,392 | 2,802 | 43.6 | 9 | 376.9 | 396.1 |
| Gorai.009G003100 | 182 | 21.728 | 13.5 | 10.054 | -0.522 | 1,216 | 549 | 42.4 | 5 | 243.2 | 332.5 |
| Gorai.009G003200 | 613 | 68.392 | 5 | 6.997 | -0.044 | 2,426 | 1,842 | 42.6 | 5 | 485.2 | 296.3 |
| Gorai.009G003300 | 611 | 70.365 | -6.5 | 6.083 | -1.223 | 2,466 | 1,836 | 42 | 14 | 176.1 | 488.5 |
| Gorai.009G008500 | 391 | 43.517 | -12.5 | 4.675 | -0.141 | 2,096 | 1,176 | 49.3 | 3 | 698.7 | 119.5 |
| Gorai.009G008600 | 403 | 44.693 | 7 | 7.69 | -0.061 | 1,576 | 1,212 | 46.8 | 2 | 788 | 87 |
| Gorai.009G008700 | 60 | 7.141 | 2 | 9.252 | 0.423 | 1,047 | 183 | 37.7 | 3 | 349 | 856 |
| Gorai.009G008800 | 372 | 42.068 | 5.5 | 7.915 | -0.833 | 1,650 | 1,119 | 44.5 | 8 | 206.3 | 185.6 |
| Gorai.009G008900 | 361 | 38.912 | -9.5 | 4.741 | -0.177 | 1,181 | 1,086 | 49.5 | 6 | 196.8 | 305.6 |
| Gorai.009G009000 | 120 | 12.662 | 5.5 | 11.752 | 0.239 | 942 | 363 | 47.7 | 1 | 942 | No intron |
| Gorai.009G009100 | 1,152 | 129.68 | -18 | 5.629 | -0.552 | 4,572 | 3,459 | 43.5 | 5 | 914.4 | 617.8 |
| Gorai.009G010400 | 799 | 88.815 | -12 | 5.498 | -0.065 | 3,295 | 2,400 | 38.8 | 6 | 549.2 | 280.8 |
| Gorai.009G010500 | 360 | 40.808 | -15 | 4.421 | -0.107 | 1,303 | 1,083 | 34.8 | 6 | 217.2 | 357.4 |
| Gorai.009G010600 | 673 | 76.314 | 8 | 7.915 | 0.122 | 2,022 | 2,022 | 41.9 | 13 | 155.5 | 109.7 |
| Gorai.009G010700 | 142 | 15.117 | 0.5 | 7.124 | 0.075 | 429 | 429 | 52.4 | 2 | 214.5 | 106 |
| Gorai.009G010800 | 443 | 49.048 | 6.5 | 8.237 | -0.144 | 2,026 | 1,332 | 45.4 | 5 | 405.2 | 284.5 |
| Gorai.009G010900 | 380 | 41.65 | -7 | 5.189 | -0.306 | 1,820 | 1,143 | 46.3 | 4 | 455 | 383 |
| Gorai.009G011300 | 490 | 55.79 | -1.5 | 6.214 | -0.394 | 2,059 | 1,473 | 41.5 | 12 | 171.6 | 424.9 |
| Gorai.009G011400 | 451 | 48.97 | 6 | 7.55 | -0.196 | 2,071 | 1,356 | 45.2 | 6 | 345.2 | 340.8 |
| Gorai.009G011500 | 1,127 | 123.87 | -22.5 | 5.06 | -0.834 | 3,916 | 3,384 | 44 | 5 | 783.2 | 499.8 |
| Gorai.009G011600 | 337 | 37.338 | 4.5 | 8.802 | -0.723 | 1,730 | 1,014 | 44.6 | 4 | 432.5 | 534.7 |
| Gorai.009G011700 | 137 | 15.031 | -11.5 | 4.058 | -0.304 | 1,003 | 414 | 44 | 4 | 250.8 | 437.3 |
| Gorai.009G011800 | 2,183 | 241.098 | 11 | 6.971 | -0.225 | 7,434 | 6,552 | 42.6 | 23 | 323.2 | 476.8 |
| Gorai.009G013300 | 253 | 28.629 | -6 | 4.818 | -0.217 | 1,037 | 762 | 42.9 | 4 | 259.3 | 216.7 |
| Gorai.009G013400 | 343 | 38.504 | 12 | 9.298 | -0.247 | 1,626 | 1,032 | 44.2 | 8 | 203.3 | 171.3 |
| Gorai.009G013500 | 241 | 27.473 | 11 | 8.426 | -0.402 | 1,300 | 726 | 39.4 | 4 | 325 | 81.7 |
| Gorai.009G013600 | 109 | 12.018 | 16.5 | 12.134 | -0.525 | 689 | 330 | 52.7 | 1 | 689 | No intron |
| Gorai.009G013700 | 205 | 22.446 | 28.5 | 12.407 | -0.71 | 618 | 618 | 50.3 | 1 | 618 | No intron |
| Gorai.009G013800 | 637 | 69.63 | 22.5 | 9.236 | 0.529 | 2,362 | 1,914 | 43.9 | 3 | 787.3 | 299.5 |
| Gorai.009G013900 | 258 | 29.705 | 21.5 | 10.445 | -0.725 | 777 | 777 | 44 | 1 | 777 | No intron |
| Gorai.009G023100 | 534 | 57.862 | -8 | 5.013 | -0.057 | 2,060 | 1,605 | 45.2 | 15 | 137.3 | 217.6 |
| Gorai.009G023200 | 717 | 79.549 | 3.5 | 6.818 | -0.443 | 2,814 | 2,154 | 42.3 | 5 | 562.8 | 924.3 |
| Gorai.009G023300 | 158 | 17.316 | 3 | 8.666 | -0.123 | 1,217 | 477 | 48.8 | 2 | 608.5 | 1,498.00 |
| Gorai.009G023400 | 133 | 14.876 | 3.5 | 8.122 | -0.041 | 877 | 402 | 38.6 | 4 | 219.3 | 355 |
| Gorai.009G023500 | 742 | 79.816 | 4.5 | 7.587 | -0.72 | 3,208 | 2,229 | 47.3 | 12 | 267.3 | 222.2 |
| Gorai.009G023600 | 400 | 45.44 | 7 | 7.762 | 0.454 | 1,456 | 1,203 | 44.4 | 13 | 112 | 246.1 |
| Gorai.009G026300 | 163 | 17.491 | -0.5 | 6.256 | 0.04 | 1,179 | 492 | 50.4 | 3 | 393 | 793.5 |
| Gorai.009G026400 | 409 | 44.267 | 4 | 7.728 | -0.164 | 1,446 | 1,230 | 42.8 | 3 | 482 | 177 |
| Gorai.009G026500 | 1,338 | 149.506 | -10 | 6.001 | -0.212 | 4,441 | 4,017 | 42.5 | 15 | 296.1 | 139.4 |
| Gorai.009G026600 | 332 | 35.934 | 6.5 | 7.478 | -0.585 | 1,742 | 999 | 47.6 | 7 | 248.9 | 184.7 |
| Gorai.009G026700 | 310 | 33.193 | 2.5 | 7.166 | -0.585 | 1,387 | 933 | 48.3 | 6 | 231.2 | 171.2 |
| Gorai.009G030900 | 255 | 27.82 | 17 | 10.346 | -0.531 | 1,374 | 768 | 43.6 | 3 | 458 | 113 |
| Gorai.009G031000 | 269 | 30.173 | 10 | 8.518 | 0.436 | 1,216 | 810 | 48.5 | 2 | 608 | 1,194.00 |
| Gorai.009G031100 | 304 | 32.975 | 0 | 6.5 | -0.174 | 915 | 915 | 51.6 | 1 | 915 | No intron |
| Gorai.009G031200 | 201 | 22.552 | 10 | 8.881 | -0.152 | 1,202 | 606 | 40.8 | 4 | 300.5 | 195.3 |
| Gorai.009G031300 | 488 | 54.348 | -6.5 | 5.818 | -0.154 | 1,467 | 1,467 | 42.6 | 1 | 1,467.00 | No intron |
| Gorai.009G031400 | 301 | 33.515 | 13 | 8.652 | -0.819 | 1,449 | 906 | 43.6 | 3 | 483 | 135.5 |
| Gorai.009G031500 | 560 | 61.184 | -3.5 | 6.172 | -0.303 | 2,484 | 1,683 | 44.7 | 2 | 1,242.00 | 1,970.00 |
| Gorai.009G036600 | 618 | 68.593 | 16.5 | 8.937 | -0.164 | 2,743 | 1,857 | 44.6 | 2 | 1,371.50 | 90 |
| Gorai.009G036700 | 495 | 55.491 | 8 | 8.214 | -0.158 | 2,110 | 1,488 | 45.4 | 5 | 422 | 289.5 |
| Gorai.009G036800 | 240 | 25.547 | 9.5 | 9.938 | -0.397 | 1,283 | 723 | 49 | 5 | 256.6 | 243.5 |
| Gorai.009G036900 | 70 | 8.008 | 5 | 9.41 | -0.147 | 664 | 213 | 48.8 | 1 | 664 | No intron |
| Gorai.009G037000 | 142 | 15.704 | 2 | 8.199 | -0.739 | 616 | 429 | 52.2 | 1 | 616 | No intron |
| Gorai.009G037100 | 1,106 | 123.565 | 5 | 6.795 | -0.615 | 4,854 | 3,321 | 45.2 | 13 | 373.4 | 224.8 |
| Gorai.009G040600 | 220 | 23.401 | 0 | 6.494 | 0.017 | 1,316 | 663 | 46.8 | 2 | 658 | 602 |
| Gorai.009G040700 | 519 | 57.928 | -5.5 | 5.659 | -0.192 | 1,919 | 1,560 | 48.7 | 4 | 479.8 | 526 |
| Gorai.009G040800 | 199 | 23.133 | 5.5 | 8.619 | -0.937 | 1,381 | 600 | 43 | 3 | 460.3 | 114 |
| Gorai.009G040900 | 190 | 21.921 | 3.5 | 8.317 | -0.841 | 898 | 573 | 41.2 | 3 | 299.3 | 85 |
| Gorai.009G041000 | 283 | 30.407 | 2.5 | 7.862 | -0.017 | 1,405 | 852 | 47.7 | 3 | 468.3 | 1,268.50 |
| Gorai.009G041100 | 235 | 26.038 | 2 | 7.499 | -0.462 | 736 | 708 | 45.2 | 5 | 147.2 | 114.3 |
| Gorai.009G041200 | 240 | 26.969 | 12 | 9.967 | -0.279 | 1,288 | 723 | 48.5 | 6 | 214.7 | 334.6 |
| Gorai.009G041300 | 743 | 84.141 | 18.5 | 9.038 | -0.077 | 3,411 | 2,232 | 41.3 | 7 | 487.3 | 188.5 |
| Gorai.009G041700 | 361 | 40.864 | 6 | 7.61 | -0.438 | 1,436 | 1,086 | 44.8 | 6 | 239.3 | 511.4 |
| Gorai.009G041800 | 352 | 37.86 | 15 | 9.968 | 0.082 | 1,059 | 1,059 | 44 | 1 | 1,059.00 | No intron |
| Gorai.009G041900 | 92 | 10.934 | 14.5 | 11.288 | -0.876 | 399 | 279 | 35.8 | 1 | 399 | No intron |
| Gorai.009G042000 | 159 | 18.108 | 0.5 | 7.359 | -0.561 | 1,167 | 480 | 47.7 | 1 | 1,167.00 | No intron |
| Gorai.009G042100 | 156 | 17.514 | -0.5 | 6.021 | -0.447 | 896 | 471 | 49.5 | 1 | 896 | No intron |
| Gorai.009G042200 | 504 | 56.489 | 30 | 10.129 | -0.224 | 1,808 | 1,515 | 44.1 | 13 | 139.1 | 205.4 |
| Gorai.009G042300 | 98 | 11.438 | 0 | 6.527 | -0.454 | 1,195 | 297 | 46.1 | 4 | 298.8 | 1,432.30 |
| Gorai.009G042400 | 355 | 41.758 | 1.5 | 6.784 | -0.291 | 1,335 | 1,068 | 41.6 | 9 | 148.3 | 369.9 |
| Gorai.009G042500 | 94 | 11.126 | 3 | 9.385 | -0.159 | 1,119 | 285 | 41.4 | 4 | 279.8 | 152.7 |
| Gorai.009G042600 | 558 | 60.759 | -31 | 4.389 | -0.256 | 3,134 | 1,677 | 43.9 | 6 | 522.3 | 308.2 |
| Gorai.009G042700 | 520 | 59.946 | -5.5 | 5.973 | -0.534 | 2,447 | 1,563 | 40.9 | 13 | 188.2 | 395.9 |
| Gorai.009G045700 | 193 | 21.729 | -2 | 6.089 | -0.454 | 764 | 582 | 47.4 | 1 | 764 | No intron |
| Gorai.009G045800 | 346 | 38.53 | -1 | 6.34 | 0.163 | 1,717 | 1,041 | 43.3 | 11 | 156.1 | 288.6 |
| Gorai.009G045900 | 533 | 59.957 | -7.5 | 5.394 | -0.071 | 2,371 | 1,602 | 43.2 | 5 | 474.2 | 577.5 |
| Gorai.009G051800 | 225 | 25.398 | 4 | 8.438 | 0.37 | 971 | 678 | 42.9 | 6 | 161.8 | 127.2 |
| Gorai.009G051900 | 95 | 10.788 | 27.5 | 12.472 | -0.982 | 790 | 288 | 51.4 | 4 | 197.5 | 189 |
| Gorai.009G052000 | 434 | 48.722 | 14 | 8.881 | -0.228 | 2,276 | 1,305 | 48.5 | 4 | 569 | 336 |
| Gorai.009G052100 | 396 | 42.135 | 20.5 | 9.552 | -0.326 | 1,838 | 1,191 | 46.2 | 3 | 612.7 | 254.5 |
| Gorai.009G052200 | 367 | 40.099 | 2.5 | 6.832 | -0.258 | 1,315 | 1,104 | 44.2 | 4 | 328.8 | 3,508.30 |
| Gorai.009G052300 | 209 | 23.952 | 7.5 | 8.495 | -0.131 | 630 | 630 | 40.3 | 4 | 157.5 | 302.3 |
| Gorai.009G059800 | 291 | 32.532 | 3 | 6.957 | -0.284 | 1,610 | 876 | 42.2 | 8 | 201.3 | 258.3 |
| Gorai.009G059900 | 714 | 81.028 | -18.5 | 4.95 | -0.486 | 2,930 | 2,145 | 43.1 | 3 | 976.7 | 202 |
| Gorai.009G060000 | 562 | 62.208 | -1 | 6.305 | 0.343 | 2,222 | 1,689 | 43.2 | 10 | 222.2 | 133.8 |
| Gorai.009G060100 | 122 | 14.004 | 5.5 | 9.203 | -0.631 | 894 | 369 | 49.6 | 1 | 894 | No intron |
| Gorai.009G060200 | 425 | 46.817 | -3.5 | 6.162 | -0.576 | 2,149 | 1,278 | 48.9 | 6 | 358.2 | 297.8 |
| Gorai.009G066600 | 199 | 22.603 | 22 | 11.43 | -1.218 | 2,242 | 600 | 50.8 | 8 | 280.3 | 188.9 |
| Gorai.009G066700 | 523 | 58.765 | 19.5 | 9.386 | -0.082 | 1,880 | 1,572 | 44.4 | 5 | 376 | 552.8 |
| Gorai.009G066800 | 2,798 | 315.516 | -159.5 | 4.528 | -0.548 | 9,099 | 8,397 | 41.4 | 10 | 909.9 | 239.8 |
| Gorai.009G066900 | 330 | 36.66 | 20.5 | 9.804 | -0.567 | 1,563 | 993 | 50.1 | 3 | 521 | 277.5 |
| Gorai.009G078000 | 532 | 59.506 | -12 | 5.314 | -0.562 | 1,646 | 1,599 | 46.4 | 8 | 205.8 | 101.4 |
| Gorai.009G078100 | 292 | 33.184 | 14 | 8.431 | -0.986 | 879 | 879 | 45.4 | 1 | 879 | No intron |
| Gorai.009G078200 | 416 | 47.007 | -12.5 | 4.836 | -0.41 | 2,296 | 1,251 | 42 | 12 | 191.3 | 208.8 |
| Gorai.009G078300 | 253 | 27.357 | -2.5 | 5.683 | 0.001 | 966 | 762 | 45.9 | 8 | 120.8 | 322.1 |
| Gorai.009G078400 | 845 | 92.564 | 10 | 7.586 | -0.239 | 3,642 | 2,538 | 43.5 | 19 | 191.7 | 211.5 |
| Gorai.009G078500 | 174 | 19.845 | 17 | 10.079 | -0.288 | 866 | 525 | 52.2 | 2 | 433 | 265 |
| Gorai.009G078600 | 587 | 67.882 | 14.5 | 7.634 | -0.137 | 1,926 | 1,764 | 42 | 15 | 128.4 | 172.2 |
| Gorai.009G082000 | 160 | 17.258 | -2 | 5.525 | 0.262 | 1,055 | 483 | 41.6 | 4 | 263.8 | 1,067.30 |
| Gorai.009G082100 | 435 | 47.975 | 30 | 10.842 | -0.777 | 2,242 | 1,308 | 45.3 | 8 | 280.3 | 739.9 |
| Gorai.009G082200 | 357 | 41.347 | 23 | 10.121 | -0.097 | 2,166 | 1,074 | 49.6 | 2 | 1,083.00 | 1,106.00 |
| Gorai.009G082300 | 1,016 | 108.394 | -6.5 | 6.136 | -0.214 | 4,353 | 3,051 | 48.6 | 22 | 197.9 | 306.6 |
| Gorai.009G084500 | 149 | 15.882 | 4.5 | 7.384 | -0.036 | 860 | 450 | 46.4 | 3 | 286.7 | 424.5 |
| Gorai.009G084600 | 95 | 10.819 | 5.5 | 9.51 | -0.067 | 498 | 288 | 44.4 | 2 | 249 | 413 |
| Gorai.009G084700 | 82 | 8.831 | 2.5 | 9.107 | -0.795 | 249 | 249 | 45.8 | 2 | 124.5 | 338 |
| Gorai.009G084800 | 484 | 53.56 | -26.5 | 4.405 | -0.368 | 2,114 | 1,455 | 44.4 | 5 | 422.8 | 373.5 |
| Gorai.009G084900 | 501 | 58.181 | -0.5 | 6.462 | -0.403 | 1,506 | 1,506 | 41.6 | 8 | 188.3 | 155.6 |
| Gorai.009G085000 | 670 | 74.292 | -27.5 | 4.474 | -0.05 | 2,564 | 2,013 | 43.9 | 14 | 183.1 | 495.2 |
| Gorai.009G085100 | 474 | 52.521 | -20 | 4.681 | -0.774 | 2,148 | 1,425 | 43.4 | 9 | 238.7 | 301.5 |
| Gorai.009G085200 | 167 | 19.207 | -6.5 | 5.006 | -1.213 | 877 | 504 | 44.2 | 3 | 292.3 | 102.5 |
| Gorai.009G085300 | 411 | 46.039 | -3.5 | 5.397 | -0.037 | 1,668 | 1,236 | 40.5 | 2 | 834 | 601 |
| Gorai.009G085400 | 150 | 16.117 | -4 | 4.775 | -0.08 | 1,253 | 453 | 43.5 | 2 | 626.5 | 1,339.00 |
| Gorai.009G085500 | 137 | 14.939 | 3.5 | 8.461 | -0.211 | 414 | 414 | 45.7 | 2 | 207 | 53 |
| Gorai.009G085600 | 216 | 24.561 | 21 | 10.576 | -0.38 | 961 | 651 | 42.9 | 6 | 160.2 | 208.6 |
| Gorai.009G085700 | 224 | 25.255 | -3.5 | 5.25 | -0.698 | 1,239 | 675 | 40.1 | 5 | 247.8 | 307.8 |
| Gorai.009G085800 | 216 | 24.531 | 21 | 10.576 | -0.369 | 1,068 | 651 | 42.2 | 6 | 178 | 252 |
| Gorai.009G085900 | 143 | 15.868 | 6.5 | 9.325 | -0.767 | 1,262 | 432 | 44.9 | 1 | 1,262.00 | No intron |
| Gorai.009G086000 | 592 | 67.368 | 5.5 | 7.432 | 0.396 | 2,298 | 1,779 | 41.5 | 7 | 328.3 | 436 |
| Gorai.009G086100 | 111 | 12.505 | 10 | 11.145 | -0.241 | 1,081 | 336 | 47 | 2 | 540.5 | 1,043.00 |
| Gorai.009G086200 | 509 | 57.013 | 3.5 | 6.75 | -0.541 | 2,235 | 1,530 | 43.9 | 13 | 171.9 | 330.7 |
| Gorai.009G086900 | 485 | 55.319 | 14 | 8.757 | -0.174 | 1,458 | 1,458 | 45.7 | 3 | 486 | 164.5 |
| Gorai.009G087000 | 149 | 17.094 | 0 | 6.511 | -0.075 | 450 | 450 | 43.6 | 6 | 75 | 396.8 |
| Gorai.009G087100 | 394 | 43.898 | 4.5 | 8.186 | -0.244 | 1,582 | 1,185 | 46.7 | 9 | 175.8 | 300.5 |
| Gorai.009G087200 | 2,204 | 252.863 | 11.5 | 6.829 | -0.355 | 6,832 | 6,615 | 41.1 | 49 | 139.4 | 264.9 |
| Gorai.009G092100 | 178 | 20.195 | -5 | 4.803 | -0.432 | 2,371 | 537 | 46.2 | 5 | 474.2 | 153.5 |
| Gorai.009G092200 | 451 | 49.19 | 2.5 | 7.136 | 0.527 | 1,941 | 1,356 | 44.8 | 11 | 176.5 | 190.1 |
| Gorai.009G092300 | 243 | 28.269 | 3 | 7.615 | -0.235 | 1,139 | 732 | 43.9 | 5 | 227.8 | 321.8 |
| Gorai.009G092400 | 901 | 100.725 | -18 | 5.132 | -0.387 | 3,272 | 2,706 | 41.4 | 23 | 142.3 | 404 |
| Gorai.009G098200 | 427 | 49.09 | 13.5 | 9.469 | -0.498 | 1,284 | 1,284 | 42.9 | 2 | 642 | 84 |
| Gorai.009G098300 | 701 | 77.592 | 17.5 | 8.792 | -0.657 | 3,010 | 2,106 | 43.5 | 10 | 301 | 357.4 |
| Gorai.009G098400 | 456 | 52.221 | -11.5 | 5.096 | -0.612 | 2,196 | 1,371 | 43.3 | 10 | 219.6 | 398 |
| Gorai.009G098500 | 129 | 14.654 | -1 | 6.223 | 0.426 | 1,065 | 390 | 41.8 | 4 | 266.3 | 417.7 |
| Gorai.009G098600 | 727 | 85.245 | 0 | 6.533 | -0.945 | 2,396 | 2,184 | 41.9 | 2 | 1,198.00 | 90 |
| Gorai.009G098700 | 418 | 46.246 | 22 | 9.718 | 0.283 | 1,852 | 1,257 | 44.6 | 6 | 308.7 | 420.8 |
| Gorai.009G107600 | 188 | 19.969 | -1 | 6.06 | 0.477 | 1,499 | 567 | 47.1 | 4 | 374.8 | 697.3 |
| Gorai.009G107700 | 842 | 93.93 | -69.5 | 4.123 | -0.379 | 3,435 | 2,529 | 42 | 20 | 171.8 | 184.4 |
| Gorai.009G107800 | 695 | 77.079 | 15.5 | 8.522 | -0.419 | 3,077 | 2,088 | 46.5 | 4 | 769.3 | 338.3 |
| Gorai.009G107900 | 234 | 25.748 | 23 | 10.679 | -0.657 | 2,468 | 705 | 47.2 | 3 | 822.7 | 420.5 |
| Gorai.009G108000 | 748 | 84.963 | 14 | 8.151 | -0.352 | 2,317 | 2,247 | 41.5 | 20 | 115.9 | 231.9 |
| Gorai.009G108100 | 340 | 37.168 | 9.5 | 9.17 | 0.656 | 1,821 | 1,023 | 41.2 | 8 | 227.6 | 124.7 |
| Gorai.009G108200 | 379 | 43.807 | 10 | 8.549 | -0.444 | 1,793 | 1,140 | 42.6 | 15 | 119.5 | 256.6 |
| Gorai.009G110300 | 530 | 57.267 | 14 | 8.931 | 0.337 | 2,014 | 1,593 | 48.1 | 3 | 671.3 | 90 |
| Gorai.009G110400 | 172 | 19.061 | 6.5 | 8.987 | -0.127 | 1,360 | 519 | 44.7 | 4 | 340 | 490.3 |
| Gorai.009G110500 | 168 | 18.849 | 16.5 | 10.314 | -0.077 | 883 | 507 | 44.2 | 2 | 441.5 | 23 |
| Gorai.009G110600 | 722 | 79.653 | 16 | 8.682 | 0.259 | 3,040 | 2,169 | 42.2 | 13 | 233.8 | 300.6 |
| Gorai.009G110700 | 338 | 36.029 | 16.5 | 9.381 | -0.859 | 1,769 | 1,017 | 46.6 | 5 | 353.8 | 141.8 |
| Gorai.009G112600 | 238 | 24.678 | 11 | 9.864 | -0.479 | 1,759 | 717 | 51.7 | 2 | 879.5 | 730 |
| Gorai.009G112700 | 406 | 41.409 | 14 | 8.965 | -1.062 | 2,352 | 1,221 | 48.7 | 7 | 336 | 197.2 |
| Gorai.009G112800 | 303 | 33.326 | 4.5 | 7.408 | -0.521 | 1,252 | 912 | 51 | 4 | 313 | 351 |
| Gorai.009G112900 | 158 | 17.655 | 2 | 7.772 | -0.254 | 603 | 477 | 47 | 2 | 301.5 | 172 |
| Gorai.009G113000 | 213 | 24.988 | -13 | 4.538 | -1.023 | 1,456 | 642 | 43.6 | 3 | 485.3 | 183 |
| Gorai.009G120200 | 476 | 54.224 | 10.5 | 8.984 | -0.168 | 1,599 | 1,431 | 46.3 | 3 | 533 | 481 |
| Gorai.009G120300 | 1,452 | 164.883 | 15.5 | 8.062 | 0.049 | 4,908 | 4,359 | 43.5 | 19 | 258.3 | 177.8 |
| Gorai.009G120400 | 758 | 87.647 | -2 | 6.39 | -0.36 | 2,791 | 2,277 | 45.5 | 18 | 155.1 | 273.1 |
| Gorai.009G120500 | 710 | 77.836 | 12.5 | 8.302 | -0.361 | 2,155 | 2,133 | 44.1 | 16 | 134.7 | 126.9 |
| Gorai.009G120600 | 383 | 43.645 | 23.5 | 10 | -0.326 | 1,581 | 1,152 | 42 | 7 | 225.9 | 429.8 |
| Gorai.009G124500 | 300 | 30.974 | 8 | 9.001 | 0.424 | 1,350 | 903 | 50.2 | 5 | 270 | 282.5 |
| Gorai.009G124600 | 271 | 29.556 | 8.5 | 8.485 | -0.725 | 1,214 | 816 | 46.9 | 1 | 1,214.00 | No intron |
| Gorai.009G124700 | 260 | 30.458 | 5.5 | 8.463 | -0.792 | 1,203 | 783 | 42.1 | 3 | 401 | 288.5 |
| Gorai.009G124800 | 221 | 24.418 | 8 | 8.557 | -0.453 | 1,225 | 666 | 43.2 | 3 | 408.3 | 388 |
| Gorai.009G124900 | 234 | 27.325 | -3.5 | 5.024 | -0.415 | 1,623 | 705 | 41 | 11 | 147.5 | 582.5 |
| Gorai.009G127700 | 2,354 | 275.08 | 66 | 9.029 | -0.47 | 7,604 | 7,065 | 42.9 | 23 | 330.6 | 160.8 |
| Gorai.009G127800 | 356 | 39.757 | 1.5 | 6.699 | -0.313 | 1,809 | 1,071 | 44.9 | 5 | 361.8 | 475.3 |
| Gorai.009G127900 | 489 | 53.454 | 8 | 8.109 | -0.028 | 1,676 | 1,470 | 43.9 | 1 | 1,676.00 | No intron |
| Gorai.009G128000 | 378 | 43.224 | 23.5 | 8.374 | -0.775 | 1,625 | 1,137 | 42.1 | 7 | 232.1 | 286 |
| Gorai.009G128100 | 435 | 49.251 | 11 | 8.53 | -0.396 | 1,725 | 1,308 | 45.1 | 4 | 431.3 | 151.7 |
| Gorai.009G128200 | 371 | 40.53 | -8.5 | 4.934 | 0.019 | 1,456 | 1,116 | 48.5 | 3 | 485.3 | 92 |
| Gorai.009G128300 | 291 | 32.27 | 19.5 | 10.488 | 0.247 | 1,185 | 876 | 46.3 | 3 | 395 | 175 |
| Gorai.009G128400 | 386 | 44.321 | 19 | 9.775 | -0.099 | 1,597 | 1,161 | 44.2 | 5 | 319.4 | 199 |
| Gorai.009G128500 | 206 | 22.963 | -4 | 5.142 | -0.247 | 1,112 | 621 | 46.7 | 2 | 556 | 578 |
| Gorai.009G128600 | 84 | 10.084 | 8.5 | 10.37 | -0.345 | 765 | 255 | 32.2 | 1 | 765 | No intron |
| Gorai.009G130400 | 200 | 23.192 | 1.5 | 6.742 | -0.461 | 868 | 603 | 41.8 | 7 | 124 | 432.8 |
| Gorai.009G130500 | 130 | 14.648 | 12.5 | 10.901 | -1.352 | 1,569 | 393 | 47.6 | 1 | 1,569.00 | No intron |
| Gorai.009G130600 | 180 | 20.002 | 3 | 7.767 | 0.279 | 1,215 | 543 | 43.6 | 8 | 151.9 | 367.4 |
| Gorai.009G130700 | 165 | 18.805 | 3 | 8.314 | -0.13 | 901 | 498 | 43.4 | 4 | 225.3 | 257.7 |
| Gorai.009G130800 | 207 | 24.456 | 8 | 9.412 | -0.392 | 787 | 624 | 41.7 | 4 | 196.8 | 129.7 |
| Gorai.009G130900 | 123 | 14.053 | 12.5 | 10.756 | -0.678 | 971 | 372 | 41.1 | 2 | 485.5 | 120 |
| Gorai.009G131000 | 214 | 24.189 | -2 | 5.242 | -0.282 | 645 | 645 | 41.9 | 5 | 129 | 253 |
| Gorai.009G131100 | 237 | 26.977 | 5 | 8.89 | -0.811 | 1,244 | 714 | 44.5 | 2 | 622 | 104 |
| Gorai.009G131200 | 127 | 14.597 | 12 | 11.042 | -0.484 | 855 | 384 | 50 | 2 | 427.5 | 793 |
| Gorai.009G131300 | 263 | 29.393 | -24.5 | 4.196 | -0.701 | 1,703 | 792 | 43.6 | 3 | 567.7 | 172 |
| Gorai.009G131400 | 358 | 40.429 | -2.5 | 6.144 | -0.252 | 1,370 | 1,077 | 47.3 | 1 | 1,370.00 | No intron |
| Gorai.009G131500 | 200 | 21.127 | -4.5 | 4.467 | 0.268 | 603 | 603 | 48.4 | 2 | 301.5 | 592 |
| Gorai.009G131600 | 704 | 81.89 | 22.5 | 9.265 | 0.131 | 2,295 | 2,115 | 43.8 | 3 | 765 | 364.5 |
| Gorai.009G142000 | 593 | 65.328 | 28.5 | 9.92 | -0.147 | 2,229 | 1,782 | 46.2 | 6 | 371.5 | 518.8 |
| Gorai.009G142100 | 814 | 89.786 | -9.5 | 5.28 | -0.257 | 3,373 | 2,445 | 44 | 24 | 140.5 | 308.2 |
| Gorai.009G142200 | 369 | 40.949 | 3.5 | 6.792 | -0.323 | 1,475 | 1,110 | 44.1 | 4 | 368.8 | 137 |
| Gorai.009G142300 | 448 | 50.93 | 36 | 10.761 | -0.861 | 1,688 | 1,347 | 45.8 | 4 | 422 | 119.7 |
| Gorai.009G149100 | 357 | 40.7 | 7 | 7.476 | -0.273 | 1,776 | 1,074 | 44.1 | 3 | 592 | 1,002.50 |
| Gorai.009G149200 | 427 | 48.323 | -3.5 | 5.388 | -0.428 | 1,664 | 1,284 | 42 | 12 | 138.7 | 300.8 |
| Gorai.009G149300 | 94 | 11.236 | 9 | 10.837 | -0.565 | 932 | 285 | 47 | 2 | 466 | 1,377.00 |
| Gorai.009G149400 | 374 | 41.736 | 15 | 9.282 | -0.424 | 2,295 | 1,125 | 42.8 | 1 | 2,295.00 | No intron |
| Gorai.009G149500 | 314 | 35.811 | 2 | 6.938 | -0.455 | 1,525 | 945 | 44.2 | 8 | 190.6 | 407.1 |
| Gorai.009G149600 | 669 | 74.118 | -8.5 | 5.861 | -0.477 | 2,700 | 2,010 | 41.1 | 22 | 122.7 | 262.8 |
| Gorai.009G166800 | 999 | 113.618 | 0.5 | 6.549 | -0.22 | 3,251 | 3,000 | 42.6 | 22 | 147.8 | 253.1 |
| Gorai.009G166900 | 155 | 18.191 | 10 | 10.114 | -0.27 | 627 | 468 | 48.1 | 1 | 627 | No intron |
| Gorai.009G167000 | 214 | 25.193 | -8.5 | 4.714 | -1.334 | 1,269 | 645 | 47.3 | 2 | 634.5 | 69 |
| Gorai.009G167100 | 218 | 24.302 | 2 | 7.339 | 0.079 | 1,497 | 657 | 42.2 | 3 | 499 | 409.5 |
| Gorai.009G167200 | 161 | 18.111 | 3.5 | 9.631 | -0.619 | 1,119 | 486 | 48.6 | 2 | 559.5 | 610 |
| Gorai.009G167300 | 607 | 67.515 | 19.5 | 9.288 | -0.217 | 2,084 | 1,824 | 45.2 | 7 | 297.7 | 345.8 |
| Gorai.009G167400 | 308 | 33.113 | 12.5 | 9.541 | 0.146 | 1,493 | 927 | 43.5 | 8 | 186.6 | 284.1 |
| Gorai.009G167500 | 123 | 13.6 | 5.5 | 9.989 | -0.743 | 1,200 | 372 | 52.4 | 3 | 400 | 175 |
| Gorai.009G167600 | 774 | 85.51 | 1 | 6.591 | -0.801 | 2,852 | 2,325 | 41.6 | 6 | 475.3 | 390.4 |
| Gorai.009G176800 | 143 | 16.022 | 7 | 10.106 | -0.813 | 1,452 | 432 | 41.4 | 5 | 290.4 | 236.5 |
| Gorai.009G176900 | 475 | 52.935 | 18 | 8.753 | -0.221 | 1,854 | 1,428 | 43.1 | 10 | 185.4 | 240.2 |
| Gorai.009G177000 | 1,328 | 147.277 | -1.5 | 6.438 | -0.15 | 4,116 | 3,987 | 44.1 | 18 | 228.7 | 159.2 |
| Gorai.009G177100 | 292 | 33.833 | 9 | 9.041 | 0.638 | 1,522 | 879 | 42 | 11 | 138.4 | 229.8 |
| Gorai.009G177200 | 243 | 28.019 | 7.5 | 9.346 | 0.479 | 1,413 | 732 | 42.6 | 6 | 235.5 | 352.6 |
| Gorai.009G177300 | 164 | 18.436 | 7 | 8.407 | -0.144 | 609 | 495 | 40.8 | 2 | 304.5 | 188 |
| Gorai.009G177400 | 132 | 15.563 | 14 | 10.395 | -0.891 | 1,045 | 399 | 42.4 | 3 | 348.3 | 196 |
| Gorai.009G177500 | 239 | 27.1 | 5.5 | 8.197 | -0.297 | 1,134 | 720 | 41.5 | 1 | 1,134.00 | No intron |
| Gorai.009G177600 | 139 | 15.79 | -3.5 | 4.971 | -0.382 | 855 | 420 | 46 | 2 | 427.5 | 125 |
| Gorai.009G222500 | 237 | 28.452 | 11.5 | 10.051 | -1.376 | 1,583 | 714 | 46.1 | 7 | 226.1 | 339.5 |
| Gorai.009G222600 | 260 | 28.591 | 15 | 10.09 | -0.117 | 1,376 | 783 | 46.5 | 6 | 229.3 | 261.4 |
| Gorai.009G222700 | 468 | 52.994 | 12 | 8.832 | -0.285 | 1,407 | 1,407 | 44.8 | 4 | 351.8 | 130.3 |
| Gorai.009G222800 | 734 | 84.307 | 31 | 9.457 | 0.234 | 3,428 | 2,205 | 41 | 12 | 285.7 | 292.5 |
| Gorai.009G222900 | 356 | 39.437 | 3 | 7.19 | -0.625 | 1,401 | 1,071 | 46.7 | 3 | 467 | 128 |
| Gorai.009G229300 | 694 | 77.182 | -22 | 4.771 | -0.292 | 2,655 | 2,085 | 45.2 | 18 | 147.5 | 164.6 |
| Gorai.009G229400 | 1,265 | 141.538 | 4.5 | 6.815 | -0.789 | 4,292 | 3,798 | 42.1 | 27 | 159 | 163 |
| Gorai.009G229500 | 518 | 58.518 | 17 | 9.061 | -0.192 | 2,136 | 1,557 | 43.5 | 11 | 194.2 | 165.6 |
| Gorai.009G229600 | 1,018 | 113.458 | -88.5 | 4.111 | -0.45 | 3,844 | 3,057 | 41.1 | 16 | 240.3 | 376.1 |
| Gorai.009G229700 | 74 | 8.52 | 3.5 | 8.643 | -0.491 | 579 | 225 | 46.2 | 2 | 289.5 | 1,349.00 |
| Gorai.009G229800 | 344 | 38.061 | 8.5 | 8.713 | -0.556 | 1,680 | 1,035 | 50 | 2 | 840 | 83 |
| Gorai.009G251100 | 520 | 59.303 | 15.5 | 8.624 | -0.404 | 1,920 | 1,563 | 41 | 9 | 213.3 | 274.5 |
| Gorai.009G251200 | 451 | 50.402 | 3 | 6.823 | -0.281 | 2,814 | 1,356 | 45.4 | 9 | 312.7 | 290.3 |
| Gorai.009G251300 | 148 | 17.126 | 2.5 | 7.459 | -0.141 | 2,308 | 447 | 43 | 5 | 461.6 | 207.5 |
| Gorai.009G251400 | 372 | 40.967 | 8 | 7.687 | 0.104 | 1,794 | 1,119 | 45 | 6 | 299 | 556.8 |
| Gorai.009G251500 | 305 | 34.546 | 4 | 8.053 | 0.859 | 964 | 918 | 42.9 | 2 | 482 | 64 |
| Gorai.009G251600 | 127 | 13.478 | 8 | 9.605 | 0.141 | 672 | 384 | 48.4 | 2 | 336 | 95 |
| Gorai.009G251700 | 339 | 38.267 | 13.5 | 9.637 | -0.799 | 1,789 | 1,020 | 40.3 | 2 | 894.5 | 539 |
| Gorai.009G262100 | 1,036 | 116.843 | 8.5 | 7.201 | -0.408 | 3,469 | 3,111 | 46.4 | 13 | 266.8 | 271.1 |
| Gorai.009G262200 | 316 | 35.675 | 19.5 | 10.031 | -0.177 | 1,369 | 951 | 43 | 9 | 152.1 | 887 |
| Gorai.009G262300 | 178 | 19.2 | 10.5 | 10.489 | 0.838 | 1,163 | 537 | 47.5 | 5 | 232.6 | 411.5 |
| Gorai.009G262400 | 99 | 11.051 | 5.5 | 9.005 | -0.241 | 300 | 300 | 38.7 | 2 | 150 | 104 |
| Gorai.009G262500 | 790 | 89.919 | -4.5 | 6.229 | -0.136 | 2,813 | 2,373 | 39.7 | 3 | 937.7 | 1,605.50 |
| Gorai.009G262600 | 413 | 46.329 | 6 | 7.113 | -0.38 | 2,126 | 1,242 | 41.5 | 10 | 212.6 | 720.3 |
| Gorai.009G262700 | 601 | 65.971 | 10 | 8.361 | 0.317 | 1,859 | 1,806 | 45.9 | 4 | 464.8 | 88 |
| Gorai.009G272800 | 346 | 39.2 | 7 | 8.84 | -0.047 | 1,041 | 1,041 | 39.6 | 5 | 208.2 | 417.3 |
| Gorai.009G272900 | 519 | 57.824 | 31.5 | 10.214 | -0.287 | 2,134 | 1,560 | 41.7 | 5 | 426.8 | 128.3 |
| Gorai.009G273000 | 86 | 9.801 | 0 | 6.526 | -1.06 | 839 | 261 | 45.2 | 2 | 419.5 | 1,164.00 |
| Gorai.009G304600 | 420 | 48.58 | -1 | 6.347 | -0.978 | 1,846 | 1,263 | 46.5 | 2 | 923 | 673 |
| Gorai.009G304700 | 227 | 25.58 | 6.5 | 8.016 | -0.199 | 1,465 | 684 | 44.9 | 3 | 488.3 | 2,371.50 |
| Gorai.009G307700 | 118 | 12.932 | 5.5 | 9.09 | -0.423 | 920 | 357 | 38.4 | 1 | 920 | No intron |
| Gorai.009G308500 | 334 | 35.924 | 3 | 7.273 | -0.254 | 1,583 | 1,005 | 43.3 | 9 | 175.9 | 651.5 |
| Gorai.009G308600 | 79 | 8.716 | 2.5 | 9.697 | 0.853 | 270 | 237 | 42.2 | 1 | 270 | No intron |
| Gorai.009G308700 | 209 | 17.272 | 4 | 9.282 | -0.482 | 630 | 630 | 54.4 | 1 | 630 | No intron |
| Gorai.009G308800 | 79 | 9.638 | -0.5 | 5.85 | -0.71 | 237 | 237 | 36.3 | 1 | 237 | No intron |
| Gorai.009G308900 | 520 | 57.304 | -7.5 | 5.615 | -0.276 | 2,003 | 1,563 | 43.5 | 14 | 143.1 | 242.4 |
| Gorai.009G310400 | 854 | 93.51 | 15.5 | 8.934 | -0.259 | 2,565 | 2,565 | 44.4 | 3 | 855 | 320.5 |
| Gorai.009G310500 | 255 | 27.682 | 2.5 | 8.035 | -0.922 | 1,119 | 768 | 53.6 | 2 | 559.5 | 34 |
| Gorai.009G310600 | 209 | 22.734 | -14.5 | 4.07 | 0.023 | 1,096 | 630 | 41.9 | 8 | 137 | 372 |
| Gorai.009G311400 | 536 | 60.138 | 15 | 9.17 | 0.21 | 1,804 | 1,611 | 42.8 | 3 | 601.3 | 335.5 |
| Gorai.009G311500 | 553 | 60.085 | -4 | 5.805 | 0.316 | 2,309 | 1,662 | 41.9 | 14 | 164.9 | 458.9 |
| Gorai.009G313800 | 1,011 | 111.206 | 4 | 6.728 | -0.035 | 3,154 | 3,036 | 41.3 | 2 | 1,577.00 | 156 |
| Gorai.009G315600 | 179 | 19.3 | -1.5 | 5.731 | -0.652 | 1,459 | 540 | 45.2 | 7 | 208.4 | 913.3 |
| Gorai.009G315700 | 381 | 42.458 | 9 | 9.008 | -0.303 | 1,585 | 1,146 | 41.9 | 12 | 132.1 | 279.7 |
| Gorai.009G315800 | 227 | 25.409 | 8.5 | 9.936 | -0.3 | 1,019 | 684 | 48.7 | 2 | 509.5 | 103 |
| Gorai.009G318900 | 244 | 26.74 | -1 | 6.139 | -0.03 | 1,646 | 735 | 49.1 | 3 | 548.7 | 767.5 |
| Gorai.009G319000 | 505 | 57.608 | 1 | 6.614 | -0.512 | 2,330 | 1,518 | 44.4 | 2 | 1,165.00 | 426 |
| Gorai.009G319100 | 161 | 18.869 | 1 | 6.93 | -0.035 | 486 | 486 | 38.1 | 2 | 243 | 89 |
| Gorai.009G327300 | 328 | 36.547 | -2.5 | 5.944 | -0.936 | 1,471 | 987 | 44.6 | 7 | 210.1 | 257.2 |
| Gorai.009G328000 | 82 | 9.806 | 9 | 10.157 | -0.751 | 535 | 249 | 33.7 | 2 | 267.5 | 346 |
| Gorai.009G328100 | 264 | 29.024 | 11 | 8.146 | 0.453 | 1,144 | 795 | 47 | 5 | 228.8 | 181.3 |
| Gorai.009G328200 | 568 | 64.197 | 4 | 6.942 | -0.659 | 2,148 | 1,707 | 42.1 | 14 | 153.4 | 264.8 |
| Gorai.009G330400 | 474 | 54.535 | -6 | 5.283 | -0.216 | 1,843 | 1,425 | 40.7 | 6 | 307.2 | 82.8 |
| Gorai.009G403000 | 174 | 19.471 | -10.5 | 4.774 | -0.97 | 862 | 525 | 47.4 | 2 | 431 | 332 |
| Gorai.009G415100 | 70 | 7.912 | 10.5 | 10.493 | -0.474 | 213 | 213 | 42.3 | 2 | 106.5 | 237 |
| Gorai.010G008200 | 342 | 37.905 | 16 | 9.343 | -0.65 | 1,574 | 1,029 | 40.3 | 7 | 224.9 | 392.3 |
| Gorai.010G008300 | 430 | 48.333 | 17 | 8.894 | -0.167 | 1,496 | 1,293 | 44 | 10 | 149.6 | 168.7 |
| Gorai.010G008400 | 443 | 50.154 | 5.5 | 7.474 | -0.202 | 2,985 | 1,332 | 39.4 | 10 | 298.5 | 402.8 |
| Gorai.010G016000 | 519 | 57.459 | 15.5 | 9.327 | 0.471 | 1,850 | 1,560 | 43.7 | 5 | 370 | 479.8 |
| Gorai.010G016100 | 366 | 40.168 | -2.5 | 5.742 | -0.138 | 2,078 | 1,101 | 47.1 | 6 | 346.3 | 196 |
| Gorai.010G016200 | 550 | 60.06 | 18 | 9.439 | 0.442 | 2,248 | 1,653 | 46.4 | 15 | 149.9 | 154 |
| Gorai.010G016300 | 123 | 13.298 | 0.5 | 6.764 | -0.262 | 700 | 372 | 51.3 | 1 | 700 | No intron |
| Gorai.010G016400 | 182 | 20.372 | 4 | 9.784 | -0.346 | 1,382 | 549 | 44.4 | 1 | 1,382.00 | No intron |
| Gorai.010G016500 | 129 | 14.329 | 1.5 | 8.495 | -0.178 | 569 | 390 | 45.9 | 1 | 569 | No intron |
| Gorai.010G016600 | 159 | 17.761 | -0.5 | 5.897 | -0.231 | 808 | 480 | 37.7 | 2 | 404 | 140 |
| Gorai.010G016700 | 139 | 15.707 | 1 | 7.403 | 0.063 | 722 | 420 | 44.5 | 2 | 361 | 160 |
| Gorai.010G016800 | 198 | 21.965 | 6 | 9.194 | -0.697 | 1,060 | 597 | 48.6 | 2 | 530 | 353 |
| Gorai.010G016900 | 718 | 80.309 | -2 | 6.165 | -0.298 | 2,433 | 2,157 | 43.4 | 18 | 135.2 | 230.8 |
| Gorai.010G033400 | 360 | 39.489 | -19.5 | 4.46 | -0.711 | 1,749 | 1,083 | 45.7 | 11 | 159 | 288.3 |
| Gorai.010G033500 | 296 | 31.806 | -3.5 | 5.812 | -0.545 | 1,524 | 891 | 49.4 | 7 | 217.7 | 475.7 |
| Gorai.010G033600 | 483 | 54.045 | -6 | 5.503 | -0.1 | 1,822 | 1,452 | 44.1 | 1 | 1,822.00 | No intron |
| Gorai.010G033700 | 2,826 | 318.34 | 3.5 | 6.602 | -0.071 | 8,995 | 8,481 | 39.3 | 33 | 272.6 | 359.4 |
| Gorai.010G035900 | 382 | 41.912 | -8.5 | 4.978 | -0.42 | 1,146 | 1,146 | 43.3 | 4 | 286.5 | 123 |
| Gorai.010G036000 | 765 | 85.016 | 2.5 | 6.634 | -0.504 | 3,047 | 2,298 | 43.4 | 20 | 152.4 | 492.9 |
| Gorai.010G048100 | 88 | 9.373 | 3.5 | 10.4 | 0.056 | 766 | 267 | 49.1 | 4 | 191.5 | 566 |
| Gorai.010G048200 | 470 | 53.347 | 13.5 | 8.291 | 0.509 | 1,871 | 1,413 | 40.8 | 7 | 267.3 | 92.8 |
| Gorai.010G048300 | 173 | 18.257 | 2.5 | 7.791 | -0.802 | 522 | 522 | 51.9 | 1 | 522 | No intron |
| Gorai.010G058600 | 221 | 25.253 | 4.5 | 8.863 | -0.204 | 938 | 666 | 44.1 | 4 | 234.5 | 105.3 |
| Gorai.010G060300 | 774 | 85.023 | 16.5 | 8.333 | 0.501 | 2,949 | 2,325 | 43.6 | 7 | 421.3 | 247.8 |
| Gorai.010G060400 | 505 | 56.263 | 10.5 | 8.205 | -0.257 | 2,447 | 1,518 | 42.8 | 8 | 305.9 | 278 |
| Gorai.010G060500 | 360 | 41.097 | 8.5 | 8.221 | 0.251 | 1,546 | 1,083 | 45 | 4 | 386.5 | 290 |
| Gorai.010G063100 | 191 | 21.162 | 3.5 | 8.204 | 0.802 | 576 | 576 | 45.3 | 4 | 144 | 151 |
| Gorai.010G063200 | 288 | 31.376 | -20.5 | 4.202 | -0.574 | 1,270 | 867 | 47.4 | 4 | 317.5 | 73.3 |
| Gorai.010G064400 | 176 | 19.797 | 2.5 | 8.432 | 0.231 | 832 | 531 | 42 | 9 | 92.4 | 331.5 |
| Gorai.010G064500 | 130 | 14.464 | -1.5 | 5.293 | -0.301 | 1,078 | 393 | 44 | 5 | 215.6 | 264 |
| Gorai.010G064600 | 396 | 45.971 | 2.5 | 6.849 | -0.353 | 1,448 | 1,191 | 41.4 | 11 | 131.6 | 363 |
| Gorai.010G064700 | 428 | 48.504 | 28 | 9.713 | -0.298 | 1,960 | 1,287 | 45.6 | 3 | 653.3 | 931 |
| Gorai.010G072400 | 284 | 33.535 | 20 | 10.205 | -0.309 | 855 | 855 | 29.7 | 7 | 122.1 | 801.3 |
| Gorai.010G072500 | 139 | 15.669 | 13.5 | 10.161 | -0.413 | 451 | 420 | 42.9 | 2 | 225.5 | 509 |
| Gorai.010G072600 | 93 | 10.234 | -2 | 4.99 | 0.017 | 852 | 282 | 41.1 | 2 | 426 | 141 |
| Gorai.010G072700 | 881 | 98.923 | 14.5 | 8.397 | -0.746 | 3,544 | 2,646 | 41.2 | 5 | 708.8 | 344.5 |
| Gorai.010G072800 | 439 | 49.702 | -9 | 5.067 | -0.551 | 1,690 | 1,320 | 42.9 | 6 | 281.7 | 89.4 |
| Gorai.010G072900 | 405 | 44.249 | 8 | 8.341 | -0.086 | 1,891 | 1,218 | 44.1 | 12 | 157.6 | 226.1 |
| Gorai.010G073400 | 409 | 45.456 | 4 | 7.896 | -0.259 | 2,746 | 1,230 | 43.3 | 4 | 686.5 | 187.3 |
| Gorai.010G073500 | 112 | 13.202 | -2 | 5.495 | 0.418 | 1,338 | 339 | 33.9 | 3 | 446 | 878 |
| Gorai.010G073600 | 353 | 38.885 | 4 | 7.534 | -0.729 | 1,721 | 1,062 | 46.5 | 9 | 191.2 | 180.9 |
| Gorai.010G074600 | 1,101 | 121.808 | -28 | 4.971 | -0.199 | 3,859 | 3,306 | 44.2 | 16 | 241.2 | 225.5 |
| Gorai.010G074700 | 88 | 10.169 | 0 | 6.491 | 0.04 | 1,152 | 267 | 42.7 | 5 | 230.4 | 250.3 |
| Gorai.010G074800 | 414 | 47.424 | 8 | 7.736 | -0.376 | 1,795 | 1,245 | 42.2 | 1 | 1,795.00 | No intron |
| Gorai.010G074900 | 463 | 49.445 | -5 | 5.968 | -0.642 | 1,946 | 1,392 | 44.1 | 4 | 486.5 | 347.7 |
| Gorai.010G075000 | 189 | 21.95 | 6.5 | 8.484 | -0.394 | 570 | 570 | 42.8 | 1 | 570 | No intron |
| Gorai.010G075100 | 513 | 56.661 | 12 | 8.944 | 0.426 | 2,053 | 1,542 | 45.1 | 4 | 513.3 | 193 |
| Gorai.010G075200 | 765 | 84.99 | 30.5 | 9.879 | -0.718 | 2,635 | 2,298 | 46.1 | 5 | 527 | 127 |
| Gorai.010G078600 | 278 | 31.074 | -3 | 5.387 | -0.237 | 1,160 | 837 | 49.3 | 4 | 290 | 398.3 |
| Gorai.010G078700 | 405 | 42.873 | -4.5 | 5.061 | 0.239 | 1,435 | 1,218 | 45.9 | 1 | 1,435.00 | No intron |
| Gorai.010G078800 | 283 | 31.78 | 9.5 | 8.885 | -0.066 | 1,364 | 852 | 46.4 | 6 | 227.3 | 159.6 |
| Gorai.010G078900 | 476 | 53.857 | 6.5 | 6.831 | -0.977 | 2,377 | 1,431 | 47.5 | 7 | 339.6 | 435.5 |
| Gorai.010G080400 | 569 | 63.711 | -0.5 | 6.454 | -0.225 | 2,118 | 1,710 | 47 | 6 | 353 | 141.6 |
| Gorai.010G080500 | 121 | 14.022 | 11.5 | 10.576 | -0.888 | 1,001 | 366 | 47 | 3 | 333.7 | 391 |
| Gorai.010G080600 | 127 | 13.78 | -3 | 5.008 | 0.022 | 553 | 384 | 45.8 | 3 | 184.3 | 84.5 |
| Gorai.010G082500 | 413 | 47.666 | 18.5 | 9.622 | -0.129 | 1,721 | 1,242 | 40.9 | 13 | 132.4 | 179.3 |
| Gorai.010G082600 | 686 | 76.897 | 30.5 | 9.903 | -0.779 | 2,869 | 2,061 | 39.9 | 14 | 204.9 | 236.8 |
| Gorai.010G148000 | 159 | 18.074 | 7.5 | 8.942 | -0.254 | 818 | 480 | 45 | 7 | 116.9 | 588.7 |
| Gorai.010G148100 | 189 | 20.885 | -19.5 | 4.166 | -0.934 | 570 | 570 | 44.7 | 3 | 190 | 243 |
| Gorai.010G148200 | 154 | 17.715 | 12.5 | 10.306 | -0.264 | 465 | 465 | 39.8 | 4 | 116.3 | 846.3 |
| Gorai.010G164300 | 508 | 56.738 | -29.5 | 4.313 | -0.169 | 2,546 | 1,527 | 40 | 8 | 318.3 | 735.3 |
| Gorai.010G164400 | 188 | 20.853 | 10.5 | 10.085 | -0.093 | 1,118 | 567 | 42.2 | 7 | 159.7 | 318.7 |
| Gorai.010G164500 | 370 | 41.506 | 19.5 | 9.812 | -0.039 | 1,113 | 1,113 | 50.8 | 1 | 1,113.00 | No intron |
| Gorai.010G183700 | 183 | 20.551 | 21.5 | 11.406 | -0.483 | 552 | 552 | 44.2 | 5 | 110.4 | 866.8 |
| Gorai.010G183800 | 355 | 39.952 | 2 | 6.689 | -0.73 | 2,608 | 1,068 | 46.8 | 5 | 521.6 | 606.8 |
| Gorai.010G218400 | 983 | 106.751 | -6.5 | 5.724 | 0.068 | 3,732 | 2,952 | 42.8 | 2 | 1,866.00 | 287 |
| Gorai.010G218500 | 494 | 55.662 | 11.5 | 9.217 | -0.036 | 1,552 | 1,485 | 43 | 4 | 388 | 784 |
| Gorai.010G218600 | 191 | 20.782 | 3.5 | 7.376 | 0.103 | 576 | 576 | 51.7 | 1 | 576 | No intron |
| Gorai.010G231600 | 150 | 16.765 | 28.5 | 11.617 | -0.701 | 852 | 453 | 47 | 4 | 213 | 531 |
| Gorai.010G231700 | 306 | 34.508 | -38.5 | 4.147 | -1.261 | 1,211 | 921 | 46 | 2 | 605.5 | 979 |
| Gorai.010G231800 | 462 | 51.07 | -3 | 6.278 | -0.448 | 1,805 | 1,389 | 45.9 | 15 | 120.3 | 351.9 |
| Gorai.010G231900 | 635 | 73.371 | 9.5 | 8.335 | 0.204 | 2,964 | 1,908 | 41.9 | 7 | 423.4 | 454.7 |
| Gorai.010G232000 | 64 | 6.263 | 7 | 11.531 | 0.661 | 195 | 195 | 51.3 | 1 | 195 | No intron |
| Gorai.010G232100 | 170 | 19.408 | 4.5 | 7.992 | -0.449 | 513 | 513 | 42.7 | 1 | 513 | No intron |
| Gorai.010G232200 | 155 | 17.822 | 6 | 8.154 | -0.556 | 468 | 468 | 45.1 | 1 | 468 | No intron |
| Gorai.010G232300 | 254 | 27.459 | 29.5 | 10.844 | -0.073 | 765 | 765 | 48.5 | 1 | 765 | No intron |
| Gorai.010G250000 | 551 | 64.217 | 0 | 6.523 | -0.748 | 2,252 | 1,656 | 41.8 | 10 | 225.2 | 209 |
| Gorai.010G250100 | 106 | 11.511 | 13.5 | 9.079 | 0 | 536 | 321 | 47.7 | 1 | 536 | No intron |
| Gorai.010G250200 | 214 | 24.445 | -3 | 5.432 | -0.527 | 1,034 | 645 | 51 | 1 | 1,034.00 | No intron |
| Gorai.010G250300 | 420 | 45.827 | -1.5 | 6.285 | -0.184 | 1,791 | 1,263 | 44.7 | 14 | 127.9 | 255.6 |
| Gorai.010G250400 | 170 | 18.672 | 10.5 | 10.198 | -0.578 | 513 | 513 | 41.9 | 1 | 513 | No intron |
| Gorai.010G250500 | 423 | 48.806 | 15.5 | 9.605 | 0.291 | 1,890 | 1,272 | 40.2 | 12 | 157.5 | 297.5 |
| Gorai.010G250600 | 255 | 29.528 | 14 | 9.337 | 0.291 | 2,533 | 768 | 43 | 3 | 844.3 | 210.5 |
| Gorai.010G250700 | 307 | 34.54 | 3 | 7.5 | -0.621 | 1,081 | 924 | 46.2 | 2 | 540.5 | 632 |
| Gorai.010G250800 | 216 | 23.865 | -7 | 4.915 | -1.256 | 1,201 | 651 | 45.6 | 3 | 400.3 | 730 |
| Gorai.010G250900 | 80 | 9.076 | 3.5 | 8.909 | -0.904 | 277 | 243 | 39.9 | 3 | 92.3 | 748 |
| Gorai.010G251900 | 404 | 44.376 | -1 | 6.177 | -0.166 | 1,215 | 1,215 | 44 | 2 | 607.5 | 52 |
| Gorai.010G252000 | 379 | 40.88 | 6 | 9.551 | -0.066 | 1,140 | 1,140 | 45.4 | 3 | 380 | 121.5 |
| Gorai.010G252100 | 446 | 50.315 | 11.5 | 8.434 | -0.323 | 1,781 | 1,341 | 41.6 | 3 | 593.7 | 274.5 |
| Gorai.010G252200 | 290 | 32.566 | -4.5 | 5.265 | -0.33 | 1,552 | 873 | 44.4 | 9 | 172.4 | 112.4 |
| Gorai.010G252300 | 73 | 8.35 | 2.5 | 8.524 | -0.922 | 832 | 222 | 45 | 3 | 277.3 | 79.5 |
| Gorai.010G252400 | 527 | 59.318 | -10 | 5.232 | -0.432 | 1,793 | 1,584 | 41.9 | 8 | 224.1 | 81.7 |
| Gorai.010G252500 | 125 | 14.525 | -6.5 | 4.601 | -0.623 | 705 | 378 | 42.1 | 4 | 176.3 | 578.7 |
| Gorai.010G252600 | 121 | 13.446 | -5 | 4.666 | -0.774 | 679 | 366 | 47.5 | 1 | 679 | No intron |
| Gorai.010G252700 | 506 | 57.387 | 11.5 | 8.61 | -0.077 | 1,729 | 1,521 | 46.9 | 1 | 1,729.00 | No intron |
| Gorai.010G253100 | 208 | 23.054 | 5 | 9.547 | -0.431 | 1,232 | 627 | 39.9 | 5 | 246.4 | 272 |
| Gorai.010G253200 | 240 | 27.714 | 15.5 | 10.14 | -0.246 | 1,028 | 723 | 45.9 | 1 | 1,028.00 | No intron |
| Gorai.010G253300 | 197 | 22.481 | 20 | 10.65 | -0.016 | 1,065 | 594 | 45.3 | 1 | 1,065.00 | No intron |
| Gorai.010G253400 | 671 | 75.03 | 6 | 7.666 | -0.149 | 2,318 | 2,016 | 41.7 | 7 | 331.1 | 223.8 |
| Gorai.010G253500 | 617 | 69.417 | -3.5 | 5.915 | -0.209 | 1,854 | 1,854 | 39.9 | 6 | 309 | 425.8 |
| Gorai.010G253600 | 1,162 | 128.101 | -9.5 | 5.947 | -0.857 | 3,927 | 3,489 | 43.9 | 3 | 1,309.00 | 512 |
| Gorai.010G256400 | 300 | 32.542 | 9 | 10.083 | -0.25 | 1,340 | 903 | 46.4 | 3 | 446.7 | 1,217.50 |
| Gorai.010G256500 | 775 | 84.896 | -0.5 | 6.484 | -0.778 | 2,932 | 2,328 | 41.3 | 5 | 586.4 | 238.3 |
| Gorai.010G256600 | 228 | 24.768 | 6.5 | 10.025 | -0.486 | 687 | 687 | 49.5 | 1 | 687 | No intron |
| Gorai.010G256700 | 607 | 67.835 | 13 | 8.836 | -0.307 | 2,285 | 1,824 | 45.1 | 7 | 326.4 | 203.5 |
| Gorai.010G256800 | 158 | 17.718 | 1.5 | 7.613 | -0.456 | 1,351 | 477 | 44 | 2 | 675.5 | 1,297.00 |
| Gorai.010G256900 | 1,747 | 197.744 | -42.5 | 5.07 | -0.877 | 5,548 | 5,244 | 43.2 | 19 | 292 | 205.7 |
| Gorai.011G000700 | 421 | 45.16 | 12.5 | 9.108 | -0.433 | 1,263 | 1,263 | 42.4 | 5 | 252.6 | 259 |
| Gorai.011G000800 | 705 | 77.871 | -6.5 | 5.469 | -0.112 | 2,563 | 2,118 | 45.5 | 19 | 134.9 | 373.8 |
| Gorai.011G000900 | 955 | 105.257 | 16.5 | 7.831 | 0.015 | 3,214 | 2,868 | 43 | 2 | 1,607.00 | 80 |
| Gorai.011G001000 | 488 | 54.578 | -1.5 | 6.333 | -0.463 | 1,979 | 1,467 | 42.9 | 6 | 329.8 | 311.8 |
| Gorai.011G001100 | 777 | 88.208 | -15 | 5.37 | -0.242 | 2,845 | 2,334 | 41 | 21 | 135.5 | 1,026.30 |
| Gorai.011G001500 | 433 | 48.099 | -4 | 6.041 | -0.771 | 1,957 | 1,302 | 43 | 8 | 244.6 | 489.1 |
| Gorai.011G001600 | 206 | 22.903 | -5.5 | 4.586 | 0.327 | 875 | 621 | 44.1 | 3 | 291.7 | 194.5 |
| Gorai.011G001700 | 648 | 71.417 | -8 | 5.276 | -0.756 | 2,593 | 1,947 | 44.3 | 8 | 324.1 | 521.3 |
| Gorai.011G001800 | 721 | 80.095 | 2 | 6.78 | -0.314 | 2,166 | 2,166 | 43.5 | 9 | 240.7 | 200.9 |
| Gorai.011G001900 | 639 | 71.308 | 5 | 7.257 | -0.222 | 1,920 | 1,920 | 38.5 | 3 | 640 | 281.5 |
| Gorai.011G002000 | 802 | 84.958 | -3 | 5.935 | 0.585 | 3,200 | 2,409 | 44 | 17 | 188.2 | 375.6 |
| Gorai.011G002100 | 373 | 43.084 | 7 | 7.651 | -0.173 | 1,505 | 1,122 | 39.8 | 2 | 752.5 | 1,564.00 |
| Gorai.011G002200 | 176 | 19.746 | 6.5 | 9.057 | -0.361 | 698 | 531 | 43.7 | 1 | 698 | No intron |
| Gorai.011G008100 | 769 | 84.909 | -18.5 | 4.944 | -0.261 | 2,803 | 2,310 | 44.1 | 7 | 400.4 | 435.8 |
| Gorai.011G008200 | 1,118 | 123.508 | -16.5 | 5.253 | -0.015 | 3,669 | 3,357 | 40.4 | 2 | 1,834.50 | 540 |
| Gorai.011G008300 | 330 | 36.282 | 2 | 6.786 | -0.251 | 3,152 | 993 | 45.4 | 8 | 394 | 316 |
| Gorai.011G008400 | 547 | 61.725 | -15.5 | 4.912 | -0.859 | 1,977 | 1,644 | 48.4 | 3 | 659 | 660 |
| Gorai.011G008500 | 239 | 27.401 | 11.5 | 9.707 | -0.688 | 1,326 | 720 | 46 | 5 | 265.2 | 326.8 |
| Gorai.011G008600 | 252 | 27.651 | 13 | 9.284 | -1.109 | 1,280 | 759 | 50.5 | 3 | 426.7 | 141.5 |
| Gorai.011G008700 | 759 | 83.148 | 9 | 7.303 | -0.722 | 2,860 | 2,280 | 46.9 | 11 | 260 | 294.1 |
| Gorai.011G008800 | 336 | 37.464 | 10 | 9.453 | -0.764 | 1,553 | 1,011 | 46.5 | 5 | 310.6 | 315.8 |
| Gorai.011G013600 | 418 | 47.545 | 13 | 8.525 | -0.4 | 2,071 | 1,257 | 41.3 | 11 | 188.3 | 233.6 |
| Gorai.011G013700 | 155 | 17.713 | -2 | 5.682 | -0.858 | 845 | 468 | 37.2 | 2 | 422.5 | 136 |
| Gorai.011G013800 | 1,153 | 131.428 | -1 | 6.475 | -0.48 | 4,110 | 3,462 | 40 | 16 | 256.9 | 156.3 |
| Gorai.011G013900 | 129 | 14.203 | 3 | 8.481 | 0.181 | 661 | 390 | 41 | 3 | 220.3 | 405.5 |
| Gorai.011G014000 | 1,469 | 162.828 | -32.5 | 5.125 | -0.75 | 4,410 | 4,410 | 42.2 | 8 | 551.3 | 219.6 |
| Gorai.011G015800 | 388 | 43.139 | 1 | 6.715 | -0.329 | 1,847 | 1,167 | 43.4 | 5 | 369.4 | 276.5 |
| Gorai.011G015900 | 1,101 | 122.771 | -5 | 6.16 | -0.131 | 3,511 | 3,306 | 42.4 | 18 | 195.1 | 127.4 |
| Gorai.011G016000 | 506 | 57.451 | 9.5 | 8.395 | -0.036 | 1,521 | 1,521 | 47.6 | 1 | 1,521.00 | No intron |
| Gorai.011G016100 | 724 | 82.574 | -5.5 | 6.068 | -0.31 | 2,682 | 2,175 | 42.3 | 20 | 134.1 | 311.9 |
| Gorai.011G016200 | 734 | 80.762 | -4.5 | 5.893 | -0.358 | 3,149 | 2,205 | 44.7 | 11 | 286.3 | 137 |
| Gorai.011G016300 | 129 | 14.331 | 8 | 10.242 | -0.656 | 958 | 390 | 41.8 | 5 | 191.6 | 319.5 |
| Gorai.011G016400 | 124 | 13.962 | -4.5 | 4.722 | -0.149 | 663 | 375 | 47.7 | 2 | 331.5 | 807 |
| Gorai.011G016500 | 300 | 34.878 | -3 | 5.676 | -0.557 | 1,257 | 903 | 42.3 | 4 | 314.3 | 357.3 |
| Gorai.011G016600 | 305 | 34.192 | 2 | 7.816 | -0.434 | 1,570 | 918 | 50.9 | 1 | 1,570.00 | No intron |
| Gorai.011G022100 | 330 | 37.622 | 0.5 | 6.702 | -0.477 | 1,295 | 993 | 48.6 | 3 | 431.7 | 390.5 |
| Gorai.011G022200 | 209 | 23.353 | -4 | 5.132 | -0.158 | 1,298 | 630 | 46.8 | 2 | 649 | 276 |
| Gorai.011G022300 | 307 | 34.992 | -2 | 6.025 | -0.516 | 924 | 924 | 43 | 1 | 924 | No intron |
| Gorai.011G022400 | 888 | 97.495 | 10 | 7.5 | -0.643 | 3,214 | 2,667 | 45.5 | 14 | 229.6 | 251.4 |
| Gorai.011G022500 | 490 | 54.325 | -5.5 | 5.666 | -0.151 | 2,009 | 1,473 | 44.3 | 10 | 200.9 | 207.2 |
| Gorai.011G023000 | 585 | 65.906 | 18 | 9.305 | -0.338 | 2,580 | 1,758 | 42.2 | 10 | 258 | 216 |
| Gorai.011G023100 | 586 | 65.793 | -1 | 6.357 | -0.12 | 1,988 | 1,761 | 48.4 | 3 | 662.7 | 640 |
| Gorai.011G023200 | 174 | 19.754 | 1 | 6.707 | 0.227 | 1,101 | 525 | 44.6 | 2 | 550.5 | 101 |
| Gorai.011G023300 | 584 | 64.604 | 9 | 8.195 | -0.01 | 2,334 | 1,755 | 39.9 | 1 | 2,334.00 | No intron |
| Gorai.011G023400 | 490 | 54.336 | 8 | 8.998 | 0.466 | 1,960 | 1,473 | 48.8 | 4 | 490 | 737 |
| Gorai.011G023500 | 263 | 29.309 | 12 | 10.17 | -0.583 | 1,593 | 792 | 48.7 | 7 | 227.6 | 318.5 |
| Gorai.011G023600 | 93 | 10.225 | 7.5 | 10.842 | -0.547 | 866 | 282 | 48.6 | 1 | 866 | No intron |
| Gorai.011G023700 | 415 | 45.785 | 0 | 6.505 | -0.008 | 1,919 | 1,248 | 44.9 | 9 | 213.2 | 268.9 |
| Gorai.011G023800 | 315 | 35.605 | -1.5 | 6.038 | -0.652 | 1,080 | 948 | 41.9 | 3 | 360 | 289 |
| Gorai.011G029400 | 195 | 22.68 | 8.5 | 8.304 | -0.789 | 909 | 588 | 45.1 | 2 | 454.5 | 617 |
| Gorai.011G029500 | 134 | 14.843 | 4.5 | 8.122 | -0.828 | 405 | 405 | 46.2 | 1 | 405 | No intron |
| Gorai.011G029600 | 623 | 68.238 | 5 | 7.004 | -0.117 | 2,323 | 1,872 | 43.1 | 10 | 232.3 | 319 |
| Gorai.011G029700 | 169 | 18.366 | 10.5 | 10.846 | -0.54 | 1,625 | 510 | 51.6 | 1 | 1,625.00 | No intron |
| Gorai.011G035300 | 772 | 83.724 | 7.5 | 7.189 | -0.058 | 2,319 | 2,319 | 45.5 | 9 | 257.7 | 87.1 |
| Gorai.011G035400 | 361 | 39.872 | -5.5 | 4.752 | -0.279 | 1,540 | 1,086 | 43.8 | 5 | 308 | 194 |
| Gorai.011G035500 | 270 | 31.137 | 8.5 | 9.495 | -0.676 | 1,378 | 813 | 42.3 | 8 | 172.3 | 1,208.10 |
| Gorai.011G044400 | 225 | 25.637 | 1.5 | 8.166 | -0.632 | 1,465 | 678 | 41.3 | 5 | 293 | 219.3 |
| Gorai.011G044500 | 763 | 86.651 | -8.5 | 5.792 | -0.275 | 2,912 | 2,292 | 42.1 | 17 | 171.3 | 143 |
| Gorai.011G044600 | 222 | 25.86 | 5 | 9.392 | -0.78 | 1,062 | 669 | 41.6 | 4 | 265.5 | 117.7 |
| Gorai.011G044700 | 437 | 48.27 | 8 | 9.069 | -0.13 | 1,624 | 1,314 | 42.9 | 3 | 541.3 | 434.5 |
| Gorai.011G044800 | 125 | 13.146 | -6.5 | 4.221 | 0.036 | 640 | 378 | 45.8 | 1 | 640 | No intron |
| Gorai.011G044900 | 821 | 93.777 | 12 | 8.148 | -0.415 | 2,985 | 2,466 | 43 | 17 | 175.6 | 227.2 |
| Gorai.011G045000 | 409 | 45.96 | -1.5 | 6.244 | -0.264 | 1,880 | 1,230 | 45 | 3 | 626.7 | 2,170.00 |
| Gorai.011G045100 | 341 | 36.447 | -1 | 6.245 | -0.338 | 1,428 | 1,026 | 45 | 3 | 476 | 1,434.50 |
| Gorai.011G054700 | 121 | 13.656 | 8.5 | 8.671 | 0.499 | 826 | 366 | 45.1 | 1 | 826 | No intron |
| Gorai.011G054800 | 287 | 31.357 | -2 | 6.09 | -0.272 | 864 | 864 | 43.3 | 1 | 864 | No intron |
| Gorai.011G054900 | 384 | 43.694 | 21.5 | 9.851 | -0.74 | 1,591 | 1,155 | 43.5 | 11 | 144.6 | 408.7 |
| Gorai.011G055000 | 79 | 8.299 | 10 | 12.546 | -0.299 | 648 | 240 | 41.2 | 1 | 648 | No intron |
| Gorai.011G055100 | 579 | 64.371 | 25.5 | 9.611 | -0.345 | 1,861 | 1,740 | 47 | 2 | 930.5 | 77 |
| Gorai.011G057100 | 240 | 26.21 | -2 | 5.748 | -0.803 | 1,468 | 723 | 45.1 | 6 | 244.7 | 235.2 |
| Gorai.011G057200 | 1,425 | 157.211 | 5.5 | 6.912 | -0.869 | 5,232 | 4,278 | 44.2 | 11 | 475.6 | 624.8 |
| Gorai.011G057300 | 1,614 | 177.726 | 24.5 | 7.702 | -0.125 | 5,025 | 4,845 | 43.7 | 9 | 558.3 | 149 |
| Gorai.011G057400 | 525 | 57.503 | 21 | 10.138 | -0.559 | 1,986 | 1,578 | 43.2 | 5 | 397.2 | 126.3 |
| Gorai.011G057500 | 253 | 28.589 | -6.5 | 5.013 | -0.723 | 1,359 | 762 | 44.6 | 5 | 271.8 | 581.8 |
| Gorai.011G057600 | 333 | 36.664 | 9.5 | 7.941 | -0.612 | 1,467 | 1,002 | 43.1 | 6 | 244.5 | 398.6 |
| Gorai.011G060200 | 475 | 52.239 | -1 | 6.402 | -0.186 | 1,696 | 1,428 | 44.1 | 8 | 212 | 183.9 |
| Gorai.011G060300 | 188 | 21.556 | 16.5 | 10.613 | -0.638 | 855 | 567 | 45.1 | 4 | 213.8 | 583.7 |
| Gorai.011G061600 | 276 | 31.67 | 2.5 | 7.855 | -0.923 | 1,096 | 831 | 40 | 5 | 219.2 | 86.3 |
| Gorai.011G061700 | 511 | 57.328 | 0 | 6.515 | 0.252 | 2,148 | 1,536 | 43.2 | 5 | 429.6 | 232.3 |
| Gorai.011G061800 | 627 | 71.099 | -12.5 | 5.278 | -0.331 | 2,835 | 1,884 | 45 | 2 | 1,417.50 | 141 |
| Gorai.011G065600 | 238 | 26.311 | 4 | 7.942 | -0.206 | 994 | 717 | 44.9 | 1 | 994 | No intron |
| Gorai.011G065700 | 235 | 26.568 | 7.5 | 9.701 | -0.397 | 1,186 | 708 | 46.2 | 7 | 169.4 | 402.7 |
| Gorai.011G065800 | 239 | 26.293 | -2 | 5.317 | -0.234 | 720 | 720 | 46.5 | 1 | 720 | No intron |
| Gorai.011G065900 | 239 | 26.187 | -3 | 5.027 | -0.2 | 1,052 | 720 | 45.4 | 1 | 1,052.00 | No intron |
| Gorai.011G066000 | 505 | 55.72 | 11 | 9.399 | -0.415 | 1,868 | 1,518 | 45.5 | 11 | 169.8 | 425.7 |
| Gorai.011G066100 | 195 | 22.157 | 11 | 10.255 | -0.508 | 870 | 588 | 42.3 | 2 | 435 | 1,865.00 |
| Gorai.011G068100 | 673 | 78.475 | 26.5 | 9.752 | 0.002 | 2,962 | 2,022 | 40 | 16 | 185.1 | 191.8 |
| Gorai.011G068200 | 203 | 22.639 | 2.5 | 8.982 | -0.646 | 1,319 | 612 | 50.8 | 2 | 659.5 | 197 |
| Gorai.011G068300 | 183 | 19.894 | 11.5 | 10.157 | -0.328 | 1,007 | 552 | 46.6 | 3 | 335.7 | 112 |
| Gorai.011G068400 | 453 | 48.948 | 0 | 6.514 | 0.036 | 1,883 | 1,362 | 44 | 5 | 376.6 | 212 |
| Gorai.011G068700 | 952 | 107.874 | 10.5 | 7.66 | -0.219 | 2,860 | 2,859 | 41.5 | 2 | 1,430.00 | 54 |
| Gorai.011G068800 | 377 | 40.925 | 1.5 | 6.736 | -0.11 | 1,586 | 1,134 | 47.1 | 5 | 317.2 | 279.3 |
| Gorai.011G068900 | 387 | 44.421 | 15.5 | 9.424 | -0.144 | 2,052 | 1,164 | 45.1 | 2 | 1,026.00 | 1,516.00 |
| Gorai.011G069000 | 67 | 7.617 | 17.5 | 11.92 | -1.343 | 648 | 204 | 53.4 | 2 | 324 | 662 |
| Gorai.011G069100 | 551 | 61.586 | 14.5 | 8.058 | -0.27 | 1,976 | 1,656 | 40.6 | 10 | 197.6 | 371.7 |
| Gorai.011G069200 | 542 | 59.978 | 6.5 | 7.402 | -0.555 | 1,629 | 1,629 | 41.1 | 5 | 325.8 | 414.3 |
| Gorai.011G078100 | 708 | 78.886 | 0.5 | 6.574 | -0.806 | 2,474 | 2,127 | 42.2 | 12 | 206.2 | 424.5 |
| Gorai.011G078200 | 891 | 101.804 | 7.5 | 6.97 | -0.493 | 3,566 | 2,676 | 40.8 | 26 | 137.2 | 397.4 |
| Gorai.011G078300 | 420 | 45.922 | -13 | 4.767 | -0.328 | 2,116 | 1,263 | 44.5 | 5 | 423.2 | 694 |
| Gorai.011G089300 | 109 | 12.446 | -1.5 | 6.14 | -0.018 | 909 | 330 | 43.3 | 2 | 454.5 | 279 |
| Gorai.011G089400 | 464 | 51.447 | 15 | 9.612 | -0.714 | 2,341 | 1,395 | 42.9 | 10 | 234.1 | 561.8 |
| Gorai.011G098600 | 289 | 32.185 | 0 | 6.508 | -0.875 | 998 | 870 | 45.9 | 3 | 332.7 | 96 |
| Gorai.011G098700 | 100 | 11.502 | 12.5 | 10.7 | -0.481 | 673 | 303 | 45.2 | 3 | 224.3 | 102 |
| Gorai.011G098800 | 316 | 34.545 | 15.5 | 8.839 | -0.642 | 1,401 | 951 | 45.8 | 7 | 200.1 | 290.5 |
| Gorai.011G098900 | 89 | 10.34 | 4.5 | 9.635 | -0.746 | 270 | 270 | 38.9 | 2 | 135 | 51 |
| Gorai.011G099000 | 104 | 11.827 | 6 | 9.593 | -0.321 | 964 | 315 | 41.9 | 1 | 964 | No intron |
| Gorai.011G099100 | 96 | 11.127 | 9.5 | 10.956 | -1.051 | 291 | 291 | 42.6 | 2 | 145.5 | 1,421.00 |
| Gorai.011G099200 | 260 | 28.115 | 37 | 11.517 | -0.45 | 1,224 | 783 | 52.2 | 2 | 612 | 439 |
| Gorai.011G101600 | 133 | 15.281 | 1.5 | 7.219 | -0.361 | 797 | 402 | 40 | 6 | 132.8 | 590 |
| Gorai.011G101700 | 80 | 9.09 | 4 | 9.696 | -0.88 | 791 | 243 | 48.1 | 2 | 395.5 | 579 |
| Gorai.011G102600 | 645 | 71.892 | -13 | 5.238 | -0.326 | 2,371 | 1,938 | 42.6 | 16 | 148.2 | 197.9 |
| Gorai.011G102700 | 372 | 40.197 | 0.5 | 6.607 | -0.04 | 1,696 | 1,119 | 42.4 | 1 | 1,696.00 | No intron |
| Gorai.011G105100 | 286 | 31.332 | 6 | 8.139 | 0.008 | 1,208 | 861 | 48.9 | 5 | 241.6 | 172.8 |
| Gorai.011G105200 | 402 | 45.762 | 10 | 8.624 | 0.254 | 1,209 | 1,209 | 39.7 | 4 | 302.3 | 80 |
| Gorai.011G105300 | 337 | 38.807 | 13 | 9.098 | -0.163 | 1,719 | 1,014 | 39.8 | 6 | 286.5 | 291.8 |
| Gorai.011G108400 | 475 | 52.003 | 2 | 6.717 | -0.515 | 2,380 | 1,428 | 46.5 | 4 | 595 | 320.7 |
| Gorai.011G108500 | 258 | 28.346 | 9 | 9.014 | -0.121 | 1,295 | 777 | 45.3 | 7 | 185 | 221.8 |
| Gorai.011G108600 | 279 | 30.772 | 14.5 | 10.141 | -0.111 | 840 | 840 | 47.4 | 1 | 840 | No intron |
| Gorai.011G108700 | 473 | 53.821 | 9.5 | 8.304 | -0.29 | 1,422 | 1,422 | 43.4 | 5 | 284.4 | 75 |
| Gorai.011G114300 | 174 | 18.922 | 3.5 | 8.123 | -0.156 | 941 | 525 | 45 | 2 | 470.5 | 1,736.00 |
| Gorai.011G114400 | 506 | 55.498 | 6 | 7.98 | -0.078 | 1,713 | 1,521 | 38.4 | 2 | 856.5 | 1,299.00 |
| Gorai.011G131000 | 472 | 53.384 | 17.5 | 9.487 | -0.411 | 2,182 | 1,419 | 42.2 | 12 | 181.8 | 766.7 |
| Gorai.011G135400 | 207 | 24.005 | 23 | 11.599 | -0.706 | 1,044 | 624 | 44.2 | 2 | 522 | 1,434.00 |
| Gorai.011G135500 | 523 | 58.439 | -6.5 | 5.447 | -0.723 | 2,055 | 1,572 | 42.9 | 5 | 411 | 181 |
| Gorai.011G135800 | 155 | 17.692 | 10.5 | 10.257 | -0.168 | 468 | 468 | 37.2 | 3 | 156 | 119.5 |
| Gorai.011G138600 | 449 | 51.903 | -3 | 6.026 | -0.398 | 2,137 | 1,350 | 42.5 | 10 | 213.7 | 497.4 |
| Gorai.011G138700 | 951 | 104.149 | -21 | 4.943 | -0.396 | 3,733 | 2,856 | 42.6 | 6 | 622.2 | 621.4 |
| Gorai.011G158300 | 832 | 94.669 | 20.5 | 8.401 | -0.31 | 2,499 | 2,499 | 47.1 | 1 | 2,499.00 | No intron |
| Gorai.011G158400 | 244 | 28.087 | -2 | 5.789 | -0.536 | 1,280 | 735 | 42.2 | 5 | 256 | 327 |
| Gorai.011G158900 | 447 | 50.927 | 11 | 8.839 | -0.344 | 1,806 | 1,344 | 41.1 | 14 | 129 | 584.5 |
| Gorai.011G159000 | 206 | 23.628 | 33.5 | 11.132 | -0.362 | 1,191 | 621 | 48.1 | 4 | 297.8 | 475.3 |
| Gorai.011G160100 | 407 | 45.734 | -14.5 | 4.823 | -0.652 | 1,548 | 1,224 | 46.2 | 7 | 221.1 | 233 |
| Gorai.011G162100 | 1,131 | 122.446 | -10 | 5.322 | 0.04 | 3,967 | 3,396 | 44 | 2 | 1,983.50 | 99 |
| Gorai.011G162200 | 259 | 28.9 | -1.5 | 6.157 | -0.031 | 1,124 | 780 | 42.1 | 4 | 281 | 418 |
| Gorai.011G162300 | 344 | 39.544 | 10 | 8.358 | -0.323 | 1,035 | 1,035 | 33.1 | 5 | 207 | 453 |
| Gorai.011G164100 | 461 | 51.292 | 12.5 | 8.392 | -0.298 | 1,386 | 1,386 | 42.3 | 19 | 72.9 | 555.8 |
| Gorai.011G164200 | 430 | 47.601 | 17.5 | 9.301 | -0.143 | 1,293 | 1,293 | 44.3 | 3 | 431 | 497 |
| Gorai.011G169700 | 318 | 35.63 | 5 | 7.927 | -0.086 | 1,971 | 957 | 47.9 | 2 | 985.5 | 207 |
| Gorai.011G169800 | 373 | 43.542 | 13.5 | 8.899 | -0.033 | 1,122 | 1,122 | 35.8 | 4 | 280.5 | 399.7 |
| Gorai.011G169900 | 54 | 5.961 | 1 | 8.211 | 0.663 | 165 | 165 | 36.4 | 1 | 165 | No intron |
| Gorai.011G173000 | 199 | 22.373 | -1.5 | 5.808 | -0.65 | 743 | 600 | 47.2 | 1 | 743 | No intron |
| Gorai.011G176700 | 378 | 42.486 | 6.5 | 8.423 | -0.061 | 1,291 | 1,134 | 41.3 | 2 | 645.5 | 541 |
| Gorai.011G176800 | 178 | 19.85 | 13 | 10.719 | -1.316 | 1,266 | 537 | 43.9 | 5 | 253.2 | 111.5 |
| Gorai.011G183400 | 481 | 53.975 | 9 | 7.608 | -0.538 | 1,971 | 1,446 | 44.3 | 10 | 197.1 | 285.9 |
| Gorai.011G183500 | 482 | 52.443 | -1 | 6.159 | 0.523 | 1,846 | 1,449 | 40.5 | 18 | 102.6 | 110.5 |
| Gorai.011G183600 | 171 | 19.349 | -0.5 | 6.392 | -0.326 | 736 | 516 | 41.9 | 6 | 122.7 | 230.8 |
| Gorai.011G183700 | 78 | 8.347 | 1 | 7.42 | 0.233 | 1,176 | 237 | 43 | 2 | 588 | 1,073.00 |
| Gorai.011G189100 | 128 | 14.212 | 0 | 6.595 | -0.498 | 580 | 387 | 45 | 3 | 193.3 | 117 |
| Gorai.011G189200 | 330 | 36.068 | 14.5 | 9.84 | 0.027 | 1,715 | 993 | 43.9 | 7 | 245 | 401.2 |
| Gorai.011G205600 | 580 | 64.604 | 0 | 6.525 | -0.144 | 2,033 | 1,743 | 45.8 | 5 | 406.6 | 379 |
| Gorai.011G205700 | 904 | 98.793 | 44.5 | 10.368 | 0.068 | 2,855 | 2,715 | 45.6 | 6 | 475.8 | 150 |
| Gorai.011G205800 | 383 | 43.078 | 7 | 8.622 | -0.501 | 1,670 | 1,152 | 42.8 | 17 | 98.2 | 176.4 |
| Gorai.011G205900 | 761 | 84.063 | -5 | 6.09 | 0.007 | 2,797 | 2,286 | 47.2 | 13 | 215.2 | 549.6 |
| Gorai.011G208800 | 111 | 12.566 | 2.5 | 7.635 | -0.205 | 584 | 336 | 45.5 | 1 | 584 | No intron |
| Gorai.011G208900 | 153 | 16.979 | 1 | 7.248 | -0.151 | 813 | 462 | 45 | 6 | 135.5 | 91.4 |
| Gorai.011G209500 | 555 | 62.658 | 9.5 | 8.225 | -0.175 | 2,092 | 1,668 | 46.6 | 2 | 1,046.00 | 206 |
| Gorai.011G215200 | 584 | 64.224 | 22.5 | 10.002 | 0.272 | 2,002 | 1,755 | 46.3 | 5 | 400.4 | 567.5 |
| Gorai.011G215300 | 431 | 48.921 | 10 | 8.309 | -0.339 | 2,001 | 1,296 | 43.4 | 8 | 250.1 | 332.7 |
| Gorai.011G215400 | 280 | 30.19 | 1.5 | 6.946 | -0.611 | 2,312 | 843 | 46.3 | 4 | 578 | 560.7 |
| Gorai.011G215500 | 81 | 9.363 | 5.5 | 8.913 | 0.579 | 1,191 | 246 | 38.6 | 1 | 1,191.00 | No intron |
| Gorai.011G215600 | 717 | 81.326 | 10 | 7.816 | -0.638 | 2,576 | 2,154 | 41.8 | 8 | 322 | 152.6 |
| Gorai.011G232400 | 85 | 9.43 | -6.5 | 4.239 | 0.105 | 839 | 258 | 40.3 | 1 | 839 | No intron |
| Gorai.011G232500 | 509 | 58.474 | 3 | 6.94 | -0.128 | 1,859 | 1,530 | 41.7 | 2 | 929.5 | 97 |
| Gorai.011G237900 | 217 | 24.916 | 3.5 | 7.545 | 0.166 | 1,211 | 654 | 38.1 | 8 | 151.4 | 489.7 |
| Gorai.011G266200 | 808 | 90.172 | -16 | 5.101 | -0.417 | 2,878 | 2,427 | 44 | 3 | 959.3 | 258 |
| Gorai.011G266300 | 489 | 53.454 | 20 | 9.26 | -0.454 | 2,052 | 1,470 | 43.2 | 7 | 293.1 | 86.7 |
| Gorai.011G266400 | 724 | 82.934 | -2 | 6.331 | -0.668 | 2,465 | 2,175 | 44.4 | 12 | 205.4 | 159.8 |
| Gorai.011G266500 | 137 | 15.616 | 8.5 | 9.891 | -0.558 | 522 | 414 | 46.6 | 1 | 522 | No intron |
| Gorai.011G266600 | 77 | 8.914 | 2 | 8.772 | 1.418 | 1,085 | 234 | 35.9 | 2 | 542.5 | 313 |
| Gorai.012G002900 | 1,055 | 116.734 | -15 | 5.497 | -0.233 | 3,807 | 3,168 | 42.7 | 27 | 141 | 648.5 |
| Gorai.012G003900 | 803 | 88.162 | -3.5 | 6.202 | -0.298 | 3,122 | 2,412 | 44.2 | 17 | 183.6 | 392.3 |
| Gorai.012G004000 | 282 | 32.553 | 9 | 8.462 | 0.15 | 1,379 | 849 | 42 | 3 | 459.7 | 133 |
| Gorai.012G004100 | 578 | 66.847 | 26.5 | 9.426 | -0.067 | 1,737 | 1,737 | 40.5 | 15 | 115.8 | 151.8 |
| Gorai.012G004200 | 191 | 21.299 | -2 | 5.283 | -0.445 | 1,120 | 576 | 46 | 6 | 186.7 | 652 |
| Gorai.012G013100 | 258 | 28.583 | -1.5 | 6.278 | -0.334 | 1,440 | 777 | 47.5 | 7 | 205.7 | 312.5 |
| Gorai.012G013200 | 82 | 9.345 | 9 | 10.61 | -0.235 | 289 | 249 | 45.8 | 3 | 96.3 | 1,093.00 |
| Gorai.012G013300 | 653 | 73.679 | 18.5 | 8.45 | 0.225 | 3,273 | 1,962 | 38.4 | 4 | 818.3 | 146.3 |
| Gorai.012G013400 | 220 | 25.062 | 33.5 | 11 | -0.587 | 1,142 | 663 | 48.1 | 4 | 285.5 | 415.7 |
| Gorai.012G013500 | 730 | 81.665 | 2.5 | 6.619 | -0.768 | 3,052 | 2,193 | 42.5 | 3 | 1,017.30 | 108.5 |
| Gorai.012G013600 | 555 | 61.96 | 1.5 | 7.027 | -0.079 | 2,046 | 1,668 | 42.9 | 10 | 204.6 | 281.6 |
| Gorai.012G013700 | 340 | 38.373 | -2.5 | 5.957 | -0.314 | 1,023 | 1,023 | 41 | 9 | 113.7 | 177.9 |
| Gorai.012G014000 | 373 | 40.743 | 3.5 | 6.912 | -0.693 | 1,122 | 1,122 | 45.2 | 3 | 374 | 686 |
| Gorai.012G014100 | 174 | 19.902 | 11 | 9.982 | -0.871 | 861 | 525 | 42.3 | 3 | 287 | 86 |
| Gorai.012G014200 | 792 | 88.585 | 13 | 7.344 | 0.365 | 3,333 | 2,379 | 43.5 | 10 | 333.3 | 272.8 |
| Gorai.012G014300 | 87 | 9.907 | 2 | 8.208 | -0.278 | 461 | 264 | 42 | 1 | 461 | No intron |
| Gorai.012G014400 | 253 | 27.075 | 4 | 7.879 | -0.065 | 1,218 | 762 | 49.5 | 3 | 406 | 129 |
| Gorai.012G015500 | 530 | 56.466 | 7 | 8.189 | 0.501 | 1,986 | 1,593 | 48.4 | 2 | 993 | 123 |
| Gorai.012G022300 | 304 | 33.237 | -8.5 | 5.188 | -0.76 | 1,831 | 915 | 45.8 | 6 | 305.2 | 954 |
| Gorai.012G022400 | 1,287 | 132.286 | 17 | 9.544 | -0.327 | 4,489 | 3,864 | 45.3 | 9 | 498.8 | 590.1 |
| Gorai.012G022500 | 1,392 | 141.896 | 18 | 9.02 | -0.29 | 4,571 | 4,179 | 45 | 10 | 457.1 | 694.3 |
| Gorai.012G023300 | 727 | 82.903 | 6.5 | 7.545 | -0.423 | 3,069 | 2,184 | 41.7 | 7 | 438.4 | 222.8 |
| Gorai.012G023400 | 423 | 47.296 | 3.5 | 6.908 | -0.252 | 1,272 | 1,272 | 49.3 | 2 | 636 | 2,729.00 |
| Gorai.012G023500 | 643 | 71.849 | 1 | 6.64 | -0.182 | 1,949 | 1,932 | 42.2 | 6 | 324.8 | 510 |
| Gorai.012G023600 | 1,487 | 168.404 | 7.5 | 7.06 | 0.039 | 4,633 | 4,464 | 42.3 | 20 | 231.7 | 128 |
| Gorai.012G023700 | 971 | 111.028 | 16.5 | 7.491 | -0.931 | 3,583 | 2,916 | 42.3 | 3 | 1,194.30 | 1,186.50 |
| Gorai.012G029200 | 82 | 9.482 | 0.5 | 6.778 | -0.28 | 1,070 | 249 | 38.6 | 2 | 535 | 626 |
| Gorai.012G029300 | 378 | 42.527 | 0 | 6.523 | -1.092 | 1,514 | 1,137 | 43.6 | 4 | 378.5 | 103.3 |
| Gorai.012G029400 | 111 | 13.009 | 7.5 | 8.885 | 0.292 | 1,672 | 336 | 39.6 | 4 | 418 | 764 |
| Gorai.012G029500 | 387 | 43.696 | -16.5 | 4.432 | -0.065 | 1,286 | 1,164 | 44.5 | 5 | 257.2 | 623.5 |
| Gorai.012G029600 | 294 | 33.497 | -3 | 5.295 | -0.251 | 1,153 | 885 | 46.7 | 3 | 384.3 | 412.5 |
| Gorai.012G029700 | 242 | 28.145 | 12.5 | 10.497 | -0.395 | 1,460 | 729 | 49 | 4 | 365 | 669.3 |
| Gorai.012G029800 | 580 | 65.724 | -3 | 5.969 | -0.265 | 1,927 | 1,743 | 41.5 | 4 | 481.8 | 3,596.00 |
| Gorai.012G034300 | 551 | 62.348 | 14.5 | 8.485 | -0.401 | 2,448 | 1,656 | 46.7 | 2 | 1,224.00 | 3,568.00 |
| Gorai.012G034400 | 495 | 54.015 | -32 | 4.368 | -0.449 | 2,137 | 1,488 | 43.3 | 18 | 118.7 | 252.8 |
| Gorai.012G034500 | 662 | 71.692 | 23.5 | 9.394 | -0.573 | 2,380 | 1,989 | 46 | 11 | 216.4 | 259.6 |
| Gorai.012G046500 | 239 | 27.059 | 2 | 6.841 | -0.389 | 1,834 | 720 | 44.9 | 6 | 305.7 | 740.4 |
| Gorai.012G046600 | 215 | 24.126 | -2 | 6.082 | 0.094 | 648 | 648 | 44.6 | 4 | 162 | 666 |
| Gorai.012G046700 | 197 | 22.309 | 4 | 8.351 | -0.733 | 1,373 | 594 | 44.9 | 5 | 274.6 | 134 |
| Gorai.012G047100 | 662 | 73.362 | 20 | 9.692 | -0.873 | 3,208 | 1,989 | 44.1 | 6 | 534.7 | 410.4 |
| Gorai.012G047200 | 680 | 75.352 | 17.5 | 9.498 | -0.818 | 3,043 | 2,043 | 42.9 | 6 | 507.2 | 411 |
| Gorai.012G047300 | 663 | 73.854 | 20.5 | 9.487 | -0.872 | 2,674 | 1,992 | 43 | 5 | 534.8 | 213.5 |
| Gorai.012G047400 | 99 | 11.503 | -1.5 | 6.129 | 0.029 | 300 | 300 | 35 | 1 | 300 | No intron |
| Gorai.012G056700 | 159 | 18.242 | 16.5 | 10.759 | -0.487 | 480 | 480 | 35.2 | 2 | 240 | 616 |
| Gorai.012G056800 | 554 | 60.219 | -4.5 | 5.831 | -0.689 | 1,922 | 1,665 | 40.6 | 5 | 384.4 | 144.5 |
| Gorai.012G056900 | 330 | 36.767 | 11.5 | 9.358 | -0.276 | 993 | 993 | 41.3 | 3 | 331 | 429 |
| Gorai.012G057700 | 523 | 58.104 | 5 | 7.211 | -0.248 | 2,331 | 1,572 | 41.1 | 3 | 777 | 498.5 |
| Gorai.012G057800 | 309 | 34.251 | 2.5 | 6.826 | -0.376 | 1,334 | 930 | 44.5 | 7 | 190.6 | 247.8 |
| Gorai.012G057900 | 76 | 8.8 | 4.5 | 8.919 | 1.188 | 609 | 231 | 33.3 | 1 | 609 | No intron |
| Gorai.012G058000 | 564 | 62.193 | 0.5 | 6.584 | -0.061 | 2,069 | 1,695 | 44.7 | 2 | 1,034.50 | 90 |
| Gorai.012G058100 | 1,249 | 136.463 | 16.5 | 8.164 | 0.133 | 4,566 | 3,750 | 42.7 | 8 | 570.8 | 341.6 |
| Gorai.012G060300 | 67 | 7.648 | 9 | 10.523 | -1.145 | 1,001 | 204 | 39.7 | 5 | 200.2 | 570 |
| Gorai.012G060400 | 74 | 7.98 | 3.5 | 9.381 | 0.238 | 225 | 225 | 47.6 | 1 | 225 | No intron |
| Gorai.012G060500 | 778 | 86.968 | 22.5 | 8.715 | 0.347 | 3,352 | 2,337 | 40.7 | 7 | 478.9 | 285.7 |
| Gorai.012G060600 | 314 | 35.853 | 4 | 8.034 | 0.618 | 1,071 | 945 | 39.4 | 1 | 1,071.00 | No intron |
| Gorai.012G068400 | 113 | 13.211 | 11.5 | 10.016 | 0.012 | 610 | 342 | 46.5 | 1 | 610 | No intron |
| Gorai.012G068500 | 605 | 65.943 | 10 | 8.135 | 0.36 | 2,147 | 1,818 | 41.1 | 14 | 153.4 | 191.2 |
| Gorai.012G068600 | 212 | 23.472 | 2.5 | 7.554 | -0.07 | 1,160 | 639 | 43.3 | 6 | 193.3 | 213.6 |
| Gorai.012G070200 | 310 | 36.156 | 20 | 9.774 | -0.082 | 1,644 | 933 | 43 | 4 | 411 | 501.7 |
| Gorai.012G070300 | 434 | 48.265 | -4.5 | 5.664 | -0.16 | 1,607 | 1,305 | 46.1 | 4 | 401.8 | 336.3 |
| Gorai.012G072700 | 71 | 8.701 | 5.5 | 9.327 | -0.332 | 311 | 216 | 35.6 | 2 | 155.5 | 426 |
| Gorai.012G072800 | 333 | 36.538 | 10 | 8.764 | 0.171 | 1,002 | 1,002 | 44.5 | 5 | 200.4 | 101 |
| Gorai.012G072900 | 69 | 7.84 | 1 | 6.794 | -0.304 | 801 | 210 | 50.5 | 1 | 801 | No intron |
| Gorai.012G073000 | 256 | 29.153 | -18 | 4.333 | -0.663 | 1,266 | 771 | 43.7 | 3 | 422 | 225.5 |
| Gorai.012G073100 | 258 | 28.744 | -1.5 | 5.164 | 0.413 | 1,615 | 777 | 42.1 | 5 | 323 | 508.5 |
| Gorai.012G073200 | 79 | 8.58 | 5 | 10.134 | 0.692 | 505 | 240 | 39.6 | 2 | 252.5 | 642 |
| Gorai.012G074600 | 107 | 12.66 | 1.5 | 8.205 | 0.314 | 324 | 324 | 45.1 | 1 | 324 | No intron |
| Gorai.012G082300 | 107 | 12.303 | 3.5 | 9.215 | -0.459 | 1,105 | 324 | 39.8 | 7 | 157.9 | 292.7 |
| Gorai.012G082400 | 146 | 15.776 | 3 | 7.922 | 0.158 | 660 | 441 | 45.8 | 2 | 330 | 100 |
| Gorai.012G082500 | 788 | 87.52 | 19.5 | 8.935 | 0.449 | 2,909 | 2,367 | 43.2 | 9 | 323.2 | 326.8 |
| Gorai.012G084200 | 445 | 49.124 | -5 | 5.513 | -0.07 | 1,986 | 1,338 | 46 | 3 | 662 | 509.5 |
| Gorai.012G084300 | 643 | 71.598 | 12.5 | 8.413 | -0.003 | 2,514 | 1,932 | 41.6 | 2 | 1,257.00 | 83 |
| Gorai.012G084400 | 443 | 49.593 | 0 | 6.511 | -0.193 | 1,910 | 1,332 | 43.3 | 7 | 272.9 | 180.3 |
| Gorai.012G084500 | 205 | 23.457 | 19 | 10.783 | -0.28 | 1,898 | 618 | 39.5 | 4 | 474.5 | 55.7 |
| Gorai.012G084700 | 140 | 16.288 | 7.5 | 9.565 | -1.007 | 545 | 423 | 44.9 | 2 | 272.5 | 332 |
| Gorai.012G089600 | 479 | 53.535 | -20 | 4.614 | -0.359 | 2,247 | 1,440 | 39.4 | 3 | 749 | 960.5 |
| Gorai.012G089700 | 518 | 59.657 | -0.5 | 6.45 | -0.379 | 2,255 | 1,557 | 42.9 | 15 | 150.3 | 139.6 |
| Gorai.012G089800 | 295 | 33.389 | 5.5 | 9.116 | -0.622 | 1,166 | 888 | 43.6 | 8 | 145.8 | 201.4 |
| Gorai.012G090700 | 155 | 17.637 | 16.5 | 9.999 | -0.288 | 1,073 | 468 | 42.5 | 6 | 178.8 | 210.8 |
| Gorai.012G090800 | 158 | 17.681 | -2.5 | 5.326 | -0.423 | 682 | 477 | 38.6 | 4 | 170.5 | 188 |
| Gorai.012G090900 | 70 | 8.129 | 8.5 | 10.161 | 0.066 | 828 | 213 | 34.3 | 1 | 828 | No intron |
| Gorai.012G091000 | 381 | 42.44 | -5.5 | 5.15 | -0.259 | 1,690 | 1,146 | 48.2 | 2 | 845 | 660 |
| Gorai.012G096800 | 431 | 47.772 | 24 | 10.062 | -0.248 | 2,515 | 1,296 | 48.1 | 1 | 2,515.00 | No intron |
| Gorai.012G110000 | 567 | 62.589 | 15 | 8.505 | -0.071 | 1,875 | 1,704 | 47.8 | 6 | 312.5 | 306 |
| Gorai.012G110100 | 410 | 45.318 | -3.5 | 5.848 | -0.216 | 1,751 | 1,233 | 42.7 | 4 | 437.8 | 241 |
| Gorai.012G110200 | 515 | 55.409 | 6.5 | 8.241 | -0.577 | 2,164 | 1,548 | 44.2 | 7 | 309.1 | 163.7 |
| Gorai.012G110300 | 352 | 40.042 | 22 | 10.43 | -0.569 | 1,554 | 1,059 | 43.1 | 4 | 388.5 | 328.7 |
| Gorai.012G118800 | 453 | 52.057 | 7.5 | 7.403 | -0.438 | 1,727 | 1,362 | 46.7 | 6 | 287.8 | 174.2 |
| Gorai.012G118900 | 176 | 19.795 | 12.5 | 9.717 | -0.742 | 531 | 531 | 47.8 | 2 | 265.5 | 80 |
| Gorai.012G124900 | 392 | 43.762 | 4.5 | 7.474 | 0.037 | 2,635 | 1,179 | 43.5 | 2 | 1,317.50 | 443 |
| Gorai.012G125000 | 132 | 14.259 | -7 | 4.27 | -0.458 | 1,302 | 399 | 46.4 | 4 | 325.5 | 182.3 |
| Gorai.012G125100 | 212 | 24.616 | -3 | 5.705 | -0.969 | 1,123 | 639 | 42.9 | 2 | 561.5 | 79 |
| Gorai.012G125200 | 1,272 | 139.963 | -19.5 | 5.354 | -0.556 | 5,225 | 3,819 | 43.1 | 12 | 435.4 | 125.7 |
| Gorai.012G131000 | 293 | 32.965 | 3.5 | 8.17 | 0.123 | 1,209 | 882 | 42.1 | 14 | 86.4 | 306.6 |
| Gorai.012G131100 | 976 | 109.306 | -11 | 5.487 | -0.174 | 3,588 | 2,931 | 40.9 | 2 | 1,794.00 | 102 |
| Gorai.012G131200 | 101 | 11.145 | 7.5 | 10.346 | -0.15 | 541 | 306 | 47.4 | 3 | 180.3 | 252.5 |
| Gorai.012G132800 | 604 | 69.723 | -4.5 | 6.12 | -0.289 | 2,227 | 1,815 | 41.8 | 7 | 318.1 | 126.2 |
| Gorai.012G134400 | 207 | 23.952 | 6.5 | 8.975 | -0.271 | 938 | 624 | 44.2 | 2 | 469 | 1,164.00 |
| Gorai.012G134500 | 700 | 78.076 | -3.5 | 6.246 | -0.037 | 2,210 | 2,103 | 42.8 | 2 | 1,105.00 | 84 |
| Gorai.012G134600 | 719 | 81.12 | -15.5 | 5.226 | -0.129 | 2,409 | 2,160 | 39.8 | 2 | 1,204.50 | 74 |
| Gorai.012G134700 | 509 | 57.423 | 2 | 6.75 | -0.304 | 1,749 | 1,530 | 43.3 | 9 | 194.3 | 473 |
| Gorai.012G134800 | 183 | 20.164 | 2 | 8.045 | -0.154 | 1,033 | 552 | 49.3 | 2 | 516.5 | 143 |
| Gorai.013G035300 | 624 | 70.892 | 18.5 | 8.979 | -0.53 | 2,346 | 1,875 | 42.8 | 14 | 167.6 | 241.6 |
| Gorai.013G035400 | 588 | 67.41 | 16.5 | 8.969 | -0.59 | 1,835 | 1,767 | 44.5 | 13 | 141.2 | 103.9 |
| Gorai.013G035500 | 488 | 54.088 | 7.5 | 7.429 | -0.159 | 1,905 | 1,467 | 45.7 | 8 | 238.1 | 393.9 |
| Gorai.013G035600 | 229 | 26.787 | 17.5 | 10.354 | -0.561 | 743 | 690 | 45.2 | 1 | 743 | No intron |
| Gorai.013G047800 | 235 | 26.378 | -2.5 | 5.619 | -0.301 | 937 | 708 | 47.7 | 1 | 937 | No intron |
| Gorai.013G047900 | 1,134 | 125.951 | 6.5 | 6.864 | -0.47 | 3,862 | 3,405 | 41.9 | 25 | 154.5 | 279 |
| Gorai.013G048000 | 248 | 26.519 | 1.5 | 6.776 | -0.554 | 1,165 | 747 | 48.6 | 4 | 291.3 | 99 |
| Gorai.013G072500 | 1,029 | 115.287 | -6.5 | 5.771 | -0.302 | 3,235 | 3,090 | 41.7 | 7 | 462.1 | 111 |
| Gorai.013G072600 | 120 | 13.044 | -14 | 3.929 | 0.004 | 784 | 363 | 52.6 | 1 | 784 | No intron |
| Gorai.013G073200 | 415 | 45.737 | 5.5 | 8.582 | 0.48 | 1,801 | 1,248 | 41.7 | 9 | 200.1 | 277.3 |
| Gorai.013G073300 | 94 | 10.73 | 5 | 10.004 | -0.757 | 355 | 285 | 40 | 3 | 118.3 | 594 |
| Gorai.013G085800 | 420 | 46.558 | 18 | 9.472 | 0.707 | 1,612 | 1,263 | 41.5 | 3 | 537.3 | 188 |
| Gorai.013G085900 | 272 | 30.916 | 3 | 7.223 | -0.902 | 2,271 | 819 | 42.2 | 7 | 324.4 | 231.8 |
| Gorai.013G086000 | 516 | 58.124 | 9.5 | 7.684 | -0.131 | 2,241 | 1,551 | 40.3 | 2 | 1,120.50 | 288 |
| Gorai.013G086100 | 227 | 25.329 | -5 | 4.806 | -0.343 | 1,067 | 684 | 41.1 | 4 | 266.8 | 206.3 |
| Gorai.013G088200 | 313 | 35.613 | 9.5 | 8.299 | -0.537 | 1,056 | 942 | 46.3 | 3 | 352 | 96.5 |
| Gorai.013G097700 | 516 | 58.718 | -5.5 | 5.448 | -0.337 | 1,743 | 1,551 | 42.4 | 8 | 217.9 | 152.6 |
| Gorai.013G112000 | 508 | 57.101 | -13.5 | 4.95 | -0.499 | 1,982 | 1,527 | 45.3 | 4 | 495.5 | 1,442.70 |
| Gorai.013G115000 | 273 | 30.28 | 0 | 6.517 | -0.682 | 928 | 822 | 42.2 | 1 | 928 | No intron |
| Gorai.013G115100 | 449 | 49.67 | -15.5 | 4.771 | -0.137 | 1,845 | 1,350 | 45.6 | 5 | 369 | 213.3 |
| Gorai.013G117500 | 439 | 49.545 | -16.5 | 4.713 | -0.9 | 1,416 | 1,320 | 40.6 | 11 | 128.7 | 200.5 |
| Gorai.013G117600 | 500 | 54.302 | 7 | 8.194 | -0.003 | 1,564 | 1,503 | 44.8 | 3 | 521.3 | 214 |
| Gorai.013G117700 | 330 | 36.135 | -14 | 4.537 | -0.455 | 1,860 | 993 | 45.8 | 3 | 620 | 163 |
| Gorai.013G117800 | 114 | 12.751 | -14.5 | 4.106 | -0.474 | 651 | 345 | 44.6 | 2 | 325.5 | 97 |
| Gorai.013G125500 | 85 | 9.582 | 4 | 8.131 | -0.132 | 612 | 258 | 45.7 | 3 | 204 | 468.5 |
| Gorai.013G125600 | 272 | 29.522 | 2.5 | 8.429 | -0.165 | 1,099 | 819 | 45.9 | 3 | 366.3 | 2,261.00 |
| Gorai.013G141200 | 430 | 48.289 | 14.5 | 7.971 | 0.233 | 1,293 | 1,293 | 41.1 | 5 | 258.6 | 118.3 |
| Gorai.013G141300 | 143 | 16.315 | 4.5 | 8.861 | -0.263 | 1,319 | 432 | 53.9 | 2 | 659.5 | 156 |
| Gorai.013G149400 | 69 | 7.624 | 1.5 | 7.691 | 0.709 | 743 | 210 | 37.6 | 1 | 743 | No intron |
| Gorai.013G149500 | 418 | 45.855 | -14.5 | 4.678 | -0.243 | 1,513 | 1,257 | 43.7 | 16 | 94.6 | 171.4 |
| Gorai.013G149600 | 223 | 25.146 | 12 | 10.133 | -0.327 | 1,274 | 672 | 44.2 | 2 | 637 | 406 |
| Gorai.013G149700 | 705 | 78.513 | -12 | 5.296 | -0.535 | 2,681 | 2,118 | 45.1 | 2 | 1,340.50 | 310 |
| Gorai.013G149800 | 207 | 23.506 | 2.5 | 7.008 | -0.301 | 624 | 624 | 40.1 | 3 | 208 | 104.5 |
| Gorai.013G149900 | 314 | 35.485 | 3.5 | 7.222 | -0.311 | 1,509 | 945 | 44.9 | 4 | 377.3 | 252.3 |
| Gorai.013G150000 | 357 | 38.317 | 2.5 | 7.841 | -0.168 | 1,297 | 1,074 | 53 | 3 | 432.3 | 153.5 |
| Gorai.013G157700 | 107 | 12.388 | 4 | 8.922 | 0.312 | 573 | 324 | 46.3 | 2 | 286.5 | 76 |
| Gorai.013G175100 | 503 | 57.852 | 15.5 | 8.757 | -0.271 | 3,501 | 1,512 | 42.1 | 2 | 1,750.50 | 830 |
| Gorai.013G175200 | 356 | 40.212 | 10.5 | 8.778 | -0.041 | 1,431 | 1,071 | 43.9 | 4 | 357.8 | 467.7 |
| Gorai.013G204400 | 382 | 41.801 | 4.5 | 7.504 | -0.386 | 1,547 | 1,149 | 49 | 1 | 1,547.00 | No intron |
| Gorai.013G204500 | 425 | 47.018 | 15 | 9.247 | -0.569 | 1,770 | 1,278 | 42.6 | 10 | 177 | 282.1 |
| Gorai.013G204600 | 429 | 47.671 | 20.5 | 9.404 | -0.135 | 1,727 | 1,290 | 43.7 | 6 | 287.8 | 116.8 |
| Gorai.013G204700 | 283 | 30.93 | 2 | 7.49 | 0.395 | 1,239 | 852 | 42 | 6 | 206.5 | 259.6 |
| Gorai.013G204800 | 211 | 24.352 | 14 | 10.05 | -0.293 | 636 | 636 | 38.4 | 4 | 159 | 702 |
| Gorai.013G204900 | 323 | 35.348 | 16 | 10.179 | 0.014 | 1,401 | 972 | 43.9 | 5 | 280.2 | 395.5 |
| Gorai.013G206000 | 245 | 27.202 | 4 | 8.945 | 0.642 | 1,174 | 738 | 41.9 | 6 | 195.7 | 98.2 |
| Gorai.013G206100 | 187 | 20.957 | 28.5 | 11.66 | -0.368 | 1,136 | 564 | 46.3 | 5 | 227.2 | 162 |
| Gorai.013G206200 | 801 | 90.117 | -12 | 5.308 | -0.818 | 2,978 | 2,406 | 42.4 | 4 | 744.5 | 223.7 |
| Gorai.013G208800 | 349 | 40.126 | 4.5 | 7.311 | -0.462 | 1,152 | 1,050 | 44.2 | 1 | 1,152.00 | No intron |
| Gorai.013G208900 | 139 | 15.28 | -8 | 4.501 | -1.248 | 1,351 | 420 | 47.4 | 2 | 675.5 | 118 |
| Gorai.013G227300 | 419 | 45.316 | 2 | 6.853 | -0.231 | 1,983 | 1,260 | 42.9 | 8 | 247.9 | 275.7 |
| Gorai.013G227400 | 526 | 59.528 | 13 | 9.022 | -0.234 | 1,866 | 1,581 | 41.2 | 5 | 373.2 | 466 |
| Gorai.013G227500 | 255 | 29.026 | 4.5 | 8.303 | -0.617 | 942 | 768 | 47.1 | 1 | 942 | No intron |
| Gorai.013G236300 | 221 | 24.414 | 18 | 9.915 | -0.61 | 1,209 | 666 | 44.9 | 4 | 302.3 | 588.7 |
| Gorai.013G236400 | 795 | 83.53 | -3 | 5.925 | 0.614 | 3,011 | 2,388 | 45.9 | 7 | 430.1 | 170.5 |
| Gorai.013G236500 | 609 | 67.309 | 10.5 | 7.331 | -0.123 | 2,501 | 1,830 | 43.3 | 13 | 192.4 | 184.8 |
| Gorai.013G236600 | 683 | 78.677 | -3.5 | 6.201 | -0.582 | 3,197 | 2,052 | 41.7 | 3 | 1,065.70 | 88 |
| Gorai.013G236700 | 288 | 33.082 | -3 | 6.08 | -0.498 | 1,142 | 867 | 41.4 | 9 | 126.9 | 428.3 |
| Gorai.013G236800 | 1,128 | 126.647 | -5.5 | 6.112 | -0.015 | 3,417 | 3,387 | 38.3 | 12 | 284.8 | 255.6 |
