# Supplementary material for: Simple Sequence Repeat (SSR) Genetic Linkage Map of D Genome Diploid Cotton Derived from an Interspecific Cross between Gossypium davidsonii and Gossypium klotzschianum
Source: Int J Mol Sci. 2018 Jan 11;19(1):204. doi: 10.3390/ijms19010204 (PMC5796153; doi:10.3390/ijms19010204)
Supplement: Supplementary file 1 [file ijms-19-00204-s001.zip › ijms-254865 final supplementary/Supplementary Table S4.docx]

Supplementary Table S4: RNA seq. expression profile in various tissues for the mind genes from the SSR regions

| Gene ID | Fiber in 10 days post-anthesis | Fiber in 20 days post-anthesis | Seed in 10 days post-anthesis | Seed in 20 days post-anthesis | Seed in 30 days post-anthesis | Seed in 40 days post-anthesis | Mature Leaf | Ovule | Ovule in anthesis | Ovule in 3 days post-anthesis |
| --- | --- | --- | --- | --- | --- | --- | --- | --- | --- | --- |
| Gorai.001G019600 | 0.0000 | 0.0000 | -0.3565 | -1.0969 | -0.2366 | 0.0000 | 0.3385 | 0.2279 | 0.0000 | -1.1549 |
| Gorai.001G019700 | 1.2030 | 0.9809 | 1.5384 | 1.6852 | 0.9991 | 1.1602 | 2.1410 | 2.5982 | 1.3206 | 2.8504 |
| Gorai.001G019800 | 0.7716 | 0.0000 | 0.7993 | 1.2350 | 1.0577 | 0.6580 | 1.2170 | 1.5339 | 1.1129 | 0.0000 |
| Gorai.001G022700 | 0.1931 | -0.1192 | 0.0569 | 0.4579 | 0.1703 | -0.1308 | 1.1553 | 0.5752 | 0.7679 | 0.0531 |
| Gorai.001G022800 | 0.6875 | 0.8768 | 0.7945 | 0.7490 | 0.7292 | 0.9058 | 1.0216 | 1.0145 | 0.8698 | 0.7042 |
| Gorai.001G022900 | 0.7380 | 1.0402 | 1.2261 | 1.1498 | 1.1553 | 1.7305 | 1.0924 | 1.4060 | 1.1414 | 1.5163 |
| Gorai.001G027600 | 0.0000 | 0.0000 | -1.3010 | -0.6576 | -1.0969 | -0.2757 | 0.1004 | 0.6314 | -0.4685 | 0.0000 |
| Gorai.001G027700 | -0.2291 | -0.2076 | 0.1461 | -0.4437 | -0.4815 | -1.2218 | 0.2175 | 0.9248 | 0.4639 | 0.9445 |
| Gorai.001G027800 | 1.6547 | 1.6517 | 1.6453 | 1.4038 | 1.6144 | 1.7130 | 1.8001 | 1.4850 | 1.3058 | 1.4776 |
| Gorai.001G050200 | 0.0000 | 0.0000 | 0.0000 | 0.0000 | -0.0362 | -0.6198 | 0.9380 | 0.0000 | 0.0000 | 0.0000 |
| Gorai.001G050300 | 0.0000 | 0.0000 | -2.0000 | -0.3565 | -1.6990 | 0.0000 | 0.3674 | -1.3010 | 0.0000 | 0.0000 |
| Gorai.001G050400 | 1.3187 | 1.5411 | 1.2238 | 1.0390 | 0.9763 | 0.8189 | 1.2276 | 1.4651 | 1.6878 | 0.7443 |
| Gorai.001G050500 | 0.0000 | 0.0000 | 0.0000 | 0.0000 | 0.0000 | 0.0000 | 0.7348 | -1.3979 | -1.1549 | 0.0000 |
| Gorai.001G050600 | 0.0000 | 0.0000 | 0.0000 | 0.0000 | 0.0000 | 0.0000 | 0.0000 | -0.6383 | -0.3665 | 0.0000 |
| Gorai.001G052100 | 0.3345 | 0.2430 | 0.5024 | 0.5132 | 0.4548 | 0.8142 | 0.6830 | 0.7627 | 0.7589 | 0.6981 |
| Gorai.001G052200 | 0.4728 | 0.9523 | 0.8176 | 0.6532 | 0.5211 | 0.6243 | 0.3636 | 0.6096 | 0.5988 | 0.5527 |
| Gorai.001G052300 | -0.3565 | 0.6325 | -0.0655 | -0.5229 | -0.5528 | -0.4949 | -1.0000 | -0.0410 | 0.1206 | 0.0000 |
| Gorai.001G052400 | 1.0035 | 1.2697 | 1.2151 | 1.2055 | 1.1775 | 1.1992 | 1.1755 | 1.4057 | 1.1593 | 1.5128 |
| Gorai.001G052500 | 0.9759 | 1.0652 | 1.1436 | 1.1355 | 0.9571 | 1.0973 | 1.3107 | 1.3720 | 0.1399 | 0.0000 |
| Gorai.001G059600 | 0.1959 | 0.1430 | 0.2989 | 0.2014 | 0.3139 | 0.6222 | 1.1109 | 0.7709 | 0.6085 | 0.2455 |
| Gorai.001G059700 | 1.0542 | 1.0766 | 1.3187 | 1.3568 | 1.1523 | 0.9823 | 1.0842 | 1.0414 | 1.0770 | 0.9258 |
| Gorai.001G059800 | 0.0000 | 0.0000 | 0.0000 | 0.0000 | -0.6383 | 0.0000 | -0.8539 | 0.0000 | 0.0000 | 0.0000 |
| Gorai.001G068700 | -0.2076 | -0.0706 | 0.1072 | 0.4166 | -0.2218 | -0.1487 | -0.0655 | -0.0506 | 0.0212 | -0.2218 |
| Gorai.001G068800 | 0.6243 | 0.5079 | 0.3345 | 0.6618 | 0.2480 | -0.1675 | -0.3098 | -0.7696 | -0.5086 | 0.0000 |
| Gorai.001G068900 | 1.3339 | 1.0938 | 1.1163 | 1.0828 | 0.9731 | 0.8971 | 1.8746 | 1.6128 | 1.2735 | 1.7133 |
| Gorai.001G069000 | 0.0531 | 0.3365 | 0.5315 | 0.3655 | 0.6375 | 0.4683 | 0.5051 | -0.0088 | -0.0969 | 0.0086 |
| Gorai.001G069100 | 1.5185 | 1.4932 | 1.5227 | 1.5684 | 1.4562 | 1.7085 | 1.3585 | 1.3004 | 1.3863 | 1.1035 |
| Gorai.001G069200 | 1.3524 | 1.2109 | 1.3276 | 1.2856 | 1.1853 | 1.2923 | 1.3634 | 1.2006 | 1.1300 | 1.2006 |
| Gorai.001G069300 | 0.0000 | -0.0223 | 0.1790 | -0.8861 | 0.0043 | 0.2175 | -0.9208 | 0.0000 | 0.0000 | 0.0000 |
| Gorai.001G069400 | 0.0000 | -0.2757 | 0.0000 | -2.0000 | 0.0000 | 0.0000 | 1.2507 | 0.4698 | -0.3665 | 0.2355 |
| Gorai.001G071900 | -0.7447 | 0.0000 | -1.3010 | 0.0000 | 0.0000 | 0.0000 | 0.4698 | -0.0088 | -1.1549 | 0.2405 |
| Gorai.001G072000 | 0.0000 | -0.0269 | 0.0000 | 0.0000 | -0.3372 | 0.0000 | 1.7164 | 1.6171 | 0.0000 | 0.0000 |
| Gorai.001G072100 | 0.6884 | 0.6693 | 1.1396 | 1.0228 | 0.8062 | 0.2788 | 0.5866 | 1.3012 | 1.3448 | 0.7709 |
| Gorai.001G072200 | 0.7649 | -1.2218 | 0.7910 | 0.1818 | -0.3279 | -1.0000 | 1.0626 | 1.2315 | 1.1981 | 1.1976 |
| Gorai.001G075100 | 1.9790 | 0.7604 | 0.2175 | 1.2041 | 0.4200 | 0.0000 | 1.4949 | 1.8071 | 1.2669 | 0.0000 |
| Gorai.001G075200 | 0.0000 | 0.0828 | -1.2218 | -0.7447 | -0.4089 | -2.0000 | 0.3560 | 0.0569 | 0.0000 | 0.0000 |
| Gorai.001G075300 | -0.2518 | 0.0000 | -0.9208 | 0.0000 | 0.0000 | 0.0000 | 0.7924 | 1.2603 | 0.0000 | 0.0000 |
| Gorai.001G075400 | -0.4089 | 0.4409 | -0.3979 | -0.8861 | -1.1549 | 0.0000 | 0.1303 | 0.0000 | 0.0000 | 0.0000 |
| Gorai.001G087800 | 0.0000 | 0.0000 | -0.6021 | 0.0000 | -0.9208 | 0.0000 | -1.0458 | 0.0000 | 0.0000 | 0.0000 |
| Gorai.001G087900 | 0.0000 | 0.4871 | 0.0000 | 0.5670 | -0.1938 | 0.0000 | 0.0000 | 0.0000 | 0.0000 | 0.0000 |
| Gorai.001G088000 | 0.7846 | -0.0655 | 0.1303 | -0.2218 | 0.6010 | -0.0969 | -0.7447 | 0.0000 | 0.0000 | 0.0000 |
| Gorai.001G088100 | 0.8306 | 1.1501 | 1.1089 | 1.1917 | 1.2014 | 0.8657 | 0.6628 | 0.7520 | 0.8531 | 0.5263 |
| Gorai.001G088200 | 0.7042 | 0.8876 | 0.8820 | 0.8573 | 0.6767 | 0.7882 | 0.9133 | 1.1316 | 1.0945 | 1.1017 |
| Gorai.001G088300 | 1.0839 | 1.1864 | 1.4406 | 1.4069 | 1.4259 | 1.6471 | 1.5224 | 1.2188 | 1.2292 | 1.1367 |
| Gorai.001G089900 | 0.0000 | 0.0000 | -1.5229 | 0.0414 | 0.2175 | 0.4265 | -0.0044 | -1.0969 | -0.8239 | 0.0000 |
| Gorai.001G090000 | 0.0000 | 0.0000 | 0.0000 | 0.0000 | 0.0000 | 0.0000 | -1.0000 | -0.4437 | 0.0000 | 0.0000 |
| Gorai.001G091900 | 0.0000 | 0.0000 | 0.0000 | -1.5229 | -1.3010 | 0.0000 | 0.2529 | -1.0000 | -0.7212 | 0.0000 |
| Gorai.001G094700 | -1.0969 | 0.0000 | -1.3979 | 0.0000 | -0.6021 | -1.3010 | -0.0223 | -0.8861 | -0.6021 | 0.0000 |
| Gorai.001G094800 | 0.0000 | 0.0000 | 0.0000 | 0.0000 | 0.0000 | 0.0000 | -1.0458 | 0.0000 | 0.0000 | 0.0000 |
| Gorai.001G094900 | 0.0000 | 0.0000 | 0.0000 | 0.0000 | 0.0000 | 0.0000 | 0.0000 | 0.0000 | 0.0000 | 0.0000 |
| Gorai.001G096100 | 0.0000 | 0.0000 | 0.1732 | 0.0000 | 0.3927 | 0.6444 | 2.2062 | 1.8534 | 0.0000 | 0.0000 |
| Gorai.001G096200 | 0.0000 | 0.0000 | -0.3372 | 1.2482 | 0.0000 | 0.0000 | 0.4249 | 0.0000 | 0.0000 | 0.0000 |
| Gorai.001G096300 | 0.3747 | -0.2924 | 0.3444 | -1.3979 | -1.2218 | -1.0458 | 0.2068 | 0.5145 | 0.7559 | -0.4202 |
| Gorai.001G096400 | -0.3468 | 0.0453 | -0.2147 | -1.3979 | -1.1549 | -1.0000 | 0.5328 | -0.7696 | -0.4949 | 0.0000 |
| Gorai.001G096500 | 1.0269 | 0.6053 | 1.1014 | 1.0745 | 1.1959 | 1.0302 | 0.4014 | 0.8463 | 1.1163 | 0.0000 |
| Gorai.001G096600 | 0.0000 | 0.0000 | 0.0000 | -0.9208 | 0.0000 | -0.8539 | 0.0000 | 0.0000 | 0.0000 | 0.0000 |
| Gorai.001G105700 | 0.0000 | 0.3284 | 0.0000 | 0.0000 | -0.7959 | 0.0000 | 0.0000 | 0.0000 | 0.0000 | 0.0000 |
| Gorai.001G105800 | 0.0000 | 0.0000 | 0.0000 | 0.0000 | 0.0000 | 0.0000 | 0.0000 | -1.5229 | -1.2218 | 0.0000 |
| Gorai.001G105900 | 0.5211 | 0.5478 | 0.6385 | 0.8142 | 0.7760 | 0.5955 | 0.7701 | 0.8331 | 0.6425 | 0.9138 |
| Gorai.001G106000 | 0.0000 | 0.0000 | 0.0000 | 0.0000 | 0.0000 | 0.0000 | 0.0000 | 0.0000 | 0.0000 | 0.0000 |
| Gorai.001G106100 | 0.0000 | 1.2103 | 0.0000 | 0.0607 | 0.0000 | 0.2672 | 1.1813 | 0.9628 | 0.9047 | 0.9523 |
| Gorai.001G106400 | 0.0000 | -0.1805 | 0.8513 | 0.9934 | 0.8445 | 0.8136 | 0.3404 | -0.3468 | -0.3665 | -0.4089 |
| Gorai.001G106500 | 0.0000 | 0.0000 | 0.0000 | 0.0000 | 0.0000 | 0.0000 | 0.0000 | 0.0000 | 0.0000 | 0.0000 |
| Gorai.001G106600 | 0.1106 | -0.8861 | 0.4065 | -0.6021 | -0.9208 | -0.7696 | -0.7959 | 1.3879 | -0.2518 | 1.3158 |
| Gorai.001G106700 | 0.0000 | 0.0000 | -0.7959 | 0.0000 | 0.0000 | 0.0000 | 0.0000 | -0.2007 | -0.1249 | -0.3872 |
| Gorai.001G106800 | 0.0000 | 0.0000 | 0.0000 | 0.0000 | 0.0000 | 0.0000 | 0.8096 | 1.1287 | -0.1675 | 0.0000 |
| Gorai.001G114400 | -0.1739 | -0.8861 | 0.0607 | -0.2218 | -0.4318 | -0.7212 | -0.7212 | -0.4559 | -0.6198 | -0.3768 |
| Gorai.001G114500 | 1.2343 | 1.5237 | 1.5017 | 1.7019 | 1.8278 | 1.8647 | 1.9327 | 2.0130 | 2.0345 | 1.9171 |
| Gorai.001G114600 | 1.4130 | 1.3556 | 1.4810 | 1.3829 | 1.5877 | 1.2084 | 1.2348 | 0.9917 | 1.1229 | 0.6937 |
| Gorai.001G114700 | 0.5635 | 0.7300 | 0.8451 | 0.8887 | 0.8169 | 0.8000 | 0.6972 | 0.9552 | 0.8681 | 0.9689 |
| Gorai.001G115100 | 0.0000 | -0.8539 | -1.5229 | 0.0000 | -1.5229 | -1.2218 | 0.4314 | -0.6021 | -0.5376 | -0.7447 |
| Gorai.001G116100 | 0.7193 | 0.9703 | 1.1569 | 0.8837 | 1.2335 | 1.6410 | 1.4048 | 1.5938 | 1.1268 | 1.4995 |
| Gorai.001G116200 | 0.7782 | 0.2504 | 0.9741 | 0.7497 | 0.5416 | 0.3962 | 0.6243 | 0.6571 | 0.7627 | 0.4216 |
| Gorai.001G120000 | 0.5575 | 0.0086 | 0.6551 | 0.6637 | 0.8062 | 0.6425 | 0.6493 | 0.4564 | 0.6474 | -0.0555 |
| Gorai.001G120100 | 0.0000 | 0.0000 | -1.5229 | 0.0000 | 0.0000 | 0.7694 | -1.3979 | 0.0000 | 0.0000 | 0.0000 |
| Gorai.001G120200 | -0.3768 | -0.3098 | -0.1938 | 0.1673 | 0.4871 | 1.0888 | 1.2052 | -0.0410 | -0.6778 | 0.1644 |
| Gorai.001G125900 | 0.0000 | 0.0000 | 0.0000 | 0.0000 | 0.0000 | 0.0000 | 0.0000 | 0.0000 | 0.0000 | 0.0000 |
| Gorai.001G126000 | 0.0000 | -0.6778 | 0.0000 | 0.1818 | 0.5551 | 0.4456 | 0.3927 | -1.6990 | -1.3979 | 0.0000 |
| Gorai.001G126100 | 0.0000 | 0.0000 | 0.0000 | 0.0000 | 0.0000 | 0.0000 | 0.0000 | 0.1492 | -0.2518 | 0.3096 |
| Gorai.001G126200 | -0.1024 | 0.0569 | -0.1739 | -0.2076 | -0.3188 | 0.0086 | 0.0170 | 0.5647 | 0.5866 | 0.4683 |
| Gorai.001G126300 | 0.0000 | -0.7696 | 0.0000 | 0.0000 | -0.5686 | 0.0000 | 0.1335 | 0.2201 | -0.7212 | -1.0458 |
| Gorai.001G126400 | 1.2887 | 0.3075 | 1.4071 | 1.5388 | 2.1252 | 2.5507 | 1.0626 | 1.3025 | 1.4014 | 1.0806 |
| Gorai.001G126500 | 0.5105 | 0.0294 | 1.0149 | 0.7451 | 0.5366 | 0.6656 | 1.4870 | 1.1749 | 0.6758 | 1.1062 |
| Gorai.001G126600 | 0.0000 | 0.0000 | -0.3188 | 0.0000 | 0.0000 | 0.0000 | 0.0000 | 0.0000 | 0.0000 | 0.0000 |
| Gorai.001G127000 | -0.0605 | 0.4609 | -0.7447 | 1.3614 | 1.0449 | 0.7868 | 1.3923 | 0.2900 | -0.2007 | 0.4742 |
| Gorai.001G130800 | 0.7664 | 0.5172 | 0.5011 | 0.5478 | 1.0441 | 0.5051 | 1.1984 | 0.4533 | 0.2095 | 0.5587 |
| Gorai.001G130900 | 0.0000 | 0.0000 | 0.0000 | 0.0000 | 0.0000 | 0.0000 | 0.0000 | 0.0000 | 0.0000 | 0.0000 |
| Gorai.001G133700 | 0.8162 | 0.9717 | 0.7945 | 0.6684 | 0.8116 | 0.6946 | 0.6010 | 0.6042 | 0.3096 | 0.7308 |
| Gorai.001G133800 | 0.8987 | 1.5987 | 0.4065 | 0.7709 | 0.6693 | 0.3927 | 0.6415 | 1.0542 | 0.4487 | 1.2567 |
| Gorai.001G135600 | 1.6402 | 1.4864 | 1.9501 | 1.3220 | 0.8222 | 0.8000 | 1.1335 | 1.0457 | 1.0290 | 0.5821 |
| Gorai.001G135700 | 0.0000 | 0.0000 | -2.0000 | -0.4318 | 0.0000 | 0.0000 | 0.8949 | 0.7076 | -0.5376 | 0.5465 |
| Gorai.001G135800 | 1.6322 | 1.3166 | 1.6371 | 1.5888 | 1.3408 | 1.1858 | 1.1495 | 1.0224 | 1.1294 | 0.7832 |
| Gorai.001G136300 | 0.0000 | 0.0000 | -0.6778 | 0.0000 | 0.0000 | 0.3032 | 0.5647 | 0.8949 | 0.0719 | 0.0000 |
| Gorai.001G136400 | 0.0719 | 0.5198 | 0.3945 | 0.5211 | 0.3284 | 0.4082 | 0.3655 | 0.8692 | 0.8669 | 0.8035 |
| Gorai.001G136500 | 0.0000 | 0.0000 | 0.0000 | 0.0000 | 0.0000 | 0.0000 | 0.0000 | 0.0000 | 0.0000 | 0.0000 |
| Gorai.001G136600 | 0.0000 | 0.0000 | 0.0000 | 0.0000 | 0.0000 | 0.0000 | 0.0000 | 0.0000 | 0.0000 | 0.0000 |
| Gorai.001G138600 | -0.0506 | 0.0000 | 0.0000 | -0.4815 | -1.3010 | -1.3010 | 0.0000 | -0.6198 | -0.3468 | 0.0000 |
| Gorai.001G138700 | 0.0000 | 0.0000 | 0.0000 | 0.0000 | 0.0000 | 0.0000 | 0.0000 | 0.0000 | 0.0000 | 0.0000 |
| Gorai.001G138800 | -0.9586 | -0.0458 | -1.3979 | -0.8539 | 0.0000 | 0.0000 | -0.0132 | -0.2007 | 0.0000 | 0.0607 |
| Gorai.001G143300 | 0.0000 | 0.0000 | 0.2227 | 0.4183 | 0.9309 | 1.1446 | 1.0941 | 1.2849 | 0.1399 | 1.0715 |
| Gorai.001G143400 | 0.0000 | 0.0000 | 0.0000 | 0.0000 | 0.0000 | 0.0000 | -0.7447 | -0.4949 | -0.2218 | 0.0000 |
| Gorai.001G148500 | 0.6749 | 1.7024 | 1.2209 | 1.3265 | 1.3208 | 1.0326 | 0.8363 | -0.2518 | -0.2076 | -0.3872 |
| Gorai.001G150200 | -0.8239 | 0.0000 | 0.0000 | -0.2291 | 0.3483 | 0.2330 | 0.8048 | -0.0706 | 0.0000 | 0.0000 |
| Gorai.001G153000 | 1.3992 | 0.9004 | 1.5966 | 1.1801 | 0.8982 | 0.6749 | 0.8779 | 1.2302 | 1.0492 | 1.3054 |
| Gorai.001G153100 | 0.0000 | 0.0000 | 0.0000 | -1.3979 | 0.0000 | -1.6990 | -1.2218 | -1.5229 | -1.2218 | 0.0000 |
| Gorai.001G153200 | 1.4827 | 1.9119 | 1.5247 | 1.6814 | 1.9091 | 1.8551 | 1.8556 | 2.0200 | 1.5393 | 2.1755 |
| Gorai.001G156500 | 0.6893 | 0.7259 | 1.0519 | 1.1984 | 1.0469 | 1.3414 | 0.9232 | 0.5453 | 0.1931 | 0.6920 |
| Gorai.001G156600 | 0.0492 | 0.1931 | 0.3444 | 0.3010 | 0.5877 | -0.1675 | 1.1547 | 1.1183 | 0.5366 | 0.0682 |
| Gorai.001G159400 | 0.0000 | 0.0000 | 0.0000 | -0.4949 | -1.5229 | -1.0969 | 0.0000 | -0.7212 | -0.4559 | 0.0000 |
| Gorai.001G159500 | 0.0000 | 0.0000 | 0.0000 | 0.0000 | 0.0000 | 0.0000 | 0.0000 | -0.7696 | 0.0000 | -0.5086 |
| Gorai.001G168600 | -0.3468 | 0.0000 | 0.0000 | 0.0000 | 0.0000 | 0.0000 | 0.0000 | 0.0000 | 0.0000 | 0.0000 |
| Gorai.001G168700 | 0.1206 | 0.5611 | 0.5899 | 0.5038 | 0.5465 | 0.6990 | 0.5051 | 1.0128 | 0.8338 | 0.8476 |
| Gorai.001G168800 | 0.0000 | 0.0000 | 0.0000 | -0.0044 | 0.0000 | -0.7696 | -0.9208 | 0.0000 | 0.0000 | 0.0000 |
| Gorai.001G170900 | 1.2183 | 2.2279 | 1.2758 | 2.4436 | 2.1518 | 1.8523 | 0.6170 | -0.4685 | -0.2676 | 0.0000 |
| Gorai.001G171000 | 0.0864 | 0.0000 | 0.0000 | -0.7959 | 0.0170 | 0.0000 | 0.6656 | 1.2276 | -0.2596 | 0.0000 |
| Gorai.001G171100 | 0.7649 | 0.0000 | 0.3560 | 0.3385 | 0.0000 | -0.3372 | 1.0278 | 1.0849 | 1.0906 | 1.0086 |
| Gorai.001G171400 | -0.2441 | 0.2742 | 0.3766 | 0.2355 | 0.7185 | 0.2430 | 0.6542 | 0.7356 | 0.4393 | 0.0334 |
| Gorai.001G171500 | 1.0770 | 0.5250 | 0.5185 | 0.1399 | 0.5172 | 0.0969 | 0.0334 | 0.3075 | 0.3502 | 0.1818 |
| Gorai.001G171600 | 0.9047 | 0.8312 | 0.9791 | 0.9159 | 0.8768 | 0.9096 | 1.2435 | 1.0141 | 0.8960 | 0.8261 |
| Gorai.001G172100 | 0.0000 | 0.0000 | 0.0000 | 0.0000 | 0.0000 | 0.0000 | 0.0000 | 0.0000 | 0.0000 | 0.0000 |
| Gorai.001G172200 | 0.0000 | 0.0000 | 0.0531 | 0.0492 | 0.0000 | -1.3979 | -0.1938 | 0.9170 | -0.2518 | 0.9713 |
| Gorai.001G174100 | 0.0000 | -1.3010 | 0.1492 | 0.2380 | -0.4815 | -0.2147 | 1.1313 | 1.2227 | 0.0000 | 0.0000 |
| Gorai.001G174200 | 1.2849 | 1.1892 | 1.1319 | 1.4973 | 1.2167 | 1.3247 | 1.8859 | 1.5408 | 1.2060 | 1.2380 |
| Gorai.001G174300 | 0.0128 | -0.1805 | 0.2041 | 0.3054 | 0.3617 | 0.5302 | 0.5786 | 0.7459 | 0.8267 | 0.5587 |
| Gorai.001G179300 | -0.7696 | -0.1249 | -0.7447 | -0.3665 | -0.9586 | -0.1871 | 1.4550 | 1.2212 | 0.0000 | 0.0000 |
| Gorai.001G179400 | 0.6335 | 0.5224 | 0.5775 | 0.3365 | 0.3243 | 0.3784 | 0.8531 | 0.8609 | 0.7846 | 0.7701 |
| Gorai.001G179500 | -0.0132 | -0.5850 | 0.0374 | 0.2227 | 0.3385 | -0.1427 | 0.4298 | 0.3892 | 0.5988 | -0.2366 |
| Gorai.001G182900 | 0.7275 | 0.8627 | 0.7050 | 0.7427 | 0.5237 | 0.4713 | 0.7789 | 0.8949 | 0.9025 | 0.8162 |
| Gorai.001G183500 | 0.0000 | 0.0000 | 0.0000 | 0.0000 | 0.0000 | 0.0000 | -0.3565 | -0.4815 | -0.4559 | 0.0000 |
| Gorai.001G183800 | 2.3683 | -0.2076 | 1.6574 | 0.7340 | 1.2172 | 0.8370 | 2.4631 | 1.4625 | 1.4229 | 1.4349 |
| Gorai.001G184100 | 0.8075 | 0.0000 | 0.3304 | 0.0000 | -0.0757 | 0.0000 | -0.4318 | -0.6383 | -0.3768 | 0.0000 |
| Gorai.001G184200 | 0.0000 | 0.0000 | 0.0000 | 0.0000 | 0.0000 | 0.0000 | 0.0000 | 0.0000 | 0.0000 | 0.0000 |
| Gorai.001G184300 | -1.2218 | 0.4362 | -1.0458 | 1.5261 | 1.8453 | 1.7919 | 0.0000 | -1.5229 | 0.0000 | 0.0000 |
| Gorai.001G185100 | -0.5376 | -0.3979 | -0.7447 | 0.0000 | -1.3010 | 0.0000 | 0.1703 | 0.4183 | -1.3979 | 0.0000 |
| Gorai.001G185200 | -0.0809 | 0.3201 | 0.4955 | 0.6911 | 0.5888 | 0.2068 | 0.8751 | 0.8102 | 0.7896 | 0.6042 |
| Gorai.001G185300 | 1.6909 | 1.3762 | 1.7455 | 1.7434 | 1.6081 | 1.5805 | 1.6431 | 1.1772 | 1.2256 | 1.0438 |
| Gorai.001G185400 | -0.4685 | 0.0000 | -0.1938 | -0.6778 | -0.2218 | 0.0000 | 0.0000 | -0.6021 | -0.3279 | 0.0000 |
| Gorai.001G185900 | 0.9647 | 0.8109 | 0.9096 | 0.8893 | 1.1329 | 1.7559 | 1.1024 | 0.8686 | 0.8727 | 0.7952 |
| Gorai.001G186000 | 0.8965 | 1.6941 | 0.3181 | 1.1790 | 1.4839 | 1.7860 | 1.1377 | 0.3118 | 0.1271 | 0.3892 |
| Gorai.001G186500 | 0.9961 | 1.0310 | 1.0374 | 0.4771 | 1.0241 | 1.2033 | 1.1430 | 0.5539 | 0.5289 | 0.5119 |
| Gorai.001G186600 | 0.0000 | 0.0000 | 0.0000 | 0.0000 | 0.0000 | 0.0000 | 0.0000 | 0.0000 | 0.0000 | 0.0000 |
| Gorai.001G186700 | 1.8382 | 1.6837 | 1.8821 | 1.7812 | 2.0138 | 1.8809 | 1.3495 | 1.6625 | 1.6071 | 1.6493 |
| Gorai.001G186800 | 0.0000 | 0.0000 | 0.0000 | -0.4089 | 0.0000 | 0.0000 | 0.0000 | 0.6021 | -2.0000 | -0.9586 |
| Gorai.001G186900 | -0.4437 | 0.0000 | -0.5850 | -0.7212 | -0.4685 | -0.2757 | 0.0969 | 0.8987 | 0.0000 | 0.2672 |
| Gorai.001G187900 | 1.6461 | 1.6161 | 1.7576 | 1.7155 | 1.4820 | 1.6166 | 1.8788 | 1.6158 | 1.6921 | 1.4368 |
| Gorai.001G196800 | 0.0000 | -0.1739 | -0.3979 | -0.0605 | -0.8861 | -0.6990 | -0.4437 | 0.0645 | 0.0170 | 0.0414 |
| Gorai.001G202600 | 0.0000 | 0.0000 | -0.4559 | 0.0000 | 0.0000 | 0.0000 | 0.3962 | 0.0000 | 0.0000 | 0.0000 |
| Gorai.001G204000 | 0.0000 | 0.0000 | 0.0000 | -0.9208 | 0.0000 | 0.0000 | 0.0000 | 0.0000 | 0.0000 | 0.0000 |
| Gorai.001G204100 | 0.0000 | 0.0294 | -0.0706 | 0.2455 | 0.3927 | 0.4298 | -0.3979 | -0.7447 | -0.4685 | 0.0000 |
| Gorai.001G204200 | 0.0000 | 0.0000 | 0.0000 | 0.0000 | 0.0000 | 0.0000 | -0.1612 | -0.4089 | 0.0000 | 0.0000 |
| Gorai.001G204300 | 1.6495 | 1.6161 | 1.6438 | 1.5367 | 1.4442 | 1.4615 | 1.2159 | 1.6767 | 1.5395 | 1.7257 |
| Gorai.001G204400 | 0.4609 | 0.4116 | 0.5328 | 1.2125 | 1.1818 | 0.8195 | 1.1377 | 0.7612 | 0.8627 | 0.5328 |
| Gorai.001G206200 | 0.0000 | 0.0000 | 0.0000 | 0.0000 | -1.2218 | -1.0458 | 0.9238 | 1.1973 | 0.0000 | 0.0000 |
| Gorai.001G206300 | -0.7212 | 0.0000 | 0.6274 | 0.7853 | 0.7559 | 0.5955 | 0.8976 | 1.0286 | 0.7275 | 0.7582 |
| Gorai.001G211700 | 1.3675 | 1.1361 | 1.7029 | 0.0000 | 0.0000 | -0.3188 | 0.9685 | -0.0458 | 0.0000 | 0.0000 |
| Gorai.001G211800 | -0.3768 | -0.1549 | -0.1805 | -0.0506 | -0.1549 | -0.0809 | -1.3979 | 0.0000 | 0.0000 | 0.0000 |
| Gorai.001G211900 | 0.0000 | 0.0000 | -0.9208 | -2.0000 | 0.0000 | 0.0000 | 0.3483 | 0.0000 | 0.0000 | 0.0000 |
| Gorai.001G212000 | 0.0000 | 0.0000 | 0.0000 | -0.7212 | 0.0000 | 0.0000 | 0.0000 | 0.0000 | 0.0000 | 0.0000 |
| Gorai.001G212200 | 0.0000 | 0.0000 | 0.0000 | 0.0000 | -1.1549 | 0.0000 | 0.0000 | -0.7959 | -0.5229 | 0.0000 |
| Gorai.001G212300 | 0.0000 | 0.2480 | 0.0000 | 0.0000 | 0.0000 | 0.0000 | 0.0000 | 0.0000 | 0.0000 | 0.0000 |
| Gorai.001G212400 | 0.7701 | 0.9106 | 0.9509 | 1.0237 | 0.9494 | 1.5703 | 0.7987 | 0.9590 | 0.9566 | 0.8927 |
| Gorai.001G212500 | 1.0888 | 1.3181 | 1.6178 | 1.5263 | 1.4971 | 1.0175 | 0.8007 | 1.3570 | 1.0952 | 1.4328 |
| Gorai.001G212900 | 0.0000 | 0.2227 | -1.0458 | -0.4089 | -0.5850 | -1.0969 | -0.2518 | -0.9586 | -0.6990 | 0.0000 |
| Gorai.001G215300 | 0.8445 | 1.6175 | 0.7924 | 1.7752 | 1.5624 | 1.2947 | 0.0000 | 0.0000 | 0.0000 | 0.0000 |
| Gorai.001G215400 | 0.5250 | 0.8494 | 0.4150 | 1.1752 | 0.9708 | 0.7324 | 0.0000 | 0.0000 | 0.0000 | 0.0000 |
| Gorai.001G215500 | -0.4202 | 0.0000 | -0.7212 | -0.3768 | -0.6198 | -0.2676 | 0.5366 | 0.8261 | -0.2757 | -0.2757 |
| Gorai.001G218800 | 0.6191 | 0.6522 | 0.8657 | 0.9465 | 0.8954 | 0.8331 | 1.1355 | 1.1667 | 1.1906 | 1.0674 |
| Gorai.001G218900 | 0.6345 | 0.5276 | 0.7839 | 0.8162 | 0.6138 | 0.4249 | 0.8176 | 0.0531 | 0.2227 | -0.6576 |
| Gorai.001G219000 | 0.4871 | 0.9809 | -0.0088 | 0.2856 | 0.0000 | 0.0000 | 1.9632 | 1.4933 | 0.0000 | 0.0000 |
| Gorai.001G221100 | 0.3892 | 0.4609 | 0.6107 | 0.6821 | 0.5011 | 0.5378 | 0.6884 | 1.1458 | 1.2261 | 0.9595 |
| Gorai.001G221200 | -0.6021 | 0.9304 | -0.7447 | 0.4713 | 0.1703 | -0.0044 | 0.5922 | 0.0000 | 0.0000 | 0.0000 |
| Gorai.001G222900 | 0.0000 | 0.0000 | 0.0000 | 0.0000 | 0.0000 | 0.0000 | 0.0000 | 0.0000 | 0.0000 | 0.0000 |
| Gorai.001G223700 | 1.3235 | 1.0370 | 1.3269 | 1.1483 | 1.4495 | 1.9929 | 1.5280 | 2.6675 | 1.1235 | 2.9007 |
| Gorai.001G223800 | -0.0362 | 0.0000 | -0.1938 | -0.2218 | 0.3874 | -0.0969 | 0.8222 | 0.8470 | 0.3222 | 0.0000 |
| Gorai.001G225400 | 0.0000 | 0.0000 | 0.0000 | 0.0000 | 0.0000 | 0.0000 | 0.0000 | -0.6576 | 0.0000 | 0.0000 |
| Gorai.001G225500 | 0.0000 | -0.5086 | -1.3010 | -0.2757 | -0.2291 | -0.2441 | -0.7447 | -1.3010 | -1.0458 | 0.0000 |
| Gorai.001G225600 | 1.0853 | 1.4411 | 1.2458 | 1.2676 | 1.1886 | 1.1183 | 1.2114 | 1.4055 | 1.3867 | 1.3568 |
| Gorai.001G225700 | 1.9862 | 2.1713 | 2.1390 | 1.9348 | 2.0958 | 2.2692 | 3.4942 | 0.0000 | 0.0000 | 0.0000 |
| Gorai.001G225800 | 0.5428 | 0.6010 | 0.4800 | 0.7380 | 0.9191 | 0.9474 | 0.8331 | 0.5065 | 0.3139 | 0.4014 |
| Gorai.001G225900 | 0.0086 | 0.4409 | 0.2148 | -0.0223 | 0.0086 | 0.2695 | 0.8887 | 0.8287 | 0.7419 | 0.7551 |
| Gorai.001G227700 | 0.0000 | 0.0000 | -0.6021 | -0.4318 | -0.1549 | -0.9586 | 0.7634 | 0.0000 | 0.0000 | 0.0000 |
| Gorai.001G227800 | 0.7980 | 0.5775 | 0.7033 | 0.5441 | 0.7412 | 0.6599 | 0.9930 | 1.0457 | 0.7634 | 1.1380 |
| Gorai.001G227900 | 0.0000 | 0.0000 | 0.0000 | -0.4202 | -0.8239 | 0.1335 | -1.0000 | 0.0000 | 0.0000 | 0.0000 |
| Gorai.001G228000 | 1.5491 | 1.7518 | 1.7953 | 1.7040 | 1.7459 | 1.6589 | 1.2760 | 1.6442 | 1.5710 | 1.6464 |
| Gorai.001G231100 | 0.3404 | 1.2095 | 0.1492 | 1.6911 | 2.3730 | 1.7877 | -1.3979 | -0.3188 | -0.3098 | -0.3979 |
| Gorai.001G231200 | -1.0969 | 0.5051 | -1.2218 | 0.9170 | 1.3927 | 1.7959 | -1.3010 | 0.3502 | 0.5289 | -0.1135 |
| Gorai.001G233900 | 0.2577 | 0.3997 | -0.2147 | -0.0757 | -0.4318 | -0.0605 | 1.9559 | 1.7387 | 0.0000 | 0.0000 |
| Gorai.001G234000 | -1.3979 | -1.6990 | -2.0000 | -1.3979 | -1.6990 | 0.0000 | -0.9586 | -0.2007 | 0.0000 | -0.5528 |
| Gorai.001G234100 | 0.8543 | 0.3010 | 0.7832 | 0.8035 | 1.0686 | 1.0374 | 0.7917 | 1.1092 | 0.9036 | 0.5289 |
| Gorai.001G239700 | -0.1487 | 0.0000 | 0.0000 | 0.1206 | -0.1024 | -0.2366 | -0.0315 | -1.1549 | -0.8861 | 0.0000 |
| Gorai.001G252300 | 0.9315 | 1.4547 | 1.0346 | 0.8791 | 1.1258 | 0.8082 | 1.1761 | 0.7896 | 0.3766 | 0.5966 |
| Gorai.001G252400 | 0.0000 | 0.0000 | 0.0000 | 0.0000 | 0.0000 | 0.0000 | 0.0000 | 0.0000 | 0.0000 | 0.0000 |
| Gorai.001G252500 | 0.0000 | 0.0000 | 0.0000 | 0.0000 | 0.0000 | 0.0000 | 0.0000 | 0.0000 | 0.0000 | 0.0000 |
| Gorai.001G252600 | 0.0000 | 0.0000 | -1.0969 | -1.2218 | -0.6778 | 0.0000 | 0.0000 | -0.3872 | -0.1192 | 0.0000 |
| Gorai.001G252700 | 0.0000 | 0.0000 | 0.0000 | 0.0000 | 0.0000 | 0.0000 | 0.0000 | -0.7696 | -0.5086 | 0.0000 |
| Gorai.001G252800 | 0.0000 | 0.0000 | 0.0000 | -1.0969 | 0.0000 | 0.0000 | 0.0000 | 0.0000 | 0.0000 | 0.0000 |
| Gorai.001G254400 | 1.5083 | 2.1273 | 1.4998 | 1.4268 | 1.3840 | 1.0966 | 1.3583 | 1.4193 | 1.3576 | 1.4118 |
| Gorai.001G254500 | 0.6075 | 0.3729 | 0.3636 | 0.3962 | 0.7574 | 0.8062 | 0.8274 | 0.9101 | -2.0000 | 0.7076 |
| Gorai.001G254600 | 0.0000 | 0.0000 | 0.0000 | -1.2218 | 0.0000 | 0.0000 | 0.0000 | 0.0000 | 0.0000 | 0.0000 |
| Gorai.001G254700 | -0.2676 | -0.0506 | 0.3820 | 0.6848 | 0.8388 | 0.5694 | 0.9274 | 0.6609 | 0.8235 | 0.2672 |
| Gorai.001G254800 | 0.0000 | 0.0000 | -1.3010 | 0.0000 | 0.0000 | 0.0000 | 0.7803 | 0.6232 | 0.0000 | 0.0000 |
| Gorai.001G259000 | 0.8921 | 0.7126 | 0.7210 | 0.9025 | 1.0216 | 0.7980 | 1.1055 | 0.9912 | 0.0000 | 0.0000 |
| Gorai.001G259100 | 0.5198 | 0.2175 | -0.2757 | -0.1938 | -0.7696 | 0.0000 | 1.6425 | 1.2193 | 0.0000 | 0.0000 |
| Gorai.001G259200 | 1.7057 | 1.0864 | 0.6263 | 0.3892 | 0.8779 | 1.3288 | 1.3585 | 1.3780 | 1.5495 | 0.9504 |
| Gorai.001G259300 | -0.0555 | -0.2291 | -0.1192 | -0.3098 | -0.9208 | -0.8861 | -0.4559 | 0.0334 | -0.0458 | 0.0374 |
| Gorai.001G263200 | 0.0000 | 0.0000 | 0.0000 | 0.0000 | 0.0000 | 0.0000 | 0.0000 | -0.5850 | -0.3098 | 0.0000 |
| Gorai.001G263300 | 0.2856 | 0.6160 | 0.7275 | 0.6107 | 0.7738 | 0.5092 | 1.8725 | 2.0487 | 0.5599 | 0.0755 |
| Gorai.001G263400 | 1.7353 | -1.1549 | 0.5763 | 0.0000 | 0.0000 | 0.0000 | 0.0000 | -0.7212 | -0.7212 | -0.7959 |
| Gorai.001G268500 | 1.2603 | 1.6300 | 1.8765 | 1.6759 | 1.9442 | 1.6265 | 1.3086 | 1.4695 | 1.3204 | 1.5260 |
| Gorai.001G268600 | 0.0000 | 0.0000 | 0.0000 | 0.0000 | -0.8539 | -1.5229 | 1.6454 | 0.6222 | 0.6712 | 0.0000 |
| Gorai.001G268700 | 0.0000 | 0.0000 | 0.0000 | -0.7447 | -0.2076 | -0.6198 | 0.0000 | 0.0000 | 0.0000 | 0.0000 |
| Gorai.001G268800 | 0.0000 | -0.2218 | -1.2218 | 0.0000 | 0.0000 | 0.0000 | 1.0785 | 1.0745 | 0.0000 | 0.0000 |
| Gorai.001G268900 | 0.0000 | 0.0000 | 0.0000 | 0.0000 | 0.0000 | 0.0000 | 0.0000 | 0.0000 | 0.0000 | 0.0000 |
| Gorai.001G272200 | 0.3945 | 0.0294 | 0.1732 | 0.5599 | 0.1072 | 0.1239 | -1.5229 | -1.2218 | -0.9586 | 0.0000 |
| Gorai.001G272300 | 0.0000 | 0.0000 | -0.6198 | -0.6778 | 0.0000 | -0.9586 | -0.5686 | -0.8239 | -0.5686 | 0.0000 |
| Gorai.001G272400 | -0.0605 | 0.3054 | 0.2201 | 0.4639 | 0.2765 | 0.0253 | 0.8476 | 0.7924 | 0.8241 | 0.5658 |
| Gorai.001G272500 | 0.0000 | -0.2596 | 0.0000 | -0.1249 | 0.7033 | 0.3747 | -0.5229 | 0.0000 | 0.0000 | 0.0000 |
| Gorai.002G032100 | 1.0561 | 1.3322 | 1.2900 | 1.4059 | 1.2749 | 1.2177 | 1.4926 | 1.3259 | 0.8261 | 1.5097 |
| Gorai.002G032200 | 0.0000 | 0.4579 | -0.9586 | 0.5977 | 0.8716 | 0.8007 | 1.1951 | -0.3010 | -0.0315 | 0.0000 |
| Gorai.002G032300 | 0.3636 | 0.0000 | -0.4685 | -0.3468 | -1.3010 | 0.0000 | -0.8239 | 0.0000 | 0.0000 | 0.0000 |
| Gorai.002G032400 | 0.1239 | 0.2095 | 0.3711 | 0.3674 | 0.2480 | 0.2625 | 0.2810 | 0.7419 | 0.7226 | 0.6937 |
| Gorai.002G032500 | 0.0000 | 0.0334 | 0.2718 | 0.6021 | -0.1675 | -0.3010 | 0.4533 | 0.6201 | 0.7251 | 0.3010 |
| Gorai.002G032600 | 0.0000 | 0.0000 | 0.0000 | -0.5686 | -1.5229 | 0.0000 | -1.0000 | -0.4318 | 0.0000 | -0.1739 |
| Gorai.002G038500 | 0.0000 | 0.0000 | 0.0000 | 0.0000 | 0.0000 | 0.0000 | 0.0000 | -1.3010 | -1.0458 | 0.0000 |
| Gorai.002G038600 | 0.0000 | -0.5229 | 0.0000 | 0.0000 | 0.0000 | 0.0000 | 0.8710 | 0.0000 | 0.0000 | 0.0000 |
| Gorai.002G038700 | 0.7853 | 0.7042 | 0.9263 | 0.8102 | 0.9375 | 0.9335 | 1.0449 | 0.6180 | -0.1192 | 0.4857 |
| Gorai.002G038800 | 0.7042 | 0.0000 | 0.9025 | 1.1758 | 0.5527 | -0.0088 | 1.3440 | 1.4867 | 0.5198 | -2.0000 |
| Gorai.002G063100 | -1.6990 | 0.0000 | 0.0000 | 0.0000 | -2.0000 | -2.0000 | 1.0358 | -2.0000 | 0.0000 | 0.0000 |
| Gorai.002G063200 | 0.2529 | -0.3372 | 0.3201 | -0.0809 | 0.1959 | 0.2577 | 1.5213 | -0.1938 | -0.6198 | -0.0269 |
| Gorai.002G063300 | 1.8524 | 1.7196 | 1.6824 | 1.5693 | 1.4254 | 1.2017 | 1.3755 | 1.5314 | 1.6134 | 1.3416 |
| Gorai.002G063400 | 1.2076 | 1.3402 | 1.2911 | 1.3610 | 1.3195 | 1.5131 | 1.1059 | 1.7415 | 1.6183 | 1.7813 |
| Gorai.002G063500 | 0.2227 | -0.0315 | -0.1739 | -0.1308 | -0.5229 | -1.0000 | 0.7917 | 0.9450 | 0.3522 | -0.3279 |
| Gorai.002G063600 | 0.0000 | 0.0000 | -0.7696 | 0.0000 | 0.0000 | 0.0000 | 0.0000 | 0.0000 | 0.0000 | 0.0000 |
| Gorai.002G063700 | 0.0000 | 0.0000 | 0.0000 | 0.0000 | 0.0000 | 0.0000 | 0.0000 | 0.0000 | 0.0000 | 0.0000 |
| Gorai.002G064400 | -0.0044 | -0.0809 | -0.1079 | 0.2480 | -0.0655 | -0.3565 | 0.4669 | -0.1938 | 0.0170 | -0.7959 |
| Gorai.002G067900 | 0.2625 | 0.7050 | 0.2041 | 0.0334 | 0.1523 | -0.1675 | 0.9494 | 0.7760 | 0.0000 | 0.0000 |
| Gorai.002G068000 | 0.6749 | 0.5378 | 0.9638 | 0.9004 | 1.6065 | 1.4016 | 1.0286 | 1.1380 | 1.0983 | 1.1103 |
| Gorai.002G068100 | -0.2840 | 0.0000 | -0.2676 | -0.5086 | -1.2218 | -1.0969 | 0.0969 | 0.1004 | -0.0132 | 0.1367 |
| Gorai.002G068200 | -0.0269 | -0.3872 | -0.0969 | 0.1761 | 2.0588 | 2.6953 | -1.1549 | 0.4116 | 0.5999 | -0.0915 |
| Gorai.002G068300 | 0.3674 | 0.0607 | 0.0453 | 0.2330 | 1.7168 | 2.3080 | 0.2900 | 0.6998 | 0.8351 | 0.3927 |
| Gorai.002G068400 | -0.1367 | -0.3372 | 0.0334 | 0.5611 | 1.8540 | 3.3096 | 0.8932 | 0.8202 | 1.0124 | 0.3010 |
| Gorai.002G068500 | -1.0969 | -0.3768 | -1.5229 | -1.0969 | 0.1173 | -1.3979 | 0.7084 | 0.9736 | 0.6117 | -0.5229 |
| Gorai.002G082500 | 0.0000 | 0.0000 | 0.0000 | 0.0000 | 0.0000 | 0.0000 | 0.0000 | 0.0000 | 0.0000 | 0.0000 |
| Gorai.002G082600 | -0.0223 | -0.1308 | -0.0706 | -0.0605 | -0.1675 | 0.2601 | 0.6274 | 0.1818 | 0.2227 | -0.3979 |
| Gorai.002G082800 | 0.0000 | 0.0000 | 0.0000 | 0.0000 | 0.0000 | 0.0000 | -0.2676 | 0.0000 | 0.0000 | 0.0000 |
| Gorai.002G082900 | -0.2676 | 0.3655 | -0.2147 | -0.8539 | 0.4728 | -0.0757 | 0.0000 | 0.0000 | 0.0000 | 0.0000 |
| Gorai.002G083000 | 1.6358 | 1.7790 | 1.7752 | 1.4186 | 1.6033 | 1.7305 | 1.9846 | 2.6548 | 2.4503 | 2.4311 |
| Gorai.002G084500 | 0.0000 | 0.0000 | 0.0000 | 0.0000 | 0.0000 | 0.0000 | 0.0000 | -1.5229 | 0.0000 | 0.0000 |
| Gorai.002G084600 | 0.0000 | 0.0000 | -0.8239 | 0.0000 | 0.0000 | 0.0000 | 0.1072 | 0.0000 | 0.0000 | 0.0000 |
| Gorai.002G084700 | -0.5376 | -0.0088 | -0.8539 | -0.0809 | -1.0458 | 0.0000 | 0.0000 | 0.0000 | 0.0000 | 0.0000 |
| Gorai.002G084800 | -0.3768 | 0.6675 | 0.0792 | 0.0899 | -0.2007 | 0.3075 | 0.8621 | 0.5490 | 0.4065 | 0.0000 |
| Gorai.002G084900 | 0.0000 | 0.0000 | 0.0000 | 0.0000 | 0.0000 | 0.0000 | 0.0000 | 0.0000 | 0.0000 | 0.0000 |
| Gorai.002G088400 | -0.4437 | 0.4378 | 0.0492 | 0.0607 | 0.2148 | -0.0555 | 0.7627 | 1.2030 | 0.6794 | 0.6335 |
| Gorai.002G088500 | 0.1038 | 0.0000 | -0.6990 | -0.2441 | -0.9208 | 0.0000 | 1.4965 | 0.8971 | 0.0000 | 0.0000 |
| Gorai.002G088600 | 0.0000 | 0.0000 | -1.3010 | -0.3098 | 0.0000 | 0.0000 | 0.0000 | 0.0000 | 0.0000 | 0.0000 |
| Gorai.002G091700 | 0.0000 | 0.0000 | 0.0000 | 0.0000 | 0.0000 | 0.0000 | 0.0000 | 0.0000 | 0.0000 | 0.0000 |
| Gorai.002G091800 | 0.0000 | 0.0000 | 0.0000 | 0.0000 | 0.0000 | 0.0000 | 0.0000 | -0.4089 | -0.1367 | 0.0000 |
| Gorai.002G091900 | 0.1790 | 0.3997 | -0.1367 | 0.0000 | 0.0000 | 0.0000 | 0.5944 | -0.5376 | 0.0000 | 0.0000 |
| Gorai.002G095700 | 1.5433 | 1.5363 | 1.7028 | 1.5914 | 1.5418 | 1.3302 | 1.8709 | 2.3282 | 1.4527 | -2.0000 |
| Gorai.002G095800 | 0.1818 | -0.1308 | 0.3404 | 0.0212 | 0.1239 | -0.0177 | 0.4065 | 0.5539 | 0.6665 | 0.3010 |
| Gorai.002G095900 | 1.7101 | 1.9251 | 1.1998 | 1.0607 | 0.9628 | 0.8567 | 0.7084 | 0.8457 | 0.6675 | 0.7672 |
| Gorai.002G096000 | 0.0000 | 0.0000 | 0.0000 | 0.0000 | 0.0000 | 0.0000 | 0.0000 | 0.0000 | 0.0000 | 0.0000 |
| Gorai.002G096100 | 0.8609 | 1.4874 | 0.6274 | 0.0682 | -0.1192 | -0.4949 | 0.8561 | 1.7645 | 0.0000 | 0.0000 |
| Gorai.002G096200 | 0.3201 | 0.9600 | 0.4346 | 0.1004 | 0.8445 | 0.9782 | 1.3899 | 1.1274 | 0.5514 | 1.3145 |
| Gorai.002G101700 | -0.0706 | 0.2695 | -0.1079 | 0.0828 | 0.2742 | -0.1871 | 1.0043 | 1.2538 | 0.8241 | 1.0580 |
| Gorai.002G101800 | 1.0120 | 1.1881 | 1.2962 | 1.2949 | 1.2914 | 1.1556 | 0.9850 | 1.3847 | 1.3800 | 1.3206 |
| Gorai.002G101900 | -1.5229 | 0.8651 | 0.9020 | 1.0438 | 0.7903 | 0.6580 | 1.9008 | 1.2355 | 1.2507 | 1.0422 |
| Gorai.002G102800 | 0.0000 | -0.5686 | 0.3997 | -0.8861 | 0.0000 | 0.0000 | -0.2757 | -0.6778 | -0.3979 | 0.0000 |
| Gorai.002G102900 | 1.1082 | 1.1342 | 1.1480 | 1.2009 | 1.2641 | 1.2986 | 1.5088 | 1.0546 | 0.8222 | 1.1553 |
| Gorai.002G103000 | 0.4548 | 2.0077 | 1.2909 | 1.0394 | 1.0481 | 1.2636 | 1.5605 | 1.3493 | 1.4573 | 1.1055 |
| Gorai.002G103100 | 0.0000 | 0.1367 | 0.2041 | 0.2504 | 0.4757 | -0.1805 | 1.3551 | -0.5686 | -0.2924 | 0.0000 |
| Gorai.002G103200 | 0.3560 | 0.0000 | -0.7959 | 0.0719 | 0.0253 | -0.0915 | 0.7589 | 0.7686 | 0.0000 | 0.8825 |
| Gorai.002G103300 | -0.3872 | 0.0000 | 0.2577 | -0.4318 | -0.9586 | -0.5086 | 0.2405 | 0.1875 | 0.3365 | 0.0000 |
| Gorai.002G103400 | 0.3579 | 0.7474 | 0.0531 | 0.4786 | 0.0607 | -0.0362 | 0.1931 | 0.1038 | 0.3054 | -0.4815 |
| Gorai.002G104400 | -0.6778 | -0.8539 | -1.2218 | -1.0000 | -0.3768 | -0.6778 | 0.3655 | -0.8239 | -0.7959 | -0.8861 |
| Gorai.002G106000 | 0.1847 | 0.8998 | 0.5821 | 0.5490 | 0.3365 | 0.4609 | 1.1673 | 0.9122 | 0.0569 | 1.1430 |
| Gorai.002G110600 | 0.0000 | 0.0000 | 0.0000 | 0.0000 | 0.0000 | -2.0000 | -0.7212 | -0.3188 | -0.7959 | -0.5229 |
| Gorai.002G122400 | 1.4544 | 0.9717 | 1.1587 | 0.9872 | 1.1189 | 1.2853 | 1.8392 | 1.9549 | 1.8460 | 1.1973 |
| Gorai.002G122500 | -0.0088 | 0.2253 | 0.2330 | 0.2878 | 0.5051 | 0.3766 | 0.4440 | 0.9165 | 0.3160 | 1.1179 |
| Gorai.002G122600 | 0.0000 | 0.0000 | -0.6383 | 0.0000 | 0.0294 | 0.0000 | 1.0931 | 0.5999 | 0.0000 | 0.0000 |
| Gorai.002G129200 | -1.0969 | -1.1549 | -0.1871 | -0.1612 | -1.5229 | 0.0000 | -1.0969 | -1.0000 | -0.7212 | 0.0000 |
| Gorai.002G132400 | -0.2924 | 1.2776 | -0.7959 | 0.1239 | -0.4202 | 0.2201 | 0.0334 | -1.0458 | -0.7696 | 0.0000 |
| Gorai.002G132500 | 0.0000 | 0.0000 | 0.0000 | -0.7959 | 0.9894 | 0.5977 | 0.5899 | 0.7435 | 0.1004 | 0.9513 |
| Gorai.002G137800 | 0.8248 | 1.1761 | 0.7760 | 0.7520 | 0.5911 | 0.5366 | 1.0853 | 1.3017 | 1.2792 | 1.2567 |
| Gorai.002G137900 | -1.3979 | 0.0000 | 0.1553 | -0.9586 | 0.3160 | -0.0315 | -0.5086 | 1.4800 | 0.0000 | 0.5441 |
| Gorai.002G141400 | 0.4425 | 0.5694 | 0.7459 | 0.8627 | 0.7284 | 1.2487 | 1.9525 | 1.1872 | 1.0350 | 1.2460 |
| Gorai.002G146200 | 1.0082 | 1.2749 | 1.0759 | 0.7067 | 0.5855 | 0.3118 | 0.3766 | 1.1123 | 1.2651 | 0.7520 |
| Gorai.002G147700 | 0.0000 | 0.0000 | 0.0000 | 0.0000 | 0.0000 | 0.0000 | 0.0000 | 1.1535 | 0.7404 | 1.3172 |
| Gorai.002G147800 | 1.2923 | 0.7973 | 2.0168 | 1.4108 | 1.4580 | 2.0169 | 1.5489 | 2.6023 | 0.0000 | 0.0000 |
| Gorai.002G226600 | 0.6075 | 0.9542 | 0.8848 | 0.9274 | 0.9777 | 1.1021 | 1.5714 | 1.0322 | 1.0422 | 0.9509 |
| Gorai.002G228000 | 1.0261 | 0.0000 | 0.6415 | 0.7235 | 0.0000 | 0.0000 | -0.2291 | 0.2330 | 0.0000 | 0.2430 |
| Gorai.002G228100 | 1.2317 | 1.2164 | 1.3235 | 1.2041 | 1.2127 | 1.2693 | 0.9325 | 1.0878 | 1.0795 | 1.0278 |
| Gorai.002G230500 | -0.3468 | -0.1938 | -0.0315 | -0.1079 | -0.3098 | -0.4559 | -0.2924 | -0.0757 | -0.2441 | -0.0088 |
| Gorai.002G230600 | 1.7640 | 1.9000 | 1.8763 | 1.4634 | 1.5904 | 1.3058 | 1.8423 | 1.3533 | 1.2889 | 1.3483 |
| Gorai.002G230700 | 0.0000 | 0.0000 | -1.0969 | 0.0000 | -0.8239 | 0.0000 | 1.2847 | 0.9699 | 0.0000 | 0.0000 |
| Gorai.002G231500 | 0.5391 | 0.6928 | 1.0124 | 0.7364 | 1.0469 | 1.4401 | 0.7709 | 0.1553 | -0.2007 | 0.3032 |
| Gorai.002G231600 | 0.7251 | 1.3379 | 0.8182 | 0.8122 | 1.0306 | 1.1703 | 0.6561 | 0.0864 | 0.1430 | -0.0605 |
| Gorai.002G231700 | -0.9208 | 0.0000 | 0.0000 | 0.0374 | -1.5229 | -1.2218 | 1.0224 | 0.7059 | 0.0000 | -2.0000 |
| Gorai.002G231800 | 2.5414 | 2.2697 | 2.3884 | 2.4277 | 2.1591 | 2.3136 | 2.5153 | 2.3160 | 2.4001 | 2.1229 |
| Gorai.002G231900 | 0.0000 | 0.0043 | 0.6981 | 0.6243 | 0.4900 | 0.6794 | 1.1464 | 1.1867 | 0.0000 | 0.0000 |
| Gorai.002G232900 | 1.1133 | 0.7810 | 1.1998 | 1.1038 | 0.9479 | 1.0924 | 0.9170 | 1.1923 | 1.0792 | 1.2251 |
| Gorai.002G233000 | 0.5092 | 0.3324 | 0.5587 | 0.2480 | 0.3424 | 0.4518 | 0.1903 | 0.2989 | 0.3444 | 0.1673 |
| Gorai.002G233100 | 0.8597 | 1.2519 | 0.5011 | 0.2405 | -0.1024 | 0.3444 | 0.6375 | 0.6821 | -0.6383 | -2.0000 |
| Gorai.002G233200 | 0.6998 | 0.6493 | 0.4249 | 0.6990 | 0.6571 | 0.6590 | 0.7612 | 0.7388 | 0.2148 | 0.9274 |
| Gorai.002G233300 | 0.0000 | 0.0000 | 0.0000 | 0.0000 | 0.0000 | 0.0000 | 0.5490 | 0.0000 | 0.0000 | 0.0000 |
| Gorai.002G235000 | 0.0000 | 0.0000 | 0.0000 | 0.1139 | 0.7275 | -0.3872 | 0.0000 | 0.0000 | 0.0000 | 0.0000 |
| Gorai.002G235100 | 0.9881 | 1.2143 | 1.2403 | 1.2047 | 1.3008 | 1.3952 | 1.4252 | 1.6121 | 1.2284 | 1.7680 |
| Gorai.002G235200 | 1.8955 | 1.5936 | 1.6573 | 1.8392 | 1.7152 | 2.2481 | 1.7424 | 1.6731 | 1.5676 | 1.6724 |
| Gorai.002G235300 | 0.0000 | 0.0000 | 0.0000 | 0.2380 | -0.3768 | 0.0000 | 0.5611 | 1.2175 | 0.6096 | 0.0000 |
| Gorai.002G236100 | 0.0000 | 0.0000 | 0.0000 | -0.0969 | -0.3468 | -0.2840 | -1.0969 | 0.0000 | 0.0000 | 0.0000 |
| Gorai.002G241600 | -0.1308 | -0.8539 | -0.2366 | 0.5224 | 1.0682 | 0.6149 | 0.4997 | 0.2742 | 0.3617 | 0.0755 |
| Gorai.002G256400 | 0.0000 | 0.0000 | 0.0000 | 0.0000 | 0.0000 | 0.0000 | 0.5250 | -1.5229 | 0.0000 | 0.0000 |
| Gorai.002G256500 | 0.0000 | 0.0000 | 0.0000 | 0.0000 | 0.0000 | 0.0000 | -0.4318 | 0.0000 | 0.0000 | 0.0000 |
| Gorai.002G256600 | 0.0000 | 0.0000 | 0.0000 | 0.0000 | 0.0000 | 0.0000 | 0.0000 | 0.0000 | 0.0000 | 0.0000 |
| Gorai.002G256700 | -0.1079 | 0.0000 | -0.0088 | -0.0655 | 0.4409 | 0.2900 | 0.9703 | 0.6170 | 0.7396 | 0.3404 |
| Gorai.002G256800 | 0.0000 | 0.0000 | -0.5086 | -0.4685 | 0.3032 | 0.0682 | 0.6875 | 0.4857 | 0.7566 | 0.0000 |
| Gorai.002G256900 | 0.0294 | -0.3665 | -0.2076 | 0.5416 | 0.8169 | 0.3636 | 1.0580 | 0.6848 | 0.9112 | -0.0655 |
| Gorai.002G257000 | 0.0000 | 0.0000 | -0.3768 | -1.0969 | -0.5376 | -0.0315 | 0.9101 | 1.3585 | 0.0000 | 0.0000 |
| Gorai.002G257100 | -1.0458 | -0.6778 | -1.6990 | 0.0000 | -1.2218 | -0.8861 | 1.2000 | -0.7696 | -1.1549 | -0.6198 |
| Gorai.002G262500 | 0.2095 | 0.5694 | 0.2648 | -0.1135 | 0.1492 | 0.1335 | 0.2810 | 0.1430 | -0.4202 | -0.4202 |
| Gorai.002G262600 | 1.9366 | 1.6333 | 1.9759 | 1.9319 | 1.8631 | 1.3043 | 1.7167 | 1.8743 | 1.9384 | 1.7163 |
| Gorai.002G262700 | 1.5868 | 1.3895 | 1.4823 | 1.3842 | 1.3576 | 1.9037 | 1.8215 | 1.8593 | 1.5654 | 1.5091 |
| Gorai.002G262800 | 0.0000 | 0.0000 | 0.0000 | 0.8055 | 0.6628 | 0.3324 | 0.0000 | -1.3979 | 0.0000 | 0.0000 |
| Gorai.002G262900 | 0.0000 | 0.0000 | 0.0000 | 0.0000 | -1.3010 | 0.0000 | -0.8861 | 0.0000 | 0.0000 | 0.0000 |
| Gorai.002G263000 | 0.0000 | -0.5528 | -1.6990 | -0.0223 | 0.0000 | 0.0000 | 1.1629 | 1.3502 | 0.0000 | 0.0000 |
| Gorai.002G263100 | -0.9208 | -1.0458 | 0.0000 | 0.0000 | -0.0969 | 1.5708 | 1.2651 | -0.1024 | -0.4949 | 0.0531 |
| Gorai.002G263200 | 1.4419 | 1.4325 | 1.4249 | 1.4506 | 1.4495 | 1.5517 | 1.3456 | 1.5252 | 1.4299 | 1.5449 |
| Gorai.003G000500 | 0.0000 | 1.1673 | 0.0000 | 0.0000 | 0.0000 | 0.0000 | 0.5694 | 0.0000 | 0.0000 | 0.0000 |
| Gorai.003G000600 | 0.3692 | 0.6812 | 0.7101 | 0.5403 | 0.5172 | 0.4997 | 1.3955 | 1.2660 | 0.4955 | 0.1038 |
| Gorai.003G000700 | 0.1523 | 0.1847 | 0.5441 | 0.4698 | 0.6513 | 0.9009 | 0.3483 | -0.2757 | -0.0088 | 0.0000 |
| Gorai.003G000800 | 0.5092 | 0.5198 | 0.2430 | 0.0828 | 0.4183 | 0.4757 | 1.0682 | 1.2487 | 0.0000 | 0.0000 |
| Gorai.003G000900 | 0.0000 | 0.0000 | 0.0000 | 0.0000 | 0.0000 | 0.0000 | 0.4200 | 0.0294 | 0.0000 | 0.0000 |
| Gorai.003G001000 | 0.5315 | -0.0605 | 0.2945 | 0.1523 | 0.1931 | 0.4082 | 0.8169 | 1.1801 | 0.5490 | 0.7980 |
| Gorai.003G001400 | 0.0000 | 0.0000 | -1.3979 | 0.0000 | 0.0000 | 0.1173 | 1.4201 | 1.1700 | 0.0000 | 0.0000 |
| Gorai.003G001500 | -0.3565 | 0.1523 | -0.3768 | 0.0128 | -1.5229 | -0.8239 | -0.3279 | 0.3139 | 0.0000 | 0.3874 |
| Gorai.003G001600 | 0.6284 | -0.0315 | 0.9773 | 0.8096 | 0.3075 | 1.0976 | 1.3997 | 1.9279 | 1.2068 | 2.1459 |
| Gorai.003G001700 | 0.2068 | 0.0374 | -0.9586 | -0.8239 | -1.5229 | -2.0000 | -0.6576 | 0.1072 | 0.0000 | 0.0000 |
| Gorai.003G001800 | 0.9605 | 1.4654 | 1.1335 | 0.8887 | 0.7412 | 0.7110 | 1.0777 | 1.3545 | 0.6474 | 0.0492 |
| Gorai.003G001900 | 0.0000 | 0.5740 | 0.3404 | -0.0223 | 0.4487 | 0.3747 | 0.9890 | 1.1758 | 1.0711 | 1.1673 |
| Gorai.003G002000 | 0.4099 | 0.4609 | 0.6532 | 0.5752 | 0.5933 | 0.8779 | 0.8325 | 0.4082 | 0.3909 | 0.3579 |
| Gorai.003G002100 | 0.8293 | 1.0849 | 0.9638 | 1.0592 | 1.0204 | 0.9699 | 1.0484 | 0.9518 | 1.0715 | 0.6345 |
| Gorai.003G002200 | 0.7177 | 1.2240 | 1.3856 | 1.2445 | 1.2721 | 1.3363 | 1.8098 | 1.5587 | 1.6331 | 1.3831 |
| Gorai.003G002300 | 0.1732 | 0.0000 | -0.6383 | -0.2366 | -0.8239 | 0.0000 | 0.3385 | -0.0757 | -0.2291 | 0.0000 |
| Gorai.003G002400 | 0.0212 | 0.2480 | 0.1523 | 0.6160 | 0.4298 | 0.1847 | 0.3284 | 0.2405 | 0.1847 | 0.2279 |
| Gorai.003G002500 | 1.1664 | 0.6042 | 0.5263 | 1.2533 | 1.0648 | 0.7474 | 0.2068 | 0.6325 | 0.7251 | 0.3243 |
| Gorai.003G002600 | 0.7664 | 0.6128 | 0.6253 | 1.0175 | 0.9294 | 0.5843 | 1.1928 | 1.3008 | 1.5403 | 0.3997 |
| Gorai.003G002700 | 0.0000 | 0.4564 | -0.0177 | -0.0862 | -0.3979 | -0.2441 | 1.2170 | 0.9894 | 0.9243 | 0.9850 |
| Gorai.003G002800 | -0.5686 | -0.6576 | -0.6383 | -0.3565 | -0.2924 | -0.6778 | 2.2768 | -0.2366 | 0.0000 | 0.0294 |
| Gorai.003G006600 | 0.7767 | 1.4631 | 0.9154 | 0.9106 | 1.1339 | 0.8785 | 0.8035 | 0.9320 | 1.1523 | 0.2330 |
| Gorai.003G006700 | 1.2598 | 1.1909 | 1.5710 | 1.5047 | 1.4371 | 2.0568 | 1.5861 | 1.1329 | 1.1593 | 1.0302 |
| Gorai.003G006800 | 1.0026 | 1.1342 | 1.0492 | 1.0966 | 1.1553 | 1.4354 | 0.7716 | 1.1443 | 1.1775 | 1.0322 |
| Gorai.003G006900 | 1.1981 | 1.6004 | 0.9175 | 1.0500 | 0.9058 | 0.5944 | 0.6532 | 0.7443 | 0.0000 | 0.0000 |
| Gorai.003G007500 | 0.0086 | -0.0809 | 0.0043 | 0.5366 | -0.2007 | -0.3468 | 0.6345 | 0.7093 | 0.9258 | 0.0414 |
| Gorai.003G007600 | 0.0000 | 0.0000 | 0.0000 | 0.1271 | 0.0000 | 0.0000 | 2.0066 | -0.6021 | 0.0000 | 0.0000 |
| Gorai.003G007700 | 0.0000 | 0.0000 | 0.0000 | -0.0555 | 0.0000 | 0.0000 | 0.0000 | 0.0000 | 0.0000 | 0.0000 |
| Gorai.003G007800 | -0.4437 | 0.0000 | -0.8861 | -0.1612 | -0.4949 | -0.4559 | 0.0000 | -1.2218 | -0.9586 | 0.0000 |
| Gorai.003G007900 | 1.0073 | 0.7300 | 0.8727 | 0.5821 | 0.8657 | 1.0183 | 1.3606 | 1.5916 | 1.5925 | 1.5096 |
| Gorai.003G008000 | 0.0719 | -0.2596 | 0.0086 | 0.3160 | 0.3201 | 0.1106 | -0.0706 | 0.1492 | 0.2330 | -0.0458 |
| Gorai.003G008100 | 0.2672 | 0.5145 | 0.8182 | 1.1746 | 1.3126 | 0.8825 | 0.2601 | 1.0086 | 1.2271 | 0.3201 |
| Gorai.003G008200 | -0.2076 | 0.1761 | -0.7959 | 0.0000 | -0.1367 | -0.7959 | 0.6096 | -0.2366 | 0.0000 | 0.0000 |
| Gorai.003G008300 | 1.5480 | 1.7284 | 1.6892 | 1.5077 | 1.6310 | 1.6870 | 2.0375 | 1.8747 | 1.5867 | 1.3531 |
| Gorai.003G008400 | -2.0000 | -2.0000 | 0.0000 | -2.0000 | -1.1549 | -1.6990 | -0.0555 | 0.1206 | 0.0000 | 0.0334 |
| Gorai.003G008500 | 0.4409 | 0.7597 | 0.4487 | 0.5428 | -0.0969 | 0.0000 | 0.9956 | 1.1209 | 1.0952 | 1.0795 |
| Gorai.003G009200 | -0.0410 | 0.0000 | 0.7760 | -0.1805 | 0.2810 | 0.1206 | -0.0506 | 0.9031 | 1.0759 | 0.4713 |
| Gorai.003G009300 | 0.6522 | 0.8831 | 1.0934 | 1.4315 | 1.5185 | 1.2711 | 2.0799 | 1.3657 | 0.3729 | 1.6061 |
| Gorai.003G009400 | 0.3927 | 0.3674 | 0.6160 | 0.6513 | 0.5877 | 0.5539 | 0.2148 | 0.4314 | -0.0044 | 0.6010 |
| Gorai.003G009500 | 0.7451 | 1.2751 | 0.3838 | 0.4757 | 0.4362 | 0.3444 | 0.9854 | 1.0596 | 0.5966 | 0.6284 |
| Gorai.003G009600 | -0.4202 | 0.6010 | 0.2201 | 1.1225 | 0.5289 | 0.2253 | 0.9079 | 0.9355 | 0.8021 | 0.0000 |
| Gorai.003G011900 | 0.8028 | 0.3075 | 1.2219 | 1.1723 | 1.1898 | 0.9680 | 1.2317 | 1.0641 | 1.2620 | 0.5132 |
| Gorai.003G012000 | 0.2380 | -0.5528 | 0.0792 | 0.1523 | 0.0128 | 0.2355 | 0.3692 | 0.1847 | 0.3075 | -0.0915 |
| Gorai.003G012100 | 0.0000 | 0.2553 | 0.3838 | 0.8407 | 1.0565 | 1.2172 | 1.0592 | 0.4786 | 0.0000 | 0.6542 |
| Gorai.003G012200 | 1.7059 | 1.7203 | 1.6989 | 1.6437 | 1.5240 | 1.6492 | 1.8345 | 1.7730 | 1.7476 | 1.7312 |
| Gorai.003G012300 | 0.0000 | -1.3010 | -2.0000 | 0.0000 | 0.0000 | 0.0000 | -0.7212 | 0.0000 | 0.0000 | 0.0000 |
| Gorai.003G014500 | 0.0000 | 0.0000 | 0.0000 | 0.0000 | 0.0000 | 0.0000 | 0.7267 | -0.2007 | 0.0000 | 0.0000 |
| Gorai.003G014600 | 0.0000 | 0.0000 | -0.1739 | -0.7212 | 0.0000 | 0.0000 | -0.4815 | 0.0569 | -0.4949 | 0.2504 |
| Gorai.003G014700 | 0.2330 | 0.4742 | 0.6675 | 0.3927 | 0.7284 | 0.4487 | -0.2441 | 1.0013 | 0.7356 | 0.8439 |
| Gorai.003G014800 | 0.4928 | 1.0993 | 0.9868 | 1.1732 | 0.9159 | 0.9004 | -0.2441 | 0.5378 | 0.4518 | 0.5502 |
| Gorai.003G014900 | 0.8768 | 1.0663 | 0.7738 | 0.8686 | 1.2063 | 0.7987 | -0.2676 | 0.1987 | 0.3729 | -0.2441 |
| Gorai.003G015000 | 0.8306 | 0.5911 | 0.8768 | 0.8482 | 0.4378 | 0.6085 | 0.7443 | 1.0477 | 1.0945 | 0.9165 |
| Gorai.003G015100 | 0.3711 | -0.1308 | 0.0170 | 0.4728 | 1.0449 | 1.3218 | -0.3098 | 0.2810 | 0.2878 | 0.2041 |
| Gorai.003G016400 | 0.0000 | -2.0000 | 0.8325 | 0.8591 | -0.0362 | 0.7497 | 1.6497 | 1.3197 | 0.8537 | -1.3010 |
| Gorai.003G016500 | -1.1549 | -0.5086 | -0.5376 | -0.1612 | -0.7696 | -0.8861 | 0.1553 | -0.0088 | 0.1703 | -0.4685 |
| Gorai.003G016600 | 0.2430 | 0.0000 | 1.4019 | 1.3856 | -0.8861 | -0.2596 | -0.1308 | 2.7032 | 0.9666 | 2.9636 |
| Gorai.003G016700 | 1.2405 | 1.2755 | 0.9841 | 0.8859 | 0.8871 | 1.0103 | 0.7380 | 0.8062 | 0.4548 | 0.9523 |
| Gorai.003G023300 | 0.0000 | 0.0000 | 0.0000 | 0.0000 | 0.0000 | 0.0000 | 0.0000 | 0.0000 | 0.0000 | 0.0000 |
| Gorai.003G023400 | 0.7007 | 0.1106 | 0.9886 | 1.1514 | 0.8960 | 0.8463 | 0.9991 | -0.2366 | -0.1612 | -0.4202 |
| Gorai.003G030900 | 0.5763 | -0.4089 | 0.8692 | 0.9571 | 1.0418 | 1.0414 | 0.7738 | 0.9380 | 0.6474 | 0.6484 |
| Gorai.003G031000 | 1.2867 | 1.5012 | 1.4433 | 1.4887 | 1.6339 | 2.0363 | 1.6802 | 1.8274 | 1.6981 | 1.8713 |
| Gorai.003G031100 | 1.7192 | 0.5955 | 1.2739 | 0.7093 | 0.0899 | 0.0899 | 1.3766 | 1.2049 | 1.0310 | 1.2758 |
| Gorai.003G031200 | 0.0000 | 0.0000 | -0.7212 | 0.2989 | 0.1367 | -0.8861 | -0.0605 | -0.0088 | -0.3665 | 0.1367 |
| Gorai.003G031300 | 0.0000 | 0.0000 | -1.3979 | 0.0000 | -1.3979 | 0.0000 | -1.3979 | -0.7696 | -0.4949 | 0.0000 |
| Gorai.003G038800 | 1.4161 | 1.3844 | 1.3147 | 1.1335 | 0.7419 | 0.4786 | 0.7810 | 1.4784 | 1.5149 | 1.3579 |
| Gorai.003G038900 | 0.0000 | -0.5376 | 0.0000 | -1.1549 | -2.0000 | -2.0000 | -1.0000 | -0.8539 | -1.5229 | -0.6576 |
| Gorai.003G039000 | 0.2430 | 1.1235 | 0.5694 | 0.6646 | 0.7284 | 0.8506 | 0.4393 | 0.7612 | 0.3636 | 0.9212 |
| Gorai.003G039100 | 0.0000 | -0.2441 | -0.8861 | -1.0000 | -0.7212 | -0.6021 | -0.6021 | 0.0000 | 0.0000 | 0.0000 |
| Gorai.003G039700 | 0.0000 | 0.0000 | 0.0000 | 0.0000 | 0.0000 | 0.0000 | 0.0000 | 0.0000 | 0.0000 | 0.0000 |
| Gorai.003G047100 | 0.7126 | 0.2923 | 1.1738 | 0.9445 | 0.3892 | 0.5647 | 1.5252 | 1.4203 | 1.3555 | 1.3428 |
| Gorai.003G047200 | 0.0000 | 0.0000 | 0.0000 | 0.0000 | 0.0000 | 0.0000 | 1.5097 | 0.0334 | 0.0000 | 0.2989 |
| Gorai.003G058400 | -0.2840 | -0.6576 | -0.5086 | -0.2840 | -0.4318 | -0.2007 | 0.9727 | 1.1962 | 0.0000 | 0.0000 |
| Gorai.003G058500 | 0.9365 | 0.8982 | 0.9899 | 0.8494 | 0.8388 | 0.9206 | 0.9628 | 0.9991 | 1.0133 | 0.9128 |
| Gorai.003G058600 | 1.0358 | 1.8312 | 1.2453 | 1.6587 | 1.5774 | 1.3290 | 1.5001 | 1.7270 | 1.4571 | 1.8440 |
| Gorai.003G072300 | 0.0000 | -0.6990 | -0.4318 | -0.6021 | -0.4949 | 0.0000 | 0.7284 | 0.5966 | 0.0000 | 0.4871 |
| Gorai.003G083700 | 0.1959 | 0.7694 | 0.6848 | 0.4900 | 0.9823 | 1.0803 | 0.5024 | 1.2297 | 0.0000 | 1.3690 |
| Gorai.003G083800 | -0.8239 | -0.8861 | -0.7212 | -0.6990 | 0.0000 | 0.0000 | -0.8861 | 0.0000 | 0.0000 | 0.0000 |
| Gorai.003G083900 | -1.0000 | 0.0000 | -1.6990 | -0.6383 | -0.6990 | -1.0969 | -0.2840 | -0.7959 | -0.8239 | 0.0000 |
| Gorai.003G088400 | -0.1739 | 0.0000 | 0.2430 | 0.3483 | 0.1399 | 0.2095 | 1.0073 | 0.9395 | 0.0000 | 0.0000 |
| Gorai.003G088500 | 0.0000 | 0.0000 | 0.0000 | -0.4559 | 0.0000 | 0.0000 | 0.0000 | 0.0000 | 0.0000 | 0.0000 |
| Gorai.003G090500 | -0.6198 | 0.0000 | -0.0223 | -0.4202 | -0.5686 | 0.0043 | -0.3665 | 0.9969 | 0.5185 | 1.1764 |
| Gorai.003G093000 | 0.0000 | 0.0000 | 0.0000 | 0.0492 | 0.0000 | 0.0000 | 0.0000 | 0.0000 | 0.0000 | 0.0000 |
| Gorai.003G093100 | 0.1461 | 0.6170 | 0.3541 | 0.4314 | 0.6010 | 1.0874 | 1.4106 | 0.8287 | 0.6590 | 0.8971 |
| Gorai.003G098800 | 0.0128 | -0.4949 | 0.1584 | 0.1847 | 0.3263 | 0.0294 | 1.1755 | 0.9566 | 1.0618 | 0.7210 |
| Gorai.003G098900 | 1.5914 | 1.9795 | 0.6730 | 0.6053 | 0.6201 | -0.1308 | 0.3365 | 0.6656 | 0.6160 | 0.6464 |
| Gorai.003G099000 | 0.0000 | 0.0000 | 0.0000 | 0.0000 | 0.0000 | 0.0000 | 0.0000 | 0.0000 | 0.0000 | 0.0000 |
| Gorai.003G109100 | 0.1335 | 0.3160 | 0.4683 | 0.5011 | 0.2529 | 0.0128 | 0.8716 | 0.7497 | 0.6794 | 0.7497 |
| Gorai.003G109200 | -0.0044 | 0.3075 | -0.3468 | 0.6767 | 0.7427 | 0.5599 | -0.7447 | 0.0086 | 0.1206 | -0.2441 |
| Gorai.003G111000 | 0.3502 | 0.5911 | 0.6739 | 0.6274 | 0.5775 | 0.6335 | 0.9380 | 0.3636 | 0.4983 | 0.0531 |
| Gorai.003G111100 | 0.0000 | 0.0000 | 0.0000 | -0.5229 | 0.0000 | 0.0000 | 0.0000 | 0.0000 | 0.0000 | 0.0000 |
| Gorai.003G111200 | 0.0000 | -0.6778 | 0.0000 | -1.0000 | -1.2218 | 0.0000 | 0.0000 | -0.9208 | -0.6576 | 0.0000 |
| Gorai.003G111800 | 0.0000 | 0.0000 | 0.0000 | 0.0000 | -1.1549 | 0.0000 | 1.1744 | -0.3768 | 0.0000 | -0.1135 |
| Gorai.003G111900 | 0.3139 | 0.0000 | -1.5229 | 0.0000 | 0.0000 | 0.0000 | -0.1427 | 0.0000 | 0.0000 | 0.0000 |
| Gorai.003G118900 | 0.0000 | 1.3220 | -0.3098 | 0.4886 | 0.7924 | -0.0223 | 1.0162 | 1.2315 | 0.0000 | 1.2079 |
| Gorai.003G119000 | 0.0000 | 2.9005 | -1.3010 | 0.6637 | 0.7067 | 0.8062 | 0.6314 | 0.2504 | 0.0000 | 0.4997 |
| Gorai.003G119100 | 0.8722 | 1.2256 | 0.9232 | 1.1726 | 1.0496 | 0.8927 | -0.0088 | 0.7251 | 0.7067 | -1.6990 |
| Gorai.003G119200 | 0.2945 | 0.1614 | 0.5933 | 0.1818 | 0.6785 | 1.0382 | 1.2343 | 0.9768 | 0.5224 | 0.2856 |
| Gorai.003G119300 | 0.0000 | 0.6375 | 0.0000 | 0.0000 | 0.0000 | 0.0000 | 1.3959 | 0.7536 | 0.5775 | 0.0000 |
| Gorai.003G126400 | 1.0934 | 0.6981 | 0.8710 | 0.6375 | 0.7016 | 1.0290 | 1.6808 | 1.5656 | 1.5345 | 1.2973 |
| Gorai.003G126500 | 1.6097 | 1.2882 | 1.4958 | 1.5722 | 1.4742 | 1.5709 | 1.7680 | 1.5514 | 1.6601 | 1.2860 |
| Gorai.003G126600 | -0.4949 | -0.2366 | 0.5340 | 0.0645 | 0.2672 | -1.0458 | 1.2507 | 0.9227 | 0.3927 | -0.2147 |
| Gorai.003G126700 | -1.0969 | 0.0000 | 0.0000 | 0.0000 | 0.0000 | 0.0000 | 0.4857 | 1.2960 | 0.0000 | 0.0000 |
| Gorai.003G132600 | -0.2924 | 0.0000 | 0.0000 | 0.0000 | -0.9586 | 0.0000 | 0.6425 | -0.9586 | 0.0000 | 0.0000 |
| Gorai.003G143700 | 0.0000 | 0.3747 | -0.1739 | -0.7212 | -0.1192 | -0.2291 | 1.2159 | 0.9547 | 1.0111 | 0.0000 |
| Gorai.003G143800 | 0.0000 | 0.0000 | -0.9208 | 0.0000 | -0.3279 | -0.0809 | 0.6170 | 0.8785 | 0.0000 | -2.0000 |
| Gorai.003G154200 | 0.4472 | 0.1303 | 0.2175 | 0.0414 | -0.0458 | 0.0000 | 1.2368 | 1.0013 | 0.6138 | 0.7924 |
| Gorai.003G154300 | 0.3838 | 0.0000 | 0.4800 | 0.2742 | 0.0000 | -0.3565 | 1.5715 | 1.8252 | 0.4314 | 0.1818 |
| Gorai.003G154400 | -0.7212 | -0.1805 | -0.3188 | 0.0000 | -1.1549 | -1.1549 | 1.1129 | 1.3353 | 0.5527 | 0.1106 |
| Gorai.003G154500 | 0.0000 | 0.0000 | 0.0000 | 0.0000 | 0.0000 | 0.0000 | 0.0000 | 0.0000 | 0.0000 | 0.0000 |
| Gorai.003G154600 | 1.2345 | 0.4487 | 1.0253 | 1.0535 | 0.9542 | 1.0656 | 1.3615 | 1.2707 | -0.1135 | -0.2076 |
| Gorai.003G155400 | -0.0706 | 2.1358 | -0.8539 | 0.7796 | -0.8539 | -1.0969 | 0.3032 | 0.0000 | 0.0000 | 0.0000 |
| Gorai.003G155500 | 2.4152 | 2.4536 | 2.1314 | 2.1174 | 2.0070 | 2.1639 | 1.7826 | 1.2101 | 1.0515 | 1.2723 |
| Gorai.003G155600 | -0.3098 | -0.4318 | -0.6198 | 0.9479 | -0.3979 | 1.8201 | -1.3010 | -0.4437 | -0.3979 | -0.5686 |
| Gorai.003G155700 | 0.3598 | 0.1644 | 0.5729 | -0.9208 | 0.2718 | 0.1761 | 1.2345 | 1.0386 | 0.8915 | 0.6964 |
| Gorai.003G155800 | 1.0124 | 0.3522 | 1.4000 | 1.2550 | 1.1348 | 1.2851 | 1.2536 | 1.6455 | 1.4257 | 1.4270 |
| Gorai.003G155900 | 0.9926 | 0.5635 | 1.0920 | 1.1186 | 0.9227 | 0.6212 | 1.0073 | 1.0973 | 1.1909 | 0.8854 |
| Gorai.003G161300 | 0.0000 | -0.8861 | -1.5229 | 0.0000 | 0.0000 | 0.0000 | 0.0000 | 0.0000 | 0.0000 | 0.0000 |
| Gorai.003G161400 | 0.0000 | 0.0000 | 0.0000 | 0.0000 | 0.0000 | 0.0000 | 0.0000 | 0.0000 | 0.0000 | 0.0000 |
| Gorai.003G161500 | 0.0000 | 0.0000 | 0.0000 | 0.0000 | -0.7447 | -1.0969 | 0.0000 | 0.0000 | 0.0000 | 0.0000 |
| Gorai.003G161600 | 0.0000 | 0.0000 | 0.0000 | 0.0000 | 0.0000 | 0.0000 | 0.0000 | 0.0000 | 0.0000 | 0.0000 |
| Gorai.003G161700 | 0.0000 | 0.0000 | 0.0000 | 0.0000 | 0.0000 | 0.0000 | 0.0000 | 0.0000 | 0.0000 | 0.0000 |
| Gorai.003G161800 | 0.0000 | 0.0000 | 0.0000 | 0.0000 | 0.0000 | 0.0000 | 0.0000 | 0.0000 | 0.0000 | 0.0000 |
| Gorai.003G161900 | 0.0000 | 0.0000 | 0.0000 | 0.0000 | 0.0000 | -1.0969 | 0.0000 | 0.0000 | 0.0000 | 0.0000 |
| Gorai.003G162000 | 0.7210 | 0.4548 | 1.1278 | 1.0090 | 1.0191 | 0.7767 | 1.4826 | 1.8388 | 1.8067 | 1.8038 |
| Gorai.003G163700 | -0.0862 | -0.0362 | -0.3565 | -0.5229 | -0.4089 | -0.4949 | -0.1938 | -0.2218 | -0.4685 | -0.1192 |
| Gorai.003G163800 | 1.2594 | 2.3612 | 1.4371 | 1.9263 | 1.9563 | 1.6509 | 1.5936 | 1.6747 | 0.9818 | 1.8273 |
| Gorai.003G163900 | -0.6990 | 0.0000 | 0.0000 | 0.0000 | 0.0000 | -0.2291 | 1.4837 | 0.0000 | 0.0000 | 0.0000 |
| Gorai.004G034000 | 1.5964 | 1.6987 | 1.6696 | 1.7356 | 1.7223 | 1.9255 | 1.5315 | 1.6546 | 1.7107 | 1.5093 |
| Gorai.004G034100 | 0.0000 | 0.0000 | 0.0000 | 0.0000 | 0.0000 | 0.0000 | 0.0000 | -1.3979 | -1.0969 | 0.0000 |
| Gorai.004G034200 | 0.0000 | 0.5933 | -0.2676 | -0.2007 | -0.1805 | -0.1427 | 0.9304 | 1.0962 | 0.7896 | 0.9703 |
| Gorai.004G034300 | 2.1402 | 2.3689 | 2.2518 | 2.2143 | 2.3695 | 2.4560 | 1.8900 | 2.1205 | 2.1442 | 1.9252 |
| Gorai.004G034400 | 0.5599 | 0.7987 | 0.2355 | 0.2648 | -0.1938 | -0.5376 | -0.0915 | 0.7126 | 0.6107 | 0.7380 |
| Gorai.004G034500 | 0.3560 | 0.6425 | 0.4564 | 0.5159 | 0.4609 | 0.2923 | 0.4116 | 0.7135 | 0.8932 | 0.2529 |
| Gorai.004G035600 | -0.5850 | -0.4685 | -0.0915 | 0.3997 | -0.7447 | 0.6964 | 0.0645 | 0.3784 | 0.6191 | -0.5376 |
| Gorai.004G035700 | 1.0986 | 1.1810 | 1.1048 | 1.0158 | 0.8457 | 0.7152 | 1.1196 | 0.9657 | 1.0302 | 0.7896 |
| Gorai.004G035800 | 0.0000 | -0.2076 | 0.1875 | -0.1249 | 0.5172 | 0.6010 | 1.7279 | 0.9106 | 0.7889 | 0.6503 |
| Gorai.004G035900 | 0.0000 | 0.6812 | 0.4871 | 0.4624 | 0.5065 | 0.7987 | 1.8404 | 1.5808 | -0.2924 | 0.0000 |
| Gorai.004G037200 | 1.6035 | 1.7334 | 1.6780 | 1.6860 | 1.4055 | 1.5116 | 1.6568 | 1.3383 | 1.3655 | 1.2350 |
| Gorai.004G037300 | -0.6778 | 0.0000 | -1.0458 | -1.3979 | -1.3979 | 0.0000 | -0.2518 | -1.3979 | -1.1549 | 0.0000 |
| Gorai.004G037400 | -0.3872 | 0.0000 | -0.1938 | 0.0000 | -1.6990 | -0.9586 | 0.0000 | -0.4089 | 0.0000 | 0.0000 |
| Gorai.004G037500 | 0.9289 | 0.7716 | 0.9859 | 0.9090 | 0.7202 | 0.5145 | 0.4314 | 1.0896 | 0.9741 | 1.1242 |
| Gorai.004G037600 | 0.4099 | -0.6990 | -0.3098 | -0.7447 | -0.8239 | 0.0000 | -1.6990 | -0.6383 | -0.6990 | -0.6383 |
| Gorai.004G037700 | -0.1549 | 0.0086 | -0.0362 | -0.3010 | -0.3098 | -0.1024 | 0.6415 | -0.1675 | -0.1135 | 0.0000 |
| Gorai.004G046500 | 0.0000 | 0.0000 | 0.2201 | -0.1427 | 0.4914 | 0.1818 | 1.1529 | 1.2209 | 0.3324 | 0.7118 |
| Gorai.004G046600 | 0.7738 | 0.6561 | 0.8482 | 0.8675 | 0.8591 | 0.6893 | 1.2550 | 1.6597 | 1.1617 | 1.1830 |
| Gorai.004G046700 | 1.0813 | 0.8837 | 1.1287 | 1.0445 | 0.9304 | 1.2620 | 1.1867 | 1.2519 | 1.2355 | 1.1844 |
| Gorai.004G046800 | 0.0000 | 0.0000 | 0.0000 | 0.0000 | 0.0000 | 0.0000 | 0.0000 | 0.0000 | 0.0000 | 0.0000 |
| Gorai.004G046900 | 0.0000 | 0.0000 | 0.0000 | 0.0000 | 0.0000 | -0.6576 | 0.0000 | 0.0000 | 0.0000 | 0.0000 |
| Gorai.004G047000 | 0.0000 | 0.0000 | 0.0000 | 0.0000 | 0.0000 | 0.0000 | 0.0000 | 0.0000 | 0.0000 | 0.0000 |
| Gorai.004G047100 | 0.5729 | 0.7050 | 0.7059 | 0.7340 | 0.6375 | 0.8136 | 0.4669 | 0.8182 | 0.9284 | 0.5729 |
| Gorai.004G047200 | 0.9638 | 0.6542 | 1.3284 | 1.0342 | 1.0370 | 0.9533 | 0.9727 | 1.4255 | 0.6656 | 0.7566 |
| Gorai.004G049000 | 0.0000 | -0.9208 | -1.5229 | -0.7696 | -0.0915 | -1.3010 | 1.0048 | 0.0000 | 0.0000 | 0.0000 |
| Gorai.004G049100 | 0.0000 | 0.0000 | 0.0000 | 0.0000 | 0.0000 | -1.1549 | 0.0000 | 0.0000 | 0.0000 | 0.0000 |
| Gorai.004G049200 | 0.0000 | 0.0000 | 0.0000 | 0.0000 | 0.0000 | -1.2218 | 0.0000 | -1.3979 | -1.1549 | 0.0000 |
| Gorai.004G049300 | 1.8058 | 1.9919 | 1.8706 | 1.9684 | 1.7761 | 1.8468 | 2.0621 | 2.1606 | 2.2948 | 1.8548 |
| Gorai.004G049400 | 1.2274 | 1.2292 | 1.4901 | 1.1316 | 1.3762 | 1.5695 | 0.9085 | 2.2466 | 1.9082 | 2.3886 |
| Gorai.004G053100 | -0.3188 | 0.2201 | -0.1549 | 0.2742 | 0.5328 | 0.4800 | 0.3766 | 0.0253 | -0.1135 | 0.0755 |
| Gorai.004G053200 | 0.3617 | 0.8055 | 0.5599 | 0.6284 | 0.4800 | 0.5065 | 0.8062 | 0.6665 | 0.4456 | 0.7627 |
| Gorai.004G053300 | -0.3872 | -0.4202 | 0.2967 | 0.2648 | 0.0682 | 0.0531 | 0.2900 | -0.4089 | -0.7696 | -0.2676 |
| Gorai.004G069100 | 0.0000 | 0.0000 | 0.0000 | 0.0000 | -0.2757 | 0.0000 | 0.0000 | 0.0000 | 0.0000 | 0.0000 |
| Gorai.004G069200 | -0.0862 | -0.6198 | -1.0000 | -0.9586 | -0.2366 | -1.0969 | -0.5086 | -0.9208 | -0.6383 | 0.0000 |
| Gorai.004G069300 | 0.0719 | 0.8420 | 0.1004 | 0.4150 | 0.4150 | 0.3075 | 0.4548 | 0.4166 | 0.6042 | -0.0809 |
| Gorai.004G069400 | -0.0809 | 0.0334 | 0.1206 | -0.2007 | 0.2095 | 0.6304 | 1.8404 | 1.2119 | 0.8513 | 0.1367 |
| Gorai.004G069500 | -0.9208 | 0.0000 | -1.3010 | 0.0000 | -1.0458 | -0.9586 | -0.2840 | 0.0000 | 0.0000 | 0.0000 |
| Gorai.004G078000 | 0.0000 | 0.0000 | 0.0000 | -0.4949 | 0.0000 | 0.0000 | 1.4426 | 1.4373 | 0.0000 | -2.0000 |
| Gorai.004G078100 | 0.4425 | 0.2253 | 0.3075 | 0.3979 | 0.1004 | -0.3768 | -0.5850 | -0.8861 | -0.6198 | 0.0000 |
| Gorai.004G079600 | 0.0000 | 0.0000 | -0.4437 | -0.6198 | -0.8539 | 0.0000 | 0.0000 | 0.0000 | 0.0000 | 0.0000 |
| Gorai.004G079700 | 0.0000 | 0.0000 | -0.4089 | 0.0086 | 0.2405 | -0.6778 | 0.0000 | 0.0000 | 0.0000 | 0.0000 |
| Gorai.004G088100 | 0.5224 | 0.6149 | 0.7559 | 0.6857 | 0.6542 | 0.7093 | 0.8954 | 0.9671 | 1.0022 | 0.8531 |
| Gorai.004G088200 | 0.0000 | 0.1875 | -0.0862 | -0.3665 | -0.3098 | 0.0934 | 0.9983 | 0.8585 | 0.0000 | 0.0000 |
| Gorai.004G088300 | 0.1761 | 0.0569 | -0.6576 | -0.3188 | -1.0000 | -0.7959 | -0.2924 | -0.3468 | -0.0809 | 0.0000 |
| Gorai.004G117300 | 0.2577 | 1.1284 | 0.9410 | 0.7372 | 0.4771 | 0.6532 | 1.2572 | 1.4771 | 0.8675 | 1.0573 |
| Gorai.004G117400 | 0.6848 | 1.2022 | 0.5478 | 0.5966 | 0.4082 | 0.5514 | 1.8173 | 1.5529 | 0.0000 | 0.0000 |
| Gorai.004G117500 | 0.0000 | 0.0000 | 0.0000 | 0.0000 | 0.0000 | -0.1135 | 0.9657 | 1.3952 | 0.0000 | 0.0000 |
| Gorai.004G124700 | 0.0000 | 0.0000 | 0.0569 | -0.1079 | 0.3010 | 0.5416 | 0.7259 | 0.3892 | -0.4949 | 0.4265 |
| Gorai.004G124800 | 0.1399 | 0.0000 | -0.8861 | -0.3665 | -0.5229 | -0.1427 | 0.9859 | 1.0508 | 0.0000 | 0.0000 |
| Gorai.004G126800 | 0.0000 | -0.1487 | 0.0000 | -0.4949 | -0.7447 | -0.7447 | 0.2945 | 0.8982 | -0.6990 | 0.0000 |
| Gorai.004G126900 | 0.0000 | 0.0000 | 0.0000 | 0.0000 | 0.0000 | 0.5038 | 1.2903 | 1.4087 | 0.0000 | 0.0000 |
| Gorai.004G128800 | -0.2757 | 0.1367 | -0.6778 | -0.8539 | 0.0000 | 0.0000 | 0.2553 | 0.1614 | -0.2291 | 0.0000 |
| Gorai.004G128900 | 0.4200 | 0.0000 | -0.4949 | -0.7696 | -0.1024 | 0.3385 | 1.3075 | 1.5968 | 0.0000 | 0.0000 |
| Gorai.004G129000 | 1.0496 | -0.0458 | 0.0000 | 0.0000 | -0.3279 | 0.0000 | 0.6128 | 1.3577 | 0.0000 | 0.0000 |
| Gorai.004G133600 | 0.0000 | 0.0000 | 0.0000 | 0.0000 | 0.0000 | 0.0000 | -0.9586 | 0.0000 | 0.0000 | 0.0000 |
| Gorai.004G133700 | 0.7126 | 0.3636 | 0.8982 | 1.1206 | 1.4661 | 1.0726 | 1.7473 | 1.8013 | 1.6119 | 1.8808 |
| Gorai.004G133900 | 0.6618 | 0.9727 | 0.6355 | 0.3892 | 0.6675 | 1.0224 | 0.9552 | 1.0233 | 1.0322 | 0.8062 |
| Gorai.004G134000 | 0.4249 | 0.4116 | 0.4487 | 0.4281 | 0.5490 | 0.8000 | 1.2969 | 0.8007 | 0.3927 | 0.9633 |
| Gorai.004G134100 | 2.9640 | 2.8950 | 2.5087 | 2.1617 | 2.2232 | 2.2289 | 1.5592 | 2.6170 | 2.0866 | 2.8069 |
| Gorai.004G134200 | 0.0000 | 0.0000 | 0.0000 | -0.4815 | -0.2596 | 0.0000 | 0.1461 | -0.1675 | 0.0000 | 0.0000 |
| Gorai.004G134500 | 0.0000 | 0.0000 | -1.0969 | 0.7033 | 0.0000 | 0.0000 | -0.9586 | 0.0000 | 0.0000 | 0.0000 |
| Gorai.004G134600 | -2.0000 | -0.3565 | -0.4202 | 0.0000 | 0.0000 | -0.3098 | 1.5228 | 1.7660 | 1.9737 | 1.0149 |
| Gorai.004G134700 | 0.8357 | 0.4857 | 0.9469 | 0.8573 | 0.8982 | 0.7076 | 1.6898 | 1.1477 | 0.8382 | 1.0821 |
| Gorai.004G135500 | 0.0000 | 0.3444 | 0.0000 | -1.3010 | -0.7959 | -1.6990 | -1.0458 | 0.0000 | 0.0000 | 0.0000 |
| Gorai.004G135900 | 0.4728 | 1.2531 | 1.2936 | 1.2760 | 1.2833 | 1.4291 | 2.4106 | 2.5349 | 0.6503 | 0.8319 |
| Gorai.004G136000 | 0.0000 | 0.0000 | -0.0362 | 0.0719 | -0.2840 | 0.1818 | 0.9465 | 0.5119 | -0.0605 | 0.3243 |
| Gorai.004G136100 | 0.7694 | 0.7160 | 0.5514 | 0.6243 | 0.8028 | 0.9309 | 0.3424 | 0.6149 | 0.1553 | 0.7910 |
| Gorai.004G137300 | 0.0000 | 0.0000 | -1.6990 | -1.6990 | 0.0000 | -0.9586 | -1.3010 | -1.6990 | -1.5229 | 0.0000 |
| Gorai.004G138100 | -0.9586 | -1.5229 | -1.3010 | -1.0458 | -1.2218 | -1.1549 | -0.6021 | -0.5376 | -0.7212 | -0.4685 |
| Gorai.004G138400 | 2.3465 | 2.1183 | 2.2015 | 2.3658 | 2.1627 | 2.4653 | 2.1816 | 1.8621 | 1.9234 | 1.7087 |
| Gorai.004G138500 | 0.7520 | 0.4742 | 0.0607 | 0.1206 | -0.1024 | 0.1430 | 0.3243 | 0.3909 | 0.6085 | -0.2757 |
| Gorai.004G138600 | 0.2553 | 0.6637 | 0.3927 | 0.9469 | 0.6474 | 1.2188 | -0.8239 | 0.1271 | 0.1271 | 0.0569 |
| Gorai.004G138700 | 0.0000 | 0.0000 | 0.0000 | 0.9212 | 0.0000 | 0.0000 | 0.0000 | 0.0000 | 0.0000 | 0.0000 |
| Gorai.004G138800 | 0.0000 | 0.0000 | 0.0000 | 0.4757 | 0.0000 | 0.0000 | 0.0000 | 0.0000 | 0.0000 | 0.0000 |
| Gorai.004G139700 | -1.1549 | 0.9750 | 0.7536 | 0.9934 | 1.0390 | 0.4378 | 0.6776 | 1.1206 | 0.0000 | 0.7853 |
| Gorai.004G139800 | 1.1059 | 1.2797 | 1.1652 | 0.9850 | 1.0204 | 1.0618 | 0.9795 | 1.3318 | 1.2721 | 1.3228 |
| Gorai.004G152300 | -1.0000 | -0.2757 | -1.3979 | 0.3729 | 0.5843 | 0.9745 | 1.2154 | -0.2757 | 0.0000 | -0.3979 |
| Gorai.004G153900 | 0.5623 | 0.9547 | 0.8751 | 1.2140 | 1.0199 | 2.4628 | 0.3201 | 0.5024 | 0.4624 | 0.4378 |
| Gorai.004G154000 | 0.0000 | 0.0000 | 0.0000 | 0.0000 | 0.0000 | 0.0000 | 0.0000 | -1.3010 | -1.0000 | 0.0000 |
| Gorai.004G168900 | -0.2291 | 0.3838 | 0.2253 | 1.2653 | 1.3239 | 1.4774 | 0.9722 | 0.1875 | 0.1644 | 0.0969 |
| Gorai.004G169000 | -0.1427 | 0.4298 | -0.1192 | 0.5145 | 1.3766 | 0.8089 | -0.9208 | -1.0000 | -0.7447 | 0.0000 |
| Gorai.004G169100 | 1.1069 | 1.4526 | 0.8519 | 0.7396 | 0.9930 | 1.2878 | 2.0753 | 2.0972 | 2.0294 | 2.0950 |
| Gorai.004G172700 | 0.0000 | -1.0458 | -1.0458 | -0.8861 | 0.0000 | 0.0000 | 0.0828 | -1.1549 | -0.8539 | 0.0000 |
| Gorai.004G172800 | 0.4698 | 1.4777 | 0.7810 | 0.8791 | 0.6180 | 0.7760 | 1.1544 | 1.7702 | 1.0000 | 1.9934 |
| Gorai.004G172900 | 0.3892 | 0.0000 | 0.0000 | 0.0000 | 0.0000 | -1.0969 | 1.1741 | 1.0785 | 0.0000 | 0.6365 |
| Gorai.004G173100 | -0.0506 | -0.0969 | 0.5263 | 0.6138 | 1.0626 | 1.9457 | 0.9581 | 1.4670 | 0.3541 | 1.7133 |
| Gorai.004G173200 | 1.8792 | 1.6845 | 1.7651 | 1.6027 | 1.5190 | 1.5592 | 1.4856 | 1.2704 | 1.2586 | 1.2143 |
| Gorai.004G173300 | 0.5428 | 0.7686 | 0.5502 | 0.6628 | 0.6920 | 0.7118 | 0.3979 | 0.1931 | 0.0755 | 0.2279 |
| Gorai.004G184400 | 0.0000 | 0.0000 | -1.5229 | -0.4559 | -0.5686 | -0.6990 | -0.5086 | -0.8239 | 0.0000 | -0.5528 |
| Gorai.004G184500 | 0.8698 | 1.6126 | 1.0611 | 0.9777 | 0.9863 | 0.5922 | 0.7796 | 1.4939 | 1.6234 | 1.2006 |
| Gorai.004G187000 | 0.0000 | 0.0000 | 0.0000 | 0.0000 | 0.0000 | 0.0000 | 0.0000 | 0.0000 | 0.0000 | 0.0000 |
| Gorai.004G187100 | 0.0000 | 0.0000 | -1.5229 | 0.0000 | -1.3010 | 0.0000 | 0.0000 | -1.3979 | -1.0969 | 0.0000 |
| Gorai.004G187300 | 0.0000 | 0.0000 | -0.4202 | 0.4265 | 0.0000 | 0.0000 | -0.0555 | 0.7551 | 0.7513 | 0.6435 |
| Gorai.004G187400 | 0.0000 | 0.0000 | 0.0000 | -1.0969 | -0.8539 | -0.4685 | -0.4815 | -1.3010 | -1.0458 | 0.0000 |
| Gorai.004G192300 | -0.5229 | 0.0000 | 0.0000 | 0.0000 | 0.5302 | 1.2014 | 0.0000 | 0.6149 | 0.0000 | 0.6721 |
| Gorai.004G192400 | 0.0000 | 0.0000 | 0.0000 | 0.0000 | 0.0000 | -0.0506 | -1.5229 | 0.0000 | 0.0000 | 0.0000 |
| Gorai.004G192500 | -0.5528 | 0.0000 | 0.4249 | -0.6576 | 0.0000 | -0.3279 | 1.4796 | 1.2028 | 0.0000 | 1.4673 |
| Gorai.004G192600 | -0.7447 | 0.0000 | -0.2596 | -0.0177 | 0.0000 | 0.0000 | 0.2810 | 0.2672 | -0.5086 | 0.4900 |
| Gorai.004G200500 | 0.0000 | 0.0000 | 0.0000 | 0.0000 | 0.0000 | 0.0000 | 0.1173 | 0.0000 | 0.0000 | 0.0000 |
| Gorai.004G200800 | -0.3468 | 0.0000 | 0.0934 | -0.3188 | 0.0000 | 0.0000 | 0.0000 | 0.1903 | 0.0170 | 0.2625 |
| Gorai.004G200900 | 0.0000 | 0.0000 | -0.3468 | 0.1875 | -1.0969 | -0.1367 | 1.9581 | 0.9943 | 0.5224 | 0.9961 |
| Gorai.004G201000 | 0.0000 | -0.2596 | -0.5686 | -0.2840 | -0.7447 | 0.0000 | -0.7959 | -0.9208 | -0.6576 | 0.0000 |
| Gorai.004G201100 | -0.7447 | 0.0000 | -0.4949 | -0.3665 | -0.0410 | -0.2596 | -0.0655 | -1.2218 | -0.9208 | 0.0000 |
| Gorai.004G208600 | 1.1287 | 1.1455 | 1.1099 | 0.9315 | 0.9759 | 0.7896 | 0.9165 | 1.0842 | 1.0554 | 0.0000 |
| Gorai.004G208700 | -0.1871 | 0.3404 | -0.0362 | -0.6198 | 0.0899 | 0.3655 | 0.7657 | 0.8998 | 0.6096 | 0.9614 |
| Gorai.004G208800 | 0.0000 | 0.7634 | 0.9978 | 1.0004 | 1.2106 | 1.9828 | 0.9934 | 1.2945 | 1.1113 | 1.3705 |
| Gorai.004G208900 | 0.0492 | -0.0506 | 0.0492 | -0.1938 | 0.1461 | 0.3304 | 0.0645 | -0.1805 | 0.0000 | 0.0000 |
| Gorai.004G209000 | -1.3010 | -0.0706 | 0.0000 | 0.0000 | -0.8239 | 0.0000 | 1.0386 | 0.5328 | 0.1903 | 0.3522 |
| Gorai.004G209100 | 0.0000 | 0.0000 | -1.1549 | 0.0000 | 0.0000 | -0.9208 | 0.1903 | -0.3768 | 0.0000 | -0.1079 |
| Gorai.004G209200 | -0.6778 | 0.1139 | 0.7709 | 0.3139 | 0.2718 | 0.7126 | 1.7974 | 1.8284 | 0.0000 | 0.4669 |
| Gorai.004G209300 | 0.1335 | 0.0000 | -0.1192 | -1.3010 | 0.0000 | -0.4437 | 0.0000 | -0.4685 | 0.0000 | -0.2076 |
| Gorai.004G210200 | 0.0682 | 1.0748 | 0.4983 | 0.0334 | -0.0915 | -0.3565 | 0.7634 | 0.7796 | 0.4579 | 0.4393 |
| Gorai.004G210300 | 0.0000 | 0.0000 | 0.0000 | 0.8837 | 0.0000 | 0.0000 | 0.0000 | 0.0000 | 0.0000 | 0.0000 |
| Gorai.004G210400 | 0.0000 | 0.0000 | 0.0000 | 0.0000 | 0.0000 | 0.0000 | 0.0000 | 0.0000 | 0.0000 | 0.0000 |
| Gorai.004G214800 | 0.2788 | 0.1732 | 0.1614 | -0.0655 | -0.0605 | 0.1614 | -0.3372 | 1.0086 | 1.0997 | 0.8021 |
| Gorai.004G214900 | 0.4265 | 0.6444 | 0.3598 | 0.5011 | 0.5328 | 0.5775 | 0.3424 | 0.1523 | -0.0132 | 0.2148 |
| Gorai.004G215000 | 0.8014 | 0.4518 | 1.1411 | 0.7348 | 0.8299 | 0.4624 | 0.4216 | 0.8351 | 0.4728 | 0.9499 |
| Gorai.004G215100 | -0.7959 | -0.3468 | 0.1461 | 0.4298 | 0.1335 | 0.2504 | 0.5877 | 0.4425 | 0.1790 | 0.3892 |
| Gorai.004G215200 | -0.5229 | 0.0000 | -0.8861 | 0.0000 | 0.0000 | 0.0000 | -0.1739 | -1.0458 | -0.7696 | 0.0000 |
| Gorai.004G219300 | 0.4456 | 0.7007 | 0.9440 | 1.1517 | 0.9566 | 0.8312 | 1.0686 | 1.5991 | 1.5990 | 1.5296 |
| Gorai.004G219400 | 0.4393 | 0.6821 | 0.6284 | 0.9903 | 1.0881 | 1.0145 | 1.1278 | 0.8651 | 0.6767 | 0.9445 |
| Gorai.004G220400 | -0.3098 | 0.1673 | -0.5850 | -0.2366 | -0.5850 | -0.1308 | 0.1523 | -0.9208 | -0.6576 | 0.0000 |
| Gorai.004G220500 | -0.9208 | 0.0000 | -0.7447 | 0.0000 | -1.3010 | 0.0000 | -0.3979 | -0.2218 | -0.5229 | -0.4089 |
| Gorai.004G220600 | 0.0000 | 0.0000 | -1.2218 | -0.4815 | 0.0294 | -0.4089 | -1.3010 | -0.0915 | -0.7959 | 0.1239 |
| Gorai.004G220700 | 0.0899 | 0.0000 | -0.0915 | 0.0000 | -1.0969 | 0.0170 | 0.1584 | 0.1303 | -0.4318 | 0.3263 |
| Gorai.004G220800 | 0.0000 | -0.9208 | -1.0458 | -1.3979 | -1.3010 | -0.0088 | 0.8954 | 0.0253 | 0.2253 | -0.5376 |
| Gorai.004G220900 | 0.7152 | 0.2765 | 0.5899 | 0.4886 | -0.0088 | 0.0969 | 1.2574 | 1.1514 | 0.0000 | 0.0000 |
| Gorai.004G221000 | 0.7024 | 0.6928 | 0.8915 | 0.9675 | 0.9609 | 1.1511 | 0.9309 | 0.6021 | 0.7451 | 0.2672 |
| Gorai.004G223200 | 0.0000 | 0.0000 | 0.0000 | -1.5229 | -1.5229 | -1.5229 | 0.0000 | -1.1549 | -0.8861 | 0.0000 |
| Gorai.004G223300 | -0.7447 | -0.7212 | -0.0269 | -0.9208 | -0.3188 | -0.1805 | 0.7259 | 1.2340 | 0.5866 | 1.0298 |
| Gorai.004G223400 | 1.6069 | 1.7776 | 1.5945 | 1.5668 | 1.5974 | 1.5196 | 1.8110 | 1.8707 | 1.6218 | 1.9789 |
| Gorai.004G226600 | 0.9518 | 0.7701 | 0.8062 | 0.7679 | 0.9232 | 0.6821 | 1.1679 | 1.1389 | 0.8014 | 1.2723 |
| Gorai.004G226700 | 0.0000 | 0.0000 | -1.6990 | 0.0000 | -0.8539 | 0.0000 | 0.3945 | 0.1875 | 0.0000 | 0.0000 |
| Gorai.004G226800 | 1.4622 | 1.4084 | 1.6946 | 1.7904 | 1.5095 | 1.1917 | 1.4783 | 1.4570 | 1.5292 | 1.2256 |
| Gorai.004G226900 | 0.1987 | 0.1399 | 0.5024 | 0.5276 | 0.6830 | 0.5263 | -0.2007 | -1.3979 | -1.1549 | 0.0000 |
| Gorai.004G227000 | 0.0000 | 0.0000 | -0.6990 | 0.0374 | 0.5250 | -0.0969 | 0.0607 | 0.0492 | -0.4318 | 0.0000 |
| Gorai.004G227100 | 0.8488 | 0.8235 | 0.8910 | 0.8704 | 1.1335 | 0.9410 | 2.1230 | 1.4791 | 0.9542 | 0.3838 |
| Gorai.004G229200 | 0.0000 | 0.3945 | -0.0506 | -0.3768 | 0.3096 | 0.0294 | 0.2765 | 0.4669 | 0.0000 | 0.2014 |
| Gorai.004G229300 | 1.4512 | 1.0864 | 1.4302 | 1.3657 | 1.3158 | 1.1858 | 1.4148 | 1.6344 | 1.6843 | 1.4987 |
| Gorai.004G229400 | 0.2625 | 0.2430 | 0.6702 | 0.8055 | 0.4969 | 0.2095 | 0.1038 | 0.1303 | 0.0000 | 0.0000 |
| Gorai.004G229500 | 0.0000 | 0.0000 | 0.3962 | 0.0000 | 0.1959 | 0.0000 | 1.4763 | 1.1323 | 0.0000 | 0.0000 |
| Gorai.004G229800 | 0.7152 | 1.0269 | 0.9063 | 0.9009 | 1.1258 | 0.9624 | 1.0835 | 1.2074 | 0.4031 | 0.6053 |
| Gorai.004G232400 | -0.3010 | -0.7447 | -0.3468 | -0.6990 | -0.0655 | 0.7235 | -0.2518 | 1.4414 | 1.5987 | 1.0652 |
| Gorai.004G232500 | 2.3526 | 2.1396 | 1.8876 | 1.9145 | 1.7389 | 1.7398 | 1.5607 | 1.9212 | 1.8081 | 1.9540 |
| Gorai.004G232600 | -0.0410 | 0.1703 | 0.2279 | 0.4378 | 0.0414 | -0.1308 | 0.9509 | 0.6138 | 0.6405 | 0.5105 |
| Gorai.004G232700 | 1.5485 | 1.9114 | 1.5108 | 1.7236 | 1.8302 | 1.5283 | 0.5105 | 2.0328 | 1.2156 | 2.2605 |
| Gorai.004G232800 | 0.0000 | 0.0000 | -1.6990 | 0.0000 | 0.0000 | 0.0000 | -1.5229 | 0.3365 | 0.2788 | 0.3263 |
| Gorai.004G241400 | 0.0000 | 0.0000 | 0.0000 | 0.0000 | -1.5229 | -1.3979 | 0.0000 | 0.0000 | 0.0000 | 0.0000 |
| Gorai.004G241500 | 0.0000 | 0.2788 | 0.0792 | 1.4168 | 3.2973 | 3.5499 | 0.0000 | 0.0000 | 0.0000 | 0.0000 |
| Gorai.004G241600 | 0.6998 | 0.8028 | 0.7825 | 0.8506 | 0.7505 | 1.1752 | 0.8585 | 1.0641 | 0.9868 | 0.7284 |
| Gorai.004G253100 | 1.0261 | 1.0917 | 0.9652 | 0.9263 | 1.3902 | 1.3475 | 1.6533 | 1.2148 | 1.2555 | 1.0927 |
| Gorai.004G253200 | 0.0569 | -0.1135 | 0.0000 | -0.6778 | 0.0000 | -0.8539 | 2.0347 | 1.3220 | 0.0000 | 0.0000 |
| Gorai.004G253300 | 0.0000 | 0.0000 | 0.0000 | 0.0000 | -1.2218 | 0.0000 | 0.0000 | 0.0000 | 0.0000 | 0.0000 |
| Gorai.004G253400 | 0.0000 | -0.6778 | -1.3010 | -0.2007 | 1.2639 | 0.7694 | 0.7348 | -0.2076 | -0.5686 | -0.0605 |
| Gorai.004G258900 | 0.3655 | 0.6875 | 0.8627 | 0.9263 | 0.8241 | 0.5977 | 0.7931 | 0.9238 | 1.0022 | 0.7419 |
| Gorai.004G259000 | 1.5015 | 1.4481 | 1.4398 | 1.4121 | 1.5148 | 1.4498 | 1.1903 | 1.4728 | 1.1732 | 1.6011 |
| Gorai.004G259100 | 0.2380 | -0.8861 | -0.0410 | -1.0458 | 0.0000 | 0.0000 | 0.0000 | -0.6778 | -0.4089 | 0.0000 |
| Gorai.004G259200 | 1.3499 | 0.8000 | 1.3890 | 1.2702 | 1.0781 | 0.9375 | 1.3381 | 1.5038 | 1.5494 | 1.3465 |
| Gorai.004G259300 | 0.1644 | 0.7243 | 0.9694 | 0.9499 | 0.8633 | 0.7860 | 0.6812 | 1.2109 | 1.0660 | 1.0245 |
| Gorai.004G262100 | 1.3004 | 1.5395 | 1.0671 | 0.8525 | 1.1099 | 0.9365 | 1.3216 | 1.3913 | 1.2958 | 1.4113 |
| Gorai.004G262200 | -0.1079 | 0.1072 | -0.4685 | -0.0410 | -0.6198 | -0.4685 | 0.8774 | 1.0370 | 0.0000 | 0.0000 |
| Gorai.004G262300 | 0.5539 | 0.7160 | 0.8041 | 0.7513 | 0.7435 | 0.8482 | 0.6839 | 0.7952 | 0.6263 | 0.8645 |
| Gorai.004G262400 | 0.3962 | -1.6990 | 1.4138 | 0.6096 | 0.5705 | -0.5086 | 1.0000 | 0.9809 | 0.6284 | 0.9850 |
| Gorai.004G262500 | 0.0000 | -2.0000 | 0.0000 | 0.7435 | -0.2291 | 0.0000 | 1.9593 | 1.9151 | 0.0000 | 0.0000 |
| Gorai.004G264200 | 0.1335 | 0.4564 | 0.4857 | 0.6222 | 0.6749 | 0.7589 | 0.3075 | 0.6454 | 0.5623 | 0.6561 |
| Gorai.004G264300 | 0.0000 | 0.0000 | -1.5229 | 0.0000 | 0.0000 | 0.0000 | -0.9208 | -1.0969 | -1.5229 | -0.9586 |
| Gorai.004G264400 | 0.0000 | 0.7210 | 0.6702 | 0.5302 | 0.6964 | 0.8228 | 1.0496 | 0.7723 | 0.3560 | 0.0000 |
| Gorai.004G265400 | 0.5289 | 0.5717 | 0.7973 | 0.7259 | 0.7716 | 0.7839 | 0.9330 | 0.5328 | 0.5798 | 0.1614 |
| Gorai.004G265500 | 0.0000 | 0.0000 | -0.1192 | -0.0458 | -0.6990 | 0.0000 | 1.0561 | 1.3066 | 0.0000 | 0.0453 |
| Gorai.004G265600 | 0.0000 | 0.0000 | 0.0000 | -1.5229 | 1.0962 | 1.2248 | 0.0000 | 0.0000 | 0.0000 | 0.0000 |
| Gorai.004G265700 | 0.1987 | 0.0000 | -0.5528 | -1.0969 | -0.1249 | -0.7696 | 0.0000 | -0.5086 | -0.6778 | -0.4437 |
| Gorai.004G265800 | 0.0000 | 0.0000 | -1.3010 | -1.5229 | -1.1549 | 0.0000 | 0.0000 | 0.0000 | 0.0000 | 0.0000 |
| Gorai.004G265900 | 0.0000 | -0.4202 | 0.0000 | -0.7212 | -1.0458 | -0.1135 | 0.3096 | 0.4133 | 0.0000 | 0.0000 |
| Gorai.004G266000 | 1.5215 | 1.5231 | 1.6961 | 1.5932 | 1.7169 | 1.7604 | 1.6061 | 1.6390 | 1.6145 | 1.5960 |
| Gorai.004G268300 | 0.0000 | 0.0000 | 0.0000 | 0.0000 | 0.0000 | 0.0000 | -1.6990 | 0.0000 | 0.0000 | 0.0000 |
| Gorai.004G268400 | 0.0000 | 0.0000 | -1.0458 | -1.2218 | -0.2596 | -1.1549 | -0.9208 | -1.0458 | -0.7696 | 0.0000 |
| Gorai.004G268500 | 0.0000 | -0.6198 | 0.0000 | 0.3502 | 0.6911 | 0.5353 | 0.0000 | -1.3010 | -1.0000 | 0.0000 |
| Gorai.004G268600 | 1.4350 | 1.6031 | 1.3572 | 1.2130 | 1.0204 | 0.8344 | 1.4135 | 1.3334 | 1.1945 | 1.3835 |
| Gorai.004G269300 | 0.4378 | 0.5763 | 0.7093 | 0.7110 | 0.6561 | 0.8965 | 0.4082 | 0.4900 | 0.4857 | 0.4265 |
| Gorai.004G269400 | -0.3372 | 0.4757 | -0.4437 | 0.6405 | 0.6884 | 0.4346 | 0.7505 | 0.4800 | -0.2518 | 0.6998 |
| Gorai.004G269500 | 1.3414 | 1.6188 | 1.4589 | 1.0453 | 0.6464 | 0.6128 | 0.0755 | 1.8049 | 1.0828 | 2.0230 |
| Gorai.005G001200 | 0.0000 | 0.0000 | 0.0000 | -0.8539 | 0.0000 | -0.5086 | 0.0000 | 0.0000 | 0.0000 | 0.0000 |
| Gorai.005G001300 | 0.0000 | 0.0000 | 0.0000 | -0.9208 | -1.5229 | -0.1612 | -1.6990 | 0.0000 | 0.0000 | 0.0000 |
| Gorai.005G001400 | 0.0000 | 0.0000 | 0.0000 | 0.0000 | 0.0000 | 0.0000 | 0.0000 | -1.3010 | -1.0969 | 0.0000 |
| Gorai.005G001500 | 1.4513 | 1.2709 | 1.4726 | 1.4363 | 1.6433 | 1.4203 | 1.6087 | 1.8515 | 1.6370 | 1.9194 |
| Gorai.005G001600 | -2.0000 | 0.0000 | -0.2147 | 0.2227 | -2.0000 | 0.0000 | 1.6948 | 0.2041 | 0.0000 | 0.3502 |
| Gorai.005G001700 | 0.8169 | 0.9494 | 0.7882 | 0.8182 | 0.8344 | 1.1119 | 1.1858 | 1.4751 | 1.1461 | 1.5542 |
| Gorai.005G001800 | 0.0000 | 0.3054 | -1.6990 | -0.3468 | -0.3872 | -0.6778 | -0.4949 | -0.5229 | 0.0000 | 0.0000 |
| Gorai.005G001900 | 0.0000 | 0.0000 | 0.0000 | -0.6198 | 0.0000 | -1.0969 | 0.0000 | -1.5229 | -1.2218 | 0.0000 |
| Gorai.005G002000 | 1.1886 | 1.1099 | 1.0656 | 1.0457 | 1.0111 | 1.2953 | 0.9217 | 1.2641 | 0.9926 | 1.3817 |
| Gorai.005G002100 | 0.0000 | -0.2676 | -1.6990 | 0.9335 | 1.1290 | 0.7160 | 1.2206 | 0.7839 | 0.8762 | 0.5752 |
| Gorai.005G002200 | 0.4886 | 0.5079 | 0.4502 | 0.7810 | 0.5539 | 0.7084 | 0.6253 | 0.8344 | 0.9542 | 0.5658 |
| Gorai.005G002300 | 1.3464 | 1.2826 | 1.4053 | 1.3804 | 1.5696 | 1.4967 | 1.2824 | 1.1284 | 1.1638 | 1.0141 |
| Gorai.005G002400 | 0.7152 | 0.6075 | 0.3784 | -0.0706 | 0.1461 | 0.0374 | 0.8142 | 1.3336 | 1.0208 | 1.3448 |
| Gorai.005G002500 | 0.9961 | 1.1219 | 1.2900 | 1.2847 | 1.3353 | 1.1364 | 0.9974 | 1.3952 | 1.3627 | 1.3606 |
| Gorai.005G002600 | 0.0000 | -2.0000 | 0.2330 | -0.0410 | 0.3118 | 0.5172 | 0.7589 | 1.2365 | 0.0000 | 1.0077 |
| Gorai.005G002700 | 0.0000 | -0.7959 | -0.7212 | -1.6990 | -1.2218 | -1.3010 | 1.3553 | 0.5263 | -0.3565 | 0.3181 |
| Gorai.005G002800 | 0.1523 | 0.8998 | 0.7513 | 0.7380 | 0.8149 | 0.4579 | 0.1072 | 0.9370 | 0.8014 | 0.9845 |
| Gorai.005G002900 | 0.7846 | 1.1281 | 1.0934 | 1.1351 | 0.9542 | 0.7435 | 0.5966 | 1.3414 | 1.1000 | 1.3406 |
| Gorai.005G003000 | 0.0000 | 0.0000 | -0.3665 | 0.0000 | 0.0000 | 0.0000 | 0.7789 | 0.9571 | 0.0000 | 0.1703 |
| Gorai.005G003100 | 0.8382 | 0.5539 | 0.8675 | 0.2945 | 0.2648 | 0.8692 | 0.2742 | 0.5877 | -0.1549 | 0.4200 |
| Gorai.005G003200 | 0.0000 | 1.6274 | 1.8184 | 2.2628 | 2.1420 | 2.4201 | 2.5972 | 2.9047 | 1.5046 | 2.5441 |
| Gorai.005G003300 | 2.4474 | 1.9908 | 1.7043 | 1.4987 | 1.3122 | 1.8835 | 2.2101 | 2.6954 | 2.1082 | 2.7517 |
| Gorai.005G003400 | -0.7212 | 0.0000 | -1.0000 | -0.4089 | -0.7212 | 0.4440 | 1.1523 | -0.1612 | -0.4685 | -0.0315 |
| Gorai.005G010900 | 0.6599 | 0.6776 | 0.8915 | 0.7284 | 0.7396 | 0.9800 | 0.3304 | 0.7126 | 0.5378 | 0.7846 |
| Gorai.005G011000 | 0.4150 | 0.4082 | 0.3598 | 0.4014 | 0.4330 | 0.5866 | 0.7007 | 0.6675 | 0.4166 | 0.0000 |
| Gorai.005G011100 | 1.1096 | 1.0137 | 1.0162 | 0.6646 | 0.5224 | 0.8831 | 1.5046 | 1.1007 | -0.1135 | -0.2676 |
| Gorai.005G011200 | 0.1987 | -0.1805 | -0.2076 | 0.2480 | -0.1192 | 0.0569 | 0.8597 | 1.2025 | 1.2485 | 1.0722 |
| Gorai.005G011300 | 0.0000 | 0.0000 | 0.0000 | 0.0000 | 0.0000 | 0.0000 | -0.2366 | 0.0000 | 0.0000 | 0.0000 |
| Gorai.005G011400 | 0.0000 | 0.0000 | -0.7959 | -1.5229 | -0.9208 | -1.6990 | -0.3372 | 0.4048 | -0.0362 | 0.5763 |
| Gorai.005G016300 | -0.1549 | 0.7177 | 0.6721 | 0.7566 | 0.8585 | 0.7497 | 0.7218 | 0.9159 | 0.9309 | 0.7300 |
| Gorai.005G016400 | 1.3122 | 1.4342 | 1.3572 | 1.3412 | 1.3508 | 1.5184 | 1.2860 | 1.2949 | 1.0469 | 1.4026 |
| Gorai.005G016500 | -1.0969 | -0.6990 | 0.0000 | -1.6990 | 0.0000 | 0.0000 | -1.6990 | 0.0000 | 0.0000 | 0.0000 |
| Gorai.005G016600 | 1.1370 | 1.1945 | 1.1511 | 1.1229 | 1.0763 | 1.4168 | 1.0633 | 1.2333 | 1.0350 | 1.3174 |
| Gorai.005G016700 | 0.0000 | 0.0000 | -0.5229 | 0.0719 | 0.0000 | 0.0000 | 0.7604 | 0.7042 | 0.0000 | 0.7597 |
| Gorai.005G016800 | 1.0170 | 1.1414 | 0.7657 | 0.9269 | 0.5966 | 0.7716 | 1.7699 | 1.5505 | 1.5550 | 1.2548 |
| Gorai.005G016900 | 0.0000 | -0.0555 | 0.0000 | 0.0000 | 0.0000 | 0.0000 | 0.0000 | 0.0000 | 0.0000 | 0.0000 |
| Gorai.005G021100 | 0.2330 | 0.4742 | 0.1303 | -0.1367 | -0.0655 | 0.2380 | 0.4346 | 0.5289 | 0.1703 | 0.6767 |
| Gorai.005G021200 | 1.0212 | 1.2408 | 1.0366 | 1.0561 | 0.9763 | 1.0722 | 1.1396 | 1.3381 | 1.2898 | 1.3187 |
| Gorai.005G021300 | 0.6064 | 0.4857 | 0.6884 | 1.0426 | 0.6405 | 0.2625 | 0.5132 | 1.4723 | 1.6162 | 1.1389 |
| Gorai.005G021400 | 0.7839 | -0.0410 | 1.6458 | 1.0906 | -1.2218 | -0.9586 | 0.0000 | 0.6693 | 0.6117 | 0.6580 |
| Gorai.005G025000 | 0.0000 | 0.0000 | 0.0000 | 0.0000 | 0.0000 | 0.0000 | 1.7560 | 2.2651 | 0.0000 | 0.0000 |
| Gorai.005G025100 | 0.2330 | 0.0000 | 0.3655 | 0.8960 | -0.2076 | -0.3372 | 0.7642 | 1.1816 | 1.3564 | 0.0000 |
| Gorai.005G025200 | 0.0000 | 0.0000 | 0.0000 | 0.0000 | 0.0000 | 0.0000 | 0.0000 | 0.0000 | 0.0000 | 0.0000 |
| Gorai.005G025300 | 0.0000 | 0.8837 | -1.0000 | -0.5229 | 0.1818 | -0.6576 | 0.0212 | -0.6383 | -1.0000 | -0.4815 |
| Gorai.005G025400 | -0.1079 | 0.1367 | 0.5211 | 0.1303 | 0.4609 | 0.4624 | 0.9217 | 1.0519 | 0.9538 | 1.0737 |
| Gorai.005G047100 | -1.1549 | -1.2218 | -0.7447 | -1.5229 | 0.0000 | -1.3979 | -0.8239 | -0.5686 | 0.0000 | -0.3098 |
| Gorai.005G047200 | -0.2840 | -0.1192 | 0.0934 | 0.5988 | -0.3279 | -0.0757 | 0.5729 | 0.8149 | 0.8971 | 0.6243 |
| Gorai.005G050100 | 0.5378 | 0.6758 | 1.0969 | 1.1798 | 1.3471 | 1.4542 | 1.3833 | 1.3318 | 0.9877 | 1.4219 |
| Gorai.005G050200 | 0.0000 | 0.0000 | 0.0000 | 0.0000 | 1.5043 | 1.4682 | 2.4612 | 2.4340 | 0.0000 | 0.0000 |
| Gorai.005G050300 | 1.1691 | 1.3840 | 1.3677 | 1.2704 | 1.3450 | 1.6641 | 1.4771 | 1.6562 | 1.4273 | 1.7553 |
| Gorai.005G050400 | 0.0000 | 0.0000 | 0.0000 | 0.0000 | -0.9208 | -1.0458 | 0.0000 | -0.8861 | -0.6198 | 0.0000 |
| Gorai.005G050500 | 1.3915 | 1.4604 | 1.4739 | 1.1807 | 1.3280 | 1.1937 | 1.4445 | 1.1867 | 1.2918 | 0.9513 |
| Gorai.005G053300 | 1.2380 | 1.6448 | 1.5827 | 1.6671 | 1.3820 | 1.8672 | 1.9979 | 1.6377 | 1.4440 | 1.5748 |
| Gorai.005G053400 | 0.0000 | 0.0000 | 0.0000 | 0.0000 | 0.0000 | 0.0000 | 0.8195 | 1.5959 | 0.0000 | 0.0000 |
| Gorai.005G053500 | 0.0000 | 0.0000 | 0.0000 | 0.0000 | 0.0000 | 0.0000 | 0.0000 | 0.0000 | 0.0000 | 0.0000 |
| Gorai.005G053600 | 0.7427 | 0.7796 | 0.8657 | 0.8633 | 0.8915 | 0.9009 | 0.8287 | 0.7716 | 0.7875 | 0.6821 |
| Gorai.005G053700 | 0.9186 | 0.1732 | 1.0726 | 0.6160 | 0.7589 | 0.7701 | 1.0187 | 0.7210 | 0.7243 | 0.5599 |
| Gorai.005G056900 | 0.7889 | 0.9410 | 0.9020 | 1.0314 | 1.0170 | 1.0334 | 1.2269 | 1.5219 | 1.5549 | 1.4108 |
| Gorai.005G057000 | -0.0269 | 0.1367 | 0.1761 | 0.6345 | -0.1024 | -0.3468 | 0.3655 | 0.6776 | 0.7752 | 0.4579 |
| Gorai.005G057100 | 0.6785 | 1.0326 | 1.0469 | 1.0362 | 0.6955 | 0.2856 | 1.4850 | 1.2558 | 1.4004 | 0.9201 |
| Gorai.005G057200 | -0.0862 | 0.9360 | -0.1549 | 0.5933 | 0.7218 | 0.5105 | 0.9258 | 0.2095 | -0.1938 | 0.3711 |
| Gorai.005G057300 | 0.0000 | -1.0000 | 0.0000 | -0.1549 | 0.0000 | 0.0000 | 0.7582 | 0.6138 | 0.0000 | 0.0000 |
| Gorai.005G065700 | 0.4472 | 0.8075 | 0.4216 | 0.9786 | 0.6590 | 0.5763 | -0.8239 | 0.0414 | 0.0682 | -0.0655 |
| Gorai.005G065800 | 1.7425 | 1.6572 | 1.4026 | 1.6144 | 1.9494 | 2.2110 | 1.0734 | 1.3593 | 1.0580 | 1.4883 |
| Gorai.005G065900 | 0.1367 | 0.6646 | 0.0000 | 0.0000 | 0.0000 | 0.0000 | 0.8162 | 0.2989 | 0.0253 | 0.4183 |
| Gorai.005G066000 | 2.6867 | 2.1564 | 2.4838 | 2.5778 | 2.2547 | 2.6919 | 2.7711 | 2.3928 | 2.5323 | 1.9874 |
| Gorai.005G066100 | -0.0809 | 0.2175 | 0.4969 | 0.5763 | 0.7716 | 0.9745 | 0.8476 | 0.8669 | 0.3636 | 0.3096 |
| Gorai.005G066900 | 0.0000 | 0.0000 | 0.0000 | -0.5850 | -0.9586 | 0.0000 | 0.0000 | -1.3010 | -1.0000 | 0.0000 |
| Gorai.005G067000 | 0.0000 | 0.0000 | -0.8239 | -1.0458 | 0.0000 | 0.0000 | 0.0000 | -0.8539 | -0.5850 | 0.0000 |
| Gorai.005G067100 | 0.0000 | 0.0000 | -2.0000 | 0.0000 | 0.0000 | 0.0000 | 0.0000 | -1.6990 | -1.3979 | 0.0000 |
| Gorai.005G097800 | 3.1180 | 2.6671 | 3.1159 | 2.1329 | 1.4232 | 1.1471 | 2.0207 | 2.1668 | 1.9047 | 2.2806 |
| Gorai.005G101700 | 0.5289 | 0.8338 | 0.7396 | 0.8267 | 0.5729 | 0.9196 | 1.0090 | 1.3103 | 1.1232 | 1.3764 |
| Gorai.005G106800 | -0.1938 | -0.1192 | -0.7447 | 0.1903 | 0.1903 | 0.4216 | -0.4202 | 0.0294 | 0.0000 | 0.2330 |
| Gorai.005G106900 | 0.6946 | 0.4456 | 0.6972 | 0.6345 | 0.6599 | 0.8585 | 0.9777 | 0.6493 | 0.6405 | 0.5888 |
| Gorai.005G107000 | 0.8797 | 1.0370 | 1.2079 | 1.4666 | 1.6748 | 1.2625 | 1.1310 | 1.5694 | 1.5676 | 1.5017 |
| Gorai.005G109800 | 0.0000 | -0.2366 | -0.0088 | 0.0000 | 0.1430 | 0.1430 | 0.8609 | 1.0481 | 0.0000 | 0.0000 |
| Gorai.005G109900 | 1.6162 | 1.6243 | 1.6858 | 1.5984 | 1.5885 | 1.4881 | 1.3874 | 1.5903 | 1.5768 | 1.5357 |
| Gorai.005G110000 | 1.0853 | 1.0577 | 1.4156 | 1.3286 | 1.2087 | 2.0095 | 1.9977 | 1.3056 | 1.1386 | 1.3729 |
| Gorai.005G110100 | 0.0000 | 0.0000 | -0.6990 | -0.2596 | 0.1461 | 2.6677 | -0.6778 | -0.4202 | 0.0000 | -0.1549 |
| Gorai.005G112100 | 0.9320 | -0.7447 | 0.8312 | 0.9484 | 0.7067 | 0.4518 | 0.6444 | 1.1449 | 0.9304 | 1.2370 |
| Gorai.005G114400 | 0.0043 | 0.0000 | -0.0862 | 0.3483 | -0.1938 | 0.0828 | -0.4089 | -0.0757 | -0.5850 | 0.0043 |
| Gorai.005G114500 | 0.0000 | 0.0000 | -0.1871 | 1.9414 | 0.7168 | 0.7966 | 2.6669 | 2.4317 | 0.3579 | 0.0000 |
| Gorai.005G144600 | 0.0000 | 0.3243 | -0.1308 | 0.4116 | -0.0223 | 0.5119 | 0.6405 | 1.0554 | 0.8089 | 1.0920 |
| Gorai.005G144700 | 1.1575 | 1.0603 | 1.0986 | 0.8938 | 0.9685 | 0.6425 | 1.1813 | 0.9090 | 0.9253 | 0.8195 |
| Gorai.005G150700 | 0.0000 | 0.2480 | 0.0000 | -0.6778 | 0.7767 | 0.6920 | 1.3827 | 1.7453 | 0.0000 | 0.9370 |
| Gorai.005G150800 | 0.8299 | -0.3565 | 0.2253 | 0.6928 | 0.5065 | 0.5065 | 1.9731 | 0.5911 | -0.1367 | 0.6522 |
| Gorai.005G150900 | 0.0000 | 0.0000 | 0.0000 | -0.2218 | 0.0000 | -0.6576 | 0.0000 | 0.0000 | 0.0000 | 0.0000 |
| Gorai.005G163000 | -0.0757 | -0.6198 | -1.3979 | 0.0000 | -0.3979 | 0.0374 | -0.5086 | 1.3265 | 1.5912 | -0.3098 |
| Gorai.005G174300 | -0.7696 | 0.0828 | -1.5229 | -0.4949 | 0.0294 | -0.1675 | 0.4786 | -0.2924 | -0.0223 | 0.0000 |
| Gorai.005G185500 | 0.0128 | 0.1139 | -0.1871 | 0.4564 | -0.1805 | 0.0000 | 0.1584 | 0.7284 | 0.6075 | 0.7672 |
| Gorai.005G185600 | -0.0706 | 0.0000 | -0.4437 | 0.2788 | -0.2366 | -0.5850 | 0.8915 | 0.6042 | 0.4048 | 0.6893 |
| Gorai.005G190500 | 0.0607 | 0.5933 | 0.6304 | 0.6866 | 0.4150 | 0.4814 | 1.5227 | 1.8217 | 0.0000 | 0.0000 |
| Gorai.005G190600 | 0.0000 | 1.0682 | 1.1159 | 1.0043 | 0.9903 | 0.7825 | 1.2507 | 1.6297 | 1.5326 | 1.5567 |
| Gorai.005G193500 | 0.9504 | 0.6385 | 1.1641 | 1.3479 | 1.4609 | 1.0366 | 1.0374 | 1.3537 | 1.4589 | 1.1139 |
| Gorai.005G193600 | -0.1079 | -0.3010 | -0.0655 | 0.2380 | 0.8910 | 0.4533 | -0.5376 | 0.3560 | 0.2553 | 0.3802 |
| Gorai.005G193700 | 0.2529 | 0.9154 | 0.6170 | 0.5478 | 0.7701 | 0.5490 | -0.4949 | 0.3711 | 0.6201 | -0.6778 |
| Gorai.005G193800 | 0.4857 | 0.3284 | 0.4955 | 0.3856 | 0.3856 | 0.1875 | 0.5211 | 0.7657 | 0.8609 | 0.5514 |
| Gorai.005G214400 | 0.4409 | 0.8692 | 0.7152 | 0.7642 | 0.7042 | 0.6075 | 0.5763 | 0.6812 | 0.5263 | 0.7404 |
| Gorai.005G214500 | -0.1871 | 0.0969 | -1.0000 | 0.0755 | 0.6405 | 0.6335 | 0.0000 | -1.3010 | -1.0458 | 0.0000 |
| Gorai.005G214600 | 0.4065 | 0.1818 | 0.6405 | 0.5999 | -0.4437 | 0.3856 | 1.7244 | 1.2903 | 1.0099 | 1.1781 |
| Gorai.005G214700 | 1.1421 | 1.3120 | 1.3530 | 1.4496 | 1.4640 | 1.4817 | 1.7859 | 1.4891 | 1.4706 | 1.3530 |
| Gorai.005G214800 | 0.9624 | 1.5729 | 1.3736 | 1.5642 | 1.6382 | 1.6631 | 2.0778 | 1.8724 | 1.3795 | 2.0148 |
| Gorai.005G214900 | 1.9361 | 1.6403 | 2.0725 | 1.8531 | 1.8915 | 1.9763 | 1.8557 | 1.8913 | 1.8230 | 1.7570 |
| Gorai.005G215000 | -0.4949 | 0.0000 | -0.4437 | -0.3565 | 0.0000 | -0.5376 | 1.0959 | 0.5843 | 0.0000 | 0.0000 |
| Gorai.005G216200 | 0.7110 | 0.9647 | 1.0504 | 1.1126 | 1.2931 | 1.1767 | 1.0374 | 0.8645 | 0.8854 | 0.7694 |
| Gorai.005G216300 | 0.7218 | 0.8129 | 0.9269 | 1.0043 | 1.0993 | 1.1550 | 0.8500 | 0.8274 | 0.8062 | 0.7818 |
| Gorai.005G216400 | 1.6431 | 2.0756 | 1.6080 | 1.2636 | 1.4526 | 1.2428 | 1.7616 | 1.1229 | 1.3446 | 0.4099 |
| Gorai.005G216500 | 1.6335 | 1.7885 | 1.5926 | 1.3948 | 1.2790 | 1.3187 | 1.1004 | 1.8747 | 1.8717 | 1.7956 |
| Gorai.005G217300 | 0.0000 | 0.0000 | 0.0000 | 0.0000 | 0.0899 | -0.2007 | 0.6170 | 0.6628 | 0.0899 | 0.0000 |
| Gorai.005G217400 | -0.4089 | -0.2076 | -0.3372 | -0.0506 | -0.0757 | 1.1316 | -0.0506 | -1.0969 | -0.8539 | 0.0000 |
| Gorai.005G217500 | 0.0000 | 0.0000 | 0.0000 | 0.0000 | 0.0000 | 0.0000 | 0.3365 | 0.0000 | 0.0000 | 0.0000 |
| Gorai.005G217600 | -0.5376 | -1.2218 | 0.3201 | -0.2076 | -0.7212 | -0.7212 | -0.6990 | -1.6990 | -1.3979 | 0.0000 |
| Gorai.005G217700 | 0.6646 | 0.3160 | 0.9736 | 0.8136 | 0.0170 | 0.2068 | 0.8439 | 0.7388 | 0.8325 | 0.5263 |
| Gorai.005G226700 | -2.0000 | -1.1549 | 1.1297 | 1.0199 | 1.0473 | 0.5658 | 1.3617 | 1.2605 | 1.3555 | 0.9557 |
| Gorai.005G226800 | 1.7270 | 2.0891 | 1.8886 | 1.9557 | 1.9077 | 1.7282 | 1.2603 | 2.2122 | 1.5740 | 2.4178 |
| Gorai.005G226900 | 0.0000 | -0.3010 | -0.5686 | -0.1427 | 0.1523 | -0.3279 | 0.9090 | 0.4594 | 0.0000 | -0.1938 |
| Gorai.005G232300 | -0.8239 | -0.9586 | -0.2441 | -0.8239 | -0.6383 | 1.5436 | 1.1133 | 1.4574 | 1.5475 | 1.2526 |
| Gorai.005G232400 | 0.0000 | 0.4393 | -0.8239 | -0.2840 | 0.0253 | -0.4202 | -0.4949 | -0.3872 | 0.0000 | -0.7212 |
| Gorai.005G232500 | 1.3444 | 1.5453 | 1.4310 | 1.7276 | 1.5630 | 1.2858 | 0.3892 | 0.7007 | 0.5119 | 0.7803 |
| Gorai.005G232600 | 0.0000 | 0.4871 | -0.4318 | 0.0000 | -0.2218 | -0.6021 | 0.6474 | 0.8116 | -0.0655 | 0.0000 |
| Gorai.005G233700 | 0.2718 | 1.0004 | -0.1079 | 0.8893 | 0.9212 | 0.6128 | 0.7938 | 0.7752 | 0.7267 | 0.7466 |
| Gorai.005G233800 | -0.4559 | -0.5229 | -0.2757 | 0.8274 | 2.3328 | 3.3579 | 1.3189 | 0.0253 | -0.6021 | 0.2304 |
| Gorai.005G233900 | 1.8227 | 1.7417 | 1.6098 | 1.6498 | 1.6740 | 1.4603 | 1.3103 | 1.4681 | -0.5528 | 0.0000 |
| Gorai.005G234000 | 0.0000 | 0.0000 | 0.0000 | 0.0000 | 0.0000 | 0.0000 | 0.0000 | 0.0000 | 0.0000 | 0.0000 |
| Gorai.005G234100 | 0.3802 | 0.8537 | 0.8041 | 0.9562 | 0.8241 | 1.3617 | 1.1278 | 1.1679 | 1.0257 | 1.1433 |
| Gorai.005G234200 | 0.8802 | 0.9191 | 0.8591 | 1.1446 | 0.1614 | 0.5717 | 0.4564 | 2.0835 | 2.3405 | 0.8235 |
| Gorai.005G237800 | 0.0000 | 0.0000 | 0.0000 | 0.0000 | 0.0000 | 0.0000 | 0.1703 | 0.0000 | 0.0000 | 0.0000 |
| Gorai.005G237900 | 0.0000 | 0.0000 | 0.0000 | 0.0000 | 0.0000 | 0.0000 | 0.0000 | 0.0000 | 0.0000 | 0.0000 |
| Gorai.005G238000 | -0.2676 | 0.0000 | -0.7959 | 0.0000 | 0.0000 | 0.0000 | -0.6990 | 1.2840 | 1.5473 | 0.0000 |
| Gorai.005G238100 | -0.0915 | 0.1072 | 0.0334 | -0.7447 | 0.2095 | 0.1903 | -0.5528 | -1.6990 | -1.5229 | 0.0000 |
| Gorai.005G238200 | 0.9542 | 0.6803 | 0.8069 | 0.6911 | 1.1004 | 1.0641 | 0.9805 | 0.7050 | 0.6928 | 0.6493 |
| Gorai.005G238300 | 0.0000 | 0.0000 | 0.0000 | 0.0000 | 0.0000 | 0.0000 | 0.0000 | 0.0000 | 0.0000 | 0.0000 |
| Gorai.005G238400 | 0.0000 | 0.0000 | 0.0000 | -1.0458 | 0.0000 | -1.2218 | 0.0000 | 0.0000 | 0.0000 | 0.0000 |
| Gorai.005G238500 | 0.0000 | 0.0000 | 0.0000 | -1.1549 | 0.0000 | 0.0000 | 0.3464 | 0.8439 | 0.0000 | 0.0000 |
| Gorai.005G239100 | -0.3565 | -0.1427 | -0.3979 | 0.2405 | -0.7447 | 1.1278 | 1.1942 | 0.3598 | 0.2330 | 0.4014 |
| Gorai.005G239200 | 0.6693 | 1.0561 | 0.7860 | 1.0860 | 1.2676 | 1.4703 | 1.4115 | 0.9854 | 0.9015 | 0.9956 |
| Gorai.005G239300 | 0.0000 | 0.4843 | 0.5877 | 0.4014 | 0.0934 | -0.0088 | 1.0099 | -0.1427 | 0.0000 | 0.0000 |
| Gorai.005G239400 | 1.2159 | 1.6467 | 1.1953 | 1.1153 | 1.1048 | 1.1367 | 1.3619 | 1.6399 | 1.6279 | 1.5839 |
| Gorai.005G239500 | -0.4089 | -0.9586 | -0.7447 | 0.0000 | -1.3010 | -0.6778 | -0.5528 | -1.0000 | -0.7447 | 0.0000 |
| Gorai.005G239600 | 0.8597 | 1.6270 | 1.5245 | 1.9172 | 1.7480 | 1.5762 | 0.6064 | -0.0757 | 0.0000 | 0.1903 |
| Gorai.005G240900 | 0.9415 | 1.0386 | 1.0245 | 1.1206 | 1.1461 | 1.0322 | 1.1508 | 1.3553 | 0.9917 | 1.3744 |
| Gorai.005G241000 | 0.4249 | -0.1308 | 0.2430 | 0.3010 | 0.1644 | -0.2840 | 0.4099 | 1.4005 | 1.6706 | 0.0000 |
| Gorai.005G241100 | -0.0362 | -0.0862 | 0.0000 | 0.2330 | 0.3222 | 0.2648 | -0.2441 | 0.0128 | 0.2833 | 0.0000 |
| Gorai.005G241200 | 0.3075 | -0.0088 | 0.6474 | 0.8028 | 0.7709 | 1.2256 | 0.9657 | 1.4289 | 1.4476 | 1.3047 |
| Gorai.005G241300 | 0.0000 | 0.0000 | 0.0000 | 0.0000 | 0.0000 | 0.0000 | 0.0000 | 0.0000 | 0.0000 | 0.0000 |
| Gorai.005G241400 | 0.9557 | 1.1976 | 0.8633 | 1.2327 | 1.2135 | 1.1547 | 1.4562 | 1.2438 | 1.2579 | 1.1569 |
| Gorai.005G241500 | 0.9595 | 1.1632 | 0.8751 | 0.9133 | 0.8814 | 1.0641 | 1.5949 | 1.5784 | 0.9385 | 1.0249 |
| Gorai.005G241600 | 0.0000 | 0.0000 | -0.8539 | 0.0000 | 0.0000 | 0.0000 | 0.9974 | 1.4631 | 0.1584 | 0.5832 |
| Gorai.005G242500 | 0.3222 | -0.1612 | 0.6739 | 0.5977 | 0.5635 | 0.3655 | 1.0993 | 0.2148 | 0.1335 | 0.2227 |
| Gorai.005G242600 | 0.2765 | 0.4698 | 0.6821 | 0.6493 | 0.6928 | 0.8215 | 0.6749 | 0.7202 | 0.6405 | 0.6503 |
| Gorai.005G242700 | -0.6198 | 0.1173 | -0.7447 | -1.2218 | 0.0899 | 0.0086 | -0.7959 | 0.0000 | 0.0000 | 0.0000 |
| Gorai.005G242800 | 1.9676 | 1.8972 | 1.9363 | 1.7077 | 1.6961 | 1.5676 | 1.6013 | 1.4428 | 1.2887 | 1.4987 |
| Gorai.005G242900 | 1.2028 | 1.4849 | 1.2964 | 1.2087 | 1.0806 | 0.7917 | 0.9243 | 0.8976 | 1.0030 | 0.6618 |
| Gorai.005G243000 | 1.3918 | 1.3391 | 1.4732 | 1.5138 | 1.3714 | 1.3856 | 1.3397 | 1.3595 | 1.1587 | 1.4451 |
| Gorai.005G245300 | 0.0000 | 0.0000 | 0.0000 | -1.3979 | 0.0000 | 0.0000 | 0.0000 | 0.0000 | 0.0000 | 0.0000 |
| Gorai.005G245400 | 0.0000 | 0.0000 | 0.0000 | 0.0000 | 0.0000 | 0.0000 | 1.0806 | 1.2127 | 0.0000 | 0.0000 |
| Gorai.005G245500 | 0.0000 | 0.0000 | 0.0000 | 0.0000 | 0.0000 | 0.0000 | -1.0000 | 0.0000 | 0.0000 | 0.0000 |
| Gorai.005G245600 | 0.0000 | 0.0000 | -0.9586 | 0.0000 | -0.8861 | 0.0000 | 1.4094 | 1.4354 | 0.0000 | 0.0000 |
| Gorai.005G245700 | 1.7710 | 1.8742 | 1.5315 | 1.6357 | 1.5852 | 1.1103 | 0.8235 | 0.2788 | 0.5502 | 0.0000 |
| Gorai.005G245800 | -0.5850 | -0.0655 | -0.3279 | 0.8639 | 0.7832 | 0.2672 | -0.5376 | 0.1072 | 0.0000 | 0.3729 |
| Gorai.005G245900 | 0.0000 | 0.0000 | -1.5229 | -0.4318 | 1.4867 | 2.6649 | 0.0000 | 0.0000 | 0.0000 | 0.0000 |
| Gorai.005G246000 | 0.9435 | 0.8089 | 1.0386 | 0.9360 | 0.8075 | 0.7559 | 0.5403 | 0.6902 | 0.5682 | 0.7292 |
| Gorai.005G246100 | 0.0453 | 0.0000 | -0.3279 | -0.3565 | -1.3010 | -1.3010 | -0.1249 | -1.3010 | -1.0000 | 0.0000 |
| Gorai.005G246200 | -0.9586 | 0.0000 | -1.5229 | 0.0000 | -1.6990 | 0.0000 | -1.5229 | 0.0000 | 0.0000 | 0.0000 |
| Gorai.005G246300 | 0.0000 | 0.2279 | -0.2596 | -0.7447 | 0.0755 | 0.1644 | 0.8887 | 1.1096 | 0.6684 | 1.0326 |
| Gorai.005G246400 | 0.0000 | 0.4298 | -0.7212 | -0.2676 | -0.2757 | -0.6990 | 0.7664 | 0.3118 | 0.3075 | 0.2455 |
| Gorai.005G246500 | -0.5229 | -0.3768 | -0.1079 | -0.1427 | -0.0969 | -0.4685 | -0.3010 | -0.6198 | -0.6990 | -0.6198 |
| Gorai.005G246600 | 1.1599 | 0.3747 | 0.8639 | 0.1239 | 0.1072 | -0.1938 | -0.1487 | 0.2648 | 0.3444 | 0.0792 |
| Gorai.005G246700 | 1.1183 | 1.0641 | 1.2030 | 0.9763 | 1.1075 | 1.0948 | 0.9440 | 1.5747 | 1.1738 | 1.7353 |
| Gorai.005G246800 | 0.0000 | 0.0000 | 0.0000 | 0.0000 | 0.0000 | -1.3010 | -1.6990 | 0.0000 | 0.0000 | 0.0000 |
| Gorai.005G246900 | 1.0500 | 2.2661 | 1.5366 | 1.7530 | 1.8609 | 1.3149 | 1.5880 | 0.6693 | 0.4713 | 0.7536 |
| Gorai.005G260300 | 0.9827 | 0.6365 | 0.9212 | 0.9542 | 0.8943 | 0.8169 | 0.8797 | 0.8189 | 0.7177 | 0.8426 |
| Gorai.005G260400 | 0.3502 | 0.1732 | 0.1335 | 0.0374 | 0.4425 | -0.0132 | -0.0706 | 0.4150 | 0.5092 | 0.1987 |
| Gorai.005G260500 | 0.3560 | 0.6064 | 0.4609 | 0.6365 | 0.9201 | 0.7774 | 0.4133 | 0.7818 | -0.2676 | 0.8014 |
| Gorai.005G260600 | -0.7959 | 0.0000 | -0.3279 | -0.4202 | 0.0000 | 0.0000 | -0.2441 | -0.3768 | -0.7447 | -0.2291 |
| Gorai.005G260700 | -1.5229 | 0.0000 | 0.0000 | -0.2757 | -1.6990 | 0.0000 | 1.1978 | 0.8645 | 0.0000 | 0.0000 |
| Gorai.005G260800 | 0.0000 | 0.0000 | 0.0000 | -0.7696 | 0.0000 | 0.0000 | -1.5229 | 0.0000 | 0.0000 | 0.0000 |
| Gorai.005G260900 | 0.3945 | 1.1287 | 0.8482 | 0.7832 | 0.4843 | 0.8338 | 0.8739 | 0.9360 | 0.7604 | 0.5132 |
| Gorai.006G001800 | 0.0000 | 0.0000 | -0.6198 | 0.4757 | 2.2090 | 2.9023 | 0.0000 | 0.0000 | 0.0000 | 0.0000 |
| Gorai.006G001900 | -0.8539 | -0.3565 | -1.0458 | -0.1549 | -1.5229 | -1.0969 | -1.3979 | 0.0000 | 0.0000 | 0.0000 |
| Gorai.006G002000 | 0.3747 | 0.5647 | 0.8089 | 0.8000 | 0.8482 | 0.9773 | 0.7672 | 0.6345 | 0.5877 | 0.6149 |
| Gorai.006G002100 | 0.0000 | 0.0000 | 0.0000 | 0.0000 | 0.0000 | 0.0000 | 0.0000 | 0.0000 | 0.0000 | 0.0000 |
| Gorai.006G004700 | 1.1380 | 0.5065 | 0.2355 | 0.4654 | -0.2291 | -0.2076 | 2.2682 | 0.9832 | 0.9991 | 0.8943 |
| Gorai.006G004800 | 1.6060 | 1.5626 | 1.6282 | 1.5306 | 1.5687 | 1.8751 | 1.5093 | 1.5383 | 1.2541 | 1.6610 |
| Gorai.006G004900 | 0.0000 | -0.1249 | 0.3263 | 0.1271 | -0.2007 | -0.4089 | 0.5465 | -0.7696 | -0.8539 | 0.0000 |
| Gorai.006G005900 | 0.0000 | 0.0000 | 0.0000 | 0.0000 | 0.0000 | 0.0000 | 0.0000 | 0.0000 | 0.0000 | 0.0000 |
| Gorai.006G009400 | -0.0269 | 0.0000 | 0.1335 | 0.3201 | -0.2924 | 0.5977 | 1.5160 | 1.3094 | 0.3054 | 1.5504 |
| Gorai.006G009500 | 0.9020 | 0.8169 | 1.0166 | 0.8921 | 0.8156 | 0.9227 | 1.2925 | 1.2947 | 1.2927 | 1.1706 |
| Gorai.006G009600 | 1.0484 | 0.7267 | 1.1355 | 0.9465 | 1.0073 | 1.0592 | 1.1358 | 0.9165 | 0.5877 | 0.5051 |
| Gorai.006G009700 | 0.8182 | 0.7404 | 0.9881 | 1.1031 | 1.1816 | 1.1867 | 1.2460 | 1.4323 | 1.3833 | 1.4135 |
| Gorai.006G018200 | 0.0000 | 0.0000 | -2.0000 | -1.6990 | -1.6990 | 0.0000 | -2.0000 | -1.5229 | -1.3010 | 0.0000 |
| Gorai.006G018600 | 0.0000 | 0.0000 | 0.0000 | 0.0000 | 0.0000 | 0.0000 | -0.1427 | 0.0000 | 0.0000 | 0.0000 |
| Gorai.006G018700 | 0.0000 | 0.0000 | 0.0000 | 0.0000 | 0.0000 | 0.0000 | 0.0000 | -0.6576 | -0.9586 | -0.5086 |
| Gorai.006G018800 | 0.0000 | 0.0000 | 0.0000 | 0.0000 | 0.0000 | 0.0000 | -0.4318 | 0.0000 | 0.0000 | 0.0000 |
| Gorai.006G019400 | 0.0000 | 0.0000 | 0.0000 | 0.0000 | 0.0000 | 0.0000 | 0.0000 | 0.0000 | 0.0000 | 0.0000 |
| Gorai.006G020700 | 2.1037 | 2.0813 | 2.1500 | 2.2510 | 2.1290 | 2.4346 | 2.0211 | 1.6485 | 1.6861 | 1.5307 |
| Gorai.006G020800 | -0.9586 | 0.0000 | -1.3010 | -1.6990 | -1.6990 | -0.7696 | 0.1004 | -0.5850 | -1.0458 | -0.4089 |
| Gorai.006G044200 | 0.0000 | 0.0719 | -0.7212 | 0.0000 | 0.0000 | -0.5376 | 0.4997 | 0.7275 | 0.4362 | 0.5366 |
| Gorai.006G050500 | 0.0000 | 0.0000 | 0.0000 | 0.0000 | 0.0000 | 0.0000 | 0.0000 | 0.0000 | 0.0000 | 0.0000 |
| Gorai.006G050600 | 0.0000 | 0.0000 | 0.0000 | -0.6990 | 0.0000 | 0.0000 | 0.0000 | 0.0000 | 0.0000 | 0.0000 |
| Gorai.006G051100 | 1.1523 | 0.8241 | 1.2512 | 1.0504 | 1.1694 | 0.9499 | 1.4064 | 1.2984 | 1.3967 | 1.0745 |
| Gorai.006G053000 | 1.2526 | 1.3092 | 1.4597 | 1.4982 | 1.3191 | 1.3888 | 1.1706 | 1.1562 | 1.1099 | 0.9385 |
| Gorai.006G056600 | 0.4857 | -1.6990 | 0.4502 | 0.6010 | 0.3385 | 0.3424 | 0.8513 | 0.4133 | 0.4757 | 0.2455 |
| Gorai.006G063500 | 0.4472 | 0.0000 | 0.4942 | 0.7135 | 0.2380 | 0.7589 | 0.8202 | 0.7185 | 0.6821 | 0.6580 |
| Gorai.006G075300 | -0.3665 | 0.0000 | 0.2923 | 0.5611 | 0.3598 | 0.0645 | 0.5977 | 0.4330 | 0.2718 | -1.6990 |
| Gorai.006G075400 | 0.1614 | -0.6383 | 0.3304 | 0.4654 | 0.3424 | 0.1004 | 0.8597 | 0.7664 | -0.4202 | 1.0162 |
| Gorai.006G075500 | 0.7101 | 0.2480 | 0.8987 | 0.6721 | 0.6128 | 0.6937 | 1.1626 | 0.8325 | 0.4698 | 0.1430 |
| Gorai.006G075600 | 0.0828 | 0.6117 | -0.1938 | -0.0132 | 0.1584 | 0.5623 | -0.3372 | 0.2923 | 0.0000 | 0.3979 |
| Gorai.006G079800 | 0.0682 | -0.6778 | 0.3010 | -0.6576 | -0.6576 | 1.1498 | -0.1871 | 0.9474 | 0.0000 | 1.2122 |
| Gorai.006G080200 | 0.0000 | 0.0000 | 0.0000 | 0.0000 | 0.0000 | 0.0000 | 0.0000 | 0.0000 | 0.0000 | 0.0000 |
| Gorai.006G080300 | 0.0000 | 0.0000 | 0.0000 | 0.0000 | 0.0000 | 0.0000 | 0.0000 | 0.0000 | 0.0000 | 0.0000 |
| Gorai.006G080400 | 0.0000 | 0.0000 | 0.0000 | -1.5229 | -0.1192 | -0.2757 | 0.0000 | 0.0000 | 0.0000 | 0.0000 |
| Gorai.006G080700 | -0.9208 | 0.5289 | -0.0269 | 0.2900 | 1.0422 | 1.0810 | 1.3339 | 1.0199 | -0.1427 | 1.1827 |
| Gorai.006G080800 | 0.0000 | 0.0000 | -0.8861 | -0.7959 | 0.3284 | 0.2878 | -0.0458 | -0.6021 | -0.7447 | -0.5528 |
| Gorai.006G082000 | -0.6990 | -0.1871 | -0.6576 | -0.3279 | -0.4089 | 0.0000 | 0.7774 | 1.2709 | 0.5855 | 0.9638 |
| Gorai.006G082400 | 0.5966 | 1.2079 | 0.7340 | 0.6972 | 1.0039 | 0.9154 | 1.3979 | 0.9138 | 0.8241 | 0.9294 |
| Gorai.006G082500 | 1.5396 | 1.2281 | 1.4847 | 1.4125 | 1.0386 | 1.0931 | 1.4691 | 1.6216 | 1.6473 | 1.5201 |
| Gorai.006G085700 | 0.0000 | 0.0000 | -0.7959 | -0.7212 | 0.0000 | 0.0000 | 0.0492 | -1.0969 | -0.7959 | 0.0000 |
| Gorai.006G085800 | 0.0000 | 0.0000 | 0.0000 | 0.0000 | 0.0000 | 0.0000 | 0.0000 | 0.0000 | 0.0000 | 0.0000 |
| Gorai.006G085900 | 0.0000 | 0.0000 | 0.0000 | 0.0000 | 0.0000 | 0.0000 | 0.0000 | 0.0000 | 0.0000 | 0.0000 |
| Gorai.006G086000 | -1.2218 | 0.7284 | -1.0969 | 0.3284 | 0.2601 | 0.0453 | 0.0899 | -0.7959 | 0.0000 | 0.0000 |
| Gorai.006G088000 | 0.8451 | 1.3522 | 0.9345 | 1.0980 | 1.1847 | 1.6719 | 0.7723 | 1.7115 | 1.9133 | 1.1386 |
| Gorai.006G088100 | 0.0000 | 1.2307 | 0.7024 | 0.7412 | 1.0763 | 1.0030 | 1.6938 | 1.4512 | 1.2420 | 1.2801 |
| Gorai.006G088200 | 0.9165 | 1.1967 | 0.9699 | 1.2036 | 1.3092 | 0.8943 | 1.3981 | 0.7767 | 0.7931 | 0.6866 |
| Gorai.006G089900 | 0.6212 | 0.6702 | 0.3766 | 0.2742 | 0.1004 | 0.4886 | 0.6107 | 1.1691 | 1.2629 | 0.9566 |
| Gorai.006G090000 | 0.1038 | -0.3979 | 0.1271 | -0.0605 | -0.0757 | -0.3372 | 0.7931 | 0.6454 | 0.7404 | 0.4314 |
| Gorai.006G090100 | 0.0000 | 0.0000 | 0.0000 | 0.0000 | 0.0000 | 0.0000 | 0.0000 | 0.0000 | 0.0000 | 0.0000 |
| Gorai.006G090200 | 0.0000 | 0.0000 | -1.0000 | -1.3010 | -1.2218 | 0.0000 | 0.0000 | 0.0000 | 0.0000 | 0.0000 |
| Gorai.006G090300 | -0.6198 | -0.1249 | -0.0969 | 0.0934 | 0.0828 | 0.0755 | -1.5229 | -0.2924 | -0.1249 | -0.6990 |
| Gorai.006G092300 | 0.0000 | 0.0000 | -0.4089 | -0.4437 | 0.0000 | -0.0269 | 0.7185 | 0.3747 | 0.0000 | 0.0000 |
| Gorai.006G092400 | 0.0000 | -0.5086 | -0.3372 | -0.3565 | -0.3279 | -0.3010 | -0.7212 | 0.2279 | 0.3838 | -0.1427 |
| Gorai.006G095500 | 0.0000 | -1.3979 | 0.0000 | -1.3010 | -1.6990 | -2.0000 | 0.0000 | -1.3979 | -1.1549 | 0.0000 |
| Gorai.006G095600 | -0.0555 | 0.1903 | 0.0864 | 0.7143 | -0.2757 | -0.6198 | -0.4202 | 0.2380 | -0.1367 | 0.3909 |
| Gorai.006G095700 | 0.0000 | 0.0000 | 0.0000 | -1.3010 | 0.0000 | 0.0000 | -1.1549 | 0.0000 | 0.0000 | 0.0000 |
| Gorai.006G103100 | -1.0969 | -0.6021 | 0.1239 | 0.3345 | -0.2840 | -0.6778 | 0.1732 | -0.5528 | -0.9586 | -0.5086 |
| Gorai.006G103200 | 0.0000 | 0.5391 | 0.4116 | 0.5185 | 0.7760 | 0.8414 | 1.2028 | 0.9474 | 0.4200 | 0.7634 |
| Gorai.006G103300 | 0.0000 | -0.3768 | -0.3010 | 0.0000 | -0.8239 | 0.0000 | 0.0000 | -0.8539 | -0.5850 | 0.0000 |
| Gorai.006G109900 | 0.6561 | 0.3892 | 0.0374 | 0.8382 | 0.6920 | 0.1492 | 1.1242 | 0.9149 | 0.4609 | 1.0888 |
| Gorai.006G111500 | 0.7050 | 0.1847 | 0.3054 | -0.4202 | 0.0934 | -0.4815 | -0.3188 | 0.0719 | -0.8239 | 0.3054 |
| Gorai.006G111600 | -1.0458 | 0.8927 | -0.7959 | 1.5263 | 2.2954 | 1.8522 | -2.0000 | -1.6990 | -1.3979 | 0.0000 |
| Gorai.006G111700 | -2.0000 | -0.0315 | -0.0506 | -0.4559 | -0.3768 | -0.2924 | 0.4698 | 0.6425 | -0.3188 | 0.0864 |
| Gorai.006G111800 | 0.0000 | 0.0000 | 0.0000 | -0.0555 | -0.4815 | -0.3665 | -0.1805 | -0.5528 | -0.4685 | 0.0000 |
| Gorai.006G117900 | 0.0000 | 0.0000 | 0.0000 | -0.9586 | 0.0000 | 0.0000 | 0.5587 | 0.8686 | 0.0000 | 0.0000 |
| Gorai.006G119400 | 1.2302 | 1.1242 | 1.2627 | 1.0592 | 1.0641 | 1.1501 | 1.4451 | 1.2538 | 0.7490 | 0.8306 |
| Gorai.006G119500 | -0.8539 | 0.0000 | -0.3565 | -0.4949 | -0.1938 | -0.2007 | 0.5798 | -0.7696 | -0.4949 | 0.0000 |
| Gorai.006G120400 | 0.0000 | 0.0000 | -0.6021 | -0.7959 | -0.4089 | 0.0000 | 0.9400 | 0.3711 | 0.4330 | -0.0269 |
| Gorai.006G120500 | -0.0223 | 0.0000 | 0.2330 | 0.2330 | 0.0755 | 0.7076 | 1.5413 | 1.8332 | 0.0000 | 0.8089 |
| Gorai.006G122400 | 0.5145 | 0.5441 | 0.5955 | 0.5428 | 0.5416 | 0.9335 | 0.7619 | 0.6571 | 0.6580 | 0.5866 |
| Gorai.006G122500 | -0.8239 | 0.0607 | -0.2676 | -1.1549 | -0.7696 | -0.2007 | -0.5850 | -0.8539 | 0.0000 | 0.0000 |
| Gorai.006G122600 | 2.3115 | 2.3116 | 2.4195 | 2.4170 | 2.6449 | 2.9049 | 2.0252 | 2.4425 | 2.3736 | 2.3722 |
| Gorai.006G122700 | 0.0000 | 0.0000 | 0.0000 | 0.0000 | 0.0000 | 0.0000 | 0.9474 | 1.3365 | 0.0000 | 0.0000 |
| Gorai.006G122800 | 0.0864 | 0.4362 | 0.3692 | -0.0132 | -1.0969 | 0.5866 | 0.2967 | 0.9004 | -0.0458 | 1.1383 |
| Gorai.006G127700 | 0.0000 | 0.0000 | 0.0000 | 0.0000 | 0.0000 | 0.0000 | 0.0000 | 0.0000 | 0.0000 | 0.0000 |
| Gorai.006G127800 | 0.0000 | 0.0000 | 0.0000 | 0.0000 | 0.0000 | 0.0000 | 0.0000 | -0.4437 | 0.0000 | -0.1805 |
| Gorai.006G127900 | 0.3054 | -0.3665 | -0.0555 | 0.2833 | -0.5528 | -0.3979 | -1.0000 | 0.6551 | 0.3139 | -0.0362 |
| Gorai.006G128700 | 0.5145 | 0.8519 | 0.5353 | 0.5132 | 0.5763 | 0.8414 | 0.6232 | 0.9122 | 0.5821 | 1.0090 |
| Gorai.006G128800 | 0.0000 | 0.0000 | 0.0000 | 0.0000 | 0.0000 | 0.0000 | 0.7084 | 1.1565 | 0.0000 | 0.0000 |
| Gorai.006G128900 | 0.0000 | -0.1805 | 0.0000 | -0.1549 | -0.7212 | 0.0000 | 0.0000 | 0.0000 | 0.0000 | 0.0000 |
| Gorai.006G131600 | 0.2041 | -0.6021 | 0.4031 | 0.6785 | -0.2291 | -1.1549 | 0.4200 | 0.7694 | 0.0000 | 0.3424 |
| Gorai.006G131700 | 0.0000 | 0.0000 | 0.2253 | 0.0000 | -1.1549 | 0.0000 | 0.9736 | 1.3339 | 0.0000 | 0.0000 |
| Gorai.006G131800 | 0.8082 | -0.5229 | -0.2366 | -0.4437 | -0.1487 | 0.0000 | 0.7218 | 0.8432 | 0.5353 | 0.4548 |
| Gorai.006G131900 | 1.3316 | 1.9890 | 1.3004 | 1.1159 | 0.9841 | 0.6955 | -0.0506 | 0.1139 | 0.0000 | 0.1461 |
| Gorai.006G132000 | 0.0000 | 0.0000 | 0.0000 | 0.0000 | 0.0000 | 0.0000 | 0.0000 | 0.0000 | 0.0000 | 0.0000 |
| Gorai.006G132100 | 0.0000 | 0.0000 | 0.0000 | 0.0000 | 0.0000 | 0.0000 | 0.0000 | 0.0000 | 0.0000 | 0.0000 |
| Gorai.006G132200 | -0.5086 | -0.2518 | 0.3139 | 0.1790 | 0.0755 | 0.0645 | 1.3634 | 1.1332 | 0.3962 | 0.1903 |
| Gorai.006G149200 | -1.0458 | -0.0809 | -0.2518 | -0.3279 | -0.4202 | -1.1549 | -1.6990 | -0.6778 | -0.7447 | -0.6778 |
| Gorai.006G149300 | 2.0952 | 1.9898 | 1.8566 | 1.8849 | 2.0443 | 2.2697 | 1.7336 | 1.5581 | 1.2292 | 1.6970 |
| Gorai.006G164900 | 0.0000 | 0.0000 | 0.0000 | 0.4116 | -0.1079 | 0.0000 | 1.1433 | -0.0655 | 0.2068 | 0.0000 |
| Gorai.006G165000 | 0.0000 | 0.0000 | 0.0000 | 0.0000 | 0.0000 | -1.1549 | -1.1549 | 0.0000 | 0.0000 | 0.0000 |
| Gorai.006G165100 | -0.0706 | 0.1072 | -0.1024 | -0.0223 | 0.1206 | 0.0645 | -0.1249 | 0.6628 | 0.4082 | 0.7731 |
| Gorai.006G165200 | 0.0000 | -0.5376 | -0.1938 | -0.6576 | 0.2405 | 0.6561 | 1.4615 | 0.4502 | -0.2840 | 0.1072 |
| Gorai.006G165300 | 0.2304 | 1.4002 | 0.4955 | 1.4909 | 1.4847 | 1.7223 | -1.0969 | 0.3820 | 0.5977 | -0.2840 |
| Gorai.006G165400 | 0.0000 | 0.0000 | 0.0000 | 0.0000 | 0.0000 | 0.0000 | 0.5705 | 0.1072 | 0.0000 | 0.0000 |
| Gorai.006G165500 | 0.0000 | 0.0000 | 0.0000 | 0.0000 | 0.0000 | -1.3979 | 1.2911 | 1.1937 | 0.0492 | 0.0253 |
| Gorai.006G173300 | 0.0000 | 0.0000 | 0.0000 | 0.0000 | 0.0000 | -1.5229 | -1.3010 | -2.0000 | -1.5229 | 0.0000 |
| Gorai.006G173400 | -0.5850 | 0.0000 | -0.2924 | 0.0864 | -0.9208 | -0.7447 | 0.2480 | 0.4116 | 0.3160 | 0.3365 |
| Gorai.006G173500 | 1.0465 | 0.8704 | 0.9666 | 1.0831 | 1.0592 | 0.5670 | 0.1790 | 0.8561 | 1.0314 | 0.4133 |
| Gorai.006G173600 | 0.0000 | 0.0000 | 0.0000 | 0.0000 | 0.0000 | 0.0000 | 0.0000 | 0.0000 | 0.0000 | 0.0000 |
| Gorai.006G173700 | -0.9586 | -0.2924 | -0.1612 | 0.0453 | 0.0682 | 0.0934 | 0.0128 | -0.1367 | 0.0755 | -0.7447 |
| Gorai.006G173800 | 0.3263 | 0.1959 | 0.0645 | 0.3201 | 0.5302 | 0.4533 | 0.5527 | 0.6911 | 0.8871 | 0.1523 |
| Gorai.006G173900 | -0.3665 | 0.6702 | 0.0000 | 0.0000 | -0.8539 | 0.0000 | -0.4437 | 0.0000 | 0.0000 | 0.0000 |
| Gorai.006G174000 | 1.4986 | 1.6383 | 1.5373 | 1.3600 | 0.6721 | 0.5798 | 1.3113 | 1.2066 | 1.4486 | 0.2695 |
| Gorai.006G174100 | 0.0000 | -0.9586 | -1.3979 | 0.5775 | 0.0000 | 0.0000 | -1.3010 | -1.3979 | -1.0969 | 0.0000 |
| Gorai.006G174200 | 1.4499 | 1.3176 | 1.1758 | 1.2393 | 1.2338 | 1.4997 | 0.7490 | 0.9827 | 0.8401 | 1.0354 |
| Gorai.006G186600 | 0.0000 | 0.6656 | -1.6990 | 0.2253 | -0.9208 | -0.9586 | 0.0000 | 0.0000 | 0.0000 | 0.0000 |
| Gorai.006G186700 | -1.3979 | -1.2218 | -1.6990 | -1.3979 | -2.0000 | 0.0000 | 0.0000 | -1.2218 | -0.9208 | 0.0000 |
| Gorai.006G186800 | 0.7332 | 0.6794 | 0.9047 | 0.8156 | 0.7135 | 0.9128 | 0.6866 | 0.9206 | 0.6232 | 0.9818 |
| Gorai.006G186900 | 0.6532 | 0.5575 | 0.5465 | 0.8976 | 0.6981 | 1.3456 | 1.3555 | 0.9903 | 0.4393 | 1.0175 |
| Gorai.006G187000 | 1.0107 | 0.4298 | 1.1374 | 0.8062 | 0.9547 | 0.0253 | 0.7202 | 0.7152 | 0.6232 | 0.6513 |
| Gorai.006G194600 | -0.5528 | -0.3768 | -0.1739 | 0.0899 | 0.3979 | -0.2366 | 0.0294 | 0.1106 | -0.0269 | 0.1614 |
| Gorai.006G194700 | 1.3610 | 1.2997 | 0.6998 | 1.3598 | 0.5250 | 0.9206 | 2.2526 | 3.1267 | 0.7959 | 1.7245 |
| Gorai.006G211000 | -0.0132 | -0.0315 | -0.0223 | -0.1367 | -0.0605 | 0.2672 | 0.3424 | 0.2380 | 0.3324 | 0.0253 |
| Gorai.006G211100 | 0.0000 | 0.0000 | 0.0000 | 0.0000 | 0.0000 | 0.0000 | -0.5229 | 0.0000 | 0.0000 | 0.0000 |
| Gorai.006G211200 | 0.0000 | 0.0000 | -1.0458 | -0.4089 | 0.0000 | 0.0000 | -0.8239 | 0.0000 | 0.0000 | 0.0000 |
| Gorai.006G211300 | 1.5557 | 1.5816 | 1.6192 | 1.6477 | 1.4897 | 2.0215 | 1.4610 | 1.7127 | 1.6115 | 1.7370 |
| Gorai.006G211400 | 0.5132 | -0.0655 | 0.2742 | 0.4378 | 0.7259 | 0.7701 | 0.6325 | 0.8382 | 0.7966 | 0.8116 |
| Gorai.006G211500 | 0.0000 | -0.9586 | -0.3372 | -0.2366 | 0.1399 | 0.5237 | -0.6576 | -0.1938 | -0.3979 | -0.1024 |
| Gorai.006G212700 | 0.9031 | 0.9768 | 0.7308 | 0.8645 | 0.8162 | 1.2667 | 2.0427 | 1.4673 | 1.2553 | 1.5084 |
| Gorai.006G212800 | 0.4728 | 0.6848 | 0.4487 | 0.7672 | 0.8482 | 0.5340 | 1.2716 | 0.7846 | 0.6191 | 0.0000 |
| Gorai.006G212900 | -1.6990 | 0.0000 | -2.0000 | 0.0000 | -0.9208 | -0.3768 | 0.4232 | 0.4683 | 0.0000 | 0.0000 |
| Gorai.006G220000 | 0.0000 | -0.0706 | -0.9208 | 0.0000 | -0.7959 | -1.0969 | 0.4314 | 0.9763 | -0.7212 | 0.0000 |
| Gorai.006G220100 | 1.0903 | 0.6946 | 0.6522 | 0.5635 | 0.2742 | 0.7218 | 1.3062 | 1.2304 | 1.1735 | 1.2188 |
| Gorai.006G220200 | 0.3962 | 0.0000 | -0.7696 | -0.3098 | -0.8239 | -0.5086 | 0.0899 | -1.3010 | -1.0969 | 0.0000 |
| Gorai.006G220300 | 0.8445 | 1.0065 | 0.9101 | 0.8774 | 1.0656 | 1.0817 | 1.1059 | 1.1970 | -0.6198 | 0.0000 |
| Gorai.006G220400 | 0.7679 | 0.7796 | 0.8344 | 0.9299 | 1.0195 | 0.6928 | 0.7033 | 0.7767 | 0.5670 | 0.8663 |
| Gorai.006G220500 | 1.6466 | 1.6382 | 1.4877 | 1.3489 | 1.2753 | 1.3518 | 1.8727 | 2.2039 | 1.7227 | 1.8672 |
| Gorai.006G222400 | 0.8591 | 0.0000 | 0.0086 | 0.9717 | 0.5403 | 0.0334 | 1.4692 | 1.8516 | 0.4900 | 0.5038 |
| Gorai.006G222500 | 0.2175 | 0.5211 | -0.1739 | 0.1430 | 0.1303 | -0.0555 | 0.0000 | -0.2291 | -0.6576 | 0.0000 |
| Gorai.006G222600 | 0.7945 | 1.3608 | 1.2106 | 1.3608 | 1.2365 | 1.0017 | 0.0000 | 0.0755 | -0.1427 | 0.1703 |
| Gorai.006G222700 | 0.3655 | 0.1038 | 0.7672 | 0.5763 | 0.6758 | 0.4487 | 0.6474 | 0.7973 | 0.9694 | 0.3674 |
| Gorai.006G233100 | -0.6990 | -0.8861 | -0.2676 | -0.1308 | -0.8239 | -0.6778 | -0.2007 | -0.6778 | -0.7447 | -0.6778 |
| Gorai.006G233200 | 0.0000 | 0.0000 | 0.0000 | 0.0000 | 0.0000 | 0.0000 | -1.2218 | 0.0000 | 0.0000 | 0.0000 |
| Gorai.006G233300 | 0.2068 | 0.1959 | -0.0132 | 0.3560 | -0.1675 | 0.0719 | 1.6121 | 1.0652 | 0.0000 | -2.0000 |
| Gorai.006G233400 | 0.5843 | 0.3222 | 0.8048 | 0.4624 | 0.5866 | 0.9675 | 0.7372 | 1.5667 | -0.1487 | 1.8270 |
| Gorai.006G233500 | 0.9335 | 0.5563 | 0.8669 | 0.3579 | 0.8525 | 0.8854 | -1.3010 | -1.0969 | -0.8239 | 0.0000 |
| Gorai.006G233600 | 2.1832 | 1.7576 | 1.9430 | 1.2480 | 1.7646 | 1.6034 | 0.2041 | 1.3744 | 1.4861 | 1.1245 |
| Gorai.006G233700 | 0.0000 | 0.0000 | 0.0000 | 0.4249 | -0.8539 | 0.0000 | -0.8861 | 0.4298 | 0.0000 | 0.1523 |
| Gorai.006G247900 | 0.0000 | -0.8861 | -1.3979 | -0.2596 | -0.0655 | -0.2218 | 0.0000 | 0.0000 | 0.0000 | 0.0000 |
| Gorai.006G248000 | 1.2222 | 1.0990 | 0.9133 | 1.0523 | 1.0892 | 0.9713 | 1.2327 | 0.8837 | 0.9542 | 0.7143 |
| Gorai.006G248100 | 0.0000 | 0.0000 | 0.0000 | 0.0000 | 0.0000 | 0.0000 | -0.8861 | 0.0000 | 0.0000 | 0.0000 |
| Gorai.006G248200 | 0.0000 | 0.0000 | 0.0000 | 0.0000 | -0.7696 | 0.0000 | 0.0000 | 0.0000 | 0.0000 | 0.0000 |
| Gorai.006G248300 | -0.4949 | 0.0000 | 0.0000 | 0.0000 | 0.0000 | -1.0969 | -1.0458 | 0.0000 | 0.0000 | 0.0000 |
| Gorai.006G248400 | 0.4518 | -0.2924 | 0.3139 | 0.2148 | 0.1987 | -0.0969 | 0.2833 | 0.4409 | 0.5539 | 0.1875 |
| Gorai.006G248500 | 0.6243 | 0.5132 | 0.6990 | 0.4742 | 0.7818 | 0.3304 | 0.0719 | -0.2757 | -0.0088 | 0.0000 |
| Gorai.006G248600 | 0.0000 | 0.0000 | 0.0000 | 0.0000 | 0.0000 | 0.0000 | -0.3979 | 0.0000 | 0.0000 | 0.0000 |
| Gorai.006G248700 | 1.4813 | 1.5973 | 1.6469 | 1.3450 | 1.3292 | 1.2586 | 1.9597 | 1.7886 | 1.6038 | 1.8656 |
| Gorai.006G248800 | -0.4318 | -0.5686 | -0.4202 | 0.3181 | -0.9208 | -1.0969 | -0.3372 | 0.3541 | 0.5922 | -0.5376 |
| Gorai.006G248900 | 0.0000 | 0.0000 | 0.0000 | 0.0000 | 0.0000 | 0.0000 | 0.0000 | 0.0000 | 0.0000 | 0.0000 |
| Gorai.006G249000 | 0.0934 | 0.0000 | -1.3979 | -0.1871 | 0.0792 | 0.2014 | 0.7910 | 0.8802 | -2.0000 | 0.0000 |
| Gorai.006G249100 | 0.0000 | -0.9208 | -1.6990 | 0.0000 | 0.0000 | 0.0000 | 0.3820 | 0.0000 | 0.0000 | 0.0000 |
| Gorai.006G249200 | 0.0000 | 0.0000 | 0.0000 | 0.0000 | 0.0000 | 0.0000 | 0.7731 | 0.0000 | 0.0000 | 0.0000 |
| Gorai.006G249300 | 0.0000 | 0.0000 | 0.0000 | 0.0000 | 0.0000 | 0.0000 | 0.0000 | 0.0000 | 0.0000 | 0.0000 |
| Gorai.006G249400 | -0.1938 | 0.6096 | 0.2833 | 0.5276 | 0.2304 | 0.0607 | 1.6194 | 1.0170 | 1.0386 | 0.9212 |
| Gorai.007G005400 | 0.2577 | 0.7084 | 0.5328 | 0.6946 | 0.4065 | 0.7152 | 1.6356 | 0.8202 | 0.7910 | 0.7825 |
| Gorai.007G005500 | 0.0000 | 0.0000 | -0.5376 | -0.7696 | -0.6990 | 0.0000 | 1.2380 | 1.0986 | 0.0000 | 0.0000 |
| Gorai.007G005600 | 0.0000 | 0.0000 | 0.1303 | 0.2304 | 0.0000 | 0.4031 | 2.1149 | 1.8044 | 0.0000 | 0.0000 |
| Gorai.007G005700 | 1.1599 | -0.7696 | 0.6435 | -0.5376 | -0.3979 | 0.1106 | 0.0719 | -0.6576 | -0.3872 | 0.0000 |
| Gorai.007G005800 | 0.0000 | -0.4437 | 0.0569 | -1.0000 | 0.1492 | -0.5850 | -0.3098 | 0.0000 | 0.0000 | 0.0000 |
| Gorai.007G005900 | 0.0000 | 0.0000 | 0.0000 | -0.7696 | 0.0000 | 0.0000 | -0.2518 | -0.7696 | 0.0000 | 0.0000 |
| Gorai.007G007000 | -0.4685 | -0.3979 | 0.0374 | 0.0792 | 0.0864 | -0.0177 | 0.8739 | -0.4559 | -0.1805 | 0.0000 |
| Gorai.007G007100 | 0.0000 | -0.3098 | -0.3468 | -0.0655 | -0.4949 | 0.0000 | 1.1103 | 1.0441 | 0.1584 | 0.0000 |
| Gorai.007G007200 | 0.5353 | 0.7243 | 0.6075 | 0.6355 | 0.5866 | 0.8235 | 0.9533 | 0.9445 | 0.6990 | 1.0508 |
| Gorai.007G007300 | 0.0000 | 0.0000 | 0.0000 | -0.3768 | -0.9586 | -0.7696 | 1.2172 | 0.0755 | -0.3768 | 0.2480 |
| Gorai.007G007400 | 0.6325 | 0.8035 | 0.6893 | 0.7152 | 0.7267 | 0.6274 | 0.5966 | 0.6955 | 0.7993 | 0.4472 |
| Gorai.007G007500 | 0.0000 | 0.0000 | 0.0000 | 0.0000 | 0.0000 | 0.0000 | -0.3565 | 0.0000 | 0.0000 | 0.0000 |
| Gorai.007G022200 | -0.2757 | -0.1871 | -0.9586 | -0.3468 | -1.5229 | 0.0000 | -2.0000 | 0.0000 | 0.0000 | 0.0000 |
| Gorai.007G022300 | 1.1655 | 0.9934 | 1.2274 | 1.2276 | 1.2942 | 1.4079 | 1.2049 | 0.9504 | 0.4472 | 0.4082 |
| Gorai.007G022400 | 0.0000 | 0.0000 | 0.0000 | -0.6990 | 0.0000 | 0.0000 | -0.4559 | -0.8539 | 0.0000 | 0.0000 |
| Gorai.007G022500 | 0.9269 | 1.0821 | 0.9881 | 0.9355 | 0.9841 | 0.9863 | 0.8028 | 1.0052 | 0.8698 | 1.0531 |
| Gorai.007G022600 | 2.3848 | 2.3261 | 1.9599 | 1.6659 | 1.6775 | 1.2993 | 1.2949 | 1.4352 | 1.4577 | 1.3218 |
| Gorai.007G022700 | 0.0000 | 0.0000 | -0.1427 | 0.0170 | 1.0973 | 0.8299 | 0.0453 | 0.6042 | 0.6253 | 0.5092 |
| Gorai.007G022800 | 0.7210 | 1.0434 | 0.8414 | 0.6911 | 0.8971 | 1.0212 | 1.1841 | 0.8109 | 0.7910 | 0.7627 |
| Gorai.007G033100 | -0.5686 | -0.1367 | -0.1739 | -0.2518 | 0.0000 | 0.0000 | 0.6920 | 1.2558 | 0.0000 | 0.0000 |
| Gorai.007G033200 | -2.0000 | -2.0000 | -2.0000 | -2.0000 | -2.0000 | -1.6990 | 0.5315 | 1.3253 | 0.0000 | -2.0000 |
| Gorai.007G033300 | 0.3617 | 0.3284 | 0.7300 | 0.5302 | 0.7177 | 0.6981 | 0.6493 | 0.4362 | 0.1931 | 0.5416 |
| Gorai.007G033400 | -0.1675 | -0.1487 | 0.0000 | 0.3560 | -0.3768 | -0.1024 | 1.4726 | 1.4370 | 1.2801 | 0.1367 |
| Gorai.007G033500 | 0.3979 | 0.7210 | 0.9069 | 0.8591 | 0.2601 | 0.6274 | 2.1349 | 1.1268 | 0.1875 | 0.7559 |
| Gorai.007G033600 | 0.7745 | 0.9657 | 1.0228 | 1.0828 | 1.1364 | 0.9921 | 1.1351 | 0.9154 | 0.8312 | 0.8176 |
| Gorai.007G033700 | 1.3806 | 1.6897 | 1.4666 | 1.3909 | 1.3410 | 1.2390 | 1.6401 | 1.2574 | 0.7135 | 1.4499 |
| Gorai.007G043500 | 0.0000 | 0.0000 | 0.0000 | -0.3188 | 0.0000 | 0.0000 | 0.6222 | 0.6053 | 0.0000 | 0.0000 |
| Gorai.007G043600 | 1.9457 | 1.6154 | 1.8508 | 2.0618 | 1.7847 | 2.0858 | 2.1061 | 2.0448 | 2.1332 | 1.7684 |
| Gorai.007G043700 | 0.0000 | 0.0000 | 0.0000 | -0.6021 | 0.0000 | 0.0000 | -1.1549 | -0.6778 | -0.4202 | 0.0000 |
| Gorai.007G043800 | 0.0000 | 0.0000 | 0.0000 | 0.0000 | 0.0000 | 0.0000 | 0.0000 | 0.0000 | 0.0000 | 0.0000 |
| Gorai.007G043900 | -0.4815 | -0.5528 | -0.1427 | -1.0458 | -1.0969 | -0.7212 | 0.3617 | -0.5229 | -0.2596 | 0.0000 |
| Gorai.007G044000 | -0.2924 | -0.3372 | -0.1805 | -0.4202 | -0.6383 | -0.9586 | 0.2900 | 0.8525 | 0.3909 | 1.0282 |
| Gorai.007G050300 | 0.0000 | 0.0000 | 0.0000 | 0.0000 | 0.0000 | 0.0000 | 0.7185 | 1.1396 | 0.0000 | 0.0000 |
| Gorai.007G050400 | -0.2007 | 0.2529 | 0.2304 | -0.6021 | -0.3098 | -0.5376 | -0.4437 | -0.4559 | -0.4559 | -0.5376 |
| Gorai.007G050500 | -0.0458 | -0.7447 | -0.0862 | 0.1931 | 1.0810 | 0.7574 | 0.0531 | -0.1427 | -0.0044 | -0.4437 |
| Gorai.007G050600 | 0.0000 | -0.6198 | -0.6198 | -1.0969 | 0.3385 | -0.1249 | -0.7212 | -0.2366 | 0.0000 | 0.0253 |
| Gorai.007G050700 | 0.0000 | 0.0000 | -0.0362 | 0.0000 | 0.2122 | 0.0000 | 0.2833 | 0.5988 | 0.0000 | 0.0000 |
| Gorai.007G050800 | 1.4396 | 2.2005 | 1.5616 | 1.7287 | 1.9079 | 1.6184 | 0.4857 | 1.3160 | 0.5428 | 1.5395 |
| Gorai.007G050900 | 0.0000 | -1.0969 | -1.5229 | 0.5502 | 1.0573 | 0.7752 | 0.9440 | 0.2648 | 0.4456 | -0.2007 |
| Gorai.007G062900 | -2.0000 | -1.6990 | 0.1271 | 0.3404 | 1.2550 | 1.1351 | 1.0461 | 0.7760 | 0.5490 | 0.7566 |
| Gorai.007G063000 | 1.0257 | 1.4654 | 1.1383 | 1.2744 | 1.9780 | 2.0173 | 1.0484 | 1.4436 | 1.3402 | 1.4694 |
| Gorai.007G063100 | 0.0000 | 0.0000 | 0.0000 | 0.0000 | 0.0000 | -1.3979 | 0.0000 | -0.6021 | -1.0458 | -0.4318 |
| Gorai.007G063200 | 0.0000 | -0.2007 | 0.0000 | 0.0000 | 0.0000 | -0.5086 | 0.0000 | 0.7427 | 1.0128 | 0.0000 |
| Gorai.007G063300 | 0.0000 | 0.0000 | 0.0000 | 0.0000 | 0.0000 | 0.0000 | 0.0000 | -1.1549 | 0.0000 | 0.0000 |
| Gorai.007G063400 | 0.0000 | 0.0000 | -1.6990 | 0.0000 | 0.0000 | 0.0000 | -2.0000 | 0.0000 | 0.0000 | 0.0000 |
| Gorai.007G063500 | 0.7839 | 1.0069 | 1.2011 | 1.1761 | 1.2135 | 1.3336 | 1.1045 | 1.4247 | 1.3703 | 1.4108 |
| Gorai.007G063600 | 2.0852 | 2.2574 | 1.9768 | 1.9824 | 1.8154 | 1.6825 | 1.9530 | 2.2737 | 1.9440 | 2.0746 |
| Gorai.007G064100 | 0.0000 | 1.5087 | -0.3768 | -0.7696 | 0.1173 | 0.5011 | 1.4071 | 0.9643 | 0.0000 | 0.0000 |
| Gorai.007G064200 | 0.0000 | -1.0000 | -1.0969 | -0.2757 | -0.3665 | 2.2018 | -1.0000 | -0.5850 | -0.9586 | -0.4318 |
| Gorai.007G064300 | 0.0000 | 0.0000 | 0.0000 | 0.0000 | 0.0000 | 1.2704 | -1.3979 | 0.0000 | 0.0000 | 0.0000 |
| Gorai.007G064400 | 0.0000 | 0.0000 | 0.0000 | 0.0000 | 0.0000 | -0.5528 | 0.0000 | 0.0000 | 0.0000 | 0.0000 |
| Gorai.007G064500 | 1.2882 | 0.0000 | 1.2458 | 0.9961 | 1.3854 | 1.3842 | 0.7490 | 0.9590 | 0.7316 | 0.8055 |
| Gorai.007G069300 | -1.0969 | 0.5911 | -0.0605 | -0.3565 | 0.3692 | 0.3838 | 1.4052 | 1.1173 | 0.6160 | 0.5587 |
| Gorai.007G069400 | 0.0000 | 0.0000 | 0.0000 | -1.0000 | 0.0000 | 0.0000 | -1.0000 | -1.0458 | -0.7696 | 0.0000 |
| Gorai.007G069500 | 0.0000 | -1.6990 | -0.8239 | 0.6415 | 2.2727 | 3.1388 | -0.2218 | -0.5686 | -0.7447 | 0.0000 |
| Gorai.007G069600 | 1.6115 | 1.7444 | 1.4876 | 1.3522 | 1.5559 | 1.5730 | 1.1041 | 1.2243 | 1.0095 | 1.3043 |
| Gorai.007G069700 | 2.4242 | 1.5871 | 1.8706 | 1.4706 | 0.9759 | 0.4983 | 1.3568 | 1.8015 | 0.9154 | 1.4140 |
| Gorai.007G069800 | 1.5373 | 0.8603 | 1.6953 | 1.8496 | 1.7747 | 1.3791 | 1.2610 | 1.5744 | 1.8077 | 0.7482 |
| Gorai.007G069900 | 0.9258 | 0.9974 | 0.8338 | 0.7723 | 0.8299 | 1.0667 | 1.4971 | 1.5759 | 1.0233 | 1.0565 |
| Gorai.007G070000 | 0.8149 | 0.1959 | 0.7536 | 0.1271 | -0.2007 | -0.1079 | 0.4843 | -0.2518 | -0.6576 | -0.0862 |
| Gorai.007G070100 | 0.5740 | -0.2676 | 0.3010 | 0.0212 | -0.5686 | -0.2518 | 0.2355 | -1.0458 | -0.7959 | 0.0000 |
| Gorai.007G084600 | 0.0000 | 0.0000 | -0.0809 | 0.0000 | -2.0000 | 0.0000 | 1.4330 | 0.7482 | 0.7774 | 0.0000 |
| Gorai.007G084700 | 0.0000 | 0.2253 | -0.2441 | -0.1427 | 0.0531 | 0.0000 | 0.3541 | 0.7466 | 0.0000 | 0.4518 |
| Gorai.007G084800 | 0.2601 | 0.6721 | 0.0934 | 0.6444 | 0.0645 | -0.2441 | -0.2676 | 0.1703 | -0.4202 | 0.3711 |
| Gorai.007G084900 | 0.8109 | 0.9455 | 0.8627 | 0.7168 | 0.9405 | 0.9085 | 1.0052 | 1.2400 | 1.1351 | 1.2655 |
| Gorai.007G085000 | 0.3874 | 0.5079 | -0.3768 | 0.8681 | 1.2206 | 0.7243 | 0.0000 | 0.0000 | 0.0000 | 0.0000 |
| Gorai.007G085100 | 0.0086 | 0.5752 | -0.3768 | -0.2924 | 0.0000 | -0.6198 | 0.7235 | 1.2443 | 0.0000 | 0.0000 |
| Gorai.007G087300 | 0.0000 | 0.0000 | -0.7696 | 0.0000 | -0.5376 | 0.0864 | -0.0177 | -0.0410 | 0.0000 | 0.0000 |
| Gorai.007G087400 | 0.0000 | -0.4202 | 0.0000 | -0.3665 | -0.2007 | -0.6778 | 1.3549 | -0.3768 | 0.0000 | -0.1135 |
| Gorai.007G087500 | 0.0000 | 0.8579 | 0.0000 | 0.4216 | 0.1931 | -0.3565 | 0.0000 | 0.0000 | 0.0000 | 0.0000 |
| Gorai.007G087600 | 0.3692 | 0.2529 | 0.2923 | 0.5391 | 0.5391 | 0.8451 | 0.6542 | 0.2967 | 0.4928 | -0.2441 |
| Gorai.007G087700 | 0.0000 | 0.4843 | -0.3468 | -0.2518 | -0.2218 | -0.5229 | 1.4403 | 1.0370 | 0.0000 | 0.0000 |
| Gorai.007G087800 | 0.0000 | 0.0000 | -0.9208 | 0.4150 | 0.1038 | 0.2923 | 1.4577 | 1.0785 | 0.0000 | 0.0000 |
| Gorai.007G090200 | 0.0000 | 0.0000 | 0.0000 | 0.0000 | 0.0000 | 0.0000 | -1.1549 | 0.0000 | 0.0000 | 0.0000 |
| Gorai.007G090300 | 0.7380 | 0.9309 | 0.8971 | 0.7938 | 0.9675 | 1.0056 | 1.4829 | 2.2323 | 2.0079 | 2.3230 |
| Gorai.007G090400 | 1.4244 | 1.6073 | 1.4747 | 1.5173 | 1.3251 | 0.9717 | 1.3760 | 1.2755 | 1.2330 | 1.2507 |
| Gorai.007G090500 | 0.6355 | 0.7126 | 0.7076 | 0.8274 | 0.7356 | 0.7612 | 0.7482 | 0.9479 | 1.0237 | 0.7701 |
| Gorai.007G090600 | 1.7572 | 1.7048 | 1.8733 | 1.8404 | 1.6571 | 1.9675 | 1.8463 | 1.6567 | 1.7517 | 1.4398 |
| Gorai.007G090700 | 0.1206 | 0.6637 | 0.2380 | 0.3979 | 0.1903 | 0.0294 | 1.2063 | 0.4200 | 0.6599 | -0.4949 |
| Gorai.007G090800 | -0.3010 | -0.0458 | -0.4815 | 0.4683 | -0.0223 | 1.5755 | -1.6990 | -0.3565 | 0.0000 | 0.0000 |
| Gorai.007G090900 | 0.6920 | 0.2601 | 0.7210 | 0.5786 | 0.4314 | 0.4579 | 1.5489 | 1.2524 | 0.2095 | 0.3444 |
| Gorai.007G091000 | -0.8861 | -0.9208 | -1.0969 | -0.8539 | -1.0969 | 0.0000 | -0.5686 | -1.2218 | -0.9586 | 0.0000 |
| Gorai.007G091100 | 0.4594 | 0.6395 | 0.7513 | 0.8704 | 0.7251 | 1.0128 | 0.9149 | 0.9983 | 1.0199 | 0.8960 |
| Gorai.007G093500 | 1.1143 | 1.1287 | 1.1807 | 1.0637 | 0.7474 | 0.6561 | 1.2629 | 1.3267 | 0.8751 | 1.5002 |
| Gorai.007G093600 | 1.9174 | 1.5467 | 1.8563 | 1.8903 | 1.7088 | 1.7663 | 1.6189 | 1.7610 | 1.7828 | 1.6112 |
| Gorai.007G093700 | 1.2430 | 0.5428 | 1.9442 | 1.4695 | 1.5257 | 0.9552 | -0.1675 | 1.8602 | 1.7024 | 1.9153 |
| Gorai.007G093800 | 0.0000 | 0.0000 | 0.0000 | 0.0000 | -0.3979 | -0.4685 | 0.8751 | -0.4559 | 0.0000 | 0.0000 |
| Gorai.007G099100 | -0.7447 | 0.0000 | -0.5229 | -0.3279 | -0.1487 | -0.8239 | 0.5119 | 0.2765 | 0.0000 | 0.0000 |
| Gorai.007G099200 | 0.8768 | 1.2548 | 1.0700 | 1.0504 | 1.0056 | 0.7160 | 0.5752 | 0.6776 | 0.7332 | 0.5340 |
| Gorai.007G099300 | 0.1732 | 0.0000 | -0.6778 | -0.0362 | 0.5717 | -0.3468 | 0.4713 | 0.8228 | -0.0315 | 0.8831 |
| Gorai.007G099400 | 0.0000 | 0.0000 | -0.3665 | -0.1367 | -0.7447 | -0.8861 | 0.0755 | -0.1308 | -0.1192 | 0.0000 |
| Gorai.007G099500 | -0.2218 | -0.2366 | -0.3979 | -0.1249 | -0.3872 | -0.2757 | 0.6232 | 1.0366 | -0.2840 | -0.4949 |
| Gorai.007G099600 | 0.9494 | 1.1119 | 1.4163 | 1.2087 | 1.0997 | 0.7973 | 0.9370 | 1.6436 | 1.5488 | 1.6629 |
| Gorai.007G128700 | 0.9186 | 1.0469 | 1.0810 | 1.2653 | 1.3019 | 1.3021 | 0.8457 | 1.4135 | 1.0558 | 1.5616 |
| Gorai.007G193400 | -0.8861 | -1.0458 | -0.7959 | -0.5850 | 0.0000 | -1.3979 | 1.1691 | -0.2366 | -0.4202 | -0.1549 |
| Gorai.007G193500 | 0.0000 | 0.0000 | 0.0000 | 0.0000 | 0.0000 | 0.0000 | -0.8539 | -0.5850 | -0.3098 | 0.0000 |
| Gorai.007G193600 | 0.0000 | 0.8280 | -0.6576 | 0.5478 | -0.3665 | 0.1931 | 0.9736 | -0.7212 | -0.4559 | 0.0000 |
| Gorai.007G221900 | 0.0000 | 0.3139 | 0.0000 | 0.8692 | -0.2596 | -0.2291 | 0.7760 | 0.0000 | 0.0000 | 0.0000 |
| Gorai.007G222800 | -0.1938 | -0.6198 | -0.6021 | -0.3872 | -0.6021 | -1.0458 | -1.6990 | -0.8861 | -0.6383 | 0.0000 |
| Gorai.007G222900 | -0.2291 | -0.0706 | 0.0864 | 0.5353 | -0.4202 | -0.3468 | 0.3444 | 0.7604 | 0.8102 | 0.6243 |
| Gorai.007G229800 | -0.4949 | 0.4564 | 0.2625 | -0.1675 | -0.6990 | 0.5647 | 0.4393 | 0.3598 | -0.2366 | 0.5623 |
| Gorai.007G235800 | -0.3872 | -0.1739 | 0.4409 | 0.8739 | 0.9890 | 1.4755 | 1.2365 | 1.1225 | 0.1903 | 1.3595 |
| Gorai.007G236900 | -0.1024 | -0.0605 | 0.0000 | 0.0000 | 0.0000 | -0.6576 | -0.0506 | 0.0000 | 0.0000 | 0.0000 |
| Gorai.007G237000 | 0.0000 | 0.0000 | -0.2147 | -0.3468 | 0.0000 | 0.0000 | 0.4609 | 0.6474 | -2.0000 | -2.0000 |
| Gorai.007G246600 | 0.5237 | 0.0000 | 0.7853 | 0.7412 | 0.8445 | 0.5623 | 1.1364 | 0.8727 | 0.4082 | 0.0374 |
| Gorai.007G265200 | 0.0000 | 0.0000 | 0.0000 | 0.0000 | -0.6778 | 0.0000 | 0.0043 | -0.4685 | 0.0000 | 0.0000 |
| Gorai.007G272800 | 1.0107 | 0.6911 | 1.0959 | 0.9128 | 0.9020 | 1.3895 | 1.0086 | 0.3541 | 0.2900 | 0.3483 |
| Gorai.007G273100 | -1.0969 | 0.0000 | -0.8861 | -1.6990 | -1.3010 | 0.0000 | 1.4428 | -0.3468 | -0.6383 | -0.2147 |
| Gorai.007G273900 | 0.0864 | 0.2672 | 0.1644 | -0.0757 | -0.1805 | 0.2529 | 0.4298 | 0.4579 | 0.0000 | 0.0000 |
| Gorai.007G274000 | 0.8445 | 1.6169 | 0.8136 | 0.9465 | 1.0881 | 1.1186 | 0.9703 | 1.5045 | 1.2011 | 1.6343 |
| Gorai.007G274400 | 0.2900 | 0.6503 | 0.6314 | 0.4857 | 0.6454 | 0.3345 | 0.2041 | 0.1038 | -0.0506 | 0.1673 |
| Gorai.007G274500 | -0.6383 | -1.0000 | -0.7959 | -1.0458 | -1.0969 | -1.0458 | -0.2076 | -0.3279 | -1.0458 | -0.1079 |
| Gorai.007G274600 | 0.5798 | 0.1847 | 0.2122 | -0.3565 | 0.1584 | 0.4728 | -0.0223 | -0.3188 | -0.8239 | -0.1367 |
| Gorai.007G274700 | 0.0000 | 0.0000 | -0.5850 | 0.0000 | 0.0212 | -0.0315 | 0.8733 | 0.9952 | -2.0000 | 0.0000 |
| Gorai.007G274800 | 0.1703 | 0.3802 | 0.3820 | 0.3874 | 0.6656 | 0.7520 | 0.7528 | 0.9170 | 0.6702 | 0.0000 |
| Gorai.007G274900 | 0.2718 | 0.0569 | 0.2742 | 0.6405 | 0.3385 | 0.3404 | 0.8543 | 0.5353 | 0.7938 | -0.7696 |
| Gorai.007G278200 | 0.0000 | 0.0000 | -1.0458 | -0.9208 | 0.0000 | 0.0000 | -0.1549 | 0.0000 | 0.0000 | 0.0000 |
| Gorai.007G278300 | 0.0000 | 0.0000 | 0.0000 | 0.0000 | 0.0000 | 0.0000 | 0.0000 | 0.0000 | 0.0000 | 0.0000 |
| Gorai.007G278600 | 0.7135 | 0.9713 | 1.1617 | 0.7340 | 1.0626 | 0.7810 | 1.1355 | 1.4237 | 1.0370 | 1.5805 |
| Gorai.007G278700 | 0.0645 | -0.8539 | 0.0682 | 0.0334 | -0.7959 | -0.8539 | -0.3188 | 0.0531 | 0.2279 | -0.3979 |
| Gorai.007G278800 | 0.0000 | 0.0000 | 0.0000 | 0.0000 | 0.0000 | 0.0000 | 1.4309 | 1.1572 | 0.0000 | 0.0000 |
| Gorai.007G278900 | 0.3541 | 0.5079 | 0.4330 | 0.6222 | 0.4871 | 0.5465 | 0.4983 | 0.9624 | 0.8062 | 1.0228 |
| Gorai.007G280200 | -0.3872 | -0.2840 | -0.0088 | -0.0410 | 0.0294 | 0.0682 | 0.7050 | 0.6335 | 0.5775 | 0.4265 |
| Gorai.007G280400 | 0.0000 | -0.8861 | 0.0000 | 0.0755 | 0.6911 | 1.1602 | 0.0000 | 0.0000 | 0.0000 | 0.0000 |
| Gorai.007G280500 | 0.0000 | 0.0000 | -0.4202 | 0.0000 | 0.0000 | 0.0000 | 1.3237 | 1.1937 | 0.6075 | 0.0000 |
| Gorai.007G280600 | -0.6021 | -1.3010 | -1.2218 | -0.8539 | -0.5086 | -0.4318 | 0.2810 | -0.6576 | -0.3872 | 0.0000 |
| Gorai.007G286400 | 0.0000 | 0.7202 | 0.6405 | 0.6920 | -2.0000 | 0.1492 | 0.5763 | 1.1658 | 0.6893 | 0.5563 |
| Gorai.007G287300 | 1.7982 | 0.5502 | 2.3776 | 1.1892 | 0.3010 | -0.3010 | 1.5431 | 1.7789 | 1.8870 | 1.5368 |
| Gorai.007G287400 | 2.8459 | 2.3564 | 2.3463 | 1.7462 | 0.7042 | 1.0867 | 0.8445 | 1.2693 | 1.0398 | 1.3688 |
| Gorai.007G287500 | -0.6021 | -1.1549 | -0.4318 | -0.8539 | -0.7696 | -0.7447 | 0.8549 | -0.5229 | -0.7696 | -0.4202 |
| Gorai.007G287600 | -0.7447 | 0.0000 | 0.5478 | -1.1549 | 0.6064 | 0.1173 | 0.7903 | 1.0896 | -0.3372 | 0.3424 |
| Gorai.007G287700 | 0.3365 | 1.0382 | 0.6294 | 1.0607 | 0.8704 | 1.2162 | 1.1449 | 0.8549 | 0.7789 | 0.8591 |
| Gorai.007G287800 | 0.9133 | 0.1673 | 0.4183 | 1.1319 | 1.1945 | 1.1967 | 1.5179 | 0.9504 | 0.7709 | 1.0245 |
| Gorai.007G287900 | -0.1024 | 0.0000 | -0.3279 | 0.2279 | 0.1673 | -0.2218 | 1.3314 | 0.8000 | 0.5599 | -0.0757 |
| Gorai.007G288000 | 0.0000 | 0.0000 | 0.0000 | 0.0000 | 0.0000 | 0.0000 | 1.9495 | 1.3233 | 0.0000 | 0.0000 |
| Gorai.007G288100 | 0.3222 | 0.7559 | 0.1271 | 0.4639 | 0.3711 | 0.6444 | 0.0414 | 0.1959 | 0.0253 | 0.2648 |
| Gorai.007G304300 | -0.1427 | 0.6053 | 0.3945 | 0.1367 | 0.5502 | 0.6031 | 1.1290 | 1.0120 | 0.5441 | 0.6590 |
| Gorai.007G304400 | 1.0314 | 1.5024 | 1.3818 | 1.9839 | 2.4624 | 2.2490 | 1.1813 | 1.5432 | 1.1103 | 1.6799 |
| Gorai.007G304500 | 0.9186 | 0.3263 | 1.2728 | 1.6362 | 1.8138 | 1.5413 | 1.2017 | 1.4130 | 0.7853 | 1.3655 |
| Gorai.007G304600 | 2.0652 | 2.0131 | 1.7930 | 1.6246 | 1.5213 | 1.4111 | 1.6336 | 1.4104 | 1.4962 | 1.2138 |
| Gorai.007G304700 | 0.0000 | 0.0000 | 0.0000 | 0.0000 | 0.0000 | 0.0000 | 0.0000 | 0.0000 | 0.0000 | 0.0000 |
| Gorai.007G306000 | 1.0878 | 0.9165 | 1.0584 | 1.0175 | 0.8476 | 0.9274 | 1.3174 | 1.0770 | 0.8494 | 1.1755 |
| Gorai.007G306100 | 0.0000 | 0.0000 | 0.0000 | -0.8539 | 0.0000 | 0.0000 | -1.6990 | 0.0000 | 0.0000 | 0.0000 |
| Gorai.007G306200 | -0.3768 | 0.4728 | 0.2553 | 0.0128 | 0.2810 | 0.4728 | 0.6839 | 0.5250 | -0.4685 | 0.1004 |
| Gorai.007G306300 | -0.1739 | -0.1675 | 0.0170 | -0.1549 | -0.1427 | -0.1549 | -0.0132 | 0.3139 | 0.4942 | -0.1549 |
| Gorai.007G307100 | -1.3979 | 0.0000 | -0.0177 | -1.3010 | 0.0000 | 0.5729 | 0.8426 | 0.9217 | -0.0458 | 0.0000 |
| Gorai.007G307200 | -1.2218 | -0.4815 | 0.2765 | 0.2718 | 1.1436 | 0.7007 | -0.2596 | 0.5237 | 0.5416 | 0.4314 |
| Gorai.007G307300 | 0.4533 | 1.0542 | 0.0792 | 0.0000 | 0.1139 | 1.0663 | 1.1629 | 0.0253 | 0.2967 | 0.0000 |
| Gorai.007G307400 | 0.0000 | 0.0000 | 0.0000 | 0.0000 | 0.0000 | 0.0000 | 0.0000 | 0.0000 | 0.0000 | 0.0000 |
| Gorai.007G318500 | 0.0000 | 0.0000 | 0.0000 | 0.0000 | 0.0000 | 0.0000 | -0.7447 | 0.1072 | 0.3304 | 0.0000 |
| Gorai.007G318600 | 0.0828 | 0.0000 | 0.0000 | -0.6021 | 0.0000 | 0.0000 | 0.0000 | 0.0000 | 0.0000 | 0.0000 |
| Gorai.007G318700 | 0.0000 | -1.6990 | -1.6990 | -1.6990 | -1.6990 | 0.0000 | 0.1761 | -0.0362 | -0.0915 | 0.0000 |
| Gorai.007G318800 | -1.2218 | 0.0000 | -1.6990 | -1.3979 | -2.0000 | 0.0000 | -1.0969 | -0.4685 | -0.9586 | -0.2840 |
| Gorai.007G318900 | 0.0000 | 0.0000 | -0.8539 | 0.0000 | 0.0000 | 0.0000 | -0.7212 | 0.7316 | 0.6848 | 0.4425 |
| Gorai.007G319300 | 0.0000 | 0.0000 | -1.5229 | -1.1549 | -2.0000 | 0.0000 | -1.0969 | -1.0969 | -1.2218 | 0.0000 |
| Gorai.007G319400 | 0.3118 | -0.1024 | 0.5250 | 0.3927 | 0.1761 | 0.0000 | 1.3064 | -0.4437 | -0.6990 | -0.6198 |
| Gorai.007G319500 | 1.1389 | 0.5092 | 0.9614 | 0.8865 | 0.4886 | 0.3181 | 1.2146 | 1.4338 | 1.3647 | 1.4325 |
| Gorai.007G319600 | 0.0000 | 0.0000 | -0.2518 | 0.5211 | 0.0000 | 0.0000 | 1.5358 | 1.1274 | 0.0000 | 0.0000 |
| Gorai.007G319700 | 0.6884 | 0.3263 | 0.7701 | 0.8837 | 0.8202 | 0.9165 | 1.1738 | 0.5224 | 0.5763 | 0.3820 |
| Gorai.007G324500 | 0.0000 | 0.0000 | 0.0000 | 0.0000 | 0.0000 | 0.0000 | 0.0000 | 0.0000 | 0.0000 | 0.0000 |
| Gorai.007G324600 | 0.2455 | -0.6198 | -0.1192 | -0.7696 | -0.9586 | -1.2218 | -0.1675 | 0.1206 | 0.2330 | -0.1308 |
| Gorai.007G324700 | 0.0000 | -1.3979 | -1.6990 | 0.0000 | 0.0000 | 0.0000 | -0.8539 | -1.6990 | -1.3979 | 0.0000 |
| Gorai.007G327600 | 0.0000 | 0.0000 | 0.0000 | 0.0000 | 0.0000 | 0.0000 | 0.0000 | 0.0000 | 0.0000 | 0.0000 |
| Gorai.007G327700 | 0.0000 | 0.0000 | 0.0000 | 0.0000 | 0.0000 | 0.0000 | 0.5038 | -0.5376 | 0.0000 | 0.0000 |
| Gorai.007G332600 | -1.3979 | -0.8539 | -0.9586 | -0.7212 | -0.3665 | -0.7212 | -0.4089 | -0.7959 | -0.7959 | -0.8539 |
| Gorai.007G332700 | -0.0862 | -0.2676 | 0.0374 | -0.0177 | -0.0506 | 0.0374 | 0.1761 | -0.2147 | -0.0177 | -0.7447 |
| Gorai.007G332800 | 0.0000 | 0.0000 | -0.8539 | -0.0969 | -0.7212 | -0.7696 | -1.0969 | -1.0969 | -0.8239 | 0.0000 |
| Gorai.007G332900 | -1.5229 | -0.6576 | -1.3979 | -1.2218 | -1.0458 | -1.3010 | -0.6778 | -1.0969 | -0.8239 | 0.0000 |
| Gorai.007G333000 | 0.0000 | 0.0000 | 0.0000 | -1.3979 | -1.5229 | -1.0969 | -0.6021 | 0.0000 | 0.0000 | 0.0000 |
| Gorai.007G333100 | 0.0000 | 0.0000 | 0.0000 | -0.2596 | 0.0000 | 0.0000 | 0.0000 | 0.0000 | 0.0000 | 0.0000 |
| Gorai.007G335100 | 0.6522 | 0.4281 | 0.7185 | 0.6053 | 0.8209 | 0.7505 | 1.0212 | 0.6294 | 0.6107 | 0.5809 |
| Gorai.007G335200 | 0.0000 | -0.0809 | 0.0294 | 0.2810 | 0.6201 | 0.5237 | 1.1720 | 0.9661 | 0.4150 | 0.3784 |
| Gorai.007G335300 | 0.7007 | 0.8280 | 0.9523 | 0.5955 | 0.6464 | 0.8267 | 0.2788 | 1.1679 | 0.6180 | 0.0000 |
| Gorai.007G340100 | 0.0000 | 0.0000 | 0.0000 | 0.0000 | 0.0000 | 0.0000 | -0.5376 | 0.0000 | 0.0000 | 0.0000 |
| Gorai.007G340200 | 0.0000 | -2.0000 | -2.0000 | 0.0000 | -1.6990 | 0.0000 | -0.6021 | 0.0000 | 0.0000 | 0.0000 |
| Gorai.007G345500 | 0.0000 | 0.1790 | 0.0043 | -0.4685 | 0.3404 | -0.0655 | 0.9170 | 0.8420 | 0.0000 | 0.0000 |
| Gorai.007G345600 | 0.0000 | 0.0000 | 0.0000 | -1.0458 | 0.0000 | -0.4949 | -1.3979 | -1.5229 | -1.2218 | 0.0000 |
| Gorai.007G345700 | 0.0000 | -0.9208 | 0.0000 | 0.0000 | 0.0000 | 0.0000 | 0.0000 | 0.0000 | 0.0000 | 0.0000 |
| Gorai.007G345800 | 0.3201 | 0.2695 | 0.1072 | -0.1079 | 0.1644 | 0.5441 | -0.1739 | -0.1739 | -0.1938 | -0.2291 |
| Gorai.007G345900 | 1.0927 | 0.7782 | 0.8228 | 0.6314 | 0.8837 | 1.0542 | 0.6474 | 1.5316 | 1.7535 | 0.8182 |
| Gorai.007G346000 | 0.0334 | 0.1523 | -0.4202 | 0.0000 | -0.2596 | -0.3665 | -0.8239 | 0.0000 | 0.0000 | 0.0000 |
| Gorai.007G346100 | 1.6109 | 2.1022 | 1.8188 | 1.4733 | 1.5314 | 1.2368 | 1.9306 | 1.4884 | 1.5823 | 1.2765 |
| Gorai.007G346200 | 0.0000 | 1.3758 | -0.8239 | 1.1717 | 0.7760 | 0.1492 | 0.0000 | -1.0000 | -0.7212 | 0.0000 |
| Gorai.007G346300 | -0.7959 | -0.3768 | -0.4815 | 0.5933 | 0.6493 | 0.5899 | -1.6990 | -0.6383 | -0.9208 | -0.5229 |
| Gorai.007G347100 | 1.1146 | 0.8921 | 1.0722 | 1.0667 | 1.1679 | 1.2232 | 0.6454 | 0.8899 | 0.7284 | 0.9542 |
| Gorai.007G347200 | 0.7474 | 0.8932 | 0.8069 | 0.8615 | 0.7143 | 0.9400 | 1.0973 | 1.4173 | 1.5581 | 1.0931 |
| Gorai.007G347300 | 0.0000 | 0.0492 | 0.0043 | -0.4202 | -0.0757 | 0.5378 | 1.9507 | 0.6702 | 0.0864 | 0.4814 |
| Gorai.007G347400 | 0.0000 | 0.0000 | 0.0000 | 0.0000 | 0.0000 | 0.0000 | 0.0000 | 0.0000 | 0.0000 | 0.0000 |
| Gorai.007G347500 | 0.0000 | 0.0000 | -0.1367 | -2.0000 | 0.0000 | 0.0000 | 1.2658 | 1.3056 | 0.0000 | 0.0000 |
| Gorai.007G347600 | 2.2837 | 1.8425 | 2.1075 | 2.0359 | 2.1154 | 1.8891 | 1.6265 | 1.6603 | 1.6125 | 1.6404 |
| Gorai.007G347700 | -1.0000 | -1.0458 | -1.3979 | -0.6990 | 0.0000 | -0.3010 | 0.0000 | -1.0969 | -0.8239 | 0.0000 |
| Gorai.007G347800 | -1.0969 | -0.7959 | -0.6576 | -0.9208 | -1.5229 | -1.6990 | 0.5798 | -0.4949 | -0.2218 | 0.0000 |
| Gorai.007G355900 | 0.0000 | 0.0000 | 0.0000 | -0.4559 | 0.0000 | 0.0000 | 0.0000 | 0.0000 | 0.0000 | 0.0000 |
| Gorai.007G356000 | -0.4559 | -0.6021 | 0.1335 | 0.0043 | 0.1430 | 0.1959 | 0.4014 | 0.4346 | 0.4624 | 0.3284 |
| Gorai.007G357000 | 0.0000 | 0.0000 | 0.0000 | 0.0000 | 0.0000 | 0.0000 | 0.0000 | 0.0000 | 0.0000 | 0.0000 |
| Gorai.007G357100 | 0.0000 | 0.0000 | 0.0000 | -0.2441 | -0.4559 | 0.3997 | 0.8451 | 1.0788 | -0.2757 | 0.0000 |
| Gorai.007G357200 | 0.0000 | 0.0000 | 0.0000 | 0.1239 | 0.0000 | 0.0000 | -0.6576 | 0.0000 | 0.0000 | 0.0000 |
| Gorai.007G357300 | 0.9196 | 0.9138 | 0.7126 | 0.7218 | 0.9987 | 0.8513 | 1.1082 | 1.2159 | 1.2867 | 1.0461 |
| Gorai.007G359900 | 1.2388 | 0.9405 | 0.6580 | 0.4579 | 0.9253 | 0.6294 | 0.5658 | 0.7076 | -0.3279 | 0.9499 |
| Gorai.007G360000 | -0.2291 | 0.6484 | -0.5376 | 0.3365 | -0.7696 | -0.3872 | 0.0000 | -1.1549 | -0.8861 | 0.0000 |
| Gorai.007G360100 | 0.3075 | 0.3010 | 0.3979 | 0.6646 | 0.6107 | 1.3036 | 0.4116 | 0.6160 | 0.3962 | 0.4786 |
| Gorai.007G360200 | -0.4437 | -0.2676 | -0.6990 | 0.0000 | 0.0000 | -0.7959 | -0.3372 | -0.1079 | -0.2676 | -0.2441 |
| Gorai.007G360300 | 0.0000 | 0.0000 | -0.9208 | -0.6383 | 0.0414 | -0.4815 | -0.3768 | 0.0000 | 0.0000 | 0.0000 |
| Gorai.007G360400 | 0.0000 | 0.0000 | 0.0000 | 0.0000 | -0.9208 | 0.0000 | 0.0000 | -1.0000 | -0.7447 | 0.0000 |
| Gorai.007G360500 | -0.4559 | 0.6160 | -1.5229 | 0.3304 | -0.6778 | -1.6990 | -1.0969 | -0.7212 | -0.8539 | -0.6778 |
| Gorai.007G362300 | 0.2742 | -0.1024 | -0.3279 | 0.0000 | 0.0000 | 0.0000 | 2.3475 | 2.2597 | 0.0000 | 0.0000 |
| Gorai.007G362400 | 1.3353 | 1.2521 | 1.2591 | 1.1694 | 1.2159 | 1.1632 | 1.4128 | 1.4809 | 0.0000 | 0.0000 |
| Gorai.007G362500 | 0.0000 | 0.5694 | -1.0969 | 0.0000 | -0.9208 | -0.5686 | 0.0000 | 0.0000 | 0.0000 | 0.0000 |
| Gorai.007G362600 | 0.0000 | 2.0871 | 0.0000 | 0.5502 | -0.6990 | -0.3872 | -0.3468 | 0.0000 | 0.0000 | 0.0000 |
| Gorai.007G362700 | 0.0000 | -1.5229 | 0.0000 | -0.8861 | -0.2147 | 0.2148 | 0.9600 | 1.1647 | 0.0000 | 0.0000 |
| Gorai.007G363400 | 0.7007 | 0.0000 | 0.8156 | 0.4928 | 0.0000 | -1.0969 | 0.0414 | -1.3010 | -1.0000 | 0.0000 |
| Gorai.007G363500 | 0.0000 | 0.0000 | 0.0000 | -1.0969 | 0.0000 | 0.0000 | 0.0000 | 0.0000 | 0.0000 | 0.0000 |
| Gorai.007G363600 | 0.0000 | 0.0000 | 0.0000 | 0.0000 | 0.0000 | 0.0000 | 0.0000 | 0.0000 | 0.0000 | 0.0000 |
| Gorai.007G364800 | 0.4249 | 0.3444 | -0.3372 | 0.0374 | 0.0000 | 0.0000 | 0.3201 | 0.0000 | 0.0000 | 0.0000 |
| Gorai.007G364900 | 0.0000 | 0.0000 | -2.0000 | 0.0000 | 0.0000 | 0.0000 | -1.1549 | -1.6990 | -1.5229 | 0.0000 |
| Gorai.007G365000 | 0.0000 | 0.0000 | -1.5229 | 0.0000 | 0.0000 | 0.0000 | -2.0000 | 0.0000 | 0.0000 | 0.0000 |
| Gorai.007G365100 | 0.0000 | 0.0000 | 0.0000 | 0.0000 | 0.0000 | 0.0000 | 0.0000 | 0.0000 | 0.0000 | 0.0000 |
| Gorai.007G369400 | 0.0000 | 0.0000 | 0.0000 | 0.0000 | 0.0000 | 0.0000 | 0.0000 | 0.0000 | 0.0000 | 0.0000 |
| Gorai.007G369500 | 0.0000 | 0.0000 | 0.0000 | 0.0000 | 0.0000 | 0.0000 | -0.7959 | 0.0000 | 0.0000 | 0.0000 |
| Gorai.007G369600 | 0.4654 | 0.1303 | -1.3010 | 0.0000 | -1.3979 | -1.0000 | 0.0492 | -0.5086 | -0.7447 | -0.4089 |
| Gorai.007G369700 | 0.6990 | 0.3424 | 0.7024 | 0.6785 | 0.7482 | 0.6767 | 0.7716 | 0.3747 | 0.0682 | 0.5065 |
| Gorai.007G369900 | 0.0000 | 0.0000 | 0.0000 | -1.0969 | 0.0000 | 0.0000 | 0.7076 | 0.0000 | 0.0000 | 0.0000 |
| Gorai.007G370000 | 0.0000 | -2.0000 | 0.0000 | 0.0000 | 0.0000 | 0.0000 | 1.6462 | -1.3979 | 0.0000 | 0.0000 |
| Gorai.007G370100 | 0.0000 | 0.0000 | 0.0000 | 0.0000 | 0.0000 | 0.0000 | 0.0000 | 0.0000 | 0.0000 | 0.0000 |
| Gorai.007G370200 | 0.5752 | 0.2601 | -0.1739 | -0.6383 | -0.2007 | -0.5376 | 0.5353 | -0.4949 | -0.2218 | 0.0000 |
| Gorai.007G370600 | 0.0334 | -0.1192 | -0.1805 | 0.3243 | -0.0315 | 0.3802 | 0.0000 | 0.0000 | 0.0000 | 0.0000 |
| Gorai.007G370700 | 0.1072 | -0.1739 | 0.1038 | 0.2945 | 0.0086 | 0.3945 | 0.3711 | 0.6042 | 0.7993 | 0.0645 |
| Gorai.007G370800 | 0.0000 | 0.0000 | 0.0000 | 0.0000 | 0.0000 | 0.0000 | 0.0000 | 0.0000 | 0.0000 | 0.0000 |
| Gorai.007G370900 | 0.4997 | 0.6503 | 0.7657 | 0.5185 | 0.2279 | -0.3279 | 0.7340 | 0.6749 | 0.6893 | 0.5877 |
| Gorai.007G371000 | -0.5376 | 0.0000 | 0.0000 | -0.2596 | -1.1549 | -0.6990 | 0.3096 | 0.4857 | -0.2007 | 0.0000 |
| Gorai.007G373800 | 0.1931 | 0.8814 | -0.1871 | 0.4249 | 0.4669 | 0.1239 | 1.0406 | 1.6241 | 1.4409 | 1.6983 |
| Gorai.007G373900 | 0.0000 | 0.0000 | 0.0000 | -0.1487 | 0.0000 | 0.0000 | 0.0000 | 0.0000 | 0.0000 | 0.0000 |
| Gorai.007G374000 | 1.2151 | 0.6263 | 0.9385 | 0.3541 | 0.8267 | 0.7903 | 0.7536 | 1.4160 | 1.5792 | 1.0195 |
| Gorai.007G374100 | 1.5882 | 2.9574 | 0.7427 | -0.3665 | -1.3979 | 0.0000 | 1.5163 | -0.2441 | -0.8239 | -0.0458 |
| Gorai.007G374200 | -0.5528 | 0.0000 | -0.0269 | -0.3372 | -0.2218 | -0.8861 | -0.8861 | -0.1805 | -0.5686 | -0.0269 |
| Gorai.007G374300 | 0.7135 | 0.0000 | 0.5599 | 0.8021 | 0.6484 | 1.0282 | 0.5403 | 0.7574 | 0.7348 | 0.7118 |
| Gorai.007G374800 | 0.9009 | 1.2060 | 0.7868 | 1.1235 | 1.0835 | 1.4333 | 1.4357 | 1.1708 | 1.1109 | 1.1602 |
| Gorai.007G374900 | 0.0000 | 2.5976 | 0.0000 | 0.0645 | -0.1308 | 0.5966 | 0.3324 | 0.0000 | 0.0000 | 0.0000 |
| Gorai.007G375000 | 0.0000 | -0.1549 | -1.3010 | 0.0000 | -1.6990 | 0.0000 | 0.2672 | -0.6021 | -0.3372 | 0.0000 |
| Gorai.007G375100 | 1.1884 | 0.0569 | 1.2962 | 0.1072 | -0.4318 | 0.0000 | 0.4031 | 1.2159 | 1.4859 | 0.0000 |
| Gorai.007G375200 | 0.9614 | 0.5966 | 1.2076 | 1.2087 | 1.0500 | 1.0060 | 1.9864 | 1.0149 | 0.9096 | 0.3617 |
| Gorai.007G375300 | 1.3030 | 1.2014 | 1.2125 | 1.2322 | 1.2227 | 1.5149 | 1.6448 | 1.4606 | 1.4967 | 1.3454 |
| Gorai.007G375400 | 0.5944 | 0.7774 | 0.8222 | 0.7380 | 0.6599 | 0.5340 | 0.6821 | 0.3075 | 0.0334 | 0.4265 |
| Gorai.007G377000 | 0.0000 | 0.0000 | -0.7447 | -0.1805 | -0.6576 | -0.6990 | -1.2218 | 0.7924 | -0.1612 | 1.0302 |
| Gorai.007G377100 | 0.0212 | -0.4202 | 0.2430 | -0.1308 | -0.7696 | -1.1549 | 0.3010 | 0.3385 | -0.4318 | 0.5623 |
| Gorai.007G377200 | 0.0000 | 0.0000 | 0.0000 | 0.0000 | 0.0000 | 0.0000 | -0.5376 | 0.0899 | 0.0000 | 0.0000 |
| Gorai.007G377300 | 0.2529 | 1.1720 | 0.3444 | 1.3995 | 1.2167 | 1.4796 | 1.7362 | 1.6620 | -0.1675 | 1.7597 |
| Gorai.007G377400 | -0.5528 | 0.2430 | 0.1072 | 0.8567 | 1.2225 | 1.1647 | 0.4362 | 1.1590 | 0.4564 | 1.3749 |
| Gorai.007G377500 | 0.0170 | 0.6253 | 0.4330 | 0.8344 | 0.8156 | 0.8021 | 0.0569 | 1.3101 | 0.7218 | 1.1427 |
| Gorai.007G377600 | 0.7619 | 1.0422 | 0.9425 | 1.0378 | 0.8395 | 0.3802 | 0.4232 | 0.8109 | 0.9009 | 0.6064 |
| Gorai.007G377700 | -0.7959 | 2.4577 | 0.2923 | 1.5398 | 0.1303 | -0.6198 | -0.3979 | -0.4949 | -0.2291 | 0.0000 |
| Gorai.007G377800 | -0.3188 | -0.3565 | -1.5229 | -0.4559 | -0.0223 | -1.1549 | 2.4805 | 0.2718 | -1.2218 | 0.5276 |
| Gorai.007G377900 | 0.0000 | 0.0000 | 0.0000 | -1.0969 | -1.6990 | 0.0000 | 0.0000 | 0.0000 | 0.0000 | 0.0000 |
| Gorai.007G378000 | 0.4031 | 0.6031 | 0.3979 | 0.4378 | 0.4698 | 0.7050 | 1.1909 | 1.2662 | 1.1424 | 0.8142 |
| Gorai.007G378100 | 0.3945 | 0.1106 | 0.1875 | 0.7427 | 0.5079 | 0.6464 | 0.5211 | 0.7672 | 0.7324 | 0.7348 |
| Gorai.007G378200 | -0.5850 | 1.5126 | -0.5686 | 0.2788 | -1.6990 | -1.3979 | 1.5285 | -0.0809 | -0.3279 | 0.0253 |
| Gorai.007G378300 | 0.8312 | 0.5465 | 0.6884 | 0.5024 | 0.2279 | 0.0969 | 1.3861 | 1.3387 | 1.3464 | 0.6730 |
| Gorai.007G378400 | 0.3139 | -0.1487 | 0.5775 | -0.1024 | -1.3979 | -0.1367 | 0.2504 | 0.7419 | 0.8960 | 0.3766 |
| Gorai.007G378500 | 0.7226 | 1.0426 | 0.9036 | 1.0515 | 1.0599 | 1.7321 | 0.9420 | 1.0095 | 0.8585 | 1.0671 |
| Gorai.008G042500 | 1.0980 | 1.0711 | 1.3908 | 1.0955 | 1.3172 | 1.2560 | 1.1861 | 1.0704 | 1.1661 | 0.8543 |
| Gorai.008G042600 | 0.6561 | 0.4900 | 0.4564 | 0.3560 | 0.3284 | 0.4669 | 0.2695 | 0.0086 | -0.0132 | -0.0315 |
| Gorai.008G042700 | 1.2109 | 1.0906 | 1.4302 | 1.2817 | 1.2605 | 1.5275 | 1.7462 | 1.4273 | 1.3064 | 1.4404 |
| Gorai.008G042800 | 1.4619 | 1.3998 | 1.3881 | 1.0170 | 0.7520 | 0.7882 | 2.1861 | 1.2507 | 1.3361 | 1.0550 |
| Gorai.008G042900 | 1.3122 | 1.3831 | 1.5364 | 1.4685 | 1.5026 | 1.4908 | 1.4579 | 1.4533 | 1.3943 | 1.4065 |
| Gorai.008G043000 | 0.6998 | 1.1212 | 0.7868 | 0.7451 | 0.4624 | 0.5065 | 1.3233 | 1.2375 | 0.9614 | 1.3572 |
| Gorai.008G048200 | 0.4409 | 0.1492 | 0.3909 | 0.4757 | -0.0862 | 0.0569 | 0.7860 | 0.6561 | -1.0000 | 0.0000 |
| Gorai.008G048300 | 1.2953 | 1.2378 | 1.4115 | 1.2403 | 1.2858 | 1.0149 | 1.7106 | 0.7597 | 0.3598 | 0.9196 |
| Gorai.008G048400 | 0.1732 | -0.2007 | 0.0086 | 0.2253 | -0.4202 | -0.7447 | 1.9011 | 1.2674 | -0.1249 | 0.0000 |
| Gorai.008G048500 | 0.0000 | 0.0000 | 0.0000 | 0.0000 | 0.0000 | 0.0000 | 0.0000 | -0.7959 | -0.5229 | 0.0000 |
| Gorai.008G048600 | 0.2279 | 0.0607 | -0.0132 | -1.1549 | -1.0000 | -1.0458 | -0.5850 | -0.1675 | -0.1549 | -0.2596 |
| Gorai.008G049500 | 0.0000 | 0.0000 | 0.0000 | 0.0000 | -2.0000 | 0.0000 | 0.0000 | 0.0000 | 0.0000 | 0.0000 |
| Gorai.008G049600 | 0.0934 | -0.3565 | -0.2757 | 0.0792 | 1.2198 | 0.7267 | 1.2188 | 1.6262 | 1.5457 | 1.6215 |
| Gorai.008G049700 | 0.5185 | 1.7282 | 0.5705 | 0.7738 | 1.1402 | 0.9841 | 1.0048 | 0.8633 | 0.7466 | 0.8865 |
| Gorai.008G050900 | 1.0512 | 0.7050 | 0.5119 | 0.5172 | -0.2218 | 0.2430 | 1.7634 | 1.7524 | 0.0000 | 0.0000 |
| Gorai.008G051000 | 0.4425 | 0.0000 | 0.1847 | -0.0605 | 0.1761 | 0.4698 | 0.7110 | 0.9430 | 0.5490 | 1.0931 |
| Gorai.008G051100 | 0.0000 | -0.8861 | -1.0969 | -0.8861 | -0.7447 | -1.1549 | 0.5821 | -0.5528 | -0.9586 | -0.3979 |
| Gorai.008G051200 | 0.0000 | 0.0000 | -1.0458 | -1.3010 | -0.8539 | -0.5850 | 0.9717 | -0.0655 | -0.3979 | 0.0719 |
| Gorai.008G051300 | -0.4949 | -0.4318 | -0.3768 | -0.2518 | -0.3565 | 0.0086 | 0.5211 | -0.0362 | -0.0410 | -0.0969 |
| Gorai.008G051400 | 0.5465 | 0.8943 | 0.8363 | 0.8506 | 0.6981 | 0.8993 | 0.4014 | 0.8028 | 0.9258 | 0.5263 |
| Gorai.008G051900 | 1.8953 | 2.0686 | 2.0811 | 1.9666 | 1.7028 | 1.7392 | 1.7475 | 1.6232 | 1.5606 | 1.6164 |
| Gorai.008G052000 | 0.0000 | 0.0000 | 0.0000 | 0.0000 | 0.0000 | 0.0000 | 0.0000 | -2.0000 | -1.6990 | 0.0000 |
| Gorai.008G052100 | 0.8048 | 0.9886 | 0.8007 | 0.3874 | 0.2201 | 0.6712 | 0.7076 | 0.7604 | 0.7924 | 0.6493 |
| Gorai.008G055000 | -1.6990 | 1.0402 | 0.5079 | 0.7931 | 0.9956 | 1.4350 | 1.3058 | 1.1855 | 0.7642 | 1.0715 |
| Gorai.008G055100 | -0.4437 | -0.1612 | -0.4685 | -0.0605 | -0.4437 | -0.0969 | 0.0000 | 0.1271 | 0.3010 | -0.3188 |
| Gorai.008G055200 | -0.0757 | 0.6937 | 0.4669 | 0.5966 | 0.5441 | 0.8028 | 1.2953 | 1.0004 | 0.0000 | 0.0000 |
| Gorai.008G062200 | 0.0000 | 0.0000 | 0.0000 | 0.0000 | 0.0000 | 0.0000 | 1.4897 | 1.7185 | 0.0000 | 0.0000 |
| Gorai.008G062300 | 0.0000 | 0.2967 | 0.3598 | 0.6990 | 0.7042 | 0.5211 | 0.8109 | 0.4298 | 0.6758 | -0.5850 |
| Gorai.008G064900 | 0.0000 | 0.0000 | -0.6990 | -0.4437 | -0.3279 | -0.2291 | -0.3665 | 0.3522 | -0.1487 | 0.0000 |
| Gorai.008G069700 | 0.1959 | -0.0555 | -1.0458 | 0.0253 | -0.8539 | 0.2455 | 0.6821 | 0.4814 | 0.0000 | 0.0000 |
| Gorai.008G069800 | 1.1926 | 1.1824 | 1.1086 | 1.1421 | 1.2956 | 1.0881 | 1.3576 | 1.5773 | 1.6038 | 1.4747 |
| Gorai.008G069900 | 1.2413 | 0.9805 | 1.0107 | 0.8954 | 1.0438 | 0.8519 | 1.0580 | 1.1239 | 0.0969 | 0.3945 |
| Gorai.008G074700 | 0.0000 | 0.0000 | 0.0000 | 0.0000 | 0.0000 | 0.0000 | 0.0000 | 0.0000 | 0.0000 | 0.0000 |
| Gorai.008G077500 | 0.5453 | 1.2844 | 0.7466 | 1.1433 | 0.6335 | 0.8808 | 1.1045 | 0.3483 | 0.0492 | 0.0000 |
| Gorai.008G077600 | 0.6304 | 1.6142 | 0.8500 | 0.8887 | 0.8751 | 0.6064 | 1.1553 | 0.2553 | 0.4683 | -0.3979 |
| Gorai.008G077700 | 0.5694 | 0.5832 | 0.6599 | 0.4997 | 0.8000 | 0.6031 | 0.6415 | 0.3464 | 0.0531 | 0.0128 |
| Gorai.008G077800 | 0.7875 | 0.6812 | 0.5185 | 0.6232 | 0.3304 | 0.4014 | 1.5064 | 0.6222 | 0.0755 | 0.0000 |
| Gorai.008G078200 | 1.3438 | 1.1741 | 1.3353 | 1.6292 | 1.1878 | 1.5145 | 1.1976 | 0.2648 | 0.5353 | 0.0000 |
| Gorai.008G079500 | 0.1644 | 0.0334 | 0.0899 | 0.0792 | -0.0132 | -0.0315 | 0.8055 | 0.6345 | 0.6580 | 0.5353 |
| Gorai.008G080800 | 0.9360 | 1.2074 | 1.4091 | 1.4728 | 1.7605 | 1.2874 | -0.1805 | 1.3483 | 0.9299 | 1.5056 |
| Gorai.008G080900 | -0.0315 | -0.2366 | 0.1959 | 0.1038 | 0.2201 | 0.0043 | -0.0410 | 0.3997 | 0.2455 | 0.4609 |
| Gorai.008G081000 | 0.0000 | 0.0000 | 0.0000 | 0.0000 | 0.0000 | 0.0000 | 0.0000 | 0.0000 | 0.0000 | 0.0000 |
| Gorai.008G081100 | 1.6608 | 2.0938 | 2.2056 | 1.9564 | 1.6459 | 1.3218 | 0.3927 | 1.6951 | 0.9509 | 1.9157 |
| Gorai.008G081200 | 0.5250 | 0.4698 | -0.1192 | -0.1487 | 1.3727 | 3.1768 | 0.0000 | 0.0000 | 0.0000 | 0.0000 |
| Gorai.008G094200 | 0.0000 | 1.0774 | 0.0000 | 0.0000 | 0.0000 | 0.0000 | -0.6021 | 0.2648 | 0.0000 | 0.5289 |
| Gorai.008G094300 | 0.8293 | 1.4193 | 1.2718 | 0.9805 | 1.2248 | 0.9671 | 1.2801 | 1.2905 | 1.3017 | 1.2074 |
| Gorai.008G096000 | 0.0000 | 0.0000 | -0.5850 | -2.0000 | -0.3010 | 0.0000 | 0.8306 | 0.9694 | -0.4815 | 0.0000 |
| Gorai.008G106200 | 1.6116 | 1.5700 | 1.9125 | 1.8385 | 2.0983 | 2.2683 | 1.8927 | 0.8681 | 0.6375 | 0.9675 |
| Gorai.008G106300 | 0.0000 | 0.0000 | 0.0000 | 0.0000 | 0.0000 | 0.0000 | 0.0000 | 0.0000 | 0.0000 | 0.0000 |
| Gorai.008G106400 | 0.4330 | 0.6031 | -0.6021 | 0.1399 | 0.2625 | 0.8162 | 1.2052 | 0.8733 | 0.0000 | 0.0000 |
| Gorai.008G109400 | 0.0000 | 0.0000 | -0.6778 | 0.2480 | -0.4949 | 0.0000 | 2.3972 | 2.4964 | 1.0233 | 0.5635 |
| Gorai.008G109500 | 0.7101 | 0.7443 | 0.8549 | 0.9823 | 1.0945 | 1.4444 | 1.2570 | 0.9581 | 0.8344 | 0.9983 |
| Gorai.008G109600 | -0.5850 | 0.0000 | -1.3010 | -0.9208 | -0.5686 | -1.0969 | -0.8539 | -1.0000 | -0.7212 | 0.0000 |
| Gorai.008G115000 | -0.1135 | 0.0000 | -0.0044 | -1.3010 | -1.1549 | -0.9586 | -0.1135 | 0.4116 | 0.5539 | -0.1487 |
| Gorai.008G115100 | 0.9643 | 0.8215 | 0.9983 | 1.1196 | 1.2253 | 1.1970 | 1.2284 | 1.3512 | 0.9410 | 1.2279 |
| Gorai.008G115200 | -0.1367 | -0.8861 | -0.4318 | -0.5376 | -0.4949 | -1.1549 | 1.7229 | 0.2279 | -0.6383 | 0.2095 |
| Gorai.008G122900 | 1.0888 | 1.5420 | 1.2792 | 1.2925 | 1.1587 | 1.1833 | 1.1278 | 1.4849 | 1.3725 | 1.5171 |
| Gorai.008G123000 | 0.3160 | 0.5786 | -0.4089 | -0.6778 | -0.7212 | -0.4559 | -0.4089 | 0.0253 | 0.0934 | -0.1367 |
| Gorai.008G123100 | -0.3372 | -0.6576 | -0.5229 | -0.3188 | -0.0915 | 0.3729 | 0.6263 | 0.2330 | 0.4456 | -0.3979 |
| Gorai.008G123200 | 0.0000 | 0.1931 | 0.3054 | -1.1549 | 0.6294 | 0.3560 | 0.2601 | 1.2751 | 0.0000 | 0.0000 |
| Gorai.008G128100 | 0.3284 | 0.6138 | 0.4564 | 0.8162 | 1.6002 | 1.5879 | 1.5809 | 0.2405 | 0.3284 | 0.0414 |
| Gorai.008G128200 | 1.2620 | 1.0618 | 1.2964 | 1.3760 | 1.2340 | 1.5660 | 1.4004 | 1.3193 | 1.4648 | 0.9180 |
| Gorai.008G129100 | 1.0418 | 1.0656 | 1.2269 | 0.9624 | 1.2022 | 1.6549 | 1.7168 | 1.7740 | 1.4160 | 1.8148 |
| Gorai.008G132300 | 0.0000 | 0.0000 | 0.0000 | -0.6576 | 0.0000 | 0.0000 | 0.0000 | 0.0000 | 0.0000 | 0.0000 |
| Gorai.008G132400 | 0.0000 | -0.4815 | -0.2366 | -0.6990 | -0.7447 | 0.0000 | -0.0362 | 0.3522 | 0.0253 | 0.4914 |
| Gorai.008G135500 | 0.0000 | -1.3979 | 0.0000 | 0.0000 | 0.0000 | 0.0000 | 0.8555 | 1.1517 | -0.1427 | 0.1004 |
| Gorai.008G135600 | 0.6263 | 0.7016 | 1.0678 | 0.9850 | 0.9841 | 0.6637 | 0.6946 | 0.7251 | 0.7551 | 0.6180 |
| Gorai.008G135700 | 0.8363 | 1.1889 | 1.0216 | 1.1855 | 1.3312 | 1.2235 | 1.2758 | 1.3369 | 1.1119 | 1.4299 |
| Gorai.008G135800 | -0.2596 | 0.3139 | 0.6031 | 1.3692 | 0.2529 | 0.4425 | -0.7447 | 1.4755 | 1.3621 | -0.0269 |
| Gorai.008G151500 | 0.7505 | 0.0000 | 0.4393 | 0.9562 | 0.5172 | 0.1399 | 0.3784 | 0.3892 | 0.5635 | -2.0000 |
| Gorai.008G151600 | 0.0000 | 0.0000 | -0.1427 | -0.1192 | 0.7050 | 0.3424 | 0.5024 | 0.0253 | 0.1335 | -0.4559 |
| Gorai.008G151700 | 0.0000 | 0.0719 | -1.5229 | -1.6990 | 0.0000 | 0.0000 | 1.3122 | -0.9208 | 0.0000 | -0.8539 |
| Gorai.008G153300 | 0.0000 | 0.0000 | -0.3872 | 0.0000 | -0.0655 | -1.3010 | 0.1987 | -0.4559 | -1.5229 | 0.0000 |
| Gorai.008G153400 | 1.1017 | 0.9547 | 1.2594 | 1.1278 | 1.0993 | 1.2159 | 1.3308 | 1.5853 | 1.5049 | 1.5935 |
| Gorai.008G153500 | 0.0000 | 0.0000 | 0.0000 | -0.6576 | -0.5086 | -1.6990 | -1.1549 | -0.6383 | -0.3665 | 0.0000 |
| Gorai.008G155600 | 0.0000 | 0.0000 | 0.0000 | 0.0000 | 0.0000 | 0.0000 | 0.0000 | 0.0000 | 0.0000 | 0.0000 |
| Gorai.008G155700 | 0.0000 | -0.5850 | 0.0607 | 0.8848 | 0.6749 | 0.7959 | 1.9509 | 1.1992 | -0.0757 | 1.4513 |
| Gorai.008G156200 | 0.0000 | -0.7447 | -0.9208 | -0.4559 | -0.0655 | -0.9586 | -0.4318 | -1.2218 | -0.9586 | 0.0000 |
| Gorai.008G156300 | -0.2518 | -0.3372 | -0.4559 | 0.0000 | -0.2840 | -0.1938 | -0.5850 | -0.1487 | 0.0170 | -0.5528 |
| Gorai.008G156400 | 0.2967 | 0.6928 | 0.6542 | 1.2914 | 1.2794 | 0.8195 | 1.0993 | 1.2792 | 1.3545 | 1.1021 |
| Gorai.008G156500 | 1.0938 | 1.5723 | 1.3103 | 1.3412 | 1.4270 | 1.3920 | 1.0374 | 1.5065 | 1.3101 | 1.5899 |
| Gorai.008G157600 | 0.0000 | -0.1675 | -0.9208 | -0.9208 | -0.3665 | -0.2924 | 0.6096 | 0.6021 | 0.0000 | 0.0000 |
| Gorai.008G157700 | 0.0000 | 0.0000 | 0.0000 | -1.0969 | -1.1549 | 0.0000 | 0.0000 | 0.0000 | 0.0000 | 0.0000 |
| Gorai.008G191300 | 0.9400 | -0.4089 | 1.1489 | 0.8785 | 1.0233 | 0.7966 | 1.6014 | 1.4625 | 1.3094 | 1.3501 |
| Gorai.008G191400 | 0.0000 | 0.5250 | 0.0253 | -0.3468 | -0.7212 | -0.8539 | 0.0000 | 0.0000 | 0.0000 | 0.0000 |
| Gorai.008G191500 | 1.5075 | 1.6805 | 1.0945 | 0.9058 | 1.1055 | 0.9768 | 1.2092 | 1.2813 | 1.2749 | 1.2191 |
| Gorai.008G191600 | 0.0000 | 0.0000 | -0.5686 | 0.0000 | 0.0000 | 0.0000 | 1.4943 | 1.4074 | 0.0000 | 0.0000 |
| Gorai.008G191700 | 0.0000 | 0.0000 | -0.1487 | 0.0000 | -1.0000 | -0.4202 | 0.8871 | 0.8482 | -0.1249 | 0.5092 |
| Gorai.008G192000 | 0.0000 | 0.0000 | -0.3665 | 0.0000 | 0.0000 | 0.0000 | 0.0000 | 0.0000 | 0.0000 | 0.0000 |
| Gorai.008G192100 | 1.5119 | 1.7060 | 1.3043 | 1.4195 | 1.8918 | 2.0406 | 0.9248 | 1.3274 | 1.3520 | 1.2271 |
| Gorai.008G192200 | 0.0000 | 0.0000 | 0.0000 | 0.0000 | 0.0000 | 0.0000 | 0.0000 | 0.0000 | 0.0000 | 0.0000 |
| Gorai.008G192300 | 0.3541 | 0.2253 | 0.6180 | 0.3324 | -0.0088 | -0.1135 | 1.6053 | 0.9841 | -0.3665 | 0.0000 |
| Gorai.008G192400 | 0.6191 | -0.2676 | 0.3874 | -0.6990 | -0.4949 | -0.6778 | 0.1271 | 0.7672 | 0.8209 | 0.6263 |
| Gorai.008G209500 | 1.4535 | 0.7042 | 0.3464 | 0.8998 | -2.0000 | 0.3160 | 1.6751 | 1.5556 | 0.0000 | 0.0000 |
| Gorai.008G209600 | 0.9494 | 1.0145 | 1.1987 | 1.0484 | 0.8116 | 1.0212 | 1.5707 | 1.4844 | 1.1844 | 1.6130 |
| Gorai.008G209700 | -0.4949 | 0.4200 | -0.0044 | -0.2291 | 0.2405 | 0.1761 | 0.3054 | 0.9939 | -0.2218 | 0.2227 |
| Gorai.008G209800 | 0.5011 | 0.7528 | 0.9133 | 0.7259 | 0.8585 | 0.5441 | 0.8698 | 0.5237 | 0.0000 | 0.6085 |
| Gorai.008G214000 | 0.0453 | -0.0410 | -0.8861 | 0.8500 | 1.8459 | 1.4299 | 1.8618 | 1.0418 | 0.7513 | 1.1667 |
| Gorai.008G214100 | 1.0538 | 0.9791 | 1.0671 | 1.1844 | 1.0920 | 1.1801 | 0.8756 | 0.8344 | 0.5999 | 0.5798 |
| Gorai.008G214200 | 0.0000 | 0.0000 | 0.0000 | 0.0000 | 0.0000 | 0.0000 | 0.0000 | 0.0000 | 0.0000 | 0.0000 |
| Gorai.008G214300 | 0.7443 | 1.1048 | 0.9263 | 0.9633 | 0.9978 | 0.6571 | 0.9494 | 1.2320 | 1.1723 | 0.8287 |
| Gorai.008G222400 | -0.8239 | -0.2840 | -0.1805 | 0.0719 | 1.0813 | 1.2613 | 2.1810 | 0.4914 | -0.2366 | 0.7110 |
| Gorai.008G222500 | 1.3649 | 0.1106 | 1.7758 | 1.1987 | -0.4559 | -0.3279 | 0.7559 | 1.4436 | 1.5586 | 1.1861 |
| Gorai.008G222600 | 1.3795 | 2.0451 | 1.0473 | 1.3300 | 1.2450 | 1.0306 | 0.3222 | 1.1235 | 1.0000 | 1.1632 |
| Gorai.008G222700 | -1.3010 | 0.0000 | -1.5229 | -0.7959 | -0.7212 | -0.7959 | -0.5086 | 0.2810 | 0.5502 | 0.0000 |
| Gorai.008G222800 | 0.1303 | -0.4089 | 0.6646 | 0.5855 | 0.5302 | 0.6117 | 0.5353 | 0.4579 | 0.3802 | 0.4183 |
| Gorai.008G247600 | 0.7796 | 0.0000 | 0.6435 | 0.0043 | -1.6990 | -1.1549 | 1.2395 | 0.2405 | 0.4314 | -0.2676 |
| Gorai.008G247700 | 0.0000 | 0.0000 | -0.9586 | -0.4949 | 0.0000 | 0.0000 | 0.0000 | 0.0000 | 0.0000 | 0.0000 |
| Gorai.008G247800 | 0.1703 | 0.6314 | 0.1206 | 0.0569 | 0.5809 | 0.4533 | -0.0177 | 1.4592 | 1.7032 | 0.4900 |
| Gorai.008G247900 | 0.1553 | -1.1549 | 0.0682 | -2.0000 | -0.3872 | 0.7839 | 1.0099 | 1.6720 | 1.6689 | 1.2920 |
| Gorai.008G248000 | 1.6412 | 1.5252 | 1.7406 | 1.6765 | 1.6071 | 1.2497 | 1.6466 | 1.8203 | 1.4764 | 1.9488 |
| Gorai.008G248100 | 0.7745 | 0.9294 | 0.8082 | 0.8102 | 0.7910 | 1.0386 | 0.3838 | 0.5977 | 0.3874 | 0.6875 |
| Gorai.008G249000 | 0.0000 | 0.0000 | 0.0000 | 0.0000 | 0.0000 | 0.0000 | 0.0000 | 0.0000 | 0.0000 | 0.0000 |
| Gorai.008G249100 | 0.0000 | 0.0000 | 0.0000 | 0.0000 | 0.0000 | 0.0000 | 0.0000 | 0.0000 | 0.0000 | 0.0000 |
| Gorai.008G249200 | 0.3655 | 0.4800 | 0.3979 | 0.0969 | 0.3010 | 0.2122 | 0.2279 | 0.5729 | 0.6304 | 0.4265 |
| Gorai.008G268600 | 1.3879 | 0.7459 | 1.1405 | 1.3856 | 1.2833 | 1.0107 | 1.2322 | 1.2914 | 1.2243 | 1.0892 |
| Gorai.008G268700 | 0.5729 | 0.5315 | 0.7738 | 0.9325 | 1.2482 | 0.9365 | 0.7649 | 1.4255 | 1.2558 | 1.1746 |
| Gorai.008G268800 | -0.5686 | 0.0000 | -1.0000 | -1.0969 | 0.0000 | 0.0000 | 1.2947 | 1.1870 | -0.6198 | 0.0000 |
| Gorai.008G268900 | 1.3030 | 0.8506 | 1.3017 | 0.5944 | 0.3201 | 0.3365 | 1.5084 | 1.4019 | 0.7924 | 1.6052 |
| Gorai.008G283400 | -0.7959 | 0.0000 | -1.2218 | -1.3979 | -0.9208 | 0.0000 | -0.4089 | -0.1871 | -0.9208 | 0.0334 |
| Gorai.008G283500 | 0.0000 | 0.5786 | -1.6990 | 0.2672 | -0.7959 | -0.0915 | 0.6222 | 0.5682 | -0.0862 | 0.3766 |
| Gorai.008G283600 | -0.1024 | 0.2765 | -0.0862 | 0.2695 | 0.3118 | 0.7497 | 0.2253 | 0.2878 | 0.4914 | -0.3010 |
| Gorai.008G283700 | -0.0269 | 0.0000 | -0.0655 | -0.2840 | -0.1938 | 0.0253 | 0.0000 | 0.0000 | 0.0000 | 0.0000 |
| Gorai.008G283800 | 0.4742 | 0.0170 | 0.5911 | 0.3010 | -0.1487 | 0.0128 | 0.4997 | 0.6884 | 0.5065 | 0.7634 |
| Gorai.008G283900 | 0.6325 | 0.5682 | 0.1732 | 0.0755 | -0.0862 | 0.2695 | 0.4183 | 0.7404 | 0.0000 | 0.0000 |
| Gorai.008G291100 | 1.2697 | 1.1793 | 1.3310 | 1.3760 | 1.0535 | 1.4473 | 1.4695 | 1.4143 | 1.5005 | 1.2170 |
| Gorai.008G291200 | 0.0000 | 0.0000 | 0.0000 | 0.0000 | 0.0000 | 0.0000 | 0.0000 | 0.0000 | 0.0000 | 0.0000 |
| Gorai.008G291300 | 0.4548 | 0.0000 | -0.1805 | -0.7959 | -0.7212 | 0.8488 | -0.0605 | 1.1647 | 0.2923 | 1.3971 |
| Gorai.008G291400 | 0.0000 | -1.5229 | -1.5229 | 0.1106 | 0.7308 | 0.8082 | -1.3979 | -1.2218 | -1.3010 | 0.0000 |
| Gorai.008G291500 | 0.7709 | 0.6532 | 0.8401 | 0.8733 | 0.9722 | 1.3214 | 1.0626 | 0.8351 | 0.7513 | 0.8463 |
| Gorai.008G291600 | 0.0000 | 0.0000 | 0.0000 | -0.4437 | -0.3665 | -0.0458 | 0.0000 | 0.0000 | 0.0000 | 0.0000 |
| Gorai.009G002800 | 0.0000 | 0.3263 | -0.4089 | 0.1847 | 0.4200 | 0.5366 | 1.0496 | -0.1549 | 0.0000 | 0.1072 |
| Gorai.009G002900 | 0.0334 | 0.8506 | 0.5843 | 0.5198 | 0.8932 | 0.7168 | 0.8651 | 0.9138 | 0.7459 | 0.7404 |
| Gorai.009G003000 | -0.0269 | 0.8745 | 0.3284 | 0.6928 | 0.6928 | 0.5237 | 0.3945 | 0.8382 | 0.0000 | -0.0088 |
| Gorai.009G003100 | 1.5824 | 1.2723 | 1.3867 | 1.4527 | 1.3473 | 1.4495 | 1.2781 | 1.3216 | 1.3797 | 1.1430 |
| Gorai.009G003200 | 0.0000 | -0.6383 | -1.2218 | 0.0000 | 0.0000 | -0.9586 | -0.0410 | -0.0555 | 0.0000 | 0.0000 |
| Gorai.009G003300 | 0.4440 | 0.6693 | 0.4346 | 0.5428 | 0.5065 | 0.8228 | 1.1345 | 1.2146 | 0.9138 | 0.8338 |
| Gorai.009G008500 | -0.4685 | 0.3284 | 0.5276 | 0.9562 | 0.2405 | 1.1255 | 0.0212 | 0.8904 | 0.8820 | 0.0000 |
| Gorai.009G008600 | 1.6438 | 1.8472 | 1.5935 | 1.4747 | 1.3243 | 1.2662 | 1.3249 | 1.4224 | 1.4104 | 1.3664 |
| Gorai.009G008700 | 0.0000 | 0.0000 | 0.6128 | 0.6031 | 0.6415 | 0.1847 | 0.5821 | 1.2038 | 0.0000 | -0.5229 |
| Gorai.009G008800 | 0.0000 | 0.0000 | -1.3979 | 0.0000 | -1.3010 | 0.0000 | -0.3372 | 0.0000 | 0.0000 | 0.0000 |
| Gorai.009G008900 | 0.0043 | 0.4843 | -0.6198 | -0.7447 | 0.0682 | 0.2014 | 0.9058 | 1.0111 | 0.0000 | -0.3279 |
| Gorai.009G009000 | -0.1739 | 2.0169 | -0.5850 | -0.3872 | 0.0000 | -0.8861 | 0.7657 | 0.1732 | -0.4437 | 0.3784 |
| Gorai.009G009100 | 0.4116 | 0.5821 | 0.6618 | 0.5866 | 0.6590 | 0.6484 | 0.7033 | 1.2999 | 1.1647 | 1.3477 |
| Gorai.009G010400 | 0.0000 | 0.0000 | -2.0000 | 0.0000 | -1.6990 | 0.0000 | 0.3324 | 0.0000 | 0.0000 | 0.0000 |
| Gorai.009G010500 | 0.0000 | 0.0000 | 0.0000 | -1.2218 | 0.0000 | -1.5229 | 0.8222 | -0.1487 | 0.0000 | 0.1173 |
| Gorai.009G010600 | 0.0000 | 0.0000 | -1.5229 | -0.8239 | 0.0000 | 0.0000 | -1.0969 | 0.0000 | 0.0000 | 0.0000 |
| Gorai.009G010700 | 0.0000 | 0.0000 | -0.8539 | -0.7696 | 0.0000 | -0.4815 | -0.2366 | -0.1871 | 0.0828 | 0.0000 |
| Gorai.009G010800 | 0.8129 | 1.3901 | 1.4555 | 1.7585 | 1.8122 | 1.6921 | 1.3545 | 1.5317 | 1.6380 | 1.2936 |
| Gorai.009G010900 | -1.3010 | 0.3222 | 0.2742 | 0.4200 | 0.7135 | 1.0803 | 0.9079 | 0.7716 | 0.6031 | -1.6990 |
| Gorai.009G011300 | -0.1871 | 0.0253 | 0.0000 | -0.7959 | -0.0088 | -0.2147 | 0.9474 | 1.0426 | 0.9886 | 0.5966 |
| Gorai.009G011400 | 0.0000 | 0.0000 | 0.0000 | 0.0000 | 0.0000 | 0.0000 | 1.4869 | 1.4741 | 0.0000 | 0.0000 |
| Gorai.009G011500 | 0.0000 | 0.3747 | 0.0294 | 0.3010 | 0.1038 | 0.6646 | 0.9085 | 1.0730 | 0.6212 | 0.8762 |
| Gorai.009G011600 | 0.0000 | -0.2147 | 0.0000 | -0.2596 | 0.0000 | -0.3010 | 0.5922 | 0.7168 | 0.0000 | 0.0000 |
| Gorai.009G011700 | 0.0000 | 0.0000 | -1.0458 | 0.0000 | -0.3098 | -0.7212 | -0.9208 | 0.0000 | 0.0000 | 0.0000 |
| Gorai.009G011800 | 0.2095 | 0.4232 | 0.5809 | 0.3979 | 0.5403 | 0.6314 | 0.3766 | 0.4942 | 0.4771 | -0.2366 |
| Gorai.009G013300 | 0.3304 | -0.1487 | -0.0223 | 0.3345 | 0.3927 | -0.0969 | 2.1568 | -0.2924 | -0.5686 | 0.0000 |
| Gorai.009G013400 | 0.4698 | 1.3084 | 0.8075 | 0.6812 | 0.8463 | 0.5250 | 1.4694 | 1.0310 | 0.9450 | 1.0434 |
| Gorai.009G013500 | -0.3279 | -0.3188 | 0.3304 | 0.0334 | 0.6085 | 0.2833 | -0.4318 | 1.1303 | 1.0278 | 1.1556 |
| Gorai.009G013600 | 0.2625 | 1.0162 | 0.6365 | 0.4698 | -0.5850 | 0.1335 | 0.7627 | 0.7202 | 0.2175 | 0.9047 |
| Gorai.009G013700 | 1.6839 | 2.0926 | 1.8999 | 1.9814 | 2.3322 | 2.0421 | 1.7622 | 2.0107 | 1.6499 | 2.1597 |
| Gorai.009G013800 | 1.0022 | 1.1830 | 1.1833 | 0.7259 | 1.1816 | 1.3953 | 0.7604 | 1.1284 | 1.0039 | 0.9996 |
| Gorai.009G013900 | 1.2842 | 0.1875 | 1.9241 | 1.7317 | 2.1458 | 1.7455 | 1.6991 | 1.9813 | 2.0074 | 1.8793 |
| Gorai.009G023100 | 0.0000 | 0.0000 | -0.0044 | -0.1487 | -0.8239 | -0.5086 | 1.9433 | -0.1739 | -0.2007 | 0.0000 |
| Gorai.009G023200 | 1.3895 | 1.1942 | 1.3962 | 1.3713 | 0.9170 | 0.8865 | 1.2408 | 1.3714 | 1.3962 | 1.2711 |
| Gorai.009G023300 | 2.1500 | 1.6902 | 2.1475 | 1.9757 | 2.3578 | 2.2232 | 1.9735 | 1.9974 | 1.8842 | 1.9735 |
| Gorai.009G023400 | 0.3483 | 0.0000 | 1.3257 | 0.2330 | -0.0223 | -0.5686 | -0.1308 | -0.2218 | -0.6383 | 0.0000 |
| Gorai.009G023500 | 0.0000 | -0.1675 | -0.5528 | -1.6990 | -0.6198 | -0.4815 | 0.7767 | 0.7966 | 0.0864 | 0.1761 |
| Gorai.009G023600 | 0.0000 | -2.0000 | -0.2596 | 0.0128 | -0.3468 | 0.0000 | 0.5855 | 0.4713 | 0.0000 | 0.3766 |
| Gorai.009G026300 | 0.3979 | 1.5458 | 0.1847 | 1.2315 | 1.7380 | 1.3640 | 1.3570 | 0.1790 | 0.3243 | -0.1487 |
| Gorai.009G026400 | 0.5378 | 0.7185 | 0.6031 | 0.7505 | 0.7789 | 1.1878 | 0.3502 | 0.6776 | 0.6253 | 0.6609 |
| Gorai.009G026500 | 0.0000 | 0.0000 | 0.0000 | 0.0000 | -0.6198 | 0.3032 | -0.9208 | -0.3468 | -0.6990 | -0.2007 |
| Gorai.009G026600 | 0.4330 | 0.1644 | 0.2504 | 0.5999 | 0.8768 | 0.7987 | 0.8915 | 0.6232 | 0.4249 | 0.6637 |
| Gorai.009G026700 | 0.0000 | 1.5703 | 0.0000 | 0.9899 | -2.0000 | -0.1249 | 0.8439 | 1.9799 | 0.0000 | 0.0000 |
| Gorai.009G030900 | -0.3565 | 0.0000 | -0.6778 | 0.0000 | -1.0969 | 0.0000 | 1.5243 | 0.3075 | 0.0607 | 0.4150 |
| Gorai.009G031000 | 0.6385 | 0.7694 | 1.1149 | 0.8871 | 1.0208 | 1.3391 | 1.2622 | 1.0603 | 0.7649 | 1.1872 |
| Gorai.009G031100 | 0.1072 | 0.6243 | 0.5821 | 0.5490 | 0.7760 | 0.3201 | 0.8457 | 1.0611 | 1.1697 | 0.8182 |
| Gorai.009G031200 | 0.0000 | 0.0000 | 0.0000 | 0.0000 | -1.0458 | 0.0334 | 0.0492 | 0.0682 | 0.2430 | -0.3665 |
| Gorai.009G031300 | 0.4133 | 1.4038 | 0.1553 | 0.4713 | 0.0212 | 0.0828 | 0.9657 | 1.0864 | 0.5599 | 0.0000 |
| Gorai.009G031400 | 0.0000 | -1.3010 | -1.6990 | 0.7796 | 0.6405 | 1.8801 | 1.6012 | -0.9586 | 0.0000 | -0.6778 |
| Gorai.009G031500 | 0.8686 | 0.0043 | 0.7251 | 0.7597 | 0.3997 | 0.2455 | 1.1714 | 1.1411 | 1.0821 | 1.1313 |
| Gorai.009G036600 | -0.3872 | 0.5502 | -0.4202 | -0.2518 | -0.5528 | -0.5850 | -0.8861 | 0.0294 | -0.0706 | 0.0569 |
| Gorai.009G036700 | 0.0000 | 1.8473 | 0.5224 | 0.7007 | 0.9708 | 1.1035 | 0.6767 | 0.6503 | 0.5527 | 0.1206 |
| Gorai.009G036800 | 0.0000 | -2.0000 | 0.0000 | 0.0000 | 0.0000 | 0.0000 | 0.9430 | 1.5831 | 0.0000 | 0.0000 |
| Gorai.009G036900 | 0.0000 | 0.0000 | -1.1549 | 0.0000 | 0.0000 | -0.8239 | -0.3010 | 0.0000 | 0.0000 | 0.0000 |
| Gorai.009G037000 | 0.6955 | -0.2676 | 0.5366 | 0.5441 | 1.9053 | 2.5173 | 0.1523 | 1.1367 | 1.2813 | 0.8021 |
| Gorai.009G037100 | -0.6990 | -0.0044 | -0.3665 | -0.1024 | -0.6383 | -0.5686 | 0.9805 | 0.7760 | 0.5159 | 0.7168 |
| Gorai.009G040600 | 1.0370 | 2.1539 | 1.3934 | 1.3304 | 1.1798 | 1.2395 | 1.4428 | 0.9703 | 1.1793 | 0.3541 |
| Gorai.009G040700 | 1.4804 | 1.5869 | 1.8847 | 1.2867 | 0.7404 | 0.5024 | 0.7340 | 1.4624 | 1.4425 | 1.3946 |
| Gorai.009G040800 | -0.6778 | -0.9586 | -0.5229 | -0.3565 | 0.3444 | -0.2007 | 0.0000 | -0.4815 | -0.2076 | 0.0000 |
| Gorai.009G040900 | -0.3010 | 0.0000 | -0.9586 | -0.1367 | -0.4202 | -0.8239 | 0.0000 | 0.0000 | 0.0000 | 0.0000 |
| Gorai.009G041000 | 1.6635 | 1.8391 | 1.6229 | 1.6717 | 1.7153 | 1.6504 | 1.5416 | 1.9898 | 1.7292 | 1.7764 |
| Gorai.009G041100 | 0.0000 | 0.0000 | -1.3979 | 0.0000 | 0.0000 | -0.7447 | 1.7546 | -0.8239 | 0.0000 | 0.0000 |
| Gorai.009G041200 | 1.7036 | 1.7565 | 1.7327 | 1.7151 | 1.7768 | 2.1515 | 1.2911 | 1.1310 | 1.1278 | 1.0394 |
| Gorai.009G041300 | 1.2704 | 1.3058 | 1.4437 | 1.4888 | 1.3771 | 1.6062 | 1.1810 | 1.1523 | 1.2986 | 0.8116 |
| Gorai.009G041700 | 0.0569 | -0.0044 | 0.0000 | -0.1024 | -0.1024 | 0.0000 | 1.3300 | 1.3147 | 0.0000 | 0.0000 |
| Gorai.009G041800 | 0.7931 | 1.2087 | 1.0924 | 1.2716 | 1.1255 | 0.9731 | 0.6972 | 1.1245 | 1.2475 | 0.8470 |
| Gorai.009G041900 | -0.1675 | 0.7152 | 0.5490 | 0.5877 | 0.6656 | 0.6656 | -0.2147 | 0.3655 | 0.6355 | 0.0000 |
| Gorai.009G042000 | -0.0315 | -0.6383 | -0.2676 | 0.9079 | -0.2441 | 2.2636 | -0.6778 | -0.4202 | -0.1487 | 0.0000 |
| Gorai.009G042100 | 2.6754 | 2.1054 | 2.8147 | 3.0204 | 2.3645 | 2.1238 | 0.8609 | 2.7350 | 2.7945 | 2.5845 |
| Gorai.009G042200 | 0.5490 | 0.5105 | 0.7101 | 0.6758 | 0.7672 | 1.0504 | 0.8325 | 0.9227 | 0.9112 | 0.7160 |
| Gorai.009G042300 | 0.7218 | -0.3372 | 0.5465 | 0.2430 | 0.0000 | -1.1549 | 0.6010 | 0.8938 | 0.0000 | 0.0000 |
| Gorai.009G042400 | 0.0000 | -0.8539 | 0.0000 | 0.4969 | 0.0000 | -0.7959 | 1.6152 | 0.7536 | 0.0000 | -1.5229 |
| Gorai.009G042500 | 0.2856 | 0.4014 | 0.3747 | 0.1847 | 0.1875 | -0.3372 | -1.3979 | -0.6778 | -0.4089 | 0.0000 |
| Gorai.009G042600 | 0.0000 | -0.3979 | -0.5850 | 0.0000 | -1.0458 | -0.3468 | 1.2714 | 0.7931 | -0.5528 | 0.4393 |
| Gorai.009G042700 | 0.6274 | 0.9926 | -1.5229 | 0.0000 | -0.1739 | -2.0000 | 1.1313 | 1.0216 | 0.0000 | 0.0000 |
| Gorai.009G045700 | 0.0000 | -0.2757 | -0.8539 | -0.8239 | 0.0000 | 0.0000 | -0.1024 | -0.0177 | -0.4318 | 0.1461 |
| Gorai.009G045800 | -0.2007 | 0.9800 | 0.1790 | 0.0792 | 0.5888 | 0.3747 | 1.1889 | 0.6253 | 0.2945 | 0.0000 |
| Gorai.009G045900 | -0.0506 | 0.0000 | 0.1206 | 0.5740 | -0.4559 | -0.0809 | 0.4564 | 0.2122 | 0.2878 | 0.0000 |
| Gorai.009G051800 | 0.7235 | 0.5966 | 0.4487 | 0.3502 | 0.5899 | 0.7324 | 1.2423 | 0.5955 | 0.6493 | 0.4518 |
| Gorai.009G051900 | 1.7402 | 1.3553 | 1.6211 | 1.7693 | 1.6465 | 1.7514 | 2.4008 | 1.7275 | 1.8298 | 1.4978 |
| Gorai.009G052000 | 0.0000 | 0.0000 | 0.0000 | -1.0969 | -1.0000 | -0.6576 | -0.4318 | -1.2218 | -0.9208 | 0.0000 |
| Gorai.009G052100 | 1.5722 | -0.2007 | 0.8357 | 1.2594 | -0.0132 | 1.5180 | 2.0630 | 1.8145 | 1.6035 | 1.9052 |
| Gorai.009G052200 | 2.7751 | 0.9872 | 2.0800 | 1.8868 | 0.1106 | -0.0809 | 1.2170 | 2.3599 | 1.0141 | 2.2311 |
| Gorai.009G052300 | 0.9996 | 0.8169 | 0.7093 | 0.6628 | 0.5999 | 0.5563 | -0.6198 | 0.0000 | 0.0000 | 0.0000 |
| Gorai.009G059800 | 1.4548 | 1.3959 | 1.3506 | 1.2840 | 1.2453 | 1.2325 | 1.1307 | 1.2066 | 1.0976 | 1.2363 |
| Gorai.009G059900 | 0.6304 | 1.0382 | 0.2253 | 0.0934 | -0.0655 | -0.2366 | 0.5159 | 0.7701 | 0.5705 | 0.7474 |
| Gorai.009G060000 | -0.4437 | -0.4949 | 0.2068 | 0.7839 | 0.5539 | 0.3304 | 0.5888 | 0.6821 | -0.2291 | 0.2900 |
| Gorai.009G060100 | -0.5850 | -0.6576 | -0.9586 | 0.0043 | -0.0362 | -0.2840 | 0.5011 | 0.8500 | 1.0588 | 0.2330 |
| Gorai.009G060200 | 0.0000 | -0.3372 | -0.2596 | -0.0269 | 0.0253 | -0.0315 | 0.7709 | 0.8000 | 0.0043 | 0.0000 |
| Gorai.009G066600 | 0.0000 | 0.0000 | -0.6198 | 0.0000 | 0.0000 | 0.0000 | 1.2732 | 1.4458 | 0.0000 | 0.0000 |
| Gorai.009G066700 | 0.0000 | -0.0223 | -0.4685 | 0.3997 | 0.7292 | 0.4216 | -0.0044 | 0.5453 | 0.0000 | 0.6201 |
| Gorai.009G066800 | 0.0086 | 0.3747 | 0.2201 | 0.2648 | 0.3729 | 0.4281 | 0.0128 | 0.2380 | 0.1038 | -0.2676 |
| Gorai.009G066900 | 0.3464 | -0.2757 | 0.1614 | 0.2878 | 0.7340 | 0.6031 | 0.6830 | 1.2960 | 1.3923 | 1.0788 |
| Gorai.009G078000 | 0.0000 | 0.0000 | 0.0000 | 0.0000 | 0.0000 | 0.0000 | 0.0000 | 0.0000 | 0.0000 | 0.0000 |
| Gorai.009G078100 | 0.0000 | 0.0000 | -1.5229 | 0.0000 | 0.0000 | 0.0000 | 0.0000 | -0.8861 | 0.0000 | -0.6021 |
| Gorai.009G078200 | 0.5185 | 0.5514 | 0.9289 | 0.8014 | 0.7152 | 0.2480 | 1.1159 | 0.8293 | 0.8573 | 0.7243 |
| Gorai.009G078300 | 0.4871 | 0.9025 | 0.5340 | 0.7774 | 0.0719 | 0.5465 | 1.1884 | 0.6821 | -0.5376 | 0.3118 |
| Gorai.009G078400 | 0.7597 | 2.0340 | 1.3773 | 1.2033 | 0.9175 | 1.4060 | 1.4103 | 1.5868 | 1.5656 | 1.3170 |
| Gorai.009G078500 | -0.1079 | 0.2856 | -0.2291 | -0.0706 | 0.4502 | 0.1072 | -1.0458 | 0.0000 | 0.0000 | 0.0000 |
| Gorai.009G078600 | 0.0000 | 0.0000 | -2.0000 | -1.5229 | -0.9208 | 0.0000 | -0.7212 | -0.6990 | -1.3979 | -0.4949 |
| Gorai.009G082000 | 2.0190 | 2.1258 | 2.1155 | 2.0270 | 1.8448 | 1.5748 | 2.2196 | 2.0560 | 1.9846 | 2.0566 |
| Gorai.009G082100 | 1.0430 | 0.7324 | 1.0314 | 1.0398 | 0.6972 | 0.7093 | 0.8779 | 1.0755 | 0.8745 | 1.1611 |
| Gorai.009G082200 | 1.1738 | 1.3703 | 1.2856 | 1.2284 | 1.1644 | 1.1146 | 0.7860 | 1.2693 | 1.3483 | 1.0853 |
| Gorai.009G082300 | 0.0000 | -0.2218 | 0.0000 | 0.0000 | 0.0000 | -0.5086 | 0.8129 | 0.8096 | 0.0000 | 0.0000 |
| Gorai.009G084500 | 0.0000 | 0.0000 | -0.1805 | 0.0000 | -0.6778 | 0.9074 | 0.4378 | 1.1156 | 0.0000 | 0.9201 |
| Gorai.009G084600 | 0.2765 | 0.3365 | 0.3617 | 0.3945 | 0.7267 | 1.4087 | 0.3598 | 0.6928 | -0.1192 | 0.9201 |
| Gorai.009G084700 | 0.7482 | 0.1732 | 0.8537 | 0.8733 | 1.0273 | 1.8290 | 0.5658 | 0.0000 | 0.0000 | 0.0000 |
| Gorai.009G084800 | 0.0000 | 1.6383 | -0.7212 | 0.2095 | -0.2840 | -0.3468 | 1.2760 | 0.3766 | -0.3665 | 0.3054 |
| Gorai.009G084900 | 0.0000 | 0.0000 | 0.0000 | 0.0000 | 0.0000 | 0.0000 | 0.7110 | 0.0000 | 0.0000 | 0.0000 |
| Gorai.009G085000 | 0.0000 | 0.0000 | -0.6383 | 0.0000 | -0.6383 | -0.2007 | 0.4183 | -0.0605 | -1.6990 | 0.0000 |
| Gorai.009G085100 | 0.1523 | -0.0223 | -0.6576 | -0.9208 | -0.4815 | -0.4318 | 1.6219 | 1.4185 | 0.0000 | 0.0000 |
| Gorai.009G085200 | 1.7758 | 1.7985 | 1.8450 | 1.8347 | 1.8533 | 1.7550 | 1.6778 | 1.9684 | 2.1157 | 1.6094 |
| Gorai.009G085300 | 0.0000 | 0.0000 | -1.0969 | 0.0000 | 0.0000 | 0.0000 | 0.8202 | 0.9694 | 0.0000 | 0.0000 |
| Gorai.009G085400 | 1.2340 | 1.0990 | 1.1670 | 1.3137 | 1.5762 | 1.6104 | 2.3165 | 2.0976 | 1.5446 | 1.4943 |
| Gorai.009G085500 | 0.0000 | 0.0000 | 0.0000 | 0.0000 | -0.6576 | 0.0043 | 0.0000 | 0.0000 | 0.0000 | 0.0000 |
| Gorai.009G085600 | 1.4347 | 1.6884 | 1.8203 | 1.8044 | 2.0011 | 2.3858 | 1.9590 | 1.8605 | 1.7101 | 1.5656 |
| Gorai.009G085700 | -0.9586 | -0.9586 | -1.6990 | 0.2122 | 0.6866 | 0.4281 | -1.0458 | -1.3979 | 0.0000 | 0.0000 |
| Gorai.009G085800 | 2.2290 | 2.0891 | 2.1961 | 2.3401 | 2.3184 | 2.7278 | 2.0811 | 2.1434 | 2.1309 | 2.0880 |
| Gorai.009G085900 | 0.0000 | 0.0000 | 0.0000 | -0.2518 | -1.5229 | -1.3979 | 0.6902 | 0.0000 | 0.0000 | 0.0000 |
| Gorai.009G086000 | 0.4548 | 0.3820 | 0.5575 | 0.4900 | 0.5378 | 0.2878 | 0.3636 | 0.4609 | 0.4594 | 0.3927 |
| Gorai.009G086100 | -0.3188 | -0.2676 | -0.6576 | 0.1931 | -0.2518 | -0.5686 | 2.7513 | 0.0000 | 0.0000 | 0.0000 |
| Gorai.009G086200 | 0.0000 | 0.0000 | -1.0969 | 0.0000 | -0.5229 | 1.4604 | -0.0757 | -0.6778 | 0.0000 | -0.5376 |
| Gorai.009G086900 | 0.0000 | 0.7016 | 0.0000 | 0.3674 | -0.1249 | -0.4815 | -0.9208 | -0.6383 | -0.7959 | -0.5528 |
| Gorai.009G087000 | -0.3565 | 0.2945 | 0.3444 | 0.8751 | 0.6721 | 0.6803 | 0.8791 | 0.0000 | 0.0000 | 0.0000 |
| Gorai.009G087100 | 0.9791 | 0.7067 | 1.3481 | 1.1116 | 1.2201 | 1.0224 | 1.5842 | 0.8965 | 0.7193 | 0.9694 |
| Gorai.009G087200 | 0.0000 | 0.0000 | -0.3565 | -0.3188 | -0.7447 | -1.6990 | -0.0915 | 0.1335 | 0.0000 | 0.0000 |
| Gorai.009G092100 | 0.0000 | 0.0000 | 0.0000 | 0.0000 | 0.0000 | 0.0000 | 1.4148 | 1.4468 | 0.0000 | 0.0000 |
| Gorai.009G092200 | -0.7959 | -0.2007 | -0.7212 | 0.8420 | 1.9839 | 2.0880 | 1.3758 | 0.9015 | 0.9859 | 0.7076 |
| Gorai.009G092300 | 0.9689 | 1.2060 | 1.2470 | 1.6125 | 1.8199 | 1.6570 | 1.9357 | 1.8592 | 1.1038 | 2.0789 |
| Gorai.009G092400 | 0.6875 | 0.9180 | 0.9253 | 1.1096 | 0.7604 | 0.4843 | 0.8904 | 0.7459 | 0.5551 | 0.8182 |
| Gorai.009G098200 | 0.0000 | 0.0000 | 0.0000 | 0.0000 | 0.0000 | 0.0000 | -0.6576 | -0.2840 | 0.0000 | -0.0223 |
| Gorai.009G098300 | 0.0000 | -0.3872 | -0.2840 | -0.3665 | -0.7959 | -0.3098 | 1.1225 | 1.1593 | -0.3372 | 0.0000 |
| Gorai.009G098400 | 1.3899 | 1.5790 | 1.4794 | 1.3922 | 1.4186 | 1.4323 | 1.3170 | 1.6212 | 1.6012 | 1.5736 |
| Gorai.009G098500 | 1.1818 | 0.8069 | 1.1850 | 1.0269 | 0.9818 | 0.7952 | 1.2472 | 1.0457 | 1.1520 | 0.8082 |
| Gorai.009G098600 | -0.0605 | 0.6304 | -1.0969 | -0.5850 | 0.0000 | 0.0000 | -2.0000 | 0.0000 | 0.0000 | 0.0000 |
| Gorai.009G098700 | 0.3222 | 0.1367 | -0.0315 | 0.2175 | 0.4639 | 0.2122 | -0.1192 | 0.0755 | 0.3444 | 0.0000 |
| Gorai.009G107600 | 0.9375 | 0.8195 | 1.1255 | 0.7160 | 0.8837 | 0.7694 | 1.3758 | 1.2964 | 1.1146 | 1.3493 |
| Gorai.009G107700 | 0.0000 | 0.0000 | -0.0506 | -0.6198 | 0.0000 | 0.0000 | 0.9435 | 1.0785 | 0.0000 | 0.0000 |
| Gorai.009G107800 | 0.7300 | 0.6138 | 0.6160 | 0.6160 | 0.6522 | 0.4624 | 0.3160 | 0.2279 | 0.0531 | 0.2480 |
| Gorai.009G107900 | 0.1004 | -0.0555 | 0.3838 | 0.2279 | 0.0719 | 0.2148 | 0.9863 | 1.4695 | -0.3768 | -0.4318 |
| Gorai.009G108000 | 0.0000 | 0.0000 | -1.3010 | -1.1549 | -1.1549 | 0.0000 | -0.3665 | 0.2455 | 0.0000 | 0.0000 |
| Gorai.009G108100 | 0.0000 | 1.2122 | 0.0000 | 0.2304 | -0.0132 | 0.3243 | 1.0993 | -1.2218 | 0.0000 | 0.0000 |
| Gorai.009G108200 | 1.0253 | 1.0630 | 0.6128 | 0.2480 | 0.5966 | 0.2810 | 1.3537 | 1.2092 | 0.5922 | 1.0116 |
| Gorai.009G110300 | 0.0000 | 0.0000 | 0.0000 | -1.5229 | 0.0000 | -0.8539 | 0.0682 | -1.5229 | -1.2218 | 0.0000 |
| Gorai.009G110400 | 0.4579 | 0.2923 | 0.3674 | 0.5490 | 0.9513 | 0.8733 | 1.4807 | 0.4843 | -0.4949 | 0.0000 |
| Gorai.009G110500 | 2.6317 | 1.2679 | 1.4904 | 1.4260 | 0.0792 | -0.2757 | 1.3032 | 1.7185 | 1.6748 | 1.6947 |
| Gorai.009G110600 | 0.0000 | -0.0362 | -0.3872 | -1.2218 | 0.0000 | 0.0000 | 0.2923 | 0.6998 | 0.0000 | 0.0000 |
| Gorai.009G110700 | 0.3118 | -0.4089 | 0.0374 | 0.4624 | -0.6198 | 0.0000 | 0.7243 | 1.7236 | 1.5214 | 1.1474 |
| Gorai.009G112600 | 1.3047 | 1.2512 | 1.7403 | 1.3071 | 0.4456 | 0.4742 | 0.4065 | 2.1433 | 1.7942 | 2.2887 |
| Gorai.009G112700 | 0.6170 | 0.4065 | 0.8457 | 0.6875 | 0.8971 | 1.1392 | 0.6590 | 0.1492 | 0.0828 | 0.1461 |
| Gorai.009G112800 | 0.7760 | 0.9375 | 0.9533 | 0.9489 | 1.0449 | 1.0973 | 0.9504 | 0.9269 | 0.9741 | 0.7952 |
| Gorai.009G112900 | 0.0000 | 0.0000 | 0.0000 | 0.0000 | 0.0000 | 0.0000 | 0.0000 | 0.0000 | 0.0000 | 0.0000 |
| Gorai.009G113000 | -0.2076 | -0.2291 | -0.7959 | -0.4949 | -0.9208 | 0.0000 | 1.4445 | -0.3468 | -0.3468 | 0.0000 |
| Gorai.009G120200 | 0.0000 | 0.0000 | -0.4318 | -1.3979 | -1.6990 | -1.5229 | 0.0000 | -1.5229 | -1.3010 | 0.0000 |
| Gorai.009G120300 | 0.5185 | 0.0000 | -0.3279 | 0.0000 | 0.0000 | 0.0000 | 1.3685 | 0.2625 | 0.0000 | 0.0000 |
| Gorai.009G120400 | 0.0000 | 0.0000 | 0.0000 | -2.0000 | 0.0000 | 0.0000 | 0.8482 | -0.7959 | -1.0458 | 0.0000 |
| Gorai.009G120500 | 0.0000 | 0.0000 | -1.3010 | 0.0000 | -1.6990 | -1.6990 | -1.1549 | -1.6990 | -1.3979 | 0.0000 |
| Gorai.009G120600 | 0.0000 | 0.0000 | -1.0458 | -0.1487 | 0.0000 | -0.5686 | 1.4489 | 1.7615 | 0.0000 | 0.0000 |
| Gorai.009G124500 | -0.4202 | -0.4559 | 0.1732 | 0.0414 | 0.2648 | -0.4559 | 1.2490 | -0.0410 | -0.1612 | 0.0000 |
| Gorai.009G124600 | 0.5441 | -0.2218 | -0.4437 | -0.5850 | -0.7447 | -0.5086 | -1.3010 | -1.0969 | -0.8239 | 0.0000 |
| Gorai.009G124700 | 0.8432 | 0.2227 | 0.5551 | 0.6972 | 0.6031 | 0.9154 | 0.9713 | 0.8351 | 1.0099 | 0.3945 |
| Gorai.009G124800 | 1.0111 | 0.3404 | 0.9750 | 0.9494 | 0.5988 | 0.3502 | 0.5575 | 0.1303 | 0.3365 | -0.4559 |
| Gorai.009G124900 | 0.9930 | 0.4914 | 0.2945 | 0.5224 | 0.6721 | 0.5911 | 1.2824 | 0.9385 | 0.0000 | 0.1173 |
| Gorai.009G127700 | 0.5502 | 0.6107 | 0.7412 | 0.7267 | 0.7185 | 0.9004 | 0.8189 | 1.0004 | 1.0035 | 0.9274 |
| Gorai.009G127800 | 0.8395 | 1.1917 | 0.5999 | 0.4594 | 0.2833 | 0.1004 | 1.2276 | 1.4539 | 0.9547 | 1.1864 |
| Gorai.009G127900 | -0.9586 | 0.0086 | -1.1549 | 1.2887 | 1.0881 | 0.8129 | 0.8122 | 0.4871 | 0.4014 | 0.4997 |
| Gorai.009G128000 | -2.0000 | 0.0000 | 0.6405 | 0.4594 | 0.8482 | 0.6981 | 0.9405 | 1.0170 | 0.6435 | 1.0941 |
| Gorai.009G128100 | 0.0000 | -2.0000 | 0.0000 | -0.4202 | -2.0000 | -2.0000 | 0.6325 | 0.6551 | 0.0000 | 0.0000 |
| Gorai.009G128200 | 0.0000 | -0.0757 | -0.7212 | 0.5809 | 2.2919 | 2.9658 | -0.8239 | 0.0000 | 0.0000 | 0.0000 |
| Gorai.009G128300 | -2.0000 | 0.0000 | 0.0719 | -1.0458 | -0.7696 | 0.0000 | -0.3768 | 0.0128 | 0.1614 | -0.3279 |
| Gorai.009G128400 | -0.6990 | -1.0458 | -0.8239 | -0.0177 | 0.1523 | -0.4318 | 1.1550 | -0.0915 | 0.0969 | -0.6021 |
| Gorai.009G128500 | 0.7388 | 1.7371 | 0.4440 | 0.7818 | 0.6646 | 0.1271 | 1.4128 | 0.6484 | 0.6884 | 0.5276 |
| Gorai.009G128600 | 0.8657 | 1.7258 | 0.3324 | 0.7202 | 0.6628 | 0.0000 | 0.7050 | 0.1903 | 0.1584 | 0.1553 |
| Gorai.009G130400 | 0.0000 | -0.4685 | -0.2518 | 0.3324 | -0.3665 | 0.1399 | 1.0531 | 0.6767 | 0.0000 | 0.0000 |
| Gorai.009G130500 | 1.4714 | 1.4739 | 1.1281 | 1.4231 | 1.6497 | 1.1440 | 1.3237 | 1.5186 | -0.1192 | 1.7781 |
| Gorai.009G130600 | 1.4237 | 1.3502 | 1.5478 | 1.4363 | 1.3531 | 1.2591 | 1.7976 | 1.4814 | 1.3872 | 1.4472 |
| Gorai.009G130700 | 1.6128 | 1.0763 | 1.2292 | 1.5405 | 1.7099 | 1.2350 | 2.1626 | 1.4576 | 1.3595 | 1.4720 |
| Gorai.009G130800 | 0.0000 | -0.6990 | -1.3979 | 0.0000 | 0.0000 | 0.0000 | -0.9586 | -0.4815 | 0.0000 | -0.2147 |
| Gorai.009G130900 | 0.7818 | -0.6778 | 0.0607 | -0.5086 | 0.2625 | 0.4564 | -0.2676 | 0.8407 | 1.0615 | 0.1367 |
| Gorai.009G131000 | 1.4971 | 1.2310 | 1.4294 | 1.3464 | 1.2844 | 1.1793 | 1.4040 | 1.1287 | 1.1934 | 0.9703 |
| Gorai.009G131100 | -0.0506 | -0.9586 | -0.2840 | 0.2330 | 0.3424 | 1.7588 | 0.2304 | -0.8539 | 0.0000 | -0.5850 |
| Gorai.009G131200 | 0.2788 | 0.7832 | 0.4298 | 0.2355 | 0.4871 | 0.8357 | 0.7966 | 0.9350 | 0.7543 | 0.9638 |
| Gorai.009G131300 | -0.5686 | 0.5527 | 0.5877 | 0.9335 | 0.3075 | 0.1367 | 1.0888 | 1.7788 | -0.3665 | 2.0104 |
| Gorai.009G131400 | 0.0000 | -1.0969 | -1.0000 | -0.3565 | -1.0000 | -0.8239 | 0.5705 | -0.5528 | -0.2840 | 0.0000 |
| Gorai.009G131500 | 0.0000 | 0.0000 | 0.0000 | 0.0000 | 0.0000 | -0.4089 | -1.0000 | 0.0000 | 0.0000 | 0.0000 |
| Gorai.009G131600 | 0.0000 | 0.0000 | -2.0000 | 0.0000 | 0.0000 | 0.0000 | -0.2840 | -1.5229 | -1.2218 | 0.0000 |
| Gorai.009G142000 | 1.0310 | 0.0000 | 0.7910 | 0.9509 | 1.1199 | 1.1578 | 1.1477 | 1.5501 | 1.2984 | 1.6458 |
| Gorai.009G142100 | 0.7987 | 0.3139 | 0.3118 | 0.2068 | 0.5079 | 0.4393 | 0.7380 | 0.7497 | 0.6385 | -1.6990 |
| Gorai.009G142200 | 1.5868 | 1.5655 | 1.4771 | 1.1735 | 0.0645 | -0.5686 | -0.2924 | 1.3047 | 0.5038 | 1.1136 |
| Gorai.009G142300 | 0.2878 | -0.2596 | 0.4983 | 0.5453 | -0.0088 | -0.0605 | 0.5011 | 0.6920 | 0.7731 | 0.5038 |
| Gorai.009G149100 | 0.0000 | 0.0000 | 0.0000 | 0.0000 | 0.0000 | 0.0000 | 0.9818 | 0.5551 | 0.5011 | 0.0000 |
| Gorai.009G149200 | -0.6021 | -0.2676 | -0.1549 | -0.0177 | 0.0086 | -0.4089 | 1.4738 | -0.5086 | -0.7447 | 0.0000 |
| Gorai.009G149300 | 0.9133 | 1.1149 | 1.1836 | 1.2276 | 1.1602 | 0.6096 | 1.5245 | 1.9630 | 1.1146 | 2.1369 |
| Gorai.009G149400 | 0.3892 | 0.4728 | 0.6201 | 0.6972 | 0.7168 | 0.6893 | 0.7210 | 0.6117 | 0.7160 | 0.3802 |
| Gorai.009G149500 | 0.0000 | -0.3010 | 0.3304 | 0.4440 | 0.4116 | 0.8982 | 0.4362 | 0.8910 | 0.0000 | 1.0542 |
| Gorai.009G149600 | 0.0000 | 0.2648 | -0.4089 | -0.1427 | 0.4200 | 0.4533 | 0.2878 | 0.1106 | -0.1871 | 0.0000 |
| Gorai.009G166800 | 0.9469 | 0.5441 | 1.1072 | 0.8089 | 0.5105 | 0.1790 | 0.5611 | 0.6201 | 0.4594 | 0.6839 |
| Gorai.009G166900 | 0.6191 | 1.0330 | 1.4962 | 1.0781 | 1.3185 | 1.1535 | 1.1901 | 0.9106 | 0.7903 | 0.9489 |
| Gorai.009G167000 | 3.1738 | 2.1908 | 2.4023 | 2.0240 | -0.2007 | 0.1399 | 2.0812 | 2.9357 | 1.9775 | 2.6005 |
| Gorai.009G167100 | 1.2929 | 0.7536 | 1.0554 | 0.6010 | 0.3802 | -0.1249 | 0.2878 | 0.9036 | 0.9943 | 0.6972 |
| Gorai.009G167200 | 2.1899 | 2.0855 | 2.1242 | 1.9664 | 1.8833 | 1.8338 | 1.5933 | 2.0055 | 1.7683 | 2.1084 |
| Gorai.009G167300 | -0.3279 | -0.7212 | -0.2366 | -0.3768 | -0.4202 | 0.1584 | 0.4378 | 0.3522 | 0.2788 | 0.3560 |
| Gorai.009G167400 | 0.9657 | 0.6335 | 0.7226 | 0.3243 | 0.8669 | 0.5366 | 0.7451 | 0.1987 | 0.3118 | 0.0000 |
| Gorai.009G167500 | 2.2025 | 2.7526 | 2.3599 | 2.6120 | 2.9064 | 2.3659 | 2.6466 | 3.1038 | 2.3320 | 3.3272 |
| Gorai.009G167600 | 0.0000 | 0.2765 | -0.0362 | 0.1584 | -0.2218 | 0.1761 | 0.1461 | 0.5276 | 0.0000 | 0.0000 |
| Gorai.009G176800 | -0.4202 | -0.3665 | 1.0120 | 0.4533 | -0.1135 | -0.6021 | 1.5111 | 1.5243 | 1.3363 | 1.1514 |
| Gorai.009G176900 | 0.7582 | 0.4031 | 0.9513 | 0.8445 | 0.8808 | 1.0145 | 1.1889 | 0.4886 | 0.4771 | 0.4330 |
| Gorai.009G177000 | -0.1192 | -0.1549 | -0.2076 | -0.3979 | -0.2147 | -0.3188 | -0.4318 | -0.1427 | 0.0212 | -0.5528 |
| Gorai.009G177100 | 0.9685 | 0.8756 | 1.2375 | 0.8899 | 0.4216 | 0.8698 | 1.3288 | 1.1072 | 0.0000 | 0.0000 |
| Gorai.009G177200 | 1.6238 | 1.7886 | 1.7295 | 1.7035 | 1.4823 | 1.3722 | 1.7081 | 1.9661 | 1.8843 | 1.9716 |
| Gorai.009G177300 | 0.0000 | 0.0000 | -0.0044 | -0.4202 | -1.0000 | 0.0000 | 0.0000 | -0.6576 | -0.3979 | 0.0000 |
| Gorai.009G177400 | -0.1549 | 0.0000 | -0.7696 | -1.0458 | 0.0000 | 0.0000 | 0.0000 | 0.8401 | -0.3979 | 0.0000 |
| Gorai.009G177500 | -0.4949 | 0.0969 | -0.6576 | -0.0315 | -0.3010 | -0.1427 | -0.2076 | -1.2218 | -0.9208 | 0.0000 |
| Gorai.009G177600 | 0.9053 | 0.1818 | 1.0228 | 0.0969 | -0.1739 | -0.3188 | 1.4115 | 0.8555 | 1.0492 | 0.3284 |
| Gorai.009G222500 | 0.6149 | 0.8751 | 0.9335 | 0.8142 | 0.7226 | 0.8609 | 0.9294 | 0.6425 | 0.7482 | 0.3784 |
| Gorai.009G222600 | 1.0584 | 1.0535 | 0.8681 | 0.8494 | 0.6064 | 0.3802 | 0.4843 | 0.9965 | 1.0795 | 0.8055 |
| Gorai.009G222700 | 0.0000 | 0.0000 | 0.0000 | 0.0000 | 0.0000 | -1.1549 | 0.0000 | 0.0000 | 0.0000 | 0.0000 |
| Gorai.009G222800 | -0.1427 | -0.7212 | 0.0682 | 0.1072 | 0.1847 | -0.2518 | -0.1427 | 0.1818 | 0.2304 | -0.6990 |
| Gorai.009G222900 | 0.0000 | 0.0000 | 0.0000 | -1.1549 | -0.5376 | -0.7212 | -0.9586 | -0.4949 | -1.1549 | -0.2840 |
| Gorai.009G229300 | 1.4944 | 1.4014 | 1.2243 | 1.1229 | 1.0976 | 1.3406 | 1.4832 | 1.5887 | 0.0000 | 1.4188 |
| Gorai.009G229400 | 0.0000 | 0.0000 | -0.4949 | 0.0000 | -0.4202 | 0.0000 | 0.8136 | 0.9494 | 0.8096 | 0.7101 |
| Gorai.009G229500 | 0.0864 | -0.0315 | -0.4685 | -0.7447 | 0.0000 | -0.6198 | 0.7789 | 1.0290 | -0.5086 | 0.0000 |
| Gorai.009G229600 | 0.4983 | 0.6201 | 0.5403 | 0.3729 | 0.5575 | 0.7536 | 1.1781 | 1.3716 | 0.8561 | 1.2655 |
| Gorai.009G229700 | 0.1206 | 0.0645 | 0.1430 | -0.7447 | 0.3139 | 0.1399 | -0.1549 | -0.8861 | -0.6198 | 0.0000 |
| Gorai.009G229800 | 0.6284 | 0.6857 | 0.8621 | 0.8331 | 0.8312 | 0.7218 | 0.8488 | 1.5123 | 0.9675 | 1.2601 |
| Gorai.009G251100 | 0.0000 | -0.4437 | 0.0000 | -1.5229 | 0.0000 | 0.0000 | -1.0000 | -1.5229 | -1.3010 | 0.0000 |
| Gorai.009G251200 | 0.0000 | -0.2076 | -1.0458 | -0.2840 | 0.7007 | 0.0086 | 0.3636 | 1.0853 | -0.2218 | 0.0000 |
| Gorai.009G251300 | 0.7796 | 0.0000 | 0.3324 | -0.3565 | -0.0506 | -0.1308 | 0.0374 | 0.2148 | -0.6990 | 0.2175 |
| Gorai.009G251400 | 1.0554 | 0.0000 | 0.9133 | 0.0492 | 0.8293 | 0.6758 | 0.4609 | 1.2368 | 0.4871 | 1.3840 |
| Gorai.009G251500 | -0.6778 | 0.0000 | -0.6383 | -1.0458 | 0.0000 | 0.0000 | 0.2529 | 0.8370 | 0.8899 | 0.6955 |
| Gorai.009G251600 | 0.8426 | -0.6990 | 0.7202 | 0.2648 | -0.6576 | -0.2757 | -0.2924 | 1.0358 | 1.1641 | 0.7451 |
| Gorai.009G251700 | 0.0969 | -0.1024 | -0.1549 | -0.5850 | -0.2596 | -1.3010 | 0.3674 | 0.4314 | 0.4757 | 0.3032 |
| Gorai.009G262100 | -0.6576 | -0.4089 | -0.6021 | -0.9208 | -0.0605 | -0.7696 | 0.4099 | -1.0969 | -0.8539 | 0.0000 |
| Gorai.009G262200 | 0.2788 | 1.3558 | 0.5809 | 0.8463 | -0.0044 | -0.1367 | 0.5821 | 0.6031 | 0.5527 | 0.5315 |
| Gorai.009G262300 | 1.3172 | 1.4830 | 1.4270 | 1.2658 | 1.2238 | 1.3101 | 1.1556 | 1.0370 | 1.0414 | 0.9624 |
| Gorai.009G262400 | 1.0060 | 0.0000 | 1.0519 | 1.0009 | 0.0000 | -0.2518 | 1.2560 | 1.5467 | 1.7068 | 1.1614 |
| Gorai.009G262500 | 0.1523 | 0.2577 | 0.3820 | 0.3579 | 0.2648 | 0.4742 | 0.1106 | 0.3766 | -0.0757 | 0.1106 |
| Gorai.009G262600 | -2.0000 | 0.2253 | 0.2068 | 0.4346 | 0.3160 | -0.0706 | -0.1367 | 0.1761 | -0.0177 | 0.0453 |
| Gorai.009G262700 | 0.0000 | 0.0000 | -1.0458 | 0.0000 | 0.0000 | 0.0000 | 0.0000 | -0.7959 | -0.5086 | 0.0000 |
| Gorai.009G272800 | 0.0000 | 0.0000 | 0.0000 | 0.0000 | 0.0000 | 0.0000 | 0.0000 | -1.0969 | -0.8539 | 0.0000 |
| Gorai.009G272900 | 0.0000 | 0.0000 | 0.0000 | 0.0000 | 0.0000 | 0.0000 | 0.0414 | 0.5514 | 0.0000 | 0.0000 |
| Gorai.009G273000 | 0.0000 | 0.0000 | -1.3010 | -1.0458 | 0.0000 | -1.2218 | 0.0000 | 0.0000 | 0.0000 | 0.0000 |
| Gorai.009G304600 | -1.1549 | -0.4437 | -0.8239 | 0.1875 | 0.0453 | -0.0315 | 0.0828 | 0.1790 | 0.3324 | -0.1938 |
| Gorai.009G304700 | 1.2543 | 1.2138 | 1.0550 | 1.0648 | 1.1535 | 0.9894 | 1.2025 | 1.3170 | 1.0469 | 1.4341 |
| Gorai.009G307700 | 1.0453 | 1.6284 | 0.4942 | 0.8035 | 1.1706 | 0.8645 | 0.5988 | 1.0253 | 0.9595 | 1.0216 |
| Gorai.009G308500 | 0.6739 | 0.8663 | 0.4116 | 0.0569 | 0.3444 | 0.2122 | 1.3404 | 1.0817 | 0.4200 | 0.0000 |
| Gorai.009G308600 | 0.0000 | 1.5038 | 0.0000 | 0.0000 | 0.0000 | 0.0000 | 0.4914 | 0.7738 | 0.0000 | 1.0386 |
| Gorai.009G308700 | 0.0000 | 0.0000 | 0.0000 | 0.0000 | 0.0000 | 0.0000 | 0.0000 | -0.3372 | -0.0655 | 0.0000 |
| Gorai.009G308800 | 0.0000 | 0.0000 | 0.0000 | 0.0000 | 0.0000 | 0.0000 | 0.0000 | 0.0000 | 0.0000 | 0.0000 |
| Gorai.009G308900 | 0.0000 | 0.0000 | 0.0000 | 0.0000 | -0.8539 | 0.0000 | 1.1726 | 1.0799 | 0.0000 | 0.0000 |
| Gorai.009G310400 | -1.3010 | -1.3010 | -2.0000 | 0.0000 | -2.0000 | 0.0000 | -2.0000 | -1.1549 | -0.8539 | 0.0000 |
| Gorai.009G310500 | 1.0386 | 1.0488 | 0.9390 | 0.8887 | 0.8189 | 0.7993 | 1.1052 | 1.5068 | 1.1414 | 1.2373 |
| Gorai.009G310600 | 0.4393 | 0.1004 | 0.6085 | 0.4624 | 0.4997 | 0.6160 | 1.5438 | 0.1173 | 0.1761 | -0.0315 |
| Gorai.009G311400 | 0.0000 | -0.7959 | 0.0000 | -1.1549 | -1.0969 | -1.3010 | -0.4089 | 0.0000 | 0.0000 | 0.0000 |
| Gorai.009G311500 | 0.5065 | 0.3541 | 0.6580 | 0.7938 | -0.2147 | -0.0269 | 1.3992 | 0.7664 | 0.3404 | 0.9335 |
| Gorai.009G313800 | 0.0000 | 0.0000 | 0.0000 | 0.0000 | -1.5229 | 0.0000 | -0.7959 | -1.6990 | -1.3010 | 0.0000 |
| Gorai.009G315600 | 1.1638 | 1.3549 | 1.4302 | 1.3860 | 1.1176 | 1.0550 | 0.9996 | 1.3296 | 1.2844 | 1.3071 |
| Gorai.009G315700 | 0.5502 | 0.0170 | 0.2672 | -0.2366 | 0.0000 | -0.0269 | 0.5276 | 0.0864 | 0.0000 | 0.0000 |
| Gorai.009G315800 | 0.6599 | 0.8209 | 1.2577 | 1.2978 | 1.1089 | 1.5352 | 2.1732 | 1.3166 | 1.2167 | 1.3397 |
| Gorai.009G318900 | 0.6646 | 0.5092 | 0.7396 | 0.5944 | 0.5092 | 0.8048 | 0.6684 | 1.1303 | 1.0162 | 1.1641 |
| Gorai.009G319000 | 0.4314 | 0.7435 | 0.3892 | 0.4116 | 0.2041 | 0.7033 | 1.0422 | 1.2806 | 1.2697 | 1.2230 |
| Gorai.009G319100 | 0.0000 | 0.0000 | 0.0000 | 0.0000 | 0.0000 | 0.0000 | 0.0000 | 0.0000 | 0.0000 | 0.0000 |
| Gorai.009G327300 | -1.3010 | 0.0000 | 0.0000 | -0.4437 | -0.9208 | -0.2076 | 1.3241 | 1.0488 | 0.0000 | 0.0000 |
| Gorai.009G328000 | 1.0573 | 1.0056 | 1.3520 | 1.1143 | 1.1807 | 1.4035 | 0.2355 | 0.4014 | 0.5428 | 0.0719 |
| Gorai.009G328100 | 0.5092 | -0.1367 | -0.3768 | 0.2455 | 0.0294 | -0.1135 | -1.5229 | -0.7959 | -0.5376 | 0.0000 |
| Gorai.009G328200 | 0.4014 | 0.0000 | 0.1732 | 0.0755 | 0.4133 | 0.6972 | 0.9258 | 1.0137 | 0.9370 | 0.0000 |
| Gorai.009G330400 | 0.0000 | 0.0000 | 0.0000 | 0.0000 | 0.0000 | 0.0000 | 0.0000 | 0.0000 | 0.0000 | 0.0000 |
| Gorai.009G403000 | -0.1427 | 0.5366 | 0.0969 | 0.7931 | 1.1471 | 0.4814 | 1.0867 | 0.3243 | 0.3118 | 0.2695 |
| Gorai.009G415100 | 0.0000 | 0.0000 | 0.0000 | 0.0000 | 0.0000 | 0.0000 | 0.0000 | 0.0000 | 0.0000 | 0.0000 |
| Gorai.010G008200 | 0.2068 | 0.8756 | 0.6117 | 0.7372 | 0.2148 | -0.3372 | 0.7267 | 0.9320 | 0.9425 | 0.8494 |
| Gorai.010G008300 | 0.1004 | -0.2076 | -1.5229 | 0.0000 | -0.8539 | 0.1139 | 2.1925 | 1.7130 | 0.0000 | 0.0000 |
| Gorai.010G008400 | -1.3979 | 0.0000 | 0.3118 | 0.1703 | -0.2007 | -0.0862 | -0.6990 | -0.2596 | 0.0000 | 0.0000 |
| Gorai.010G016000 | 0.0000 | 0.0170 | 0.1492 | 1.0026 | 0.9106 | 0.7664 | 0.4757 | 0.6767 | 0.9063 | -2.0000 |
| Gorai.010G016100 | 1.1532 | 0.8506 | 1.2494 | 1.2055 | 1.2865 | 1.3817 | 1.6801 | 1.3477 | 1.2175 | 1.3514 |
| Gorai.010G016200 | 0.5682 | 1.1872 | 1.2170 | 1.4283 | 1.4033 | 1.4539 | 1.3139 | 1.3831 | 1.2558 | 1.2524 |
| Gorai.010G016300 | 0.9671 | 0.7135 | 1.3162 | 1.4223 | 0.0755 | 0.1959 | 0.0969 | 0.9689 | 1.1565 | 0.4713 |
| Gorai.010G016400 | 1.1364 | 0.9415 | 1.1602 | 1.0584 | 0.9708 | 0.9036 | 2.3767 | 0.8681 | 0.7152 | 0.9274 |
| Gorai.010G016500 | -0.1739 | -0.5528 | 0.0086 | 0.1430 | 0.1790 | 0.2227 | 0.6335 | 0.0000 | 0.0000 | 0.0000 |
| Gorai.010G016600 | 1.1065 | 0.8122 | 1.1647 | 1.0792 | 1.1906 | 1.1928 | 0.4116 | 0.5843 | 0.3324 | 0.6937 |
| Gorai.010G016700 | 0.0000 | 0.0000 | 0.0000 | 0.0000 | 0.0000 | -0.0410 | 0.0000 | 0.0000 | 0.0000 | 0.0000 |
| Gorai.010G016800 | 0.2253 | -0.1079 | 0.3945 | 0.1761 | -0.7447 | -0.4202 | 0.6385 | 0.6712 | 0.8982 | -0.0915 |
| Gorai.010G016900 | 1.1605 | 1.0314 | 1.3558 | 1.2629 | 1.3361 | 1.6256 | 1.5885 | 1.3438 | 1.3804 | 1.2274 |
| Gorai.010G033400 | 0.0000 | -0.3872 | -0.6383 | -0.2441 | 0.0000 | 0.0000 | 1.2025 | 0.8791 | 0.0000 | 0.0000 |
| Gorai.010G033500 | 0.9420 | 0.9191 | 1.0422 | 1.0906 | 0.9814 | 1.0682 | 1.0504 | 1.0306 | 0.9547 | 1.0350 |
| Gorai.010G033600 | 0.0000 | -0.5229 | -0.6778 | -0.8539 | -0.7696 | 0.3729 | 0.5172 | -0.3372 | -0.2007 | -0.6576 |
| Gorai.010G033700 | -0.3872 | -0.1675 | -0.1079 | -0.1024 | -0.0269 | -0.0362 | -0.1549 | -0.3279 | -0.2676 | -0.4815 |
| Gorai.010G035900 | 1.5704 | 1.2636 | 1.4045 | 1.3489 | 1.1959 | 1.1906 | 0.6107 | 1.4216 | 0.9956 | 1.5887 |
| Gorai.010G036000 | 0.9186 | 0.8457 | 1.0434 | 0.9727 | 1.1804 | 1.3698 | 0.5198 | 0.7604 | 0.6454 | 0.7938 |
| Gorai.010G048100 | 2.4265 | 2.2856 | 2.2188 | 2.2252 | 2.1993 | 2.1303 | 1.9623 | 1.7821 | 1.7569 | 1.7402 |
| Gorai.010G048200 | 0.0000 | 0.1303 | -0.5686 | 0.0682 | 0.0000 | -0.6383 | 1.7543 | 0.7789 | 0.7076 | 0.0000 |
| Gorai.010G048300 | 1.0962 | 1.5930 | 1.2408 | 0.8228 | 0.9053 | 0.8149 | 0.8555 | 0.7543 | 0.8129 | 0.6053 |
| Gorai.010G058600 | -0.2518 | 0.0000 | 0.0000 | 0.0000 | 0.0000 | 0.0000 | -0.8539 | 0.0000 | 0.0000 | 0.0000 |
| Gorai.010G060300 | 0.5051 | 1.0366 | 0.7033 | 0.8797 | 0.6031 | 0.4942 | 1.0103 | 1.3174 | 1.2627 | 1.3038 |
| Gorai.010G060400 | 0.0000 | 0.0000 | 0.0000 | 0.0000 | -0.5850 | 0.0086 | 1.0910 | 1.0931 | -0.1249 | 0.0000 |
| Gorai.010G060500 | 1.6279 | 2.3294 | 1.2030 | 0.7789 | 0.2430 | -0.0223 | 1.2453 | 1.1069 | 1.2531 | 0.7672 |
| Gorai.010G063100 | 0.2504 | -0.4089 | 0.7924 | -0.4949 | -0.3468 | 0.3579 | -0.1739 | 0.0000 | 0.0000 | 0.0000 |
| Gorai.010G063200 | 1.0220 | 0.9112 | 0.0000 | 0.4133 | -0.1871 | 0.7619 | 1.3166 | 1.5719 | 1.5015 | 1.4322 |
| Gorai.010G064400 | 0.0000 | 0.0000 | 0.0000 | -0.2147 | 0.0000 | -0.2840 | 0.7226 | 0.1206 | 0.0000 | 0.0000 |
| Gorai.010G064500 | 1.2772 | 1.0777 | 1.2851 | 1.4465 | 1.3464 | 1.4470 | 1.4401 | 1.3692 | 1.4393 | 1.1970 |
| Gorai.010G064600 | 0.9528 | 0.6085 | 0.9504 | 0.5705 | 0.3502 | 0.4456 | 0.5024 | 0.4639 | 0.5145 | 0.3263 |
| Gorai.010G064700 | 0.1430 | 0.2718 | 0.2279 | 0.4393 | 0.2553 | 0.3222 | 0.8376 | 0.9047 | 0.0000 | 0.0000 |
| Gorai.010G072400 | 0.0000 | 0.0000 | 0.0000 | 0.0000 | 0.0000 | 0.0000 | 0.0000 | 0.0000 | 0.0000 | 0.0000 |
| Gorai.010G072500 | 0.0000 | 0.0000 | 0.0000 | 0.0000 | 0.0000 | 0.0000 | 0.0000 | -0.4949 | -0.2218 | 0.0000 |
| Gorai.010G072600 | 1.7250 | 1.6691 | 1.8023 | 1.9989 | 1.9248 | 2.2825 | 1.0086 | 1.2953 | 0.9689 | 1.4334 |
| Gorai.010G072700 | 0.0000 | 0.0000 | -0.4559 | -0.0132 | 0.1584 | -0.5086 | 0.4456 | 0.7076 | 0.4393 | 0.5752 |
| Gorai.010G072800 | 0.0000 | -1.1549 | -1.2218 | -0.6021 | -1.3010 | -1.3979 | -1.3010 | -0.3665 | -0.6778 | -0.2441 |
| Gorai.010G072900 | 1.7904 | 1.9139 | 1.7820 | 1.9293 | 1.8586 | 1.5058 | 1.0453 | 1.3404 | 1.3504 | 0.3802 |
| Gorai.010G073400 | 0.0000 | -0.0915 | -0.4685 | -0.6021 | -0.6198 | 0.3263 | -0.8539 | -0.0223 | -0.1805 | 0.0000 |
| Gorai.010G073500 | 0.0000 | 0.0000 | 0.0000 | 0.0000 | 0.0000 | 0.0000 | 0.0000 | -0.8861 | -0.6383 | 0.0000 |
| Gorai.010G073600 | 0.0000 | 0.0492 | -0.1675 | 0.2900 | 0.4116 | 0.0334 | 0.0934 | 0.3324 | 0.0934 | 0.3784 |
| Gorai.010G074600 | 0.0969 | 0.4200 | -0.1024 | 0.3945 | -0.0555 | -0.1612 | 0.3579 | 0.3222 | -2.0000 | 0.0000 |
| Gorai.010G074700 | -0.5229 | -0.1487 | -0.1487 | -0.2291 | -0.0132 | -0.1487 | -0.3098 | -1.3979 | -1.1549 | 0.0000 |
| Gorai.010G074800 | -1.0969 | -1.1549 | -1.0000 | -0.5850 | -0.5528 | -0.4949 | -0.2218 | -0.3565 | -0.2147 | -0.6778 |
| Gorai.010G074900 | 0.4487 | 0.9106 | 0.9159 | 0.6542 | 1.0241 | 0.7332 | 1.3400 | 1.2980 | 0.0000 | 0.7427 |
| Gorai.010G075000 | 0.0000 | 0.0000 | 0.0000 | 0.0000 | 0.0000 | 0.0000 | 0.0000 | 0.0000 | 0.0000 | 0.0000 |
| Gorai.010G075100 | 1.0473 | 1.1682 | 1.3696 | 1.1474 | 1.0366 | 0.7340 | 1.7497 | 1.0542 | 0.6675 | 1.2109 |
| Gorai.010G075200 | 0.6656 | 0.7686 | 0.8802 | 0.9415 | 0.9465 | 1.2772 | 1.2529 | 1.0504 | 1.0290 | 1.0043 |
| Gorai.010G078600 | 0.0000 | 0.0000 | -0.6990 | -0.7696 | 0.0000 | 0.0000 | 1.4522 | 1.3113 | 0.0000 | 0.7931 |
| Gorai.010G078700 | 1.4007 | -0.3188 | 0.2742 | 0.5502 | 0.4330 | 0.1173 | 0.1139 | -0.6576 | -0.3872 | 0.0000 |
| Gorai.010G078800 | 1.7105 | 1.3929 | 1.3789 | 1.2185 | 1.3377 | 1.2704 | 1.5737 | 1.5294 | 1.3118 | 1.6165 |
| Gorai.010G078900 | -0.7212 | 0.0000 | 0.0000 | 0.0000 | 0.0000 | 0.0000 | 0.6812 | 0.1206 | -0.2924 | 0.0000 |
| Gorai.010G080400 | 1.4898 | 1.5198 | 1.5397 | 1.5104 | 1.4544 | 1.5283 | 1.4509 | 1.6499 | 1.6740 | 1.5392 |
| Gorai.010G080500 | 1.8549 | 1.8405 | 2.1135 | 2.0006 | 1.8760 | 2.2214 | 2.4733 | 2.2376 | 2.2522 | 2.0988 |
| Gorai.010G080600 | 0.4594 | -0.2076 | -0.1079 | 0.4082 | 0.0000 | -0.6990 | 0.0000 | -0.6198 | -0.3565 | 0.0000 |
| Gorai.010G082500 | 0.8943 | 0.7543 | 1.1781 | 1.2284 | 1.1129 | 1.2885 | 0.9754 | 0.9350 | 0.9722 | 0.7959 |
| Gorai.010G082600 | -0.3468 | 0.0000 | 0.0000 | -0.1549 | 0.0000 | 0.0000 | 0.9170 | -0.2147 | 0.0000 | -0.1549 |
| Gorai.010G148000 | 0.0000 | -0.7696 | -1.3979 | -0.1549 | -0.1249 | -0.1308 | -0.3872 | -0.0706 | -0.7447 | 0.1430 |
| Gorai.010G148100 | 0.0000 | 0.0000 | -0.9586 | 0.4298 | 0.9227 | 0.5877 | -1.2218 | 0.3874 | -0.6778 | 0.6314 |
| Gorai.010G148200 | 0.5403 | 0.6542 | 0.8338 | 0.8182 | 0.9101 | 1.1878 | 0.5763 | -0.6576 | -0.3768 | 0.0000 |
| Gorai.010G164300 | 0.0719 | 0.5682 | 0.4425 | 0.5877 | 0.5250 | 0.7427 | 1.0596 | 1.4797 | 0.7686 | 0.9090 |
| Gorai.010G164400 | 1.1474 | 0.8463 | 1.1635 | 0.9624 | 0.9232 | 0.8293 | 1.7624 | 1.2335 | 1.2358 | 0.2148 |
| Gorai.010G164500 | 0.7076 | 0.7767 | 0.8633 | 0.8162 | 0.6304 | 0.8876 | 0.5551 | 1.1752 | 1.1995 | 1.0755 |
| Gorai.010G183700 | 0.0000 | 0.0000 | 0.0000 | 0.0000 | 0.0000 | 0.0000 | 0.0000 | 0.0000 | 0.0000 | 0.0000 |
| Gorai.010G183800 | 0.0000 | 0.0000 | 0.0000 | -1.0969 | -1.1549 | -0.7212 | 0.0000 | -1.0969 | 0.0000 | 0.0000 |
| Gorai.010G218400 | -0.0757 | -0.3665 | 0.2625 | 0.3674 | 0.1584 | -1.0969 | 0.4800 | 1.0374 | 1.1940 | 0.0000 |
| Gorai.010G218500 | 0.0000 | -1.0458 | -1.6990 | -1.6990 | -1.3979 | -1.5229 | -1.6990 | 0.0000 | 0.0000 | 0.0000 |
| Gorai.010G218600 | -0.4202 | 0.4065 | 0.4579 | 0.6222 | 0.3820 | -0.1249 | 0.7267 | -0.3098 | -0.0362 | 0.0000 |
| Gorai.010G231600 | 2.0209 | 2.1536 | 2.1673 | 2.0214 | 2.1684 | 2.2896 | 2.0668 | 2.0085 | 2.0446 | 1.8908 |
| Gorai.010G231700 | 0.4900 | 0.4166 | 0.5551 | 0.7372 | 0.8954 | 1.5410 | 0.8028 | 0.9269 | 0.8195 | 0.9552 |
| Gorai.010G231800 | 0.3424 | 0.5289 | 0.5729 | 1.0453 | 0.6684 | 0.4771 | 0.7959 | 1.4925 | 1.4697 | 1.1489 |
| Gorai.010G231900 | 1.6017 | 1.7683 | 1.7214 | 1.6884 | 1.7059 | 1.6240 | 1.3612 | 1.7571 | 1.6339 | 1.7969 |
| Gorai.010G232000 | 0.0000 | 0.0000 | 0.0000 | 0.0000 | 0.0000 | 0.0000 | 0.0000 | 0.0000 | 0.0000 | 0.0000 |
| Gorai.010G232100 | 0.0000 | 0.0000 | 0.0000 | 0.0000 | 0.0000 | 0.0000 | 0.0000 | 0.0000 | 0.0000 | 0.0000 |
| Gorai.010G232200 | 0.0000 | 0.0000 | 0.0000 | 0.0000 | -0.6778 | 1.2079 | 0.0000 | 0.0000 | 0.0000 | 0.0000 |
| Gorai.010G232300 | 0.0000 | 0.0000 | 0.0000 | -1.0458 | 1.4836 | 1.6346 | 0.0000 | -1.0969 | -0.8539 | 0.0000 |
| Gorai.010G250000 | 0.8609 | 0.8615 | 0.8943 | 0.8831 | 1.0278 | 1.1430 | 0.9243 | 0.8463 | 0.7738 | 0.8482 |
| Gorai.010G250100 | 0.1367 | 0.4871 | -0.6198 | 1.6261 | 1.8895 | 2.0669 | -1.0458 | -0.1549 | 0.0000 | 0.1106 |
| Gorai.010G250200 | 0.8561 | 0.9605 | 0.9258 | 1.0531 | 1.4128 | 1.8440 | 1.0477 | 1.2084 | 1.2238 | 1.1206 |
| Gorai.010G250300 | 1.8785 | 1.6673 | 2.0570 | 1.4454 | 0.7980 | 0.5911 | 1.6265 | 1.7775 | 1.7207 | 1.4912 |
| Gorai.010G250400 | 0.0000 | 0.0000 | 0.0000 | 0.0000 | 0.0000 | 0.0000 | 0.0000 | 0.0000 | 0.0000 | 0.0000 |
| Gorai.010G250500 | 0.4048 | 0.5752 | 0.6415 | 0.6646 | 0.8062 | 0.9926 | 1.0546 | 0.5092 | 0.5821 | 0.3365 |
| Gorai.010G250600 | -0.9208 | 0.0719 | 0.0000 | 0.0792 | 0.1732 | 0.2900 | 1.0095 | 1.1464 | 0.2014 | 0.0000 |
| Gorai.010G250700 | 0.0000 | 0.0000 | -1.5229 | -1.2218 | 0.0000 | 0.0000 | 0.0000 | -1.2218 | -0.9586 | 0.0000 |
| Gorai.010G250800 | 0.9217 | 0.9523 | 1.0095 | 1.0737 | 0.8716 | 1.0660 | 1.0481 | 0.5911 | 0.8149 | -0.1427 |
| Gorai.010G250900 | 0.0000 | 0.0000 | 0.0000 | 0.0000 | 0.0000 | 0.0000 | 0.0000 | 0.0000 | 0.0000 | 0.0000 |
| Gorai.010G251900 | 0.0000 | 0.0000 | 0.0000 | 0.0000 | 0.0000 | 0.0000 | 0.0000 | 0.0000 | 0.0000 | 0.0000 |
| Gorai.010G252000 | 0.0000 | 0.0000 | 0.0000 | 0.0000 | 0.0000 | 0.0000 | 0.0000 | 0.0000 | 0.0000 | 0.0000 |
| Gorai.010G252100 | 1.0149 | 0.8109 | 0.8261 | 0.8089 | 0.6454 | 0.6875 | 0.2742 | 0.9908 | 1.0924 | 0.7627 |
| Gorai.010G252200 | 1.0035 | 0.8609 | 1.1072 | 1.8489 | 1.6061 | 1.2345 | 1.7964 | 1.7218 | 1.9462 | 0.9863 |
| Gorai.010G252300 | -0.3372 | 0.0000 | -0.4685 | -1.2218 | 0.0000 | 0.0000 | -0.4318 | 0.4983 | 0.4871 | 0.4393 |
| Gorai.010G252400 | -1.0969 | 0.0000 | -0.1675 | -0.2840 | -0.2147 | -0.3188 | -1.0969 | 0.1399 | 0.2833 | -0.1938 |
| Gorai.010G252500 | 0.8261 | 0.3365 | -0.0458 | 0.0000 | -0.1079 | -0.2291 | 1.0056 | 1.1532 | 0.0000 | 0.4886 |
| Gorai.010G252600 | -0.1675 | 0.0000 | -1.3010 | -1.2218 | 0.0000 | 0.3802 | 1.2000 | 0.2810 | 0.4456 | -0.1192 |
| Gorai.010G252700 | 0.0000 | 0.0000 | -2.0000 | 0.0000 | 0.0000 | 0.0000 | 0.5315 | 0.4942 | 0.3541 | 0.5441 |
| Gorai.010G253100 | 0.0000 | 0.0000 | -0.6778 | 0.0000 | -0.0555 | -1.6990 | 1.1909 | 0.9699 | 0.0000 | 0.0000 |
| Gorai.010G253200 | -0.6990 | 0.0000 | -0.3279 | -0.7696 | -0.5850 | -0.5850 | -0.3010 | -0.4815 | -0.5086 | -0.5086 |
| Gorai.010G253300 | 0.0000 | 0.0000 | -0.8239 | 0.0000 | -1.1549 | 0.0000 | -0.1024 | -1.1549 | -0.8539 | 0.0000 |
| Gorai.010G253400 | 1.1915 | 0.5065 | 1.0000 | 0.1875 | 0.5302 | 0.7348 | 1.3755 | 1.5020 | 1.2172 | 1.5497 |
| Gorai.010G253500 | 0.0000 | 0.0000 | 0.0000 | 0.0000 | -1.6990 | 0.0000 | 0.0000 | 0.0000 | 0.0000 | 0.0000 |
| Gorai.010G253600 | 0.5289 | 0.8675 | 0.6222 | 0.4728 | 0.4654 | 0.5328 | 0.6212 | 1.0920 | 0.8055 | 1.2156 |
| Gorai.010G256400 | -0.0809 | 0.0000 | -0.3188 | -0.8239 | -0.0555 | -0.4202 | 1.8958 | 1.2700 | 0.0000 | 0.0000 |
| Gorai.010G256500 | 0.0000 | 0.0000 | 0.0000 | -0.6778 | 0.0000 | -0.7447 | 0.5119 | 0.7612 | -0.0915 | 0.0000 |
| Gorai.010G256600 | -0.5376 | 0.2227 | -0.3979 | -0.6990 | -0.3098 | -0.4202 | 0.5623 | 0.2601 | 0.5302 | 0.0000 |
| Gorai.010G256700 | 0.6739 | 0.6263 | 0.4548 | 0.3032 | 0.5670 | 0.3617 | 0.8401 | 0.6042 | -0.0706 | 0.8162 |
| Gorai.010G256800 | 1.6830 | 1.6792 | 1.5359 | 1.3930 | 1.3149 | 1.2049 | 1.4765 | 1.3272 | 1.4121 | 1.1323 |
| Gorai.010G256900 | 0.0000 | 0.0000 | 0.0000 | -1.5229 | -1.3979 | -1.2218 | 0.8854 | 1.0770 | 0.0000 | -0.2147 |
| Gorai.011G000700 | 0.1038 | 0.3927 | 0.4378 | 0.5105 | 0.4742 | 0.4048 | 0.4800 | -0.2518 | -0.5528 | -0.1192 |
| Gorai.011G000800 | 0.0000 | 0.0000 | 0.0000 | 0.0000 | 0.0000 | 0.0000 | 2.0686 | 2.0084 | 0.0000 | 0.0000 |
| Gorai.011G000900 | 0.0000 | -1.3979 | -1.5229 | -1.0000 | -0.7212 | -1.0000 | -0.7447 | 0.0000 | 0.0000 | 0.0000 |
| Gorai.011G001000 | 0.6964 | 0.7672 | 1.1176 | 0.9489 | 1.2415 | 1.2106 | 1.0488 | 1.7657 | 0.6866 | 2.0106 |
| Gorai.011G001100 | 0.1875 | 0.0000 | 0.0000 | -0.3372 | 0.0000 | -0.0915 | 1.1717 | 1.0318 | -0.5686 | 0.0000 |
| Gorai.011G001500 | 0.2553 | 0.2148 | 1.0128 | 1.0224 | 0.8000 | 0.5922 | 1.2499 | 0.9854 | 0.7853 | 1.0704 |
| Gorai.011G001600 | -0.5850 | 0.0000 | -0.4815 | -0.3372 | -0.0706 | -0.1805 | -0.9208 | 0.0000 | 0.0000 | 0.0000 |
| Gorai.011G001700 | 0.5740 | 0.7210 | 0.2989 | 0.4031 | 0.5821 | 0.5441 | 1.1951 | 1.2172 | 0.7825 | 0.9042 |
| Gorai.011G001800 | -1.2218 | 0.0000 | -1.3010 | -1.6990 | -1.6990 | -1.6990 | -0.8239 | -0.8239 | -0.5528 | 0.0000 |
| Gorai.011G001900 | 0.0000 | 0.0000 | 0.0000 | 0.0000 | 0.0000 | 0.0000 | 0.0000 | 0.0000 | 0.0000 | 0.0000 |
| Gorai.011G002000 | -0.5850 | -0.3468 | -1.0000 | -0.5376 | 0.0000 | -0.5376 | 0.9330 | 1.0370 | 0.0000 | 0.0000 |
| Gorai.011G002100 | 0.4487 | 0.6149 | 0.7067 | 0.8041 | 0.7597 | 0.7574 | 0.8954 | 1.1755 | 1.0824 | 1.1940 |
| Gorai.011G002200 | 0.0000 | 0.0000 | 0.0000 | -0.6021 | 0.2672 | -0.1427 | 0.0000 | 0.0000 | 0.0000 | 0.0000 |
| Gorai.011G008100 | 0.0000 | 0.7896 | -0.1549 | 0.2718 | 0.2455 | 0.3222 | 0.4698 | 0.4216 | -0.0223 | 0.4564 |
| Gorai.011G008200 | -0.2147 | -0.1249 | 0.1875 | 0.0453 | 0.6085 | 0.4362 | 0.3483 | 0.4969 | 0.3820 | 0.5302 |
| Gorai.011G008300 | 0.0000 | -0.5086 | -0.3979 | -0.3372 | -0.3188 | -0.1739 | 1.1517 | 1.4998 | 0.0000 | 0.0000 |
| Gorai.011G008400 | 0.5514 | -0.8861 | -0.0706 | -0.5686 | -0.2596 | 0.0374 | -0.4437 | 0.0212 | -0.0915 | 0.0531 |
| Gorai.011G008500 | -0.7696 | 0.0000 | 0.1072 | 0.0000 | 0.2504 | -0.0757 | 0.1673 | 0.6375 | 0.0000 | 0.3541 |
| Gorai.011G008600 | 1.5736 | 2.0059 | 1.9165 | 1.7431 | 1.7833 | 2.1760 | 1.7980 | 1.6961 | 1.0469 | 1.8949 |
| Gorai.011G008700 | 0.4116 | 0.8293 | 0.4472 | 0.5224 | 0.4099 | 1.1139 | 0.9786 | 1.0785 | 0.9750 | 1.1041 |
| Gorai.011G008800 | 0.6010 | -0.0809 | 0.1903 | 0.7868 | -0.0223 | -0.0088 | 0.5185 | 0.3284 | 0.4440 | 0.0682 |
| Gorai.011G013600 | -1.5229 | 0.8331 | 0.5866 | 0.4533 | 0.5340 | 0.4440 | 0.4624 | 0.6902 | 0.5599 | 0.0000 |
| Gorai.011G013700 | -0.6576 | 0.2695 | -0.2596 | 0.7067 | 0.0000 | -0.0044 | 0.0000 | 1.0878 | 1.2114 | 0.8089 |
| Gorai.011G013800 | -1.1549 | 0.3692 | 0.7846 | 0.7101 | -0.0655 | 0.4265 | -0.1308 | 1.4524 | 1.4115 | 1.3508 |
| Gorai.011G013900 | -0.3010 | -0.2757 | -0.6383 | -0.2218 | 0.0000 | -0.9208 | 0.0000 | -0.7959 | -0.5376 | 0.0000 |
| Gorai.011G014000 | -0.2366 | 0.1072 | 0.1139 | 0.0828 | 0.0792 | 0.0414 | 0.0453 | 0.2900 | 0.3711 | 0.1004 |
| Gorai.011G015800 | 1.6414 | 0.3909 | 1.0418 | 0.8344 | 0.4728 | 0.2765 | -0.1675 | 1.3156 | 0.7839 | 1.5056 |
| Gorai.011G015900 | -1.3979 | -1.1549 | -1.3979 | -0.5528 | -2.0000 | 0.0000 | -1.3979 | -1.6990 | -1.5229 | 0.0000 |
| Gorai.011G016000 | 0.0000 | 0.0000 | 0.0000 | 0.0000 | 0.0000 | 0.0000 | 0.8627 | 0.7076 | 0.4698 | 0.8102 |
| Gorai.011G016100 | -1.2218 | 0.4232 | -0.7959 | -0.0915 | 0.0899 | 0.2810 | 0.2122 | -0.3565 | -0.2291 | -0.6576 |
| Gorai.011G016200 | -1.0458 | -0.9208 | -0.2676 | -0.2596 | -1.3010 | -2.0000 | -0.2366 | 1.0803 | 0.0000 | 0.0000 |
| Gorai.011G016300 | -0.3372 | 0.0000 | -1.1549 | -0.4685 | 0.0000 | 0.0000 | -0.4202 | 0.0000 | 0.0000 | 0.0000 |
| Gorai.011G016400 | 0.1461 | 1.2201 | 0.5378 | 0.4362 | 0.3838 | -0.2924 | 0.5263 | 0.5453 | 0.2201 | 0.6830 |
| Gorai.011G016500 | -0.0809 | -0.2840 | -0.3979 | 0.6385 | 1.6081 | 0.8432 | -0.2147 | 0.3365 | 0.1903 | 0.3909 |
| Gorai.011G016600 | 0.0000 | -1.5229 | -0.3010 | 0.0719 | 1.8119 | 1.5314 | 2.7241 | 2.8976 | 2.8542 | 2.7601 |
| Gorai.011G022100 | 0.0000 | -2.0000 | 0.0000 | 0.0000 | 0.0000 | 0.0000 | 1.2485 | 1.4315 | 0.0000 | 0.0000 |
| Gorai.011G022200 | 0.5465 | 0.3692 | 0.3324 | 0.2122 | 0.6503 | 0.3139 | 0.9576 | 1.4214 | 0.6928 | 1.0030 |
| Gorai.011G022300 | 0.0000 | 0.0000 | 0.0000 | -1.3979 | 0.0000 | -1.3979 | -0.5686 | -1.2218 | -0.9586 | 0.0000 |
| Gorai.011G022400 | 0.1492 | 0.5877 | 0.7267 | 0.7701 | 0.7332 | 0.6435 | 0.6990 | 0.8451 | 0.7612 | 0.6444 |
| Gorai.011G022500 | 0.0000 | 0.7380 | 0.6571 | 0.7767 | 1.0233 | 0.7723 | 0.8162 | 0.4166 | 0.2355 | 0.1106 |
| Gorai.011G023000 | 0.2788 | -0.7696 | -0.9208 | -1.6990 | -1.0969 | -0.9208 | -1.6990 | -0.6778 | 0.0000 | -0.4202 |
| Gorai.011G023100 | 0.7275 | 0.5092 | 0.7316 | 0.6955 | 0.5119 | 0.2648 | 0.7218 | 0.9581 | 0.7118 | 1.0652 |
| Gorai.011G023200 | 1.2718 | 0.9159 | 1.2648 | 1.1967 | 1.1436 | 0.6739 | 1.5252 | 1.1818 | 1.1738 | 1.1216 |
| Gorai.011G023300 | 0.5611 | 0.9063 | 1.0842 | 1.2019 | 1.4928 | 1.0730 | 0.9652 | 0.8482 | 0.7657 | 0.8585 |
| Gorai.011G023400 | 0.0000 | -1.2218 | 0.0000 | -1.5229 | 0.0000 | -0.2007 | 0.8710 | -0.8861 | -0.6198 | 0.0000 |
| Gorai.011G023500 | 1.4325 | 1.2516 | 1.6072 | 1.5047 | 1.6342 | 1.3758 | 1.7342 | 1.8139 | 1.8298 | 1.7253 |
| Gorai.011G023600 | -0.3010 | 0.4728 | 0.5623 | 1.3886 | 1.8820 | 1.8085 | 1.1926 | 0.6294 | 0.2148 | 0.7938 |
| Gorai.011G023700 | 0.1106 | 0.2967 | 0.3243 | 0.1644 | 0.3802 | 0.2380 | 0.6345 | -0.1192 | 0.1492 | 0.0000 |
| Gorai.011G023800 | 0.0000 | 0.0000 | 0.0000 | 0.0000 | -1.0458 | 0.0000 | 0.4133 | 0.5599 | 0.6010 | 0.4362 |
| Gorai.011G029400 | 0.0000 | 0.0000 | -0.2147 | -0.6576 | 0.0000 | 0.0000 | 2.0661 | 0.7839 | 0.8837 | 0.5587 |
| Gorai.011G029500 | 0.0000 | 0.0000 | 0.0000 | 0.0000 | 0.0000 | 0.0000 | 0.0000 | 0.0000 | 0.0000 | 0.0000 |
| Gorai.011G029600 | 0.8854 | 0.0000 | 0.4533 | -0.9208 | -0.0655 | -0.7959 | 0.5821 | 1.0017 | 0.4548 | 0.0000 |
| Gorai.011G029700 | 0.4166 | 0.4362 | 0.6284 | 0.8136 | 0.6532 | 1.6124 | 1.0531 | 2.1558 | 2.1844 | 2.0504 |
| Gorai.011G035300 | 0.0000 | 0.0000 | 0.0000 | -1.6990 | 0.0000 | 0.0000 | 0.0000 | 0.0000 | 0.0000 | 0.0000 |
| Gorai.011G035400 | 2.8110 | 0.5011 | 2.0450 | 1.9533 | 1.9000 | 1.3497 | 1.3278 | 1.7689 | 1.5145 | 1.8778 |
| Gorai.011G035500 | 2.1130 | 1.8174 | 1.9803 | 1.3572 | 1.2320 | 0.8407 | 0.0000 | 2.2727 | 2.0725 | 1.0550 |
| Gorai.011G044400 | 1.7683 | 1.6328 | 1.8460 | 1.8308 | 1.7343 | 1.9899 | 1.7022 | 1.9625 | 2.1190 | 1.5893 |
| Gorai.011G044500 | 0.3838 | -0.4318 | 0.2856 | 0.2625 | 0.4928 | 0.4487 | 0.4843 | 0.2577 | 0.2480 | 0.2014 |
| Gorai.011G044600 | -0.0706 | -0.2147 | -0.4437 | -0.1805 | -0.4437 | -0.1192 | -1.1549 | 0.0000 | 0.0000 | 0.0000 |
| Gorai.011G044700 | 0.0000 | 0.0000 | 0.0000 | 0.0000 | -1.0000 | 0.0000 | 0.0531 | -0.6198 | -0.3372 | 0.0000 |
| Gorai.011G044800 | 0.0000 | 0.0000 | 0.0000 | 0.0000 | 0.0000 | 0.0000 | 1.2418 | 0.0000 | 0.0000 | 0.0000 |
| Gorai.011G044900 | 0.5966 | 0.8376 | 0.8854 | 0.9294 | 1.0149 | 1.1361 | 0.9917 | 0.8704 | 0.7782 | 0.8876 |
| Gorai.011G045000 | 1.4444 | 1.4939 | 1.1455 | 0.9921 | 0.9699 | 0.6990 | 1.3442 | 1.4794 | 1.4002 | 1.4765 |
| Gorai.011G045100 | -2.0000 | 0.0000 | 0.0000 | 0.0000 | -0.6778 | 0.0000 | 0.9232 | 0.0969 | -0.1675 | 0.0000 |
| Gorai.011G054700 | 0.0000 | 0.0000 | -1.2218 | 0.0000 | -1.1549 | -1.1549 | 0.4232 | 0.0000 | 0.0000 | 0.0000 |
| Gorai.011G054800 | -0.0706 | 1.6381 | 0.2765 | 0.1072 | -0.3979 | -0.2924 | 0.0934 | 0.4814 | 0.3324 | 0.5378 |
| Gorai.011G054900 | 1.6633 | 1.4894 | 1.4228 | 1.3649 | 1.4099 | 1.2204 | 0.8122 | 1.0795 | 0.8814 | 1.1638 |
| Gorai.011G055000 | 0.0000 | 0.0000 | -0.6198 | 0.4742 | 1.1623 | 0.6884 | 0.1430 | 0.4579 | 0.6513 | -0.0605 |
| Gorai.011G055100 | 0.0000 | 0.0000 | -1.5229 | -0.5229 | -0.1805 | -0.5086 | 0.0000 | -0.8539 | -0.5850 | 0.0000 |
| Gorai.011G057100 | -2.0000 | 0.0000 | 0.0000 | 0.0000 | 0.0000 | 0.0000 | 2.0999 | 0.8388 | 0.0000 | 0.0000 |
| Gorai.011G057200 | 0.5237 | 0.5635 | 0.7316 | 0.5159 | 0.5855 | 0.6998 | 0.8779 | 0.9814 | 0.1614 | 0.0645 |
| Gorai.011G057300 | 0.0000 | 0.0000 | -1.3010 | -1.3010 | 0.0000 | 0.0000 | -2.0000 | 0.0000 | 0.0000 | 0.0000 |
| Gorai.011G057400 | 0.0000 | 0.0000 | -0.9208 | 0.0000 | 0.3345 | -0.3665 | 1.0314 | -1.6990 | -2.0000 | 0.0000 |
| Gorai.011G057500 | 1.5070 | 1.3019 | 1.6933 | 1.7924 | 1.8295 | 1.6823 | 1.3506 | 1.7987 | 1.7134 | 1.7756 |
| Gorai.011G057600 | 0.0000 | 0.0000 | -0.1024 | 0.9269 | 0.8762 | 0.7796 | 1.0774 | 1.3079 | 0.0000 | 0.0000 |
| Gorai.011G060200 | 0.7168 | 0.4409 | 0.8344 | 0.8494 | 1.1239 | 1.1772 | 0.5899 | -0.1675 | -0.5686 | -0.0088 |
| Gorai.011G060300 | 0.6911 | 0.6212 | 0.6222 | 0.6425 | 0.8363 | 0.9117 | 1.0233 | 0.5132 | 0.0969 | 0.5539 |
| Gorai.011G061600 | 0.0000 | 0.0000 | 0.0000 | 0.0000 | 0.0000 | 0.0000 | 0.0000 | 0.0000 | 0.0000 | 0.0000 |
| Gorai.011G061700 | 1.4506 | 1.0394 | 1.4309 | 1.2603 | 1.3579 | 1.0781 | 1.1813 | 1.5690 | 1.7327 | 1.1045 |
| Gorai.011G061800 | 0.8791 | 1.0039 | 0.8407 | 0.6884 | 0.5378 | 0.6646 | 0.8280 | 1.2084 | 1.1248 | 1.2191 |
| Gorai.011G065600 | 0.8351 | -0.1871 | -0.4949 | -0.8861 | -1.2218 | -0.7212 | 2.5332 | -0.4949 | -0.2218 | 0.0000 |
| Gorai.011G065700 | 0.8156 | 0.7024 | 1.0224 | 1.0554 | 1.3081 | 1.8081 | 1.3139 | 0.7868 | 0.7024 | 0.7860 |
| Gorai.011G065800 | 0.0000 | 0.0000 | 0.0000 | 0.0000 | 0.0000 | 0.0000 | -1.3010 | 0.0000 | 0.0000 | 0.0000 |
| Gorai.011G065900 | 0.0000 | 0.0000 | 0.0000 | 0.0000 | 0.0000 | 0.0000 | -0.5376 | 0.0000 | 0.0000 | 0.0000 |
| Gorai.011G066000 | 0.6405 | 0.4014 | 0.7774 | 0.8927 | 0.6998 | 0.8344 | 0.9227 | 0.9518 | 0.0000 | 0.0000 |
| Gorai.011G066100 | 1.2410 | 1.4633 | 1.4425 | 1.2378 | 1.4084 | 0.9978 | 1.5465 | 1.5711 | 0.7839 | 0.8129 |
| Gorai.011G068100 | 0.0755 | 0.1206 | -0.1675 | -0.1739 | 0.4594 | 0.9440 | 0.8686 | 0.7980 | 0.5378 | 0.0000 |
| Gorai.011G068200 | 1.9276 | 2.2526 | 1.9820 | 1.8802 | 2.0204 | 1.7750 | 1.8295 | 2.0809 | 2.0963 | 1.9927 |
| Gorai.011G068300 | 1.6155 | -0.8539 | 1.5101 | -0.0605 | 0.7959 | 0.1239 | -0.1739 | -0.9586 | -0.6778 | 0.0000 |
| Gorai.011G068400 | 0.1903 | 0.0374 | -0.0458 | 0.0969 | 0.4425 | 1.2109 | 1.0948 | 1.0477 | 1.2425 | 0.5132 |
| Gorai.011G068700 | -0.4318 | -0.2676 | -0.0269 | 0.0492 | 0.1790 | 0.0719 | -0.0555 | -0.1739 | -0.5686 | -0.0177 |
| Gorai.011G068800 | 0.2718 | -0.2007 | 0.4393 | 0.7024 | 0.4983 | 1.0615 | 0.5911 | 0.6232 | 0.0000 | 0.0000 |
| Gorai.011G068900 | 0.2227 | 0.4533 | 0.4843 | 0.2810 | 0.2648 | 0.4249 | 0.0531 | 0.5024 | 0.6085 | 0.2648 |
| Gorai.011G069000 | 1.7104 | 1.4655 | 0.3560 | 0.9518 | 0.9581 | 0.9079 | 2.4001 | 2.1650 | 0.0000 | 0.0000 |
| Gorai.011G069100 | 0.1761 | 0.0000 | 0.4579 | 0.6335 | 0.3181 | 0.7952 | 1.4884 | 0.4579 | 0.4814 | 0.1673 |
| Gorai.011G069200 | 0.0000 | 0.0000 | 0.0000 | -0.6021 | -0.0458 | -0.4949 | -1.6990 | 0.0000 | 0.0000 | 0.0000 |
| Gorai.011G078100 | 0.0000 | 0.0000 | -2.0000 | -0.8539 | 0.0000 | 0.0000 | 0.6180 | 0.7993 | 0.0000 | 0.0000 |
| Gorai.011G078200 | 0.0128 | 0.7679 | 0.2601 | 0.2788 | 0.4871 | 0.6803 | 1.1021 | 1.1261 | 0.6232 | 0.6637 |
| Gorai.011G078300 | 0.9773 | 1.2627 | 0.9708 | 0.8802 | 0.9666 | 0.8048 | 0.9217 | 1.0799 | 0.9741 | 1.1075 |
| Gorai.011G089300 | 2.4387 | 2.4085 | 2.4684 | 2.4746 | 2.4227 | 2.0217 | 1.8369 | 1.9066 | 1.7886 | 1.9428 |
| Gorai.011G089400 | 0.0000 | 0.0000 | -0.2366 | 0.0000 | 0.0000 | 0.0000 | 1.0000 | 1.4035 | 0.0682 | 0.0000 |
| Gorai.011G098600 | 0.0000 | 0.0000 | 0.0000 | -0.5086 | -1.1549 | 0.0000 | 0.0000 | -1.3010 | -1.0458 | 0.0000 |
| Gorai.011G098700 | 0.3598 | 0.2201 | 0.4346 | 0.3444 | 0.4814 | 0.2430 | 0.0645 | 0.0000 | 0.0000 | 0.0000 |
| Gorai.011G098800 | 0.7701 | 1.0220 | 0.8506 | 0.7782 | 1.0892 | 1.0874 | 1.1433 | 1.1031 | 0.5587 | 0.9675 |
| Gorai.011G098900 | 0.0000 | 0.0000 | 0.0000 | -0.2076 | 0.0000 | 0.0000 | 0.0000 | 0.0000 | 0.0000 | 0.0000 |
| Gorai.011G099000 | 0.2041 | 0.0000 | 0.3444 | 0.0000 | 0.0000 | 0.0000 | 0.0000 | 0.0000 | 0.0000 | 0.0000 |
| Gorai.011G099100 | 0.0000 | 0.0000 | 0.0000 | 0.0000 | 0.0000 | 0.0000 | -0.6021 | 0.0000 | 0.0000 | 0.0000 |
| Gorai.011G099200 | 2.5065 | 2.5756 | 2.5999 | 2.6356 | 2.5882 | 2.8424 | 2.5023 | 2.6896 | 2.7220 | 2.5792 |
| Gorai.011G101600 | -0.1675 | -0.1675 | -0.3468 | 0.1644 | -0.3665 | 0.0755 | -0.6383 | 0.0000 | 0.0000 | 0.0000 |
| Gorai.011G101700 | 0.2095 | -0.5229 | 0.4669 | -0.6198 | 0.0000 | 0.0000 | 1.0043 | 0.9299 | 1.0611 | 0.6335 |
| Gorai.011G102600 | 1.5247 | 1.3497 | 1.4476 | 1.3316 | 1.1290 | 1.2098 | 1.3081 | 1.6539 | 1.4371 | 1.6895 |
| Gorai.011G102700 | -0.2924 | -1.0458 | 0.0000 | -0.8861 | 0.5211 | -0.1024 | 1.2162 | 0.3160 | 0.3032 | 0.2625 |
| Gorai.011G105100 | 0.0000 | 0.0000 | -1.5229 | -0.1871 | 0.0000 | 0.0000 | -0.7959 | -1.0000 | -0.7212 | 0.0000 |
| Gorai.011G105200 | 0.0000 | -0.8539 | -1.6990 | 0.4346 | 0.0000 | 0.0000 | 0.0000 | 0.0000 | 0.0000 | 0.0000 |
| Gorai.011G105300 | -0.6198 | -0.0269 | -1.2218 | -0.4437 | 0.3222 | 0.0864 | 0.5944 | 0.1614 | 0.3636 | 0.0000 |
| Gorai.011G108400 | 0.3909 | -0.2840 | 0.5539 | 0.5453 | 0.5563 | 0.2355 | 0.7404 | 0.4548 | 0.6345 | -0.0044 |
| Gorai.011G108500 | 1.0090 | 1.1310 | 1.3572 | 0.7657 | 1.0726 | 1.6612 | 1.5207 | 1.2516 | 1.0580 | 1.2951 |
| Gorai.011G108600 | 0.0000 | 0.0000 | 0.0000 | 0.0000 | 0.0000 | 0.0000 | 0.0000 | 0.0000 | 0.0000 | 0.0000 |
| Gorai.011G108700 | 0.0000 | 0.0000 | 0.0000 | 0.0000 | 0.0000 | 0.0000 | 0.0000 | -1.0458 | -0.7696 | 0.0000 |
| Gorai.011G114300 | 1.7691 | 1.6515 | 1.8168 | 1.6877 | 1.7485 | 1.7912 | 2.2789 | 1.7519 | 1.6095 | 1.8043 |
| Gorai.011G114400 | 0.0000 | 0.0000 | 0.0000 | 0.0000 | -0.4437 | -1.3010 | -1.0969 | -0.9586 | -0.6990 | 0.0000 |
| Gorai.011G131000 | 0.2330 | 0.4298 | 0.5250 | 0.4472 | 0.1959 | 0.2095 | 1.2853 | 0.3909 | 0.5132 | 0.1139 |
| Gorai.011G135400 | -0.7212 | -0.3468 | 0.2014 | 0.6222 | 0.9652 | 0.6053 | 0.4533 | 0.2553 | 0.4249 | -0.1612 |
| Gorai.011G135500 | -0.0132 | 0.0000 | 0.2810 | 0.5647 | -0.1249 | 0.6096 | 0.8312 | 1.0107 | 0.5798 | 0.0000 |
| Gorai.011G135800 | 0.0000 | 0.0000 | 0.0000 | 0.0000 | 0.0000 | 0.0000 | 0.0000 | 0.0000 | 0.0000 | 0.0000 |
| Gorai.011G138600 | 0.1761 | 0.8414 | 0.1303 | 0.1038 | 0.5340 | -0.0862 | 0.9320 | 0.8681 | 0.6785 | 0.5185 |
| Gorai.011G138700 | 0.3345 | 0.5888 | 0.5611 | 0.5378 | 0.2504 | 0.3054 | 0.4771 | 0.6107 | 0.4518 | 0.6739 |
| Gorai.011G158300 | 0.8420 | 1.3670 | 0.9138 | 0.8351 | 1.0162 | 0.8814 | 0.6821 | 1.3870 | 1.2762 | 1.4183 |
| Gorai.011G158400 | 0.5888 | 0.7860 | 0.8825 | 0.8028 | 0.9934 | 0.7731 | 1.1973 | 0.8854 | 0.6675 | 0.9533 |
| Gorai.011G158900 | 1.0523 | 0.9175 | 1.2162 | 1.2831 | 1.1989 | 1.2289 | 1.1380 | 0.8494 | 0.9455 | 0.6325 |
| Gorai.011G159000 | 2.0794 | 1.8866 | 1.9415 | 2.0112 | 1.8311 | 2.0480 | 1.9039 | 1.7023 | 1.8568 | 1.3359 |
| Gorai.011G160100 | 0.0000 | -0.1805 | -0.4685 | -0.1612 | 0.0212 | 0.0000 | 1.0048 | 0.7267 | 0.0000 | 0.0000 |
| Gorai.011G162100 | -0.7959 | 0.2455 | -0.5376 | 0.2253 | 0.3856 | 0.3692 | -1.5229 | 0.5079 | 0.7067 | -0.0555 |
| Gorai.011G162200 | 0.0755 | 0.3118 | 0.0294 | -0.1367 | 0.1206 | 0.4757 | -0.2366 | 0.1903 | 0.1584 | 0.1523 |
| Gorai.011G162300 | 0.0000 | 0.0000 | 0.0000 | 0.0000 | 0.0000 | 0.0000 | 0.0000 | 0.0000 | 0.0000 | 0.0000 |
| Gorai.011G164100 | 0.0645 | -0.0458 | -0.0706 | 0.6294 | 1.1212 | 0.7738 | 1.2813 | -0.1308 | -1.0000 | 0.1038 |
| Gorai.011G164200 | 0.0000 | 0.0000 | -1.5229 | 0.0000 | -1.5229 | 0.0000 | 0.0000 | 0.0000 | 0.0000 | 0.0000 |
| Gorai.011G169700 | 0.0000 | 0.0000 | 0.0000 | -1.6990 | -1.1549 | -1.3010 | -0.2147 | -1.2218 | -0.9586 | 0.0000 |
| Gorai.011G169800 | 0.0000 | 0.0000 | 0.0000 | 0.0000 | 0.0000 | 0.0000 | 0.0000 | 0.0000 | 0.0000 | 0.0000 |
| Gorai.011G169900 | 0.0000 | 0.0000 | 0.0000 | 0.0000 | 0.0000 | 0.0000 | 0.0000 | 0.0000 | 0.0000 | 0.0000 |
| Gorai.011G173000 | 0.0000 | 0.0000 | -0.9208 | 0.0000 | 0.0000 | -0.2924 | 1.0438 | 0.6435 | -1.0000 | 0.9020 |
| Gorai.011G176700 | -0.9208 | -0.6383 | -1.2218 | -1.1549 | -0.6576 | -0.7212 | 0.0000 | 0.0000 | 0.0000 | 0.0000 |
| Gorai.011G176800 | 0.0000 | 0.0000 | 0.0000 | 0.0000 | -0.5850 | 0.0000 | 0.0000 | 0.0000 | 0.0000 | 0.0000 |
| Gorai.011G183400 | 1.1928 | 1.0370 | 1.2723 | 0.8222 | 1.0817 | 1.4293 | 1.6234 | 1.3927 | 1.0630 | 1.1335 |
| Gorai.011G183500 | 0.0000 | 0.0000 | 0.0000 | -1.3979 | -0.9208 | -1.1549 | -1.5229 | -0.6021 | 0.0000 | 0.0000 |
| Gorai.011G183600 | 0.0000 | 0.0000 | 0.0000 | 0.0000 | -1.0969 | 0.0000 | 0.0000 | 0.0000 | 0.0000 | 0.0000 |
| Gorai.011G183700 | 0.4216 | 0.0000 | 0.2227 | -0.9586 | -0.4559 | -1.0000 | 0.0000 | 0.0000 | 0.0000 | 0.0000 |
| Gorai.011G189100 | 0.0000 | 0.0000 | 0.0000 | 0.0000 | 0.0000 | 0.0000 | -0.5229 | -0.9586 | -0.6990 | 0.0000 |
| Gorai.011G189200 | 0.9122 | 0.6857 | 1.0542 | 1.0469 | 0.8976 | 1.2958 | 0.7945 | 0.8762 | 0.8549 | 0.8306 |
| Gorai.011G205600 | 0.0000 | 0.1703 | 0.0000 | -1.6990 | 0.0000 | -0.6021 | 1.0730 | 0.6599 | -0.2366 | 0.0792 |
| Gorai.011G205700 | -1.3010 | -1.3979 | -1.3979 | -0.7447 | -0.7959 | -1.5229 | -0.8239 | -1.5229 | -1.1549 | 0.0000 |
| Gorai.011G205800 | 0.9096 | 0.9143 | 0.9614 | 1.0906 | 1.2063 | 1.4504 | 1.3408 | 1.7908 | 1.3562 | 1.3826 |
| Gorai.011G205900 | 0.5635 | 0.6920 | 0.6580 | 0.4472 | 0.6794 | 0.3711 | 0.5966 | 0.8681 | 0.8041 | 0.8573 |
| Gorai.011G208800 | 0.0792 | 0.0000 | -0.9586 | 0.0000 | 0.2122 | -0.2757 | 0.0000 | 0.0000 | 0.0000 | 0.0000 |
| Gorai.011G208900 | 0.0000 | 0.0000 | -1.5229 | -0.3010 | 0.3711 | -0.0088 | -0.8239 | 0.0000 | 0.0000 | 0.0000 |
| Gorai.011G209500 | 0.0000 | 0.0000 | 0.0000 | 0.0000 | 0.0000 | 0.0000 | -0.1308 | 0.0000 | 0.0000 | 0.0000 |
| Gorai.011G215200 | 0.7889 | 1.9634 | 1.4444 | 1.6889 | 1.7550 | 1.4417 | 0.0000 | 0.3324 | 0.3997 | 0.1673 |
| Gorai.011G215300 | 0.4857 | 1.1608 | 0.6345 | 0.6010 | 0.6284 | 1.3168 | 1.4036 | 1.0026 | 0.5786 | 1.1514 |
| Gorai.011G215400 | 0.7931 | 0.8287 | 1.1024 | 0.7642 | 0.6693 | 0.3711 | 0.0334 | 0.1761 | 0.3874 | -0.4559 |
| Gorai.011G215500 | 0.8651 | 0.7952 | 1.2584 | 0.8887 | 0.6160 | 0.3032 | 0.5478 | -1.2218 | 0.0000 | -0.9586 |
| Gorai.011G215600 | -1.0000 | 0.0000 | -1.3010 | -0.5686 | -1.2218 | -1.5229 | -0.5229 | -0.4949 | -0.3372 | -0.8539 |
| Gorai.011G232400 | 0.0000 | 0.0000 | 0.0000 | -0.7212 | 0.0000 | -1.0000 | 0.8910 | 0.0000 | 0.0000 | 0.0000 |
| Gorai.011G232500 | 0.0000 | 0.0000 | -1.1549 | 0.0000 | 0.0000 | 0.0253 | 0.5263 | -0.1079 | 0.0000 | 0.1584 |
| Gorai.011G237900 | 0.0000 | 0.0000 | 0.0000 | 0.0000 | 0.0000 | 0.0000 | 1.2718 | 1.3168 | 0.0000 | 0.2430 |
| Gorai.011G266200 | 0.5821 | 0.5888 | 0.2765 | 0.0000 | 0.3892 | -0.1549 | 0.1303 | 1.0903 | 0.7566 | 1.2289 |
| Gorai.011G266300 | 0.9263 | 0.8215 | 1.2555 | 1.5794 | 1.1007 | 0.8865 | 1.1364 | 0.7868 | 0.9899 | 0.0000 |
| Gorai.011G266400 | 0.4548 | 0.0334 | -0.2924 | 0.0000 | 0.0828 | 0.5211 | 0.2967 | 0.5752 | 0.6454 | 0.4048 |
| Gorai.011G266500 | 0.0000 | 0.0374 | 0.1271 | -0.1487 | -0.9208 | 0.0000 | -0.2076 | 0.5119 | 0.4969 | 0.4579 |
| Gorai.011G266600 | 0.6821 | -0.6021 | 1.5907 | 0.7945 | 0.7966 | 0.2480 | 0.0000 | 0.5843 | -0.0362 | 0.7896 |
| Gorai.012G002900 | 0.8982 | 1.2000 | 0.8549 | 0.6937 | 0.8698 | 0.8663 | 0.7634 | 0.9969 | 0.5198 | 0.8621 |
| Gorai.012G003900 | 0.5145 | 0.0414 | 0.6684 | 0.7896 | 0.7672 | 0.7597 | 1.3404 | 0.7210 | 0.6857 | 0.6884 |
| Gorai.012G004000 | 0.2041 | 0.7474 | 0.8382 | 1.0237 | 0.0294 | 0.2900 | 1.2156 | 0.6484 | 0.4639 | 0.7259 |
| Gorai.012G004100 | 0.0000 | 0.0000 | -1.6990 | 0.0000 | -1.5229 | 0.0000 | 0.2355 | -1.3979 | -1.0969 | 0.0000 |
| Gorai.012G004200 | 1.2541 | 1.1544 | 1.3339 | 1.1818 | 0.7443 | 0.7388 | 1.0561 | 1.0237 | 0.6253 | 1.1271 |
| Gorai.012G013100 | 1.5798 | 1.6331 | 1.6372 | 1.5481 | 1.7376 | 1.6630 | 0.9727 | 1.1906 | 1.0278 | 1.2553 |
| Gorai.012G013200 | 2.4681 | 2.1341 | 2.0773 | 2.0333 | 2.0035 | 1.9557 | 1.8344 | 1.5847 | 0.0000 | -2.0000 |
| Gorai.012G013300 | -0.2840 | -0.0506 | 0.1903 | 0.2014 | 0.1875 | 0.8692 | 0.6335 | -0.1024 | 0.1239 | -0.8539 |
| Gorai.012G013400 | 2.0986 | 2.1073 | 2.3018 | 2.2935 | 2.1711 | 2.3438 | 2.4044 | 2.2035 | 2.1565 | 2.1827 |
| Gorai.012G013500 | -1.3010 | 0.9504 | -0.7447 | -0.0757 | -0.1192 | 0.0253 | 0.2625 | -0.0706 | -0.0410 | -0.1805 |
| Gorai.012G013600 | 0.4829 | 0.8820 | 0.8904 | 0.8848 | 1.2521 | 0.9513 | 1.5447 | 1.0022 | 0.8932 | 1.0318 |
| Gorai.012G013700 | 0.4914 | 0.2695 | 0.6712 | 0.8531 | 1.2370 | 1.0310 | 0.6522 | 0.1644 | -0.0044 | 0.2330 |
| Gorai.012G014000 | 0.0000 | 0.0000 | -1.2218 | -0.6990 | -0.3372 | -0.0915 | -0.4202 | -1.3979 | -1.1549 | 0.0000 |
| Gorai.012G014100 | 0.0000 | -0.8239 | -1.2218 | -0.2596 | 0.6128 | 0.0253 | 0.6314 | -1.2218 | -0.9586 | 0.0000 |
| Gorai.012G014200 | -0.0458 | -1.0969 | 0.2718 | -0.2007 | 0.0492 | -0.1675 | 1.8802 | 1.1075 | 0.9948 | 0.0000 |
| Gorai.012G014300 | 0.0000 | 0.0000 | 0.0000 | 0.0000 | -0.2757 | 0.0000 | 0.6884 | 0.0000 | 0.0000 | 0.0000 |
| Gorai.012G014400 | 1.1898 | -0.9586 | 0.0043 | 0.4048 | 0.4249 | -0.1079 | 1.6574 | 0.4456 | 0.6031 | 0.0000 |
| Gorai.012G015500 | 0.1875 | 0.5911 | 0.1703 | 0.0000 | -0.2596 | -0.2366 | 0.7300 | 0.8716 | 0.4683 | 1.0326 |
| Gorai.012G022300 | 1.3556 | 1.1483 | 1.3861 | 1.2307 | 1.2847 | 1.2410 | 1.2896 | 1.3508 | 1.2869 | 1.3450 |
| Gorai.012G022400 | 0.0000 | 0.0000 | 0.0000 | -0.4437 | 0.0000 | 0.0000 | 0.4669 | 0.7551 | 0.0000 | 0.0000 |
| Gorai.012G022500 | 0.0000 | 0.0000 | 0.0000 | 0.0000 | 0.0000 | 0.0000 | 0.7709 | 1.1421 | 0.0000 | 0.0000 |
| Gorai.012G023300 | -1.2218 | 0.5079 | -0.2366 | -0.4559 | -0.1079 | 0.3181 | 1.2127 | 1.3711 | 0.1584 | 0.0000 |
| Gorai.012G023400 | 0.0000 | 0.0000 | -1.5229 | 0.0000 | 0.0000 | 0.0000 | 0.0000 | 0.0000 | 0.0000 | 0.0000 |
| Gorai.012G023500 | 0.0000 | -1.1549 | -1.3979 | 0.0000 | 0.0000 | 0.0000 | 0.0000 | 0.0000 | 0.0000 | 0.0000 |
| Gorai.012G023600 | -0.4437 | -1.3010 | -0.9586 | -1.6990 | 0.0000 | -1.3010 | -1.1549 | -0.9208 | -0.6576 | 0.0000 |
| Gorai.012G023700 | 0.7356 | 0.7767 | 0.8927 | 0.8657 | 0.9036 | 1.0030 | 0.8831 | 0.9633 | 0.9079 | 0.9504 |
| Gorai.012G029200 | -0.0269 | -0.6778 | 0.0453 | -0.1192 | -0.2676 | -0.4949 | 0.4281 | -0.0915 | -0.0177 | -0.2676 |
| Gorai.012G029300 | 0.0000 | 0.0000 | 0.0000 | 0.0000 | 0.0000 | 0.0000 | -1.6990 | -1.5229 | -1.2218 | 0.0000 |
| Gorai.012G029400 | -0.1367 | 0.1553 | -0.0555 | 0.0969 | -0.1427 | -0.2676 | 0.0000 | 0.1271 | -0.2596 | 0.2810 |
| Gorai.012G029500 | -0.6198 | 0.0000 | -1.5229 | -1.0458 | 0.0000 | 0.0000 | -1.2218 | -0.9208 | -0.6576 | 0.0000 |
| Gorai.012G029600 | 1.6451 | 1.5850 | 1.6677 | 1.4667 | 1.5206 | 1.3766 | 1.6659 | 1.5032 | 1.5454 | 1.3789 |
| Gorai.012G029700 | 1.4109 | 1.2962 | 1.5691 | 1.4389 | 1.4403 | 1.6702 | 1.6801 | 1.3292 | 1.0980 | 1.4294 |
| Gorai.012G029800 | -1.0969 | 0.0000 | -1.0969 | -0.9586 | -0.4559 | -0.3768 | -0.8861 | -0.7447 | -0.8239 | 0.0000 |
| Gorai.012G034300 | 0.9133 | 0.7745 | 1.0245 | 1.0261 | 1.0004 | 0.9063 | 0.7875 | 1.2455 | 1.2785 | 1.1339 |
| Gorai.012G034400 | 0.0000 | 0.0000 | 0.0000 | 0.0000 | 0.0000 | 0.0000 | 1.0803 | 1.0990 | 0.0000 | 0.0000 |
| Gorai.012G034500 | 1.0741 | 1.1655 | 0.9053 | 0.9335 | 0.9004 | 0.9425 | 1.8494 | 1.5038 | 0.1173 | 1.1274 |
| Gorai.012G046500 | 0.3522 | 0.4548 | 1.0257 | 0.7308 | 0.9863 | 0.6128 | 0.9513 | 0.9238 | 0.8215 | 0.8319 |
| Gorai.012G046600 | 0.0000 | 0.0000 | 0.0000 | 0.0000 | 0.0000 | 0.0000 | -1.1549 | 0.0000 | 0.0000 | 0.0000 |
| Gorai.012G046700 | -1.6990 | 0.0000 | 0.5328 | 0.0212 | 0.0334 | 0.5011 | 1.5314 | 1.6628 | 0.0000 | 0.0000 |
| Gorai.012G047100 | -0.2076 | 0.0000 | -0.5528 | 0.0000 | -0.7447 | -0.0605 | 0.7267 | 0.9703 | 0.0755 | 0.3222 |
| Gorai.012G047200 | 0.0000 | -0.7696 | -1.1549 | 0.0000 | -0.2518 | 0.0000 | 0.6064 | 0.8954 | 0.3979 | 0.2989 |
| Gorai.012G047300 | -0.2676 | 0.0000 | 0.1818 | 0.0128 | -0.1739 | 0.3560 | 0.5119 | 0.6730 | 0.0792 | 0.0864 |
| Gorai.012G047400 | 0.0000 | 0.0000 | 0.0000 | 0.0000 | 0.0000 | 0.0000 | 0.0000 | 0.0000 | 0.0000 | 0.0000 |
| Gorai.012G056700 | 0.0000 | 0.0000 | 0.0000 | 0.0000 | 0.0000 | 0.0000 | 0.0000 | 0.0000 | 0.0000 | 0.0000 |
| Gorai.012G056800 | 0.0000 | 0.0000 | 0.0000 | 0.0000 | 0.0000 | 0.0000 | -0.1249 | -0.9586 | -0.6778 | 0.0000 |
| Gorai.012G056900 | 0.0000 | 0.0000 | 0.0000 | 0.0000 | 0.0000 | 0.0000 | -0.1871 | 0.0000 | 0.0000 | 0.0000 |
| Gorai.012G057700 | -0.0915 | 0.2878 | -0.6990 | 0.6830 | 0.6646 | 0.3075 | 0.1004 | 0.3979 | 0.3927 | -0.5686 |
| Gorai.012G057800 | 1.8626 | 1.6886 | 1.7217 | 1.7411 | 1.5878 | 1.2898 | 0.9605 | 1.2973 | 1.2251 | 1.2989 |
| Gorai.012G057900 | 0.3139 | 0.2430 | 0.0864 | 0.3243 | 0.6180 | 0.2380 | -0.0655 | 0.0000 | 0.0000 | 0.0000 |
| Gorai.012G058000 | 0.3118 | 0.9227 | 0.3766 | 0.2529 | 0.4456 | 0.1106 | 1.2156 | 0.2672 | 0.2148 | 0.2504 |
| Gorai.012G058100 | 0.0934 | 0.9731 | 0.6117 | 0.3522 | -0.2676 | -0.3872 | -0.3372 | -0.1427 | 0.0969 | -1.0969 |
| Gorai.012G060300 | 0.8657 | 0.7210 | 0.8122 | 0.8089 | 0.7980 | 0.7657 | 0.8543 | 0.3263 | 0.0755 | 0.4330 |
| Gorai.012G060400 | 0.0000 | 0.0000 | 0.0000 | 0.0000 | 0.0000 | 0.0000 | 0.0000 | 0.0000 | 0.0000 | 0.0000 |
| Gorai.012G060500 | -0.6990 | 1.2643 | -0.2757 | -0.9208 | 1.1109 | 0.6848 | 0.8274 | -0.3098 | 0.0000 | 0.0000 |
| Gorai.012G060600 | 0.0000 | 0.0000 | 0.0000 | 0.0000 | 0.0000 | 0.0000 | 1.0199 | 0.0000 | 0.0000 | 0.0000 |
| Gorai.012G068400 | 0.0000 | -0.4815 | -0.5850 | -0.7696 | 0.0000 | 0.0000 | -0.5376 | -0.3010 | -0.0315 | 0.0000 |
| Gorai.012G068500 | 0.0000 | 0.0000 | 0.0000 | 0.0000 | 0.0000 | 0.0000 | 0.4942 | -0.1367 | 0.0000 | 0.0000 |
| Gorai.012G068600 | 2.8288 | 1.7365 | 2.9830 | 2.6162 | 2.5559 | 1.9278 | 2.0760 | 2.9861 | 2.7331 | 3.0930 |
| Gorai.012G070200 | 0.7945 | 0.6294 | 0.6693 | 0.7657 | 0.5051 | 0.3636 | 0.5011 | 0.4654 | 0.6599 | -0.0655 |
| Gorai.012G070300 | 0.0000 | 0.6464 | 0.2068 | -0.4815 | 0.0000 | 0.0000 | 2.1581 | 2.0806 | 0.0000 | 0.0000 |
| Gorai.012G072700 | 0.0000 | 0.0000 | 0.0000 | 0.0000 | 0.0000 | 0.0000 | 0.0000 | -0.4318 | -0.1612 | 0.0000 |
| Gorai.012G072800 | 0.4031 | 0.2380 | 0.4409 | 0.4900 | 0.6021 | 0.2601 | 0.2227 | -0.2291 | -0.5086 | -0.1024 |
| Gorai.012G072900 | 0.0000 | 0.0000 | -0.8861 | -1.3010 | -0.8861 | -0.8861 | 0.0000 | 0.0000 | 0.0000 | 0.0000 |
| Gorai.012G073000 | 1.5785 | 1.5426 | 1.6450 | 1.8506 | 1.5576 | 1.8270 | 1.7105 | 2.0821 | 2.2945 | 1.4425 |
| Gorai.012G073100 | 0.7589 | 0.0000 | 0.5289 | 0.6284 | -0.1135 | 0.0607 | 0.5866 | 1.6253 | 0.9074 | 0.8312 |
| Gorai.012G073200 | 0.0000 | 0.0000 | 0.0000 | 0.0000 | 0.0000 | 0.0000 | 0.0000 | 0.0000 | 0.0000 | 0.0000 |
| Gorai.012G074600 | 0.0000 | 0.0000 | 0.0000 | 0.0000 | 0.0000 | 0.0000 | 0.7435 | 0.0086 | 0.2810 | 0.0000 |
| Gorai.012G082300 | 0.0000 | 0.0000 | 0.0000 | 0.0000 | 0.0000 | 0.0000 | 0.5502 | 0.0682 | 0.0000 | 0.0000 |
| Gorai.012G082400 | 0.0000 | 0.5465 | 0.0000 | -0.6990 | 0.0000 | 0.0000 | 0.9991 | 0.0000 | 0.0000 | 0.0000 |
| Gorai.012G082500 | 0.9952 | 1.1082 | 0.8344 | 1.0039 | 0.9133 | 1.1998 | 1.5019 | 1.8328 | 1.0249 | 1.8006 |
| Gorai.012G084200 | 0.0000 | -0.0223 | -0.6576 | -0.3979 | -0.3010 | -0.7212 | 0.7839 | 0.2279 | 0.0086 | 0.3243 |
| Gorai.012G084300 | 0.3579 | 0.3598 | 0.3598 | 0.3711 | 0.3927 | 0.3483 | -0.2596 | 0.2601 | 0.2577 | 0.1959 |
| Gorai.012G084400 | 0.6580 | 0.6263 | 0.7419 | 0.8716 | 0.8932 | 0.8136 | 0.8982 | 0.9465 | 0.3096 | 1.1538 |
| Gorai.012G084500 | 0.0000 | 0.0000 | 0.0000 | -0.4437 | 0.0000 | 0.0000 | 0.0000 | 0.0000 | 0.0000 | 0.0000 |
| Gorai.012G084700 | 0.3502 | 0.0000 | 0.3892 | -0.2840 | -1.0000 | -0.8239 | 0.0000 | 0.3118 | 0.0000 | 0.5763 |
| Gorai.012G089600 | -0.0706 | 0.3201 | 0.1072 | 0.2068 | 0.0792 | 0.3747 | 0.5340 | 0.1239 | 0.2304 | -0.1192 |
| Gorai.012G089700 | -2.0000 | -1.6990 | 0.1303 | 0.0253 | 0.0000 | -0.1367 | 1.6483 | 0.9528 | 0.0000 | 0.4624 |
| Gorai.012G089800 | -1.3979 | -0.0809 | 0.6138 | 0.2788 | 0.7589 | 1.2245 | 1.2686 | 0.9773 | 0.0000 | -0.6576 |
| Gorai.012G090700 | 1.3164 | 1.2728 | 1.4800 | 1.5168 | 1.4467 | 2.0187 | 1.5249 | 1.3257 | 1.0346 | 0.6160 |
| Gorai.012G090800 | 1.1418 | -0.2218 | 1.3224 | 1.4675 | 1.4211 | 0.8621 | 1.3170 | 1.4524 | 1.2878 | 1.0457 |
| Gorai.012G090900 | 0.7404 | 0.3820 | 0.4150 | -0.7959 | -0.7212 | 0.0043 | 1.6403 | 1.6684 | 0.9263 | 1.8887 |
| Gorai.012G091000 | 0.0000 | -0.7959 | -0.9208 | -0.3872 | -0.0458 | -0.7447 | 0.0000 | -0.1612 | -0.0757 | -0.3665 |
| Gorai.012G096800 | 1.6583 | 1.2284 | 1.7288 | 1.4673 | 0.9552 | 1.2693 | 1.7257 | 2.6697 | 2.8799 | 2.0445 |
| Gorai.012G110000 | -0.2441 | 0.0000 | -0.2147 | 0.0000 | 0.0000 | 0.0000 | -1.5229 | -0.5229 | -0.3979 | -0.7959 |
| Gorai.012G110100 | 0.2380 | -0.1739 | 0.1761 | -0.1308 | -0.9208 | -0.6021 | 1.3226 | -0.1192 | -0.8239 | 0.0569 |
| Gorai.012G110200 | 0.0000 | -1.3979 | 0.0000 | -0.3098 | -2.0000 | 0.0000 | 0.8109 | 0.5623 | -0.5086 | -0.4815 |
| Gorai.012G110300 | 0.0000 | 0.0000 | 0.0000 | 0.0334 | -0.3768 | 0.0000 | 1.5494 | 0.8848 | 0.0000 | 0.0000 |
| Gorai.012G118800 | 0.0755 | -0.2676 | -0.0458 | 0.3424 | -0.0362 | -0.3768 | 0.0000 | 0.5211 | 0.0682 | 0.6955 |
| Gorai.012G118900 | 0.0000 | 0.0000 | 0.0000 | 0.0000 | 0.0000 | 0.0000 | 0.0000 | 0.0000 | 0.0000 | 0.0000 |
| Gorai.012G124900 | 0.1004 | 0.0828 | 0.2553 | 0.4829 | 0.4814 | 0.6503 | 0.4698 | 0.6304 | 0.7218 | 0.4249 |
| Gorai.012G125000 | 0.0000 | 0.0000 | -1.3010 | 0.0000 | -0.5086 | 0.9128 | 2.9100 | 1.2238 | 0.0000 | 0.6274 |
| Gorai.012G125100 | -0.8861 | 0.0000 | -0.2676 | 0.0000 | -0.7696 | -1.3979 | 0.2695 | -0.6778 | -0.4089 | 0.0000 |
| Gorai.012G125200 | -0.7447 | 0.1523 | -0.5086 | -0.8239 | -0.8539 | -0.4815 | 0.3617 | 0.7612 | 0.7825 | -2.0000 |
| Gorai.012G131000 | -1.0000 | 0.0000 | -0.4089 | -0.8239 | 0.0000 | -1.1549 | 0.0128 | -0.9208 | -0.9208 | 0.0000 |
| Gorai.012G131100 | 0.2380 | 0.1903 | 0.2304 | 0.2695 | 0.1847 | 0.6395 | 0.5024 | 0.7664 | 0.7497 | 0.7160 |
| Gorai.012G131200 | 1.9684 | 1.9076 | 2.1882 | 2.0728 | 2.2663 | 2.4385 | 1.8710 | 1.2971 | 1.1355 | 1.3610 |
| Gorai.012G132800 | -1.2218 | 1.1014 | 0.0000 | 0.6474 | -0.1549 | -0.8239 | -1.2218 | 0.0000 | 0.0000 | 0.0000 |
| Gorai.012G134400 | 2.5075 | 2.3274 | 2.2041 | 2.1049 | 2.1124 | 2.0714 | 1.8394 | 1.5252 | 1.5296 | 1.4502 |
| Gorai.012G134500 | 0.0000 | -1.1549 | -0.4685 | -0.8861 | -0.3188 | -0.7959 | -0.6576 | 0.1335 | 0.1239 | 0.0755 |
| Gorai.012G134600 | -0.6383 | -0.4318 | -0.2218 | -0.2757 | -0.3565 | -0.1487 | -0.0555 | -0.2840 | -0.0132 | 0.0000 |
| Gorai.012G134700 | -0.0605 | 0.0000 | 0.0000 | -0.0088 | 0.0000 | -1.5229 | 0.9504 | -0.4437 | 0.0000 | 0.0000 |
| Gorai.012G134800 | 0.8116 | 0.9345 | 1.0208 | 0.8414 | 0.7396 | 1.0133 | 1.7366 | 0.7612 | 0.8169 | 0.6160 |
| Gorai.013G035300 | 0.0086 | 0.0000 | 0.0792 | -1.6990 | -0.6198 | 0.2148 | 1.2683 | 1.3406 | 0.0000 | 0.0000 |
| Gorai.013G035400 | 0.0000 | 0.0000 | -1.1549 | 0.0000 | 0.0000 | 0.0000 | -1.5229 | -1.3010 | -1.0458 | 0.0000 |
| Gorai.013G035500 | 1.0770 | 1.1274 | 1.2274 | 1.0508 | 0.7701 | 0.8376 | 1.5635 | 1.6049 | 1.4059 | 1.0697 |
| Gorai.013G035600 | 0.6201 | 0.3032 | 1.0689 | 0.8241 | 0.2856 | -0.0132 | 0.4116 | 0.7582 | 0.2788 | 0.9370 |
| Gorai.013G047800 | 0.0000 | 0.0000 | -1.2218 | -0.0915 | -0.1938 | -0.3372 | 1.7933 | 0.3263 | 0.5955 | 0.0000 |
| Gorai.013G047900 | -0.6778 | 0.0000 | 0.0000 | -1.6990 | -0.9208 | -0.2007 | 0.0043 | 0.1303 | 0.0000 | 0.0000 |
| Gorai.013G048000 | 0.7024 | 0.1173 | 0.8976 | 0.9340 | 1.1261 | 0.9284 | -0.6576 | 1.4021 | 1.5353 | 1.0149 |
| Gorai.013G072500 | 0.0000 | 0.0000 | 0.0000 | 0.0000 | -1.2218 | 0.0000 | 0.5490 | -1.0969 | -0.8861 | 0.0000 |
| Gorai.013G072600 | -0.0132 | -0.3468 | -0.6990 | -0.6383 | -0.1427 | -0.0706 | 0.3243 | 1.0298 | 1.2702 | 0.1139 |
| Gorai.013G073200 | 0.8470 | 0.6314 | 0.9666 | 1.0441 | 1.0864 | 0.9628 | 1.1781 | 1.0641 | 0.8727 | 1.1449 |
| Gorai.013G073300 | 0.0000 | 0.0000 | 0.0000 | 0.0000 | -0.4685 | -0.5850 | 0.0000 | 0.0000 | 0.0000 | 0.0000 |
| Gorai.013G085800 | -0.2076 | 1.1284 | -0.4685 | 0.1875 | 0.3927 | -0.0706 | -0.3188 | -0.4089 | 0.0000 | 0.0000 |
| Gorai.013G085900 | 0.0000 | 0.0000 | 0.0000 | -0.6778 | 0.0000 | 0.0000 | 0.3979 | 0.0212 | 0.0000 | 0.0000 |
| Gorai.013G086000 | 0.0000 | 0.0000 | -0.0362 | -0.3665 | -0.2924 | -0.4685 | 0.4082 | -0.0177 | -0.1805 | -0.1805 |
| Gorai.013G086100 | 0.2967 | 0.0000 | -0.0655 | -0.8239 | -0.6576 | -1.1549 | -0.8539 | 0.0000 | 0.0000 | 0.0000 |
| Gorai.013G088200 | 0.0000 | 0.0000 | -0.2596 | -0.0506 | 0.2577 | -0.0410 | -0.5086 | 0.1903 | -0.1079 | 0.3201 |
| Gorai.013G097700 | -0.7696 | -1.0969 | 0.0000 | 0.0000 | 0.0000 | 0.0000 | 0.0000 | -0.7447 | -0.4685 | 0.0000 |
| Gorai.013G112000 | 1.1358 | 1.3845 | 0.7474 | 0.6375 | -0.0269 | 0.3010 | 0.7987 | 1.5938 | 0.6395 | 1.8318 |
| Gorai.013G115000 | 0.0000 | 0.0000 | -0.4559 | -1.1549 | 0.0000 | 0.0000 | -0.6576 | 0.0000 | 0.0000 | 0.0000 |
| Gorai.013G115100 | 1.7445 | 2.0952 | 1.4136 | 1.4074 | 1.5293 | 1.3145 | 1.7047 | 1.4953 | 0.9908 | 1.6802 |
| Gorai.013G117500 | 0.6395 | 0.6972 | 0.2279 | 0.3160 | 0.0682 | 0.1644 | 0.0294 | 0.0000 | -0.1487 | 0.0531 |
| Gorai.013G117600 | 0.0000 | -0.4949 | -1.0969 | -0.5528 | -1.3979 | -0.5850 | -1.3010 | 0.0000 | 0.0000 | 0.0000 |
| Gorai.013G117700 | 0.4928 | 0.1614 | -0.9208 | 0.1959 | 0.1367 | 0.2253 | 0.2577 | 0.8287 | 0.3997 | 0.9965 |
| Gorai.013G117800 | 0.6646 | 0.0294 | -0.0757 | -0.6383 | -0.9586 | 0.0000 | 0.4116 | 0.5705 | 0.5038 | 0.5694 |
| Gorai.013G125500 | 0.0000 | -1.3979 | 0.0000 | 0.2405 | 0.0000 | 0.0000 | 1.7795 | 1.8180 | 0.2765 | -0.0506 |
| Gorai.013G125600 | 0.0000 | -0.5528 | -0.4685 | -0.5376 | -0.2757 | -0.1612 | 1.3477 | -0.1249 | -0.4318 | -2.0000 |
| Gorai.013G141200 | 0.0000 | 0.0000 | 0.0000 | 0.0000 | 0.0000 | 0.0000 | 0.0000 | 0.0000 | 0.0000 | 0.0000 |
| Gorai.013G141300 | 0.0000 | 0.9253 | -0.6383 | 0.0000 | -0.3098 | -2.0000 | 0.6665 | 1.7770 | 0.0000 | 0.0000 |
| Gorai.013G149400 | 0.0000 | -0.4318 | -1.1549 | 0.0000 | -1.2218 | -0.8861 | 0.0000 | 0.4669 | 0.1703 | 0.5944 |
| Gorai.013G149500 | 0.1903 | 0.4955 | 0.4116 | 0.2648 | 0.3483 | 0.4942 | 1.5089 | 1.0997 | 0.0000 | 0.0000 |
| Gorai.013G149600 | 0.4099 | 0.4742 | 0.6274 | 0.7459 | 0.5988 | 1.0069 | 1.0249 | 1.1495 | 1.2122 | 0.9943 |
| Gorai.013G149700 | 0.8567 | 0.8021 | 0.7789 | 0.9518 | 0.7931 | 0.9605 | 1.0704 | 0.9227 | 0.9633 | 0.8000 |
| Gorai.013G149800 | 0.0000 | 0.0000 | 0.0000 | 0.0000 | 0.0000 | 0.0000 | 0.0000 | 0.0000 | 0.0000 | 0.0000 |
| Gorai.013G149900 | -0.1675 | 0.6415 | 0.1271 | 0.3096 | -0.3768 | -0.1427 | 0.8129 | 0.6821 | 0.2455 | 0.7642 |
| Gorai.013G150000 | -0.8861 | -1.0969 | 0.0000 | 0.0000 | 0.0000 | 0.0000 | 0.8395 | 0.0000 | 0.0000 | 0.0000 |
| Gorai.013G157700 | 0.0000 | 0.0000 | 0.0000 | 0.0000 | 0.0000 | 0.0000 | 0.0000 | 0.0000 | 0.0000 | 0.0000 |
| Gorai.013G175100 | 0.0000 | -0.8239 | -0.9586 | -0.5850 | -0.1487 | -0.3979 | 1.1652 | 1.0777 | 0.0000 | -0.3372 |
| Gorai.013G175200 | 0.3139 | 0.7076 | 0.7135 | 1.1189 | 1.5524 | 1.1514 | 0.8727 | 0.3284 | -0.3188 | 0.0000 |
| Gorai.013G204400 | -0.4437 | 0.3483 | -0.3768 | -0.6198 | -0.0555 | 0.1847 | 1.1339 | 1.2781 | 1.3334 | 1.1339 |
| Gorai.013G204500 | 0.7348 | 0.7566 | 0.9542 | 0.9309 | 1.1159 | 1.2509 | 0.8609 | 0.6474 | 0.6794 | 0.5185 |
| Gorai.013G204600 | -0.5376 | 0.0000 | -0.6198 | -0.0757 | -0.6778 | -0.5229 | 0.9605 | 0.1732 | 0.1903 | 0.0828 |
| Gorai.013G204700 | 1.6738 | 1.7583 | 1.7671 | 1.5819 | 1.6430 | 1.4064 | 1.2127 | 0.8609 | 0.7853 | 0.7380 |
| Gorai.013G204800 | 0.0000 | 0.0000 | 0.0000 | 0.0000 | 0.0000 | 0.0000 | 0.0000 | 0.0000 | 0.0000 | 0.0000 |
| Gorai.013G204900 | 0.9175 | 1.2095 | 1.0934 | 0.8837 | 1.1000 | 1.0294 | 0.6911 | 0.8882 | 0.7924 | 0.9079 |
| Gorai.013G206000 | 0.0000 | 0.0000 | 0.0000 | 0.0000 | 0.0000 | 0.0000 | 1.0959 | -0.1675 | -0.3872 | 0.0000 |
| Gorai.013G206100 | 1.7460 | 1.5748 | 1.6851 | 1.7985 | 1.5897 | 2.0311 | 1.9100 | 1.4216 | 1.4280 | 0.0000 |
| Gorai.013G206200 | 0.0000 | 0.0000 | -0.0605 | 0.0000 | -0.0223 | -0.6383 | 0.6571 | 1.1501 | 1.2545 | 0.9090 |
| Gorai.013G208800 | 0.0000 | 0.0000 | 0.0000 | -0.7447 | -0.7696 | 0.0000 | -1.5229 | -0.5528 | -0.2840 | 0.0000 |
| Gorai.013G208900 | 0.0000 | 0.0000 | 0.3838 | -0.2676 | -0.0605 | -0.0410 | 1.8065 | 1.6731 | 1.6806 | 0.9269 |
| Gorai.013G227300 | 0.8982 | 0.8774 | 0.8802 | 1.0237 | 0.9773 | 0.8675 | 1.1858 | 1.0107 | 1.0686 | 0.8627 |
| Gorai.013G227400 | 0.0000 | 0.6739 | -1.0969 | -1.0458 | 0.0000 | 0.0000 | 0.0000 | -1.3979 | -1.1549 | 0.0000 |
| Gorai.013G227500 | 0.0000 | -0.7447 | 0.0000 | 0.0000 | -1.1549 | 0.0000 | -1.0969 | 0.0000 | 0.0000 | 0.0000 |
| Gorai.013G236300 | -0.1192 | -2.0000 | 0.3636 | -0.4949 | 0.5478 | 0.2945 | 1.0022 | 1.2191 | 0.9859 | 1.1501 |
| Gorai.013G236400 | 0.0719 | 0.1818 | -0.4318 | 0.0000 | -0.5528 | 0.0000 | 1.0888 | 1.1377 | 0.0000 | 0.0000 |
| Gorai.013G236500 | 0.0374 | 0.0000 | 0.2148 | 0.0086 | 0.2095 | 0.1173 | -0.0706 | -0.4437 | 0.0000 | 0.0000 |
| Gorai.013G236600 | 0.2068 | 0.4983 | 0.2625 | 0.1072 | 0.4814 | 0.6803 | 0.3674 | 0.3304 | 0.2945 | 0.2989 |
| Gorai.013G236700 | 1.2646 | 1.1166 | 0.9390 | 0.9722 | 1.0488 | 1.3558 | 1.2071 | 1.3139 | 1.1824 | 1.1729 |
| Gorai.013G236800 | -0.1249 | -1.0000 | -0.3872 | -1.0969 | -0.2291 | -0.6576 | -0.1135 | 0.2695 | -0.9208 | 0.5185 |
