# Supplementary material for: Simple Sequence Repeat (SSR) Genetic Linkage Map of D Genome Diploid Cotton Derived from an Interspecific Cross between Gossypium davidsonii and Gossypium klotzschianum
Source: Int J Mol Sci. 2018 Jan 11;19(1):204. doi: 10.3390/ijms19010204 (PMC5796153; doi:10.3390/ijms19010204)
Supplement: Supplementary file 1 [file ijms-19-00204-s001.zip › ijms-254865 final supplementary/Supplementary Table S5.docx]

Supplementary Table S5: Key genes with very high expression levels in mature leaf, ovules and various fibre developmental stages

| Gene ID | Gene Name | Description | KEGG Orthology | Pathway Number | Associated Pathway List | GO Number | Associated GO List |
| --- | --- | --- | --- | --- | --- | --- | --- |
| Gorai.001G019600 | FAP2 | Fatty-acid-binding protein 2 | NA | - | - | 2 | GO:0016872; GO:0042398; |
| Gorai.001G019800 | ERECTA | LRR receptor-like serine/threonine-protein kinase ERECTA | NA | - | - | 7 | GO:0004672; GO:0005515; GO:0005524; GO:0006468; GO:0004674; GO:0004713; GO:0016772; |
| Gorai.001G022800 | EDR1 | Serine/threonine-protein kinase EDR1 | K04424( MAPK signaling pathway) | 2 | ko04010; ko04530; | 6 | GO:0004672; GO:0005524; GO:0006468; GO:0004674; GO:0004713; GO:0016772; |
| Gorai.001G022900 | RTE1 | Protein REVERSION-TO-ETHYLENE SENSITIVITY1 | NA | - | - | NA | - |
| Gorai.001G027600 | EMF1 | Protein EMBRYONIC FLOWER 1 | NA | - | - | NA | - |
| Gorai.001G027700 | EMF1 | Protein EMBRYONIC FLOWER 1 | NA | - | - | NA | - |
| Gorai.001G050200 | HEX6 | Hexose carrier protein HEX6 | NA | - | - | 7 | GO:0005215; GO:0016020; GO:0016021; GO:0022857; GO:0022891; GO:0055085; GO:0006810; |
| Gorai.001G050400 | APS1 | Acid phosphatase 1 | NA | - | - | 1 | GO:0003993; |
| Gorai.001G050600 | At5g07610 | F-box protein At5g07610 | NA | - | - | 1 | GO:0005515; |
| Gorai.001G052100 | usb1 | U6 snRNA phosphodiesterase | NA | - | - | 2 | GO:0004518; GO:0034477; |
| Gorai.001G052200 | TMEM135 | Transmembrane protein 135 | NA | - | - | 1 | GO:0005515; |
| Gorai.001G052500 | PABN1 | Polyadenylate-binding protein 1 | K14396(polyadenylate-binding protein 2) | 2 | ko03015; ko05164; | 2 | GO:0000166; GO:0003676; |
| Gorai.001G059600 | rnj | Ribonuclease J | NA | - | - | 5 | GO:0003677; GO:0003723; GO:0046872; GO:0005515; GO:0016787; |
| Gorai.001G068700 | KAN1 | Transcription repressor KAN1 | NA | - | - | 2 | GO:0003677; GO:0005515; |
| Gorai.001G069100 | MRPL24 | 54S ribosomal protein L24, mitochondrial | K02902( large subunit ribosomal protein L28) | 2 | ko03010; M00178; | 4 | GO:0003735; GO:0005622; GO:0005840; GO:0006412; |
| Gorai.001G069200 | FKBP42 | Peptidyl-prolyl cis-trans isomerase FKBP42 | NA | - | - | 3 | GO:0005515; GO:0006457; GO:0005488; |
| Gorai.001G075400 | RTNLB9 | Reticulon-like protein B9 | NA | - | - | 1 | GO:0005783; |
| Gorai.001G087900 | GDU4 | Protein GLUTAMINE DUMPER 4 | NA | - | - | NA | - |
| Gorai.001G096100 | RPS27B | 40S ribosomal protein S27-2 | K02978(small subunit ribosomal protein S27e) | 3 | ko03010; M00177; M00179; | 4 | GO:0003735; GO:0005622; GO:0005840; GO:0006412; |
| Gorai.001G096200 | ACO | 1-aminocyclopropane-1-carboxylate oxidase | NA | - | - | NA | - |
| Gorai.001G096300 | ACO3 | 1-aminocyclopropane-1-carboxylate oxidase 3 | K05933(aminocyclopropanecarboxylate oxidase) | 4 | ko00270; ko01100; ko01110; M00368; | 3 | GO:0016491; GO:0055114; GO:0016706; |
| Gorai.001G105900 | At5g60050 | BTB/POZ domain-containing protein At5g60050 | NA | - | - | 1 | GO:0005515; |
| Gorai.001G106500 | RBOHH | Putative respiratory burst oxidase homolog protein H | NA | - | - | 10 | GO:0004601; GO:0005509; GO:0016020; GO:0016491; GO:0050664; GO:0055114; GO:0005506; GO:0009055; GO:0016021; GO:0050660; |
| Gorai.001G120100 | C12RT1 | Flavanone 7-O-glucoside 2''-O-beta-L-rhamnosyltransferase | NA | - | - | 2 | GO:0008152; GO:0016758; |
| Gorai.001G126600 | SCRL11 | Putative defensin-like protein 244 | NA | - | - | 1 | GO:0007165; |
| Gorai.001G130900 | PME11 | Putative pectinesterase 11 | K01051(Metabolic pathways) | 4 | ko00040; ko00500; ko01100; M00081; | 3 | GO:0005618; GO:0030599; GO:0042545; |
