# Supplementary figures and images for: Simple Sequence Repeat (SSR) Genetic Linkage Map of D Genome Diploid Cotton Derived from an Interspecific Cross between Gossypium davidsonii and Gossypium klotzschianum
Source: Int J Mol Sci. 2018 Jan 11;19(1):204. doi: 10.3390/ijms19010204 (PMC5796153; doi:10.3390/ijms19010204)

CHR01

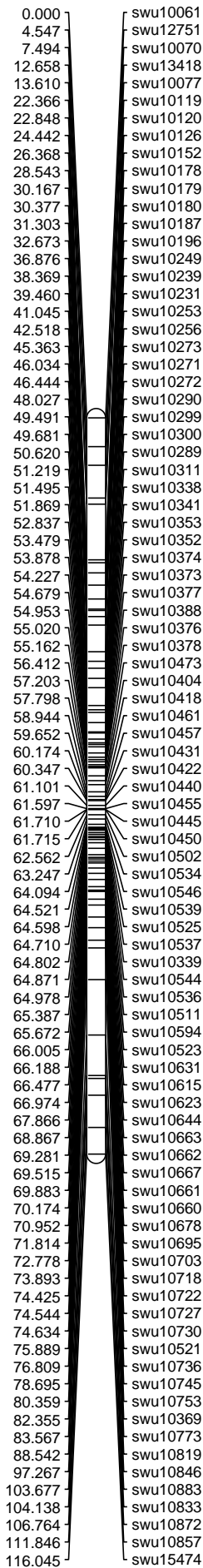

CHR02

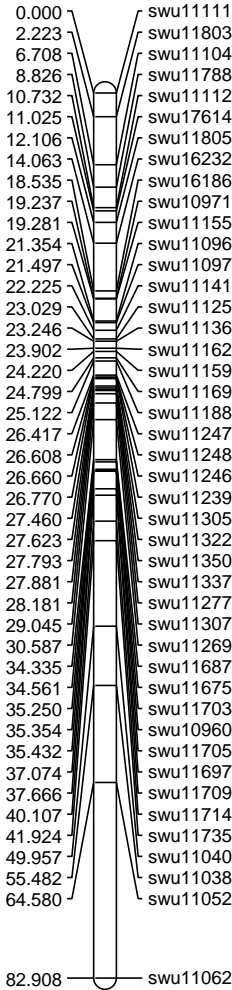

CHR03

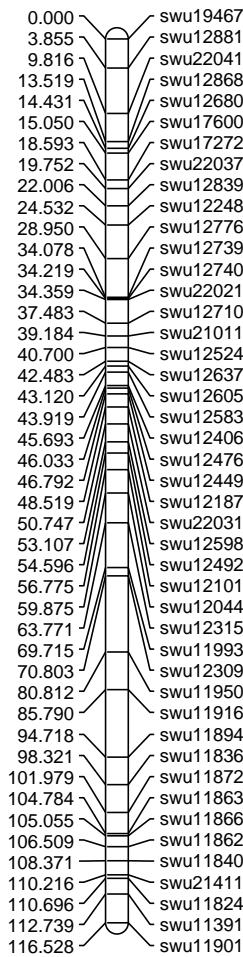

CHR04

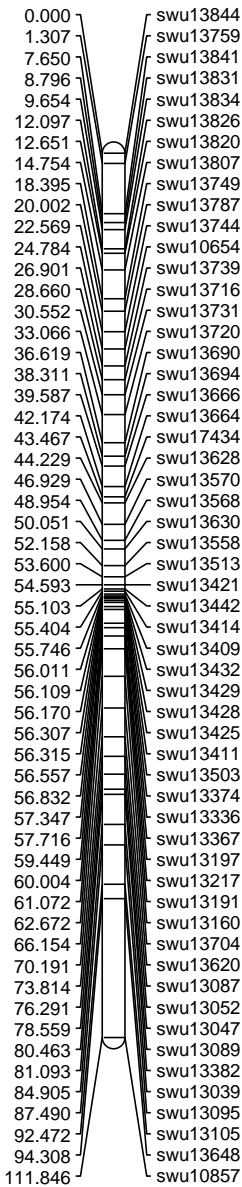

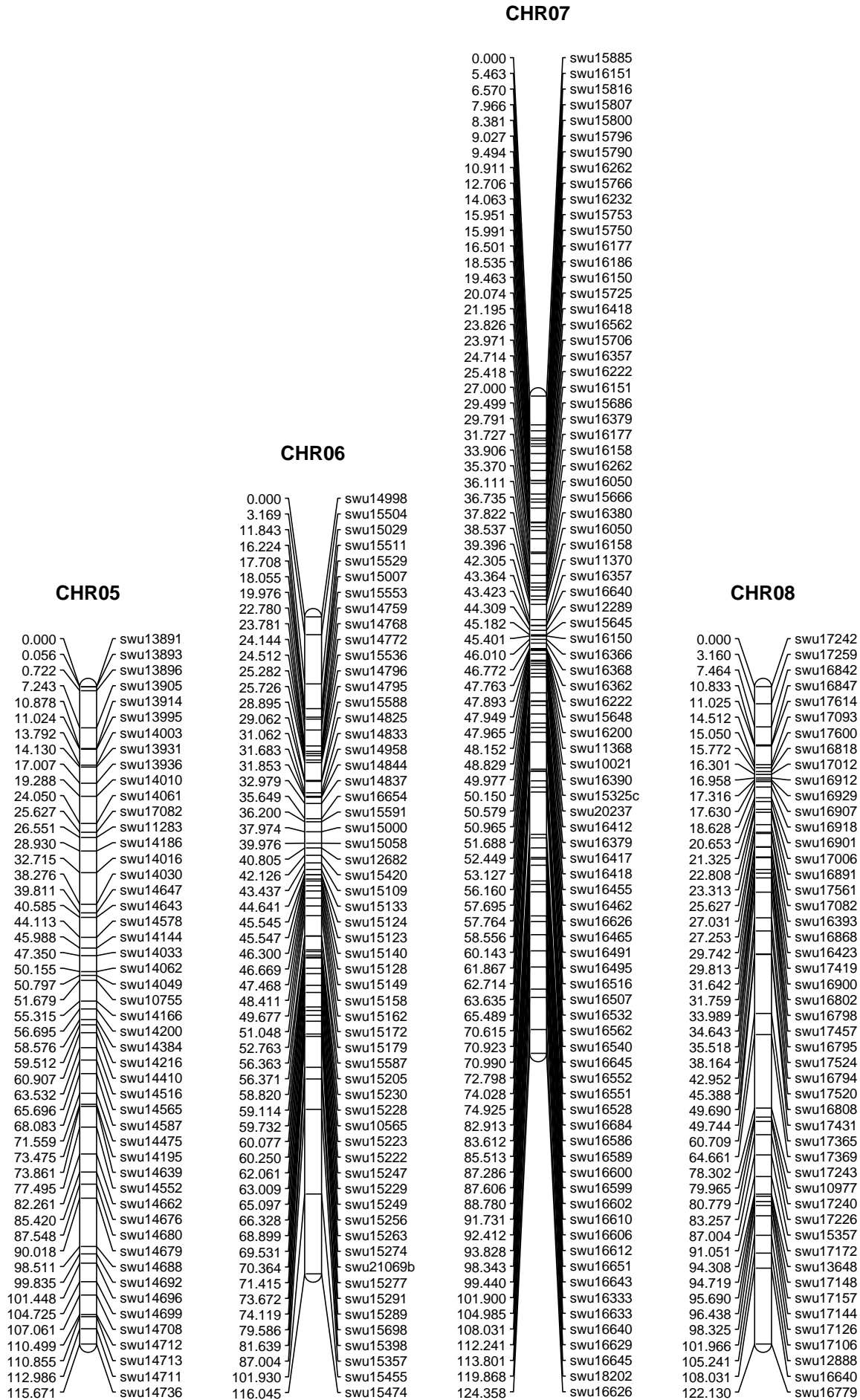

CHR09

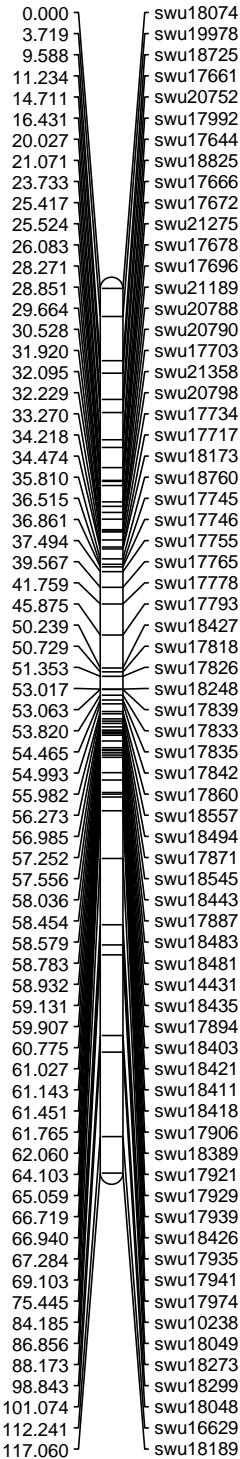

CHR11

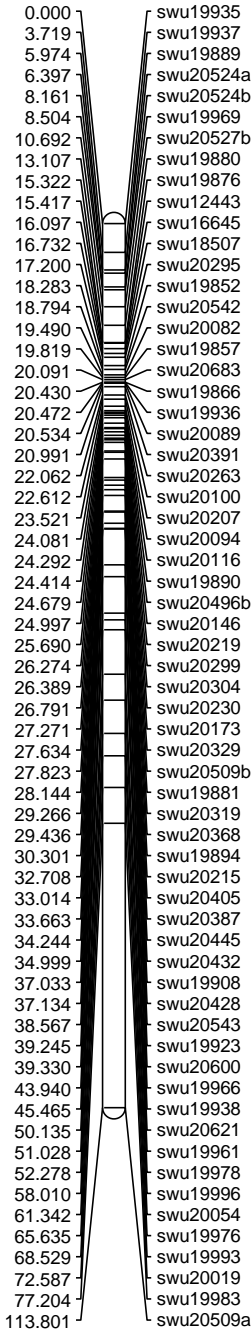

CHR10

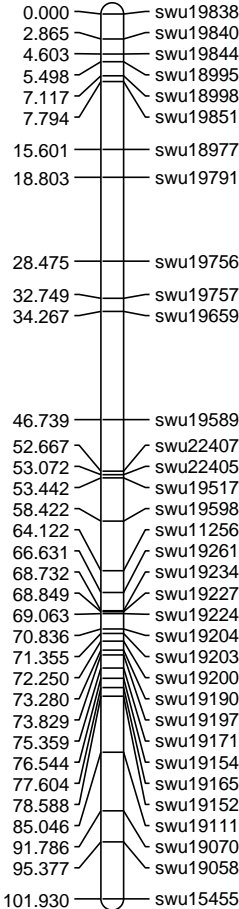

CHR12

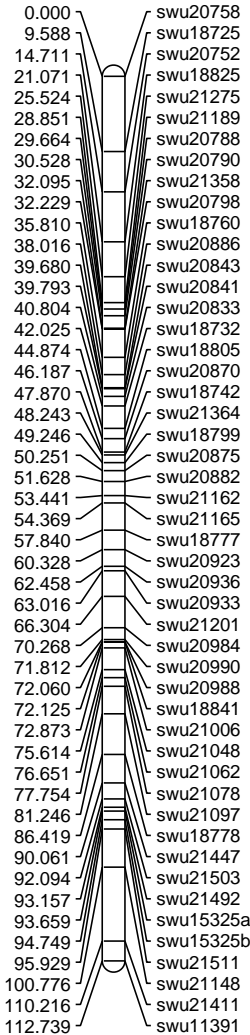

CHR13

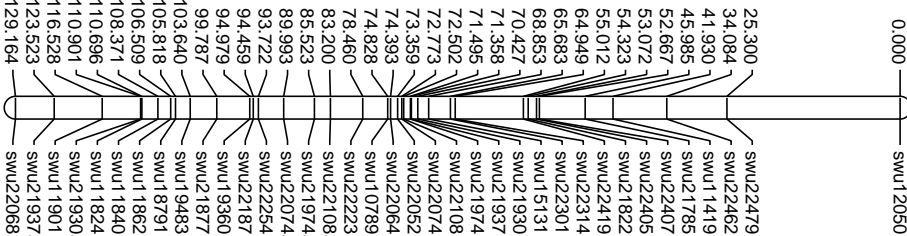

Supplement: Supplementary file 1 [file ijms-19-00204-s001.zip › ijms-254865 final supplementary/Supplementary Figure 1.pdf]
